# Supplementary material for: Vinyl cyclopropanes as a unifying platform for enantioselective remote difunctionalization of alkenes
Source: Nat Commun. 2025 Jul 29;16:6958. doi: 10.1038/s41467-025-61363-3 (PMC12307794; doi:10.1038/s41467-025-61363-3)
Supplement: Supplementary file 1 — Supplementary Information [file 41467_2025_61363_MOESM1_ESM.pdf]

Supplementary Information

Vinyl Cyclopropanes As A Unifying Platform for Enantioselective Remote Difunctionalization of Alkenes

Xiaoyong Du, Marc E. Lennon, Georgia Kriticou and Cristina Nevado[1]*

Department of Chemistry, University of Zurich, Winterthurerstrasse 190, CH 8057, Zurich, Switzerland.

Contents

1. General Information 2
2. Synthesis of Vinyl Cyclopropanes 3
3. Asymmetric Remote Difunctionalization of Vinyl Cyclopropanes via Nickel/Photoredox Dual Catalysis 13
4. Synthetic application 98
5. Mechanistic Studies 113
6. NMR spectra 123
7. X-ray Crystallographic Data 204
8. References 206

1. General Information

Unless otherwise stated, reactions were carried out using dry solvents under nitrogen atmosphere. Starting materials were purchased from Aldrich, Fluka and TCI. Pentane, hexane and ethyl acetate were purchased with HPLC quality, degassed by purging thoroughly with nitrogen and dried over activated molecular sieves of appropriate size. Tetrahydrofuran, acetonitrile and dichloromethane were dried using a solvent purification system (Pure Solv PS-MD-4EN, Innovative Technology Inc.) equipped with alumina drying columns under argon. Conversion was monitored by thin layer chromatography (TLC) using Merck TLC silica gel 60 F254 and visualized by UV-light at 254 nm. Flash column chromatography was performed over silica gel (230- 400 mesh). All NMR spectra were recorded on AV2-400 or AV2-500 MHz Bruker spectrometers. Chemical shifts are given in ppm and the spectra are calibrated using the residual chloroform signals (7.26 ppm for ^{1}H NMR and 77.0 ppm for ^{13}C NMR). Multiplicities are abbreviated as follows: singlet (s), doublet (d), triplet (t), quartet (q), doublet-doublet (dd), quintet (quint), septet (sept), multiplet (m), and broad (b). Infrared spectra were recorded on a JASCO FT/IR-4100 spectrometer. Absorptions are reported in wavenumber (cm-1). High-resolution electrospray ionization and electronic impact mass spectrometry were performed on a Finnigan MAT 900 (Thermo Finnigan, San Jose, CA; USA) double focusing magnetic sector mass spectrometer. Ten spectra were acquired. A mass accuracy ≤ 2 ppm was obtained in the peak matching acquisition mode by using a solution containing 2 <l PEG200, 2 <l PPG450, and 1.5 mg NaOAc (all obtained from Sigma-Aldrich, CH-Buchs) dissolved in 100 mL MeOH (HPLC Supra grade, Scharlau, E-Barcelona) as internal standard. Melting points were measured on melting point operators: MPM-MHV from Müller + Krempel AG. Enantiomeric ratios were determined by chiral HPLC analysis performed on JASCO HPLC system equipped with a PU-980 pump, a UV-970 detector, measured at 254 nm and a chiral column. Optical rotations were measured on a JASCO P-1010 polarimeter.

2. Synthesis of Vinyl Cyclopropanes

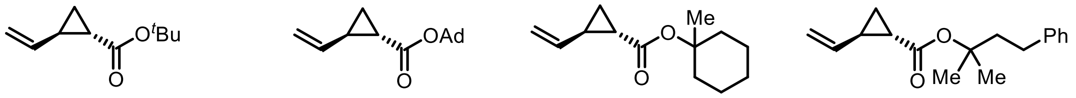

(±) *trans/cis* = 6/1 **VCP1** | (±) *trans/cis* = 10/1 **VCP2** | (±) *trans/cis* = 12/1 **VCP3** | (±) *trans/cis* = 16/1 **VCP4**

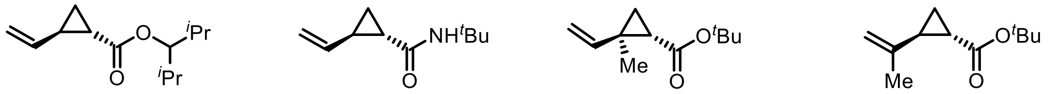

(±) *trans/cis* = 15/1 **VCP5** | (±) *trans/cis* = 5/1 **VCP6** | (±) *trans/cis* = 3/1 **VCP7** | (±) *trans/cis* > 20/1 **VCP8**

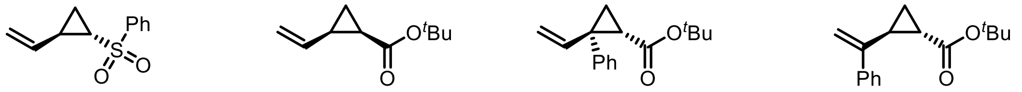

(±) *trans/cis* = 6/1 **VCP9** | (1*R*,2*R*) **VCP10** | (±) *trans/cis* > 20/1 **VCP11** | (±) *trans/cis* >20/1 **VCP12**

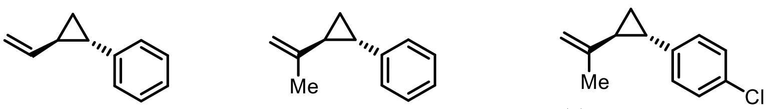

(±) *trans/cis* >20/1 **VCP13** | (±) *trans/cis* >20/1 **VCP14** | (±) *trans/cis* >20/1 **VCP15**

Cyclopropane substrates **VCP1**,[1] **VCP9**,[2] **VCP12**,[3] **VCP13**,[4] and **VCP14**[5] were prepared according to reported procedures and analytical data were in agreement with previously reported values. Cyclopropanes **VCP2**, **VCP3**, **VCP4**, **VCP5**, and **VCP6** were prepared using **GP1**. Cyclopropanes **VCP7** and **VCP8** were prepared using **GP2**. Cyclopropanes **VCP10** were prepared using **GP3**. Cyclopropane **VCP11** was prepared using **GP4**. Cyclopropane **VCP15** was prepared using **GP5.**

2.1 General procedure 1

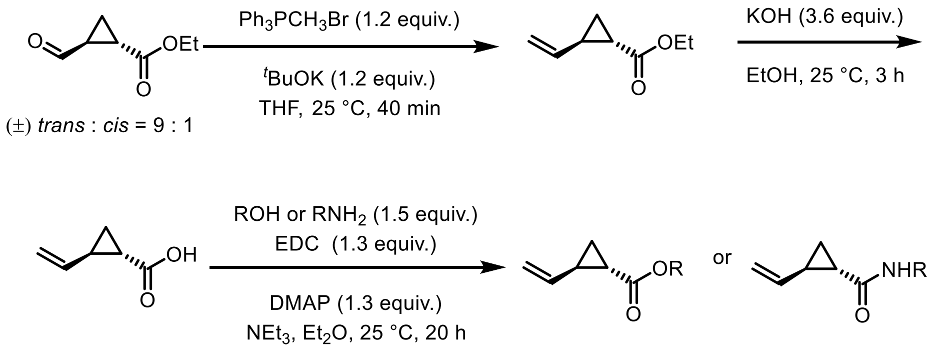

Following a modified procedure by DeLuca and Shibata *et al*,[1] an oven-dried 500 mL

round bottom flask equipped with a magnetic stirring bar was charged sequentially with methyltriphenylphosphonium bromide (8.6 g, 24 mmol, 1.2 equiv.) and dry THF (100 mL). Potassium *tert*-butoxide (2.7 g, 24 mmol, 1.2 equiv.) was added portion-wise and the mixture was stirred at 25 °C for 20 min. Then, a solution of ethyl 2-formylcyclopropane-1-carboxylate (2.8 g, 20 mmol, 1.0 equiv.) in THF (100 mL) was added dropwise via syringe. The resulting mixture was stirred at 25 °C for 30 min. The mixture was quenched with 10 mL of water and extracted with diethyl ether (3 × 100 mL). The combined organic layer was dried over $MgSO_4$. Upon concentration under reduced pressure (≥150 mbar, 25 °C), the ethyl 2-vinylcyclopropane-1-carboxylate was obtained in quantitative yield and used without further purification.

To the crude ethyl 2-vinylcyclopropane-1-carboxylate, a solution of potassium hydroxide (4.0 g, 72 mmol, 3.6 equiv.) in ethanol (20 mL) was added dropwise. The resulting mixture was stirred at 25 °C for 3 h (monitored by TLC) then the solvent was removed under reduced pressure. Water (100 mL) was added to the residue and the resulting solution extracted with DCM (3 × 30 mL). The organic washings were discarded while the water layer was acidified to pH= 2 using hydrochloric acid (2 M). The acidified aqueous layer was extracted with diethyl ether (3 × 100 mL). The organic layers were combined and dried over Na_2SO_4. Concentration under reduced pressure afforded the crude 2-vinylcyclopropane-1-carboxylic acid, which was used without further purification.

A solution of ROH or RNH_2 (30 mmol, 1.5 equiv.) in diethyl ether (100 mL) was added to the crude 2-vinylcyclopropane-1-carboxylic acid. The reaction mixture was stirred at 0 °C and triethylamine (8.3 mL, 60 mmol, 3.0 equiv.) was added dropwise via syringe. Then *N*-(3 dimethylaminopropyl)-*N'*-ethylcarbodiimide hydrochloride (EDC) (5.0 g, 26 mmol, 1.3 equiv.) and 4-dimethylaminopyridine (DMAP) (3.2 g, 26 mmol, 1.3 equiv.) were added portion-wise. After the addition was complete, the reaction mixture was stirred for 20 h at 25 °C, then diluted with diethyl ether (100 mL). The resulting solution was transferred to a separatory funnel, washed with water and brine, then dried over Na_2SO_4. After concentration under reduced pressure, the crude was purified by flash chromatography on silica gel, (pentane/Et_2O, 30:1), to give the desired products.

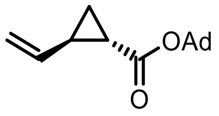

(3*s*,5*s*,7*s*)-Adamantan-1-yl 2-vinylcyclopropane-1-carboxylate (VCP2). Prepared by **GP1** at 2.68 mmol scale. Colourless oil, 181 mg, 27% yield, *trans* : *cis* = 10 : 1. ^{1}H NMR (400 MHz, $CDCl_3$) δ 5.38 (ddd, *J* = 17.0, 10.2, 8.4 Hz, 1H), 5.15 (dd, *J* = 17.0, 1.6 Hz, 1H), 4.97 (dd, *J* = 10.2, 1.6 Hz, 1H), 2.21 – 2.07 (m, 9H), 1.94 (dd, *J* = 6.3, 4.0 Hz, 1H), 1.65 (dt, *J* = 2.9, 1.6 Hz, 6H), 1.55 (ddd, *J* = 8.3, 5.1, 3.9 Hz, 1H), 1.31 – 1.25 (m, 1H), 0.89 (ddd, *J* = 8.4, 6.1, 4.3 Hz, 1H). ^{13}C NMR (101 MHz, $CDCl_3$) δ 172.5, 138.7, 114.5, 80.7, 41.5, 36.3, 31.0, 25.3, 23.1, 15.5. IR (film): ν (cm^{-1}) 2909, 2852, 1717, 1635, 1384, 1172, 1057, 897. HR-MS (ESI) calculated $[M+H]^+$ for $C_{16}H_{23}O_2^+$ = 247.16926, found: 247.16914.

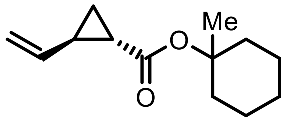

1-Methylcyclohexyl 2-vinylcyclopropane-1-carboxylate (VCP3). Prepared by **GP1** at 2.68 mmol scale. Colourless oil, 125 mg, 23% yield; *trans* : *cis* = 12 : 1. ^{1}H NMR (400 MHz, $CDCl_3$) δ 5.46 – 5.34 (m, 1H), 5.16 (ddd, *J* = 17.0, 1.6, 0.6 Hz, 1H), 4.97 (dd, *J* = 10.3, 1.6 Hz, 1H), 2.17 – 2.06 (m, 2H), 1.97 (tdd, *J* = 8.4, 6.1, 4.0 Hz, 1H), 1.61 – 1.44 (m, 9H), 1.43 – 1.35 (m, 2H), 1.33 – 1.23 (m, 3H), 0.91 (ddd, *J* = 8.4, 6.1, 4.3 Hz, 1H). ^{13}C NMR (101 MHz, $CDCl_3$) δ 172.5, 138.7, 114.5, 82.0, 36.9, 36.8, 25.7, 25.5, 25.2, 23.2, 22.2, 15.4 (one peak overlapping with others). IR (film): ν (cm^{-1}) 3004, 2931, 1737, 1717, 1447, 1376, 1201, 1147. HR-MS (ESI) calculated $[M+Na]^+$ for $C_{13}H_{20}O_2\,^{23}Na^+$ = 231.13555, found: 231.13571.

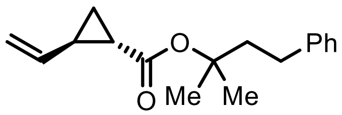

2-Methyl-4-phenylbutan-2-yl 2-vinylcyclopropane-1-carboxylate (VCP4). Prepared by **GP1** at 3 mmol scale. Colourless oil, 164 mg, 21% yield; *trans* : *cis* = 16 : 1. ^{1}H NMR (400 MHz, $CDCl_3$) δ 7.23 – 7.18 (m, 2H), 7.12 (d, *J* = 7.3 Hz, 3H), 5.33 (ddd, *J* = 17.0, 10.2, 8.4 Hz, 1H), 5.14 – 5.06 (m, 1H), 4.91 (dd, *J* = 10.3, 1.6 Hz, 1H), 2.62 – 2.51 (m, 2H), 2.03 – 1.95 (m, 2H), 1.90 (ddd, *J* = 8.5, 3.9, 2.4 Hz, 1H), 1.53 – 1.49 (m, 1H), 1.43 (s, 6H), 1.24 (ddd, *J* =

8.9, 5.2, 4.2 Hz, 1H), 0.85 (ddd, *J* = 8.3, 6.1, 4.3 Hz, 1H). ^{13}C NMR (101 MHz, $CDCl_3$) δ 172.7, 142.3, 138.6, 128.5, 128.5, 125.9, 114.6, 82.3, 43.0, 30.5, 26.3, 26.3, 25.3, 23.0, 15.5. IR (film) ν (cm^{-1}) 3027, 3004, 2977, 2933, 1717, 1638, 1605, 1495, 1472, 1454, 1386, 1368, 1327, 1285, 1249, 1197, 1181, 1163, 1120, 1089, 1073, 1045, 1031, 985, 904, 877, 851, 819, 762, 740, 698, 652, 590, 520, 499. HR-MS (ESI) m/z calcd for $C_{17}H_{22}NaO_2^+$ 281.15120, found 281.15109, [M+Na$^+$].

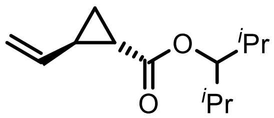

2,4-Dimethylpentan-3-yl 2-vinylcyclopropane-1-carboxylate (VCP5). Prepared by **GP1** at 3 mmol scale. Colourless oil, 395 mg, 63% yield; *trans* : *cis* = 15 : 1. ^{1}H NMR (400 MHz, $CDCl_3$) δ 5.41 (ddd, *J* = 17.1, 10.3, 8.4 Hz, 1H), 5.16 (dd, *J* = 17.1, 2.3 Hz, 1H), 4.98 (dd, *J* = 10.3, 1.6 Hz, 1H), 4.58 (t, *J* = 6.2 Hz, 1H), 2.07 – 1.99 (m, 1H), 1.89 (dqd, *J* = 13.3, 6.8, 1.6 Hz, 2H), 1.67 (ddd, *J* = 8.4, 5.2, 3.9 Hz, 1H), 1.40 – 1.34 (m, 1H), 0.97 (ddd, *J* = 8.4, 6.2, 4.3 Hz, 1H), 0.92 – 0.81 (m, 12H). ^{13}C NMR (101 MHz, $CDCl_3$) δ 173.5, 138.4, 114.7, 82.8, 29.6, 29.6, 25.3, 22.2, 19.7, 19.6, 17.4, 17.3, 15.5. IR (film) ν (cm^{-1}) 2965, 2935, 2876, 1723, 1638, 1465, 1449, 1392, 1370, 1302, 1284, 1264, 1199, 1174, 1133, 1097, 1008, 986, 935, 902, 878, 851, 826, 737, 653. HR-MS (ESI) m/z calcd for $C_{13}H_{22}NaO_2^+$ 233.15120, found 233.15108, [M+Na$^+$].

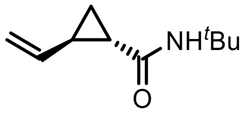

***N*-(*tert*-Butyl)-2-vinylcyclopropane-1-carboxamide (VCP6).** Prepared by **GP1** at 3.12 mmol scale. Colorless oil, 361 mg, 69% yield; *trans* : *cis* = 5 : 1. ^{1}H NMR (400 MHz, $CDCl_3$) δ 5.39 (ddd, *J* = 17.0, 10.2, 8.5 Hz, 2H), 5.14 (ddd, *J* = 17.0, 1.6, 0.7 Hz, 1H), 4.95 (dd, *J* = 10.3, 2.0 Hz, 1H), 1.94 (tdd, *J* = 8.6, 6.1, 4.1 Hz, 1H), 1.34 (d, *J* = 4.6 Hz, 9H), 1.32 – 1.23 (m, 2H), 0.81 (ddd, *J* = 8.0, 6.1, 4.0 Hz, 1H). ^{13}C NMR (101 MHz, $CDCl_3$) δ 171.1, 139.2, 114.2, 51.5, 29.1, 24.9, 24.1, 14.4. IR (film): ν (cm^{-1}) 3373, 2930, 1727, 1685, 1603, 1367, 734. HR-MS (ESI) calculated [M+H]$^+$ for $C_{10}H_{18}ON^+$ = 168.13829, found: 168.13814.

2.2 General procedure 2

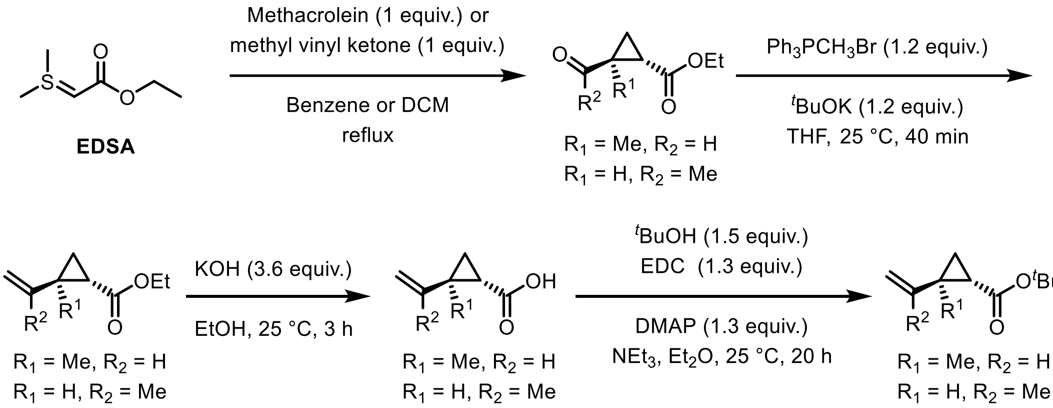

Ethyl (dimethylsulfuranylidene)acetate (**EDSA**) was prepared in accordance with the literature[6] and was used immediately in the subsequent step. Following a modified procedure from G. B. Payne,[6] to a solution of methacrolein (2.7 mL, 32 mmol, 1.0 equiv.) in benzene (8.0 mL) was slowly added a solution of EDSA (4.7 g, 32 mmol, 1.0 equiv.) in benzene (8.0 mL). The reaction mixture was warmed to 45 °C for 30 minutes and then heated to reflux for 1 h. Alternatively, to a solution of methyl vinyl ketone (2.6 mL, 32 mmol, 1.0 equiv.) in DCM (8.0 mL) was slowly added a solution of EDSA (4.7 g, 32 mmol, 1.0 equiv.) in DCM (8.0 mL). The reaction mixture was then heated to reflux for 2.5 h.

In both cases, the solvent was removed under reduced pressure and the corresponding crude used without further purification in the subsequent steps, as outlined in **GP1**.

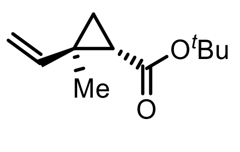

***tert*-Butyl 2-methyl-2-vinylcyclopropane-1-carboxylate (VCP7).** Colourless oil, 1.04 g, 50% yield; *trans* : *cis* = 3 : 1. ^{1}H NMR (400 MHz, $CDCl_3$) δ 5.42 (dd, J = 17.2, 10.5 Hz, 1H), 5.07 – 4.95 (m, 2H), 1.65 (dd, J = 8.2, 6.0 Hz, 1H), 1.45 (s, 9H), 1.30 (s, 3H), 1.26 – 1.22 (m, 1H), 1.04 – 0.98 (m, 1H). ^{13}C NMR (101 MHz, $CDCl_3$) δ 171.0, 144.8, 111.8, 80.5, 28.4, 27.5, 21.9, 20.7, 14.4. IR (film): ν (cm^{-1}) 2981, 1718, 1366, 1145, 748, 471. HR-MS (ESI) calculated

[M+Na]$^+$ for $C_{11}H_{18}O_2{}^{23}Na^+$ =205.11990, found: 205.12004.

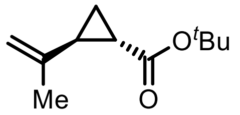

***tert*-Butyl 2-(prop-1-en-2-yl)cyclopropane-1-carboxylate (VCP8).** Colourless oil, 1.07 g, 54% yield; *trans* : *cis* > 20 : 1. ^{1}H NMR (400 MHz, $CDCl_3$) δ 4.80 – 4.72 (m, 2H), 1.99 – 1.90 (m, 1H), 1.64 (dd, J = 1.5, 0.9 Hz, 3H), 1.64 – 1.59 (m, 1H), 1.45 (s, 9H), 1.20 (ddd, J = 9.1, 5.1, 4.2 Hz, 1H), 1.00 (ddd, J = 8.4, 6.6, 4.3 Hz, 1H). ^{13}C NMR (101 MHz, $CDCl_3$) δ 173.2, 143.5, 110.5, 28.3, 28.0, 21.7, 20.3, 14.1 (one peak overlapping with others). IR (film): ν (cm^{-1}) 2977, 1719, 1367, 1148, 844, 748. HR-MS (ESI) calculated [M+Na]$^+$ for $C_{11}H_{18}O_2{}^{23}Na^+$ = 205.11990, found: 205.12000.

2.3 General procedure 3

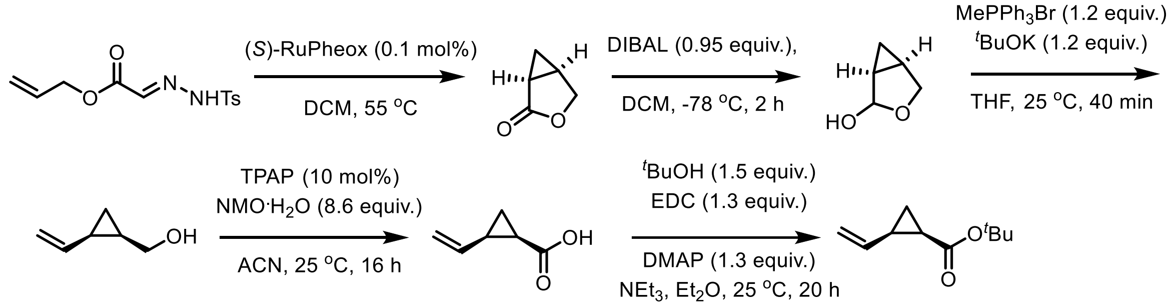

Allyl (*E*)-2-(2-tosylhydrazineylidene)acetate was prepared according to literature.[7]

A solution of allyl (*E*)-2-(2-tosylhydrazineylidene)acetate (6.7 g, 53 mmol, 1 equiv.) in DCM (70 mL) was added dropwise, at a rate of 2 mL/hr, to a suspension of the (*S*)-RuPheox catalyst (34 mg, 0.053 mmol,0.1 mol%) in DCM (100 mL) which was heated to 55 °C. The reaction was stirred for an additional 30 min after dropwise addition has been completed. The resulting solution was filtered through a pad of silica (2 cm) and the pad was washed with additional DCM (3 x 5 mL). The filtrate was concentrated under reduced pressure to afford the lactone, (1*S*,5*R*)-3-oxabicyclo[3.1.0]hexan-2-one, as a colourless oil (4.7 g, 89% yield).

DIBAL (1 M in DCM, 36 mL, 36 mmol, 0.95 equiv.) was added dropwise to a solution of lactone, (1*S*,5*R*)-3-oxabicyclo[3.1.0]hexan-2-one (3.7 g, 38 mmol, 1.0 equiv.), in DCM (75 mL)

at −78 °C. The reaction was monitored by TLC and once the lactone was consumed, the reaction mixture was quenched with a saturated solution of Rochelle salt (60 mL), and stirred until the two phases became clear. The phases were separated, and the aqueous phase extracted twice with Et_2O (3 x 15 mL). The combined organic phase was dried over $MgSO_4$ and concentrated under reduced pressure to give the corresponding lactol, (1*S*,5*R*)-3-oxabicyclo[3.1.0]hexan-2-ol, which was used directly in the subsequent step.

Methyltriphenylphosphonium bromide (16 g, 46 mmol, 1.2 equiv.) and dry THF (100 mL) were charged into an oven-dried flask. Potassium *tert*-butoxide (5.1 g, 46 mmol, 1.2 equiv.) was added portion-wise and the mixture was stirred at 25 °C for 20 min. Then, a solution of (1*S*,5*R*)-3-oxabicyclo[3.1.0]hexan-2-ol (3.8 g, 38 mmol, 1.0 equiv.) in THF (100 mL) was added dropwise via syringe. The resulting mixture was stirred at 25 °C for 30 min. The mixture was quenched with 10 mL of water and extracted with diethyl ether (3 × 100 mL). The combined organic layer was dried over $MgSO_4$. Upon concentration under reduced pressure (≥150 mbar, 25 °C), the residue was purified by silica gel column chromatography (DCM/Et_2O, 1:10) to afford the ((1*R*,2*R*)-2-vinylcyclopropyl)methanol (1.9 g, 51%) as a colourless oil.

((1*R*,2*R*)-2-vinylcyclopropyl)methanol (1.9 g, 19 mmol, 1.0 equiv.) and *N*-methylmorpholine-*N*-oxide monohydrate (20 g, 170.0 mmol, 8.6 equiv.) were dissolved in acetonitrile (75 mL). Tetra-*n*-propylammonium perruthenate (680 mg, 1.9 mmol, 10 mol%) was added portion-wise (1 mg/min) at 25 °C. The mixture was stirred at 25 °C overnight and was then quenched by the addition of an excess of isopropyl alcohol. Water (30 mL) was added to the reaction mixture, which was adjusted carefully to pH 1 by the addition of 2 M HCl. The aqueous phase was extracted with diethyl ether (3 × 50 mL), and the combined organic extracts were then washed with brine (20 mL), dried over $MgSO_4$, and concentrated under reduced pressure. The residue was purified by silica gel column chromatography (petroleum ether/ethyl acetate, 1:1) to afford the (1*R*,2*R*)-2-vinylcyclopropane-1-carboxylic acid (620 mg, 29%) as a brown oil. The (1*R*,2*R*)-2-vinylcyclopropane-1-carboxylic acid ^{1}H-NMR matched previously reported literature.[8]

Esterification of the carboxylic acid was performed as described in **GP1** to afford the final compound *tert*-butyl (1*R*,2*R*)-2-vinylcyclopropane-1-carboxylate (**VCP10**).

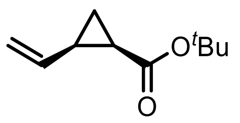

***tert*-Butyl (1*R*, 2*R*)-2-vinylcyclopropane-1-carboxylate (VCP10).** Colourless oil, 392 mg, 6% yield. $[\alpha]_D^{24}$ = -133.81 (c = 0.27, $CHCl_3$). ^1H NMR (400 MHz, $CDCl_3$) δ 5.75 (ddd, *J* = 17.1, 10.2, 8.6 Hz, 1H), 5.21 (ddd, *J* = 17.1, 1.9, 0.6 Hz, 1H), 5.03 (ddd, *J* = 10.3, 1.9, 0.6 Hz, 1H), 1.90 – 1.79 (m, 2H), 1.44 (s, 9H), 1.21 – 1.09 (m, 2H). ^{13}C NMR (101 MHz, $CDCl_3$) δ 171.2, 135.7, 115.9, 80.6, 28.4, 24.6, 22.2, 13.7. IR (film): ν (cm^{-1}) 2970, 1721, 1438, 1366, 1146, 903. HR-MS (ESI) calculated $[M+Na]^+$ for $C_{10}H_{16}O_2{}^{23}Na^+$ = 191.10425, found: 191.10498.

2.4 General procedure 4

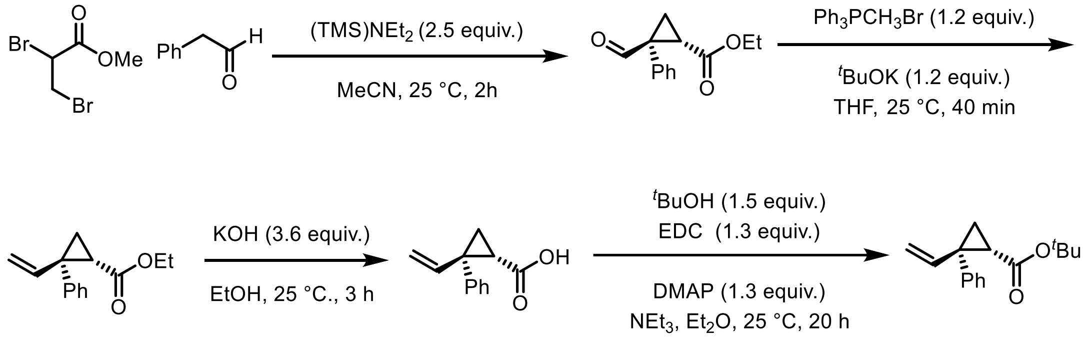

To a solution of phenylacetaldehyde (4.7 mL, 40 mmol, 1.0 equiv.) and methyl 2,3-dibromopropionate (5.1 mL, 40 mmol, 1.0 equiv.) in acetonitrile (5 mL) was added a solution of *N,N*-diethylaminotrimethylsilane (18.9 mL, 100 mmol, 2.5 equiv.) in acetonitrile (5 mL). The reaction mixture was stirred for 2 h at 25 °C. The crude was diluted with DCM (50 mL) and washed with distilled water (3 x 50 mL) and brine (1 x 50 mL). The organic layer was dried using $MgSO_4$ and the solvent was removed under reduced pressure. The crude mixture was purified by column chromatography on silica gel (pentane/diethyl ether, 5:1) to afford ethyl (1*S*,2*S*)-2-formyl-2-phenylcyclopropane-1-carboxylate (1.40 g, 31%) as a brown oil. The remaining steps were performed as outlined in **GP1** to afford compound final compound *tert*-butyl (1*S*,2*S*)-2-phenyl-2-vinylcyclopropane-1-carboxylate (**VCP11**).

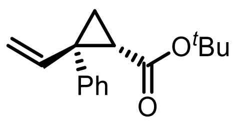

***tert*-Butyl 2-phenyl-2-vinylcyclopropane-1-carboxylate (VCP11).** Colourless oil, 250 mg, 3% yield; *trans* : *cis* > 20 : 1. ^{1}H NMR (400 MHz, $CDCl_3$) δ 7.28 – 7.25 (m, 4H), 7.22 – 7.18 (m, 1H), 6.01 (dd, *J* = 17.1, 10.5 Hz, 1H), 5.01 (dd, *J* = 10.6, 1.6 Hz, 1H), 4.57 (dd, *J* = 17.2, 1.6 Hz, 1H), 2.13 (dd, *J* = 8.2, 6.3 Hz, 1H), 1.64 (dd, *J* = 6.3, 4.8 Hz, 1H), 1.43 (s, 9H), 1.43 – 1.39 (m, 1H). ^{13}C NMR (101 MHz, $CDCl_3$) δ 170.6, 142.3, 139.0, 130.1, 128.5, 127.1, 116.5, 80.8, 37.8, 30.0, 28.4, 20.1. IR (film): ν (cm^{-1}) 2976, 2928, 1726, 1366, 1147, 700. HR-MS (ESI) calculated $[M+Na]^+$ for $C_{16}H_{20}O_2\,^{23}Na^+$ = 269.13555, found: 267.13531.

2.4 General procedure 5

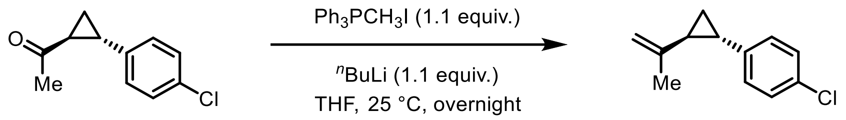

In an oven-dried 250 mL 2-necked round-bottom flask, methyltriphenylphosphonium iodide (2.22 g, 5.5 mmol, 1.1 equiv.) was suspended in 50 mL dry THF, and the mixture was cooled to 0 °C. A 2.5 M solution of *n*-butyllithium in hexanes was added dropwise until a persistent yellow colour was observed (2.2 mL, 5.5 mmol, 1.1 equiv.) and the resulting mixture was allowed to warm to 25 °C over 30 mins. 1-[2-(4-Chlorophenyl)cyclopropyl]ethenone[9] (970 mg, 5 mmol, 1.0 equiv.) was added dropwise as a solution in 10 mL dry THF. The reaction was allowed to stir overnight, quenched with methanol and then water, and diluted with diethyl ether. The phases were separated, and the aqueous phase extracted with further portions of ether. The combined organics were dried over $MgSO_4$, filtered, and the filtrate concentrated under reduced pressure (>50 mbar). The residue was purified by flash column chromatography on silica gel, eluting with pentane/ether (30/1), to afford the title compound as a colourless oil (727 mg, 75%).

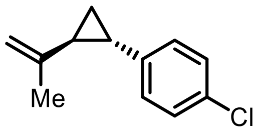

1-Chloro-4-((1*S*,2*R*)-2-vinylcyclopropyl)benzene (VCP15). ^{1}H NMR (500 MHz, $CDCl_3$) δ 7.26 – 7.21 (m, 1H), 7.05 – 7.00 (m, 1H), 4.81 – 4.72 (m, 1H), 1.97 (ddd, *J* = 8.8, 5.4, 5.0 Hz, 1H), 1.74 (s, 1H), 1.65 (ddd, *J* = 8.8, 5.9, 4.6 Hz, 1H), 1.25 (ddd, *J* = 8.8, 5.9, 5.0 Hz, 1H), 1.07 (ddd, *J* = 8.8, 5.4, 4.6 Hz, 1H). ^{13}C NMR (126 MHz, $CDCl_3$) δ 145.06, 141.57, 131.27, 128.49, 127.26, 109.02, 30.26, 23.58, 20.83, 15.35. IR (film) ν (cm^{-1}) 3079, 2971, 1738 (s, C=O), 1635, 1495, 1453, 1412, 1371, 1295, 1217, 1180, 1112, 1091, 1041, 1012, 953, 924, 878, 817, 776, 749, 711, 643, 513. HR-MS (EI) m/z calcd for $C_{11}H_{13}Cl^{+}$ 192.07003, found 192.06955, [M^{+}].

3. Asymmetric Remote Difunctionalization of Vinyl Cyclopropanes via Nickel/Photoredox Dual Catalysis

3.1 Optimization

Table S1. Ligand screening[a]

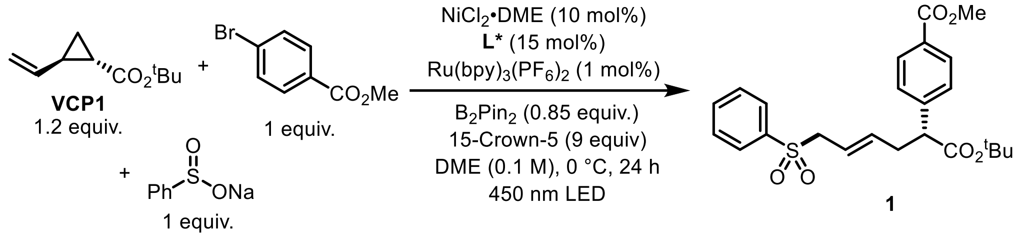

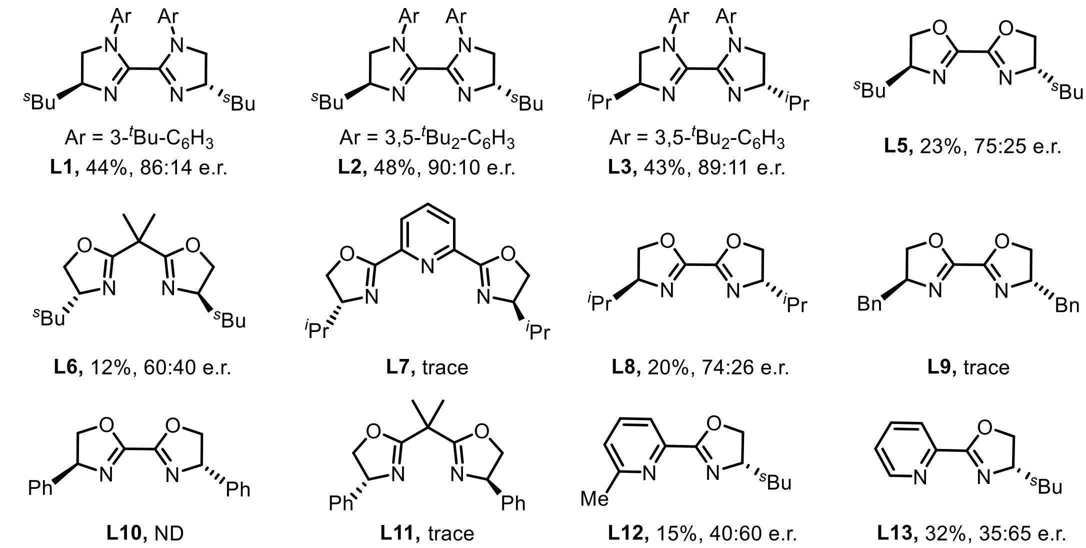

[a]Reactions were carried out with **VCP1** (0.12 mmol), methyl 4-bromobenzoate (0.1 mmol), $PhSO_2Na$ (0.1 mmol), $NiCl_2$·DME (10 mol%), **L*** (15 mol%), $Ru(bpy)_3(PF_6)_2$ (1 mol%), 15-crown-5 (9 equiv.), B_2Pin_2 (0.85 equiv.), DME (0.1 M), 450 nm 34 W EvoluChem LED, 0 °C, N_2, 24 h. ^{1}H NMR yield with mesitylene as internal standard. Enantiomeric ratios (e.r.) were determined by HPLC with a chiral stationary phase.

Table S2. Temperature screening[a]

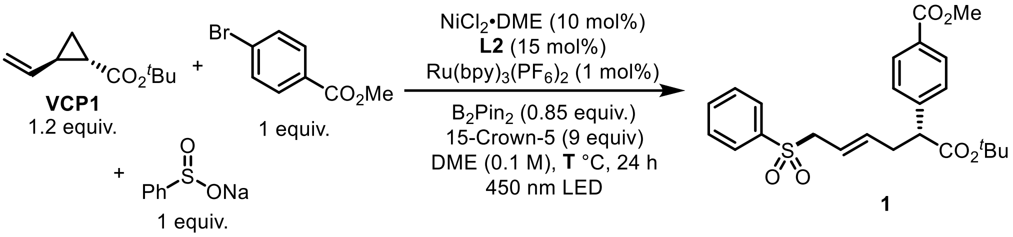

Entry	T [°C]	Yield [%]	e.r.
1	r.t.	56	86:14
2	0	48	90:10
3	-5	42	90:10
4	-10	43	89:11
5	-20	35	88:12

[a]Reactions were carried out with **VCP1** (0.12 mmol), methyl 4-bromobenzoate (0.1 mmol), $PhSO_2Na$ (0.1 mmol), $NiCl_2$·DME (10 mol%), **L2** (15 mol%), $Ru(bpy)_3(PF_6)_2$ (1 mol%), 15-crown-5 (9 equiv.), B_2Pin_2 (0.85 equiv.), DME (0.1 M), 450 nm 34 W EvoluChem LED, N_2, 24 h. ^{1}H NMR yield with mesitylene as internal standard. Enantiomeric ratios (e.r.) were determined by HPLC with a chiral stationary phase.

Table S3. Solvent screening[a]

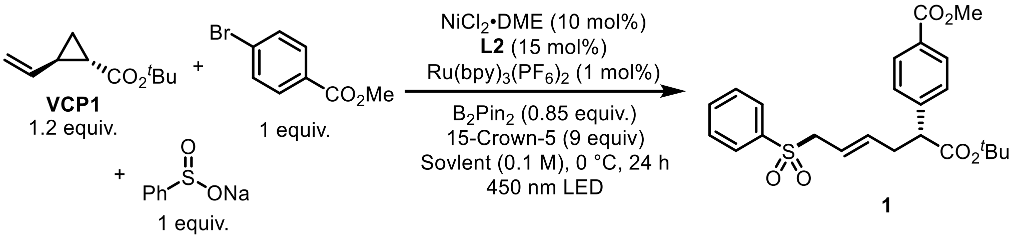

Entry	Solvent	Yield [%]	e.r.
1	DME	48	90:10
2	CH_3CN	47	84:16
3	THF	58	88:12
4	Acetone	55	85:15
5	EA	50	87:13
6	DMSO	60	70:30
7	DMA	57	72:28

[a]Reactions were carried out with **VCP1** (0.12 mmol), methyl 4-bromobenzoate (0.1 mmol), $PhSO_2Na$ (0.1 mmol), $NiCl_2$·DME (10 mol%), **L2** (15 mol%), $Ru(bpy)_3(PF_6)_2$ (1 mol%), 15-crown-5 (9 equiv.), B_2Pin_2 (0.85 equiv.), solvent (0.1 M), 450 nm 34 W EvoluChem LED, 0 °C, N_2, 24 h. ^{1}H NMR yield with mesitylene as internal standard. Enantiomeric ratios (e.r.) were

determined by HPLC with a chiral stationary phase.

Table S4. Concentration and nickel precursors screening[a]

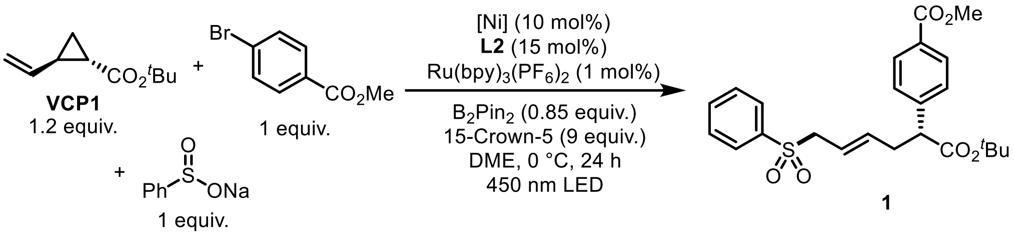

Entry	Concentration [M]	[Ni]	Yield [%]	e.r.
1	0.1	$NiCl_2$•DME	48	90:10
2	0.05	$NiCl_2$•DME	42	92:8
3	0.025	$NiCl_2$•DME	40	93:7
4	0.025	$NiBr_2$•DME	42	93:7
5	0.025	$Ni(COD)_2$	trace	--
6[b]	0.025	**L2**•$NiBr_2$	51	95:5

[a]Reactions were carried out with **VCP1** (0.12 mmol), methyl 4-bromobenzoate (0.1 mmol), $PhSO_2Na$ (0.1 mmol), [Ni] (10 mol%), **L2** (15 mol%), $Ru(bpy)_3(PF_6)_2$ (1 mol%), 15-crown-5 (9 equiv.), B_2Pin_2 (0.85 equiv.), DME, 450 nm 34 W EvoluChem LED, 0 °C, N_2, 24 h. ^{1}H NMR yield with mesitylene as internal standard. Enantiomeric ratios (e.r.) were determined by HPLC with a chiral stationary phase. [b]Using 10 mol% of complex **L2**·$NiBr_2$.

Table S5. Additive screening[a]

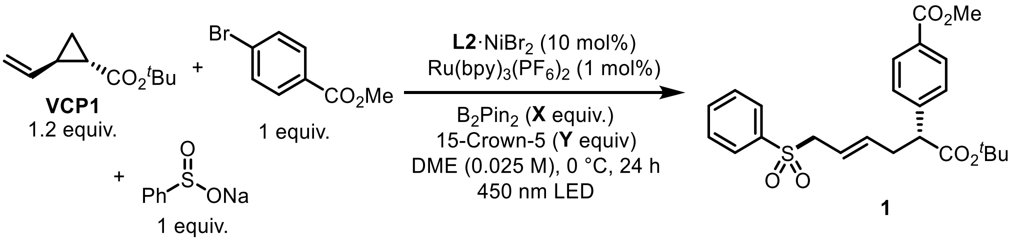

Entry	X (equiv.)	Y (equiv.)	Yield [%]	e.r.
1	0.85	9	51	95:5
2	1.5	9	10	93:7
3	0.5	9	58	95:5
4	0.1	9	58	95:5
5	0	9	13	95:5
6[b]	0.1	9	65	95:5
7[b]	0.1	3	72(70)[c]	95:5
8[b]	0.1	1	53	95:5

[a]Reactions were carried out with **VCP1** (0.12 mmol), methyl 4-bromobenzoate (0.1 mmol),

PhSO2Na (0.1 mmol), **L2**·NiBr2 (10 mol%), Ru(bpy)3(PF6)2 (1 mol%), B2Pin2 (X equiv.), 15-crown-5 (Y equiv.), DME (0.025 M), 450 nm 34 W EvoluChem LED, 0 °C, N2, 24 h. ^{1}H NMR yield with mesitylene as internal standard. Enantiomeric ratios (e.r.) were determined by HPLC with a chiral stationary phase. [b]48 h. [c]Isolated yield.

Table S6. Control experiments[a]

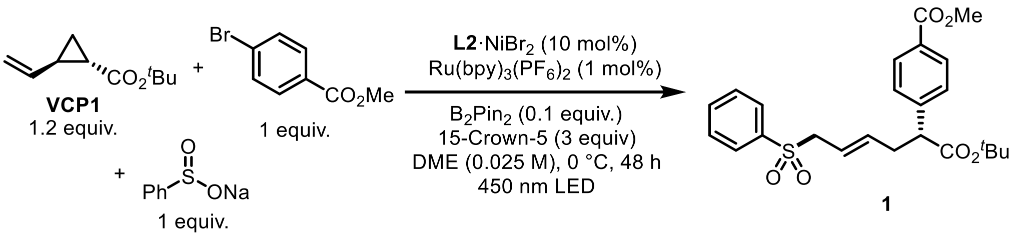

Entry	Reaction component omitted	Yield of **1** [%]	e.r.
1	none	72	95:5
2	nickel	0	--
3	photocatalyst	0	--
4	light	0	--

[a]Reactions were carried out with **VCP1** (0.12 mmol), methyl 4-bromobenzoate (0.1 mmol), $PhSO_2Na$ (0.1 mmol), **L2**·$NiBr_2$ (10 mol%), $Ru(bpy)_3(PF_6)_2$ (1 mol%), B_2Pin_2 (0.1 equiv.), 15-crown-5 (3 equiv.), DME (0.025 M), 450 nm 34 W EvoluChem LED, 0 °C, N_2, 48 h. ^{1}H NMR yield with mesitylene as internal standard. Enantiomeric ratio (e.r.) were determined by HPLC with a chiral stationary phase.

Table S7. Optimization for asymmetric 1, 5-dicarbofunctionalization of alkenes[a]

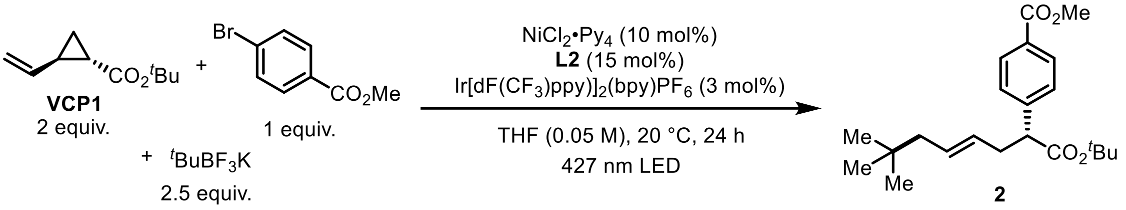

Entry	Deviations from standard conditions	Yield [%]	*E/Z*	e.r.
1	None	67(62)[b]	9/1	93:7
2	**L3** as ligand	48	8/1	96:4
3	**L4** as ligand	45	9/1	91:9
4	**L5** as ligand	40	8/1	87:13
5	$NiCl_2$•DME instead of $NiCl_2$•Py_4	68	8/1	86:14
6	$NiBr_2$•DME instead of $NiCl_2$•Py_4	73	9/1	91:9
7	$Ni(COD)_2$•DME instead of $NiCl_2$•Py_4	45	8/1	88:12
8	DME as solvent	60	8/1	86:14
9	CH_3CN as solvent	38	8/1	85:15
10	0 °C instead of 20 °C	35	8/1	93:7
11	Without nickel	ND	--	--
12	Without **L2**	ND	--	--
13	Without light	ND	--	--

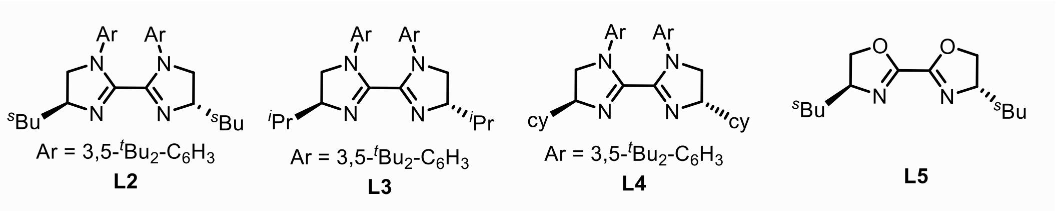

[a]Reactions were carried out with **VCP1** (0.2 mmol), methyl 4-bromobenzoate (0.1 mmol), $^{t}BuBF_3K$ (0.25 mmol), $NiCl_2$·Py_4 (10 mol%), **L2** (15 mol%), $Ir[dF(CF_3)ppy)]_2(bpy)PF_6$ (3 mol%), THF (0.05 M), 427 nm 45 W Kessil LED, 20 °C, N_2, 24 h. ^{1}H NMR yield with mesitylene as internal standard. Enantiomeric ratios (e.r.) were determined by HPLC with a chiral stationary phase. [b]Isolated yield.

Table S8. Unsuccessful examples of primary and secondary alkyl trifluoroborates[a]

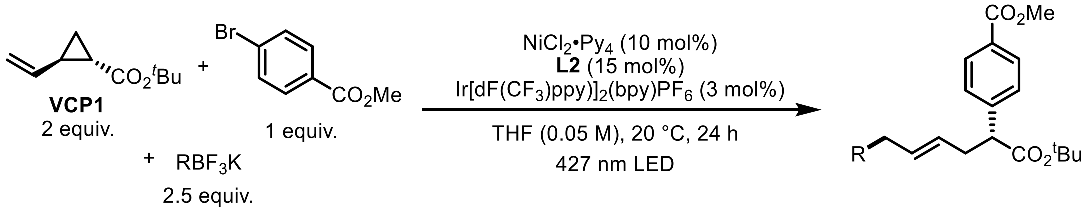

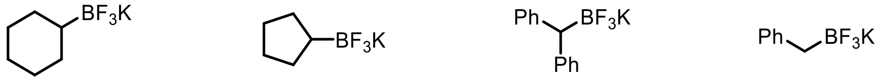

trace	ND	ND	ND

[a]Reactions were carried out with **VCP1** (0.2 mmol), methyl 4-bromobenzoate (0.1 mmol), RBF_3K (0.25 mmol), $NiCl_2 \cdot Py_4$ (10 mol %), **L2** (15 mol%), $Ir[dF(CF_3)ppy)]_2(bpy)PF_6$ (3 mol%), THF (0.05 M), 427 nm 45 W Kessil LED, 20 °C, N_2, 24 h.

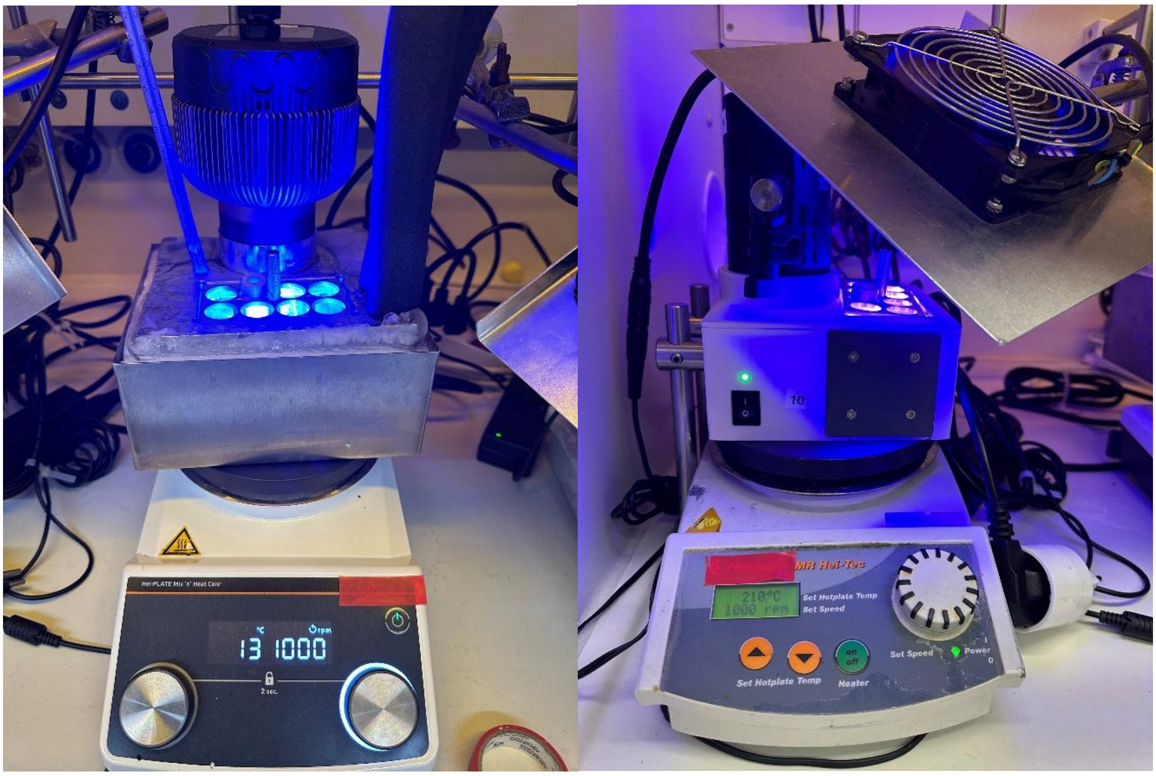

Figure S1. Reaction set-up

The set-up of the photoredox reaction is shown in Figure S1. In addition, a Julabo chiller was used to keep the temperature inside the photoreactor at 0 °C.

3.2 General Procedure for asymmetric 1,5-carbosulfonylation of vinyl cyclopropanes (standard conditions A).

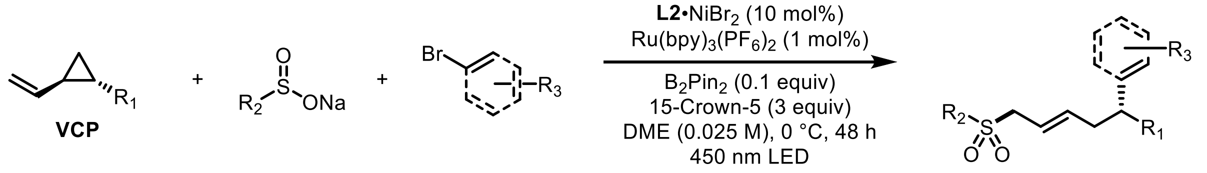

An oven-dried 7.5 mL screw-cap vial equipped with a magnetic stirring bar was charged with R_2-SO_2Na (0.1 mmol, 1 equiv.), aryl bromide or alkenyl bromide (if solid, 0.1 mmol, 1 equiv.), $Ru(bpy)_3(PF_6)_2$ (1 mol%), B_2Pin_2 (0.1 equiv.) and **L2**·$NiBr_2$ complex (0.01 mmol, 10 mol%) and then introduced into a nitrogen-filled glovebox. There, dry DME (4 mL) and 15-crown-5 (0.3 mmol, 3 equiv.) were sequentially added. The reaction vessel was then capped and removed from the glovebox. Vinyl cyclopropane (0.12 mmol, 1.2 equiv.) and aryl or alkenyl bromide (if liquid, 0.1 mmol, 1 equiv.) were subsequently added. The reaction was stirred (800 rpm) under irradiation with a 34 W 450 nm LED at 0 °C for 48 h. The reaction was quenched with saturated aq. NaCl (1 mL) and the resulting mixture was extracted with EtOAc (3 × 2 mL). The organic phase was concentrated under reduced pressure and the residue purified by column chromatography on silica gel. The enantiomeric ratio (e.r.) and *E*/*Z* ratio of the product were determined by HPLC with a chiral stationary phase.

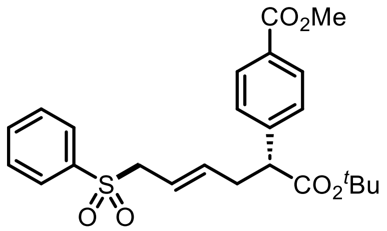

Methyl (*R,E*)-4-(1-(*tert*-butoxy)-1-oxo-6-(phenylsulfonyl)hex-4-en-2-yl)benzoate (1). Colorless oil (Hex/EA = 2/1), 70% yield, 95:5 er, $[\alpha]_D^{23}$ = -7.6 (c = 0.19, $CHCl_3$). ^{1}H NMR (500 MHz, $CDCl_3$) δ 7.99 – 7.95 (m, 2H), 7.78 (dd, *J* = 8.4, 1.3 Hz, 2H), 7.67 – 7.62 (m, 1H), 7.56 – 7.51 (m, 2H), 7.27 (d, *J* = 7.5 Hz, 2H), 5.50 – 5.39 (m, 2H), 3.91 (s, 3H), 3.71 – 3.67 (m, 2H), 3.46 (t, *J* = 7.6 Hz, 1H), 2.75 (ddd, *J* = 13.4, 7.9, 5.0 Hz, 1H), 2.48 – 2.40 (m, 1H), 1.36 (s, 9H). ^{13}C NMR (126 MHz, $CDCl_3$) δ 171.6, 167.0, 143.9, 138.6, 137.7, 133.8, 130.1,

129.3, 129.2, 128.6, 128.0, 118.8, 81.6, 60.0, 52.3, 52.1, 36.1, 28.0. IR (film): ν (cm^{-1}) 3396, 2967, 2914, 1747, 1296, 1170, 1156, 988, 789, 751. HR-MS (ESI) m/z calcd for $C_{24}H_{28}NaO_6S^+$ 467.14988, found 467.15045, [$M+Na^+$]. The enantiomeric ratio of **1** was determined by HPLC analysis on Chiralpak AD-H column. Conditions: hexane/isopropanol = 80/20, flow rate = 1.0 mL/min, uv-vis detection at λ = 254 nm, t_R = 12.7 min (major), 14.3 min (minor).

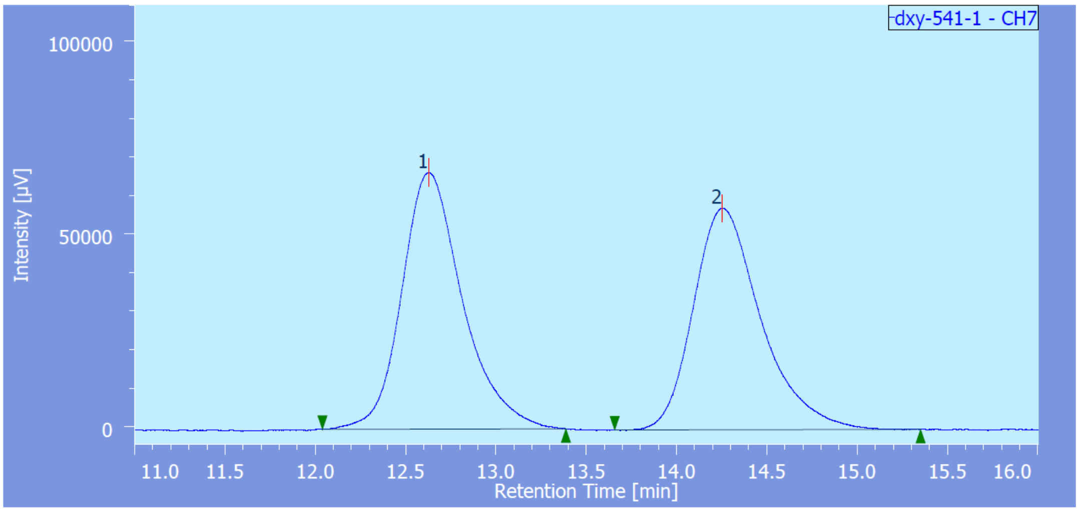

Decision

#	Peak Name	CH	tR [min]	Area [µV·sec]	Height [µV]	Area%	Height%	Quantity	NTP	Resolution	Symmetry Factor	Warning
1	Unknown	7	12.630	1559267	66507	51.011	53.647	N/A	7439	2.619	1.240	
2	Unknown	7	14.253	1497464	57464	48.989	46.353	N/A	7522	N/A	1.338	

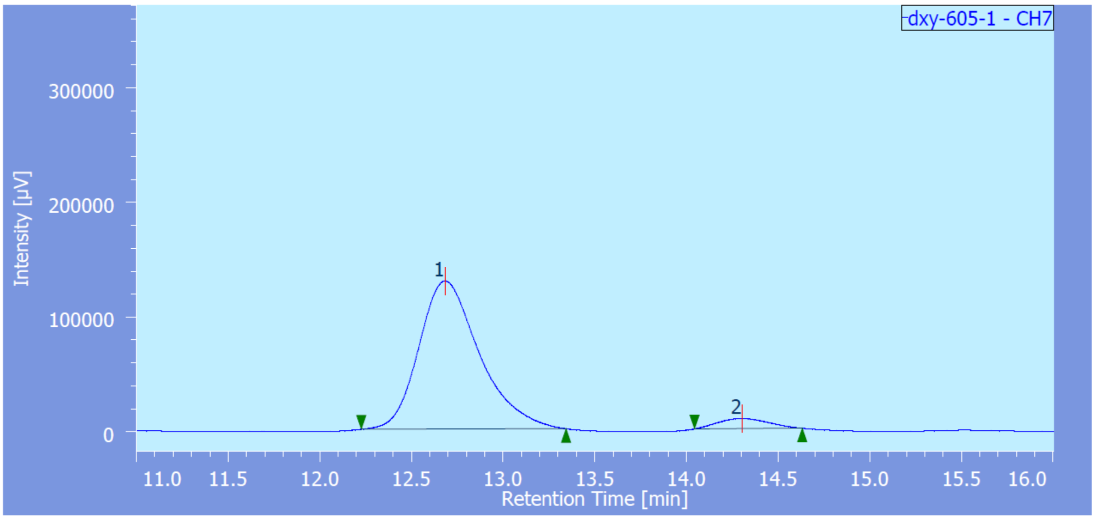

Decision

#	Peak Name	CH	tR [min]	Area [µV·sec]	Height [µV]	Area%	Height%	Quantity	NTP	Resolution	Symmetry Factor	Warning
1	Unknown	7	12.683	2970389	129195	94.646	93.618	N/A	7530	2.889	1.310	
2	Unknown	7	14.303	168040	8807	5.354	6.382	N/A	11238	N/A	1.110	

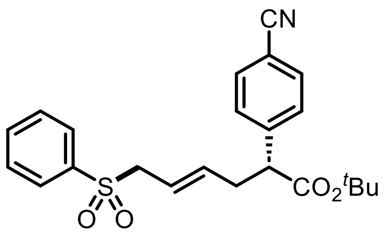

***tert*-Butyl (*R,E*)-2-(4-cyanophenyl)-6-(phenylsulfonyl)hex-4-enoate (3)**. Colorless oil (Hex/EA = 2/1), 72% yield, 88:12 er, $[\alpha]_D^{23}$ = -33.8 (c = 0.10, $CHCl_3$). ^{1}H NMR (400 MHz, $CDCl_3$) δ 7.80 (dd, *J* = 8.4, 1.3 Hz, 2H), 7.68 – 7.62 (m, 1H), 7.62 – 7.58 (m, 2H), 7.57 – 7.51 (m, 2H), 7.35 – 7.30 (m, 2H), 5.49 – 5.42 (m, 2H), 3.71 – 3.67 (m, 2H), 3.48 (t, *J* = 7.7 Hz, 1H), 2.80 – 2.70 (m, 1H), 2.47 – 2.36 (m, 1H), 1.36 (s, 9H). ^{13}C NMR (101 MHz, $CDCl_3$) δ 171.1, 144.0, 138.7, 137.3, 133.9, 132.5, 129.2, 128.8, 128.5, 119.1, 118.8, 111.4, 82.0, 59.9, 52.2, 36.1, 28.0. IR (film) ν (cm^{-1}) 2979, 2227, 1723, 1607, 1504, 1447, 1393, 1368, 1318, 1307, 1241, 1147, 1086, 970, 843, 766, 732, 689, 598, 550, 528. HR-MS (ESI) m/z calcd for $C_{23}H_{25}NNaO_4S^+$ 434.13965, found 434.14374, [M+Na$^+$]. The enantiomeric ratio of **3** was determined by HPLC analysis on Chiralpak IC column. Conditions: hexane/isopropanol = 50/50, flow rate = 1.0 mL/min, uv-vis detection at λ = 254 nm, t_R = 28.7 min (minor), 31.2 min (major).

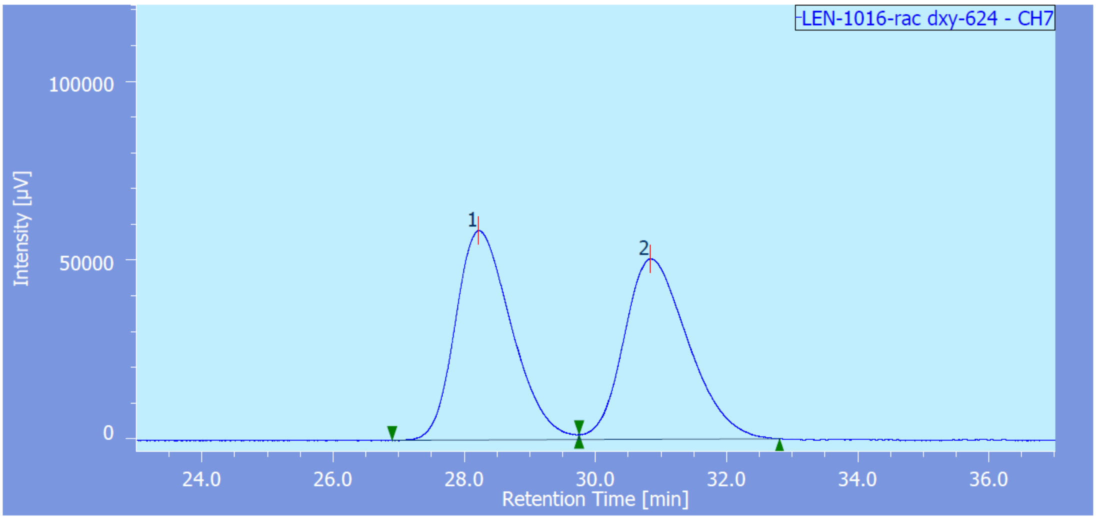

Decision

#	Peak Name	CH	tR [min]	Area [µV·sec]	Height [µV]	Area%	Height%	Quantity	NTP	Resolution	Symmetry Factor	Warning
1	Unknown	7	28.220	3477456	58484	50.351	53.741	N/A	5184	1.558	1.297	
2	Unknown	7	30.830	3429015	50341	49.649	46.259	N/A	4740	N/A	1.285	

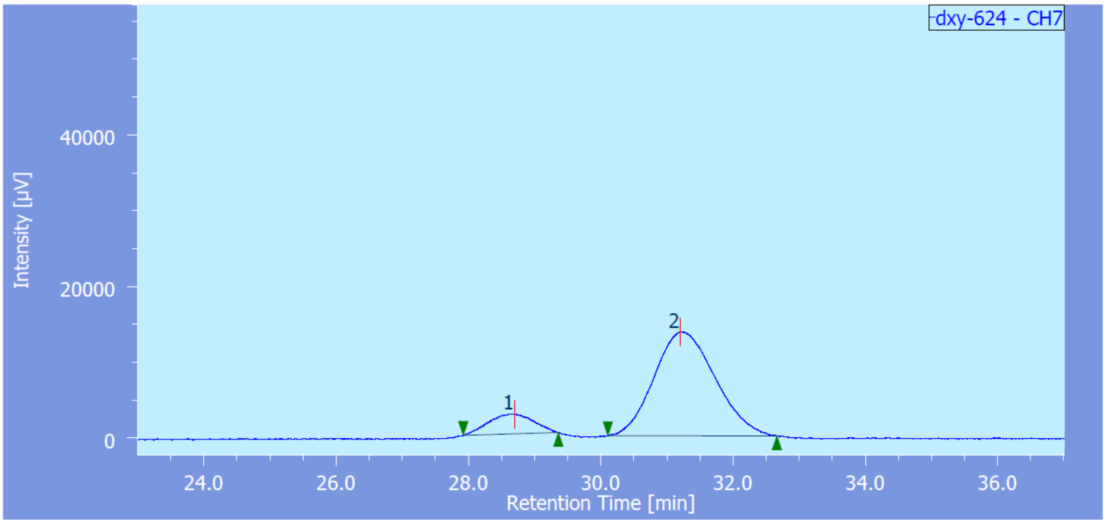

Decision

#	Peak Name	CH	tR [min]	Area [µV·sec]	Height [µV]	Area%	Height%	Quantity	NTP	Resolution	Symmetry Factor	Warning
1	Unknown	7	28.700	125127	2619	12.454	16.079	N/A	7272	1.634	0.929	
2	Unknown	7	31.200	879595	13668	87.546	83.921	N/A	5256	N/A	1.171	

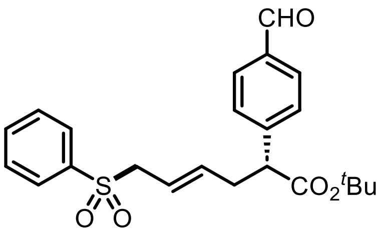

***tert*-Butyl (*R,E*)-2-(4-formylphenyl)-6-(phenylsulfonyl)hex-4-enoate (4)**. Colorless oil (Hex/EA = 1/1), 50% yield, 86:14 er, $[\alpha]_D^{23}$ = -22.8 (c = 0.17, $CHCl_3$). ^1H NMR (400 MHz, $CDCl_3$) δ 10.00 (s, 1H), 7.86 – 7.76 (m, 4H), 7.67 – 7.62 (m, 1H), 7.53 (dd, *J* = 8.3, 6.9 Hz, 2H), 7.38 (d, *J* = 8.4 Hz, 2H), 5.46 (td, *J* = 4.5, 4.1, 1.8 Hz, 2H), 3.69 (d, *J* = 6.3 Hz, 2H), 3.51 (t, *J* = 7.6 Hz, 1H), 2.84 – 2.72 (m, 1H), 2.53 – 2.39 (m, 1H), 1.37 (s, 9H). ^{13}C NMR (101 MHz, $CDCl_3$) δ 191.9, 171.4, 145.6, 138.6, 137.6, 135.7, 133.9, 130.2, 129.2, 128.7, 128.5, 118.9, 81.8, 60.0, 52.3, 36.1, 28.0. IR (film) ν (cm^{-1}) 2975, 2927, 1722, 1700, 1605, 1585, 1447, 1392, 1368, 1306, 1244, 1210, 1147, 1086, 970, 842, 765, 732, 689, 600, 555, 529. HR-MS (ESI) m/z calcd for $C_{23}H_{27}O_5S^+$ 415.15737, found 415.14886, [M+H$^+$]. The enantiomeric ratio of **4** was determined by HPLC analysis on Chiralpak IC column. Conditions: hexane/isopropanol = 70/30, flow rate = 1.0 mL/min, uv-vis detection at λ = 254 nm, t_R = 65.0 min (minor), 70.5 min (major).

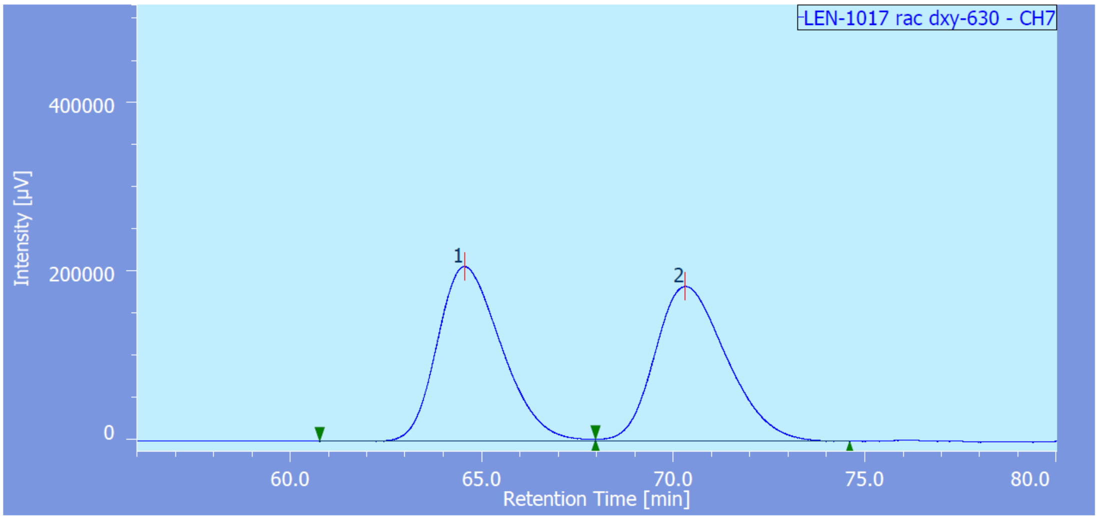

Decision

#	Peak Name	CH	tR [min]	Area [µV·sec]	Height [µV]	Area%	Height%	Quantity	NTP	Resolution	Symmetry Factor	Warning
1	Unknown	7	64.547	24231711	207372	50.253	53.002	N/A	7022	1.772	1.278	
2	Unknown	7	70.310	23988035	183880	49.747	46.998	N/A	6681	N/A	1.263	

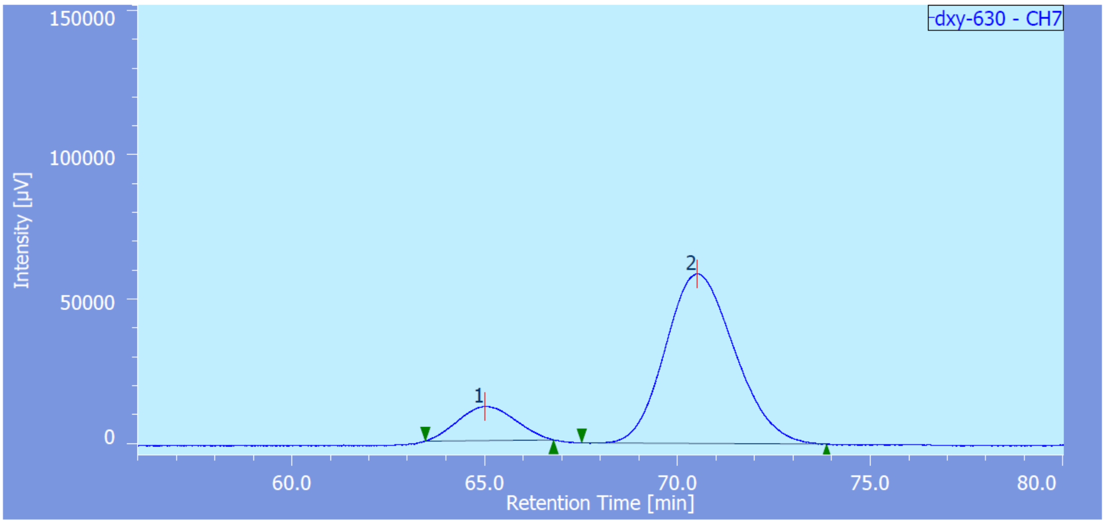

Decision

#	Peak Name	CH	tR [min]	Area [µV·sec]	Height [µV]	Area%	Height%	Quantity	NTP	Resolution	Symmetry Factor	Warning
1	Unknown	7	65.013	1221781	11739	14.249	16.670	N/A	8005	1.769	1.073	
2	Unknown	7	70.510	7352587	58682	85.751	83.330	N/A	7196	N/A	1.152	

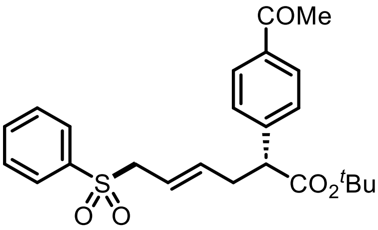

***tert*-Butyl (*R*,*E*)-2-(4-acetylphenyl)-6-(phenylsulfonyl)hex-4-enoate (5).** Colorless oil (Hex/EA = 2/1), 63%, 94:6 er, $[\alpha]_D^{23}$ = -45.5 (c = 0.077, $CHCl_3$). ^1H NMR (400 MHz, $CDCl_3$) δ 7.89 (d, *J* = 8.5 Hz, 2H), 7.78 (dd, *J* = 8.4, 1.3 Hz, 2H), 7.68 – 7.61 (m, 1H), 7.57 – 7.51 (m,

2H), 7.33 – 7.27 (m, 2H), 5.49 – 5.42 (m, 2H), 3.73 – 3.65 (m, 2H), 3.48 (t, J = 7.6 Hz, 1H), 2.80 – 2.71 (m, 1H), 2.59 (s, 3H), 2.51 – 2.35 (m, 1H), 1.36 (s, 9H). ^{13}C NMR (101 MHz, $CDCl_3$) δ 197.8, 171.6, 144.1, 138.6, 137.7, 136.3, 133.8, 129.2, 128.8, 128.5, 128.2, 118.8, 81.6, 60.0, 52.1, 36.1, 28.0, 26.8. IR (film) ν (cm^{-1}) 2976, 2934, 1722, 1682, 1605, 1478, 1446, 1415, 1393, 1366, 1306, 1267, 1146, 1086, 1017, 961, 843, 766, 733, 689, 597, 552, 528. HR-MS (ESI) m/z calcd for $C_{24}H_{28}NaO_5S^+$ 451.15497, found 451.15192, [M+Na$^+$]. The enantiomeric ratio of **5** was determined by HPLC analysis on Chiralpak AD-H column. Conditions: hexane/isopropanol = 90/10, flow rate = 1.0 mL/min, uv-vis detection at λ = 254 nm, t_R = 35.5 min (major), 38.9 min (minor).

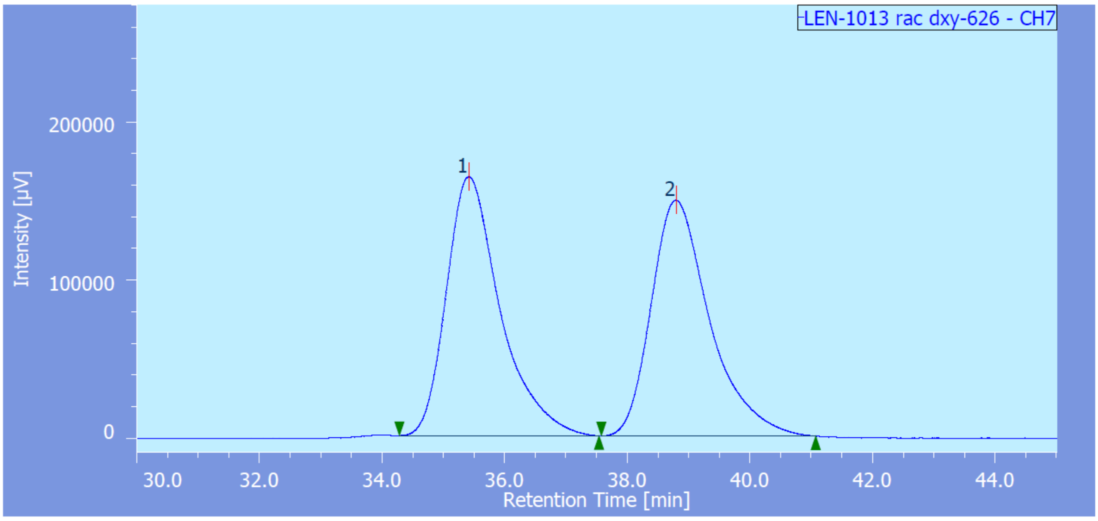

Decision

#	Peak Name	CH	tR [min]	Area [µV·sec]	Height [µV]	Area%	Height%	Quantity	NTP	Resolution	Symmetry Factor	Warning
1	Unknown	7	35.413	9988449	163739	50.270	52.362	N/A	8716	2.131	1.429	
2	Unknown	7	38.793	9881138	148965	49.730	47.638	N/A	8701	N/A	1.404	

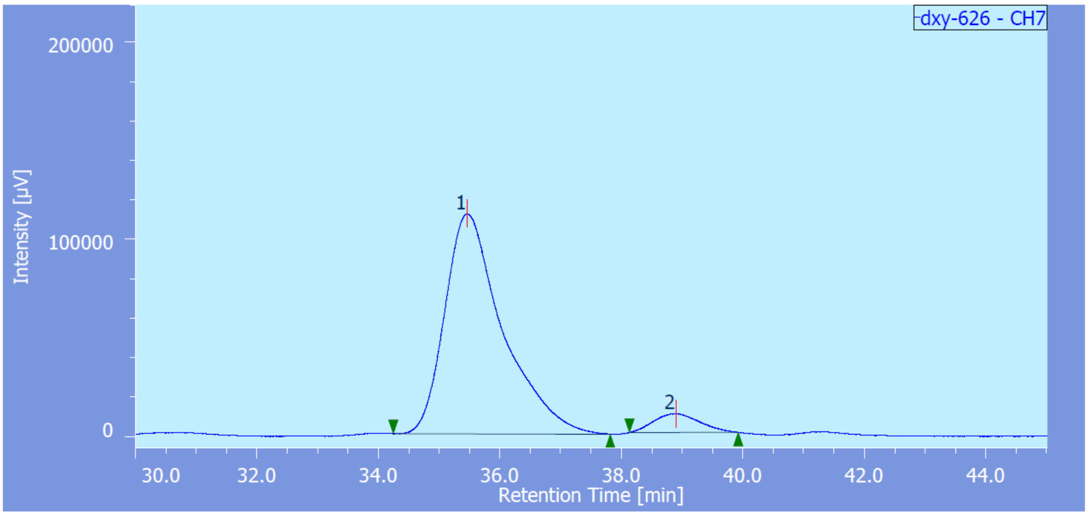

Decision

#	Peak Name	CH	tR [min]	Area [μV·sec]	Height [μV]	Area%	Height%	Quantity	NTP	Resolution	Symmetry Factor	Warning
1	Unknown	7	35.460	7385113	111601	93.612	92.159	N/A	7853	2.251	1.540	
2	Unknown	7	38.893	503957	9495	6.388	7.841	N/A	11388	N/A	1.158	

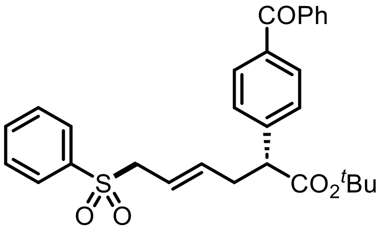

***tert*-Butyl (*R,E*)-2-(4-benzoylphenyl)-6-(phenylsulfonyl)hex-4-enoate (6)**. Colorless oil (Hex/EA = 2/1), 71%, 94:6 er, $[\alpha]_D^{23}$ = -32.7 (c = 0.090, $CHCl_3$). ^1H NMR (400 MHz, $CDCl_3$) δ 7.82 – 7.74 (m, 6H), 7.67 – 7.62 (m, 1H), 7.61 – 7.57 (m, 1H), 7.57 – 7.52 (m, 2H), 7.51 – 7.45 (m, 2H), 7.32 (d, *J* = 8.4 Hz, 2H), 5.48 (td, *J* = 4.1, 1.9 Hz, 2H), 3.70 (dd, *J* = 4.3, 2.0 Hz, 2H), 3.51 (t, *J* = 7.6 Hz, 1H), 2.84 – 2.72 (m, 1H), 2.55 – 2.42 (m, 1H), 1.38 (s, 9H). ^{13}C NMR (101 MHz, $CDCl_3$) δ 196.4, 171.6, 143.4, 138.6, 137.8, 137.6, 136.7, 133.9, 132.6, 130.6, 130.1, 129.2, 128.5, 128.4, 127.9, 118.8, 81.6, 60.0, 52.1, 36.2, 28.1. IR (film) ν (cm^{-1}) 3061, 2978, 2921, 1724, 1656, 1605, 1447, 1368, 1309, 1278, 1148, 1087, 971, 938, 925, 848, 734, 703, 530. HR-MS (ESI) m/z calcd for $C_{29}H_{31}O_5S^+$ 491.18867, found 491.18256, [$M+H^+$]. The enantiomeric ratio of **6** was determined by HPLC analysis on Chiralpak AD-H column. Conditions: hexane/isopropanol = 70/30, flow rate = 1.0 mL/min, uv-vis detection at λ = 300 nm, t_R = 12.7 min (minor), 14.7 min (major).

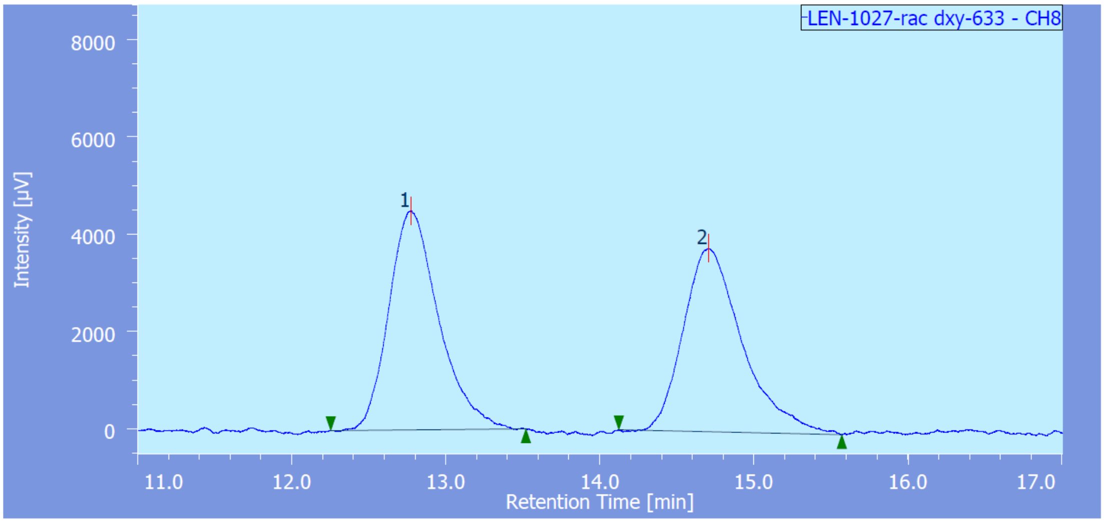

Decision

#	Peak Name	CH	tR [min]	Area [μV·sec]	Height [μV]	Area%	Height%	Quantity	NTP	Resolution	Symmetry Factor	Warning
1	Unknown	8	12.773	101887	4503	50.325	54.435	N/A	7946	3.123	1.323	
2	Unknown	8	14.703	100572	3769	49.675	45.565	N/A	7796	N/A	1.397	

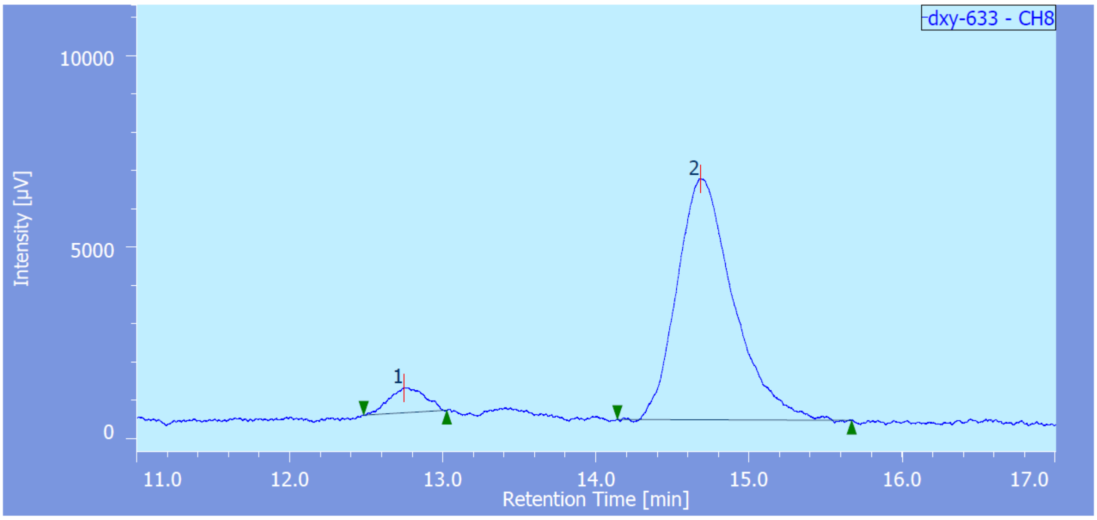

Decision

#	Peak Name	CH	tR [min]	Area [μV·sec]	Height [μV]	Area%	Height%	Quantity	NTP	Resolution	Symmetry Factor	Warning
1	Unknown	8	12.747	10694	662	6.220	9.524	N/A	13412	3.581	1.002	
2	Unknown	8	14.680	161245	6293	93.780	90.476	N/A	8354	N/A	1.369	

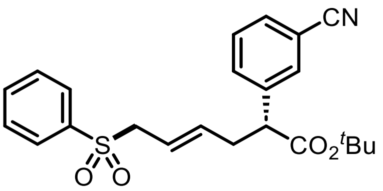

***tert*-Butyl (*R,E*)-2-(3-cyanophenyl)-6-(phenylsulfonyl)hex-4-enoate (7)**. Colorless oil (Hex/EA = 2/1), 63%, 96:4 er, $[\alpha]_D{}^{23}$ = -32.1 (c = 0.093, $CHCl_3$). ^1H NMR (400 MHz, $CDCl_3$) δ 7.80 (dd, *J* = 8.4, 1.3 Hz, 2H), 7.70 – 7.63 (m, 1H), 7.60 – 7.52 (m, 3H), 7.50 – 7.39 (m, 3H), 5.54 – 5.35 (m, 2H), 3.69 (d, *J* = 6.4 Hz, 2H), 3.44 (t, *J* = 7.7 Hz, 1H), 2.74 (ddd, *J* = 14.0, 7.9, 6.1 Hz, 1H), 2.48 – 2.37 (m, 1H), 1.37 (s, 9H). ^{13}C NMR (101 MHz, $CDCl_3$) δ 171.2, 140.1, 138.6, 137.2, 133.9, 132.5, 131.6, 131.2, 129.6, 129.2, 128.5, 119.2, 118.7, 112.9, 82.0, 59.9, 51.7, 36.2, 28.0. IR (film) ν (cm^{-1}) 2978, 2230, 1722, 1481, 1447, 1368, 1307, 1241, 1147, 1086, 972, 844, 734, 689, 597, 554, 527. HR-MS (ESI) m/z calcd for $C_{23}H_{26}NO_4S^+$ 412.15771, found 412.14719, $[M+H^+]$. The enantiomeric ratio of **7** was determined by HPLC analysis on Chiralpak OJ-H column. Conditions: hexane/isopropanol = 50/50, flow rate = 1.0 mL/min, uv-vis detection at λ = 220 nm, t_R = 10.6 min (major), 12.4 min (minor).

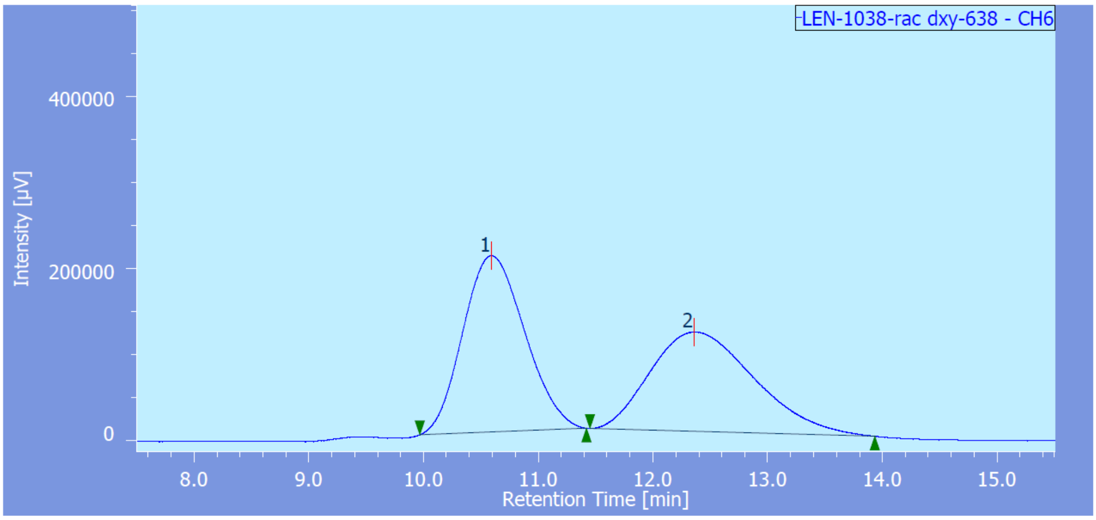

Decision

#	Peak Name	CH	tR [min]	Area [µV·sec]	Height [µV]	Area%	Height%	Quantity	NTP	Resolution	Symmetry Factor	Warning
1	Unknown	6	10.590	7684033	204951	51.332	63.981	N/A	1747	1.302	1.148	
2	Unknown	6	12.353	7285146	115379	48.668	36.019	N/A	843	N/A	1.326	

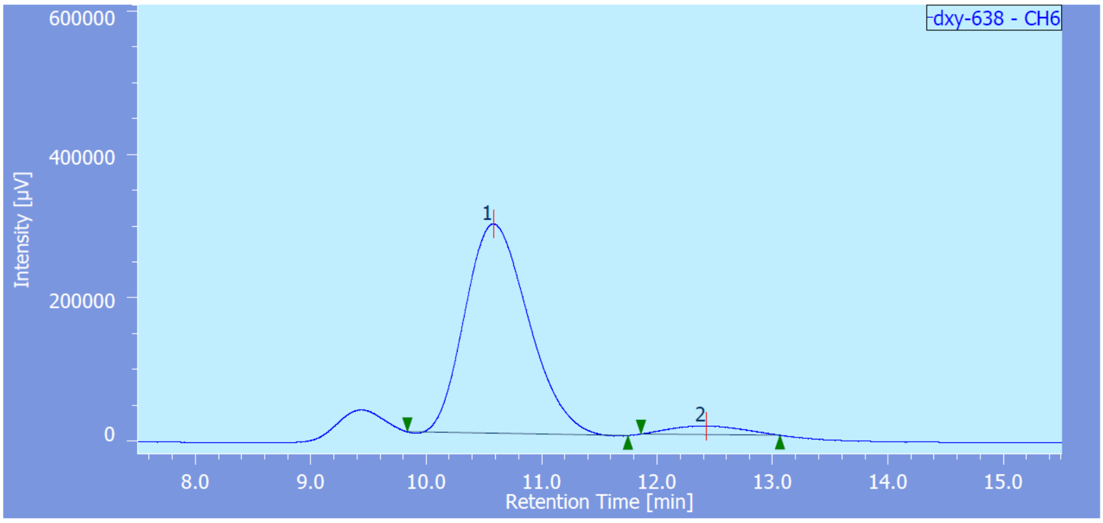

Decision

#	Peak Name	CH	tR [min]	Area [µV·sec]	Height [µV]	Area%	Height%	Quantity	NTP	Resolution	Symmetry Factor	Warning
1	Unknown	6	10.580	11162829	292395	95.550	96.093	N/A	1713	1.611	1.225	
2	Unknown	6	12.427	519843	11888	4.450	3.907	N/A	1516	N/A	1.060	

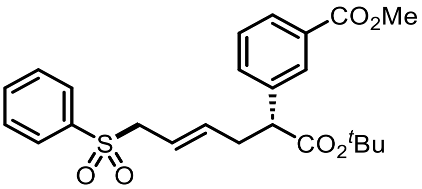

Methyl (*R*,*E*)-3-(1-(*tert*-butoxy)-1-oxo-6-(phenylsulfonyl)hex-4-en-2-yl)benzoate (8). Colorless oil (Hex/EA = 2/1), 52%, 90:10 er, $[\alpha]_D^{23}$ = -34.8 (c = 0.053, $CHCl_3$). ^1H NMR (400 MHz, $CDCl_3$) δ 7.93 (d, *J* = 7.2 Hz, 1H), 7.89 (t, *J* = 1.8 Hz, 1H), 7.82 – 7.76 (m, 2H), 7.68 – 7.60 (m, 1H), 7.57 – 7.48 (m, 2H), 7.44 – 7.34 (m, 2H), 5.52 – 5.39 (m, 2H), 3.92 (s, 3H), 3.69

(d, J = 5.6 Hz, 2H), 3.46 (t, J = 7.7 Hz, 1H), 2.81 – 2.70 (m, 1H), 2.50 – 2.38 (m, 1H), 1.36 (s, 9H). ^{13}C NMR (101 MHz, $CDCl_3$) δ 171.8, 167.0, 139.1, 138.5, 137.8, 133.8, 132.5, 130.7, 129.2, 128.8, 128.7, 128.6, 118.8, 81.5, 60.0, 52.3, 51.9, 36.3, 28.0 (one peak overlapping with others). IR (film) ν (cm^{-1}) 2978, 1719, 1586, 1446, 1368, 1284, 1196, 1147, 1109, 1086, 973, 846, 750, 733, 689, 594, 529. HR-MS (ESI) m/z calcd for $C_{24}H_{28}NaO_6S^+$ 467.14988, found 467.14224, [M+Na$^+$]. The enantiomeric ratio of **8** was determined by HPLC analysis on Chiralpak IC column. Conditions: hexane/isopropanol = 60/40, flow rate = 1.0 mL/min, uv-vis detection at λ = 220 nm, t_R = 29.7 min (minor), 32.4 min (major).

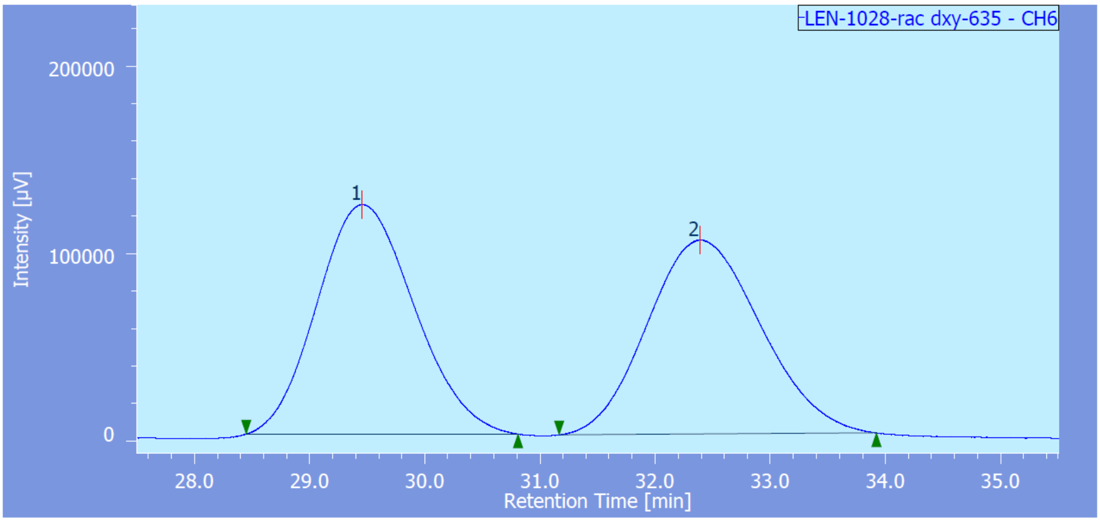

Decision

#	Peak Name	CH	tR [min]	Area [μV·sec]	Height [μV]	Area%	Height%	Quantity	NTP	Resolution	Symmetry Factor	Warning
1	Unknown	6	29.457	7192504	122437	50.383	54.185	N/A	5596	1.720	1.132	
2	Unknown	6	32.387	7083029	103523	49.617	45.815	N/A	4957	N/A	1.126	

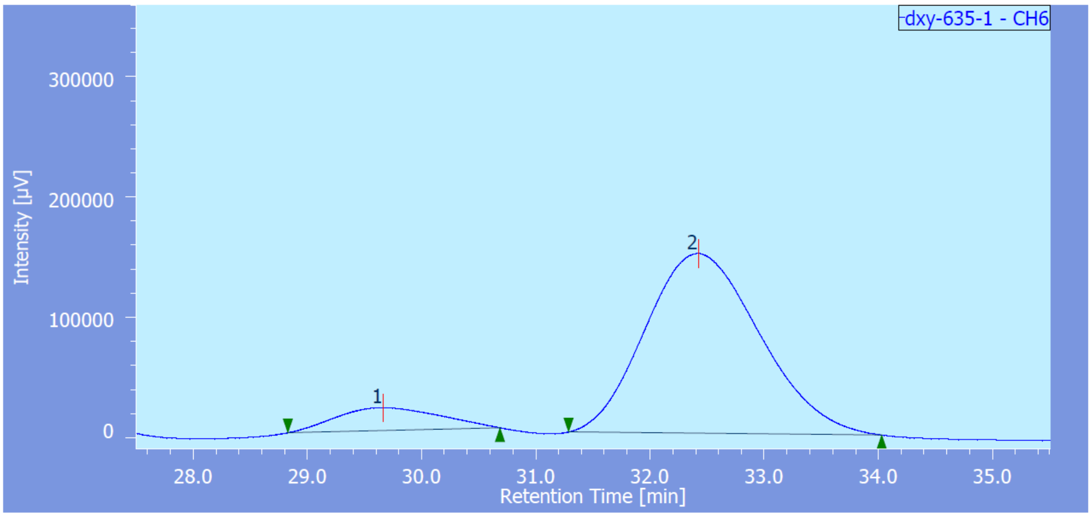

Decision

#	Peak Name	CH	tR [min]	Area [μV·sec]	Height [μV]	Area%	Height%	Quantity	NTP	Resolution	Symmetry Factor	Warning
1	Unknown	6	29.667	1203103	19010	10.362	11.303	N/A	4262	1.495	1.119	
2	Unknown	6	32.423	10407745	149172	89.638	88.697	N/A	4754	N/A	1.156	

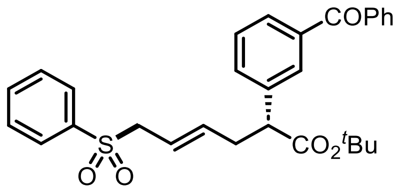

Ethyl (*S*)-4-(1-benzamido-2-(phenylsulfonyl)ethyl)benzoate (9). Colorless oil (Hex/EA = 2/1), 50%, 90:10 er, $[\alpha]_D^{23}$ = -17.9 (c = 0.13, $CHCl_3$). ^1H NMR (400 MHz, $CDCl_3$) δ 7.82 – 7.75 (m, 4H), 7.69 (dt, *J* = 7.0, 1.8 Hz, 1H), 7.66 – 7.57 (m, 3H), 7.56 – 7.41 (m, 6H), 5.53 – 5.41 (m, 2H), 3.73 – 3.65 (m, 2H), 3.48 (t, *J* = 7.6 Hz, 1H), 2.82 – 2.69 (m, 1H), 2.50 – 2.40 (m, 1H), 1.38 (s, 9H). ^{13}C NMR (101 MHz, $CDCl_3$) δ 196.6, 171.8, 139.1, 138.5, 138.1, 137.8, 137.6, 133.9, 132.7, 131.9, 130.2, 129.6, 129.2, 129.2, 128.8, 128.6, 128.5, 118.8, 81.6, 60.0, 52.0, 36.3, 28.1. IR (film) ν (cm^{-1}) 2978, 2928, 1724, 1659, 1598, 1446, 1369, 1318, 1308, 1280, 1241, 1147, 1086, 1045, 971, 913, 845, 730, 689, 644, 596, 559, 528. HR-MS (ESI) m/z calcd for $C_{29}H_{31}O_5S^+$ 491.18867, found 491.18439, $[M+H^+]$. The enantiomeric ratio of **9** was determined by HPLC analysis on Chiralpak IC column. Conditions: hexane/isopropanol = 90/10, flow rate = 1.0 mL/min, uv-vis detection at λ = 254 nm, t_R = 32.2 min (minor), 35.1 min (major).

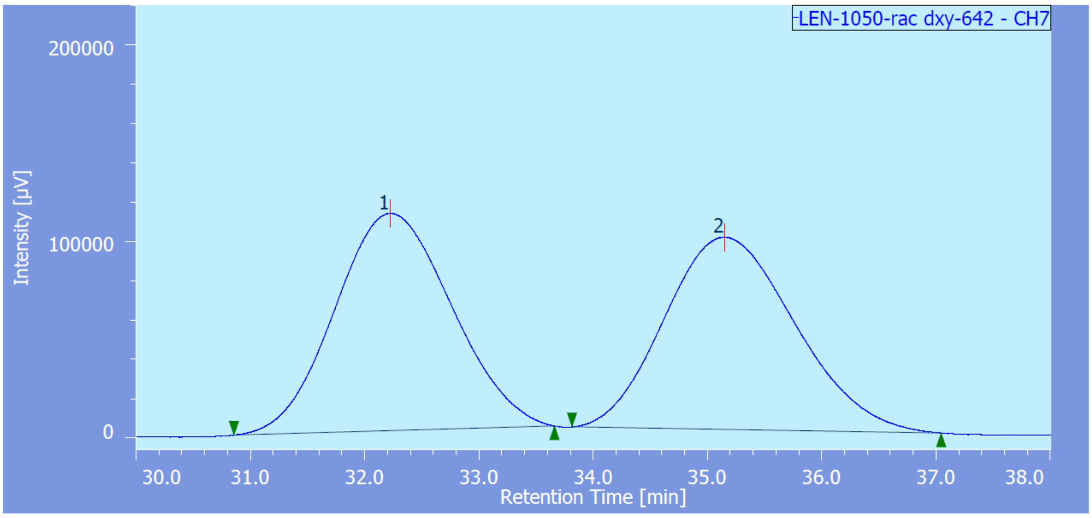

Decision

#	Peak Name	CH	tR [min]	Area [µV·sec]	Height [µV]	Area%	Height%	Quantity	NTP	Resolution	Symmetry Factor	Warning
1	Unknown	7	32.223	7845550	110517	50.277	53.038	N/A	4518	1.448	1.075	
2	Unknown	7	35.150	7759215	97857	49.723	46.962	N/A	4337	N/A	1.155	

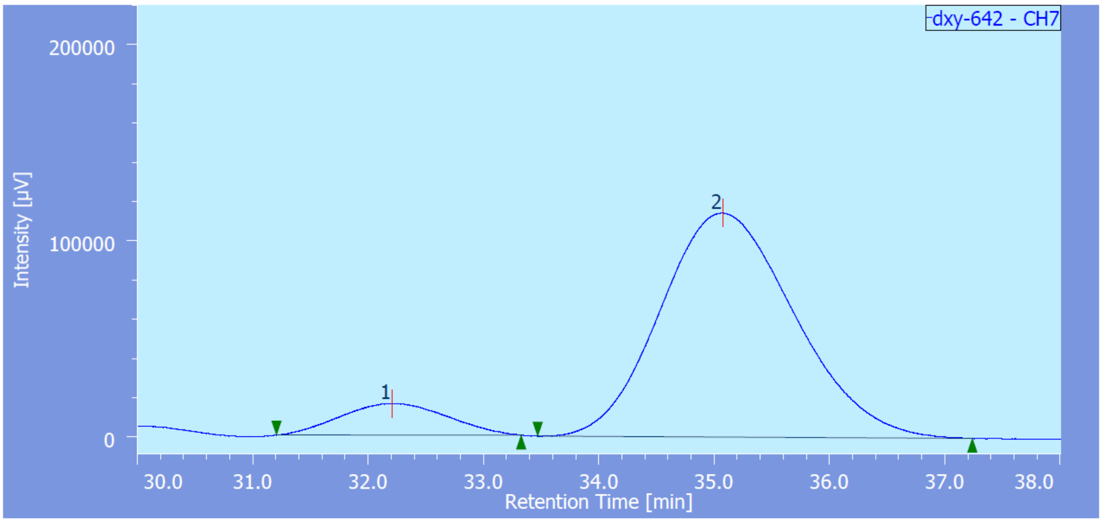

Decision

#	Peak Name	CH	tR [min]	Area [µV·sec]	Height [µV]	Area%	Height%	Quantity	NTP	Resolution	Symmetry Factor	Warning
1	Unknown	7	32.207	1037877	16140	10.101	12.384	N/A	5151	1.456	1.060	
2	Unknown	7	35.073	9236937	114185	89.899	87.616	N/A	4242	N/A	1.144	

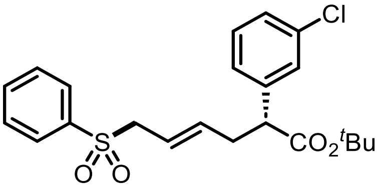

***tert*-Butyl (*R,E*)-2-(3-chlorophenyl)-6-(phenylsulfonyl)hex-4-enoate (10)**. Colorless oil (Hex/EA = 2/1), 45%, 91:9 er, $[\alpha]_D^{23}$ = -25.7 (c = 0.044, $CHCl_3$). ^1H NMR (400 MHz, $CDCl_3$) δ 7.79 (dd, *J* = 8.4, 1.3 Hz, 2H), 7.68 – 7.63 (m, 1H), 7.57 – 7.51 (m, 2H), 7.25 – 7.22 (m, 2H), 7.19 (q, *J* = 1.5 Hz, 1H), 7.09 (td, *J* = 4.6, 1.8 Hz, 1H), 5.52 – 5.36 (m, 2H), 3.70 (d, *J* = 6.5 Hz, 2H), 3.36 (t, *J* = 7.7 Hz, 1H), 2.76 – 2.65 (m, 1H), 2.46 – 2.34 (m, 1H), 1.37 (s, 9H). ^{13}C NMR (101 MHz, $CDCl_3$) δ 171.7, 140.6, 138.5, 137.7, 134.5, 133.9, 130.0, 129.2, 128.6, 128.1, 127.6, 126.2, 118.8, 81.6, 60.0, 51.8, 36.2, 28.0. IR (film) ν (cm^{-1}) 2977, 2923, 1722, 1595, 1573, 1476, 1446, 1432, 1393, 1367, 1319, 1307, 1240, 1147, 1086, 999, 970, 879, 844, 767, 731, 689, 594, 529, 501. HR-MS (ESI) m/z calcd for $C_{22}H_{25}ClNaO_4S^+$ 443.10543, found 443.09723, [M+Na $^+$]. The enantiomeric ratio of **10** was determined by HPLC analysis on Chiralpak NR column. Conditions: hexane/isopropanol = 90/10, flow rate = 1.0 mL/min, uv-vis detection at λ = 210 nm, t_R = 86.5 min (major), 96.5 min (minor).

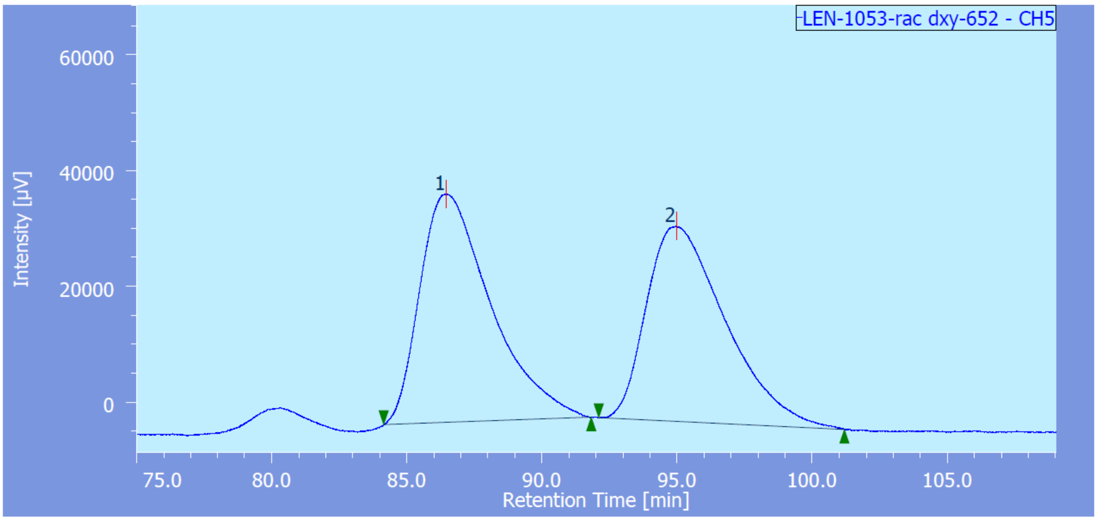

Decision

#	Peak Name	CH	tR [min]	Area [μV·sec]	Height [μV]	Area%	Height%	Quantity	NTP	Resolution	Symmetry Factor	Warning
1	Unknown	5	86.450	7212929	39437	51.241	53.924	N/A	5388	1.699	1.647	
2	Unknown	5	94.973	6863564	33698	48.759	46.076	N/A	5039	N/A	1.559	

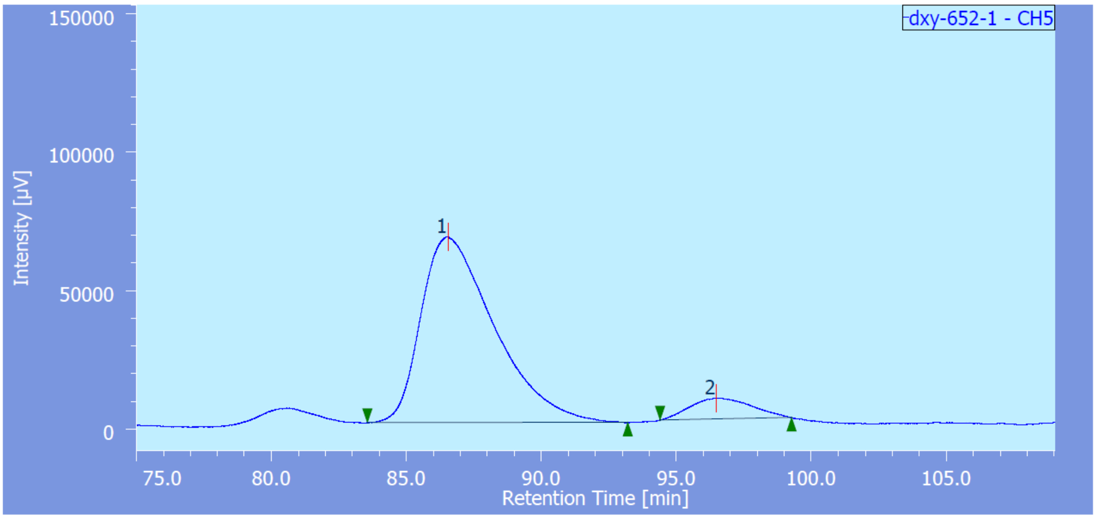

Decision

#	Peak Name	CH	tR [min]	Area [μV·sec]	Height [μV]	Area%	Height%	Quantity	NTP	Resolution	Symmetry Factor	Warning
1	Unknown	5	86.530	12665246	66854	91.418	90.024	N/A	4996	2.118	1.625	
2	Unknown	5	96.473	1188949	7408	8.582	9.976	N/A	7303	N/A	1.188	

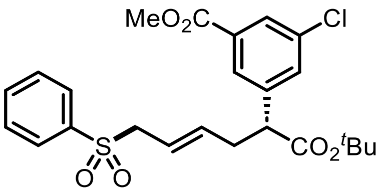

Methyl (*R,E*)-3-(1-(*tert*-butoxy)-1-oxo-6-(phenylsulfonyl)hex-4-en-2-yl)-5-chlorobenzoate (11). Colorless oil (Hex/EA = 2/1), 63%, 89:11 er, $[\alpha]_D^{23}$ = -21.7 (c = 0.080, $CHCl_3$). ^{1}H NMR (400 MHz, $CDCl_3$) δ 7.91 (dd, *J* = 2.1, 1.4 Hz, 1H), 7.81 (d, *J* = 1.3 Hz, 1H), 7.80 – 7.77 (m, 2H), 7.68 – 7.62 (m, 1H), 7.57 – 7.51 (m, 2H), 7.39 (t, *J* = 1.9 Hz, 1H), 5.54 – 5.36 (m, 2H),

3.92 (s, 3H), 3.70 (d, J = 6.3 Hz, 2H), 3.42 (t, J = 7.6 Hz, 1H), 2.79 – 2.69 (m, 1H), 2.47 – 2.36 (m, 1H), 1.37 (s, 9H). ^{13}C NMR (101 MHz, $CDCl_3$) δ 171.2, 165.8, 141.0, 138.5, 137.3, 134.8, 133.9, 132.4, 132.2, 129.2, 128.8, 128.5, 127.4, 119.2, 81.9, 60.0, 52.6, 51.7, 36.2, 28.0. IR (film) ν (cm^{-1}) 2978, 1722, 1579, 1447, 1433, 1393, 1368, 1320, 1307, 1286, 1200, 1147, 1086, 998, 973, 885, 843, 797, 767, 733, 689, 595, 560, 529. HR-MS (ESI) m/z calcd for $C_{24}H_{27}ClNaO_6S^+$ 501.11091, found 501.10663, [M+Na$^+$]. The enantiomeric ratio of **11** was determined by HPLC analysis on Chiralpak OJ-H column. Conditions: hexane/isopropanol = 95/5, flow rate = 1.0 mL/min, uv-vis detection at λ = 220 nm, t_R = 73.1 min (major), 79.5 min (minor).

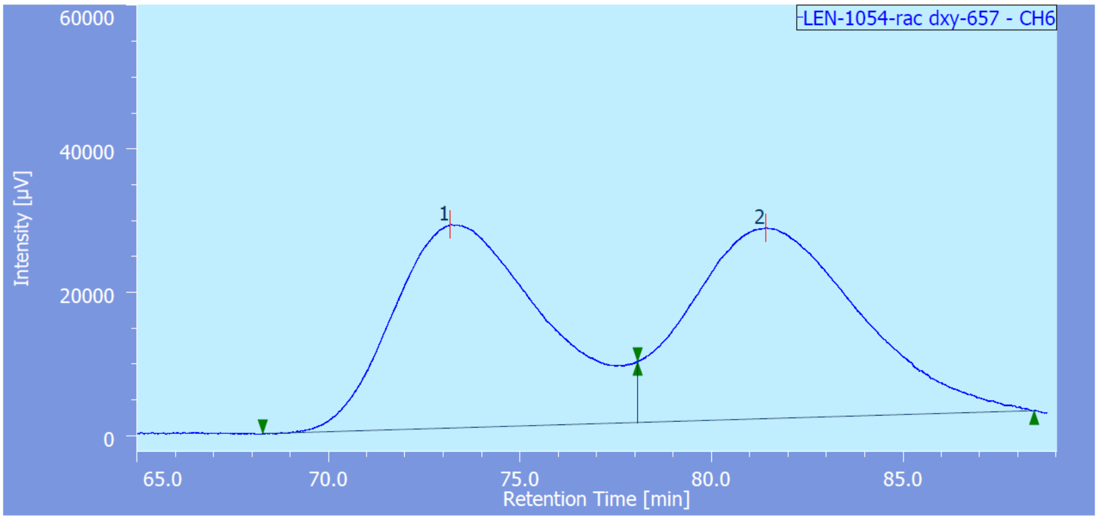

Decision

#	Peak Name	CH	tR [min]	Area [µV·sec]	Height [µV]	Area%	Height%	Quantity	NTP	Resolution	Symmetry Factor	Warning
1	Unknown	6	73.180	7785117	28333	48.680	51.644	N/A	1635	1.054	N/A	
2	Unknown	6	81.413	8207361	26529	51.320	48.356	N/A	1492	N/A	N/A	

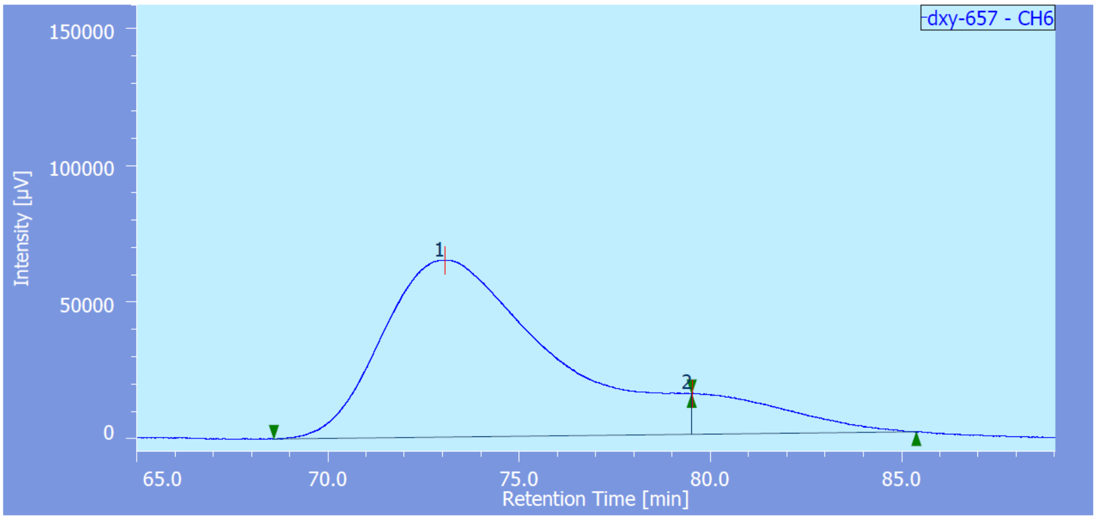

Decision

#	Peak Name	CH	tR [min]	Area [µV·sec]	Height [µV]	Area%	Height%	Quantity	NTP	Resolution	Symmetry Factor	Warning
1	Unknown	6	73.070	19592375	64623	88.550	81.230	N/A	1517	N/A	N/A	
2	Unknown	6	79.537	2533311	14932	11.450	18.770	N/A	N/A	N/A	N/A	

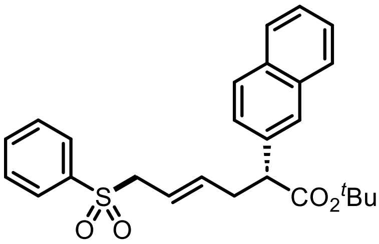

***tert*-Butyl (*R*,*E*)-2-(naphthalen-2-yl)-6-(phenylsulfonyl)hex-4-enoate (12)**. Colorless oil (Hex/EA = 2/1), 48%, 92:8 er, $[\alpha]_D{}^{23}$ = -10.5 (c = 0.23, $CHCl_3$). ^1H NMR (400 MHz, $CDCl_3$) δ 7.84 – 7.76 (m, 3H), 7.74 (dd, J = 8.5, 1.3 Hz, 2H), 7.63 (s, 1H), 7.59 (t, J = 7.5 Hz, 1H), 7.51 – 7.42 (m, 4H), 7.35 (dd, J = 8.4, 1.8 Hz, 1H), 5.51 – 5.41 (m, 2H), 3.73 – 3.65 (m, 2H), 3.57 (t, J = 7.6 Hz, 1H), 2.87 – 2.75 (m, 1H), 2.61 – 2.45 (m, 1H), 1.36 (s, 9H). ^{13}C NMR (101 MHz, $CDCl_3$) δ 172.2, 138.4, 138.1, 136.0, 133.6, 133.4, 132.6, 129.0, 128.4, 128.3, 127.9, 127.6, 126.7, 126.2, 125.9, 125.8, 118.4, 81.1, 60.0, 52.1, 36.2, 27.9. IR (film) ν (cm^{-1}) 2976, 2917, 1721, 1478, 1446, 1392, 1367, 1307, 1241, 1147, 1086, 968, 847, 821, 733, 689, 600, 558, 531, 503, 476. HR-MS (ESI) m/z calcd for $C_{26}H_{28}NaO_4S^+$ 459.16005, found 459.15671, $[M+Na^+]$. The enantiomeric ratio of **12** was determined by HPLC analysis on Chiralpak AD-H column. Conditions: hexane/isopropanol = 90/10, flow rate = 1.0 mL/min, uv-vis detection at λ = 220 nm, t_R = 19.3 min (major), 23.6 min (minor).

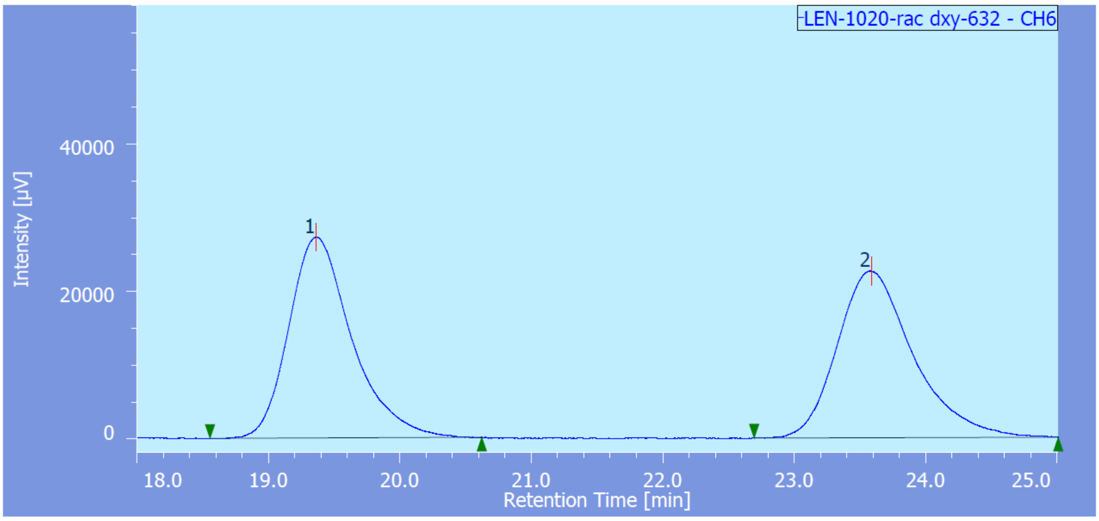

Decision

#	Peak Name	CH	tR [min]	Area [µV·sec]	Height [µV]	Area%	Height%	Quantity	NTP	Resolution	Symmetry Factor	Warning
1	Unknown	6	19.363	907941	27235	49.944	54.676	N/A	8562	4.575	1.335	
2	Unknown	6	23.583	909960	22576	50.056	45.324	N/A	8680	N/A	1.312	

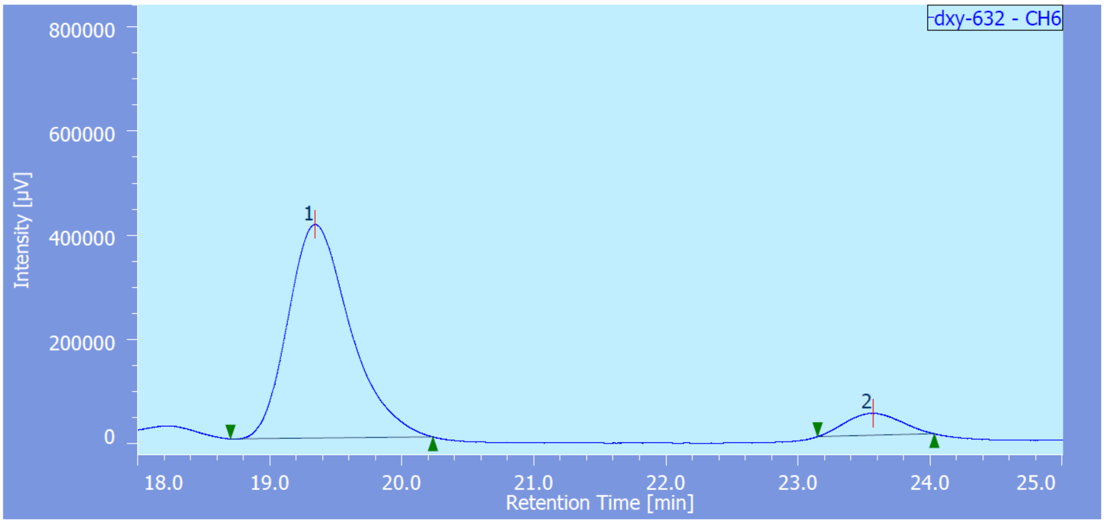

Decision

#	Peak Name	CH	tR [min]	Area [µV·sec]	Height [µV]	Area%	Height%	Quantity	NTP	Resolution	Symmetry Factor	Warning
1	Unknown	6	19.343	13434062	409960	91.634	90.674	N/A	8395	5.074	1.309	
2	Unknown	6	23.563	1226569	42165	8.366	9.326	N/A	13107	N/A	1.056	

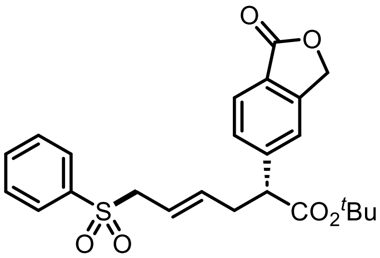

***tert*-Butyl (*R*,*E*)-2-(1-oxo-1,3-dihydroisobenzofuran-5-yl)-6-(phenylsulfonyl)hex-4-enoate (13)**. Colorless oil (Hex/EA = 1/1), 67%, 93:7 er, $[\alpha]_D^{23}$ = -38.3 (c = 0.10, $CHCl_3$). ^{1}H NMR (400 MHz, $CDCl_3$) δ 7.86 (d, *J* = 8.4 Hz, 1H), 7.76 (dd, *J* = 8.3, 1.3 Hz, 2H), 7.68 – 7.61 (m, 1H), 7.53 (t, *J* = 7.7 Hz, 2H), 7.42 – 7.34 (m, 2H), 5.56 – 5.39 (m, 2H), 5.30 (s, 2H), 3.68 (d, *J* = 6.7 Hz, 2H), 3.58 (t, *J* = 7.6 Hz, 1H), 2.79 (dt, *J* = 13.9, 6.9 Hz, 1H), 2.47 (dt, *J* = 14.0, 7.1 Hz, 1H), 1.38 (s, 9H). ^{13}C NMR (101 MHz, $CDCl_3$) δ 171.4, 170.9, 147.4, 145.5, 138.8, 137.4, 133.9, 129.2, 129.1, 128.4, 126.0, 125.1, 121.9, 119.0, 82.0, 69.7, 59.9, 52.3, 36.5, 28.0. IR (film) ν (cm^{-1}) 2978, 2932, 1760, 1724, 1619, 1446, 1368, 1306, 1241, 1147, 1086, 1045, 1003, 972, 845, 758, 733, 689, 594, 558, 528. HR-MS (ESI) m/z calcd for $C_{24}H_{26}NaO_6S^+$ 465.13423, found 465.12589, $[M+Na^+]$. The enantiomeric ratio of **13** was determined by HPLC analysis on Chiralpak OJ-H column. Conditions: hexane/isopropanol = 50/50, flow rate = 1.0 mL/min, uv-vis detection at λ = 220 nm, t_R = 21.7 min (major), 31.1 min (minor).

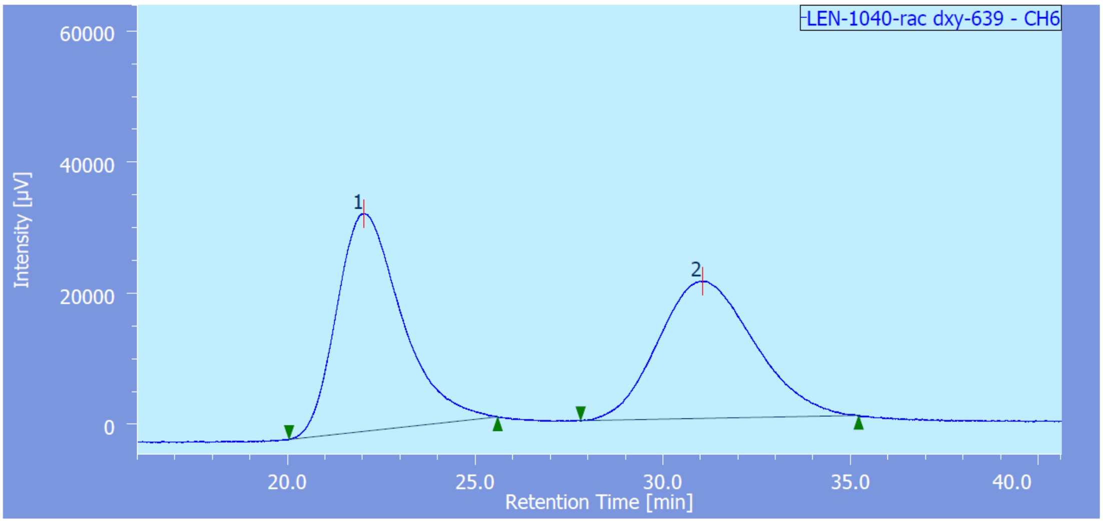

Decision

#	Peak Name	CH	tR [min]	Area [µV·sec]	Height [µV]	Area%	Height%	Quantity	NTP	Resolution	Symmetry Factor	Warning
1	Unknown	6	22.040	4012383	33203	51.802	61.323	N/A	808	2.301	1.411	
2	Unknown	6	31.043	3733264	20942	48.198	38.677	N/A	684	N/A	1.168	

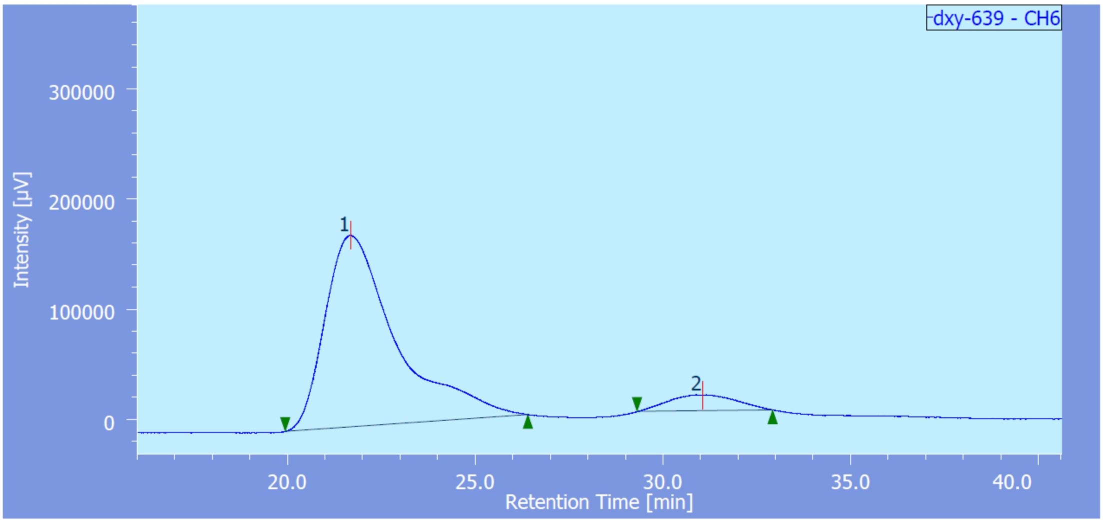

Decision

#	Peak Name	CH	tR [min]	Area [µV·sec]	Height [µV]	Area%	Height%	Quantity	NTP	Resolution	Symmetry Factor	Warning
1	Unknown	6	21.680	22887751	173719	92.669	92.364	N/A	803	2.800	1.905	
2	Unknown	6	31.050	1810596	14363	7.331	7.636	N/A	1158	N/A	1.036	

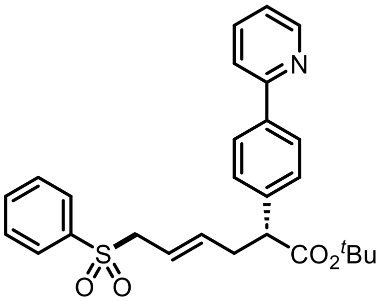

***tert*-Butyl (*R*,*E*)-6-(phenylsulfonyl)-2-(4-(pyridin-2-yl)phenyl)hex-4-enoate (14)**. Colorless

oil (Hex/EA = 1/1), 40%, 90:10 er, $[\alpha]_D^{23}$ = -13.5 (c = 0.17, $CHCl_3$). ^{1}H NMR (400 MHz, $CDCl_3$) δ 8.71 – 8.65 (m, 1H), 7.96 – 7.88 (m, 2H), 7.82 – 7.76 (m, 2H), 7.76 – 7.70 (m, 2H), 7.66 – 7.59 (m, 1H), 7.56 – 7.49 (m, 2H), 7.32 – 7.27 (m, 2H), 7.23 (ddd, *J* = 7.1, 4.9, 1.5 Hz, 1H), 5.52 – 5.40 (m, 2H), 3.73 – 3.65 (m, 2H), 3.45 (t, *J* = 7.7 Hz, 1H), 2.76 (dddd, *J* = 15.1, 7.9, 5.1, 2.2 Hz, 1H), 2.51 – 2.40 (m, 1H), 1.37 (s, 9H). ^{13}C NMR (101 MHz, $CDCl_3$) δ 172.1, 157.1, 149.7, 139.6, 138.5, 138.4, 138.1, 137.1, 133.8, 129.2, 128.6, 128.4, 127.3, 122.3, 120.7, 118.5, 81.3, 60.1, 51.9, 36.2, 28.0. IR (film) ν (cm^{-1}) 3061, 2978, 2932, 1721, 1586, 1467, 1446, 1436, 1367, 1318, 1307, 1241, 1146, 1086, 1016, 969, 844, 772, 732, 689, 596, 546, 528. HR-MS (ESI) m/z calcd for $C_{27}H_{30}NO_4S^+$ 464.18901, found 464.18204, [M+H$^+$]. The enantiomeric ratio of **14** was determined by HPLC analysis on Chiralpak AD-H column. Conditions: hexane/isopropanol = 50/50, flow rate = 1.0 mL/min, uv-vis detection at λ = 254 nm, t_R = 6.7 min (major), 9.0 min (minor).

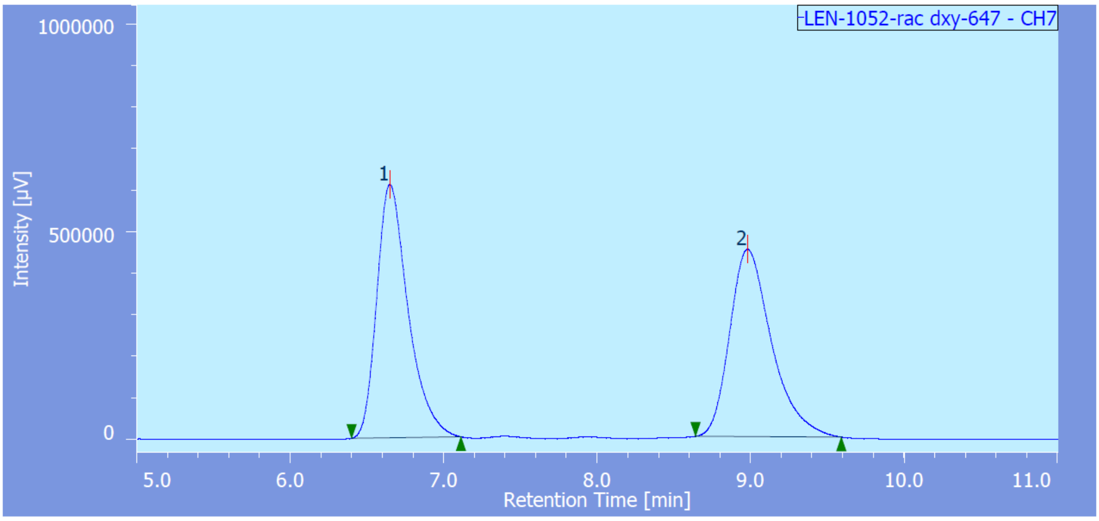

Decision

#	Peak Name	CH	tR [min]	Area [µV·sec]	Height [µV]	Area%	Height%	Quantity	NTP	Resolution	Symmetry Factor	Warning
1	Unknown	7	6.650	8656042	610423	50.276	57.529	N/A	5467	5.549	1.384	
2	Unknown	7	8.980	8561107	450655	49.724	42.471	N/A	5547	N/A	1.366	

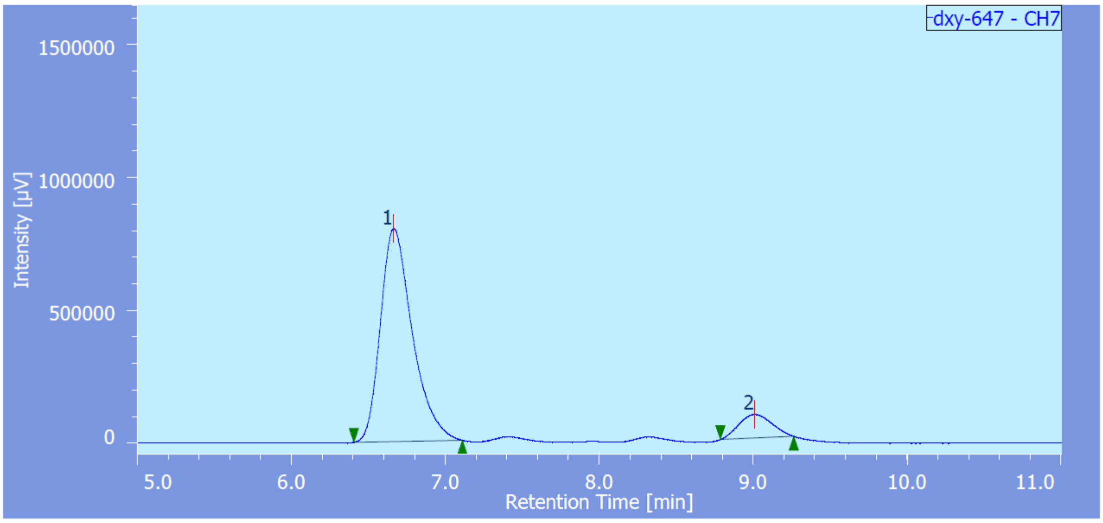

Decision

#	Peak Name	CH	tR [min]	Area [µV·sec]	Height [µV]	Area%	Height%	Quantity	NTP	Resolution	Symmetry Factor	Warning
1	Unknown	7	6.663	11541313	800876	89.649	89.995	N/A	5256	5.993	1.386	
2	Unknown	7	9.010	1332513	89039	10.351	10.005	N/A	7451	N/A	1.070	

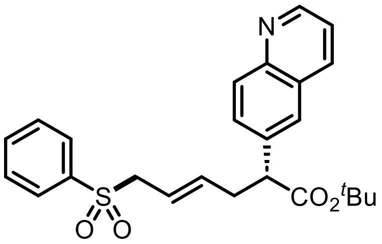

***tert* -Butyl (R,E)-6-(phenylsulfonyl)-2-(quinolin-6-yl)hex-4-enoate (15)**. Colorless oil (Hex/EA = 1/1), 55%, 91:9 er, $[\alpha]_D{}^{23}$ = -13.9 (c = 0.26, $CHCl_3$). ^{1}H NMR (500 MHz, $CDCl_3$) δ 8.91 (d, J = 4.5 Hz, 1H), 8.17 (dd, J = 34.7, 8.4 Hz, 2H), 7.75 – 7.70 (m, 2H), 7.70 – 7.67 (m, 1H), 7.64 – 7.57 (m, 2H), 7.50 – 7.43 (m, 3H), 5.49 (tq, J = 15.6, 8.9, 7.6 Hz, 2H), 3.68 (d, J = 6.5 Hz, 2H), 3.63 (t, J = 7.6 Hz, 1H), 2.84 (dt, J = 14.0, 6.9 Hz, 1H), 2.55 (dt, J = 14.1, 7.0 Hz, 1H), 1.37 (s, 9H). ^{13}C NMR (126 MHz, $CDCl_3$) δ 171.9, 149.8, 138.6, 137.8, 137.5, 137.2, 135.8, 133.8, 130.1, 129.3, 129.2, 128.6, 128.5, 126.8, 121.5, 118.8, 81.7, 60.0, 52.0, 36.3, 28.1. IR (film) ν (cm^{-1}) 2979, 2930, 2368, 2340, 2160, 2018, 1968, 1720, 1594, 1571, 1500, 1447, 1392, 1367, 1307, 1146, 1085, 969, 887, 843, 801, 769, 733, 689, 597, 560, 530. HR-MS (ESI) m/z calcd for $C_{25}H_{28}NO_4S^+$ 438.17336, found 438.16495, [M+H$^+$]. The enantiomeric ratio of **15** was determined by HPLC analysis on Chiralpak IC column. Conditions: hexane/isopropanol = 50/50, flow rate = 1.0 mL/min, uv-vis detection at λ = 220 nm, t_R = 32.3 min (minor), 35.9 min (major).

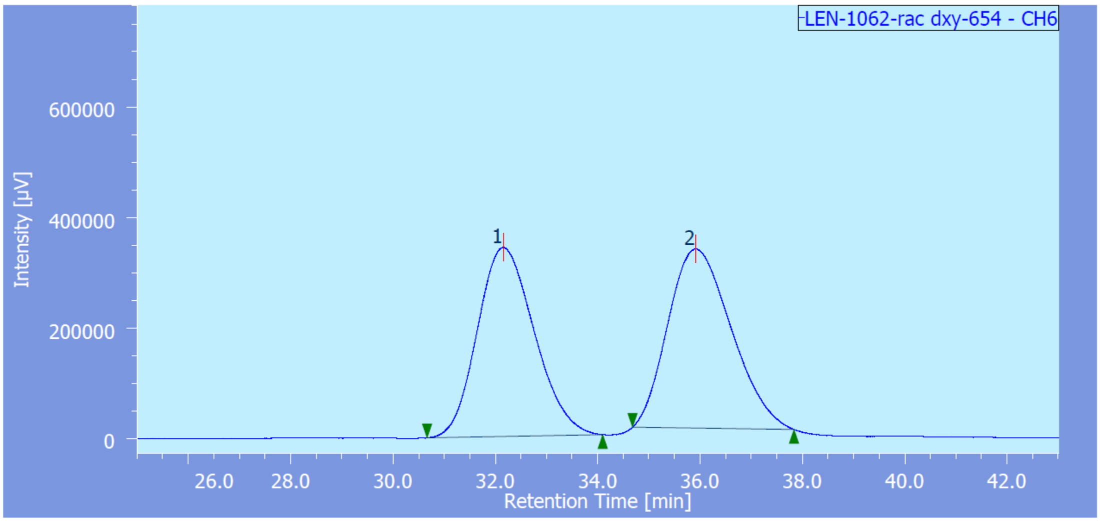

Decision

#	Peak Name	CH	tR [min]	Area [µV·sec]	Height [µV]	Area%	Height%	Quantity	NTP	Resolution	Symmetry Factor	Warning
1	Unknown	6	32.157	26438696	341375	48.716	51.343	N/A	3889	1.712	1.164	
2	Unknown	6	35.910	27832711	323510	51.284	48.657	N/A	3791	N/A	1.240	

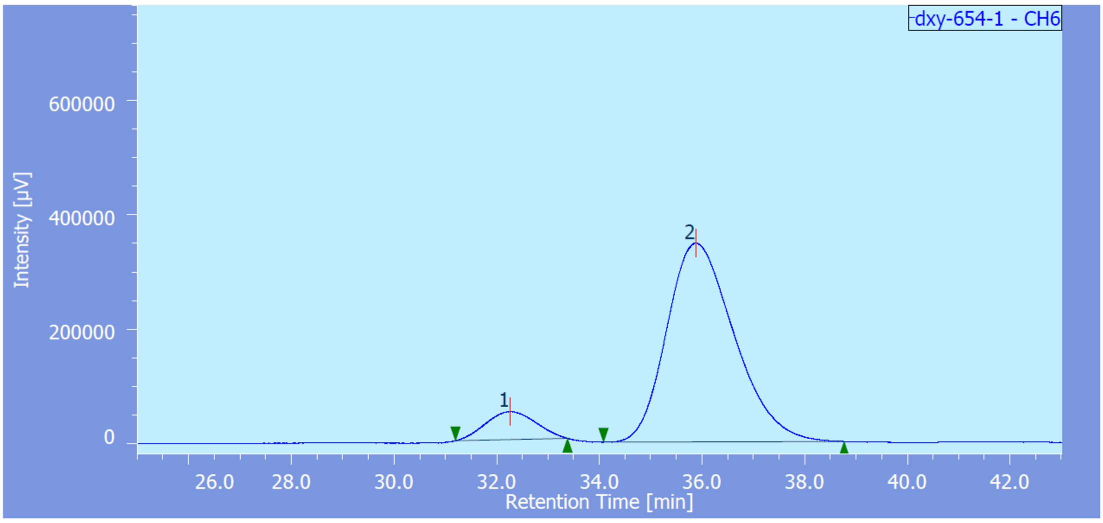

Decision

#	Peak Name	CH	tR [min]	Area [µV·sec]	Height [µV]	Area%	Height%	Quantity	NTP	Resolution	Symmetry Factor	Warning
1	Unknown	6	32.267	3308769	48817	9.413	12.304	N/A	4658	1.683	1.028	
2	Unknown	6	35.877	31842010	347945	90.587	87.696	N/A	3546	N/A	1.273	

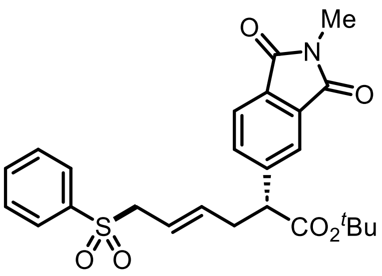

***tert*-Butyl (*R*,*E*)-2-(2-methyl-1,3-dioxoisoindolin-5-yl)-6-(phenylsulfonyl)hex-4-enoate (16)**. Colorless oil (Hex/EA = 1/1), 62%, 89:11 er, $[\alpha]_D{}^{23}$ = -28.0 (c = 0.083, $CHCl_3$). ^{1}H NMR (400 MHz, $CDCl_3$) δ 7.82 – 7.79 (m, 2H), 7.77 (dd, *J* = 7.6, 0.6 Hz, 1H), 7.69 – 7.64 (m, 2H),

7.57 – 7.52 (m, 3H), 5.53 – 5.37 (m, 2H), 3.68 (d, J = 6.4 Hz, 2H), 3.56 (t, J = 7.6 Hz, 1H), 3.17 (s, 3H), 2.80 (ddd, J = 14.0, 7.9, 6.0 Hz, 1H), 2.52 – 2.41 (m, 1H), 1.36 (s, 9H). ^{13}C NMR (101 MHz, $CDCl_3$) δ 171.0, 168.4, 168.3, 145.5, 138.6, 137.1, 133.9, 133.6, 133.0, 131.4, 129.2, 128.5, 123.6, 122.8, 119.3, 82.1, 59.9, 52.3, 36.2, 28.0, 24.1. IR (film) ν (cm^{-1}) 2977, 2929, 1771, 1709, 1622, 1585, 1445, 1428, 1380, 1318, 1307, 1268, 1248, 1147, 1086, 1009, 977, 845, 766, 733, 689, 598, 559, 530, 500. HR-MS (ESI) m/z calcd for $C_{25}H_{28}NO_6S^+$ 470.16319, found 470.15521, [M+H$^+$]. The enantiomeric ratio of **16** was determined by HPLC analysis on Chiralpak IC column. Conditions: hexane/isopropanol = 50/50, flow rate = 1.0 mL/min, uv-vis detection at λ = 254 nm, t_R = 50.1 min (minor), 58.3 min (major).

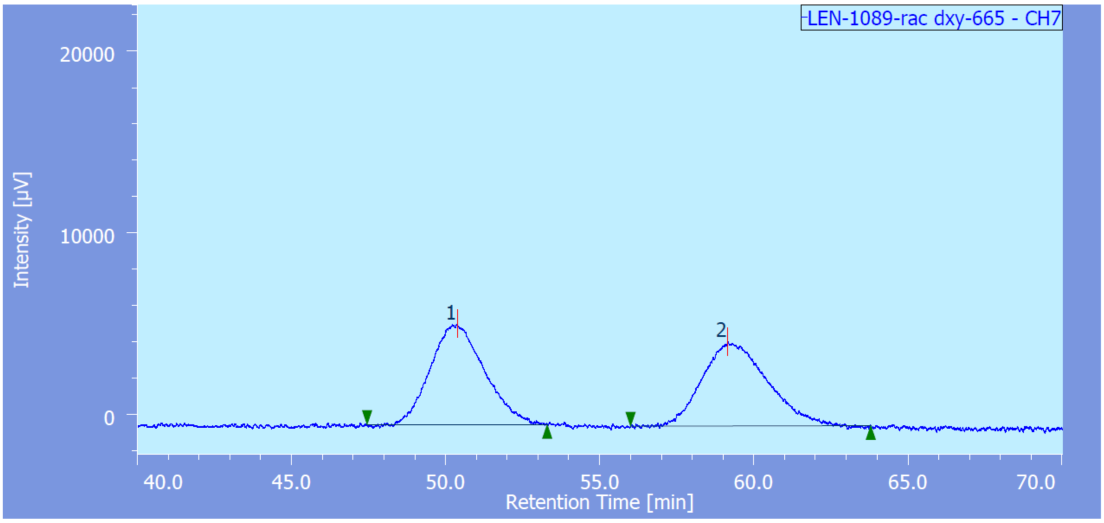

Decision

#	Peak Name	CH	tR [min]	Area [µV·sec]	Height [µV]	Area%	Height%	Quantity	NTP	Resolution	Symmetry Factor	Warning
1	Unknown	7	50.373	671183	5538	49.973	54.375	N/A	3925	2.474	1.143	
2	Unknown	7	59.147	671911	4646	50.027	45.625	N/A	3689	N/A	1.355	

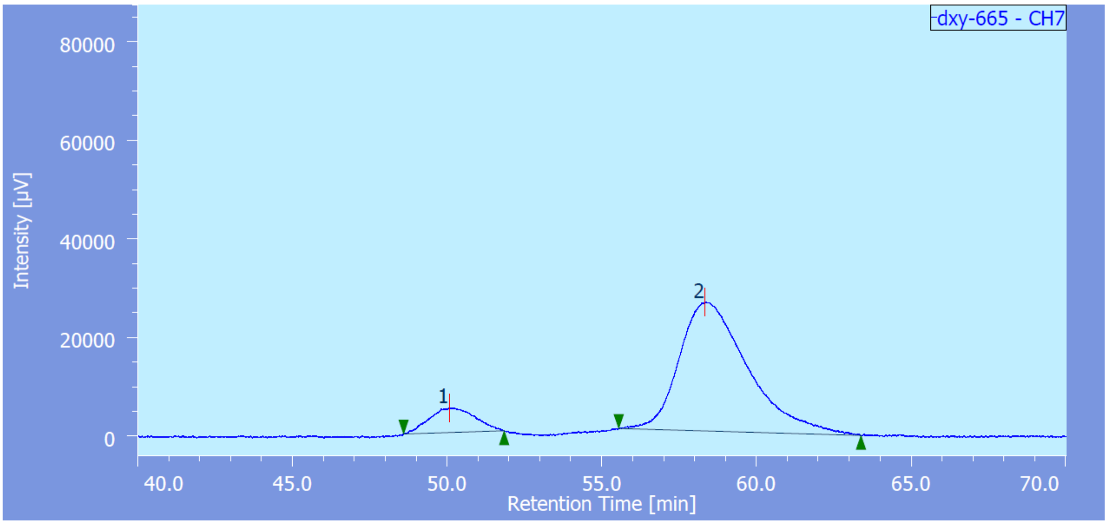

Decision

#	Peak Name	CH	tR [min]	Area [µV·sec]	Height [µV]	Area%	Height%	Quantity	NTP	Resolution	Symmetry Factor	Warning
1	Unknown	7	50.070	518914	5078	11.395	16.264	N/A	4986	2.466	1.099	
2	Unknown	7	58.340	4034819	26145	88.605	83.736	N/A	3599	N/A	1.504	

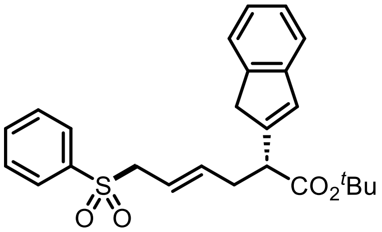

***tert*-Butyl (*R,E*)-2-(1H-inden-2-yl)-6-(phenylsulfonyl)hex-4-enoate (17)**. Colorless oil (Hex/EA = 2/1), 47%, 78:22 er, $[\alpha]_D^{23}$ = -12.6 (c = 0.18, $CHCl_3$). ^{1}H NMR (400 MHz, $CDCl_3$) δ 7.78 (dd, *J* = 8.4, 1.3 Hz, 2H), 7.63 – 7.57 (m, 1H), 7.49 – 7.44 (m, 2H), 7.41 (dd, *J* = 7.4, 1.0 Hz, 1H), 7.32 (dt, *J* = 7.5, 1.2 Hz, 1H), 7.28 – 7.23 (m, 1H), 7.16 (td, *J* = 7.3, 1.3 Hz, 1H), 6.64 – 6.60 (m, 1H), 5.57 – 5.44 (m, 2H), 3.72 (d, *J* = 6.0 Hz, 2H), 3.40 (t, *J* = 7.2 Hz, 1H), 3.35 (s, 2H), 2.71 – 2.61 (m, 1H), 2.49 – 2.40 (m, 1H), 1.41 (s, 9H). ^{13}C NMR (101 MHz, $CDCl_3$) δ 171.8, 145.5, 144.6, 143.2, 138.5, 138.2, 133.8, 129.2, 129.1, 128.5, 126.6, 124.6, 123.7, 120.8, 118.5, 81.4, 60.1, 48.4, 39.7, 35.0, 28.1. IR (film) ν (cm^{-1}) 3060, 2976, 2930, 1771, 1720, 1606, 1585, 1460, 1447, 1393, 1368, 1306, 1267, 1147, 1085, 1016, 973, 868, 844, 733, 702, 689, 596, 555, 529. HR-MS (ESI) m/z calcd for $C_{25}H_{28}NaO_4S^+$ 447.16005, found 447.15073, [M+Na$^+$]. The enantiomeric ratio of **17** was determined by HPLC analysis on Chiralpak AD-H column. Conditions: hexane/isopropanol = 60/40, flow rate = 1.0 mL/min, uv-vis detection at λ = 254 nm, t_R = 5.9 min (major), 6.8 min (minor).

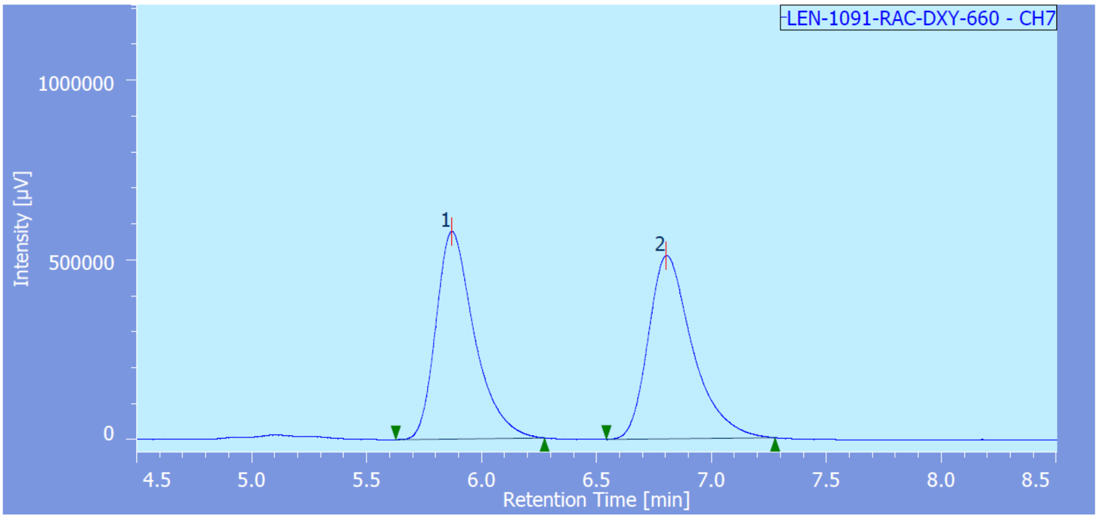

Decision

#	Peak Name	CH	tR [min]	Area [µV·sec]	Height [µV]	Area%	Height%	Quantity	NTP	Resolution	Symmetry Factor	Warning
1	Unknown	7	5.870	6724829	577761	49.581	53.127	N/A	6385	2.968	1.414	
2	Unknown	7	6.803	6838364	509741	50.419	46.873	N/A	6532	N/A	1.430	

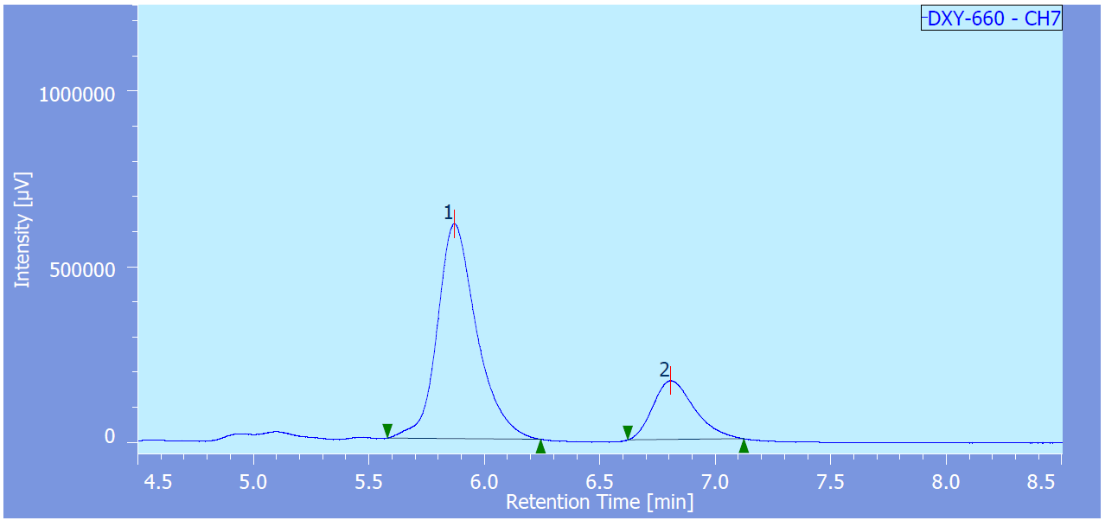

Decision

#	Peak Name	CH	tR [min]	Area [µV·sec]	Height [µV]	Area%	Height%	Quantity	NTP	Resolution	Symmetry Factor	Warning
1	Unknown	7	5.870	7255675	610838	77.679	78.513	N/A	6374	3.032	1.198	
2	Unknown	7	6.807	2084910	167170	22.321	21.487	N/A	7003	N/A	1.316	

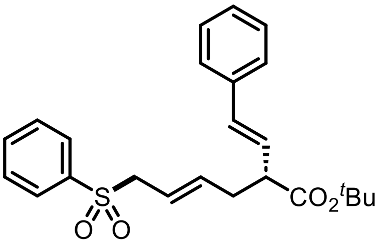

***tert*-Butyl (*R*,*E*)-6-(phenylsulfonyl)-2-((*E*)-styryl)hex-4-enoate (18)**. Colorless oil (Hex/EA = 2/1), 61%, 75:25 er, $[\alpha]_D^{23}$ = -15.5 (c = 0.21, $CHCl_3$). ^{1}H NMR (400 MHz, $CDCl_3$) δ 7.83 (dd, *J* = 8.4, 1.3 Hz, 2H), 7.64 – 7.59 (m, 1H), 7.50 (ddd, *J* = 8.1, 6.7, 1.3 Hz, 2H), 7.37 – 7.29 (m, 4H), 7.26 – 7.21 (m, 1H), 6.39 (dd, *J* = 15.9, 0.9 Hz, 1H), 6.08 (dd, *J* = 15.9, 8.7 Hz, 1H), 5.58 – 5.45 (m, 2H), 3.74 (d, *J* = 6.1 Hz, 2H), 3.06 – 2.97 (m, 1H), 2.57 – 2.47 (m, 1H), 2.37 – 2.27 (m, 1H), 1.43 (s, 9H). ^{13}C NMR (101 MHz, $CDCl_3$) δ 172.4, 138.6, 137.9, 136.9, 133.8, 132.6, 129.2, 128.7, 128.6, 127.8, 127.0, 126.5, 118.5, 81.3, 60.1, 49.9, 35.6, 28.2. IR (film) ν (cm^{-1}) 2978, 2925, 1720, 1478, 1447, 1392, 1367, 1318, 1307, 1146, 1086, 1026, 967, 845, 732, 689, 595, 554, 529, 505. HR-MS (ESI) m/z calcd for $C_{24}H_{28}NaO_4S^+$ 435.16005, found 435.15506, [M+Na$^+$]. The enantiomeric ratio of **18** was determined by HPLC analysis on Chiralpak AD-3 column. Conditions: hexane/isopropanol = 90/10, flow rate = 0.5 mL/min, uv-vis detection at λ = 210 nm, t_R = 30.0 min (major), 33.3 min (minor).

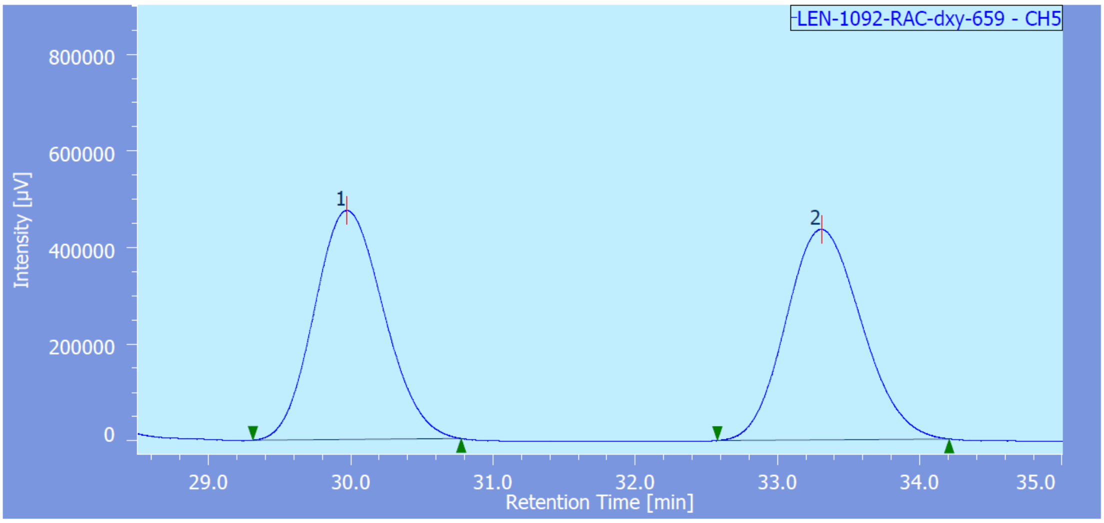

Decision

#	Peak Name	CH	tR [min]	Area [µV·sec]	Height [µV]	Area%	Height%	Quantity	NTP	Resolution	Symmetry Factor	Warning
1	Unknown	5	29.973	16101487	473864	49.953	52.100	N/A	17368	3.521	1.120	
2	Unknown	5	33.307	16131834	435669	50.047	47.900	N/A	18162	N/A	1.123	

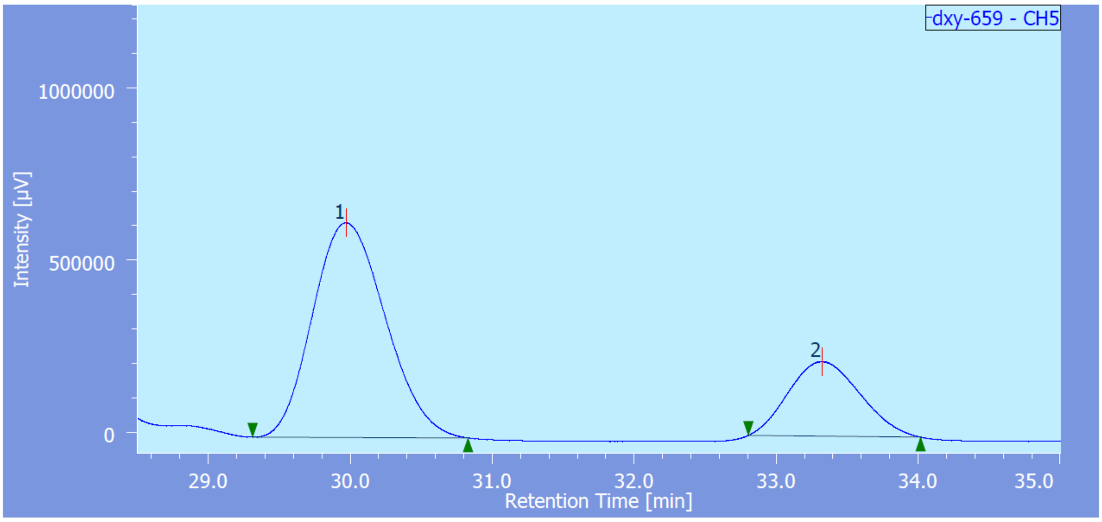

Decision

#	Peak Name	CH	tR [min]	Area [µV·sec]	Height [µV]	Area%	Height%	Quantity	NTP	Resolution	Symmetry Factor	Warning
1	Unknown	5	29.970	22220682	623263	74.580	74.220	N/A	15588	3.498	1.162	
2	Unknown	5	33.320	7573675	216489	25.420	25.780	N/A	19259	N/A	1.142	

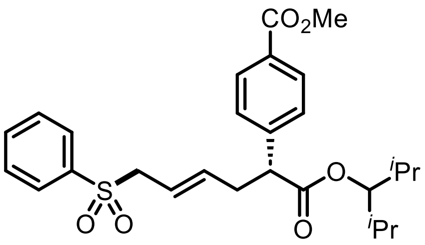

Methyl (*R*,*E*)-4-(1-((2,4-dimethylpentan-3-yl)oxy)-1-oxo-6-(phenylsulfonyl)hex-4-en-2-yl)benzoate (19). Colorless oil (Hex/EA = 2/1), 70% yield, 93:7 er, $[\alpha]_D^{24}$ = -13.4 (c = 0.22, $CHCl_3$). ^1H NMR (400 MHz, $CDCl_3$) δ 8.03 (d, *J* = 8.4 Hz, 2H), 7.85 – 7.78 (m, 2H), 7.74 –

7.66 (m, 1H), 7.58 (ddd, J = 8.0, 6.6, 1.3 Hz, 2H), 7.40 – 7.33 (m, 2H), 5.62 – 5.41 (m, 2H), 4.56 (t, J = 6.1 Hz, 1H), 3.96 (s, 3H), 3.73 (d, J = 6.3 Hz, 2H), 3.64 (t, J = 7.7 Hz, 1H), 2.88 (ddd, J = 14.0, 7.6, 5.4 Hz, 1H), 2.63 – 2.49 (m, 1H), 1.94 – 1.76 (m, 2H), 0.83 (dd, J = 11.8, 6.7 Hz, 6H), 0.65 (dd, J = 17.9, 6.8 Hz, 6H). ^{13}C NMR (101 MHz, $CDCl_3$) δ 172.4, 166.9, 143.4, 138.5, 137.5, 133.8, 130.0, 129.5, 129.2, 128.5, 128.3, 119.1, 83.7, 60.0, 52.3, 51.6, 35.7, 29.5, 29.4, 19.5, 19.5, 17.5, 16.8. IR (film) ν (cm^{-1}) 2965, 1720, 1610, 1464, 1446, 1436, 1389, 1370, 1308, 1278, 1173, 1151, 1134, 1110, 1086, 1020, 999, 969, 939, 898, 865, 770, 734, 703, 689, 596, 555, 529. HR-MS (ESI) m/z calcd for $C_{27}H_{34}NaO_6S^+$ 509.19683, found 509.19833, [M+Na$^+$]. The enantiomeric ratio of **19** was determined by HPLC analysis on Chiralpak IC column. Conditions: hexane/isopropanol = 70/30, flow rate = 0.8 mL/min, uv-vis detection at λ = 254 nm, t_R = 47.8 min (minor), 55.7 min (major).

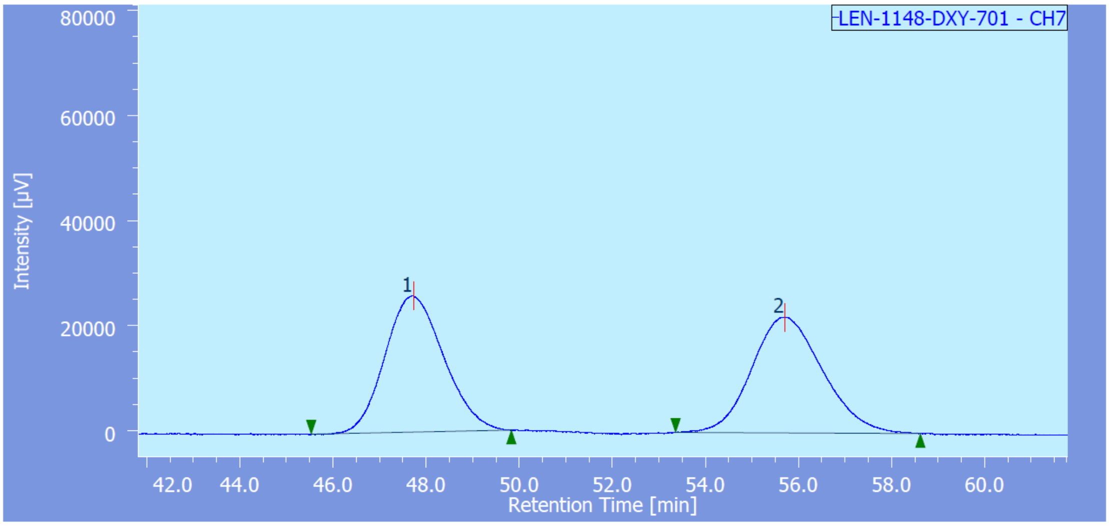

Decision

#	Peak Name	CH	tR [min]	Area [µV·sec]	Height [µV]	Area%	Height%	Quantity	NTP	Resolution	Symmetry Factor	Warning
1	Unknown	7	47.723	2275711	25922	49.336	54.065	N/A	6719	3.131	1.115	
2	Unknown	7	55.703	2336978	22024	50.664	45.935	N/A	6414	N/A	1.135	

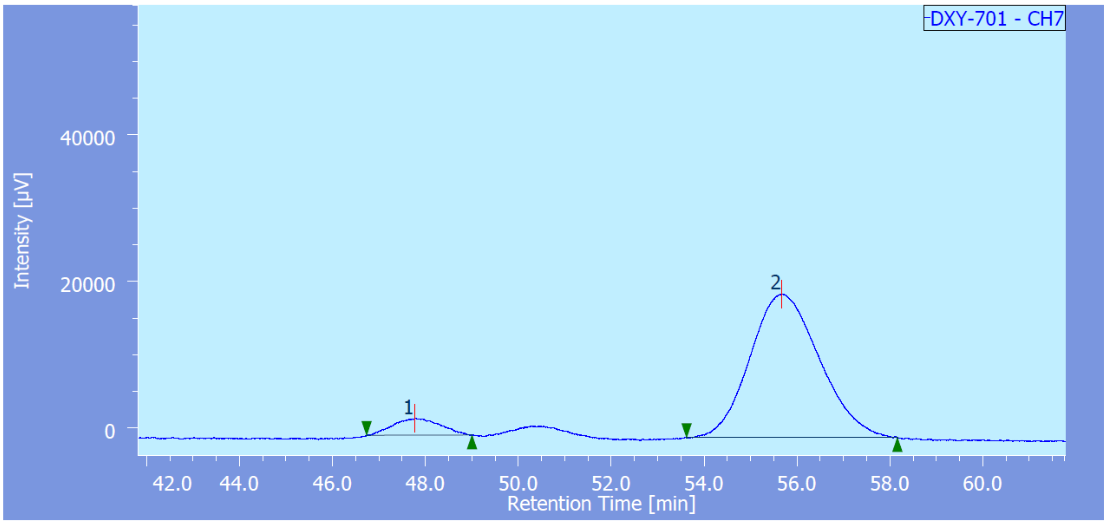

Decision

#	Peak Name	CH	tR [min]	Area [µV·sec]	Height [µV]	Area%	Height%	Quantity	NTP	Resolution	Symmetry Factor	Warning
1	Unknown	7	47.770	162614	2288	7.413	10.459	N/A	9088	3.328	1.106	
2	Unknown	7	55.670	2031090	19590	92.587	89.541	N/A	6526	N/A	1.134	

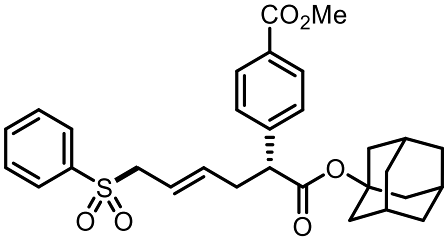

Methyl 4-((*R*,*E*)-1-(((3S,5S,7S)-adamantan-1-yl)oxy)-1-oxo-6-(phenylsulfonyl)hex-4-en-2-yl)benzoate (20). Colorless oil (Hex/EA = 2/1), 55% yield, 90:10 er, $[\alpha]_D^{23}$ = -5.0 (c = 0.20, $CHCl_3$). ^1H NMR (500 MHz, $CDCl_3$) δ 7.97 (d, *J* = 8.1 Hz, 2H), 7.78 (d, *J* = 7.9 Hz, 2H), 7.64 (t, *J* = 7.5 Hz, 1H), 7.53 (t, *J* = 7.8 Hz, 2H), 7.29 – 7.24 (m, 2H), 5.44 (q, *J* = 5.6 Hz, 2H), 3.91 (s, 3H), 3.69 (d, *J* = 5.9 Hz, 2H), 3.45 (t, *J* = 7.6 Hz, 1H), 2.78 – 2.70 (m, 1H), 2.48 – 2.36 (m, 1H), 2.11 (s, 3H), 1.98 (d, *J* = 2.7 Hz, 6H), 1.61 (s, 6H). ^{13}C NMR (126 MHz, $CDCl_3$) δ 171.3, 167.0, 144.0, 138.5, 137.8, 133.8, 130.0, 129.3, 129.2, 128.6, 128.0, 118.8, 81.7, 60.0, 52.3, 52.2, 41.2, 36.2, 36.1, 30.9. IR (film): ν (cm^{-1}) 3646, 3328, 2980, 1734, 1638, 1543, 1285, 1141, 1084, 738. HR-MS (ESI) m/z calcd for $C_{30}H_{34}NaO_6S^+$ 545.19683, found 545.19769, $[M+Na^+]$. The enantiomeric ratio of **20** was determined by HPLC analysis on Chiralpak AD-H column. Conditions: hexane/isopropanol = 80/20, flow rate = 1.0 mL/min, uv-vis detection at λ = 254 nm, t_R = 15.4 min (major), 20.8 min (minor).

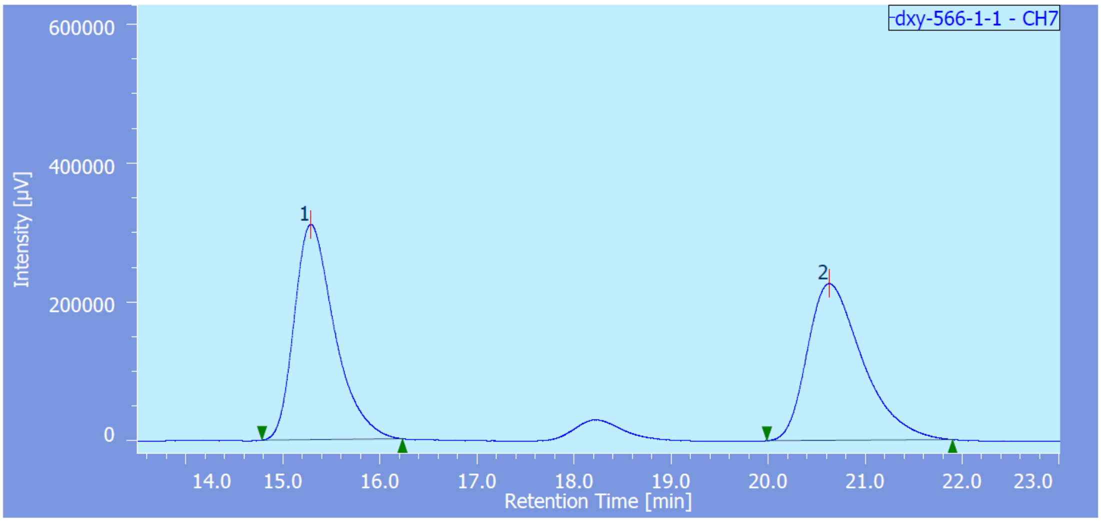

Decision

#	Peak Name	CH	tR [min]	Area [µV·sec]	Height [µV]	Area%	Height%	Quantity	NTP	Resolution	Symmetry Factor	Warning
1	Unknown	7	15.287	8970135	310007	50.086	57.843	N/A	6853	6.101	1.425	
2	Unknown	7	20.627	8939260	225943	49.914	42.157	N/A	6587	N/A	1.478	

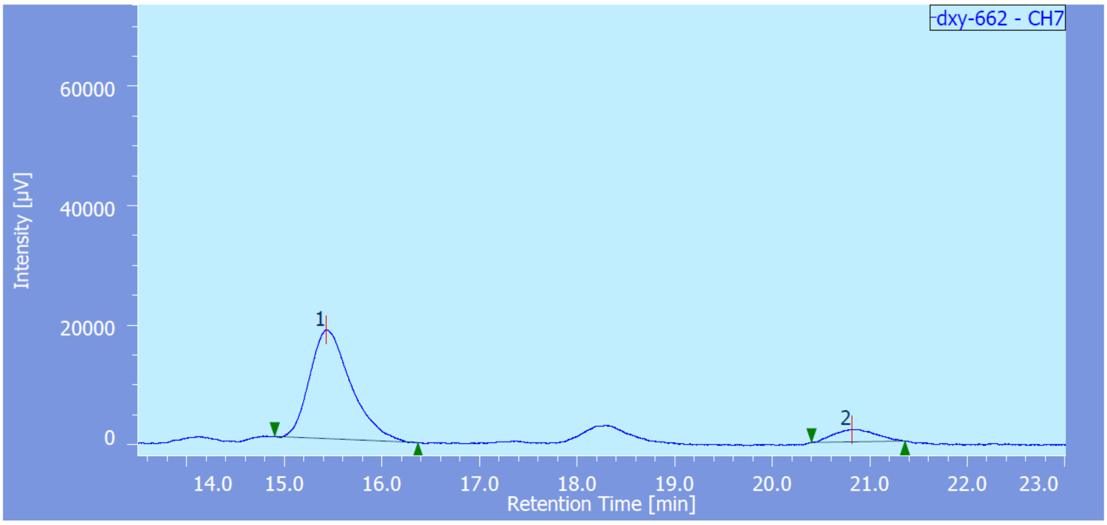

Decision

#	Peak Name	CH	tR [min]	Area [µV·sec]	Height [µV]	Area%	Height%	Quantity	NTP	Resolution	Symmetry Factor	Warning
1	Unknown	7	15.430	533835	18161	89.510	89.783	N/A	6655	6.638	1.396	
2	Unknown	7	20.817	62564	2067	10.490	10.217	N/A	9147	N/A	1.202	

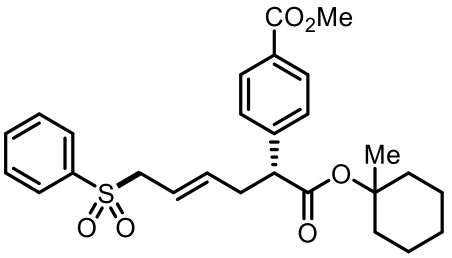

Methyl (*R*,*E*)-4-(1-((1-methylcyclohexyl)oxy)-1-oxo-6-(phenylsulfonyl)hex-4-en-2-yl)benzoate (21). Colorless oil (Hex/EA = 2/1), 43% yield, 90:10 er, $[\alpha]_D^{23}$ = -22.6 (c = 0.18, $CHCl_3$). ^{1}H NMR (400 MHz, $CDCl_3$) δ 7.97 (d, *J* = 8.5 Hz, 2H), 7.77 (dd, *J* = 8.4, 1.4 Hz, 2H),

7.67 – 7.59 (m, 1H), 7.52 (dd, J = 8.4, 6.9 Hz, 2H), 7.29 (d, J = 8.5 Hz, 2H), 5.51 – 5.37 (m, 2H), 3.91 (s, 3H), 3.70 – 3.65 (m, 2H), 3.50 (t, J = 7.7 Hz, 1H), 2.83 – 2.75 (m, 1H), 2.52 – 2.41 (m, 1H), 2.07 – 1.98 (m, 2H), 1.43 – 1.24 (m, 9H), 1.18 – 1.01 (m, 2H). ^{13}C NMR (101 MHz, $CDCl_3$) δ 171.4, 167.0, 143.9, 138.5, 137.8, 133.8, 130.0, 129.3, 129.2, 128.5, 128.1, 118.8, 83.1, 60.0, 52.3, 52.3, 37.1, 36.1, 35.7, 25.6, 25.3, 22.0, 21.9. IR (film) ν (cm^{-1}) 2932, 2860, 1718, 1609, 1585, 1446, 1436, 1417, 1376, 1308, 1278, 1248, 1180, 1146, 1107, 1087, 1042, 1019, 963, 928, 869, 812, 734, 702, 689, 598, 554, 529, 504. HR-MS (ESI) m/z calcd for $C_{27}H_{32}NaO_6S^+$ 507.18118, found 507.17793, [M+Na$^+$]. The enantiomeric ratio of **21** was determined by HPLC analysis on Chiralpak AD-H column. Conditions: hexane/isopropanol = 90/10, flow rate = 1.0 mL/min, uv-vis detection at λ = 220 nm, t_R = 32.5 min (major), 36.4 min (minor).

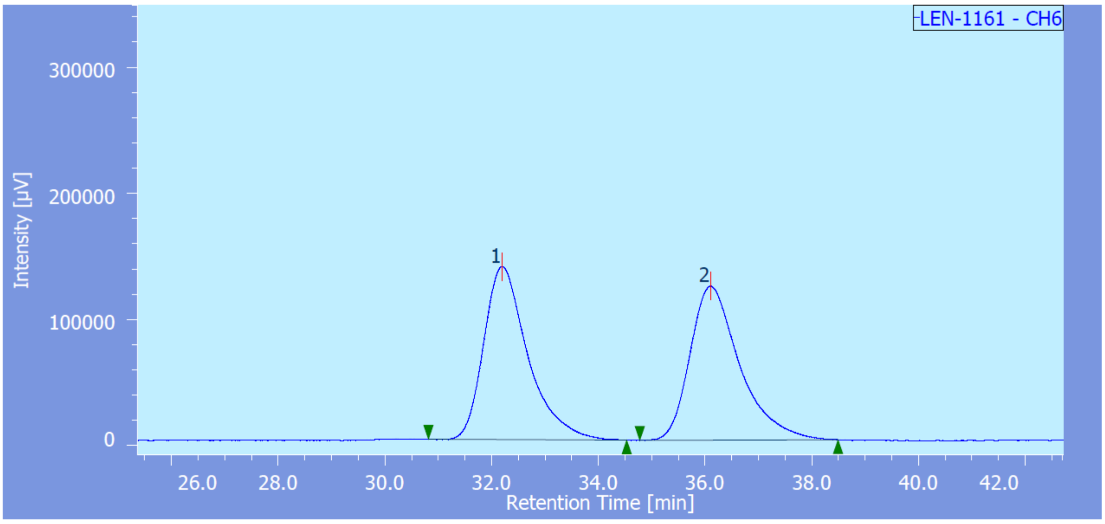

Decision

#	Peak Name	CH	tR [min]	Area [µV·sec]	Height [µV]	Area%	Height%	Quantity	NTP	Resolution	Symmetry Factor	Warning
1	Unknown	6	32.193	7887678	137065	49.948	52.892	N/A	8073	2.566	1.442	
2	Unknown	6	36.097	7904127	122075	50.052	47.108	N/A	7973	N/A	1.454	

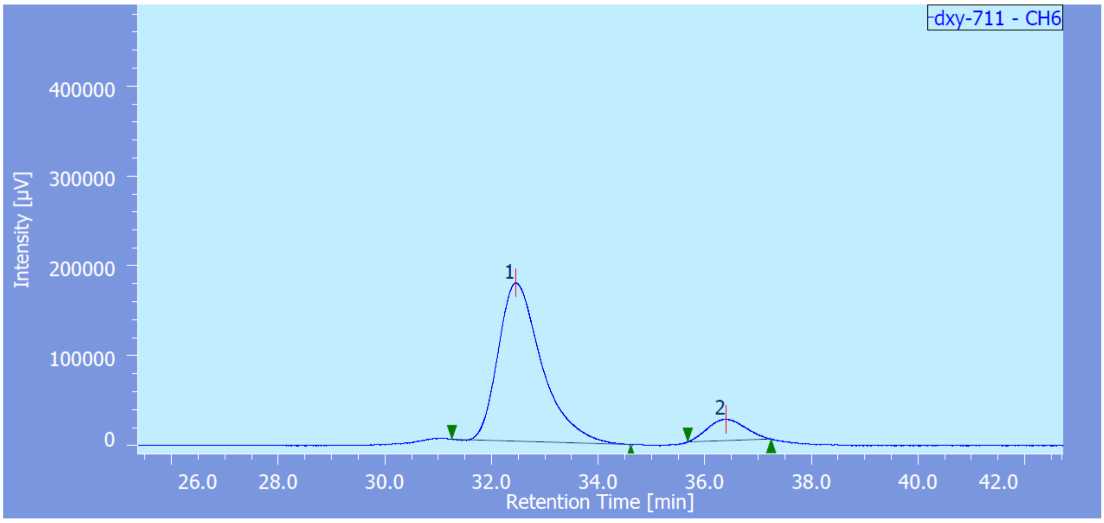

Decision

#	Peak Name	CH	tR [min]	Area [µV·sec]	Height [µV]	Area%	Height%	Quantity	NTP	Resolution	Symmetry Factor	Warning
1	Unknown	6	32.457	9988590	176009	89.545	88.117	N/A	8271	2.829	1.466	
2	Unknown	6	36.403	1166267	23735	10.455	11.883	N/A	11291	N/A	1.068	

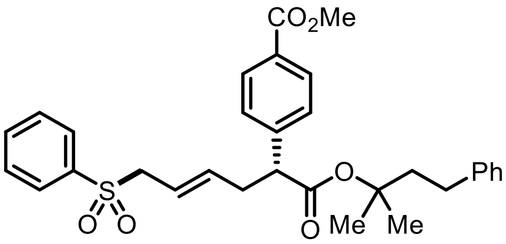

Methyl (*R,E*)-4-(1-((2-methyl-4-phenylbutan-2-yl)oxy)-1-oxo-6-(phenylsulfonyl)hex-4-en-2-yl)benzoate (22). Colorless oil (Hex/EA = 2/1), 49%, 85:15 er, $[\alpha]_D^{23}$ = -17.5 (c = 0.21, $CHCl_3$). ^1H NMR (400 MHz, $CDCl_3$) δ 7.98 (d, *J* = 8.4 Hz, 2H), 7.82 – 7.72 (m, 2H), 7.68 – 7.59 (m, 1H), 7.52 (dd, *J* = 8.3, 6.9 Hz, 2H), 7.33 – 7.22 (m, 4H), 7.20 – 7.13 (m, 1H), 7.04 (dd, *J* = 8.2, 1.4 Hz, 2H), 5.53 – 5.38 (m, 2H), 3.90 (s, 3H), 3.67 (d, *J* = 5.9 Hz, 2H), 3.49 (t, *J* = 7.7 Hz, 1H), 2.83 – 2.73 (m, 1H), 2.55 – 2.43 (m, 1H), 2.43 – 2.32 (m, 2H), 1.96 (dd, *J* = 9.8, 7.6 Hz, 2H), 1.40 (d, *J* = 7.8 Hz, 6H). ^{13}C NMR (101 MHz, $CDCl_3$) δ 171.5, 166.9, 143.8, 141.9, 138.5, 137.7, 133.8, 130.1, 129.4, 129.2, 128.5, 128.5, 128.4, 128.0, 126.0, 118.9, 83.3, 60.0, 52.3, 52.1, 42.8, 35.9, 30.2, 26.1, 25.9. IR (film) ν (cm^{-1}) 2922, 1719, 1609, 1446, 1436, 1369, 1308, 1279, 1180, 1151, 1138, 1114, 1086, 1072, 1020, 967, 862, 822, 764, 733, 699, 689, 596, 556, 529. HR-MS (ESI) m/z calcd for $C_{31}H_{34}NaO_6S^+$ 557.19683, found 557.19775, $[M+Na^+]$. The enantiomeric ratio of **22** was determined by HPLC analysis on Chiralpak IC column. Conditions: hexane/isopropanol = 60/40, flow rate = 1.0 mL/min, uv-vis detection at λ = 254 nm, t_R = 36.2 min (minor), 41.9 min (major).

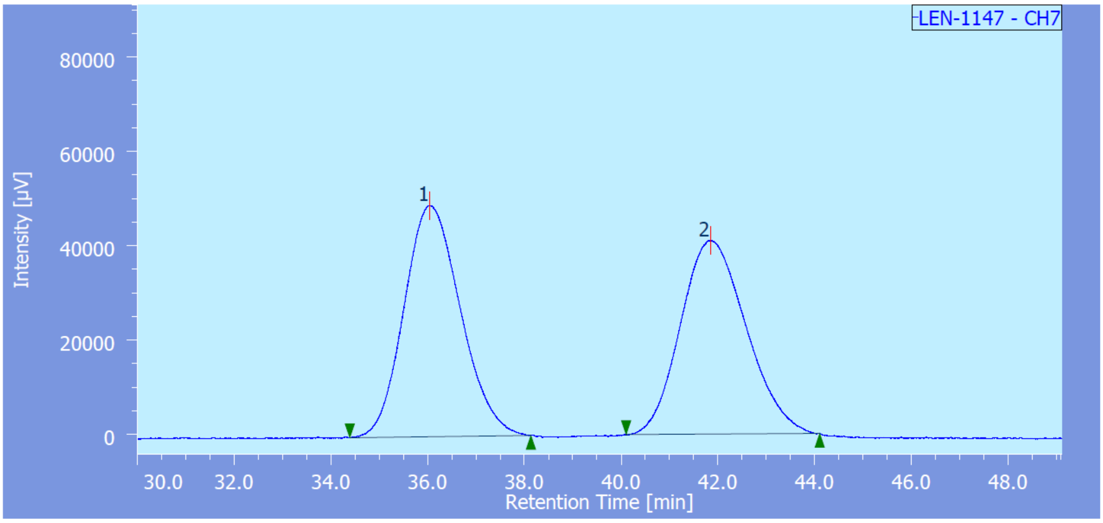

Decision

#	Peak Name	CH	tR [min]	Area [μV·sec]	Height [μV]	Area%	Height%	Quantity	NTP	Resolution	Symmetry Factor	Warning
1	Unknown	7	36.047	3976767	48993	50.664	54.403	N/A	4486	2.487	1.149	
2	Unknown	7	41.840	3872574	41062	49.336	45.597	N/A	4413	N/A	1.164	

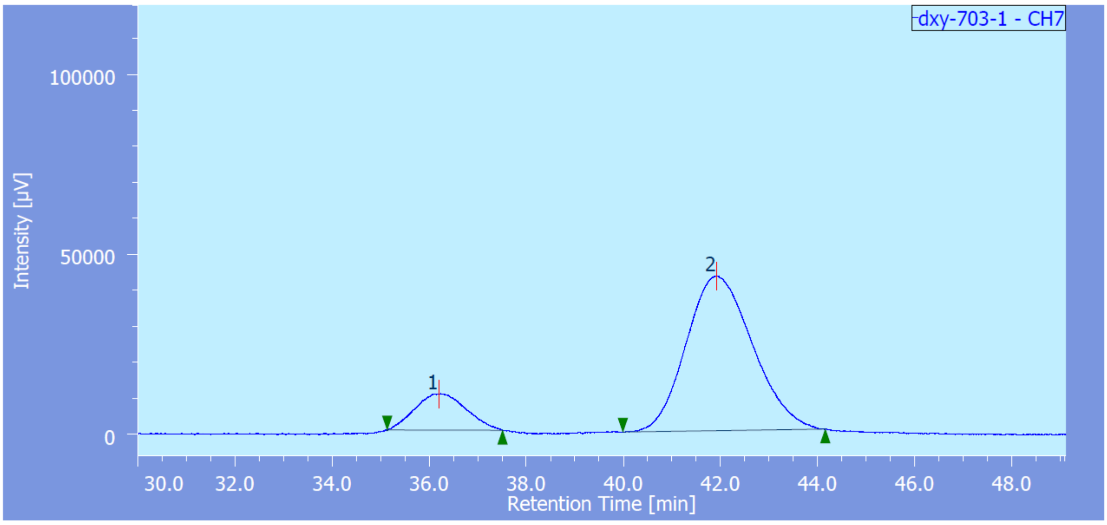

Decision

#	Peak Name	CH	tR [min]	Area [μV·sec]	Height [μV]	Area%	Height%	Quantity	NTP	Resolution	Symmetry Factor	Warning
1	Unknown	7	36.203	741456	10108	15.327	19.033	N/A	5012	2.497	1.118	
2	Unknown	7	41.920	4095991	43003	84.673	80.967	N/A	4339	N/A	1.155	

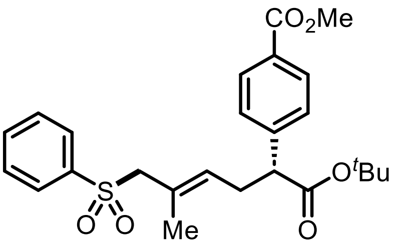

Methyl (*R*,*E*)-4-(1-(*tert*-butoxy)-5-methyl-1-oxo-6-(phenylsulfonyl)hex-4-en-2-yl)benzoate (23). Colorless oil (Hex/EA = 2/1), 40%,93:7 er, $[\alpha]_D^{24}$ = -20.4 (c = 0.19, $CHCl_3$). ^1H NMR (400 MHz, $CDCl_3$) δ 7.95 (d, J = 7.9 Hz, 2H), 7.78 (dt, J = 8.4, 1.0 Hz, 2H), 7.63 (dd,

J = 7.4, 0.9 Hz, 1H), 7.51 (t, J = 7.9 Hz, 2H), 7.28 – 7.24 (m, 2H), 5.02 (t, J = 7.2 Hz, 1H), 3.90 (d, J = 1.0 Hz, 3H), 3.65 (s, 2H), 3.38 (t, J = 7.7 Hz, 1H), 2.70 (dt, J = 14.9, 7.4 Hz, 1H), 2.38 (dt, J = 14.9, 7.1 Hz, 1H), 1.69 (s, 3H), 1.35 (s, 9H). ^{13}C NMR (101 MHz, $CDCl_3$) δ 171.9, 167.0, 144.1, 138.7, 133.7, 132.4, 130.0, 129.2, 129.1, 128.5, 128.0, 125.9, 81.5, 66.1, 52.3, 52.1, 32.1, 28.0, 17.2. IR (film) ν (cm^{-1}) 2919, 1720, 1609, 1447, 1393, 1368, 1308, 1280, 1147, 1110, 1086, 1020, 846, 740, 689, 601, 562, 530, 508. HR-MS (ESI) m/z calcd for $C_{25}H_{30}NaO_6S^+$ 481.16553, found 481.16629, [M+Na$^+$]. The enantiomeric ratio of **23** was determined by HPLC analysis on Chiralpak AD-H column. Conditions: hexane/isopropanol = 80/20, flow rate = 1.0 mL/min, uv-vis detection at λ = 254 nm, t_R = 9.7 min (major), 11.5 min (minor).

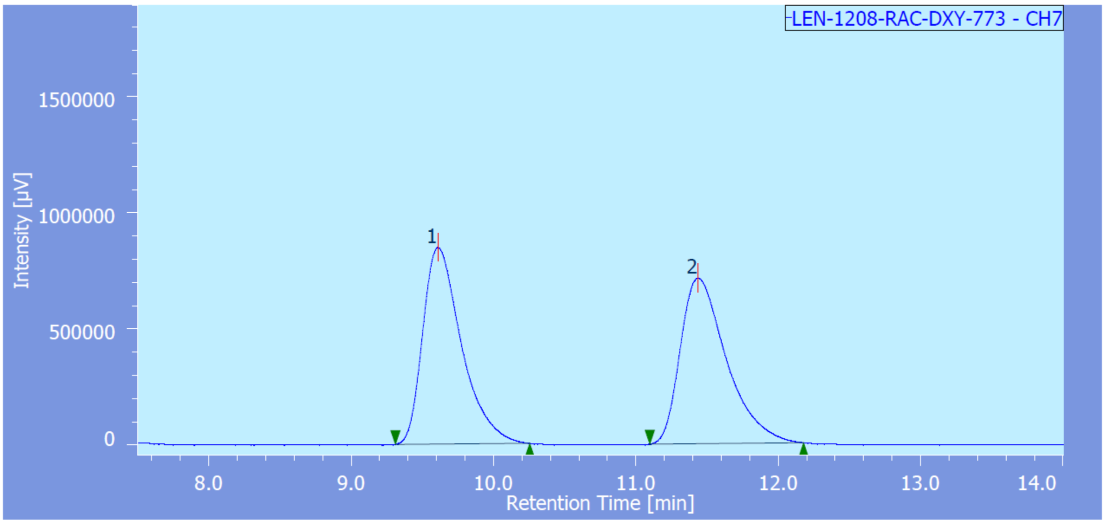

Decision

#	Peak Name	CH	tR [min]	Area [µV·sec]	Height [µV]	Area%	Height%	Quantity	NTP	Resolution	Symmetry Factor	Warning
1	Unknown	7	9.610	15992250	845338	49.861	54.234	N/A	6347	3.457	1.509	
2	Unknown	7	11.437	16081718	713361	50.139	45.766	N/A	6281	N/A	1.546	

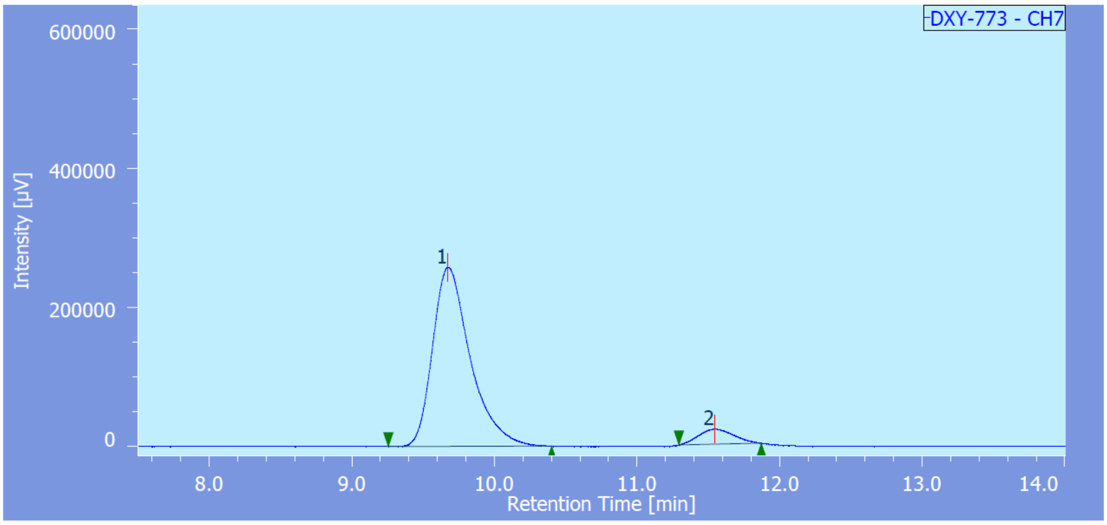

Decision

#	Peak Name	CH	tR [min]	Area [µV·sec]	Height [µV]	Area%	Height%	Quantity	NTP	Resolution	Symmetry Factor	Warning
1	Unknown	7	9.673	4734370	257453	92.699	92.274	N/A	7064	4.013	1.448	
2	Unknown	7	11.547	372859	21558	7.301	7.726	N/A	9424	N/A	1.134	

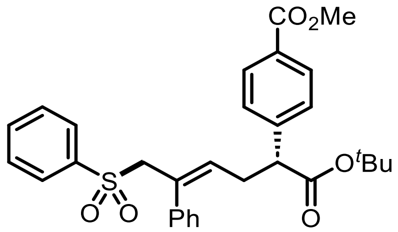

Methyl (*R,E*)-4-(1-(*tert*-butoxy)-1-oxo-5-phenyl-6-(phenylsulfonyl)hex-4-en-2-yl)benzoate (24). Colorless oil (Hex/EA = 2/1), 42%, 92:8 er, $[\alpha]_D^{23}$ = -55.0 (c = 0.13, $CHCl_3$). ^{1}H NMR (500 MHz, $CDCl_3$) δ 7.97 (d, *J* = 8.4 Hz, 2H), 7.64 – 7.57 (m, 3H), 7.49 – 7.43 (m, 2H), 7.22 (dd, *J* = 7.7, 3.4 Hz, 5H), 6.69 – 6.61 (m, 2H), 5.48 (t, *J* = 7.8 Hz, 1H), 3.91 (s, 3H), 3.63 (dd, *J* = 7.8, 2.4 Hz, 2H), 3.42 (t, *J* = 7.6 Hz, 1H), 3.10 (ddd, *J* = 14.6, 7.5, 1.1 Hz, 1H), 2.72 (dd, *J* = 14.9, 7.5 Hz, 1H), 1.32 (s, 9H). ^{13}C NMR (126 MHz, $CDCl_3$) δ 171.6, 167.0, 147.5, 143.9, 138.6, 137.7, 133.6, 132.0, 130.0, 129.1, 128.6, 128.5, 128.1, 128.0, 127.9, 115.5, 81.5, 56.7, 52.3, 50.4, 42.5, 28.0. IR (film) ν (cm^{-1}) 2980, 2119, 1719, 1610, 1446, 1368, 1309, 1281, 1147, 1112, 1085, 742, 689, 590, 546, 535, 505. HR-MS (ESI) m/z calcd for $C_{30}H_{32}NaO_6S^+$ 543.18118, found 543.18207, [M+Na$^+$]. The enantiomeric ratio of **24** was determined by HPLC analysis on Chiralpak NR-3 column. Conditions: hexane/isopropanol = 50/50, flow rate = 1.0 mL/min, uv-vis detection at λ = 254 nm, t_R = 48.7 min (major), 53.4 min (minor).

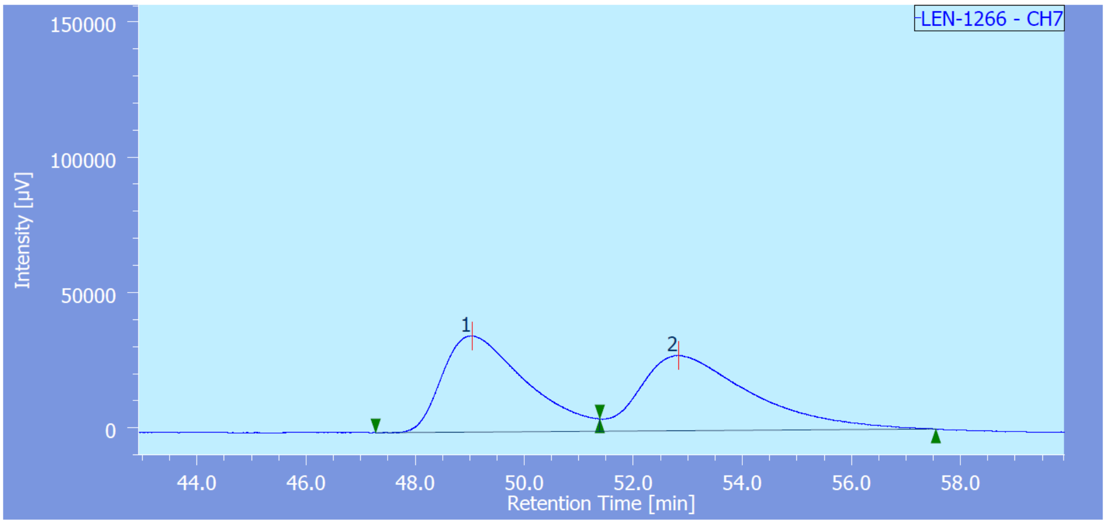

#	Peak Name	CH	tR [min]	Area [µV·sec]	Height [µV]	Area%	Height%	Quantity	NTP	Resolution	Symmetry Factor	Warning
1	Unknown	7	49.043	3788233	35534	48.923	56.143	N/A	4893	1.190	N/A	
2	Unknown	7	52.827	3955028	27758	51.077	43.857	N/A	3504	N/A	N/A	

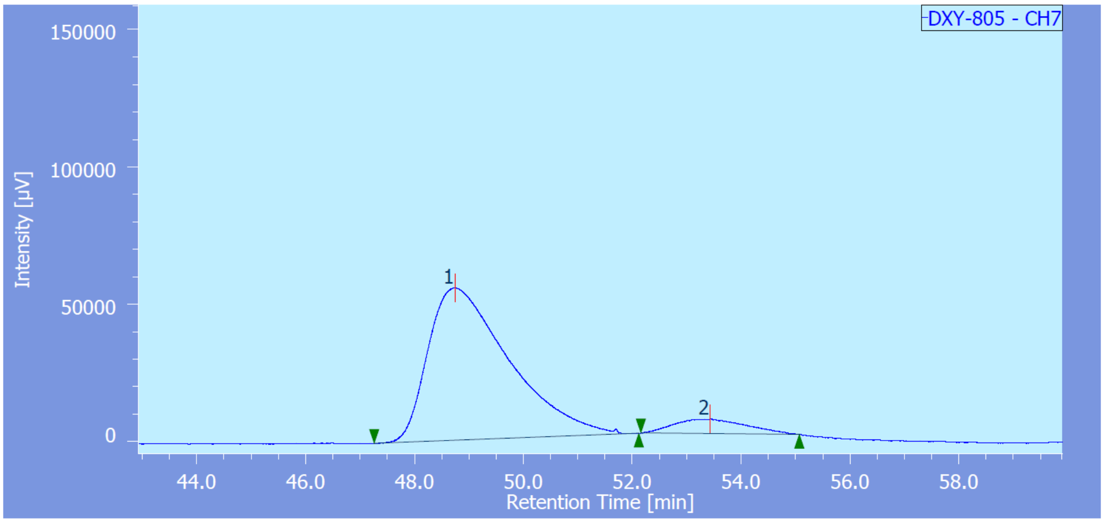

#	Peak Name	CH	tR [min]	Area [µV·sec]	Height [µV]	Area%	Height%	Quantity	NTP	Resolution	Symmetry Factor	Warning
1	Unknown	7	48.740	5735567	55393	92.096	91.210	N/A	5268	1.766	1.837	
2	Unknown	7	53.427	492214	5338	7.904	8.790	N/A	6570	N/A	1.138	

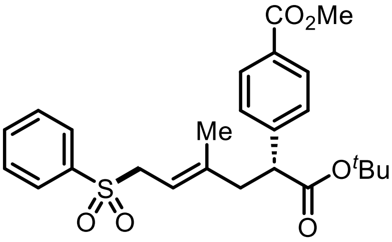

Methyl (*R*,*E*)-4-(1-(*tert*-butoxy)-4-methyl-1-oxo-6-(phenylsulfonyl)hex-4-en-2-yl)benzoate (25). Colorless oil (Hex/EA = 2/1), 48%, 93:7 er, $[\alpha]_D^{23}$ = -15.4 (c = 0.21, $CHCl_3$). ^{1}H NMR (400 MHz, $CDCl_3$) δ 8.00 – 7.93 (m, 2H), 7.73 (dt, *J* = 7.9, 1.2 Hz, 2H), 7.64 – 7.57 (m, 1H), 7.49 (ddd, *J* = 8.0, 6.8, 1.2 Hz, 2H), 7.35 – 7.28 (m, 2H), 5.16 (tq, *J* = 8.0, 1.4 Hz, 1H), 3.89 (s, 3H), 3.72 (d, *J* = 7.9 Hz, 2H), 3.67 – 3.61 (m, 1H), 2.87 – 2.69 (m, 1H), 2.47 – 2.27 (m, 1H), 1.34 (s, 9H), 1.33 (d, *J* = 1.4 Hz, 3H). ^{13}C NMR (101 MHz, $CDCl_3$) δ 171.7, 166.8, 144.0, 143.0, 138.7, 133.6, 129.9, 129.2, 129.1, 128.4, 127.9, 112.8, 81.4, 55.9, 52.1, 50.7, 42.6, 27.9, 16.4. IR (film) ν (cm^{-1}) 2918, 1720, 1609, 1446, 1368, 1308, 1281, 1149, 1112, 1085, 1020, 846, 742, 689, 559, 528, 500. HR-MS (ESI) m/z calcd for $C_{25}H_{30}NaO_6S^+$ 481.16553, found 481.16711, $[M+Na^+]$. The enantiomeric ratio of **25** was determined by HPLC analysis on Chiralpak AD-H column. Conditions: hexane/isopropanol = 80/20, flow rate = 1.0 mL/min, uv-vis detection at λ = 254 nm, t_R = 71.0 min (minor), 77.5 min (major).

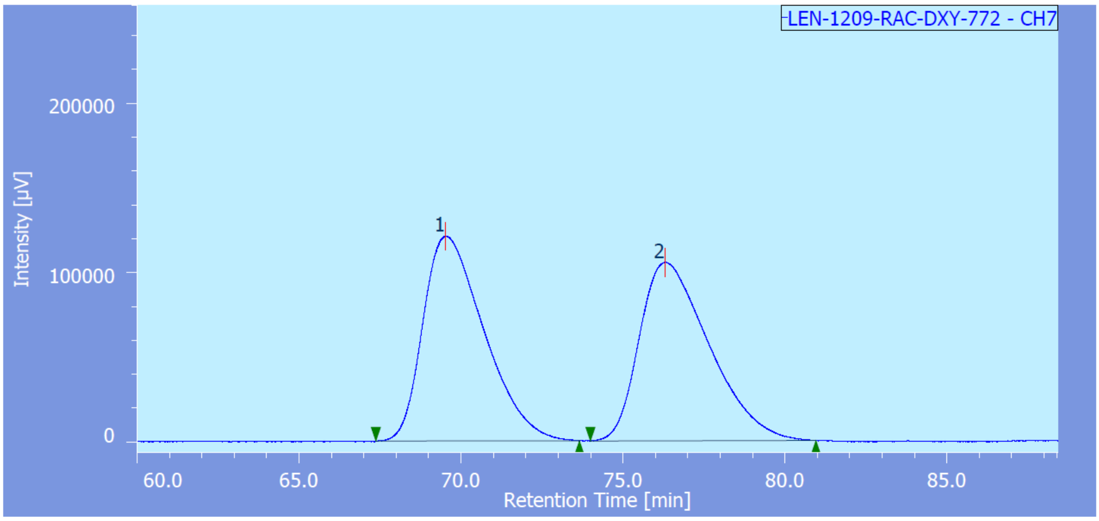

Decision

#	Peak Name	CH	tR [min]	Area [µV·sec]	Height [µV]	Area%	Height%	Quantity	NTP	Resolution	Symmetry Factor	Warning
1	Unknown	7	69.527	15858239	121032	50.122	53.484	N/A	6429	1.825	1.481	
2	Unknown	7	76.300	15781339	105264	49.878	46.516	N/A	5899	N/A	1.508	

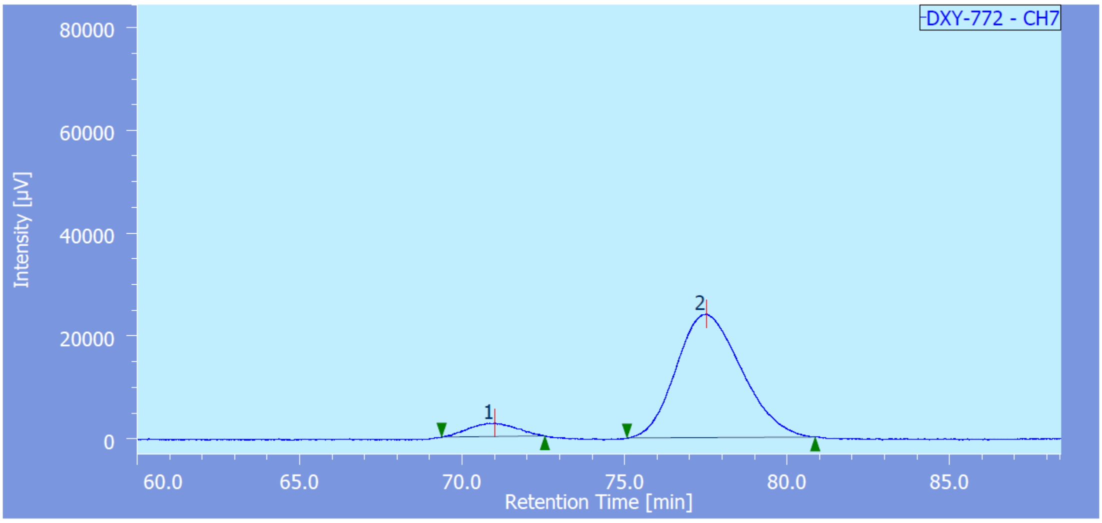

Decision

#	Peak Name	CH	tR [min]	Area [µV·sec]	Height [µV]	Area%	Height%	Quantity	NTP	Resolution	Symmetry Factor	Warning
1	Unknown	7	71.000	268928	2603	7.429	9.787	N/A	9195	1.952	0.987	
2	Unknown	7	77.507	3351240	23996	92.571	90.213	N/A	6935	N/A	1.179	

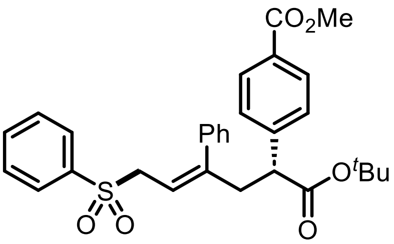

Methyl (*R*,*E*)-4-(1-(*tert*-butoxy)-4-methyl-1-oxo-6-(phenylsulfonyl)hex-4-en-2-yl)benzoate (26). Colorless oil (Hex/EA = 2/1), 63%, 90:10 er, $[\alpha]_D^{24}$ = -28.6 (c = 0.20, $CHCl_3$). ^{1}H NMR (500 MHz, $CDCl_3$) δ 8.00 (d, *J* = 8.4 Hz, 2H), 7.67 – 7.60 (m, 3H), 7.52 – 7.46 (m,

2H), 7.25 (dd, J = 7.7, 3.4 Hz, 5H), 6.71 – 6.64 (m, 2H), 5.51 (t, J = 7.8 Hz, 1H), 3.94 (s, 3H), 3.66 (dd, J = 7.8, 2.4 Hz, 2H), 3.45 (t, J = 7.6 Hz, 1H), 3.13 (ddd, J = 14.6, 7.5, 1.1 Hz, 1H), 2.75 (dd, J = 14.9, 7.5 Hz, 1H), 1.35 (s, 9H). ^{13}C NMR (126 MHz, $CDCl_3$) δ 171.6, 167.0, 147.5, 143.9, 138.6, 137.6, 133.6, 132.0, 130.0, 129.1, 128.6, 128.5, 128.2, 128.0, 127.9, 115.5, 81.5, 56.7, 52.3, 50.5, 42.5, 28.0. IR (film) ν (cm^{-1}) 2976, 2932, 1720, 1610, 1445, 1368, 1309, 1280, 1142, 1112, 1086, 1020, 953, 844, 771, 743, 703, 689, 565, 528. HR-MS (ESI) m/z calcd for $C_{30}H_{32}NaO_6S^+$ 543.18118, found 543.18195, [M+Na$^+$]. The enantiomeric ratio of **26** was determined by HPLC analysis on Chiralpak NR column. Conditions: hexane/isopropanol = 50/50, flow rate = 1.0 mL/min, uv-vis detection at λ = 220 nm, t_R = 35.7 min (minor), 39.9 min (major).

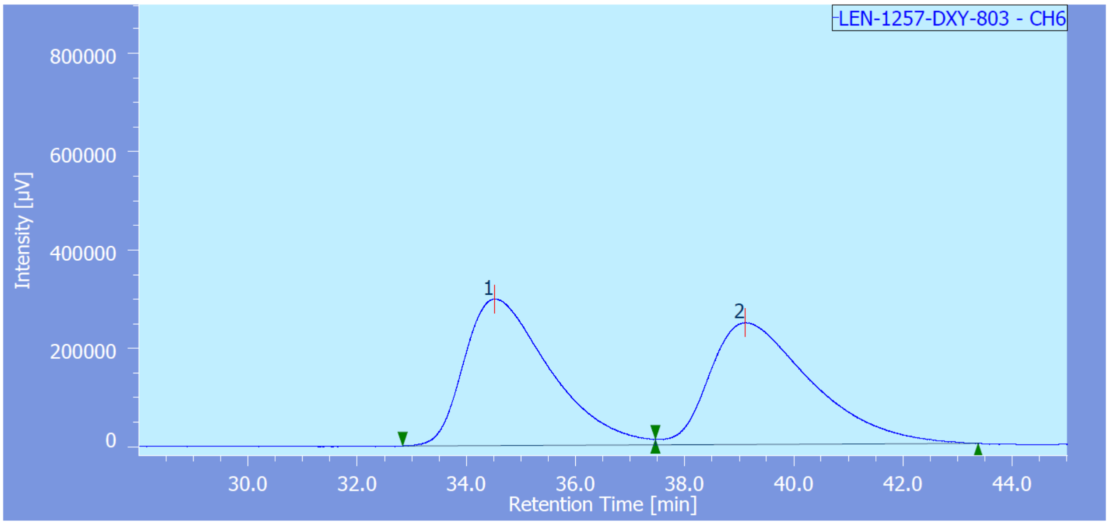

#	Peak Name	CH	tR [min]	Area [µV·sec]	Height [µV]	Area%	Height%	Quantity	NTP	Resolution	Symmetry Factor	Warning
1	Unknown	6	34.517	32449546	297773	50.284	54.640	N/A	2373	1.492	1.680	
2	Unknown	6	39.113	32083525	247198	49.716	45.360	N/A	2191	N/A	1.587	

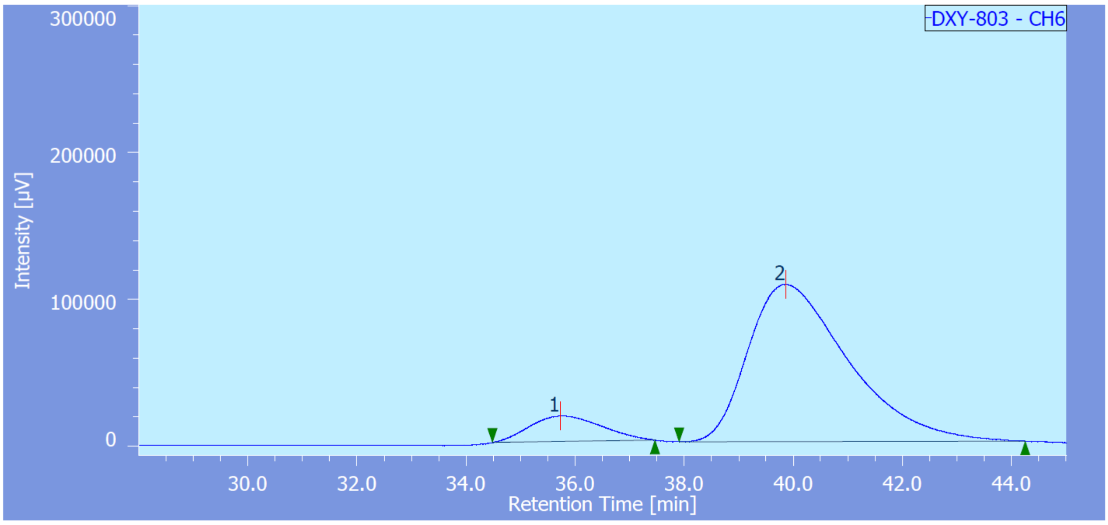

#	Peak Name	CH	tR [min]	Area [µV·sec]	Height [µV]	Area%	Height%	Quantity	NTP	Resolution	Symmetry Factor	Warning
1	Unknown	6	35.730	1606986	17422	10.499	13.998	N/A	3097	1.410	1.197	
2	Unknown	6	39.863	13698907	107036	89.501	86.002	N/A	2320	N/A	1.583	

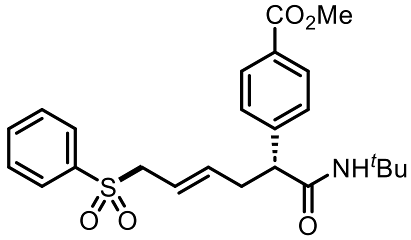

Methyl (*R,E*)-4-(1-(*tert*-butylamino)-1-oxo-6-(phenylsulfonyl)hex-4-en-2-yl)benzoate (27). Colorless oil (Hex/EA = 1/2), 70%, 76:24 er, $[\alpha]_D^{23}$ = -4.8 (c = 0.21, $CHCl_3$). ^{1}H NMR (400 MHz, $CDCl_3$) δ 7.96 (dd, *J* = 8.5, 2.0 Hz, 2H), 7.76 (dd, *J* = 8.4, 1.3 Hz, 2H), 7.66 – 7.60 (m, 1H), 7.51 (ddd, *J* = 8.0, 6.6, 1.3 Hz, 2H), 7.31 – 7.26 (m, 2H), 5.54 – 5.36 (m, 2H), 5.24 (br s, 1H), 3.90 (s, 3H), 3.66 (d, *J* = 6.6 Hz, 2H), 3.25 (t, *J* = 7.5 Hz, 1H), 2.81 (dt, *J* = 13.6, 6.8 Hz, 1H), 2.41 (dt, *J* = 14.3, 7.0 Hz, 1H), 1.23 (d, *J* = 6.3 Hz, 9H). ^{13}C NMR (101 MHz, $CDCl_3$) δ 170.9, 166.9, 144.8, 138.6, 138.5, 133.8, 130.2, 129.3, 129.2, 128.4, 127.9, 118.3, 60.0, 53.5, 52.3, 51.6, 36.5, 25.0. IR (film) ν (cm^{-1}) 3377, 2967, 1719, 1673, 1609, 1530, 1447, 1436, 1392, 1364, 1307, 1280, 1225, 1181, 1150, 1137, 1112, 1086, 1020, 967, 863, 767, 732, 703, 689, 634, 598, 553, 528. HR-MS (ESI) m/z calcd for $C_{24}H_{30}NO_5S^+$ 444.18392, found 444.18301, [M+H$^+$]. The enantiomeric ratio of **27** was determined by HPLC analysis on Chiralpak IC column. Conditions: hexane/isopropanol = 50/50, flow rate = 0.5 mL/min, uv-vis detection at λ = 210 nm, t_R = 34.2 min (major), 37.5 min (minor).(27)

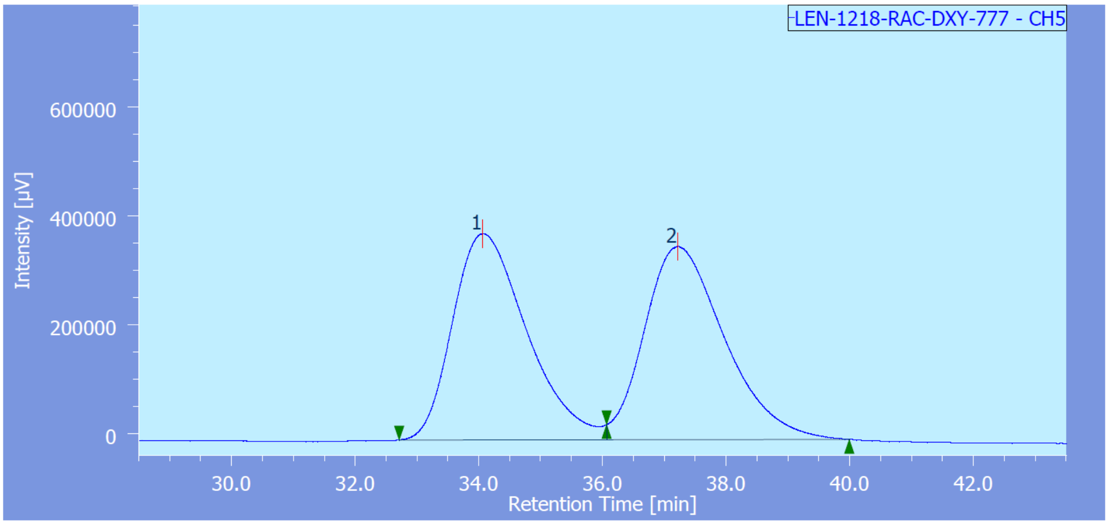

#	Peak Name	CH	tR [min]	Area [µV·sec]	Height [µV]	Area%	Height%	Quantity	NTP	Resolution	Symmetry Factor	Warning
1	Unknown	5	34.063	31675076	379171	49.767	51.673	N/A	3893	1.395	N/A	
2	Unknown	5	37.217	31971470	354618	50.233	48.327	N/A	4011	N/A	N/A	

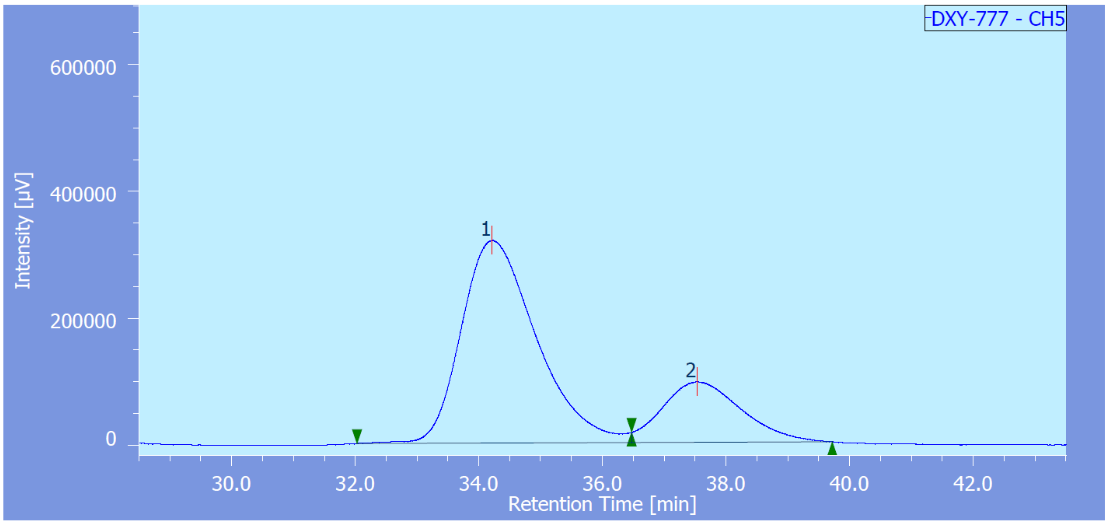

#	Peak Name	CH	tR [min]	Area [µV·sec]	Height [µV]	Area%	Height%	Quantity	NTP	Resolution	Symmetry Factor	Warning
1	Unknown	5	34.217	27056089	319216	76.145	77.019	N/A	3920	1.458	N/A	
2	Unknown	5	37.530	8476265	95250	23.855	22.981	N/A	4013	N/A	N/A	

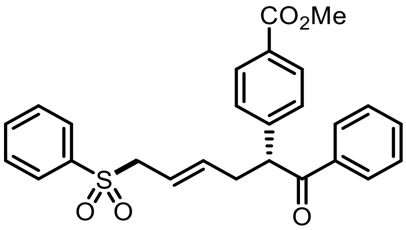

Methyl (*R*,*E*)-4-(1-oxo-1-phenyl-6-(phenylsulfonyl)hex-4-en-2-yl)benzoate (28). Colorless oil (Hex/EA = 3/1), 35%, 90:10 er, $[\alpha]_D^{23}$ = -33.4 (c = 0.18, $CHCl_3$). ^1H NMR (500 MHz, $CDCl_3$) δ 7.95 (d, *J* = 8.3 Hz, 2H), 7.87 (dd, *J* = 8.3, 1.4 Hz, 2H), 7.76 (dd, *J* = 8.3, 1.4 Hz, 2H), 7.56 (t, *J* = 7.5 Hz, 1H), 7.51 (t, *J* = 7.4 Hz, 1H), 7.46 (t, *J* = 7.8 Hz, 2H), 7.39 (t, *J* = 7.7 Hz, 2H), 7.28 (d, *J* = 8.4 Hz, 2H), 5.53 – 5.42 (m, 2H), 4.54 (t, *J* = 7.3 Hz, 1H), 3.88 (s, 3H), 3.68 (d, *J* = 6.4 Hz, 2H), 2.90 (dt, *J* = 14.3, 7.1 Hz, 1H), 2.51 (dt, *J* = 13.4, 6.7 Hz, 1H). ^{13}C NMR (126 MHz, $CDCl_3$) δ 198.1, 166.8, 143.8, 138.5, 138.2, 136.2, 133.8, 133.5, 130.5, 129.4, 129.1, 128.9, 128.8, 128.5, 128.3, 118.9, 60.0, 53.5, 52.3, 36.8. IR (film) ν (cm^{-1}) 2921, 2850, 1719, 1682, 1608, 1446, 1436, 1417, 1307, 1281, 1181, 1151, 1138, 1113, 1086, 1019, 968, 767, 735, 704, 689, 598, 555, 530, 505. HR-MS (ESI) m/z calcd for $C_{26}H_{24}NaO_5S^+$ 471.12367, found 471.12476, [$M+Na^+$]. The enantiomeric ratio of **28** was determined by HPLC analysis on Chiralpak AD-H column. Conditions: hexane/isopropanol = 50/50, flow rate = 1.0 mL/min,

uv-vis detection at λ = 210 nm, t_R = 44.9 min (major), 49.2 min (minor).

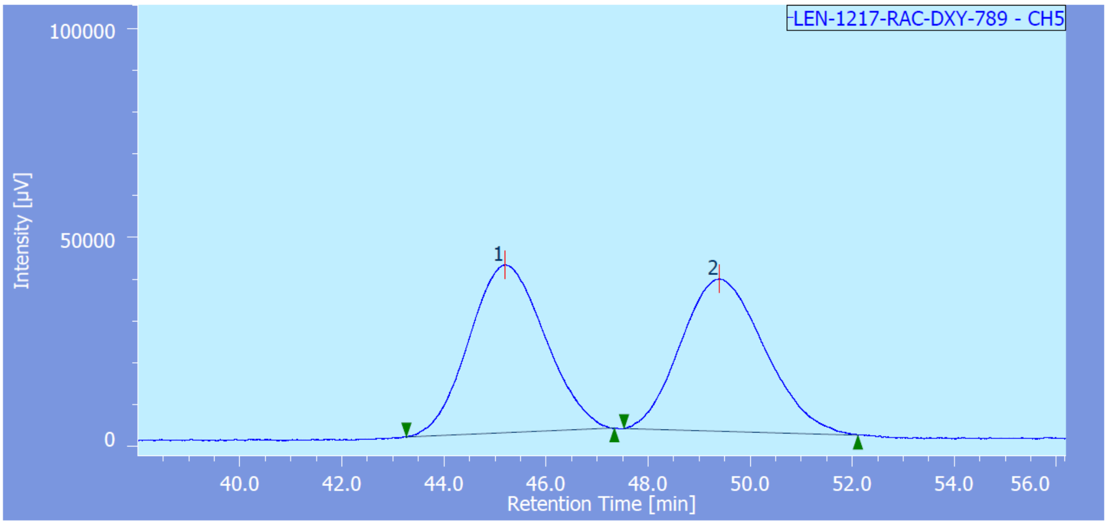

Decision

#	Peak Name	CH	tR [min]	Area [µV·sec]	Height [µV]	Area%	Height%	Quantity	NTP	Resolution	Symmetry Factor	Warning
1	Unknown	5	45.187	4150821	40150	49.933	52.406	N/A	4191	1.440	1.085	
2	Unknown	5	49.393	4161931	36463	50.067	47.594	N/A	4149	N/A	1.171	

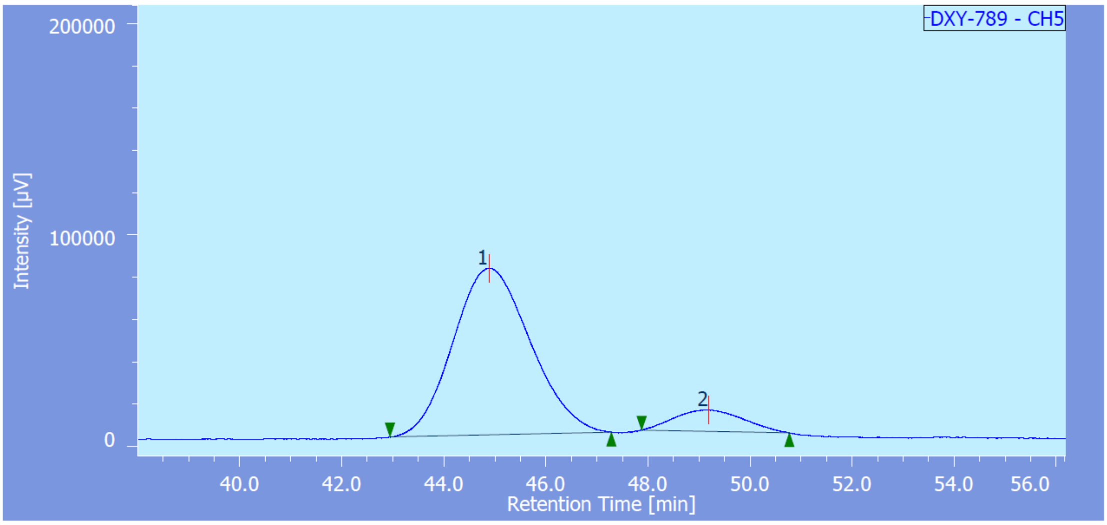

Decision

#	Peak Name	CH	tR [min]	Area [µV·sec]	Height [µV]	Area%	Height%	Quantity	NTP	Resolution	Symmetry Factor	Warning
1	Unknown	5	44.883	8268447	78583	89.623	88.548	N/A	4075	1.573	1.126	
2	Unknown	5	49.190	957395	10163	10.377	11.452	N/A	5400	N/A	1.085	

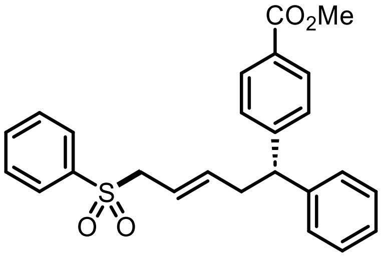

Methyl (*S*,*E*)-4-(1-phenyl-5-(phenylsulfonyl)pent-3-en-1-yl)benzoate (29). Colorless oil (Hex/EA = 3/1), 38%, 98:2 er, $[\alpha]_D^{22}$ = -11.2 (c = 0.080, $CHCl_3$). ^1H NMR (500 MHz, $CDCl_3$)

δ 7.97 – 7.89 (m, 2H), 7.77 – 7.69 (m, 2H), 7.67 – 7.58 (m, 1H), 7.54 – 7.46 (m, 2H), 7.32 – 7.23 (m, 2H), 7.24 – 7.15 (m, 3H), 7.16 – 7.08 (m, 2H), 5.45 (m, 2H), 3.93 (t, J = 7.8 Hz, 2H), 3.89 (s, 3H), 3.73 – 3.63 (m, 2H), 2.83 – 2.73 (m, 2H).. ^{13}C NMR (126 MHz, $CDCl_3$) δ 167.05, 149.33, 143.16, 138.87, 138.57, 133.75, 130.02, 129.15, 128.81, 128.53, 128.00, 127.90, 126.84, 118.39, 60.08, 52.21, 50.91, 38.47. IR (film) ν (cm^{-1}) 3058, 3027, 2951, 2922, 1717 (s, C=O), 1610, 1494, 1446, 1435, 1308, 1281, 1181, 1150, 1137, 1108, 1086, 1019, 973, 760, 733, 709, 689, 596, 560, 531. HR-MS (ESI) m/z calcd for $C_{25}H_{24}NaO_4S^+$ 443.12875, found 443.12946, [M+Na$^+$]. The enantiomeric ratio of **29** was determined by HPLC analysis on Chiralpak AD-H column. Conditions: hexane/isopropanol = 60/40, flow rate = 1.0 mL/min, uv-vis detection at λ = 254 nm, t_R = 15.2 min (minor), 16.7 min (major).

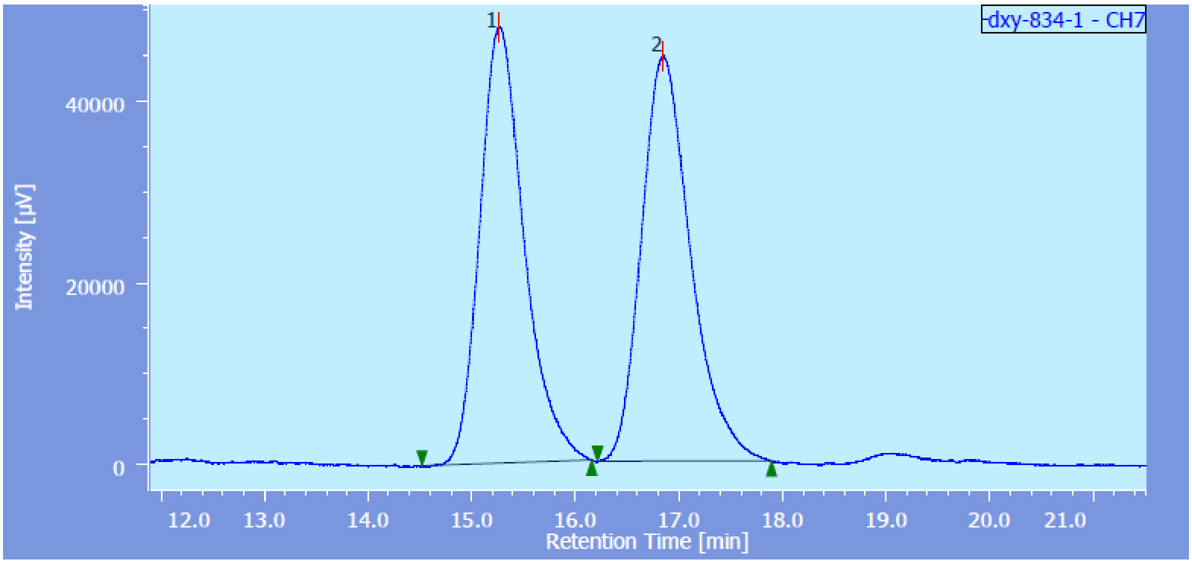

#	Peak Name	CH	tR [min]	Area [μV·sec]	Height [μV]	Area%	Height%	Quantity	NTP	Resolution	Symmetry Factor	Warning
1	Unknown	7	15.260	1424278	47881	49.164	51.853	N/A	6502	1.984	1.314	
2	Unknown	7	16.847	1472714	44459	50.836	48.147	N/A	6336	N/A	1.300	

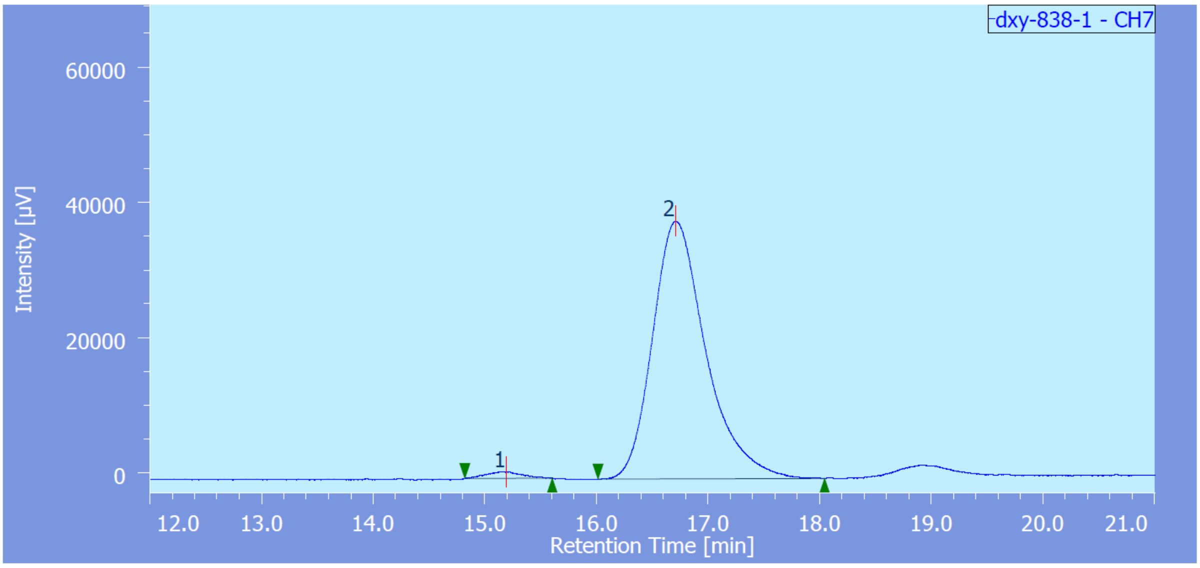

#	Peak Name	CH	tR [min]	Area [μV·sec]	Height [μV]	Area%	Height%	Quantity	NTP	Resolution	Symmetry Factor	Warning
1	Unknown	7	15.193	21894	944	1.699	2.420	N/A	9351	2.078	1.029	
2	Unknown	7	16.710	1266704	38063	98.301	97.580	N/A	6406	N/A	1.346	

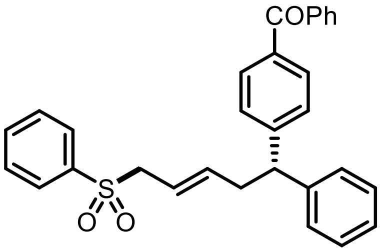

(*S*,*E*)-Phenyl(4-(1-phenyl-5-(phenylsulfonyl)pent-3-en-1-yl)phenyl)methanone (30). Colorless oil (Hex/EA = 3/1), 45%, 98:2 er, $[\alpha]_D^{22}$ = -27.8 (c = 0.080, $CHCl_3$). ^1H NMR (400 MHz, $CDCl_3$) δ 7.75 – 7.63 (m, 6H), 7.60 – 7.55 (m, 1H), 7.54 – 7.49 (m, 1H), 7.48 – 7.37 (m, 4H), 7.28 – 7.09 (m, 7H), 5.48 – 5.35 (m, 2H), 3.92 (t, J = 7.8 Hz, 1H), 3.68 – 3.57 (m, 2H), 2.83 – 2.70 (m, 2H). ^{13}C NMR (126 MHz, $CDCl_3$) δ 196.42, 148.93, 143.15, 138.88, 138.62, 137.76, 135.91, 133.76, 132.50, 130.64, 130.12, 129.18, 128.84, 128.51, 128.40, 127.94, 127.89, 126.88, 118.40, 60.09, 50.97, 38.50. IR (film) ν (cm^{-1}) 2924, 1756 (s, C=O), 1618, 1599, 1584, 1495, 1446, 1401, 1363, 1305, 1235, 1149,1085, 1046, 1002, 973, 911, 763, 730, 705, 689, 648, 591, 558, 528. HR-MS (ESI) m/z calcd for $C_{30}H_{27}O_3S^+$ 467.16754, found 467.16840, $[M+H^+]$. The enantiomeric ratio of **30** was determined by HPLC analysis on Chiralpak AD-H column. Conditions: hexane/isopropanol = 60/40, flow rate = 1.0 mL/min, uv-vis detection at λ = 254 nm, t_R = 17.9 min (minor), 21.6 min (major).

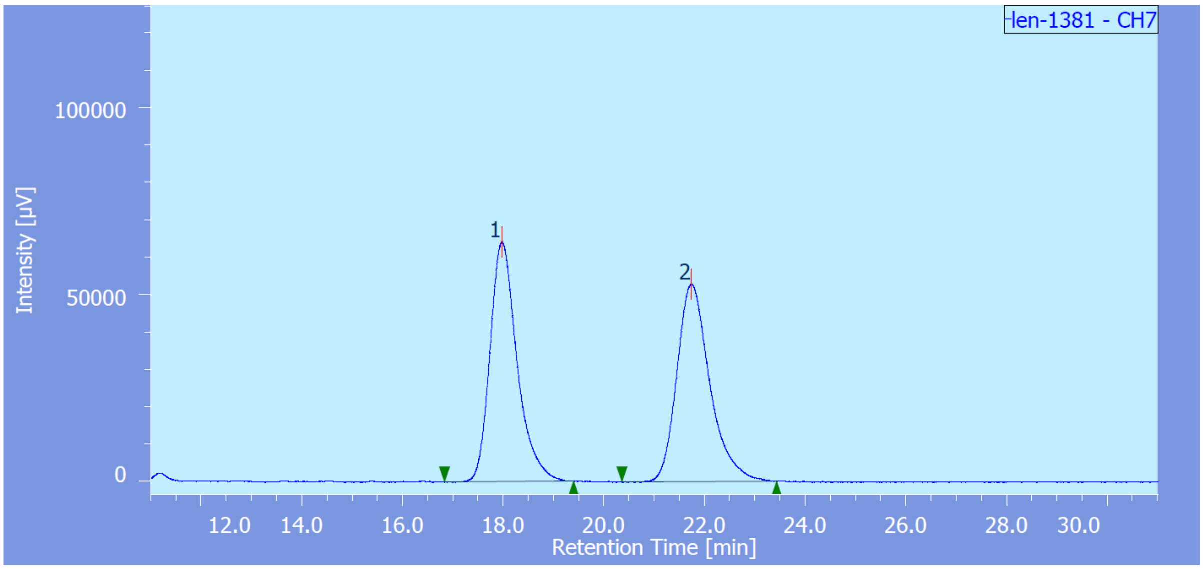

#	Peak Name	CH	tR [min]	Area [µV·sec]	Height [µV]	Area%	Height%	Quantity	NTP	Resolution	Symmetry Factor	Warning
1	Unknown	7	17.973	2389710	63981	49.890	54.795	N/A	5849	3.625	1.324	
2	Unknown	7	21.743	2400280	52784	50.110	45.205	N/A	5764	N/A	1.304	

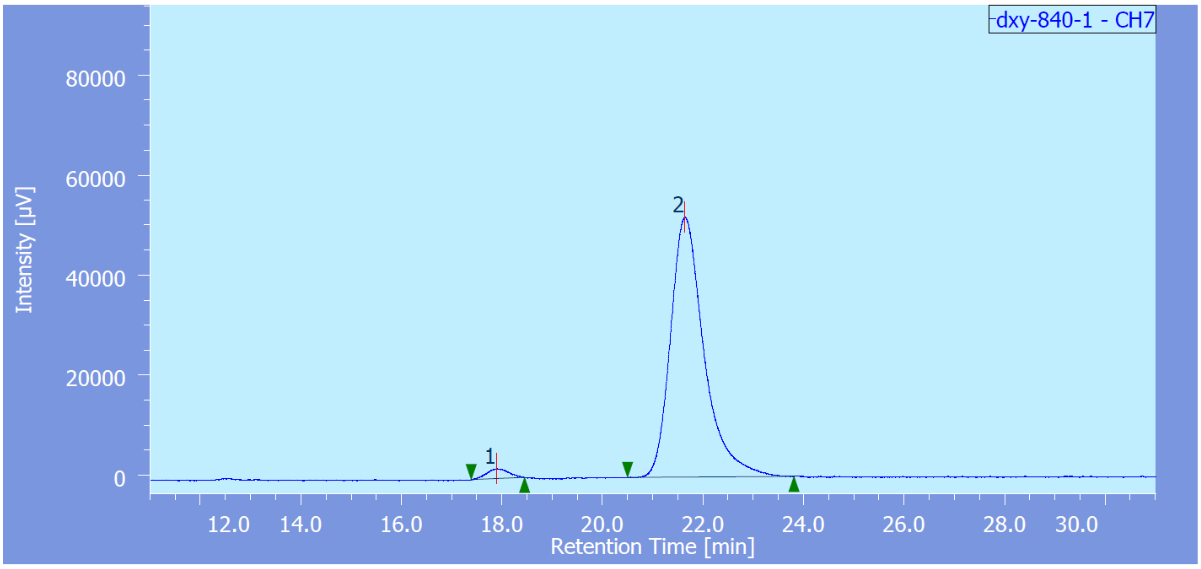

#	Peak Name	CH	tR [min]	Area [µV·sec]	Height [µV]	Area%	Height%	Quantity	NTP	Resolution	Symmetry Factor	Warning
1	Unknown	7	17.900	61041	1992	2.471	3.692	N/A	7121	3.767	1.046	
2	Unknown	7	21.640	2409439	51961	97.529	96.308	N/A	5738	N/A	1.410	

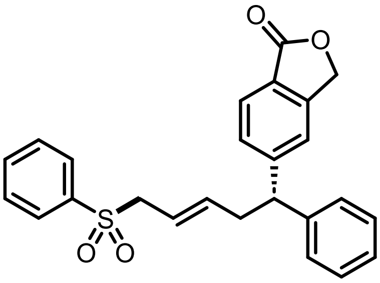

(*S*,*E*)-5-(1-phenyl-5-(phenylsulfonyl)pent-3-en-1-yl)isobenzofuran-1(3*H*)-one (31).

Colorless oil (Hex/EA = 3/1), 38%, 98:2 er, $[\alpha]_D^{22}$ = -18.4 (c = 0.10, $CHCl_3$). ^1H NMR (500 MHz, $CDCl_3$) δ7.74 (d, J = 7.9 Hz, 1H), 7.68 – 7.63 (m, 2H), 7.59 – 7.54 (m, 1H), 7.46 – 7.40 (m, 2H), 7.27 – 7.20 (m, 4H), 7.16 (td, J = 5.4, 4.9, 2.6 Hz, 1H), 7.11 – 7.06 (m, 2H), 5.50 – 5.34 (m, 2H), 5.20 (s, 2H), 3.99 (t, J = 7.8 Hz, 1H), 3.60 (d, J = 6.7 Hz, 2H), 2.67 – 2.86 (m, 2H). ^{13}C NMR (126 MHz, $CDCl_3$) δ 170.99, 151.23, 147.43, 142.59, 138.80, 138.57, 133.81, 129.21, 129.00, 128.40, 127.92, 127.14, 125.98, 124.26, 121.67, 118.60, 69.70, 59.99, 51.17, 38.57. IR (film) ν (cm^{-1}) 2970, 2922, 1737 (s, C=O), 1654, 1599, 1494, 1446, 1411, 1308, 1280, 1232, 1149, 1136, 1085, 972, 939, 923, 732, 702, 689, 646, 620, 597, 557, 529, 509. HR-MS (ESI) m/z calcd for $C_{25}H_{23}O_4S^+$ 419.13116, found 419.13147, [$M+H^+$]. The enantiomeric ratio of **31** was determined by HPLC analysis on Chiralpak AD-H column. Conditions: hexane/isopropanol = 60/40, flow rate = 1.0 mL/min, uv-vis detection at λ = 254 nm, t_R = 13.3 min (major), 15.0 min (minor).

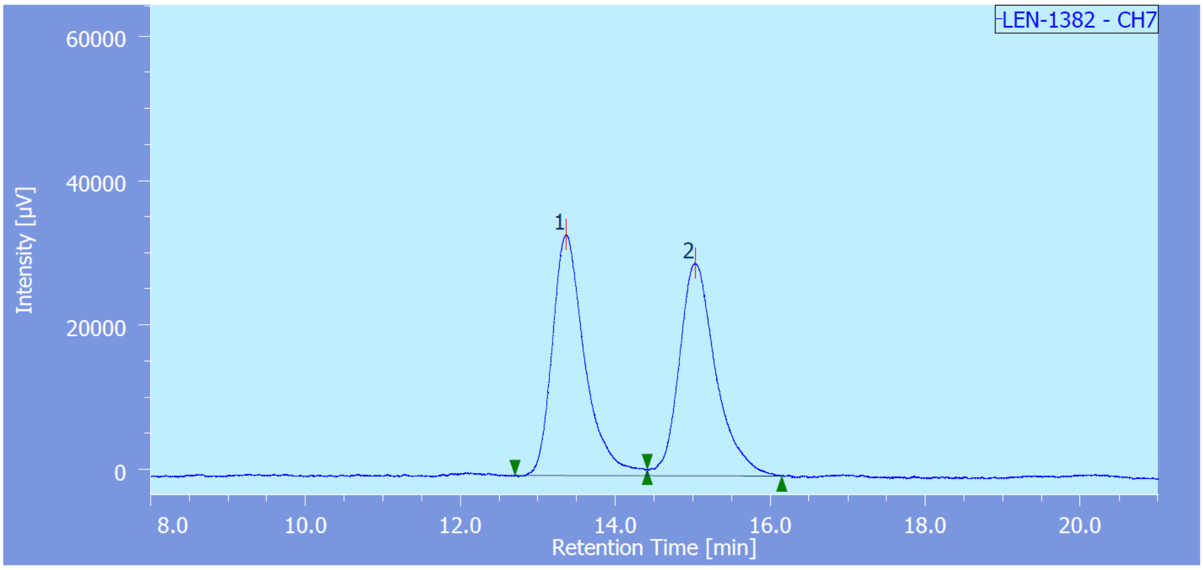

#	Peak Name	CH	tR [min]	Area [µV·sec]	Height [µV]	Area%	Height%	Quantity	NTP	Resolution	Symmetry Factor	Warning
1	Unknown	7	13.363	973994	33353	50.241	53.117	N/A	5592	2.195	1.421	
2	Unknown	7	15.030	964650	29439	49.759	46.883	N/A	5534	N/A	1.272	

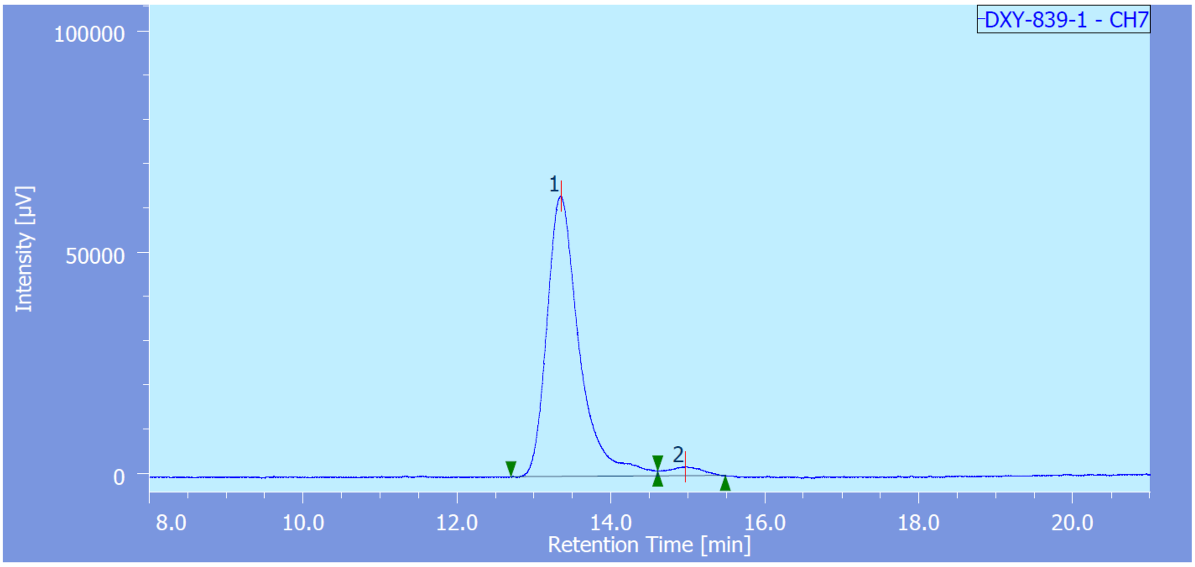

#	Peak Name	CH	tR [min]	Area [μV·sec]	Height [μV]	Area%	Height%	Quantity	NTP	Resolution	Symmetry Factor	Warning
1	Unknown	7	13.347	1850640	63280	96.701	96.910	N/A	5718	N/A	1.446	
2	Unknown	7	14.960	63130	2018	3.299	3.090	N/A	N/A	N/A	N/A	

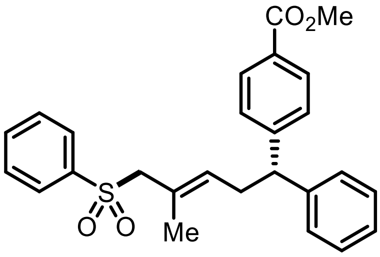

Methyl (*S*,*E*)-4-(4-methyl-1-phenyl-5-(phenylsulfonyl)pent-3-en-1-yl)benzoate (32). Colorless oil (Hex/EA = 3/1), 58%, 99:1 er, $[\alpha]_D^{23}$ = -6.98 (c = 0.13, $CHCl_3$). ^{1}H NMR (500 MHz, $CDCl_3$) δ 7.95 – 7.89 (m, 2H), 7.79 – 7.71 (m, 2H), 7.64 – 7.58 (m, 1H), 7.51 – 7.44 (m, 2H), 7.30 – 7.23 (m, 2H), 7.22 – 7.16 (m, 3H), 7.14 – 7.09 (m, 2H), 5.11 – 5.00 (m, 1H), 3.89 (s, 3H), 3.87 (t, *J* = 7.9 Hz, 1H), 3.64 (d, *J* = 0.9 Hz, 2H), 2.72 (t, *J* = 7.4 Hz, 2H), 1.72 (d, *J* = 1.4 Hz, 3H).. ^{13}C NMR (126 MHz, $CDCl_3$) δ 167.07, 149.59, 143.44, 138.73, 133.74, 133.66, 129.96, 129.07, 128.76, 128.48, 128.43, 128.03, 127.91, 126.77, 125.14, 66.17, 52.20, 50.76, 34.43, 17.22. IR (film) ν (cm^{-1}) 2970, 2917, 1718 (s, C=O), 1609, 1494, 1446, 1435, 1415, 1367, 1307, 1280, 1180, 1148, 1134, 1107, 1085, 1019, 860, 760, 742, 709, 689, 619, 564, 529. HR-MS (ESI) m/z calcd for $C_{26}H_{26}NaO_4S^+$ 457.14440, found 457.14481, [M+Na$^+$]. The enantiomeric ratio of **32** was determined by HPLC analysis on Chiralpak AD-H column. Conditions: hexane/isopropanol = 60/40, flow rate = 1.0 mL/min, uv-vis detection at λ = 254 nm, t_R = 11.7 min (minor), 13.1 min (major).

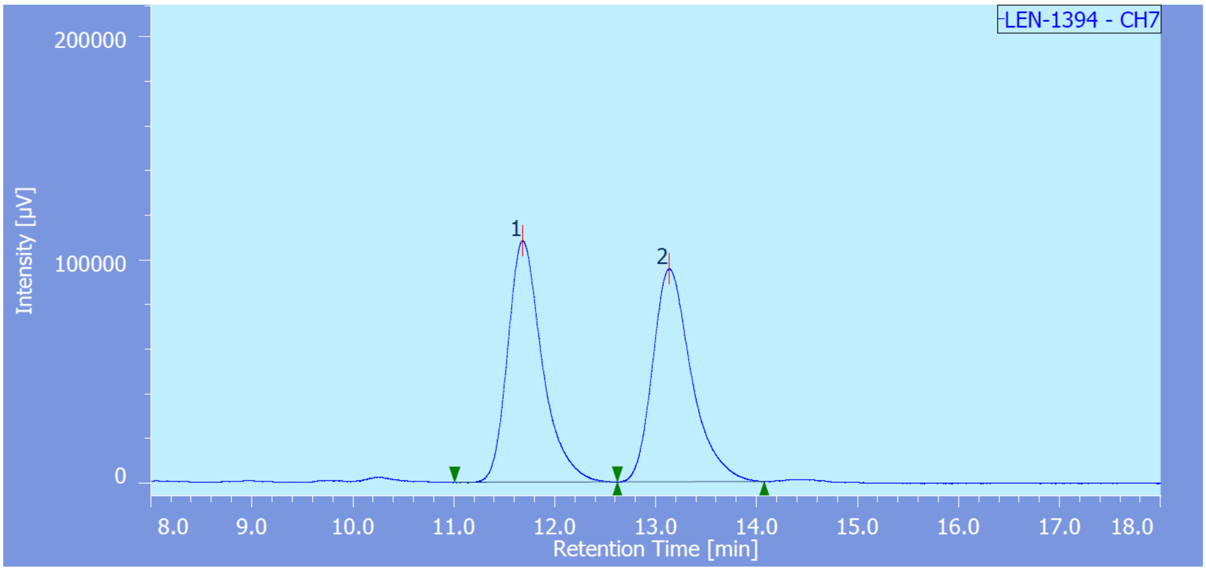

#	Peak Name	CH	tR [min]	Area [µV·sec]	Height [µV]	Area%	Height%	Quantity	NTP	Resolution	Symmetry Factor	Warning
1	Unknown	7	11.683	2571884	108171	50.217	53.129	N/A	6133	2.286	1.337	
2	Unknown	7	13.133	2549684	95431	49.783	46.871	N/A	6056	N/A	1.358	

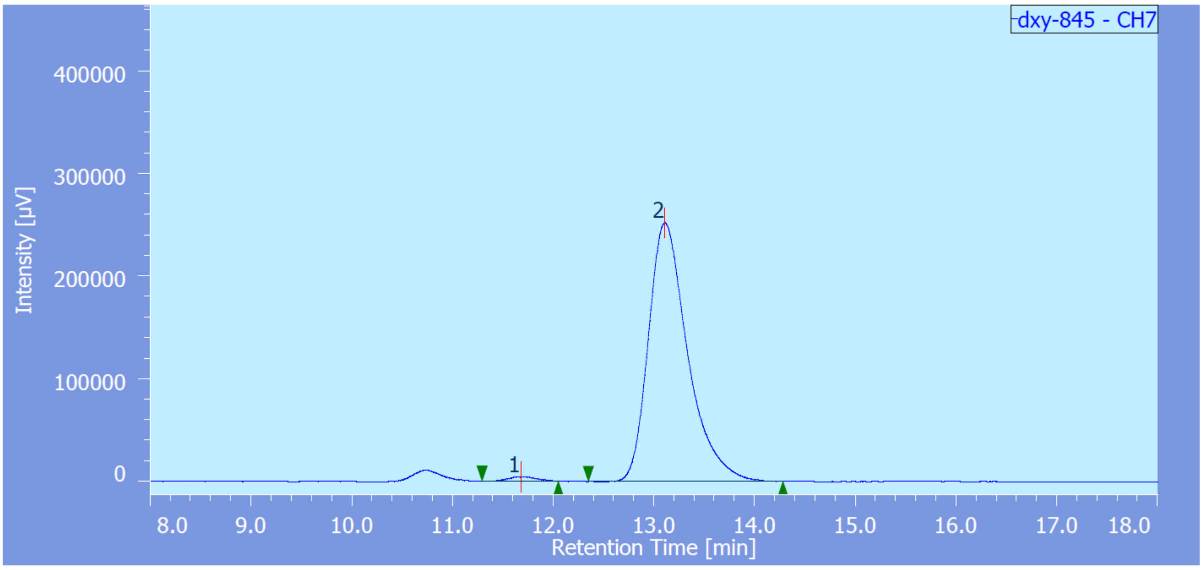

#	Peak Name	CH	tR [min]	Area [µV·sec]	Height [µV]	Area%	Height%	Quantity	NTP	Resolution	Symmetry Factor	Warning
1	Unknown	7	11.680	80718	4115	1.167	1.607	N/A	7807	2.369	1.136	
2	Unknown	7	13.110	6835647	251959	98.833	98.393	N/A	5920	N/A	1.403	

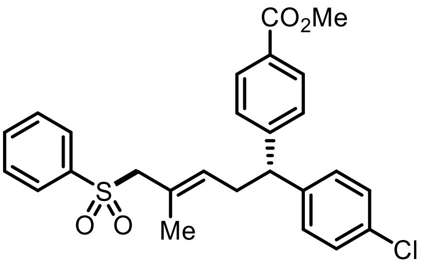

Methyl (*R*,*E*)-4-(1-(4-chlorophenyl)-4-methyl-5-(phenylsulfonyl)pent-3-en-1-yl)benzoate

(33). Colorless oil (Hex/EA = 3/1), 68%, 99:1 er, $[\alpha]_D^{23}$ = -8.45 (c = 0.15, $CHCl_3$). ^1H NMR (500 MHz, $CDCl_3$) δ 7.96 – 7.89 (m, 2H), 7.78 – 7.73 (m, 2H), 7.65 – 7.58 (m, 1H), 7.51 – 7.45 (m, 2H), 7.25 – 7.21 (m, 2H), 7.20 – 7.14 (m, 2H), 7.07 – 7.02 (m, 2H), 5.05 (td, J = 7.0, 1.5 Hz, 1H), 3.89 (s, 3H), 3.86 (t, J = 7.7 Hz, 1H), 3.70 – 3.60 (m, 2H), 2.69 (t, J = 7.4 Hz, 2H), 1.71 (s, 3H). ^{13}C NMR (126 MHz, $CDCl_3$) δ 166.97, 149.02, 141.90, 138.80, 133.69, 133.29, 132.57, 130.06, 129.30, 129.10, 128.89, 128.66, 128.43, 127.93, 125.45, 66.08, 52.24, 50.12, 34.35, 17.28. IR (film) ν (cm^{-1}) 2970, 2921, 1718 (s, C=O), 1609, 1491, 1446, 1436, 1408, 1368, 1307, 1281, 1230, 1217, 1181, 1149, 1134, 1109, 1087, 1014, 828, 760, 740, 726, 708, 689, 614, 565, 527. HR-MS (ESI) m/z calcd for $C_{26}H_{25}ClNaO_4S^+$ 491.10543, found 491.10602, [M+Na^+]. The enantiomeric ratio of **33** was determined by HPLC analysis on Chiralpak AD-H column. Conditions: hexane/isopropanol = 60/40, flow rate = 1.0 mL/min, uv-vis detection at λ = 254 nm, t_R = 14.5 min (major), 24.0 min (minor).

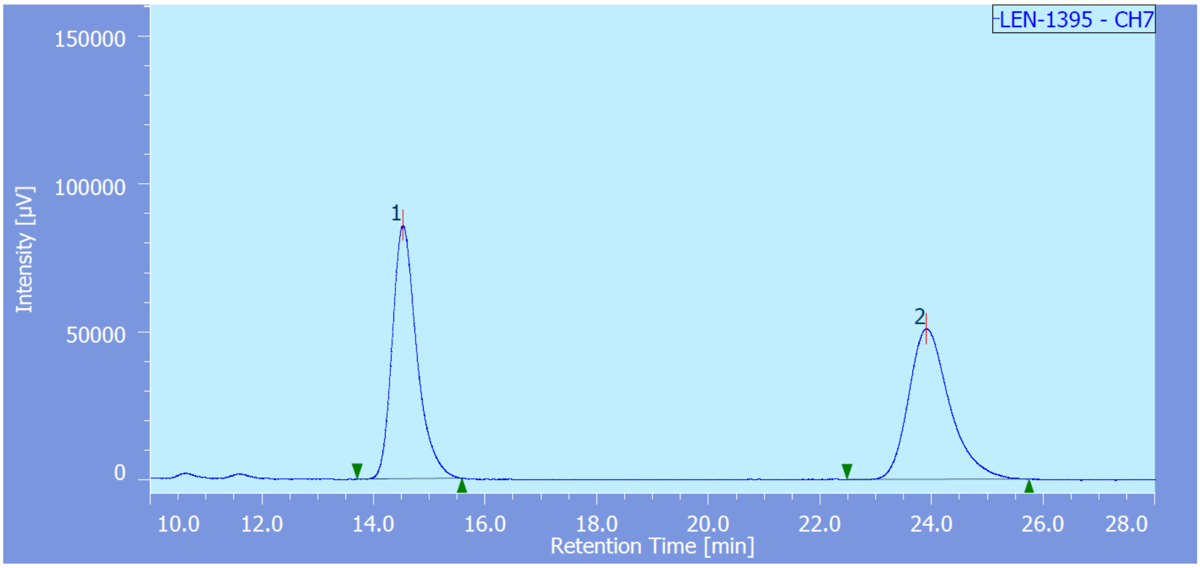

#	Peak Name	CH	tR [min]	Area [µV·sec]	Height [µV]	Area%	Height%	Quantity	NTP	Resolution	Symmetry Factor	Warning
1	Unknown	7	14.527	2601001	85578	50.172	62.755	N/A	5737	9.149	1.340	
2	Unknown	7	23.907	2583211	50790	49.828	37.245	N/A	5505	N/A	1.315	

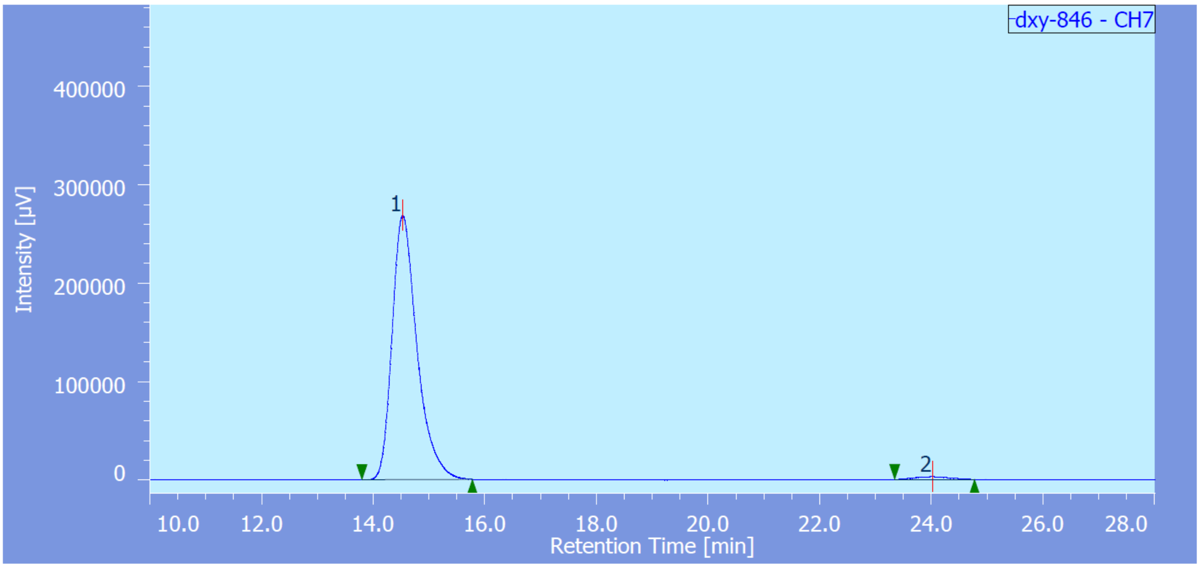

#	Peak Name	CH	tR [min]	Area [μV·sec]	Height [μV]	Area%	Height%	Quantity	NTP	Resolution	Symmetry Factor	Warning
1	Unknown	7	14.530	8319862	268327	98.578	98.973	N/A	5540	9.668	1.383	
2	Unknown	7	24.020	119998	2783	1.422	1.027	N/A	6545	N/A	1.035	

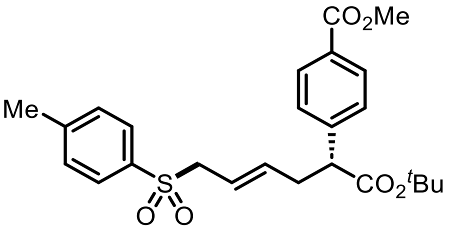

Methyl (*R*,*E*)-4-(1-(*tert*-butoxy)-1-oxo-6-tosylhex-4-en-2-yl)benzoate (34). Colorless oil (Hex/EA = 2/1), 48%, 91:9 er, $[\alpha]_D^{24}$ = -21.6 (c = 0.22, $CHCl_3$). ^{1}H NMR (400 MHz, $CDCl_3$) δ 7.99 – 7.94 (m, 2H), 7.68 – 7.63 (m, 2H), 7.33 – 7.29 (m, 2H), 7.28 – 7.25 (m, 2H), 5.49 – 5.38 (m, 2H), 3.91 (s, 3H), 3.68 – 3.63 (m, 2H), 3.47 (t, *J* = 7.6 Hz, 1H), 2.80 – 2.70 (m, 1H), 2.45 (s, 4H), 1.35 (s, 9H). ^{13}C NMR (101 MHz, $CDCl_3$) δ 171.6, 167.0, 144.8, 143.9, 137.5, 135.7, 130.0, 129.8, 129.3, 128.6, 128.0, 119.0, 81.6, 60.1, 52.3, 52.2, 36.1, 28.0, 21.8. IR (film) ν (cm^{-1}) 2974, 2928, 1719, 1609, 1598, 1436, 1368, 1316, 1302, 1278, 1181, 1146, 1111, 1088, 1019, 966, 844, 816, 758, 734, 703, 664, 628, 555, 515. HR-MS (ESI) m/z calcd for $C_{25}H_{30}NaO_6S^+$ 481.16553, found 481.16015, [M+Na$^+$]. The enantiomeric ratio of **34** was determined by HPLC analysis on Chiralpak IC column. Conditions: hexane/isopropanol = 60/40, flow rate = 1.0 mL/min, uv-vis detection at λ = 210 nm, t_R = 38.2 min (minor), 43.2 min (major).

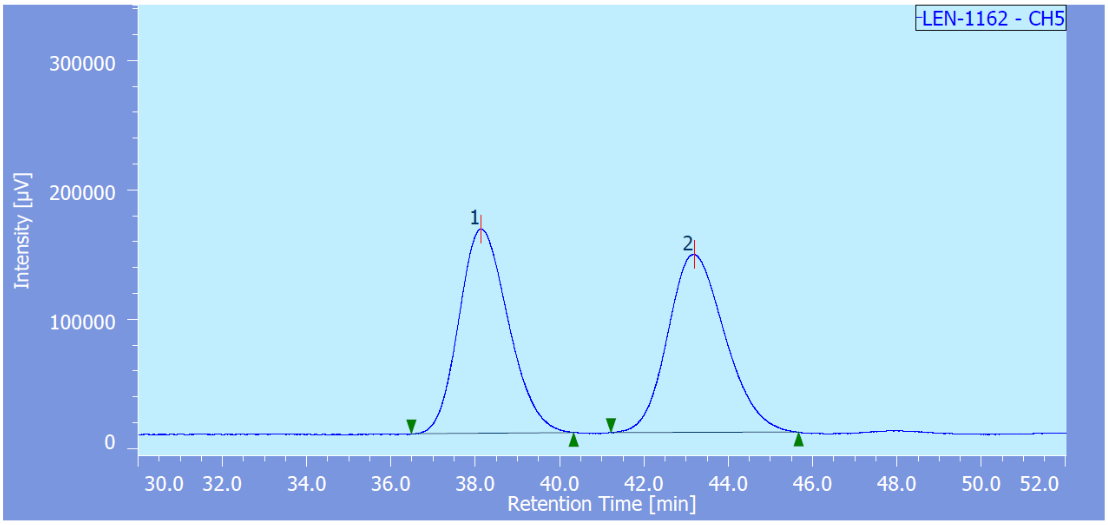

Decision

#	Peak Name	CH	tR [min]	Area [µV·sec]	Height [µV]	Area%	Height%	Quantity	NTP	Resolution	Symmetry Factor	Warning
1	Unknown	5	38.130	12878788	157857	49.772	53.459	N/A	4977	2.181	1.192	
2	Unknown	5	43.190	12996538	137431	50.228	46.541	N/A	4815	N/A	1.166	

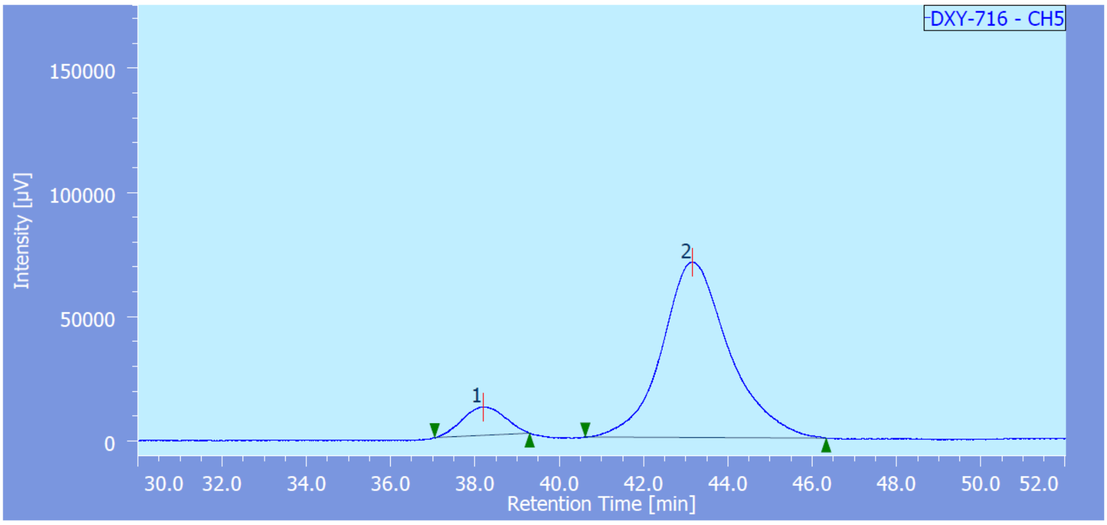

Decision

#	Peak Name	CH	tR [min]	Area [µV·sec]	Height [µV]	Area%	Height%	Quantity	NTP	Resolution	Symmetry Factor	Warning
1	Unknown	5	38.187	809640	11532	9.392	14.054	N/A	6023	2.125	1.011	
2	Unknown	5	43.153	7811071	70525	90.608	85.946	N/A	4034	N/A	1.135	

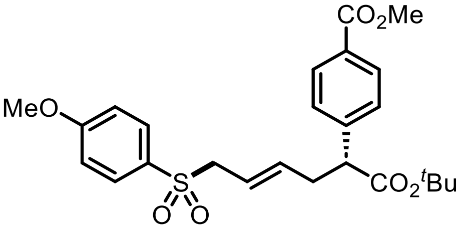

Methyl (*R*,*E*)-4-(1-(*tert*-butoxy)-6-((4-methoxyphenyl)sulfonyl)-1-oxohex-4-en-2-yl)benzoate (35). Colorless oil (Hex/EA = 2/1), 48%, 94:6 er, $[\alpha]_D^{24}$ = -16.2 (c = 0.18, $CHCl_3$). ^1H NMR (400 MHz, $CDCl_3$) δ 7.96 (dt, *J* = 8.5, 1.8 Hz, 2H), 7.73 – 7.63 (m, 2H), 7.27 (dt, *J* =

8.5, 1.6 Hz, 2H), 7.01 – 6.92 (m, 2H), 5.52 – 5.35 (m, 2H), 3.90 (d, *J* = 1.4 Hz, 3H), 3.88 (d, *J* = 1.4 Hz, 3H), 3.65 (d, *J* = 5.9 Hz, 2H), 3.47 (t, *J* = 7.6 Hz, 1H), 2.82 – 2.69 (m, 1H), 2.49 – 2.38 (m, 1H), 1.35 (s, 9H). ^{13}C NMR (101 MHz, $CDCl_3$) δ 171.6, 167.0, 163.8, 143.9, 137.4, 130.7, 130.1, 130.0, 129.3, 128.0, 119.2, 114.3, 81.5, 60.2, 55.8, 52.3, 52.1, 36.1, 28.0. IR (film) ν (cm^{-1}) 2970, 2925, 1724, 1597, 1438, 1368, 1282, 1153, 444, 737. HR-MS (ESI) m/z calcd for $C_{25}H_{30}NaO_7S^+$ 497.16045, found 497.16045, [M+Na$^+$]. The enantiomeric ratio of **35** was determined by HPLC analysis on Chiralpak IC column. Conditions: hexane/isopropanol = 50/50, flow rate = 1.0 mL/min, uv-vis detection at λ = 210 nm, t_R = 37.7 min (minor), 41.9 min (major).

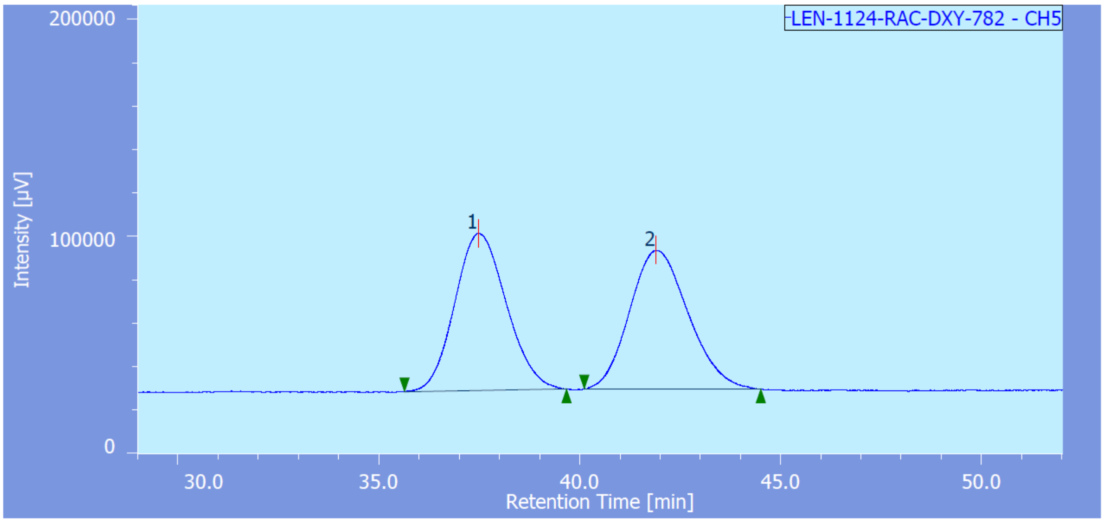

Decision

#	Peak Name	CH	tR [min]	Area [µV·sec]	Height [µV]	Area%	Height%	Quantity	NTP	Resolution	Symmetry Factor	Warning
1	Unknown	5	37.483	6466308	72396	49.950	53.122	N/A	3986	1.747	1.125	
2	Unknown	5	41.897	6479248	63887	50.050	46.878	N/A	3879	N/A	1.173	

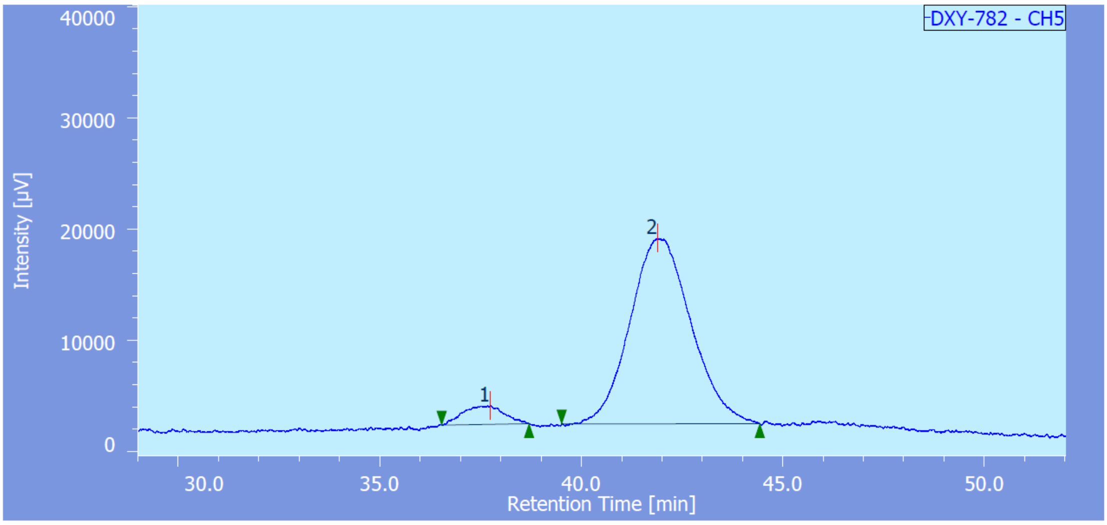

Decision

#	Peak Name	CH	tR [min]	Area [μV·sec]	Height [μV]	Area%	Height%	Quantity	NTP	Resolution	Symmetry Factor	Warning
1	Unknown	5	37.747	120457	1691	6.402	9.192	N/A	5830	1.760	0.892	
2	Unknown	5	41.897	1761101	16703	93.598	90.808	N/A	3712	N/A	1.122	

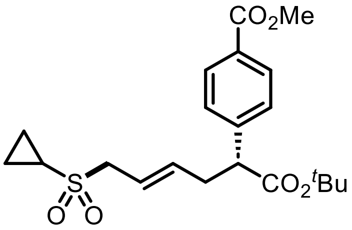

Methyl (*R,E*)-4-(1-(*tert*-butoxy)-6-(cyclopropylsulfonyl)-1-oxohex-4-en-2-yl)benzoate (36). Colorless oil (Hex/EA = 2/1), 64% yield, 93:7 er, $[\alpha]_D^{23}$ = -24.4 (c = 0.20, $CHCl_3$). ^{1}H NMR (400 MHz, $CDCl_3$) δ 8.00 – 7.94 (m, 2H), 7.37 – 7.31 (m, 2H), 5.80 – 5.56 (m, 2H), 3.90 (s, 3H), 3.65 – 3.57 (m, 3H), 2.91 – 2.79 (m, 1H), 2.62 – 2.51 (m, 1H), 2.12 (tt, *J* = 8.0, 4.9 Hz, 1H), 1.36 (s, 9H), 1.19 – 1.09 (m, 2H), 0.90 (dd, *J* = 8.1, 1.7 Hz, 2H). ^{13}C NMR (101 MHz, $CDCl_3$) δ 171.6, 166.9, 143.9, 137.2, 130.0, 129.3, 128.1, 119.4, 81.6, 57.6, 52.3, 52.2, 36.1, 28.2, 28.0, 4.8, 4.6. IR (film) ν (cm^{-1}) 2982, 1718, 1610, 1436, 1419, 1393, 1368, 1319, 1279, 1182, 1144, 1127, 1071, 1040, 1020, 968, 889, 842, 830, 734, 703, 657, 545, 500. HR-MS (ESI) m/z calcd for $C_{21}H_{28}NaO_6S^+$ 431.14988, found 431.15097, [$M+Na^+$]. The enantiomeric ratio of **36** was determined by HPLC analysis on Chiralpak AD-H column. Conditions: hexane/isopropanol = 80/20, flow rate = 1.0 mL/min, uv-vis detection at λ = 254 nm, t_R = 9.7 min (major), 11.0 min (minor).

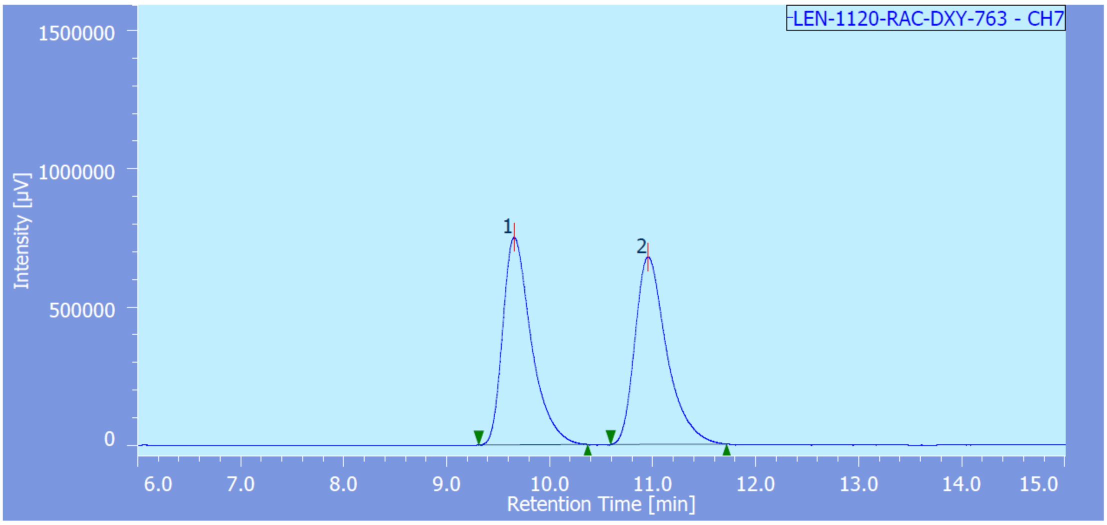

Decision

#	Peak Name	CH	tR [min]	Area [μV·sec]	Height [μV]	Area%	Height%	Quantity	NTP	Resolution	Symmetry Factor	Warning
1	Unknown	7	9.653	14463619	750752	49.907	52.568	N/A	6315	2.541	1.490	
2	Unknown	7	10.953	14517298	677394	50.093	47.432	N/A	6582	N/A	1.438	

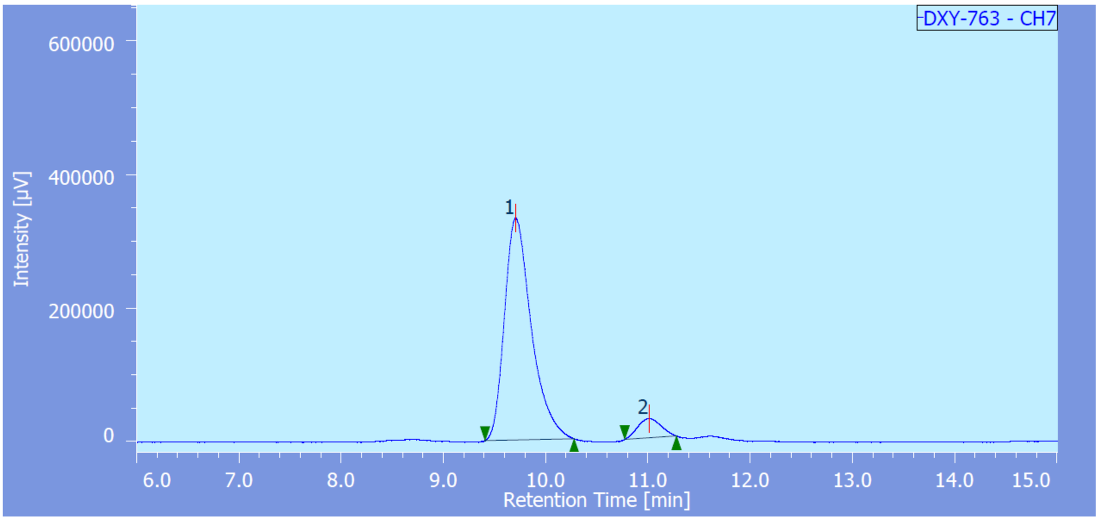

Decision

#	Peak Name	CH	tR [min]	Area [µV·sec]	Height [µV]	Area%	Height%	Quantity	NTP	Resolution	Symmetry Factor	Warning
1	Unknown	7	9.707	6076393	331963	92.989	91.986	N/A	6991	2.885	1.408	
2	Unknown	7	11.010	458164	28921	7.011	8.014	N/A	9951	N/A	1.058	

3.3 General Procedure for asymmetric 1, 5-dicarbofunctionalization of vinyl cyclopropanes (standard conditions B).

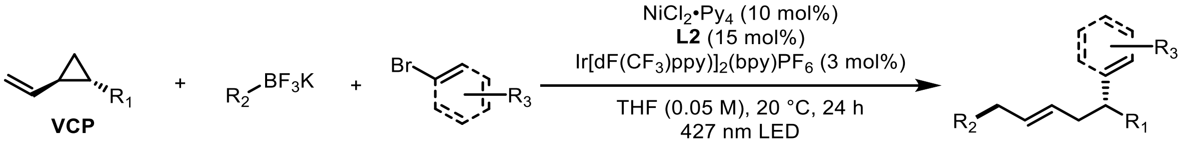

An oven-dried 7.5 mL screw-cap vial equipped with a magnetic stirring bar was charged with R_2-BF_3K (0.25 mmol, 2.5 equiv.), aryl bromide or alkenyl bromide (If solid, 0.1 mmol, 1 equiv.), Ir[dF(CF_3)ppy)]$_2$(bpy)PF_6 (3 mol%), $NiCl_2$·Py_4 (10 mol %) and **L2** (15 mol%) and then introduced into a nitrogen-filled glovebox. Dry THF (2 mL) was added, then the reaction vessel was then capped and removed from the glovebox. Vinyl cyclopropane (0.2 mmol, 2.0 equiv.) and aryl bromide (if liquid, 0.1 mmol, 1 equiv.) were subsequently added. The reaction was stirred (800 rpm) under irradiation with a 427 nm 45 W Kessil LED at 25 °C for 24 h. The reaction was quenched with saturated aq. NaCl (1 mL) and the resulting mixture was extracted with EtOAc (3 × 2 mL). The organic phase was concentrated under reduced pressure and the residue purified by column chromatography on silica gel. The enantiomeric ratio (e.r.) and

the *E*/*Z* ratio of the product were determined by HPLC with a chiral stationary phase.

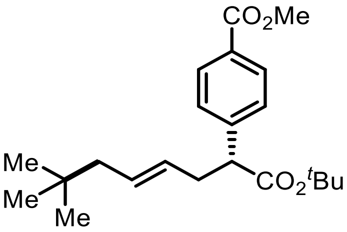

Methyl (*R,E*)-4-(1-(*tert*-butoxy)-7,7-dimethyl-1-oxooct-4-en-2-yl)benzoate (2). Colorless oil (Hex/EA = 20/1), 62%, 93:7 er, *E/Z* = 9/1, $[\alpha]_D^{22}$ = -18.04 (c = 0.18, $CHCl_3$). ^{1}H NMR (400 MHz, $CDCl_3$) δ 8.01 – 7.94 (m, 2H), 7.40 – 7.32 (m, 2H), 5.48 (dtt, *J* = 15.0, 7.4, 1.3 Hz, 1H), 5.31 – 5.21 (m, 1H), 3.90 (s, 3H), 3.55 (t, *J* = 7.8 Hz, 1H), 2.74 (dt, *J* = 15.3, 7.6 Hz, 1H), 2.49 – 2.37 (m, 1H), 1.80 (dt, *J* = 7.5, 1.1 Hz, 2H), 1.38 (d, *J* = 2.4 Hz, 9H), 0.86 (s, 1H, *Z*), 0.79 (s, 8H, *E*). ^{13}C NMR (101 MHz, $CDCl_3$) δ 172.3, 167.2, 144.7, 130.6, 129.9, 129.0, 128.6, 128.2, 81.1, 53.3, 52.2, 47.2, 36.6, 30.9, 29.3, 28.1. IR (film): ν (cm^{-1}) 2952, 1724, 1366, 1276, 757. HR-MS (ESI) calculated $[M+Na]^+$ for $C_{22}H_{32}O_4\,^{23}Na^+$ = 383.21928, found: 383.21949. The enantiomeric ratio of **2** was determined by HPLC analysis on Chiralpak NR column. Conditions: hexane/isopropanol = 99/1, flow rate = 1.0 mL/min, uv-vis detection at λ = 254 nm, t_R = 19.0 min (major, *Z*), 22.7 min (major, *E*), 24.7 min (minor, *Z*), 27.4 min (minor, *E*).

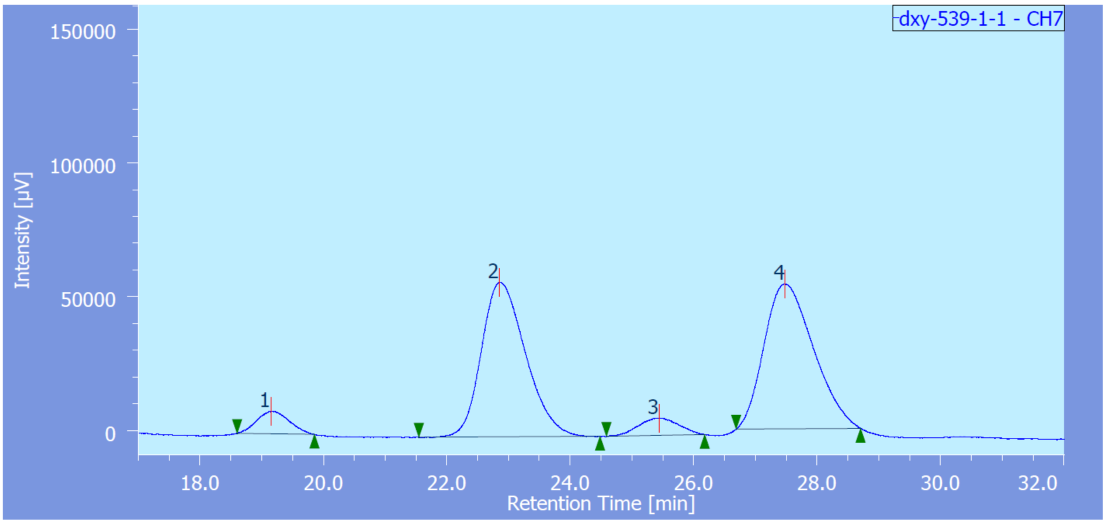

#	Peak Name	CH	tR [min]	Area [μV·sec]	Height [μV]	Area%	Height%	Quantity	NTP	Resolution	Symmetry Factor	Warning
1	Unknown	7	19.150	309688	8370	4.771	6.605	N/A	5751	3.208	1.122	
2	Unknown	7	22.853	2872678	57817	44.259	45.622	N/A	4909	2.018	1.272	
3	Unknown	7	25.443	296199	6462	4.564	5.099	N/A	6435	1.474	0.963	
4	Unknown	7	27.480	3012050	54081	46.406	42.674	N/A	5350	N/A	1.267	

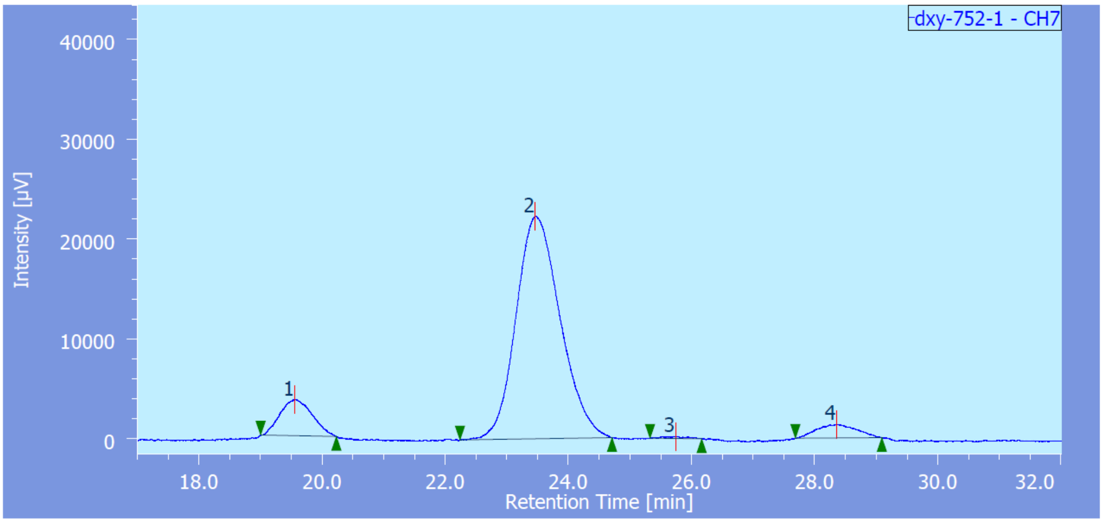

Decision

#	Peak Name	CH	tR [min]	Area [μV·sec]	Height [μV]	Area%	Height%	Quantity	NTP	Resolution	Symmetry Factor	Warning
1	Unknown	7	19.560	131732	3617	10.060	13.187	N/A	5964	3.366	1.141	
2	Unknown	7	23.460	1110694	22237	84.819	81.063	N/A	5130	2.080	1.175	
3	Unknown	7	25.740	5271	208	0.402	0.757	N/A	13441	2.385	1.009	
4	Unknown	7	28.347	61794	1370	4.719	4.993	N/A	7564	N/A	1.058	

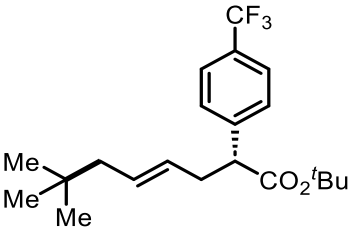

***tert*-Butyl (*R*,*E*)-7,7-dimethyl-2-(4-(trifluoromethyl)phenyl)oct-4-enoate (37)**. Colorless oil (Hex/EA = 20/1), 70%, 92:8 er, *E/Z* = 7/1, $[\alpha]_D^{23}$ = -13.87 (c = 0.14, $CHCl_3$). ^1H NMR (400 MHz, $CDCl_3$) δ 7.59 – 7.53 (m, 2H), 7.41 (dd, *J* = 8.1, 0.5 Hz, 2H), 5.53 – 5.41 (m, 1H), 5.25 (dt, *J* = 15.1, 6.9 Hz, 1H), 3.55 (t, *J* = 7.8 Hz, 1H), 2.81 – 2.68 (m, 1H), 2.49 – 2.39 (m, 1H), 1.82 – 1.78 (m, 2H), 1.39 (s, 9H), 0.85 (s, 1H, *Z*), 0.77 (s, 8H, *E*). ^{13}C NMR (101 MHz, $CDCl_3$) δ 172.1, 143.3, 130.7, 129.4, 128.4, 128.3, 126.2, 125.4 (q, *J* = 3.7 Hz), 81.1, 52.9, 47.0, 36.6, 30.7, 29.1, 27.9. ^{19}F NMR (377 MHz, $CDCl_3$) δ -62.5. IR (film): ν (cm^{-1}) 2956, 1731, 1367, 13251144,1069, 843, 457. HR-MS (ESI) calculated $[M+Na]^+$ for $C_{21}H_{29}F_3O_2$ $^{23}Na^+$ = 393.20119, found: 393.20712. The enantiomeric ratio of **37** was determined by HPLC analysis on Chiralpak NR column. Conditions: hexane/ethanol = 99.5/0.5, flow rate = 0.5 mL/min, uv-vis detection at λ = 220 nm, t_R = 17.8 min (*Z*), 19.0 min (minor, *E*), 20.3 min (major, *E*).

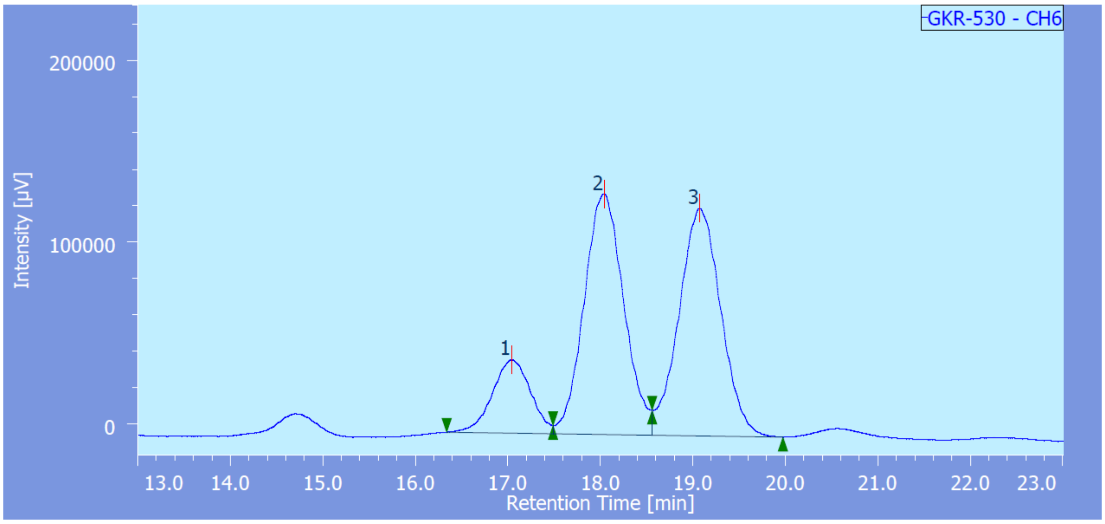

Decision

#	Peak Name	CH	tR [min]	Area [µV·sec]	Height [µV]	Area%	Height%	Quantity	NTP	Resolution	Symmetry Factor	Warning
1	Unknown	6	17.040	1158638	40393	12.801	13.571	N/A	8179	1.302	N/A	
2	Unknown	6	18.040	3953671	132202	43.683	44.417	N/A	8417	1.277	N/A	
3	Unknown	6	19.070	3938580	125046	43.516	42.012	N/A	8415	N/A	N/A	

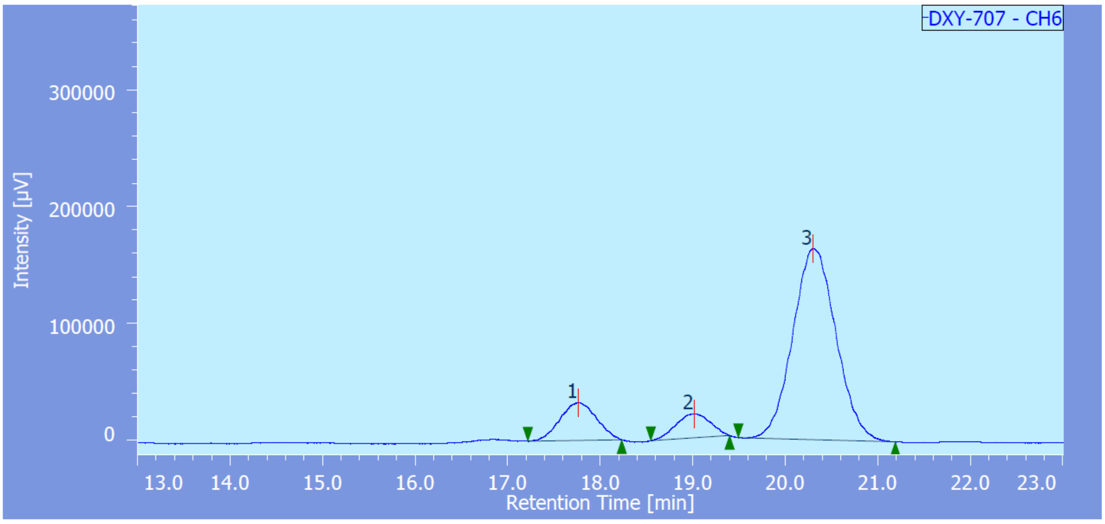

Decision

#	Peak Name	CH	tR [min]	Area [µV·sec]	Height [µV]	Area%	Height%	Quantity	NTP	Resolution	Symmetry Factor	Warning
1	Unknown	6	17.763	886755	32339	13.160	14.963	N/A	9364	1.726	1.002	
2	Unknown	6	19.017	536577	20669	7.963	9.564	N/A	11103	1.643	0.934	
3	Unknown	6	20.300	5314835	163113	78.877	75.473	N/A	9239	N/A	1.070	

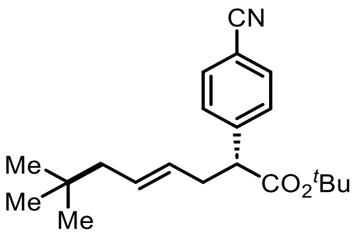

***tert*-Butyl (*R,E*)-2-(4-cyanophenyl)-7,7-dimethyloct-4-enoate (38)**. Colorless oil (Hex/EA = 20/1), 65%, 93:7 er, *E/Z* = 9/1, $[\alpha]_D^{22}$ = -11.54 (c = 0.17, $CHCl_3$). ^{1}H NMR (400 MHz, $CDCl_3$)

δ 7.63 – 7.57 (m, 2H), 7.43 – 7.37 (m, 2H), 5.46 (dt, J = 15.0, 7.5 Hz, 1H), 5.23 (dt, J = 15.1, 6.9 Hz, 1H), 3.55 (t, J = 7.7 Hz, 1H), 2.74 (dt, J = 14.3, 7.1 Hz, 1H), 2.49 – 2.35 (m, 1H), 1.80 (ddd, J = 7.8, 2.7, 1.2 Hz, 2H), 1.39 (s, 9H), 0.85 (s, 1H, Z), 0.77 (s, 8H, E). ^{13}C NMR (101 MHz, $CDCl_3$) δ 171.8, 144.8, 132.4, 131.1, 129.0, 128.1, 119.0, 111.1, 81.5, 53.3, 47.1, 36.6, 30.9, 29.3, 28.1. IR (film): ν (cm^{-1}) 2930, 2320, 1728, 1276, 1147. HR-MS (ESI) calculated $[M+Na]^+$ for $C_{21}H_{29}O_2N\,^{23}Na^+$ = 350.20905, found: 350.20930. The enantiomeric ratio of **38** was determined by HPLC analysis on Chiralpak NR column. Conditions: hexane/isopropanol = 99/1, flow rate = 1.0 mL/min, uv-vis detection at λ = 220 nm, t_R = 20.3 min (Z), 22.1 min (minor, E), 25.1 min (major, E).

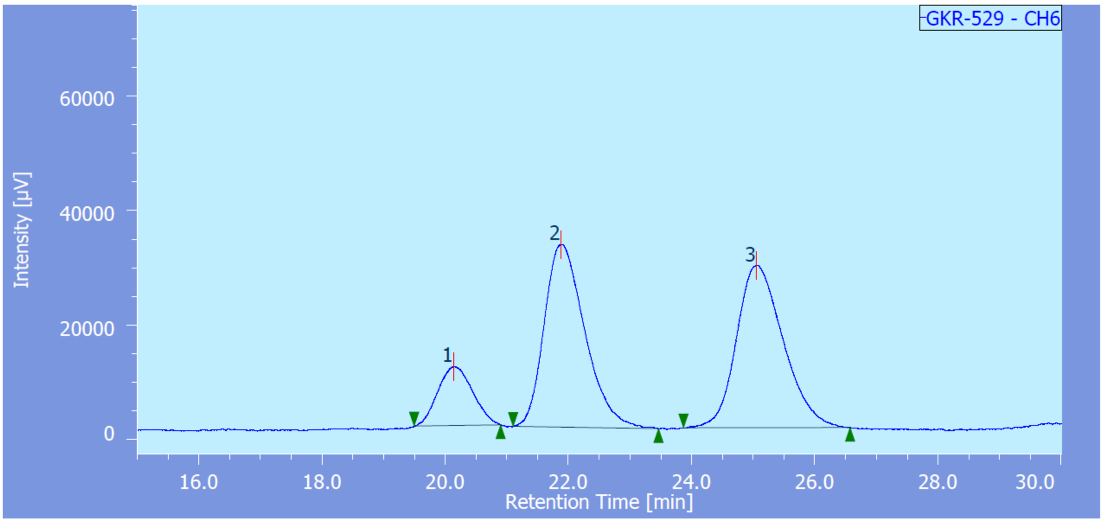

Decision

#	Peak Name	CH	tR [min]	Area [µV·sec]	Height [µV]	Area%	Height%	Quantity	NTP	Resolution	Symmetry Factor	Warning
1	Unknown	6	20.137	405434	10292	12.034	14.593	N/A	5650	1.542	1.110	
2	Unknown	6	21.880	1465407	31893	43.496	45.221	N/A	5356	2.475	1.336	
3	Unknown	6	25.053	1498198	28342	44.470	40.186	N/A	5310	N/A	1.228	

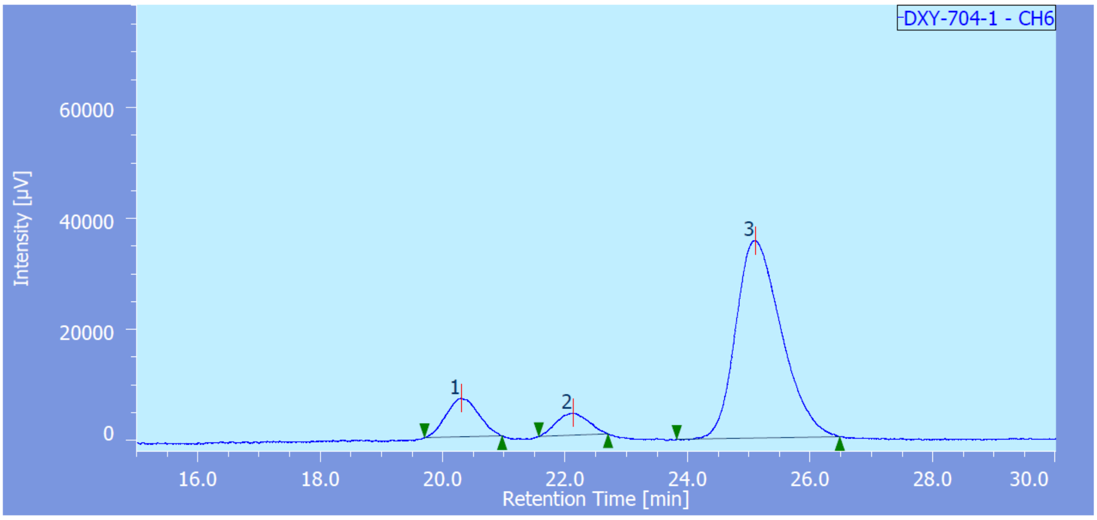

Decision

#	Peak Name	CH	tR [min]	Area [µV·sec]	Height [µV]	Area%	Height%	Quantity	NTP	Resolution	Symmetry Factor	Warning
1	Unknown	6	20.300	257495	6972	11.430	14.956	N/A	6424	1.777	1.080	
2	Unknown	6	22.123	144197	3985	6.401	8.547	N/A	7176	2.481	1.017	
3	Unknown	6	25.100	1851028	35664	82.169	76.497	N/A	5437	N/A	1.277	

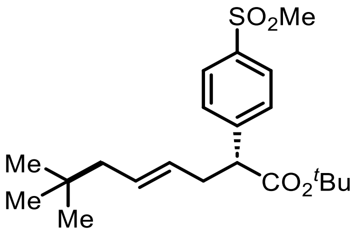

***tert*-Butyl (*R*,*E*)-7,7-dimethyl-2-(4-(methylsulfonyl)phenyl)oct-4-enoate (39)**. Colorless oil (Hex/EA = 10/1), 58%, 92:8 er, *E*/*Z* = 7/1, $[\alpha]_D^{22}$ = -11.45 (c = 0.20, $CHCl_3$). ^{1}H NMR (400 MHz, $CDCl_3$) δ 7.90 – 7.85 (m, 2H), 7.50 (d, *J* = 8.4 Hz, 2H), 5.46 (dt, *J* = 15.0, 7.3 Hz, 1H), 5.29 – 5.18 (m, 1H), 3.60 (t, *J* = 7.8 Hz, 1H), 3.04 (s, 3H), 2.81 – 2.71 (m, 1H), 2.52 – 2.39 (m, 1H), 1.85 – 1.73 (m, 2H), 1.39 (s, 9H), 0.85 (s, 1H, *Z*), 0.76 (s, 8H, *E*). ^{13}C NMR (101 MHz, $CDCl_3$) δ 171.7, 145.7, 139.1, 131.0, 129.1, 128.0, 127.6, 81.4, 53.0, 47.0, 44.5, 36.6, 30.8, 29.1, 28.0. IR (film): ν (cm^{-1}) 2952, 1718, 1608, 1435, 1365, 1275, 1146, 759, 702. HR-MS (ESI) calculated $[M+Na]^+$ for $C_{21}H_{32}O_4$ $^{23}Na^+$ = 403.19135, found: 403.19195. The enantiomeric ratio of **39** was determined by HPLC analysis on Chiralpak NR column. Conditions: hexane/isopropanol = 90/10, flow rate = 1.0 mL/min, uv-vis detection at λ = 220 nm, t_R = 45.6 min (*Z*), 54.4 min (major, *E*), 59.3 min (minor, *E*).

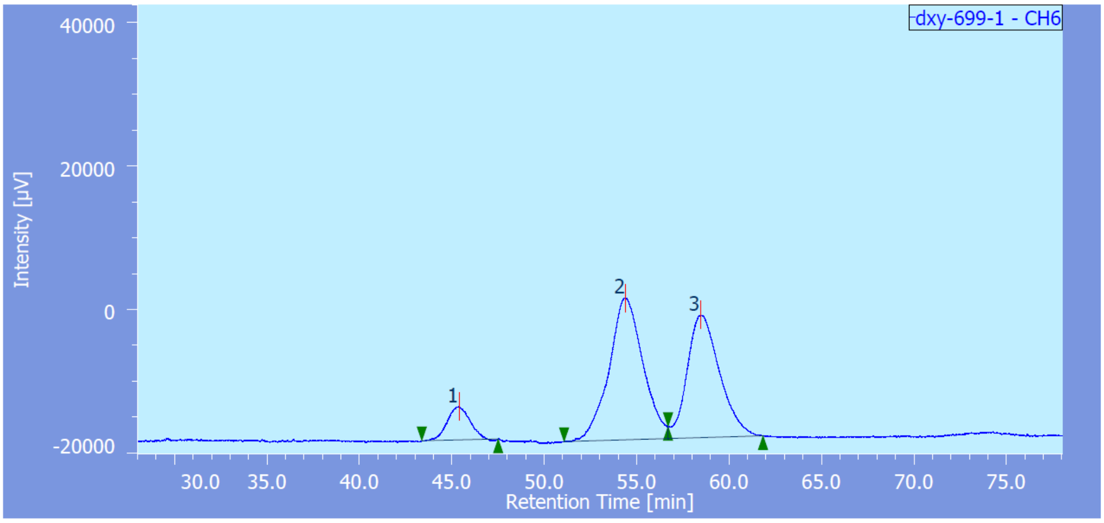

Decision

#	Peak Name	CH	tR [min]	Area [µV·sec]	Height [µV]	Area%	Height%	Quantity	NTP	Resolution	Symmetry Factor	Warning
1	Unknown	6	45.377	396379	4678	7.892	11.241	N/A	6378	3.296	0.987	
2	Unknown	6	54.393	2532749	19815	50.428	47.615	N/A	4586	1.272	N/A	
3	Unknown	6	58.440	2093332	17122	41.679	41.144	N/A	5450	N/A	N/A	

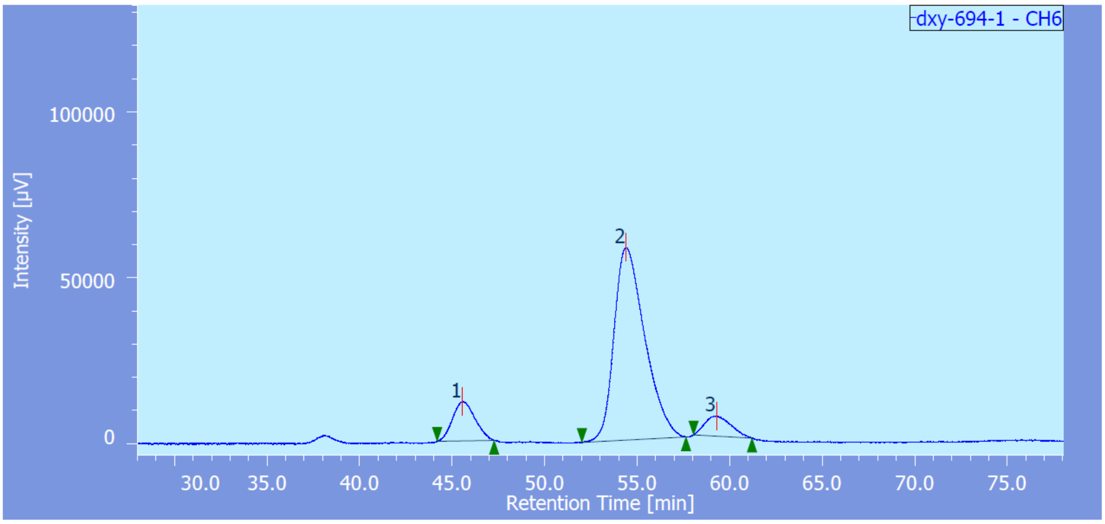

Decision

#	Peak Name	CH	tR [min]	Area [µV·sec]	Height [µV]	Area%	Height%	Quantity	NTP	Resolution	Symmetry Factor	Warning
1	Unknown	6	45.550	1011866	11866	12.165	15.622	N/A	6236	3.333	1.143	
2	Unknown	6	54.390	6702401	57990	80.578	76.343	N/A	5220	1.698	1.394	
3	Unknown	6	59.277	603639	6103	7.257	8.035	N/A	7378	N/A	1.239	

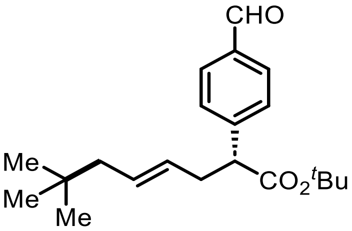

***tert*-Butyl (*R*,*E*)-2-(4-formylphenyl)-7,7-dimethyloct-4-enoate (40)**. Colorless oil (Hex/EA = 10/1), 50%, 92:8 er, *E*/*Z* = 8/1, $[\alpha]_D^{23}$ = -23.46 (c = 0.16, $CHCl_3$). ^{1}H NMR (400 MHz, $CDCl_3$) δ 9.98 (s, 1H), 7.86 – 7.79 (m, 2H), 7.51 – 7.42 (m, 2H), 5.48 (dt, *J* = 15.0, 7.4 Hz, 1H), 5.26 (dt, *J* = 15.1, 6.9 Hz, 1H), 3.58 (t, *J* = 7.8 Hz, 1H), 2.82 – 2.71 (m, 1H), 2.45 (dtd, *J* = 14.3, 7.4, 1.3 Hz, 1H), 1.83 – 1.74 (m, 2H), 1.38 (s, 9H), 0.85 (s, 1H), 0.77 (s, 8H). ^{13}C NMR (101 MHz, $CDCl_3$) δ 192.1, 172.0, 146.5, 135.4, 130.8, 130.0, 128.9, 128.4, 81.3, 53.4, 47.1, 36.6, 30.9, 29.3, 28.1. IR (film): ν (cm^{-1}) 2932, 1727, 1706, 1606, 1366, 1143, 844. HR-MS (ESI) calculated $[M+Na]^+$ for $C_{21}H_{30}O_3$ $^{23}Na^+$ = 353.20872, found: 353.20881. The enantiomeric ratio of **40** was determined by HPLC analysis on Chiralpak NR column. Conditions: hexane/isopropanol = 99/1, flow rate = 1.0 mL/min, uv-vis detection at λ = 254 nm, t_R = 19.7 min (*Z*), 22.6 min (major, *E*), 24.7 min (minor, *E*).

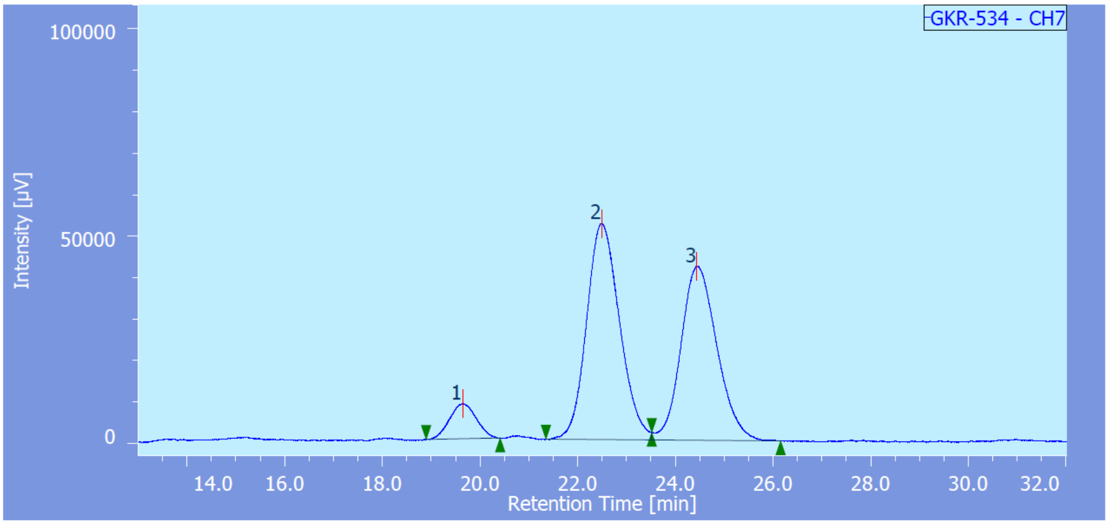

Decision

#	Peak Name	CH	tR [min]	Area [µV·sec]	Height [µV]	Area%	Height%	Quantity	NTP	Resolution	Symmetry Factor	Warning
1	Unknown	7	19.640	324016	8451	6.575	8.240	N/A	5631	2.514	1.035	
2	Unknown	7	22.497	2448389	52107	49.683	50.807	N/A	5335	1.518	1.150	
3	Unknown	7	24.440	2155568	42000	43.741	40.953	N/A	5364	N/A	1.148	

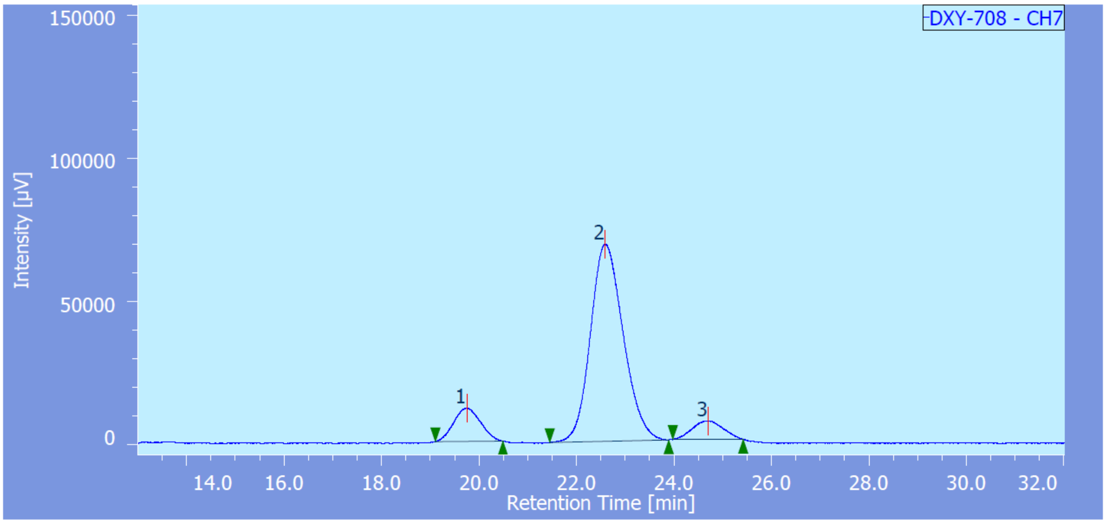

Decision

#	Peak Name	CH	tR [min]	Area [µV·sec]	Height [µV]	Area%	Height%	Quantity	NTP	Resolution	Symmetry Factor	Warning
1	Unknown	7	19.747	446420	11705	11.305	13.449	N/A	5671	2.510	1.070	
2	Unknown	7	22.590	3218261	68900	81.500	79.168	N/A	5459	1.725	1.151	
3	Unknown	7	24.700	284097	6426	7.195	7.384	N/A	6460	N/A	1.016	

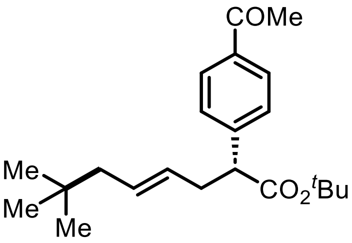

***tert*-Butyl (*R*,*E*)-2-(4-acetylphenyl)-7,7-dimethyloct-4-enoate (41)**. Colorless oil (Hex/EA = 20/1), 59%, 92:8 er, *E*/*Z* = 7/1, $[\alpha]_D^{23}$ = -15.41 (c = 0.18, $CHCl_3$). ^1H NMR (400 MHz, $CDCl_3$)

δ 7.94 – 7.86 (m, 2H), 7.39 (d, *J* = 8.4 Hz, 2H), 5.52 – 5.43 (m, 1H), 5.32 – 5.21 (m, 1H), 3.56 (t, *J* = 7.7 Hz, 1H), 2.75 (dt, *J* = 14.0, 7.2 Hz, 1H), 2.59 (d, *J* = 1.3 Hz, 3H), 2.49 – 2.39 (m, 1H), 1.80 (d, *J* = 7.4 Hz, 2H), 1.38 (s, 9H), 0.86 (s, 1H, *Z*), 0.78 (s, 8H, *E*). ^{13}C NMR (101 MHz, $CDCl_3$) δ 198.0, 172.2, 145.0, 136.1, 130.7, 128.7, 128.5, 128.4, 81.2, 53.2, 47.2, 36.6, 30.9, 29.3, 28.1, 26.8. IR (film): ν (cm^{-1}) 2956, 2871, 1727, 1685, 1275, 1122, 749. HR-MS (ESI) calculated $[M+Na]^+$ for $C_{22}H_{32}O_3\,^{23}Na^+$ = 367.22437, found: 367.22434. The enantiomeric ratio of **41** was determined by HPLC analysis on Chiralpak AD-H column. Conditions: hexane/isopropanol = 97/3, flow rate = 1.0 mL/min, uv-vis detection at λ = 254 nm, t_R = 5.0 min (*Z*), 5.6 min (minor, *E*), 6.0 min (major, *E*).

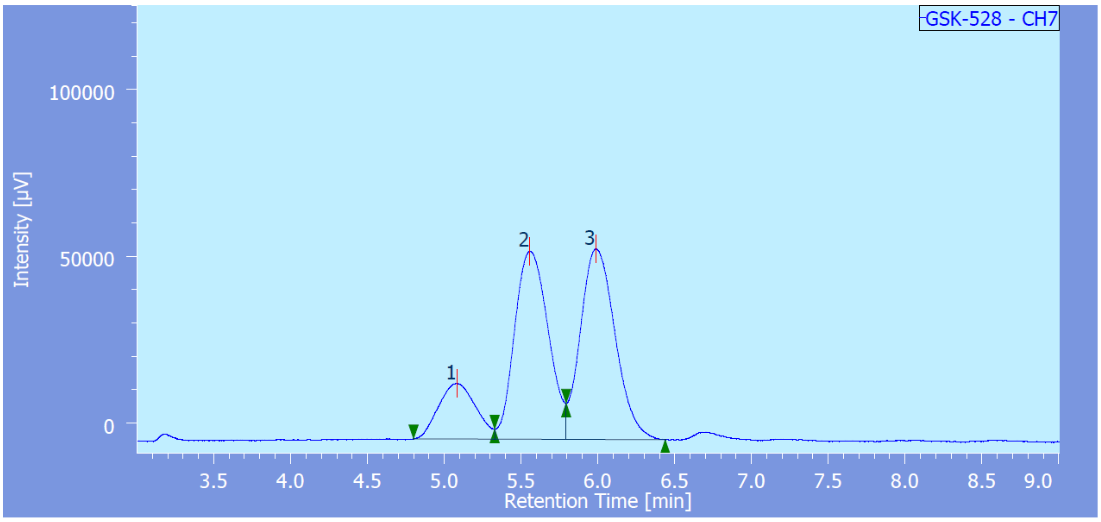

Decision

#	Peak Name	CH	tR [min]	Area [µV·sec]	Height [µV]	Area%	Height%	Quantity	NTP	Resolution	Symmetry Factor	Warning
1	Unknown	7	5.083	279842	16677	13.697	12.808	N/A	1873	1.070	N/A	
2	Unknown	7	5.557	861386	56364	42.161	43.287	N/A	2835	1.031	N/A	
3	Unknown	7	5.987	901864	57167	44.142	43.905	N/A	3269	N/A	N/A	

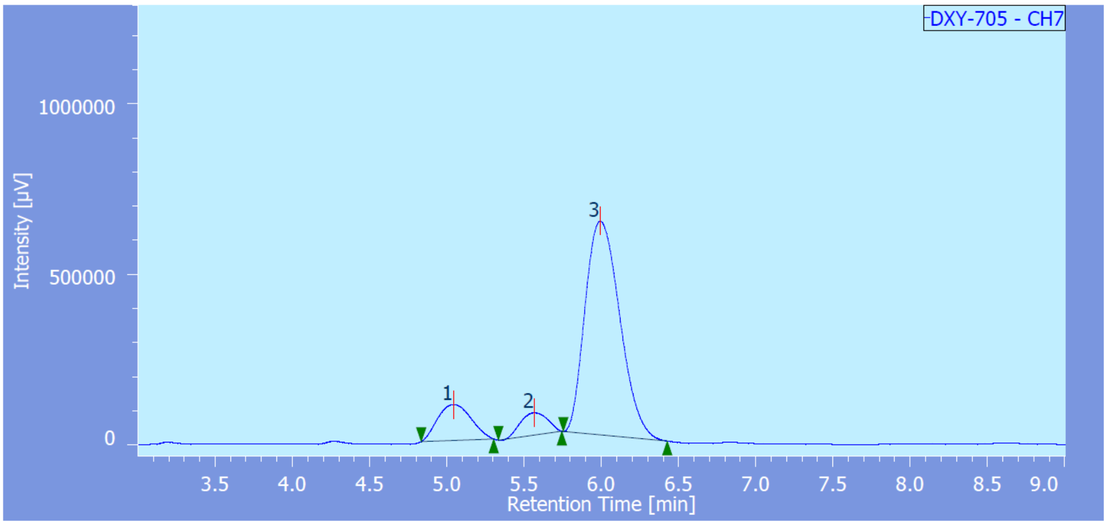

Decision

#	Peak Name	CH	tR [min]	Area [µV·sec]	Height [µV]	Area%	Height%	Quantity	NTP	Resolution	Symmetry Factor	Warning
1	Unknown	7	5.043	1589089	105618	13.022	13.227	N/A	2266	1.347	1.112	
2	Unknown	7	5.567	815403	65956	6.682	8.260	N/A	3923	1.098	0.976	
3	Unknown	7	5.993	9798896	626915	80.297	78.513	N/A	3205	N/A	1.260	

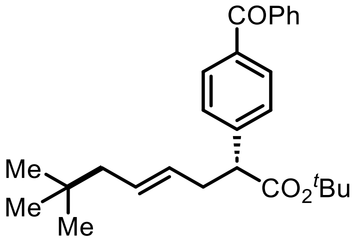

***tert*-Butyl (*R,E*)-2-(4-benzoylphenyl)-7,7-dimethyloct-4-enoate (42)**. Colorless oil (Hex/EA = 20/1), 41%, 90:10 er, *E/Z* = 8/1, $[\alpha]_D^{22}$ = -7.3 (c = 0.20, $CHCl_3$). ^{1}H NMR (400 MHz, CD_2Cl_2) δ 7.70 – 7.60 (m, 4H), 7.52 – 7.45 (m, 2H), 7.41 – 7.35 (m, 3H), 7.30 (d, *J* = 8.1 Hz, 2H), 5.45 – 5.36 (m, 1H), 5.24 – 5.15 (m, 1H), 3.48 (t, *J* = 7.8 Hz, 1H), 2.70 – 2.59 (m, 1H), 2.36 (ddd, *J* = 14.1, 7.2, 1.0 Hz, 1H), 1.71 (ddd, *J* = 7.6, 2.9, 1.1 Hz, 2H), 1.28 (s, 9H), 0.76 (s, 1H), 0.68 (s, 8H). ^{13}C NMR (101 MHz, $CDCl_3$) δ 197.9, 173.9, 146.2, 139.7, 138.2, 134.3, 134.2, 132.4, 132.1, 131.8, 131.8, 130.5, 130.2, 130.1, 129.9, 82.7, 48.8, 38.4, 32.5, 30.8, 29.7, 29.6. IR (film): ν (cm^{-1}) 2935, 1716, 1660, 1605, 1277, 1143, 701. HR-MS (ESI) calculated $[M+Na]^+$ for $C_{27}H_{34}O_3$ $^{23}Na^+$ = 429.24002, found:429.24020. The enantiomeric ratio of **42** was determined by HPLC analysis on Chiralpak NR column. Conditions: hexane/isopropanol = 95/5, flow rate = 1.0 mL/min, uv-vis detection at λ = 210 nm, t_R = 20.2 min (*Z*), 23.1 min (minor, *E*), 25.4 min (major, *E*).

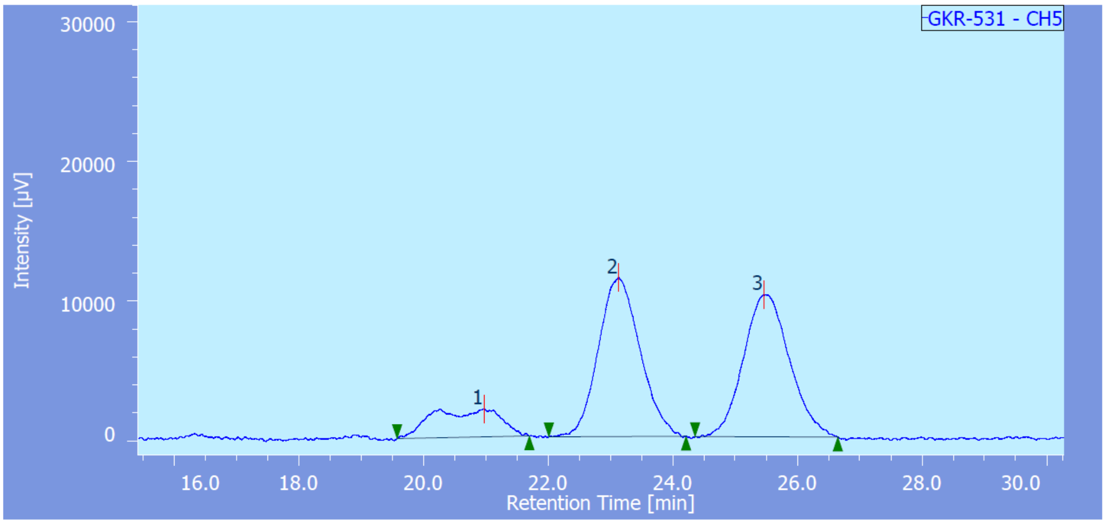

Decision

#	Peak Name	CH	tR [min]	Area [μV·sec]	Height [μV]	Area%	Height%	Quantity	NTP	Resolution	Symmetry Factor	Warning
1	Unknown	5	20.970	155546	2007	12.866	8.518	N/A	1298	1.214	0.754	
2	Unknown	5	23.127	528587	11374	43.721	48.267	N/A	5622	1.799	1.108	
3	Unknown	5	25.457	524857	10184	43.413	43.215	N/A	5575	N/A	1.163	

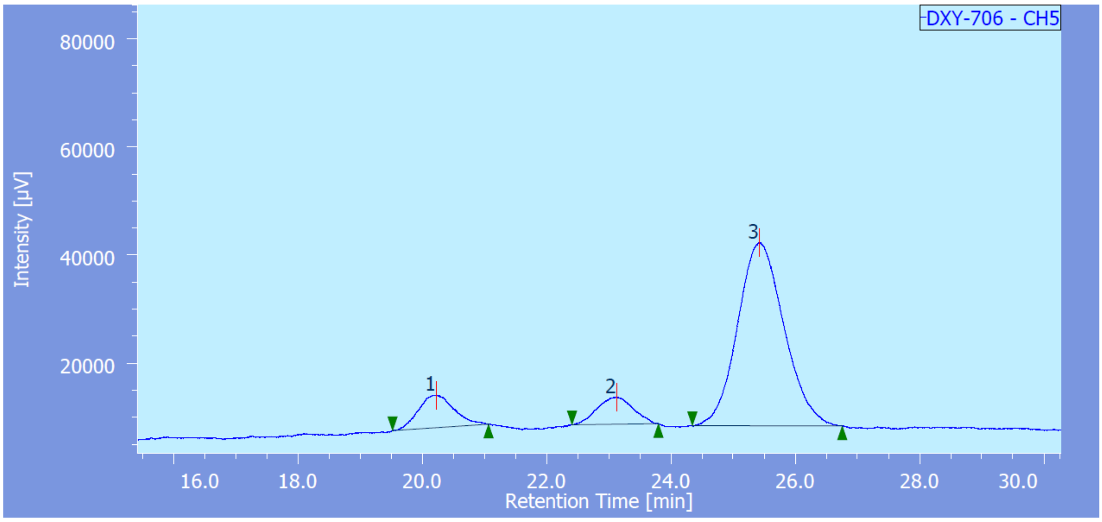

Decision

#	Peak Name	CH	tR [min]	Area [μV·sec]	Height [μV]	Area%	Height%	Quantity	NTP	Resolution	Symmetry Factor	Warning
1	Unknown	5	20.230	240359	5998	10.941	13.386	N/A	5670	2.644	1.131	
2	Unknown	5	23.127	200998	4968	9.150	11.087	N/A	6796	1.854	0.949	
3	Unknown	5	25.423	1755425	33843	79.909	75.527	N/A	5578	N/A	1.112	

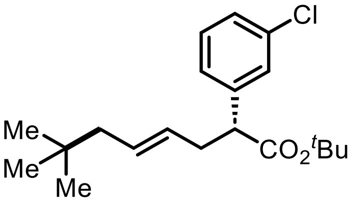

***tert*-Butyl (*R*,*E*)-2-(3-chlorophenyl)-7,7-dimethyloct-4-enoate (43)**. Colorless oil (Hex/EA = 20/1), 42%, 90:10 er, *E/Z* = 6/1, $[\alpha]_D^{23}$ = -13.82 (c = 0.18, $CHCl_3$). ^1H NMR (400 MHz, $CDCl_3$) δ 7.31 – 7.28 (m, 1H), 7.23 – 7.16 (m, 3H), 5.48 (dt, *J* = 14.9, 7.4 Hz, 1H), 5.26 (dt, *J* = 15.0, 6.8 Hz, 1H), 3.48 – 3.39 (m, 1H), 2.76 – 2.66 (m, 1H), 2.40 (dt, *J* = 14.1, 7.1 Hz, 1H), 1.81 (d, *J* = 8.3 Hz, 2H), 1.39 (s, 9H), 0.86 (s, 1H, *Z*), 0.79 (s, 8H, *E*). ^{13}C NMR (126 MHz, $CDCl_3$) δ 172.4, 141.4, 134.3, 130.6, 129.8, 128.6, 128.4, 127.3, 126.3, 81.1, 52.9, 47.2, 36.8, 30.9, 29.3, 28.1. IR (film): ν (cm^{-1}) 2928, 2360, 1727, 1366, 1141, 766, 692. HR-MS (ESI) calculated $[M+Na]^+$ for $C_{20}H_{29}O_2Cl\,{}^{23}Na^+$ = 359.17483, found: 359.17489. The enantiomeric ratio of **43** was determined by HPLC analysis on Chiralpak AD-H column. Conditions: hexane/isopropanol = 98/2, flow rate = 0.5 mL/min, uv-vis detection at λ = 220 nm, t_R = 8.4 min (major, *Z*), 9.3 min (minor, *Z*), 10.5 min (major, *E*), 11.5 min (minor, *E*).

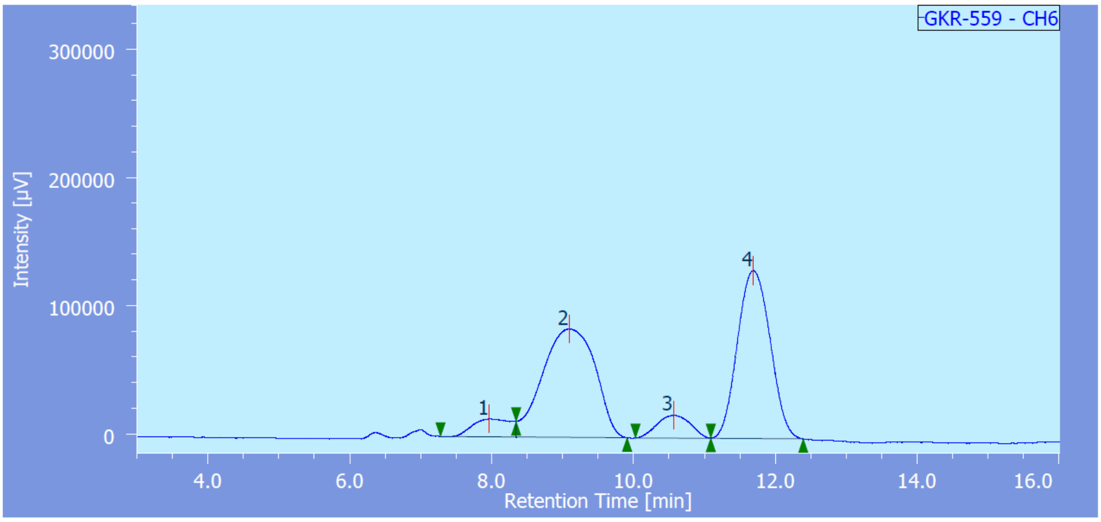

Decision

#	Peak Name	CH	tR [min]	Area [μV·sec]	Height [μV]	Area%	Height%	Quantity	NTP	Resolution	Symmetry Factor	Warning
1	Unknown	6	7.970	526036	13900	5.443	5.629	N/A	N/A	N/A	N/A	
2	Unknown	6	9.090	4365273	84469	45.170	34.208	N/A	627	1.235	N/A	
3	Unknown	6	10.560	583789	17729	6.041	7.180	N/A	2043	1.246	1.001	
4	Unknown	6	11.687	4189089	130829	43.347	52.983	N/A	2833	N/A	1.063	

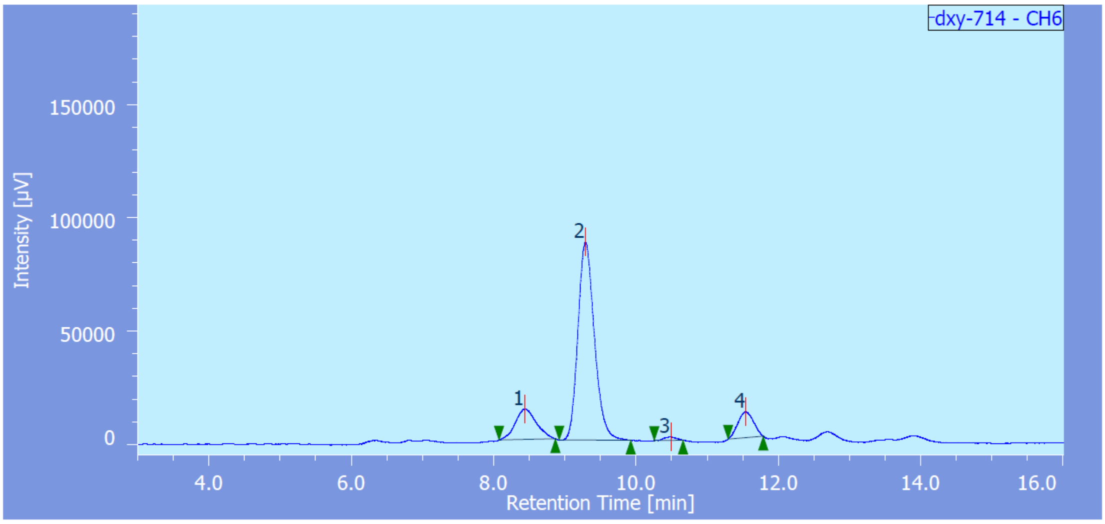

Decision

#	Peak Name	CH	tR [min]	Area [μV·sec]	Height [μV]	Area%	Height%	Quantity	NTP	Resolution	Symmetry Factor	Warning
1	Unknown	6	8.437	277434	13430	14.800	11.812	N/A	3962	1.792	1.100	
2	Unknown	6	9.287	1404281	87396	74.913	76.862	N/A	8003	3.101	1.212	
3	Unknown	6	10.487	18778	1508	1.002	1.326	N/A	13525	2.691	0.941	
4	Unknown	6	11.543	174067	11370	9.286	10.000	N/A	11699	N/A	0.990	

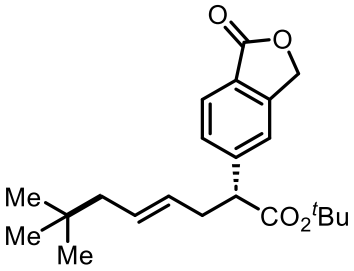

***tert*-Butyl (*R*,*E*)-7,7-dimethyl-2-(1-oxo-1,3-dihydroisobenzofuran-5-yl)oct-4-enoate (44)**. Colorless oil (Hex/EA = 20/1), 67%, 91:9 er, *E/Z* = 7/1, $[\alpha]_D^{23}$ = -15.89 (c = 0.20, $CHCl_3$). ^1H NMR (400 MHz, $CDCl_3$) δ 7.86 (dd, *J* = 7.9, 0.8 Hz, 1H), 7.49 – 7.42 (m, 2H), 5.48 (dt, *J* = 15.0, 7.4 Hz, 1H), 5.32 – 5.20 (m, 3H), 3.63 (t, *J* = 7.7 Hz, 1H), 2.78 (dt, *J* = 13.8, 6.8 Hz, 1H), 2.46 (dt, *J* = 14.5, 7.6 Hz, 1H), 1.80 (ddd, *J* = 7.6, 3.0, 1.2 Hz, 2H), 1.40 (s, 10H), 0.86 (s, 1H, *Z*), 0.77 (s, 8H, *E*). ^{13}C NMR (101 MHz, $CDCl_3$) δ 172.0, 171.0, 147.2, 146.4, 131.1, 129.5, 128.1, 125.9, 124.8, 121.7, 81.6, 69.7, 53.4, 47.1, 37.0, 30.9, 29.3, 28.1. IR (film): ν (cm^{-1}) 2932, 1763, 1726, 1619, 1145, 765. HR-MS (ESI) calculated $[M+Na]^+$ for $C_{22}H_{30}O_4{}^{23}Na^+$ = 381.20363, found: 381.20370. The enantiomeric ratio of **44** was determined by HPLC analysis on Chiralpak NR-3 column. Conditions: hexane/isopropanol = 90/10, flow rate = 1.0 mL/min, uv-vis detection at λ = 210 nm, t_R = 41.5 min (major, *Z*), 42.7 min (major, *E*), 46.3 min (minor, *Z*), 47.9 min (minor, *E*).

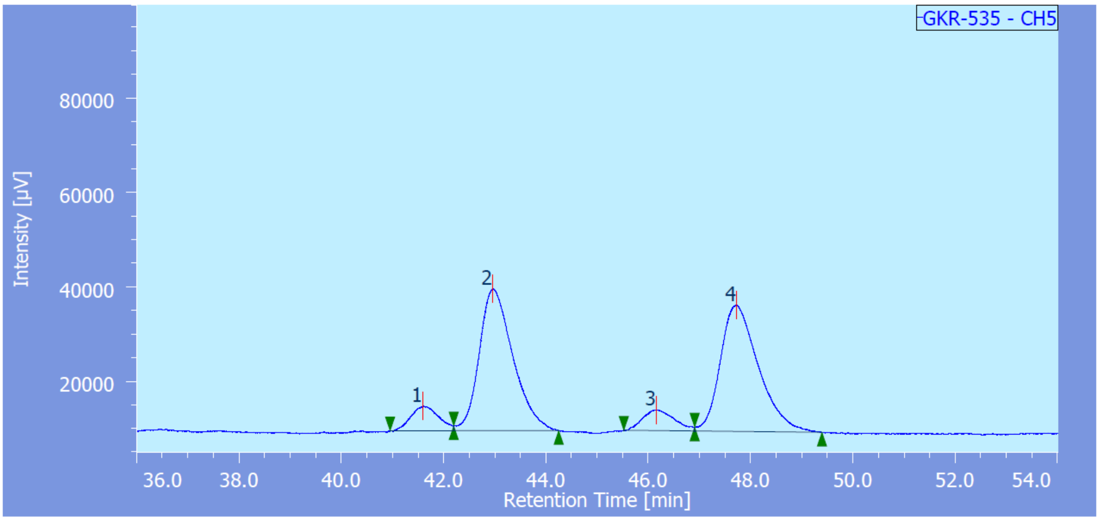

Decision

#	Peak Name	CH	tR [min]	Area [µV·sec]	Height [µV]	Area%	Height%	Quantity	NTP	Resolution	Symmetry Factor	Warning
1	Unknown	5	41.597	200210	5243	6.352	7.890	N/A	25403	1.230	N/A	
2	Unknown	5	42.960	1374886	30030	43.622	45.189	N/A	21276	2.734	1.303	
3	Unknown	5	46.157	190820	4431	6.054	6.668	N/A	25062	1.257	N/A	
4	Unknown	5	47.723	1385935	26750	43.972	40.253	N/A	20517	N/A	1.320	

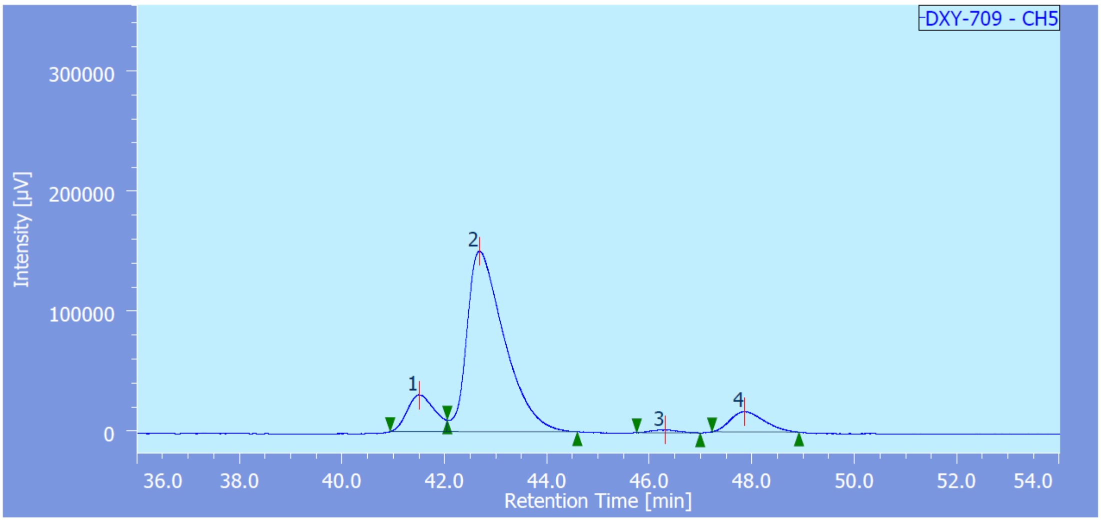

Decision

#	Peak Name	CH	tR [min]	Area [µV·sec]	Height [µV]	Area%	Height%	Quantity	NTP	Resolution	Symmetry Factor	Warning
1	Unknown	5	41.503	1144454	30452	11.859	15.210	N/A	24673	1.003	N/A	
2	Unknown	5	42.680	7633142	150392	79.096	75.118	N/A	17335	3.084	N/A	
3	Unknown	5	46.307	91208	2436	0.945	1.217	N/A	30446	1.345	1.107	
4	Unknown	5	47.853	781696	16929	8.100	8.456	N/A	23625	N/A	1.317	

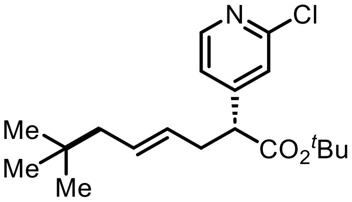

***tert*-Butyl (*R,E*)-2-(2-chloropyridin-4-yl)-7,7-dimethyloct-4-enoate (45)**. Colorless oil (Hex/EA = 5/1), 30%, 90:10 er, *E/Z* = 9/1, $[\alpha]_D^{22}$ = -9.73 (c = 0.18, $CHCl_3$). ^1H NMR (400 MHz, $CDCl_3$) δ 8.31 (dd, *J* = 5.1, 0.7 Hz, 1H), 7.29 – 7.26 (m, 1H), 7.16 (dd, *J* = 5.4, 1.3 Hz, 1H), 5.48 (dt, *J* = 15.0, 7.4 Hz, 1H), 5.23 (dt, *J* = 15.1, 6.9 Hz, 1H), 3.47 (t, *J* = 7.7 Hz, 1H), 2.72 (dt, *J* = 14.4, 7.3 Hz, 1H), 2.49 – 2.37 (m, 1H), 1.80 (d, *J* = 7.7 Hz, 2H), 1.40 (s, 9H), 0.86 (s, 1H, *Z*), 0.79 (s, 8H, *E*). ^{13}C NMR (101 MHz, $CDCl_3$) δ 171.0, 151.9, 151.6, 149.7, 131.5, 127.6, 124.1, 122.2, 81.9, 52.4, 47.1, 36.3, 30.9, 29.3, 28.1. IR (film): ν (cm^{-1}) 2954, 1727, 1591, 1547, 1366, 1145, 843, 529. HR-MS (ESI) calculated $[M+H]^+$ for $C_{19}H_{28}ClNO_2\,{}^1H^+$ = 338.18813, found: 338.181781. The enantiomeric ratio of **45** was determined by HPLC analysis on Chiralpak IC column. Conditions: hexane/isopropanol = 99/1, flow rate = 1.0 mL/min, uv-vis detection at λ = 220 nm, t_R = 12.8 min (minor, *Z*), 14.5 min (minor, *E*), 45.6 min (major, *E*), 50.9 min (major, *Z*).

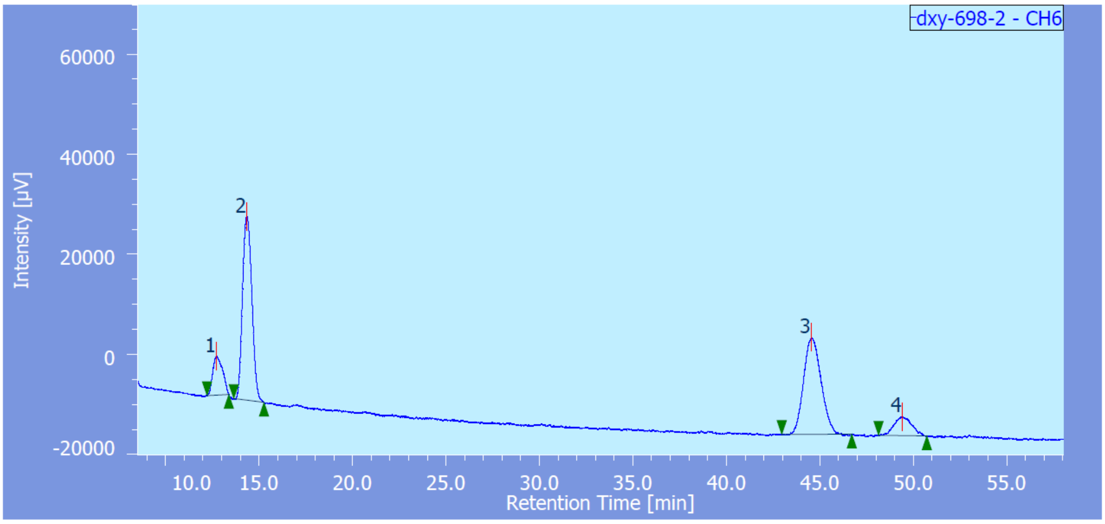

Decision

#	Peak Name	CH	tR [min]	Area [μV·sec]	Height [μV]	Area%	Height%	Quantity	NTP	Resolution	Symmetry Factor	Warning
1	Unknown	6	12.733	288155	7776	9.464	11.522	N/A	2189	1.594	1.179	
2	Unknown	6	14.340	1258606	36629	41.336	54.278	N/A	3780	22.786	1.155	
3	Unknown	6	44.540	1250073	19343	41.055	28.663	N/A	10669	2.715	1.135	
4	Unknown	6	49.397	248005	3736	8.145	5.537	N/A	11256	N/A	0.995	

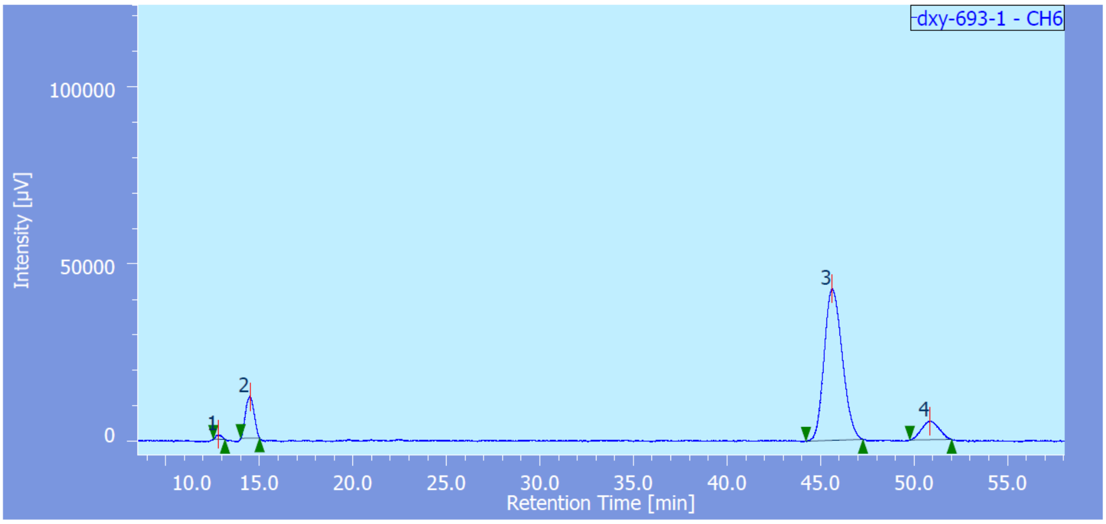

Decision

#	Peak Name	CH	tR [min]	Area [μV·sec]	Height [μV]	Area%	Height%	Quantity	NTP	Resolution	Symmetry Factor	Warning
1	Unknown	6	12.820	28405	1338	0.785	2.194	N/A	6439	2.199	1.110	
2	Unknown	6	14.497	367527	11691	10.152	19.178	N/A	4245	23.121	1.009	
3	Unknown	6	45.607	2882487	42635	79.619	69.942	N/A	10178	2.929	1.195	
4	Unknown	6	50.853	341946	5294	9.445	8.685	N/A	13003	N/A	1.030	

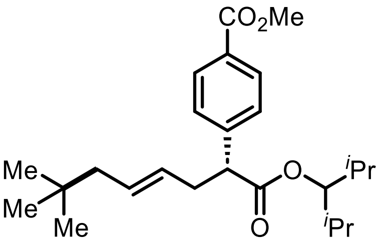

Methyl (*R*,*E*)-4-(1-((2,4-dimethylpentan-3-yl)oxy)-7,7-dimethyl-1-oxooct-4-en-2-yl)benzoate (46). Colorless oil (Hex/EA = 20/1), 66%, 95:5 er, *E*/*Z* = 8/1, $[\alpha]_D^{23}$ = -22.63 (c

= 0.17, $CHCl_3$). ^{1}H NMR (400 MHz, $CDCl_3$) δ 7.98 (d, J = 8.4 Hz, 2H), 7.40 (d, J = 8.4 Hz, 2H), 5.49 (dt, J = 15.0, 7.4 Hz, 1H), 5.26 (dt, J = 15.3, 6.9 Hz, 1H), 4.54 (t, J = 6.1 Hz, 1H), 3.90 (s, 3H), 3.67 (t, J = 7.7 Hz, 1H), 2.84 (dt, J = 14.3, 7.7 Hz, 1H), 2.53 (dt, J = 14.3, 7.2 Hz, 1H), 1.88 – 1.69 (m, 4H), 0.87 (s, 1H, *Z*), 0.80 (dd, J = 9.5, 6.6 Hz, 6H), 0.77 (s, 8H, *E*), 0.64 (dd, J = 14.0, 6.8 Hz, 6H). ^{13}C NMR (126 MHz, $CDCl_3$) δ 173.0, 167.1, 144.4, 130.9, 129.9, 129.1, 128.5, 128.4, 83.3, 52.8, 52.2, 47.1, 41.3, 36.2, 30.9, 29.6, 29.5, 29.3, 19.6, 19.6, 17.5, 16.9. IR (film): ν (cm^{-1}) 2957, 1725, 1610, 1277, 1110, 748. HR-MS (ESI) calculated [M+Na]$^+$ for $C_{25}H_{38}O_4$ $^{23}Na^+$ = 425.26623, found: 425.26644. The enantiomeric ratio of **46** was determined by HPLC analysis on Chiralpak NR-3 column. Conditions: hexane/isopropanol = 99/1, flow rate = 1.0 mL/min, uv-vis detection at λ = 220 nm, t_R = 14.4 min (*Z*), 15.1 min (major, *E*), 25.1 min (minor, *E*).

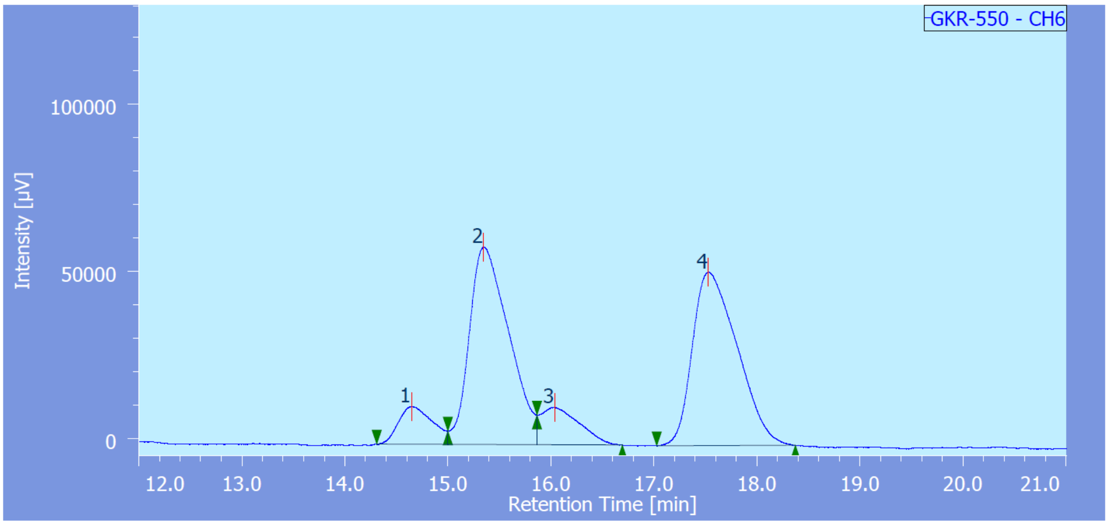

#	Peak Name	CH	tR [min]	Area [µV·sec]	Height [µV]	Area%	Height%	Quantity	NTP	Resolution	Symmetry Factor	Warning
1	Unknown	6	14.647	261452	11281	7.065	8.464	N/A	7473	0.999	N/A	
2	Unknown	6	15.347	1580355	59046	42.705	44.302	N/A	7117	N/A	N/A	
3	Unknown	6	16.037	293006	11147	7.918	8.363	N/A	N/A	N/A	N/A	
4	Unknown	6	17.527	1565837	51806	42.312	38.870	N/A	7164	N/A	1.437	

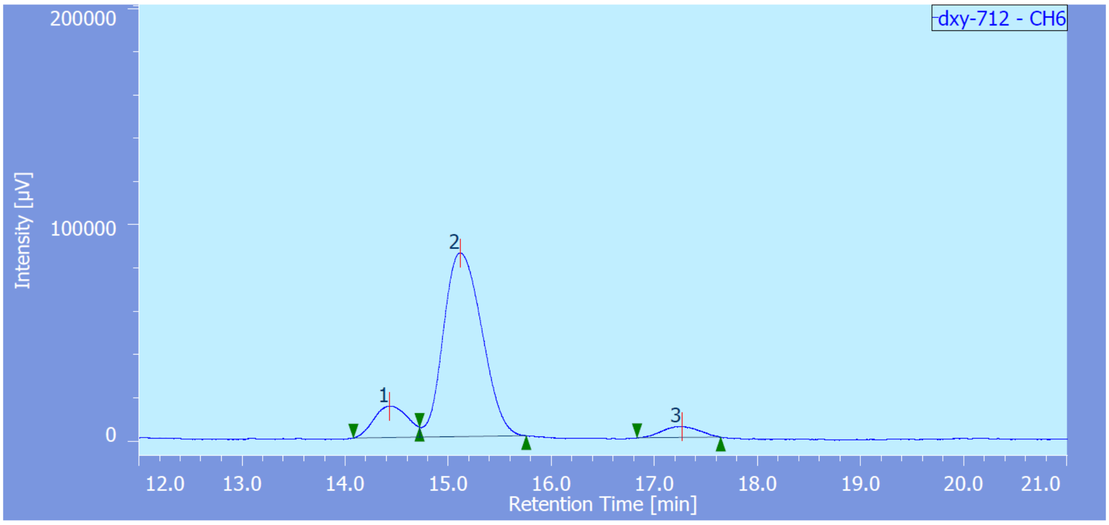

#	Peak Name	CH	tR [min]	Area [µV·sec]	Height [µV]	Area%	Height%	Quantity	NTP	Resolution	Symmetry Factor	Warning
1	Unknown	6	14.433	332551	14549	12.478	13.925	N/A	7723	1.006	N/A	
2	Unknown	6	15.117	2204645	84933	82.726	81.292	N/A	7339	3.022	N/A	
3	Unknown	6	17.267	127810	4998	4.796	4.784	N/A	9177	N/A	0.945	

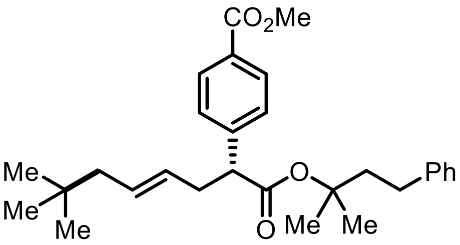

Methyl (*R,E*)-4-(7,7-dimethyl-1-((2-methyl-4-phenylbutan-2-yl)oxy)-1-oxooct-4-en-2-yl)benzoate (47). Colorless oil (Hex/EA = 20/1), 53%, 86:14 er, *E/Z* = 9/1, $[\alpha]_D^{23}$ = -14.12 (c = 0.17, $CHCl_3$). ^{1}H NMR (400 MHz, $CDCl_3$) δ 8.02 – 7.95 (m, 2H), 7.42 – 7.36 (m, 2H), 7.26 – 7.22 (m, 2H), 7.16 (t, *J* = 7.4 Hz, 1H), 7.09 – 7.04 (m, 2H), 5.49 (dt, *J* = 14.9, 7.4 Hz, 1H), 5.28 (dt, *J* = 15.1, 6.8 Hz, 1H), 3.90 (s, 3H), 3.58 (t, *J* = 7.8 Hz, 1H), 2.78 (dt, *J* = 14.6, 7.8 Hz, 1H), 2.51 – 2.38 (m, 3H), 1.98 (dd, *J* = 9.2, 7.6 Hz, 2H), 1.79 (d, *J* = 7.4 Hz, 2H), 1.43 (d, *J* = 4.9 Hz, 6H), 0.86 (s, 1H), 0.78 (s, 8H). ^{13}C NMR (126 MHz, $CDCl_3$) δ 172.0, 167.0, 144.5, 142.0, 130.6, 129.8, 129.0, 128.4, 128.4, 128.3, 128.1, 125.8, 82.7, 53.2, 52.1, 47.0, 42.8, 36.2, 30.8, 30.1, 29.2, 26.0, 25.8. IR (film): ν (cm^{-1}) 2950, 1722, 1609, 1365, 1277, 1112, 698. HR-MS (ESI) calculated $[M+Na]^+$ for $C_{29}H_{38}O_4\ {}^{23}Na^+$ = 473.26623, found: 473.26644. The enantiomeric ratio of **47** was determined by HPLC analysis on Chiralpak NR column. Conditions: hexane/isopropanol = 95/5, flow rate = 1.0 mL/min, uv-vis detection at λ = 220 nm, t_R = 12.9 min (major, *Z*), 14.0 min (major, *E*), 15.6 min (minor, *E*).

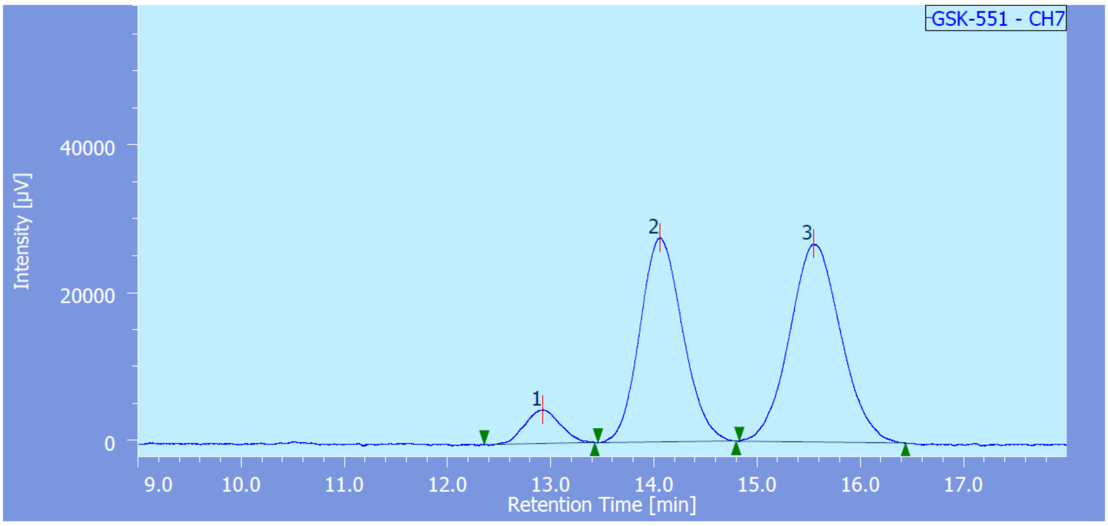

Decision

#	Peak Name	CH	tR [min]	Area [µV·sec]	Height [µV]	Area%	Height%	Quantity	NTP	Resolution	Symmetry Factor	Warning
1	Unknown	7	12.923	108348	4544	5.933	7.716	N/A	6354	1.614	1.029	
2	Unknown	7	14.057	792751	27592	43.407	46.857	N/A	5478	1.788	1.129	
3	Unknown	7	15.543	925211	26750	50.660	45.427	N/A	4694	N/A	1.085	

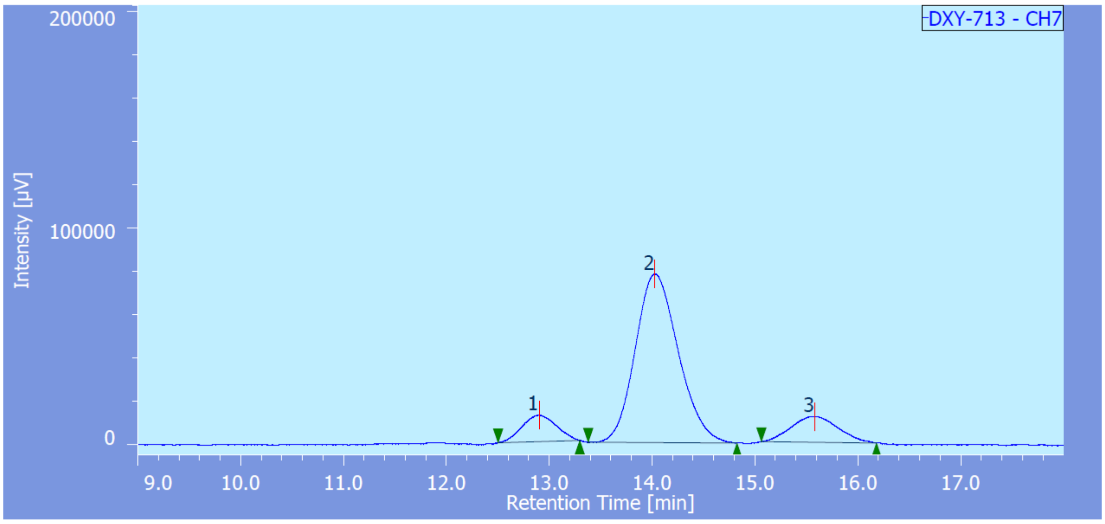

Decision

#	Peak Name	CH	tR [min]	Area [µV·sec]	Height [µV]	Area%	Height%	Quantity	NTP	Resolution	Symmetry Factor	Warning
1	Unknown	7	12.900	284193	12220	9.701	11.981	N/A	6470	1.607	1.017	
2	Unknown	7	14.027	2265183	77847	77.322	76.327	N/A	5382	1.918	1.160	
3	Unknown	7	15.583	380160	11925	12.977	11.692	N/A	5222	N/A	1.063	

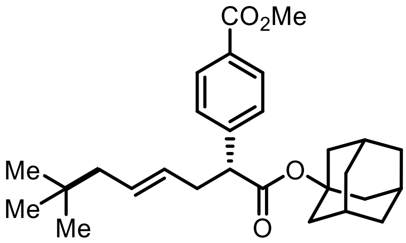

Methyl 4-((*R*,*E*)-1-(((3*S*,5*S*,7*S*)-adamantan-1-yl)oxy)-7,7-dimethyl-1-oxooct-4-en-2-yl)benzoate (48). Colorless oil (Hex/EA = 20/1), 57%, 90:10 er, *E/Z* = 8/1, $[\alpha]_D^{23}$ = -13.75 (c

= 0.20, $CHCl_3$). ^{1}H NMR (400 MHz, $CDCl_3$) δ 8.02 – 7.91 (m, 2H), 7.41 – 7.30 (m, 2H), 5.52 – 5.43 (m, 1H), 5.32 – 5.21 (m, 1H), 3.90 (s, 3H), 3.54 (t, J = 7.7 Hz, 1H), 2.74 (dt, J = 14.8, 7.4 Hz, 1H), 2.47 – 2.37 (m, 1H), 2.09 (s, 3H), 2.02 (d, J = 3.5 Hz, 6H), 1.80 (d, J = 7.5 Hz, 2H), 1.62 (d, J = 3.2 Hz, 6H), 0.86 (s, 1H, *Z*), 0.78 (s, 7H, *E*). ^{13}C NMR (101 MHz, $CDCl_3$) δ 172.0, 167.2, 144.9, 130.6, 129.9, 129.0, 128.6, 128.2, 81.2, 53.4, 52.2, 47.2, 41.5, 41.3, 36.6, 36.3, 30.9, 29.3. IR (film): ν (cm^{-1}) 2910, 2853, 1723, 1434, 1276, 1056, 968, 704. HR-MS (ESI) calculated $[M+Na]^+$ for $C_{28}H_{38}O_4$ $^{23}Na^+$ = 461.26623, found: 461.26635. The enantiomeric ratio of **48** was determined by HPLC analysis on Chiralpak NR-3 column. Conditions: hexane/isopropanol = 99/1, flow rate = 1.0 mL/min, uv-vis detection at λ = 254 nm, t_R = 33.0 min (*Z*), 39.7 min (major, *E*), 43.0 min (minor, *E*).

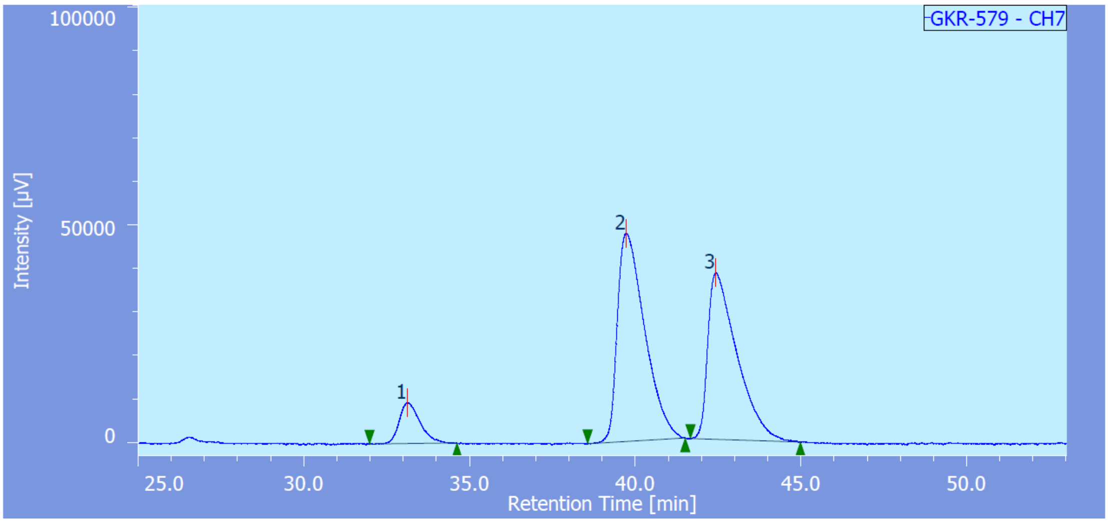

Decision

#	Peak Name	CH	tR [min]	Area [µV·sec]	Height [µV]	Area%	Height%	Quantity	NTP	Resolution	Symmetry Factor	Warning
1	Unknown	7	33.127	411682	9360	7.475	9.812	N/A	13834	5.017	1.397	
2	Unknown	7	39.733	2745416	47761	49.851	50.063	N/A	11017	1.748	1.756	
3	Unknown	7	42.427	2350153	38280	42.674	40.125	N/A	11591	N/A	2.164	

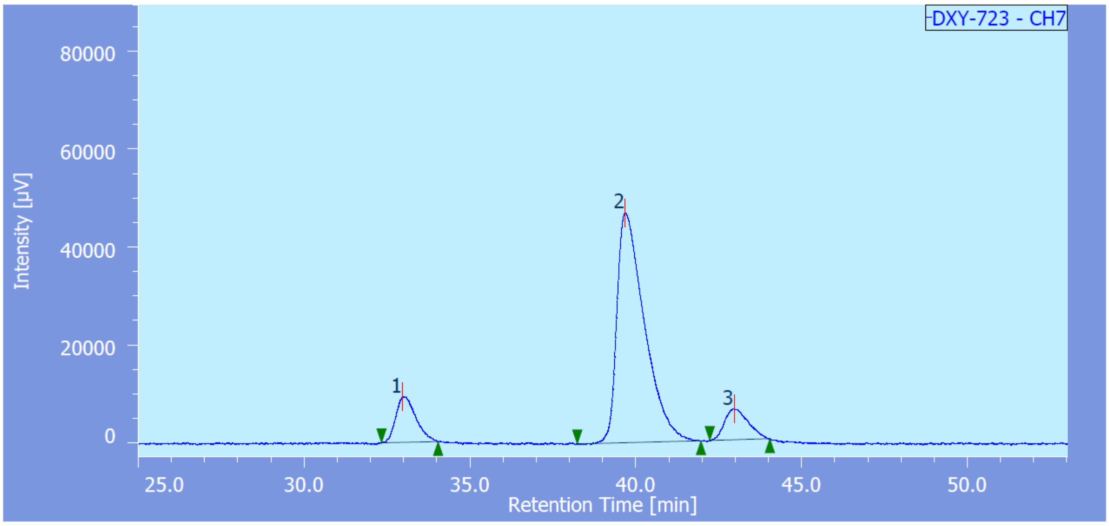

Decision

#	Peak Name	CH	tR [min]	Area [μV·sec]	Height [μV]	Area%	Height%	Quantity	NTP	Resolution	Symmetry Factor	Warning
1	Unknown	7	32.977	391865	9325	11.469	14.896	N/A	14469	5.210	1.421	
2	Unknown	7	39.673	2708650	46957	79.279	75.010	N/A	11480	2.338	1.943	
3	Unknown	7	42.963	316072	6319	9.251	10.094	N/A	16441	N/A	1.281	

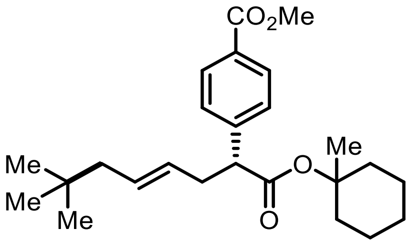

Methyl (*R*,*E*)-4-(7,7-dimethyl-1-((1-methylcyclohexyl)oxy)-1-oxooct-4-en-2-yl)benzoate (49). Colorless oil (Hex/EA = 20/1), 43%, 92:8 er, *E/Z* = 8/1, $[\alpha]_D^{23}$ = -22.47 (c = 0.17, $CHCl_3$). ^{1}H NMR (400 MHz, $CDCl_3$) δ 8.00 – 7.94 (m, 2H), 7.39 – 7.32 (m, 2H), 5.52 – 5.39 (m, 1H), 5.30 – 5.20 (m, 1H), 3.90 (s, 3H), 3.54 (t, *J* = 7.7 Hz, 1H), 2.74 (dddd, *J* = 14.0, 8.0, 6.9, 1.2 Hz, 1H), 2.49 – 2.37 (m, 1H), 1.83 (d, *J* = 7.0 Hz, 2H), 1.45 – 1.42 (m, 3H), 1.38 (s, 9H), 1.34 – 1.17 (m, 3H), 1.15 – 1.07 (m, 3H), 0.74 (s, 3H). ^{13}C NMR (101 MHz, $CDCl_3$) δ 172.3, 167.1, 144.7, 130.0, 129.9, 129.0, 128.6, 128.2, 81.1, 53.3, 52.2, 37.7, 37.6, 36.7, 33.3, 28.1, 26.5, 25.1, 22.1. IR (film): ν (cm^{-1}) 2933, 2863, 1722, 1610, 1277, 1179, 1144, 964, 528. HR-MS (ESI) calculated $[M+Na]^+$ for $C_{25}H_{36}O_4$ $^{23}Na^+$ = 423.25058, found: 423.25069. The enantiomeric ratio of **49** was determined by HPLC analysis on Chiralpak NR column. Conditions: hexane/isopropanol = 97/3, flow rate = 1.0 mL/min, uv-vis detection at λ = 254 nm, t_R = 12.0 min (major, *Z*), 13.1 min (major, *E*), 15.7 min (minor, *Z*), 17.1 min (minor, *E*).

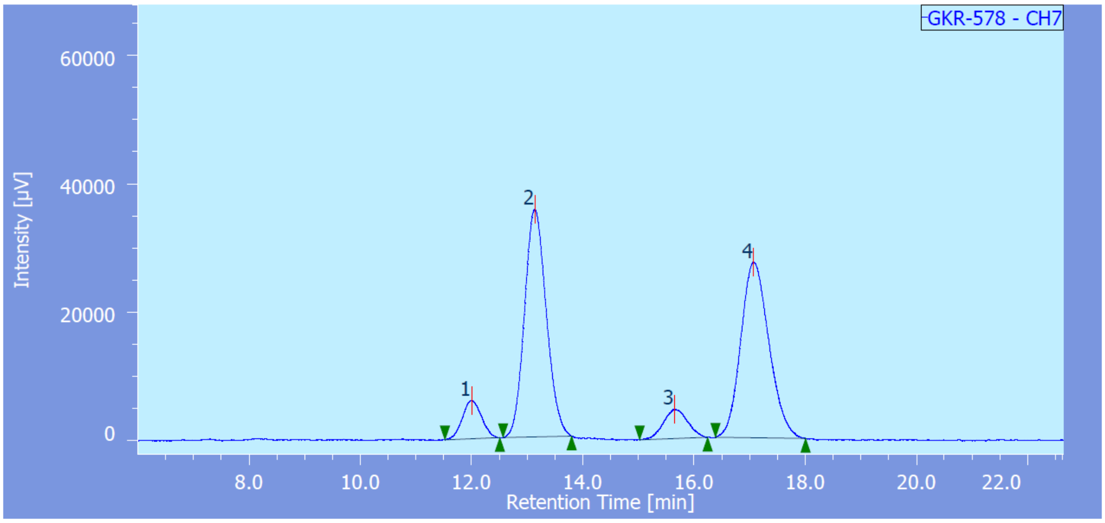

Decision

#	Peak Name	CH	tR [min]	Area [µV·sec]	Height [µV]	Area%	Height%	Quantity	NTP	Resolution	Symmetry Factor	Warning
1	Unknown	7	12.003	142734	5972	6.451	8.144	N/A	5605	1.667	1.027	
2	Unknown	7	13.133	965092	35440	43.619	48.333	N/A	5354	3.268	1.124	
3	Unknown	7	15.647	138031	4557	6.238	6.215	N/A	5766	1.623	1.036	
4	Unknown	7	17.067	966707	27356	43.692	37.308	N/A	5385	N/A	1.161	

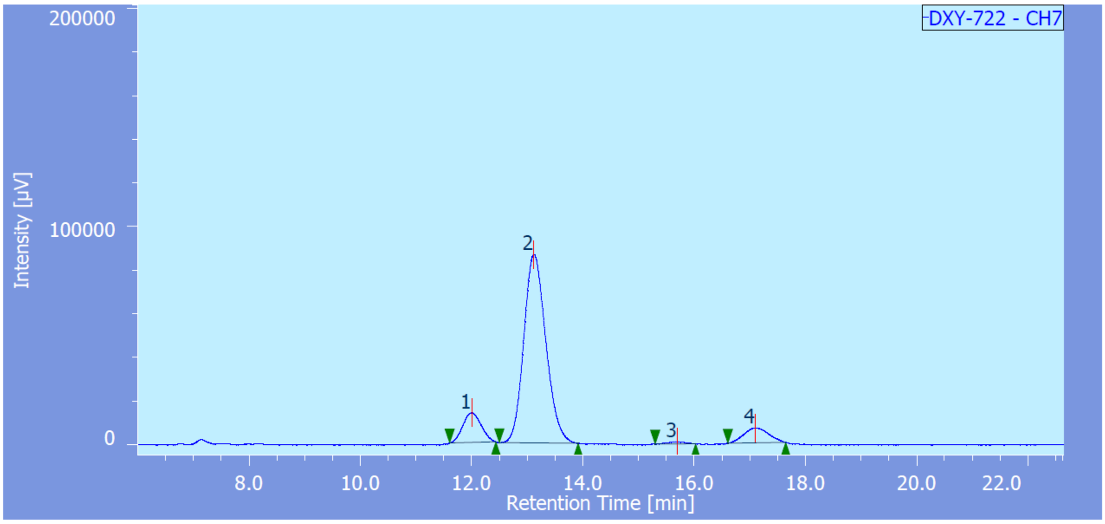

Decision

#	Peak Name	CH	tR [min]	Area [µV·sec]	Height [µV]	Area%	Height%	Quantity	NTP	Resolution	Symmetry Factor	Warning
1	Unknown	7	12.007	313754	13619	10.727	12.635	N/A	5871	1.652	1.042	
2	Unknown	7	13.117	2377209	86409	81.271	80.163	N/A	5295	3.685	1.165	
3	Unknown	7	15.697	21278	910	0.727	0.844	N/A	8450	1.824	0.906	
4	Unknown	7	17.097	212794	6854	7.275	6.358	N/A	6378	N/A	1.082	

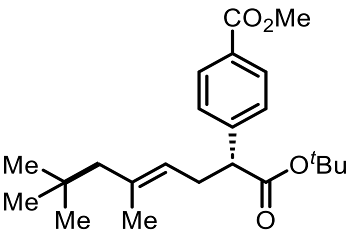

***tert*-Butyl (*R*,*E*)-2-(4-cyanophenyl)-7,7-dimethyloct-4-enoate (50)**. Colorless oil (Hex/EA =

20/1), 56%, 93:7 er, *E/Z* = 6/1, $[\alpha]_D^{24}$ = -16.63 (c = 0.18, $CHCl_3$). ^{1}H NMR (500 MHz, $CDCl_3$) δ 7.97 (d, *J* = 8.4 Hz, 2H), 7.38 (d, *J* = 8.4 Hz, 2H), 4.99 (td, *J* = 7.3, 1.3 Hz, 1H), 3.90 (s, 3H), 3.53 (t, *J* = 7.7 Hz, 1H), 2.74 (dt, *J* = 14.8, 7.5 Hz, 1H), 2.44 (dt, *J* = 14.5, 7.3 Hz, 1H), 1.83 (d, *J* = 2.4 Hz, 1H), 1.62 (d, *J* = 1.3 Hz, 3H), 1.38 (s, 9H), 0.91 (s, 1H, *Z*), 0.81 (s, 8H, *E*). ^{1}H NMR (500 MHz, $CDCl_3$) δ 7.97 (d, *J* = 8.4 Hz, 2H), 7.38 (d, *J* = 8.4 Hz, 2H), 4.99 (td, *J* = 7.3, 1.3 Hz, 1H), 3.90 (s, 3H), 3.53 (t, *J* = 7.7 Hz, 1H), 2.74 (dt, *J* = 14.8, 7.5 Hz, 1H), 2.44 (dt, *J* = 14.5, 7.3 Hz, 1H), 1.83 (d, *J* = 2.4 Hz, 2H), 1.62 (d, *J* = 1.3 Hz, 3H), 1.38 (s, 9H), 0.91 (s, 1H, *Z*), 0.81 (s, 8H, *E*). ^{13}C NMR (126 MHz, $CDCl_3$) δ 172.5, 167.1, 145.0, 136.1, 129.8, 129.0, 128.1, 124.6, 124.3, 81.0, 53.6, 53.2, 52.2, 32.4, 31.7, 30.7, 30.1, 28.1, 19.2. IR (film): ν (cm^{-1}) 2930, 1727, 1684, 1447, 1367, 1280, 1149, 769, 735. HR-MS (ESI) calculated $[M+Na]^+$ for $C_{23}H_{34}O_4{}^{23}Na^+$ = 397.23493, found: 397.23470. The enantiomeric ratio of **50** was determined by HPLC analysis on Chiralpak NR column. Conditions: hexane/isopropanol = 99/1, flow rate = 1.0 mL/min, uv-vis detection at λ = 220 nm, t_R = 20.2 min (major, *E*), 22.1 min (minor, *E*).

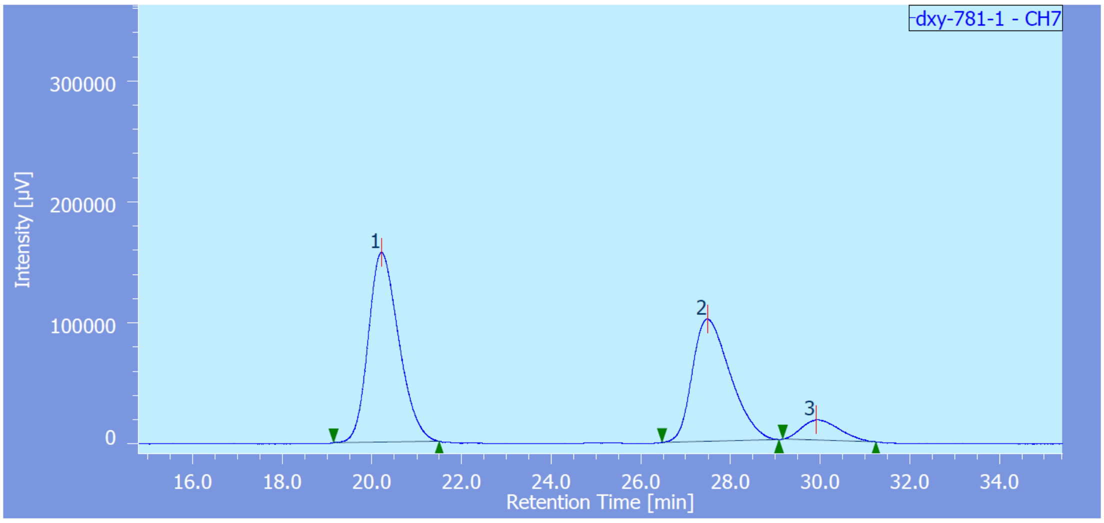

#	Peak Name	CH	tR [min]	Area [µV·sec]	Height [µV]	Area%	Height%	Quantity	NTP	Resolution	Symmetry Factor	Warning
1	Unknown	7	20.213	7411027	156622	51.702	57.138	N/A	4261	5.201	1.236	
2	Unknown	7	27.483	5942803	100922	41.459	36.818	N/A	4937	1.519	1.369	
3	Unknown	7	29.897	980367	16569	6.839	6.045	N/A	5434	N/A	1.383	

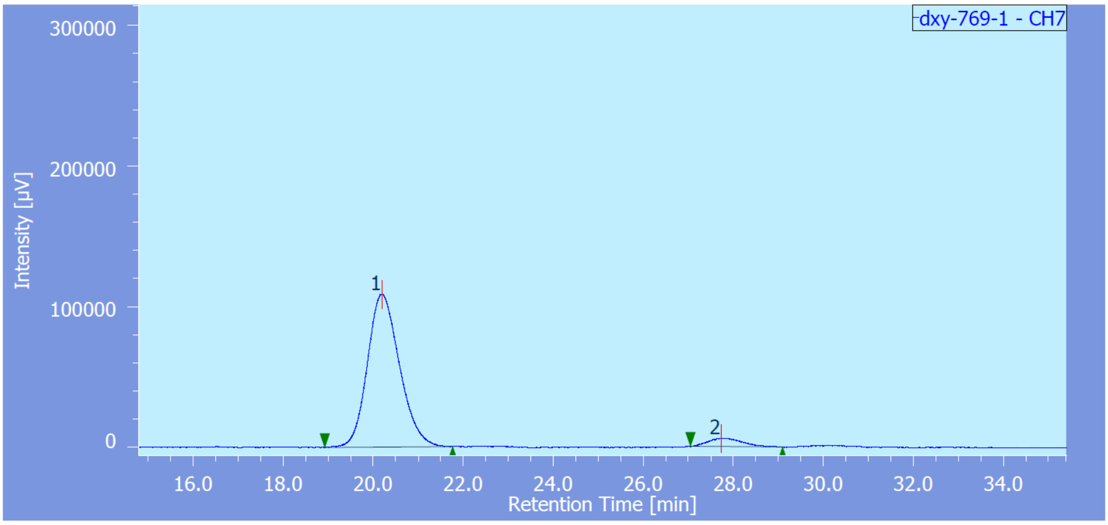

#	Peak Name	CH	tR [min]	Area [µV·sec]	Height [µV]	Area%	Height%	Quantity	NTP	Resolution	Symmetry Factor	Warning
1	Unknown	7	20.190	5383347	108604	94.449	94.920	N/A	3921	5.468	1.183	
2	Unknown	7	27.723	316403	5812	5.551	5.080	N/A	5667	N/A	1.403	

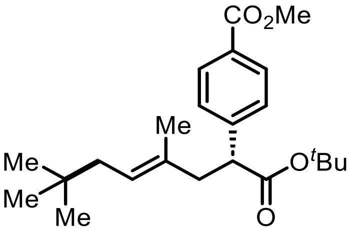

Methyl (*R,E*)-4-(1-(*tert*-butoxy)-4,7,7-trimethyl-1-oxooct-4-en-2-yl)benzoate (51). Colorless oil (Hex/EA = 20/1), 43%, 94:6 er, *E/Z* = 6/1, $[\alpha]_D^{23}$ = -8.91 (c = 0.18, $CHCl_3$). ^1H NMR (400 MHz, $CDCl_3$) δ 7.96 (dd, *J* = 8.5, 2.1 Hz, 2H), 7.37 (d, *J* = 8.4 Hz, 2H), 5.19 (ddd, *J* = 8.4, 7.2, 1.3 Hz, 1H), 3.90 (s, 3H), 3.72 (t, *J* = 7.8 Hz, 1H), 2.78 (dd, *J* = 13.7, 8.3 Hz, 1H), 2.42 (dd, *J* = 13.8, 7.6 Hz, 1H), 1.78 (dd, *J* = 11.2, 7.6 Hz, 2H), 1.57 (d, *J* = 1.1 Hz, 3H), 1.37 (s, 9H), 0.82 (s, 1H, *Z*), 0.74 (s, 8H, *E*). ^{13}C NMR (101 MHz, $CDCl_3$) δ 172.4, 167.2, 132.6, 129.8, 128.9, 128.2, 125.4, 125.1, 81.0, 52.2, 51.5, 43.7, 42.0, 31.7, 29.3, 28.1, 16.3. IR (film): ν (cm^{-1}) 2952, 1718, 1608, 1435, 1365, 1275, 1146, 759, 702. HR-MS (ESI) calculated $[M+Na]^+$ for $C_{23}H_{34}O_4{}^{23}Na^+$ = 397.23493, found: 397.23527. The enantiomeric ratio of **51** was determined by HPLC analysis on Chiralpak NR column. Conditions: hexane/isopropanol = 99/1, flow rate = 1.0 mL/min, uv-vis detection at λ = 254 nm, t_R = 17.1 min (*Z*), 19.4 min (minor, *E*), 25.9 min (major, *E*).

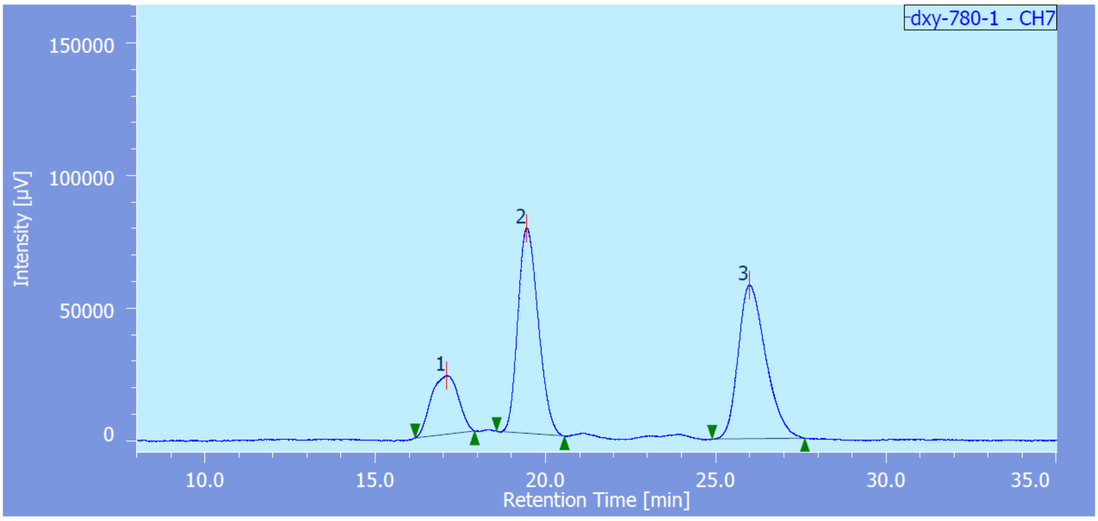

Decision

#	Peak Name	CH	tR [min]	Area [µV·sec]	Height [µV]	Area%	Height%	Quantity	NTP	Resolution	Symmetry Factor	Warning
1	Unknown	7	17.100	1228783	22117	15.942	14.041	N/A	1874	1.758	0.944	
2	Unknown	7	19.453	3207845	77301	41.618	49.075	N/A	4968	5.091	1.218	
3	Unknown	7	25.990	3271130	58097	42.439	36.884	N/A	4994	N/A	1.318	

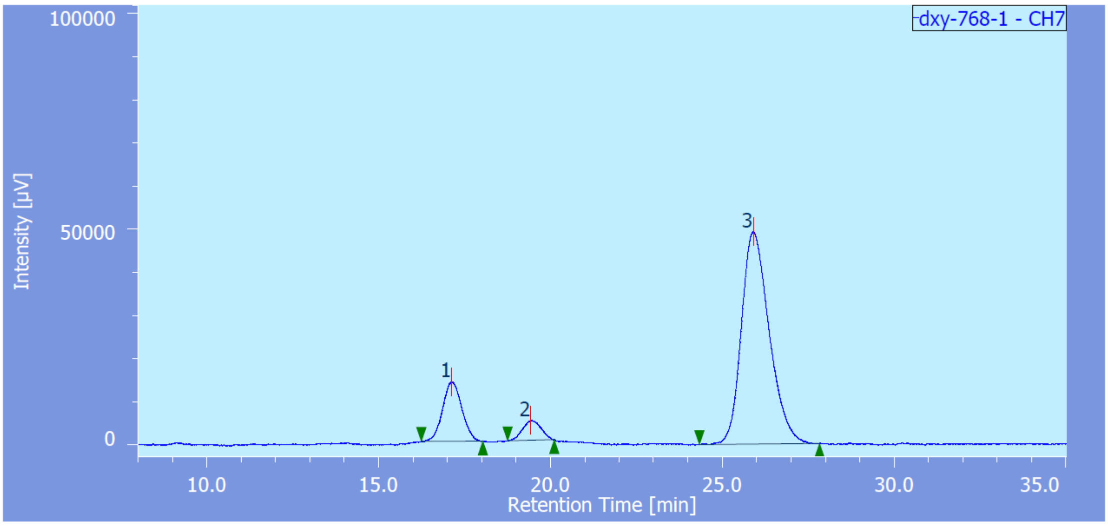

Decision

#	Peak Name	CH	tR [min]	Area [µV·sec]	Height [µV]	Area%	Height%	Quantity	NTP	Resolution	Symmetry Factor	Warning
1	Unknown	7	17.133	535971	13759	15.397	20.379	N/A	4445	2.233	1.031	
2	Unknown	7	19.433	171507	4577	4.927	6.780	N/A	5613	5.197	1.046	
3	Unknown	7	25.900	2773593	49178	79.676	72.841	N/A	5051	N/A	1.260	

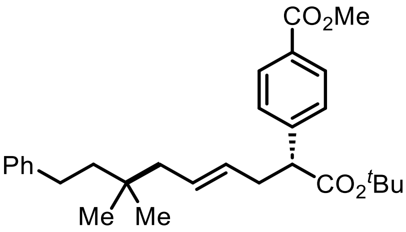

Methyl *(R,E)*-4-(1-(*tert*-butoxy)-7,7-dimethyl-1-oxo-9-phenylnon-4-en-2-yl)benzoate (52). Colorless oil (Hex/EA = 20/1), 42%, 91:9 er, *E/Z* = 9/1, $[\alpha]_D^{24}$ = -13.70 (c = 0.17, $CHCl_3$).

^{1}H NMR (400 MHz, $CDCl_3$) δ 7.95 (d, J = 8.4 Hz, 2H), 7.34 (d, J = 8.4 Hz, 2H), 7.29 – 7.23 (m, 2H), 7.18 – 7.10 (m, 3H), 5.48 (dt, J = 15.0, 7.4 Hz, 1H), 5.34 – 5.26 (m, 1H), 3.89 (s, 3H), 3.54 (t, J = 7.7 Hz, 1H), 2.75 (dt, J = 15.0, 7.4 Hz, 1H), 2.53 – 2.47 (m, 2H), 2.46 – 2.39 (m, 1H), 1.88 (d, J = 7.5 Hz, 2H), 1.41 (d, J = 4.3 Hz, 2H), 1.36 (s, 9H), 0.90 (s, 1H, *Z*), 0.83 (s, 5H, *E*). ^{13}C NMR (101 MHz, $CDCl_3$) δ 172.2, 167.1, 144.6, 143.5, 130.0, 129.9, 129.0, 128.9, 128.4, 128.2, 125.7, 81.1, 53.2, 52.2, 45.1, 44.2, 36.5, 33.5, 30.8, 28.1, 27.0, 27.0. IR (film): ν (cm^{-1}) 2953, 1723, 1610, 1366, 1277, 1144, 699. HR-MS (ESI) calculated $[M+Na]^+$ for $C_{29}H_{38}O_4\,^{23}Na^+$ = 473.26623, found: 473.26678. The enantiomeric ratio of **52** was determined by HPLC analysis on Chiralpak NR column. Conditions: hexane/isopropanol = 97/3, flow rate = 1.0 mL/min, uv-vis detection at λ = 254 nm, t_R = 17.4 min (*Z*), 21.0 min (major, *E*), 23,6 min (minor, *E*).

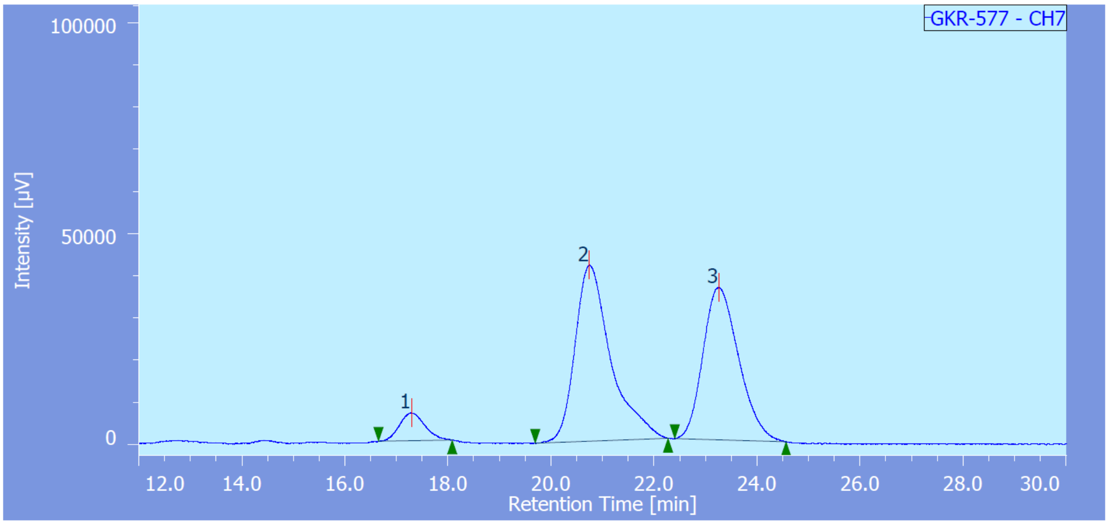

#	Peak Name	CH	tR [min]	Area [µV·sec]	Height [µV]	Area%	Height%	Quantity	NTP	Resolution	Symmetry Factor	Warning
1	Unknown	7	17.290	226804	6588	5.684	7.800	N/A	5810	3.356	1.169	
2	Unknown	7	20.747	2016803	41745	50.541	49.427	N/A	5133	2.068	1.497	
3	Unknown	7	23.257	1746809	36125	43.775	42.773	N/A	5317	N/A	1.225	

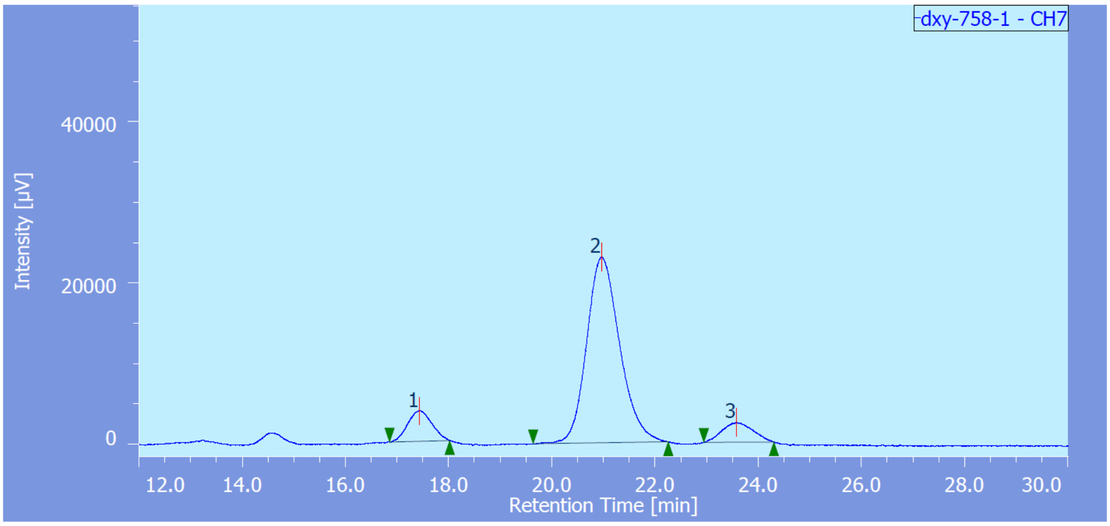

#	Peak Name	CH	tR [min]	Area [µV·sec]	Height [µV]	Area%	Height%	Quantity	NTP	Resolution	Symmetry Factor	Warning
1	Unknown	7	17.437	123202	3760	9.865	12.879	N/A	6096	3.469	1.042	
2	Unknown	7	20.963	1024100	23017	82.000	78.830	N/A	5361	2.259	1.191	
3	Unknown	7	23.577	101595	2421	8.135	8.291	N/A	6442	N/A	1.090	

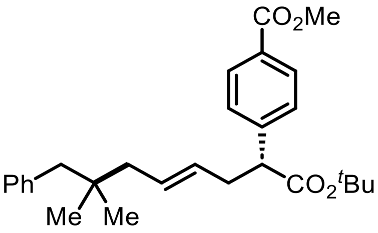

Methyl (*R*,*E*)-4-(1-(*tert*-butoxy)-7,7-dimethyl-1-oxo-8-phenyloct-4-en-2-yl)benzoate (53). Colorless oil (Hex/EA = 20/1), 31%, 90:10 er, *E/Z* = 9/1, $[\alpha]_D^{24}$ = -17.27 (c = 0.19, $CHCl_3$). ^{1}H NMR (400 MHz, $CDCl_3$) δ 7.98 (d, *J* = 8.2 Hz, 2H), 7.38 (d, *J* = 8.4 Hz, 2H), 7.27 – 7.22 (m, 2H), 7.19 (d, *J* = 7.2 Hz, 1H), 7.10 – 7.02 (m, 2H), 5.53 (dt, *J* = 15.0, 7.5 Hz, 1H), 5.31 (dt, *J* = 15.1, 6.9 Hz, 1H), 3.90 (s, 3H), 3.57 (t, *J* = 7.7 Hz, 1H), 2.78 (dt, *J* = 14.5, 7.6 Hz, 1H), 2.54 – 2.44 (m, 1H), 2.38 (s, 2H), 1.88 – 1.82 (m, 2H), 1.38 (s, 9H), 0.82 (d, *J* = 4.7 Hz, 1H), 0.74 (d, *J* = 2.3 Hz, 5H). ^{13}C NMR (101 MHz, $CDCl_3$) δ 172.2, 167.1, 144.7, 139.4, 130.7, 130.1, 129.9, 129.2, 129.1, 128.2, 127.7, 125.9, 53.2, 52.2, 48.3, 45.5, 36.6, 34.7, 28.1, 26.7, 26.6. IR (film): ν (cm^{-1}) 2954, 1722, 1610, 1366, 1277, 1143, 701. HR-MS (ESI) calculated $[M+Na]^+$ for $C_{28}H_{36}O_4{}^{23}Na^+$ = 459.25058, found: 459.24893. The enantiomeric ratio of **53** was determined by HPLC analysis on Chiralpak NR column. Conditions: hexane/isopropanol = 97/3, flow rate = 1.0 mL/min, uv-vis detection at λ = 254 nm, t_R = 17.1 min (major, *Z*), 21.3 min (major, *E*), 24.5 min (minor, *E*).

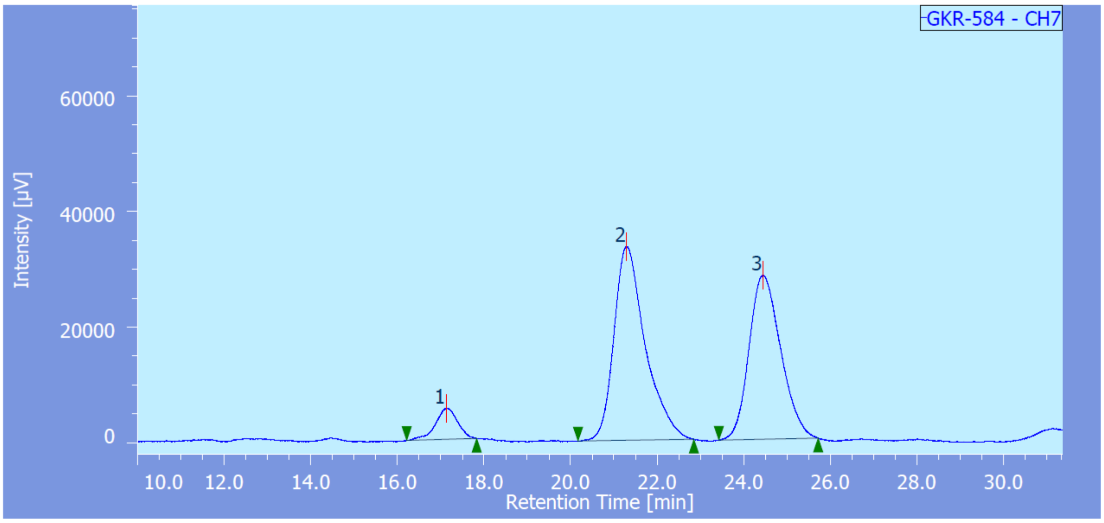

Decision

#	Peak Name	CH	tR [min]	Area [µV·sec]	Height [µV]	Area%	Height%	Quantity	NTP	Resolution	Symmetry Factor	Warning
1	Unknown	7	17.127	191904	5397	5.776	8.024	N/A	5736	3.885	0.897	
2	Unknown	7	21.290	1685670	33505	50.733	49.815	N/A	4682	2.438	1.408	
3	Unknown	7	24.440	1445055	28358	43.491	42.162	N/A	5270	N/A	1.201	

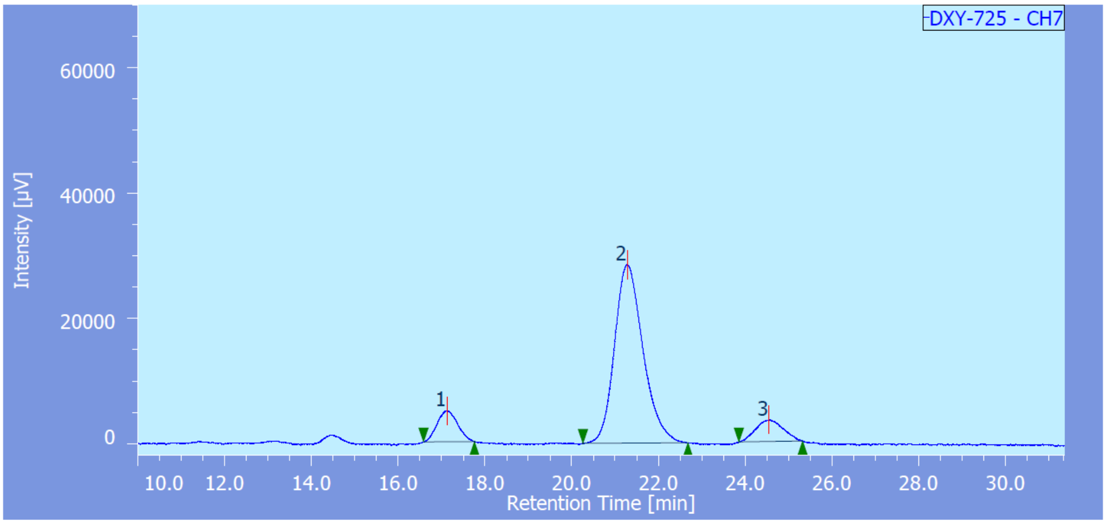

Decision

#	Peak Name	CH	tR [min]	Area [µV·sec]	Height [µV]	Area%	Height%	Quantity	NTP	Resolution	Symmetry Factor	Warning
1	Unknown	7	17.123	163098	4955	10.075	13.447	N/A	5985	4.029	1.088	
2	Unknown	7	21.273	1303855	28453	80.541	77.219	N/A	5197	2.719	1.231	
3	Unknown	7	24.543	151915	3439	9.384	9.334	N/A	6361	N/A	1.056	

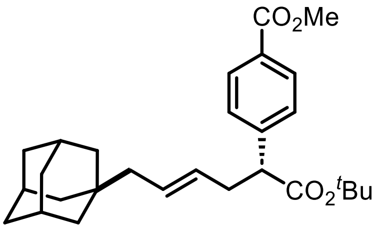

Methyl 4-((*R*,*E*)-6-((3*R*,5*R*,7*R*)-adamantan-1-yl)-1-(*tert*-butoxy)-1-oxohex-4-en-2-yl)benzoate (54). Colorless oil (Hex/EA = 20/1), 57%, 91:9 er, *E*/*Z* = 7/1, $[\alpha]_D^{24}$ = -21.61 (c

= 0.17, $CHCl_3$). ^1H NMR (400 MHz, $CDCl_3$) δ 7.98 (d, J = 8.4 Hz, 2H), 7.36 (d, J = 8.4 Hz, 2H), 5.47 – 5.37 (m, 1H), 5.21 (dt, J = 15.1, 6.9 Hz, 1H), 3.90 (s, 3H), 3.55 (t, J = 7.8 Hz, 1H), 2.79 – 2.67 (m, 1H), 2.45 (dt, J = 14.3, 7.0 Hz, 1H), 1.93 – 1.82 (m, 3H), 1.67 – 1.50 (m, 9H), 1.44 (d, J = 1.1 Hz, 1H), 1.38 (s, 8H), 1.32 – 1.26 (m, 5H). ^{13}C NMR (101 MHz, $CDCl_3$) δ 171.8, 144.8, 132.4, 131.1, 129.0, 128.1, 119.0, 111.1, 81.5, 53.3, 47.1, 36.6, 30.9, 29.3, 28.1. ^{13}C NMR (101 MHz, $CDCl_3$) δ 172.3, 167.1, 144.7, 129.9, 129.4, 129.0, 128.5, 128.3, 81.1, 53.2, 52.2, 47.7, 42.4, 37.2, 36.6, 32.8, 28.8, 28.1. IR (film): ν (cm^{-1}) 2900, 1723, 1367, 1276, 1143, 1109, 704. HR-MS (ESI) calculated $[M+Na]^+$ for $C_{28}H_{38}O_4\,^{23}Na^+$ = 461.26623, found: 461.26692.The enantiomeric ratio of **54** was determined by HPLC analysis on Chiralpak NR column. Conditions: hexane/isopropanol = 98/2, flow rate = 0.5 mL/min, uv-vis detection at λ = 254 nm, t_R = 32.4 min (major, *Z*), 41.5 min (minor, *Z*), 45.9 min (major, *E*), 49.1 min (minor, *E*).

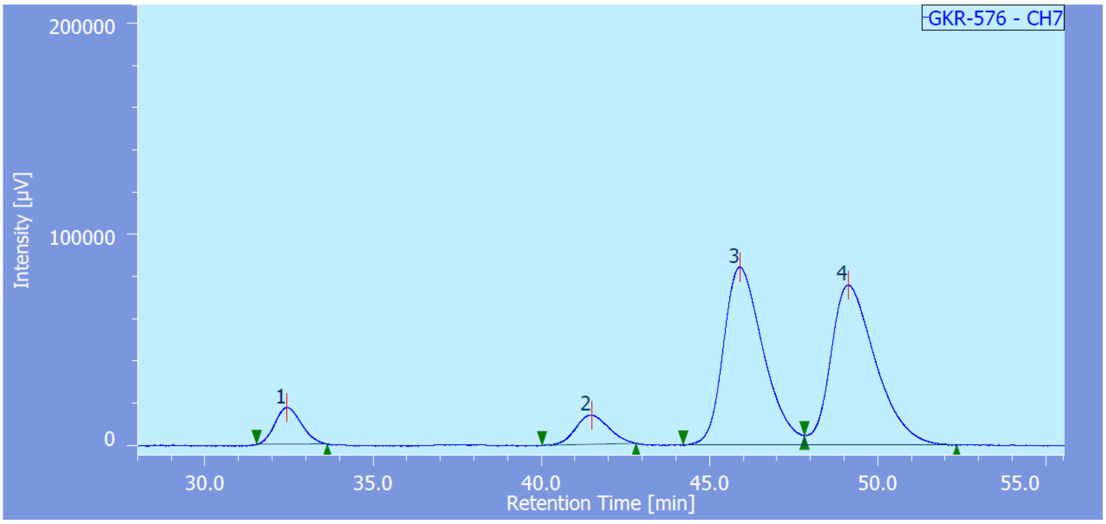

Decision

#	Peak Name	CH	tR [min]	Area [µV·sec]	Height [µV]	Area%	Height%	Quantity	NTP	Resolution	Symmetry Factor	Warning
1	Unknown	7	32.437	952115	17416	6.015	9.104	N/A	7798	5.536	1.158	
2	Unknown	7	41.500	947785	13961	5.987	7.298	N/A	8375	2.233	1.070	
3	Unknown	7	45.903	6917924	84118	43.701	43.973	N/A	7357	1.423	N/A	
4	Unknown	7	49.113	7012314	75798	44.297	39.624	N/A	6804	N/A	N/A	

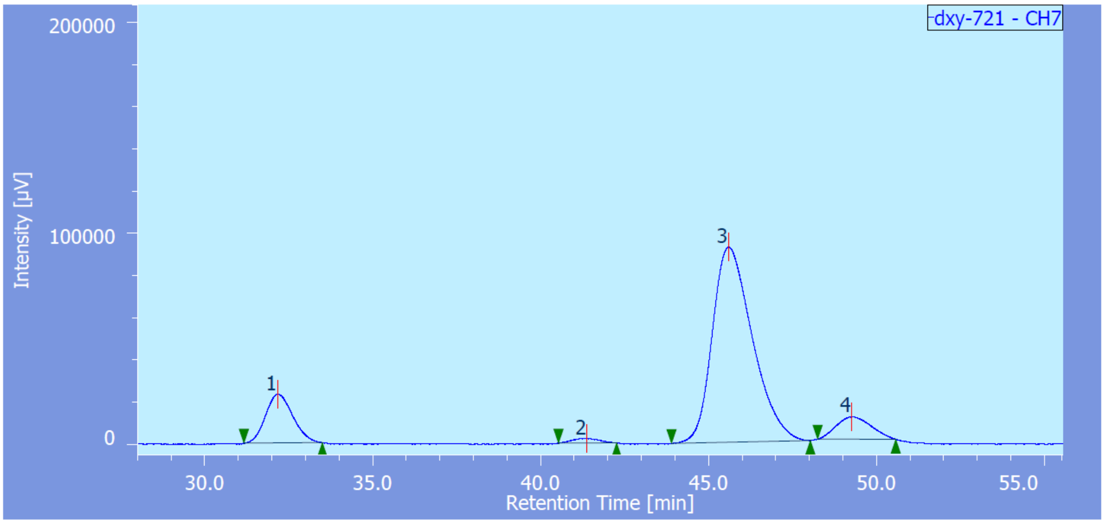

Decision

#	Peak Name	CH	tR [min]	Area [µV·sec]	Height [µV]	Area%	Height%	Quantity	NTP	Resolution	Symmetry Factor	Warning
1	Unknown	7	32.170	1306097	23162	13.241	18.051	N/A	7380	5.978	1.164	
2	Unknown	7	41.377	118892	2131	1.205	1.661	N/A	10829	2.247	0.981	
3	Unknown	7	45.590	7668190	92454	77.738	72.053	N/A	7064	1.744	1.338	
4	Unknown	7	49.250	770928	10568	7.815	8.236	N/A	9328	N/A	1.159	

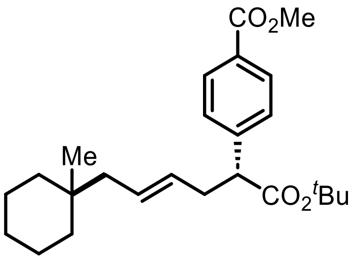

Methyl (*R*,*E*)-4-(1-(*tert*-butoxy)-6-(1-methylcyclohexyl)-1-oxohex-4-en-2-yl)benzoate (55). Colorless oil (Hex/EA = 20/1), 40%, 93:7 er, *E/Z* = 9/1, $[\alpha]_D^{23}$ = -16.92 (c = 0.16, $CHCl_3$). ^{1}H NMR (400 MHz, $CDCl_3$) δ 8.00 – 7.94 (m, 2H), 7.39 – 7.32 (m, 2H), 5.52 – 5.39 (m, 1H), 5.30 – 5.20 (m, 1H), 3.90 (s, 3H), 3.54 (t, *J* = 7.7 Hz, 1H), 2.74 (dddd, *J* = 14.0, 8.0, 6.9, 1.2 Hz, 1H), 2.49 – 2.37 (m, 1H), 2.30 – 2.20 (m, 1H), 1.83 (d, *J* = 7.0 Hz, 2H), 1.45 – 1.42 (m, 3H), 1.38 (s, 9H), 1.34 – 1.17 (m, 3H), 1.15 – 1.07 (m, 3H), 0.74 (s, 3H). ^{13}C NMR (101 MHz, $CDCl_3$) δ 172.27, 167.15, 144.74, 129.99, 129.89, 128.99, 128.60, 128.20, 81.11, 53.28, 52.20, 37.65, 37.59, 36.66, 33.26, 28.08, 26.53, 25.14, 22.12. IR (film): ν (cm^{-1}) 2926, 2360, 1724, 1276, 1145, 1110, 756. HR-MS (ESI) calculated $[M+Na]^+$ for $C_{25}H_{36}O_4\,^{23}Na^+$ = 423.25058, found: 423.25057. The enantiomeric ratio of **55** was determined by HPLC analysis on Chiralpak NR column. Conditions: hexane/isopropanol = 97/3, flow rate = 1.0 mL/min, uv-vis detection at λ = 254 nm, t_R = 12.6 min (major, *Z*), 15.4 min (major, *E*), 17.6 min (minor, *E*).

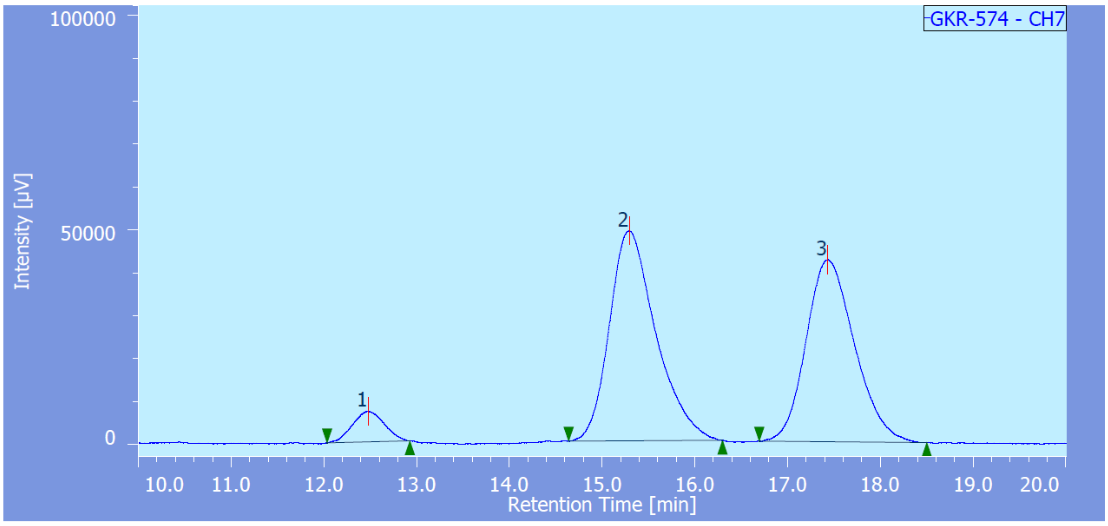

Decision

#	Peak Name	CH	tR [min]	Area [µV·sec]	Height [µV]	Area%	Height%	Quantity	NTP	Resolution	Symmetry Factor	Warning
1	Unknown	7	12.477	170580	7115	4.991	7.231	N/A	5882	3.671	1.008	
2	Unknown	7	15.293	1706140	48893	49.916	49.687	N/A	4748	2.329	1.306	
3	Unknown	7	17.430	1541280	42395	45.093	43.083	N/A	5368	N/A	1.195	

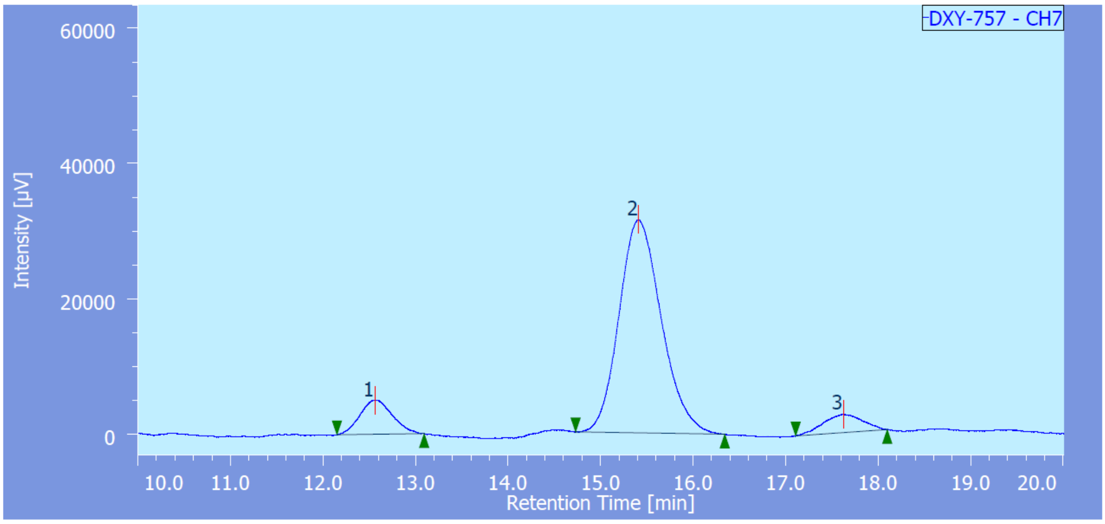

Decision

#	Peak Name	CH	tR [min]	Area [µV·sec]	Height [µV]	Area%	Height%	Quantity	NTP	Resolution	Symmetry Factor	Warning
1	Unknown	7	12.560	123403	5028	10.263	12.833	N/A	5900	3.847	1.126	
2	Unknown	7	15.410	999727	31450	83.140	80.261	N/A	5497	2.698	1.173	
3	Unknown	7	17.623	79327	2706	6.597	6.907	N/A	7503	N/A	0.958	

4. Synthetic application

4.1 Late-stage Functionalization

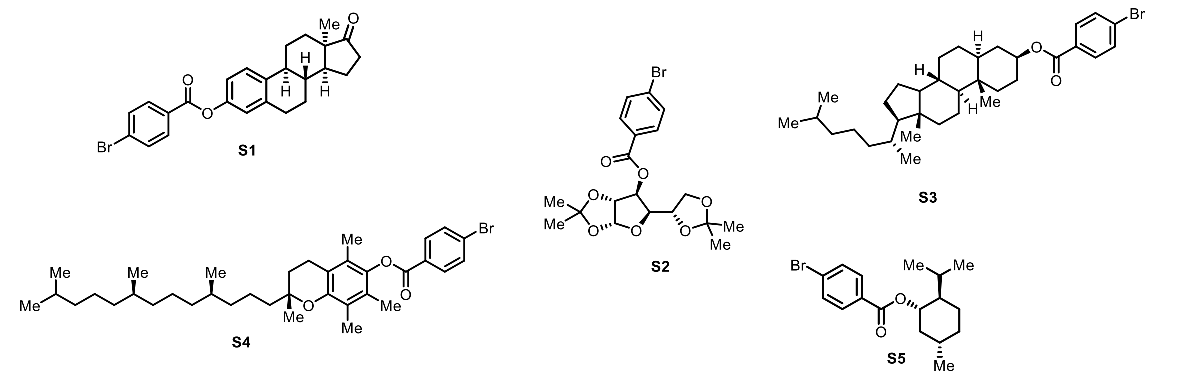

4-Bromobenzoyl esters **S1**,[10] **S2**,[11] **S3**,[12] **S4**,[13] and **S5**,[11] were prepared according to literature procedures and analytical data were in accordance with those previously reported.

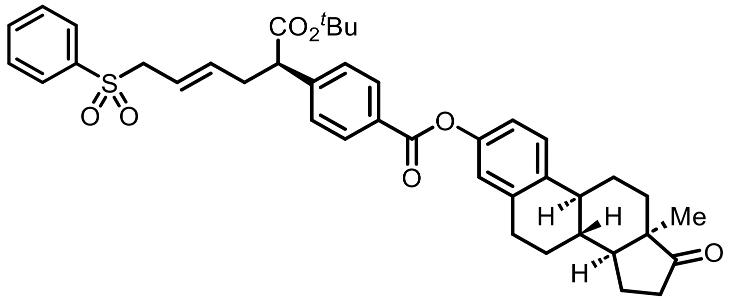

(8*R*,9*S*,13*R*,14*S*)-13-Methyl-17-oxo-7,8,9,11,12,13,14,15,16,17-decahydro-6H-cyclopenta[a]phenanthren-2-yl 4-((*R*,*E*)-1-(*tert*-butoxy)-1-oxo-6-(phenylsulfonyl)hex-4-en-2-yl)benzoate (56). White solid (Hex/EA = 2/1). 53%, >20/1 dr. $[\alpha]_D^{23}$ = +30.3 (c = 0.26, $CHCl_3$). ^1H NMR (500 MHz, $CDCl_3$) δ 8.17 – 8.08 (m, 2H), 7.83 – 7.76 (m, 2H), 7.68 – 7.62 (m, 1H), 7.58 – 7.51 (m, 2H), 7.34 (dd, *J* = 8.5, 2.1 Hz, 2H), 7.00 – 6.87 (m, 3H), 5.52 – 5.41 (m, 2H), 3.73 – 3.67 (m, 2H), 3.51 (t, *J* = 7.6 Hz, 1H), 2.96 – 2.90 (m, 2H), 2.82 – 2.73 (m, 1H), 2.55 – 2.40 (m, 3H), 2.32 (d, *J* = 4.5 Hz, 1H), 2.20 – 2.11 (m, 1H), 2.09 – 2.01 (m, 2H), 1.98 (dt, *J* = 12.7, 3.0 Hz, 1H), 1.68 – 1.58 (m, 3H), 1.53 (dd, *J* = 16.8, 14.8 Hz, 3H), 1.38 (s, 9H), 0.92 (s, 3H). ^{13}C NMR (126 MHz, $CDCl_3$) δ 221.1, 171.5, 165.3, 148.9, 144.6, 138.6, 138.2, 137.7, 137.6, 133.9, 132.6, 130.6, 129.2, 128.8, 128.5, 128.2, 126.6, 121.8, 118.9, 118.8,

115.5, 81.7, 60.0, 52.2, 50.6, 48.1, 44.3, 38.1, 36.1, 36.0, 31.7, 29.6, 28.0, 26.5, 25.9, 21.7, 14.0. IR (film): ν (cm^{-1}) 3363, 2916, 1736, 1641, 1526, 1446, 1307, 1292, 1017, 746. HR-MS (ESI) calculated $[M+Na]^+$ for $C_{41}H_{46}O_7{}^{23}Na^+$ = 705.28565, found: 705.28690.

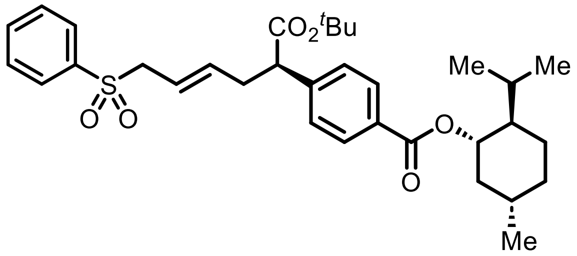

(1*S*,2*R*,4*R*)-2-Isopropyl-4-methylcyclohexyl 4-((*R,E*)-1-(*tert*-butoxy)-1-oxo-6-(phenylsulfonyl)hex-4-en-2-yl)benzoate (57). Colorless oil (Hex/EA = 2/1). 56%, >20/1 dr. $[\alpha]_D^{23}$ = -36.8 (c = 0.39, $CHCl_3$). ^{1}H NMR (500 MHz, $CDCl_3$) δ 7.90 (d, *J* = 8.3 Hz, 2H), 7.71 (dd, *J* = 8.3, 1.4 Hz, 1H), 7.59 – 7.54 (m, 1H), 7.50 – 7.44 (m, 2H), 7.22 – 7.16 (m, 2H), 5.43 – 5.33 (m, 2H), 4.86 (td, *J* = 10.9, 4.4 Hz, 1H), 3.62 (d, *J* = 6.0 Hz, 1H), 3.40 (t, *J* = 7.6 Hz, 1H), 2.71 – 2.62 (m, 1H), 2.41 – 2.30 (m, 1H), 2.04 (dq, *J* = 12.1, 2.3 Hz, 2H), 1.88 (td, *J* = 7.0, 3.1 Hz, 1H), 1.70 – 1.62 (m, 2H), 1.48 (tt, *J* = 12.4, 3.1 Hz, 2H), 1.30 (s, 9H), 1.09 – 0.96 (m, 2H), 0.85 (t, *J* = 6.6 Hz, 6H), 0.73 – 0.70 (m, 3H). ^{13}C NMR (101 MHz, $CDCl_3$) δ 171.6, 165.9, 143.5, 138.4, 137.6, 133.7, 131.8, 129.9, 129.1, 128.4, 127.8, 118.6, 81.4, 74.9, 59.9, 52.0, 47.3, 41.0, 36.0, 34.3, 31.4, 27.9, 26.5, 23.6, 22.1, 20.8, 16.5. IR (film): ν (cm^{-1}) 3363, 2916, 1736, 1641, 1526, 1446, 1307, 1292, 1017, 746. HR-MS (ESI) calculated $[M+Na]^+$ for $C_{33}H_{44}O_6{}^{23}Na^+$ = 591.27508, found: 591.27612.

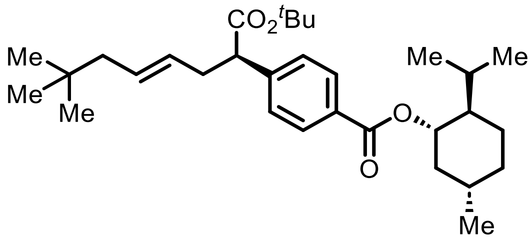

(1*S*,2*R*,4*R*)-2-Isopropyl-4-methylcyclohexyl 4-((*R,E*)-1-(tert-butoxy)-7,7-dimethyl-1-oxooct-4-en-2-yl)benzoate (58). Colorless oil (Hex/EA = 20/1). 47%, >20/1 dr. *E/Z* = 7/1. $[\alpha]_D^{24}$ = -139.03 (c = 0.14, $CHCl_3$). ^{1}H NMR (500 MHz, $CDCl_3$) δ 7.97 (d, *J* = 8.3 Hz, 2H), 7.38 – 7.32 (m, 2H), 5.48 (dt, *J* = 15.1, 7.5 Hz, 1H), 5.27 (dt, *J* = 15.1, 6.8 Hz, 1H), 4.92 (td, *J* =

10.8, 4.5 Hz, 1H), 3.55 (t, J = 7.7 Hz, 1H), 2.74 (dt, J = 14.9, 7.7 Hz, 1H), 2.42 (dt, J = 14.1, 6.9 Hz, 1H), 2.11 (dd, J = 12.9, 1.9 Hz, 1H), 1.96 (pd, J = 7.0, 2.9 Hz, 1H), 1.84 – 1.76 (m, 2H), 1.76 – 1.69 (m, 2H), 1.54 (ddt, J = 14.1, 10.8, 3.3 Hz, 3H), 1.39 (s, 9H), 1.17 – 1.04 (m, 2H), 0.92 (dd, J = 6.8, 4.1 Hz, 6H), 0.86 (s, 1H), 0.81 – 0.75 (m, 10H). ^{13}C NMR (126 MHz, $CDCl_3$) δ 172.3, 166.1, 144.5, 130.6, 129.9, 129.7, 128.6, 128.1, 81.1, 74.9, 53.3, 47.4, 47.2, 41.1, 36.7, 34.5, 31.6, 30.9, 29.3, 28.1, 26.6, 23.8, 22.2, 20.9, 16.6. IR (film): ν (cm^{-1}) 2932, 1715, 1609, 1366, 1273, 1143, 1110, 965, 758, 506. HR-MS (ESI) calculated [M+Na]$^+$ for $C_{31}H_{48}O_4{}^{23}Na^+$ = 507.34448, found: 507.34492.

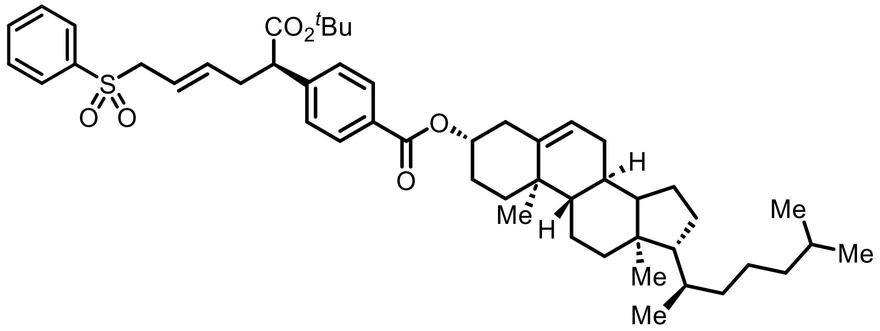

(*13R*)-13-Methyl-17-oxo-7,8,9,11,12,13,14,15,16,17-decahydro-6H-cyclopenta[a]phenanthren-2-yl 4-((*R*,*E*)-1-(*tert*-butoxy)-1-oxo-6-(phenylsulfonyl)hex-4-en-2-yl)benzoate (59). White solid (Hex/EA = 2/1). 40%, >20/1 dr. $[\alpha]_D^{24}$ = -15.0 (c = 0.29, $CHCl_3$). ^{1}H NMR (400 MHz, $CDCl_3$) δ 7.97 (d, J = 8.5 Hz, 2H), 7.78 (dd, J = 8.4, 1.4 Hz, 2H), 7.67 – 7.61 (m, 1H), 7.56 – 7.49 (m, 2H), 7.27 – 7.24 (m, 2H), 5.48 – 5.38 (m, 3H), 4.90 – 4.78 (m, 1H), 3.72 – 3.66 (m, 2H), 3.46 (t, J = 7.6 Hz, 1H), 2.81 – 2.69 (m, 1H), 2.49 – 2.39 (m, 3H), 2.05 – 1.95 (m, 3H), 1.91 (dt, J = 13.1, 3.4 Hz, 1H), 1.83 (d, J = 3.8 Hz, 1H), 1.76 – 1.67 (m, 1H), 1.63 – 1.43 (m, 8H), 1.35 (s, 9H), 1.25 – 0.95 (m, 15H), 0.92 (d, J = 6.6 Hz, 3H), 0.87 (dd, J = 6.6, 1.9 Hz, 6H), 0.69 (s, 3H). ^{13}C NMR (126 MHz, $CDCl_3$) δ 171.7, 143.6, 139.8, 138.6, 137.8, 133.8, 130.0, 129.2, 128.6, 127.9, 123.0, 118.8, 81.5, 74.8, 60.0, 56.8, 56.3, 52.1, 50.2, 42.5, 39.9, 39.7, 38.4, 37.2, 36.8, 36.3, 36.1, 35.9, 32.1, 32.0, 28.4, 28.2, 28.0, 24.4, 24.0, 23.0, 22.7, 21.2, 19.5, 18.9, 12.0. IR (film): ν (cm^{-1}) 3363, 2916, 1736, 1641, 1526, 1446, 1307, 1292, 1017, 746. HR-MS (ESI) calculated [M+Na]$^+$ for $C_{50}H_{70}O_6{}^{23}Na^+$ = 821.47853, found: 821.47975.

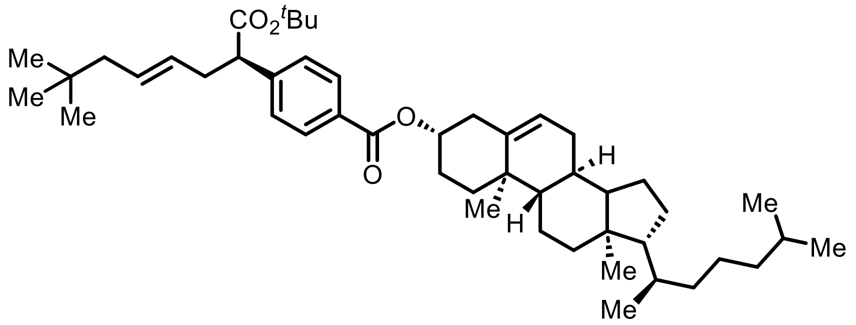

(3*S*,8*S*,9*S*,10*R*,13*R*,17*R*)-10,13-Dimethyl-17-((*R*)-6-methylheptan-2-yl)-2,3,4,7,8,9,10,11,12,13,14,15,16,17-tetradecahydro-1H-cyclopenta[a]phenanthren-3-yl 4-((*R,E*)-1-(*tert*-butoxy)-7,7-dimethyl-1-oxooct-4-en-2-yl)benzoate (60). White solid (Hex/EA = 20/1). 56%, >20/1 dr, *E/Z* = 7/1. $[\alpha]_D^{24}$ = -3.27 (c = 0.06, $CHCl_3$). ^1H NMR (500 MHz, $CDCl_3$) δ 7.97 (d, *J* = 8.4 Hz, 2H), 7.35 (d, *J* = 8.4 Hz, 2H), 5.49 (dt, *J* = 14.9, 7.5 Hz, 1H), 5.44 – 5.39 (m, 1H), 5.30 – 5.23 (m, 1H), 4.85 (dtd, *J* = 12.6, 8.5, 4.5 Hz, 1H), 3.54 (t, *J* = 7.7 Hz, 1H), 2.74 (dt, *J* = 14.6, 7.7 Hz, 1H), 2.43 (dd, *J* = 19.0, 7.2 Hz, 3H), 2.05 – 1.95 (m, 3H), 1.94 – 1.88 (m, 1H), 1.86 – 1.68 (m, 4H), 1.62 – 1.42 (m, 8H), 1.38 (s, 9H), 1.29 – 0.96 (m, 15H), 0.92 (d, *J* = 6.5 Hz, 3H), 0.89 – 0.85 (m, 7H), 0.79 (s, 7H), 0.69 (s, 3H). ^{13}C NMR (126 MHz, $CDCl_3$) δ 172.3, 166.0, 144.5, 139.8, 130.6, 129.9, 129.7, 128.7, 128.1, 122.9, 81.1, 74.7, 56.9, 56.3, 53.3, 50.2, 47.2, 42.5, 39.9, 39.7, 38.4, 37.2, 36.8, 36.6, 36.3, 36.0, 32.1, 32.0, 30.9, 29.4, 29.3, 28.4, 28.2, 28.1, 28.1, 28.0, 24.4, 24.0, 23.0, 22.7, 21.2, 19.5, 18.9, 12.0. IR (film): ν (cm^{-1}) 2931, 1727, 1603, 1509, 1447, 1367, 1149, 839, 505. HR-MS (ESI) calculated $[M+Na]^+$ for $C_{48}H_{74}O_4{}^{23}Na^+$ = 737.54793, found: 737.54892. Crystals of **60** suitable for X-ray diffraction were obtained from a mixture of hexane/EA.

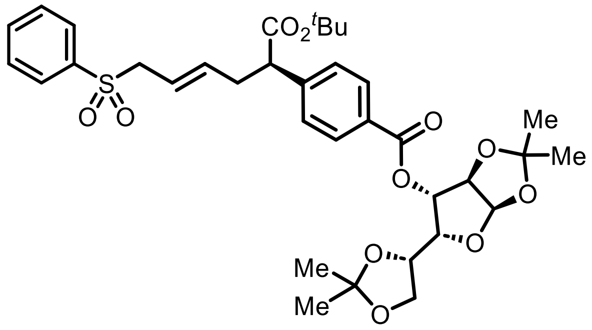

(3a*R*,5*R*,6*S*,6a*R*)-5-((*S*)-2,2-Dimethyl-1,3-dioxolan-4-yl)-2,2-dimethyltetrahydrofuro[2,3-

d][1,3]dioxol-6-yl 4-((*R*,*E*)-1-(*tert*-butoxy)-1-oxo-6-(phenylsulfonyl)hex-4-en-2-yl)benzoate (61). Colorless oil (Hex/EA = 2/1). 52%, >20/1 dr. $[\alpha]_D^{23}$ = -34.4 (c = 0.28, $CHCl_3$). ^1H NMR (500 MHz, $CDCl_3$) δ 7.95 (d, *J* = 8.3 Hz, 2H), 7.81 – 7.76 (m, 2H), 7.67 – 7.61 (m, 1H), 7.53 (t, *J* = 7.9 Hz, 2H), 7.29 (d, *J* = 8.4 Hz, 2H), 5.93 (d, *J* = 3.7 Hz, 1H), 5.48 (d, *J* = 3.0 Hz, 1H), 5.45 (t, *J* = 2.7 Hz, 2H), 4.61 (d, *J* = 3.7 Hz, 1H), 4.37 – 4.30 (m, 2H), 4.10 (qd, *J* = 8.6, 5.2 Hz, 2H), 3.71 – 3.66 (m, 2H), 3.52 – 3.45 (m, 1H), 2.74 (ddt, *J* = 10.5, 5.4, 3.0 Hz, 1H), 2.49 – 2.37 (m, 1H), 1.55 (s, 3H), 1.40 (s, 3H), 1.36 (s, 9H), 1.31 (s, 3H), 1.25 (s, 3H). ^{13}C NMR (126 MHz, $CDCl_3$) δ 171.5, 165.1, 144.5, 138.6, 137.6, 133.8, 130.2, 129.2, 128.7, 128.5, 128.2, 118.8, 112.5, 109.5, 105.2, 83.5, 81.7, 80.0, 72.7, 67.4, 60.0, 52.1, 36.1, 35.0, 31.6, 28.0, 27.0, 26.9, 26.3, 25.3. IR (film): ν (cm^{-1}) 3363, 2916, 1736, 1641, 1526, 1446, 1307, 1292, 1017, 746. HR-MS (ESI) calculated $[M+Na]^+$ for $C_{35}H_{44}O_{11}S^+$ = 695.24965, found: 695.24995.

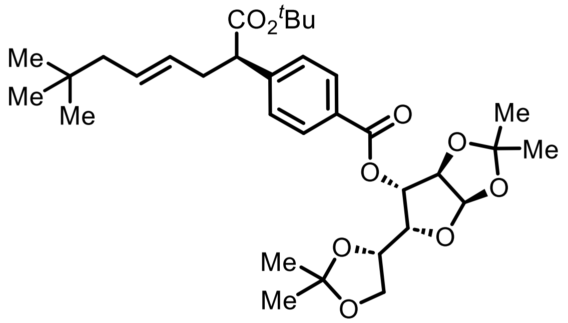

(3a*R*,5*R*,6*S*,6a*R*)-5-((*S*)-2,2-Dimethyl-1,3-dioxolan-4-yl)-2,2-dimethyltetrahydrofuro[2,3-d][1,3]dioxol-6-yl 4-((*R*,*E*)-1-(*tert*-butoxy)-7,7-dimethyl-1-oxooct-4-en-2-yl)benzoate (62). Colorless oil (Hex/EA = 20/1). 58%, >20/1 dr, *E/Z* = 6/1. $[\alpha]_D^{24}$ = -22.43 (c = 0.15, $CHCl_3$). ^1H NMR (500 MHz, $CDCl_3$) δ 7.96 (d, *J* = 8.3 Hz, 2H), 7.38 (d, *J* = 8.3 Hz, 2H), 5.94 (d, *J* = 3.8 Hz, 1H), 5.49 (q, *J* = 3.2 Hz, 2H), 5.27 (dt, *J* = 14.1, 6.5 Hz, 1H), 4.61 (d, *J* = 3.7 Hz, 1H), 4.39 – 4.28 (m, 3H), 4.15 – 4.05 (m, 2H), 3.56 (t, *J* = 7.6 Hz, 1H), 2.75 (dt, *J* = 14.8, 7.5 Hz, 1H), 2.44 (dt, *J* = 14.1, 7.2 Hz, 1H), 1.81 (d, *J* = 7.9 Hz, 2H), 1.55 (s, 3H), 1.41 (s, 3H), 1.39 (d, *J* = 2.4 Hz, 8H), 1.32 (s, 3H), 1.27 (s, 4H), 0.87 (s, 1H), 0.79 (s, 7H). ^{13}C NMR (126 MHz, $CDCl_3$) δ 171.9, 165.0, 145.3, 130.6, 129.9, 128.4, 128.3, 128.2, 112.4, 109.4, 105.2, 83.5, 81.1, 80.0, 72.6, 67.3, 53.2, 47.0, 36.4, 30.7, 29.2, 29.1, 27.9, 26.8, 26.8, 26.2, 25.2. IR (film): ν (cm^{-1}) 2934, 1726, 1609, 1368, 1265, 1144, 1019, 845, 758, 703, 507. HR-MS (ESI) calculated $[M+Na]^+$ for $C_{33}H_{48}O_9{}^{23}Na^+$ = 611.31905, found: 611.31985.

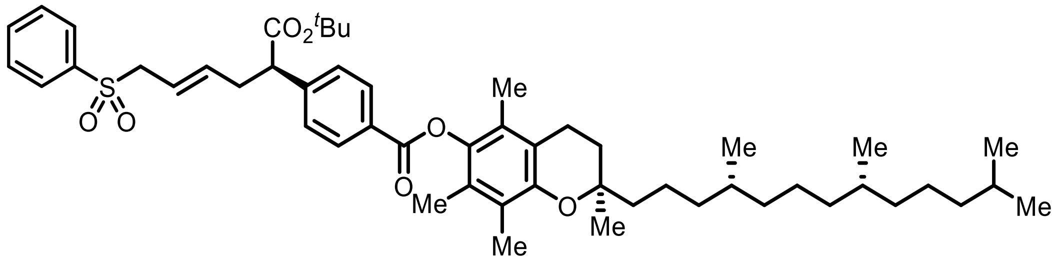

(*R*)-2,5,7,8-Tetramethyl-2-((4*R*,8*R*)-4,8,12-trimethyltridecyl)chroman-6-yl 4-((*R*,*E*)-1-(*tert*-butoxy)-1-oxo-6-(phenylsulfonyl)hex-4-en-2-yl)benzoate (63). White solid (Hex/EA = 2/1). 48%, >20/1 dr. $[\alpha]_D^{23}$ = -9.9 (c = 0.37, $CHCl_3$). ^1H NMR (400 MHz, $CDCl_3$) δ 8.17 (d, *J* = 8.4 Hz, 2H), 7.83 – 7.77 (m, 2H), 7.68 – 7.61 (m, 1H), 7.59 – 7.50 (m, 2H), 7.35 (d, *J* = 8.5 Hz, 2H), 5.52 – 5.45 (m, 2H), 3.72 (d, *J* = 6.1 Hz, 2H), 3.52 (t, *J* = 7.6 Hz, 1H), 2.84 – 2.74 (m, 1H), 2.61 (t, *J* = 7.0 Hz, 2H), 2.52 – 2.42 (m, 1H), 2.12 (s, 3H), 2.05 (d, *J* = 1.6 Hz, 3H), 2.01 (s, 3H), 1.86 – 1.73 (m, 2H), 1.55 – 1.48 (m, 2H), 1.40 (s, 12H), 1.30 – 1.19 (m, 11H), 1.18 – 1.01 (m, 7H), 0.87 (dd, *J* = 6.7, 0.7 Hz, 13H). ^{13}C NMR (126 MHz, $CDCl_3$) δ 171.6, 165.1, 149.6, 144.4, 140.7, 138.6, 137.7, 133.9, 130.6, 129.2, 128.8, 128.6, 128.2, 127.0, 125.2, 123.3, 118.9, 117.6, 81.7, 75.2, 60.0, 52.2, 39.5, 37.6, 37.4, 36.3, 32.9, 32.9, 32.8, 28.3, 28.1, 28.1, 24.9, 24.6, 24.4, 23.8, 22.9, 22.8, 21.2, 20.8, 19.9, 19.8, 13.2, 12.4, 12.0. IR (film): ν (cm^{-1}) 3363, 2916, 1736, 1641, 1526, 1446, 1307, 1292, 1017, 746. HR-MS (ESI) calculated $[M+Na]^+$ for $C_{52}H_{74}O_7S^+$ = 865.50475, found: 865.50630.

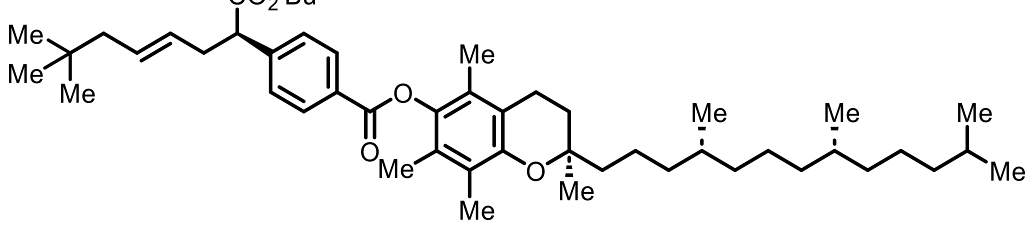

(*R*)-2,5,7,8-Tetramethyl-2-((4*R*,8*R*)-4,8,12-trimethyltridecyl)chroman-6-yl 4-((*R*,*E*)-1-(*tert*-butoxy)-7,7-dimethyl-1-oxooct-4-en-2-yl)benzoate (64). White solid (Hex/EA = 20/1). 61%, >20/1 dr, *E/Z* = 6/1. $[\alpha]_D^{24}$ = -11.12 (c = 0.15, $CHCl_3$). ^1H NMR (500 MHz, $CDCl_3$) δ 8.23 – 8.13 (m, 2H), 7.48 – 7.40 (m, 2H), 5.56 – 5.44 (m, 1H), 5.35 – 5.24 (m, 1H), 3.60 (t, *J*

= 7.7 Hz, 1H), 2.78 (dt, J = 14.8, 7.8 Hz, 1H), 2.62 (t, J = 6.8 Hz, 2H), 2.48 (dt, J = 14.5, 7.4 Hz, 1H), 2.12 (s, 3H), 2.05 (s, 3H), 2.01 (s, 3H), 1.88 – 1.72 (m, 4H), 1.64 – 1.45 (m, 6H), 1.42 (s, 12H), 1.32 – 1.02 (m, 17H), 0.87 (d, J = 6.5 Hz, 12H), 0.79 (s, 7H). ^{13}C NMR (126 MHz, $CDCl_3$) δ 172.1, 165.1, 149.5, 145.1, 140.6, 130.6, 130.3, 128.4, 128.4, 128.3, 126.9, 125.2, 123.1, 117.5, 81.1, 75.1, 53.2, 47.0, 40.4, 39.4, 37.5, 37.3, 36.7, 32.8, 32.8, 32.7, 30.8, 29.2, 28.0, 24.8, 24.5, 24.2, 23.7, 22.7, 22.6, 21.1, 20.7, 19.8, 19.7, 13.1, 12.2, 11.9. IR (film): ν (cm^{-1}) 3363, 2916, 1736, 1641, 1526, 1446, 1307, 1292, 1017, 746. HR-MS (ESI) calculated $[M+Na]^+$ for $C_{50}H_{78}O_5{}^{23}Na^+$ = 781.57415, found: 781.57447.

4.2 Derivatization of products

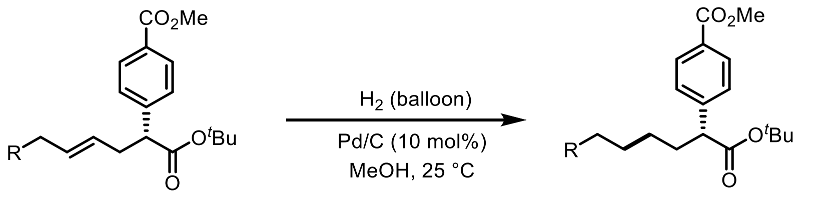

R= $PhSO_2$, **65,** 99%, 94 : 6 e.r.
R= ^{t}Bu, **66**, 99%, 93:7 e.r.

The corresponding starting material (**1** or **2,** 0.05 mmol) and 5% Pd/C (10.6 mg, 0.005 mmol Pd, 10 mol%) were added to a 7.5 ml vial equipped with a magnetic stirring bar. Methanol (4 mL) was added and the vial was sealed with a septum. The reaction mixture was sparged with dry nitrogen for 2 min, followed by hydrogen for 30 seconds. The reaction was stirred vigorously under an atmosphere of hydrogen at 25 °C overnight. Upon completion of the reaction, 5 mL of water was added to the reaction mixture, which was subsequently filtered through a celite plug with washings of wet acetone. The transformation was quantitative. The enantiomeric ratio (e.r.) and the E/Z ratio of the product were determined by HPLC with a chiral stationary phase.

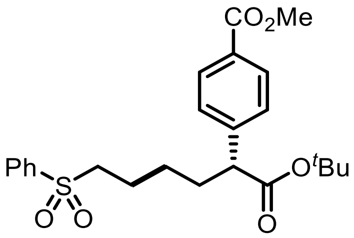

Methyl (*R*)-4-(1-(*tert*-butoxy)-1-oxo-6-(phenylsulfonyl)hexan-2-yl)benzoate (65). Colorless oil (Hex/EA = 2/1). 99%, 94:6 er. $[\alpha]_D^{23}$ = -12.8 (c = 0.16, $CHCl_3$). ^1H NMR (400 MHz, $CDCl_3$) δ 7.96 (d, *J* = 8.4 Hz, 2H), 7.90 – 7.84 (m, 2H), 7.67 – 7.61 (m, 1H), 7.55 (td, *J* = 6.8, 1.7 Hz, 2H), 7.29 (d, *J* = 8.4 Hz, 2H), 3.89 (s, 3H), 3.42 (t, *J* = 7.6 Hz, 1H), 3.08 – 2.99 (m, 2H), 2.05 – 1.93 (m, 1H), 1.78 – 1.60 (m, 4H), 1.35 (s, 10H). ^{13}C NMR (101 MHz, $CDCl_3$) δ 172.3, 167.0, 144.5, 139.2, 133.8, 130.0, 129.4, 129.2, 128.1, 127.9, 81.3, 56.1, 52.4, 52.2, 32.8, 28.0, 26.3, 22.6. IR (film) ν (cm^{-1}) 2923, 2852, 1719, 1610, 1446, 1436, 1368, 1307, 1279, 1180, 1147, 1112, 1087, 1020, 965, 847, 733, 689, 594, 563, 532. HR-MS (ESI) m/z calcd for $C_{24}H_{30}NaO_6S^+$ 469.16553, found 469.16553, [M+Na$^+$]. The enantiomeric ratio of **65** was determined by HPLC analysis on Chiralpak AD-H column. Conditions: hexane/isopropanol = 80/20, flow rate = 1.0 mL/min, uv-vis detection at λ = 220 nm, t_R = 15.5 min (major), 18.1 min (minor).

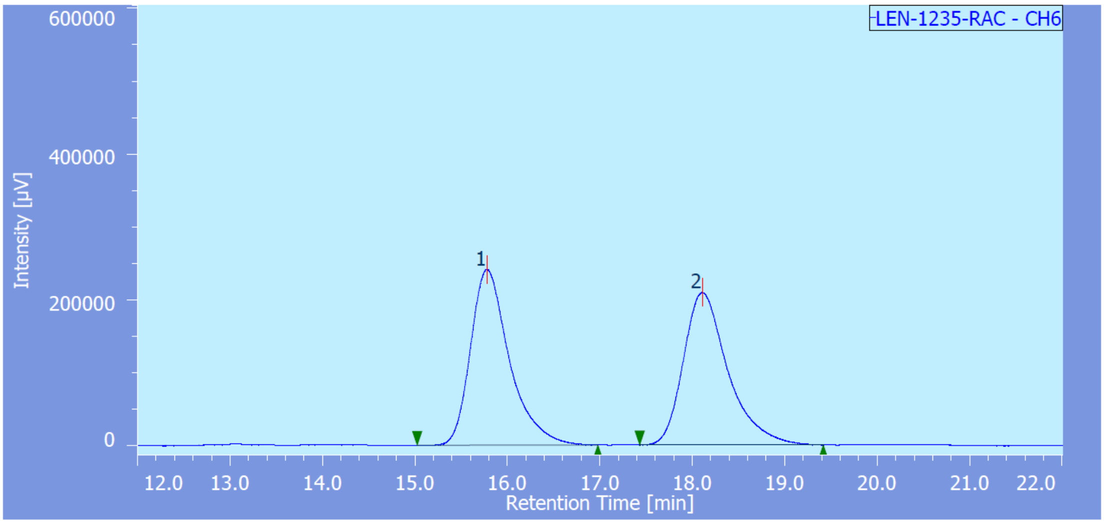

Decision

#	Peak Name	CH	tR [min]	Area [µV·sec]	Height [µV]	Area%	Height%	Quantity	NTP	Resolution	Symmetry Factor	Warning
1	Unknown	6	15.780	7055152	240845	50.146	53.532	N/A	7504	2.982	1.402	
2	Unknown	6	18.107	7014177	209063	49.854	46.468	N/A	7506	N/A	1.400	

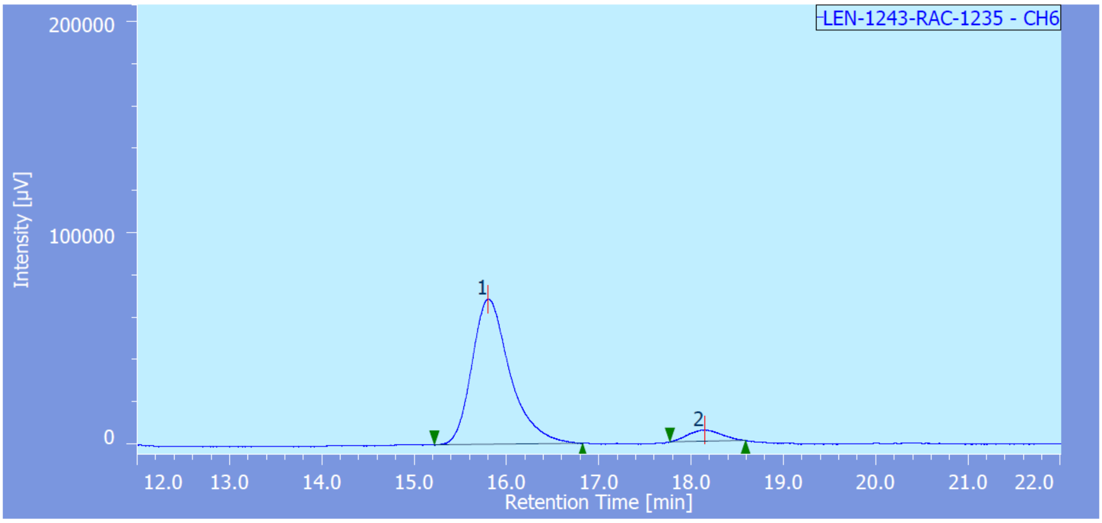

Decision

#	Peak Name	CH	tR [min]	Area [µV·sec]	Height [µV]	Area%	Height%	Quantity	NTP	Resolution	Symmetry Factor	Warning
1	Unknown	6	15.803	1966913	68761	93.578	92.856	N/A	7791	3.298	1.365	
2	Unknown	6	18.147	134981	5290	6.422	7.144	N/A	10487	N/A	1.074	

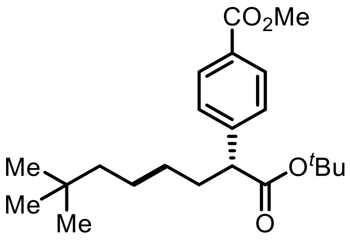

Methyl (*R*)-4-(1-(*tert*-butoxy)-7,7-dimethyl-1-oxooctan-2-yl)benzoate (66). Colorless oil (Hex/EA = 20/1). 99%, 93:7 er. $[\alpha]_D^{24}$ = -8.85 (c = 0.11, $CHCl_3$). ^1H NMR (400 MHz, $CDCl_3$) δ 7.98 (d, *J* = 8.5 Hz, 2H), 7.37 (d, *J* = 8.5 Hz, 2H), 3.90 (s, 3H), 3.48 (t, *J* = 7.7 Hz, 1H), 2.23 – 2.15 (m, 1H), 2.09 – 1.99 (m, 1H), 1.76 – 1.67 (m, 1H), 1.61 – 1.51 (m, 1H), 1.38 (s, 9H), 1.23 (d, *J* = 4.3 Hz, 2H), 1.15 – 1.08 (m, 2H), 0.84 (s, 9H). ^{13}C NMR (101 MHz, $CDCl_3$) δ 172.7, 167.0, 145.2, 129.8, 128.8, 127.9, 80.9, 52.9, 52.1, 44.0, 33.5, 29.4, 28.5, 28.1, 27.9, 24.3. IR (film): ν (cm^{-1}) 2932, 1725, 1609, 1366, 1277, 1147, 756. HR-MS (ESI) calculated $[M+Na]^+$ for $C_{22}H_{34}O_4{}^{23}Na^+$ = 385.23493, found: 385.23529. The enantiomeric ratio of **66** was determined by HPLC analysis on Chiralpak NR column. Conditions: hexane/isopropanol = 95/5, flow rate = 1.0 mL/min, uv-vis detection at λ = 254 nm, t_R = 10.7 min (major), 12.0 min (minor).

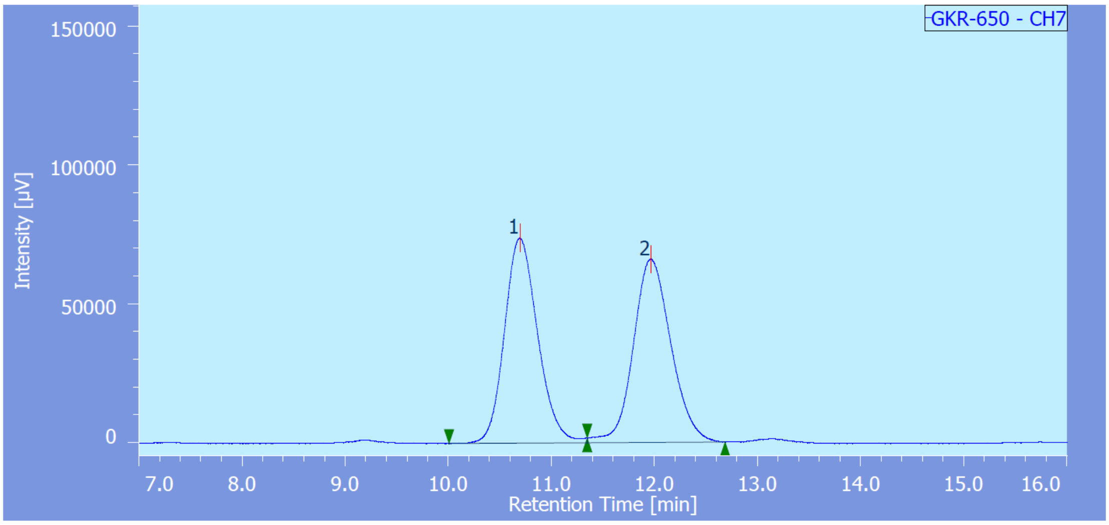

#	Peak Name	CH	tR [min]	Area [µV·sec]	Height [µV]	Area%	Height%	Quantity	NTP	Resolution	Symmetry Factor	Warning
1	Unknown	7	10.693	1655285	73766	49.716	52.807	N/A	5423	2.065	1.158	
2	Unknown	7	11.963	1674166	65923	50.284	47.193	N/A	5375	N/A	1.114	

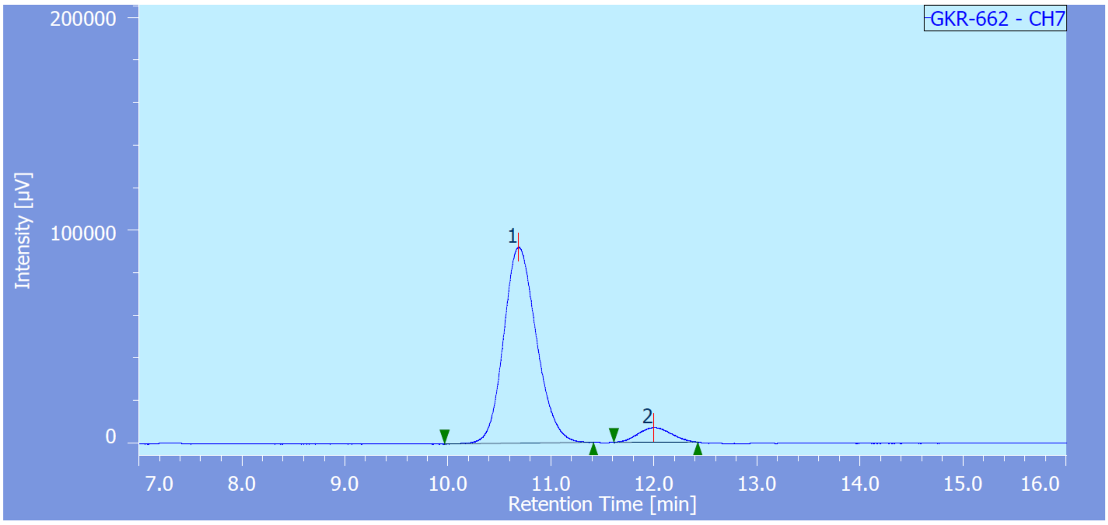

#	Peak Name	CH	tR [min]	Area [µV·sec]	Height [µV]	Area%	Height%	Quantity	NTP	Resolution	Symmetry Factor	Warning
1	Unknown	7	10.687	2035822	92092	92.844	93.027	N/A	5506	2.194	1.157	
2	Unknown	7	11.997	156920	6903	7.156	6.973	N/A	5967	N/A	1.082	

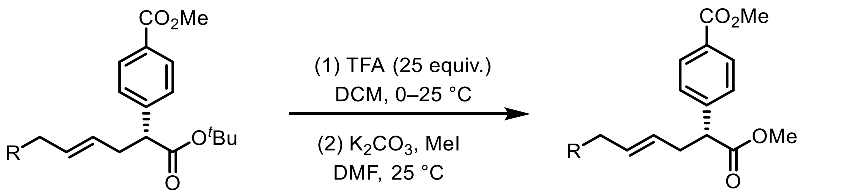

R= $PhSO_2$, **67**, 72%, 95 : 5 e.r.
R= ^{t}Bu, **68,** 49% (8:1), 93 : 7 e.r.

The corresponding starting material (**1** or **2,** 0.05 mmol) was dissolved in dry DCM (4 mL) in an oven-dried 7.5 ml vial equipped with a magnetic stirring bar and septum cap. The solution was cooled to 0 °C and TFA (9.3 µL, 0.125 mmol, 25 equiv.) was added dropwise. The

consumption of starting material was monitored by TLC and completion of reaction was observed after 24 h. The reaction mixture was then diluted with methanol and repeatedly co-evaporated with further methanol to remove residual TFA. The residue was dried under high vacuum overnight and was used in the subsequent step without further purification.

The resulting carboxylic acid and K_2CO_3 (41.5 mg, 0.3 mmol, 6 equiv.) were added to an oven-dried 7.5 mL vial equipped with a magnetic stirring bar and septum cap. Dry DMF (4 mL) was added and the reaction was stirred for 5 min. Then iodomethane (57 μL, 0.9 mmol, 18 equiv.) was added dropwise and the reaction mixture was stirred at 25 °C for 24 h. The reaction mixture was diluted with ethyl acetate and the organic phase washed repeatedly with $NaHCO_3$ (sat.) and water to remove any residual acid and DMF. The organic phase was concentrated under reduced pressure and the residue purified by column chromatography on silica gel. The enantiomeric ratio (e.r.) and the *E*/*Z* ratio of the product were determined by HPLC with a chiral stationary phase.

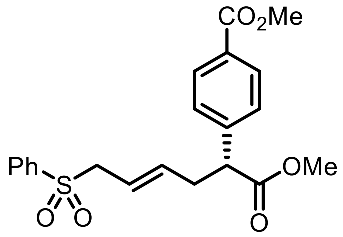

Methyl (*R,E*)-4-(1-methoxy-1-oxo-6-(phenylsulfonyl)hex-4-en-2-yl)benzoate (67). Colorless oil (Hex/EA = 2/1). 72%, 95:5 er. $[\alpha]_D^{23}$ = -19.9 (c = 0.033, $CHCl_3$). ^{1}H NMR (400 MHz, $CDCl_3$) δ 8.00 – 7.95 (m, 2H), 7.79 (dd, *J* = 8.4, 1.4 Hz, 2H), 7.65 (t, *J* = 7.4 Hz, 1H), 7.57 – 7.51 (m, 2H), 7.30 – 7.26 (m, 2H), 5.52 – 5.39 (m, 2H), 3.91 (s, 3H), 3.72 – 3.68 (m, 2H), 3.65 (s, 3H), 3.58 (t, *J* = 7.7 Hz, 1H), 2.85 – 2.76 (m, 1H), 2.53 – 2.43 (m, 1H). ^{13}C NMR (101 MHz, $CDCl_3$) δ 172.93, 166.85, 143.13, 138.56, 137.33, 133.85, 130.20, 129.63, 129.22, 128.55, 128.08, 119.21, 59.99, 52.49, 52.32, 51.05, 36.23. IR (film) ν (cm^{-1}) 2953, 2918, 1718, 1610, 1446, 1435, 1417, 1307, 1279, 1181, 1150, 1138, 1110, 1086, 1020, 970, 856, 765, 732, 703, 689, 599, 556, 529. HR-MS (ESI) m/z calcd for $C_{21}H_{22}NaO_6S^+$ 425.10293, found 425.10361, [M+Na$^+$]. The enantiomeric ratio of **67** was determined by HPLC analysis on Chiralpak NR column. Conditions: hexane/isopropanol = 50/50, flow rate = 1.0 mL/min, uv-vis detection at λ = 210 nm, t_R = 59.5 min (minor), 65.1 min (major).

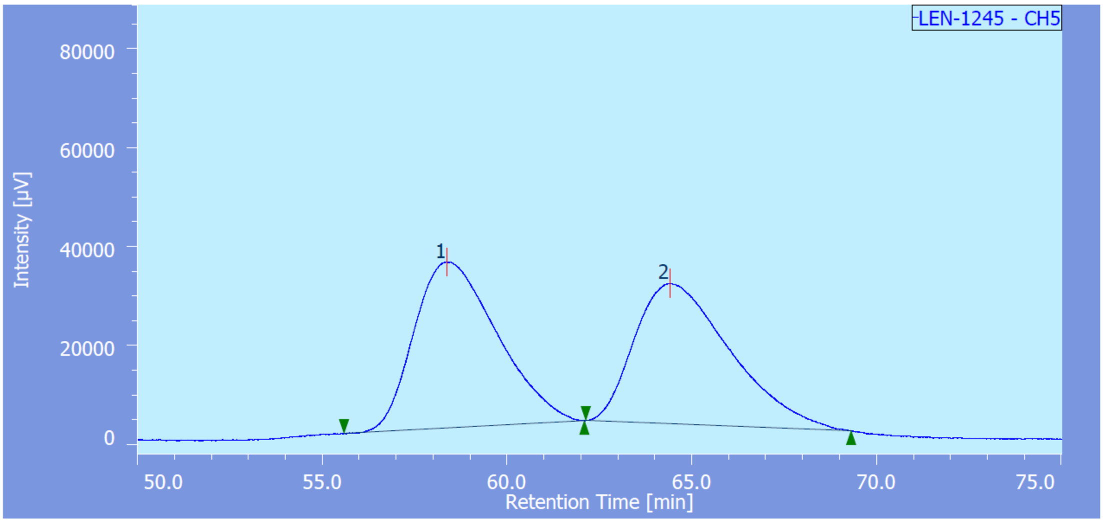

Decision

#	Peak Name	CH	tR [min]	Area [µV·sec]	Height [µV]	Area%	Height%	Quantity	NTP	Resolution	Symmetry Factor	Warning
1	Unknown	5	58.367	5245252	33581	50.692	54.204	N/A	3062	1.341	1.365	
2	Unknown	5	64.397	5102120	28372	49.308	45.796	N/A	2879	N/A	1.560	

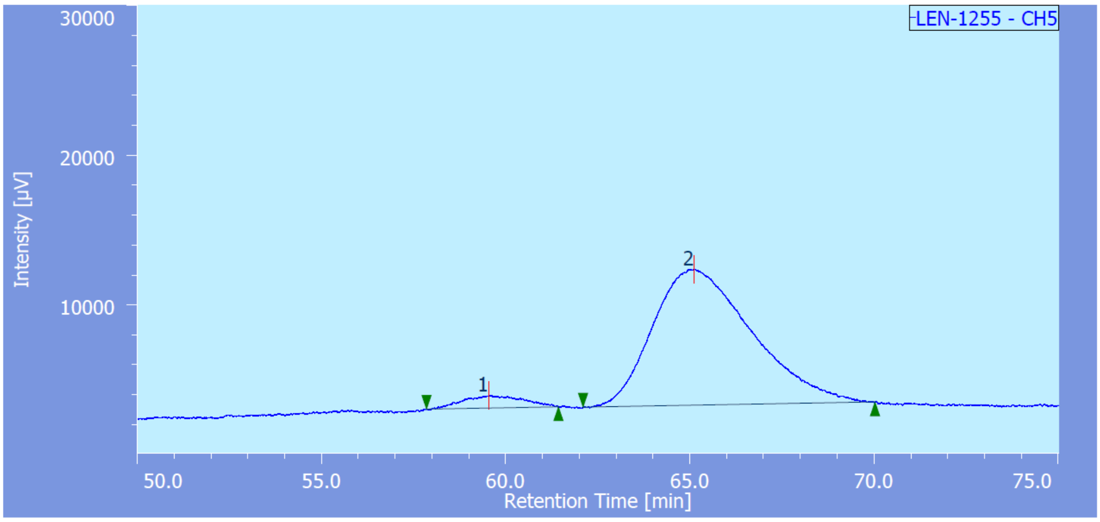

Decision

#	Peak Name	CH	tR [min]	Area [µV·sec]	Height [µV]	Area%	Height%	Quantity	NTP	Resolution	Symmetry Factor	Warning
1	Unknown	5	59.547	95816	837	5.347	8.439	N/A	4331	1.302	1.106	
2	Unknown	5	65.120	1696162	9086	94.653	91.561	N/A	2752	N/A	1.354	

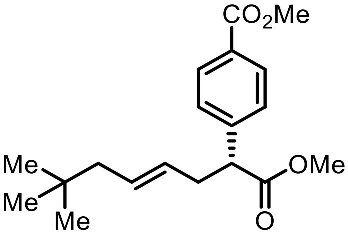

Methyl (*R*,*E*)-4-(1-methoxy-7,7-dimethyl-1-oxooct-4-en-2-yl)benzoate (68). Colorless oil (Hex/EA = 20/1). 49%, 93:7 er, *E/Z* = 8/1. $[\alpha]_D^{24}$ = -54.91 (c = 0.15, $CHCl_3$). ^1H NMR (400 MHz, $CDCl_3$) δ 8.02 – 7.94 (m, 2H), 7.41 – 7.34 (m, 2H), 5.55 – 5.42 (m, 1H), 5.31 – 5.19 (m,

1H), 3.90 (s, 3H), 3.65 (s, 4H), 2.79 (dddd, J = 14.1, 8.3, 7.1, 1.1 Hz, 1H), 2.48 (dtd, J = 14.0, 7.2, 1.3 Hz, 1H), 1.79 (dd, J = 7.5, 1.1 Hz, 2H), 0.78 (s, 9H). ^{13}C NMR (101 MHz, $CDCl_3$) δ 173.5, 166.9, 143.9, 131.0, 130.0, 129.2, 128.2, 128.2, 52.2, 52.2, 52.1, 47.1, 36.7, 30.8, 29.2. IR (film): ν (cm^{-1}) 2951, 1724, 1610, 1435, 1277, 1110, 971, 704, 505. HR-MS (ESI) calculated $[M+H]^+$ for $C_{19}H_{27}O_4^+$ = 319.19039, found: 319.19030. The enantiomeric ratio of **68** was determined by HPLC analysis on Chiralpak NR column. Conditions: hexane/isopropanol = 99/1, flow rate = 1.0 mL/min, uv-vis detection at λ = 220 nm, t_R = 40.5 min (major, *Z*), 18.4 min (minor, *E*), 50.9 min (major, *E*).

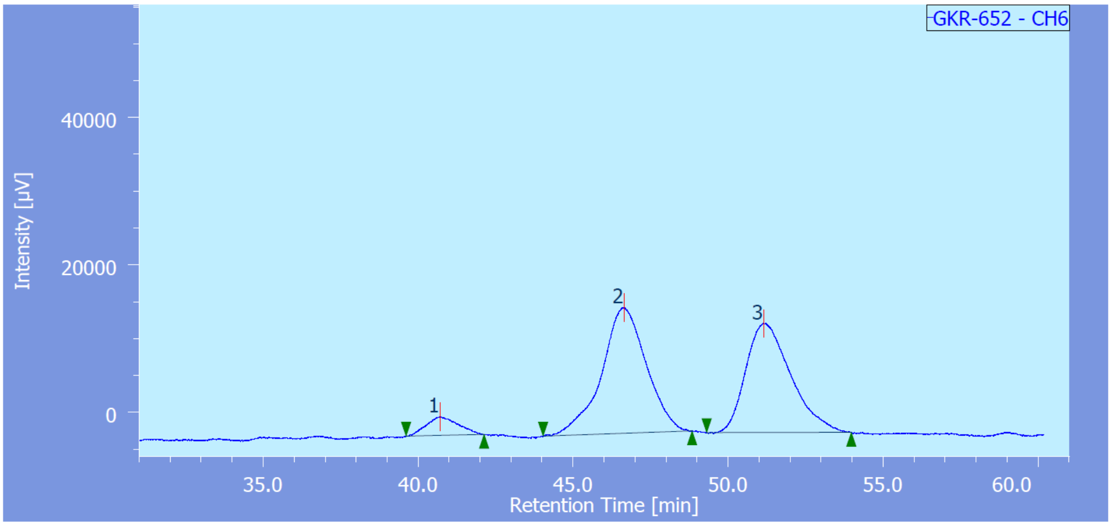

#	Peak Name	CH	tR [min]	Area [µV·sec]	Height [µV]	Area%	Height%	Quantity	NTP	Resolution	Symmetry Factor	Warning
1	Unknown	6	40.703	181988	2494	5.363	7.262	N/A	6798	2.665	1.176	
2	Unknown	6	46.643	1715388	17060	50.550	49.680	N/A	5593	1.755	0.934	
3	Unknown	6	51.143	1496099	14787	44.088	43.058	N/A	5966	N/A	1.409	

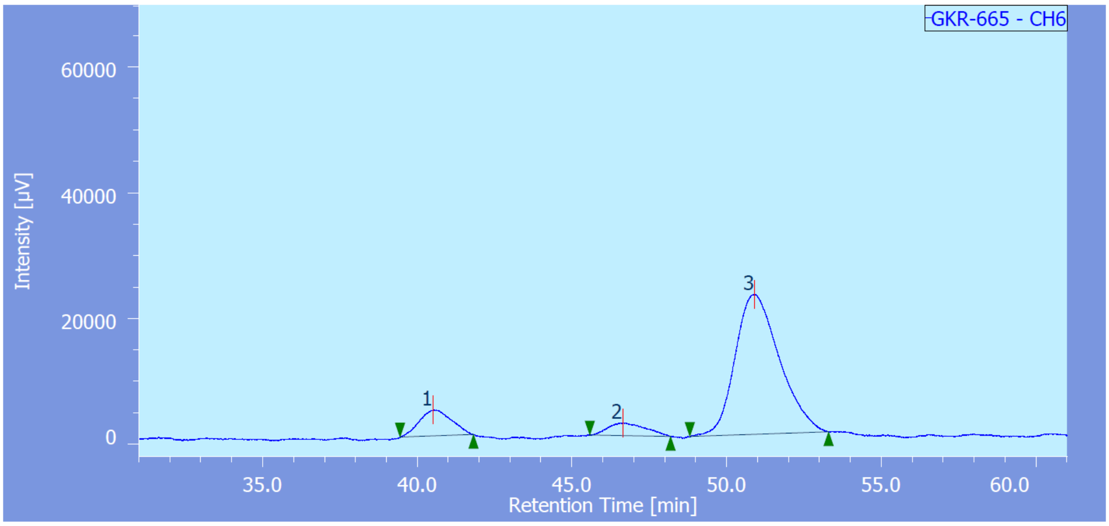

#	Peak Name	CH	tR [min]	Area [µV·sec]	Height [µV]	Area%	Height%	Quantity	NTP	Resolution	Symmetry Factor	Warning
1	Unknown	6	40.517	300535	4163	11.155	14.613	N/A	6206	2.687	1.106	
2	Unknown	6	46.643	174713	2037	6.485	7.150	N/A	5506	1.668	1.273	
3	Unknown	6	50.907	2218999	22287	82.361	78.237	N/A	6085	N/A	1.236	

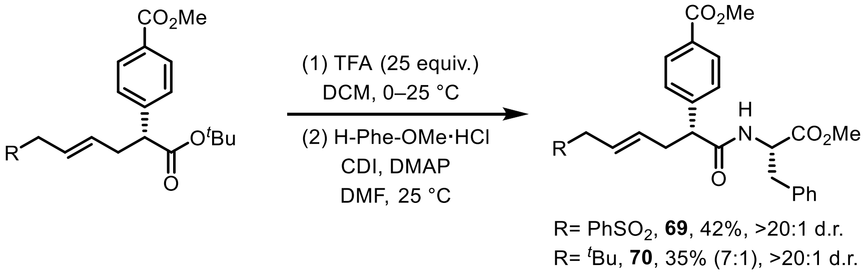

The corresponding starting material (**1** or **2,** 0.05 mmol) was dissolved in dry DCM (4 mL) in an oven-dried 7.5 ml vial equipped with a magnetic stirring bar and septum cap. The solution was cooled to 0 °C and TFA (9.3 µL, 0.125 mmol, 25 equiv.) was added dropwise. The consumption of starting material was monitored by TLC and completion of reaction was observed after 24 h. The reaction mixture was then diluted with methanol and repeatedly co-evaporated with further methanol to remove residual TFA. The residue was dried under high vacuum overnight and was used in the subsequent step without further purification.

To an oven-dried 7.5 mL vial equipped with a magnetic stirring bar and septum cap were added sequentially *L*-Phenylalanine methyl ester hydrochloride (12.9 mg, 0.06 mmol, 1.2 equiv.), *N*-(3-dimethylaminopropyl)-*N'*-ethylcarbodiimide hydrochloride (13.1 mg, 0.07 mmol, 1.4 equiv.), and DMAP (26.0 mg, 0.22 mmol, 4.4 equiv.). The crude carboxylic acid from the previous step was dissolved separately in dry DMF (4 mL, 0.012 M) and added to the other reactants by syringe, and the reaction was stirred at 25 °C for 48 h. The crude reaction mixture was diluted with ethyl acetate, washed with small portions of brine, and dried over anhydrous sodium sulfate, then concentrated under reduced pressure. The residue was purified by column chromatography on silica gel. The diastereomeric ratio (d.r.) and the *E*/*Z* ratio of the product were determined by ^{1}H NMR.

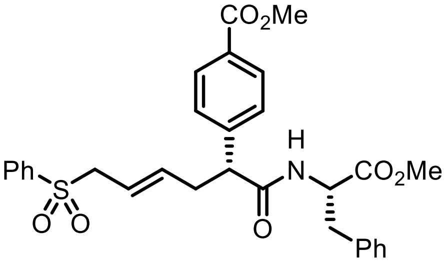

Methyl (*R*,*E*)-4-(1-methoxy-1-oxo-6-(phenylsulfonyl)hex-4-en-2-yl)benzoate (69).

Colorless oil (Hex/EA = 1/1). 42%, >20/1 dr. $[\alpha]_D^{23}$ = +1.8 (c = 0.067, $CHCl_3$). ^1H NMR (400 MHz, $CDCl_3$) δ 8.02 – 7.95 (m, 2H), 7.75 (dt, *J* = 7.9, 1.2 Hz, 2H), 7.65 – 7.60 (m, 1H), 7.51 (ddd, *J* = 8.1, 6.7, 1.3 Hz, 2H), 7.23 – 7.20 (m, 2H), 7.16 – 7.11 (m, 1H), 7.08 – 7.01 (m, 2H), 6.69 – 6.62 (m, 2H), 5.77 (d, *J* = 7.6 Hz, 1H), 5.47 – 5.35 (m, 2H), 4.84 (dt, *J* = 7.9, 5.6 Hz, 1H), 3.94 (s, 3H), 3.73 (s, 3H), 3.69 – 3.63 (m, 2H), 3.32 (t, *J* = 7.6 Hz, 1H), 3.02 – 2.90 (m, 2H), 2.88 – 2.79 (m, 1H), 2.51 – 2.38 (m, 1H). ^{13}C NMR (101 MHz, $CDCl_3$) δ 171.9, 170.9, 166.8, 143.9, 138.6, 137.9, 135.3, 133.8, 130.4, 129.6, 129.2, 129.2, 128.6, 128.5, 128.1, 127.2, 118.9, 60.0, 53.0, 52.6, 52.6, 52.4, 37.6, 35.9. IR (film) ν (cm^{-1}) 3354, 3029, 2953, 2252, 1742, 1718, 1672, 1609, 1524, 1436, 1368, 1307, 1280, 1180, 1151, 1138, 1112, 1085, 1020, 968, 911, 860, 805, 729, 701, 689, 647, 598, 552, 528, 505. HR-MS (ESI) m/z calcd for $C_{30}H_{32}NO_7S^+$ 550.18940, found 550.18994, $[M+H^+]$.

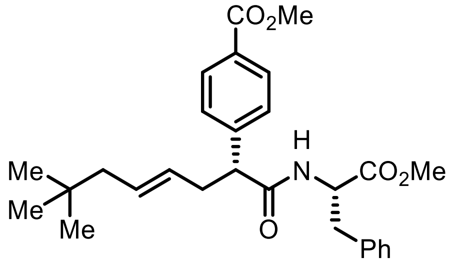

Methyl 4-((*R*,*E*)-1-(((*S*)-1-methoxy-1-oxo-3-phenylpropan-2-yl)amino)-7,7-dimethyl-1-oxooct-4-en-2-yl)benzoate (70). Colorless oil (Hex/EA = 10/1). 35%, >20/1 dr, *E/Z* = 7/1. $[\alpha]_D^{24}$ = + 31.59 (c = 0.17, $CHCl_3$). ^1H NMR (400 MHz, $CDCl_3$) δ 8.02 – 7.95 (m, 2H), 7.33 – 7.29 (m, 2H), 7.17 – 7.11 (m, 1H), 7.09 – 7.03 (m, 2H), 6.71 – 6.63 (m, 2H), 5.83 (d, *J* = 7.9 Hz, 1H), 5.48 – 5.39 (m, 1H), 5.26 – 5.14 (m, 1H), 4.87 (dt, *J* = 7.8, 5.4 Hz, 1H), 3.94 (s, 3H), 3.73 (s, 3H), 3.40 (t, *J* = 7.6 Hz, 1H), 3.05 – 2.92 (m, 2H), 2.87 – 2.78 (m, 1H), 2.47 (dt, *J* = 14.3, 7.5 Hz, 1H), 1.79 – 1.69 (m, 2H), 0.83 (s, 1H), 0.73 (s, 7H). ^{13}C NMR (101 MHz, $CDCl_3$) δ 171.9, 171.6, 167.0, 144.7, 135.4, 130.8, 130.2, 129.3, 128.6, 128.3, 127.1, 53.8, 53.0, 52.5, 52.3, 47.1, 37.7, 36.2, 30.8, 29.2 (two peaks are overlapping with others). IR (film): ν (cm^{-1}) 3363, 2916, 1736, 1641, 1526, 1446, 1307, 1292, 1017, 746. HR-MS (ESI) calculated $[M+H]^+$ for $C_{19}H_{27}O_4^+$ = 466.25880, found: 466.25909.

5. Mechanistic Studies

5.1 Substrate control experiments

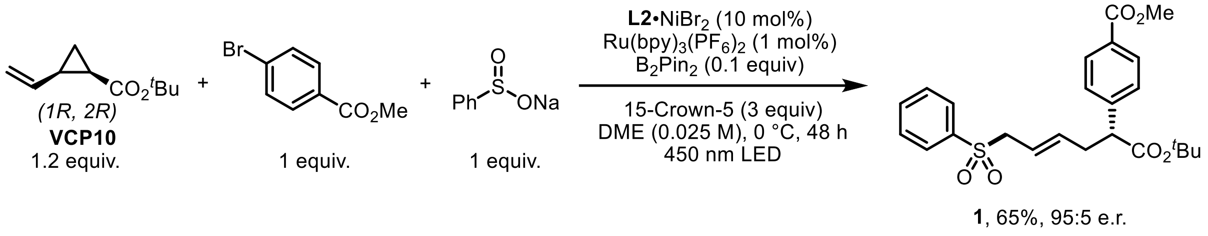

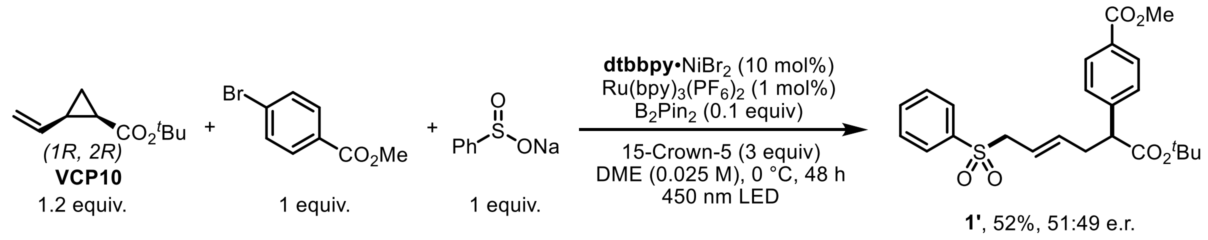

An oven-dried 7.5 mL screw-cap vial equipped with a magnetic stirring bar was charged with $PhSO_2Na$ (16.4 mg, 0.1 mmol, 1 equiv.), methyl 4-bromobenzoate (21.5 mg, 0.1 mmol, 1 equiv.), $Ru(bpy)_3(PF_6)_2$ (0.9 mg, 0.001 mmol, 1 mol%), B_2Pin_2 (2.6 mg, 0.01 mmol, 0.1 equiv.) and **L2**·$NiBr_2$ (8.5 mg, 0.01 mmol, 10 mol%) or **dtbbpy**·$NiBr_2$ complex (4.9 mg, 0.01 mmol, 10 mol%) and then introduced into a nitrogen-filled glovebox. There, dry DME (4 mL) and 15-crown-5 (66 mg, 0.3 mmol, 3 equiv.) were sequentially added. The reaction vessel was then capped and removed from the glovebox. **VCP10** (20.2 mg, 0.12 mmol, 1.2 equiv.) was then added. The reaction was stirred (800 rpm) under irradiation with a 34 W 450 nm LED at 0 °C for 48 h. The reaction was quenched with saturated aq. NaCl (1 mL) and the resulting mixture was extracted with EtOAc (3 × 2mL). The organic phase was concentrated under reduced pressure and the residue purified by column chromatography on silica gel (Hex/EA = 2/1). The enantiomeric ratio (e.r.) and *E*/*Z* ratio of the product were determined by HPLC with a chiral stationary phase.

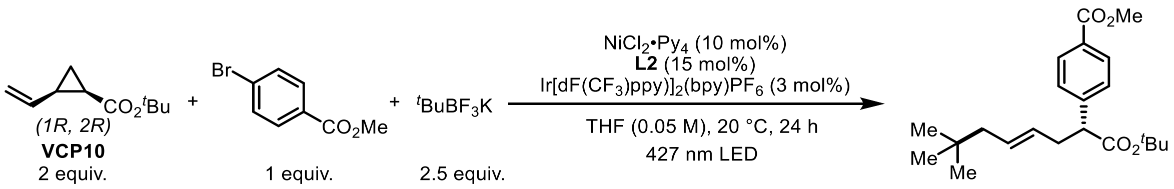

2, 55% (9:1), 93:7 e.r.

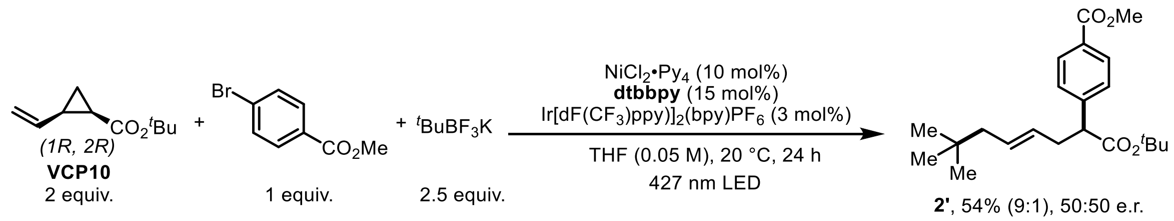

2', 54% (9:1), 50:50 e.r.

An oven-dried 7.5 mL screw-cap vial equipped with a magnetic stirring bar was charged ^{t}BuBF$_3$K (41.0 mg, 0.25 mmol, 2.5 equiv.), methyl 4-bromobenzoate (21.5 mg, 0.1 mmol, 1 equiv.), Ir[dF(CF$_3$)ppy)]$_2$(bpy)PF$_6$ (3.0 mg, 0.003 mmol, 3 mol%), NiCl$_2$·Py$_4$ (4.5 mg, 0.01 mmol, 10 mol%) and **L2** (9.4 mg, 0.015 mmol, 15 mol%) or **dtbbpy** (4.0 mg, 0.015 mmol, 15 mol%) and then introduced into a nitrogen-filled glovebox. Dry THF (2 mL) was added, then the reaction vessel was capped and removed from the glovebox. **VCP10** (33.6 mg, 0.2 mmol, 2 equiv.) was then added. The reaction was stirred (800 rpm) under irradiation with a 45 W 427 nm Kessil LED at 25 °C for 24 h. The reaction was quenched with saturated aq. NaCl (1 mL), and the resulting mixture was extracted with EtOAc (3 × 2 mL). The organic phase was dried over anhydrous sodium sulfate and concentrated under reduced pressure, then the residue was purified by column chromatography on silica gel (Hex/EA = 20/1). The enantiomeric ratio (e.r.) and the *E*/*Z* ratio of the product were determined by HPLC with a chiral stationary phase.

When enantiopure **VCP10** was used as substrate, the multicomponent reaction occurred smoothly under the standard conditions, giving the corresponding remote difunctionalization products **1** and **2** in 65 and 55% yield with 95:5 and 93:7 e.r., respectively. Replacing chiral ligand **L2** with dtbbpy under otherwise identical conditions, the products were generated in similar yields but as the corresponding racemates (**1’** and **2’**). These results indicate that stereocontrol originates with the chiral nickel complex, and that the absolute configuration of the substrate does not affect the stereochemical outcome of the reaction.

5.2 Radical inhibition experiments

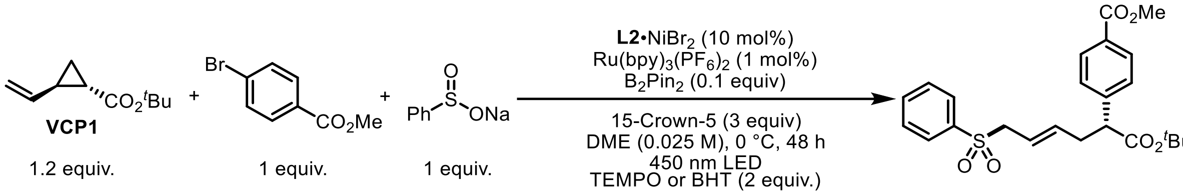

1, ND

An oven-dried 7.5 mL screw-cap vial equipped with a magnetic stirring bar was charged with $PhSO_2Na$ (16.4 mg, 0.1 mmol, 1 equiv.), methyl 4-bromobenzoate (21.5 mg, 0.1 mmol, 1 equiv.), $Ru(bpy)_3(PF_6)_2$ (0.9 mg, 0.001 mmol, 1 mol%), B_2Pin_2 (2.6 mg, 0.01 mmol, 0.1 equiv.), **L2**·$NiBr_2$ complex (8.5 mg, 0.01 mmol, 10 mol%) and TEMPO (31.2 mg, 0.2 mmol, 2 equiv.) or BHT (44.1 mg, 0.2 mmol, 2 equiv.) and then introduced into a nitrogen-filled glovebox. There, dry DME (4 mL) and 15-crown-5 (66 mg, 0.3 mmol, 3 equiv.) were sequentially added. The reaction vessel was then capped and removed from the glovebox. **VCP1** (20.2 mg, 0.12 mmol, 1.2 equiv.) was then added. The reaction was stirred (800 rpm) under irradiation with a 34 W 450 nm LED at 0 °C for 48 h. The reaction mixture was then concentrated under reduced pressure and the residue was analyzed directly by HR-MS.

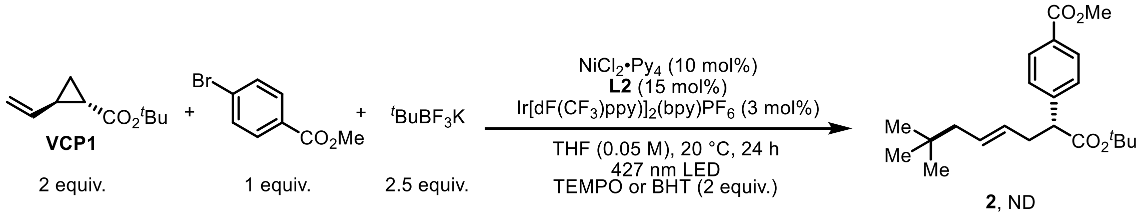

2, ND

An oven-dried 7.5 mL screw-cap vial equipped with a magnetic stirring bar was charged $^tBuBF_3K$ (41.0 mg, 0.25 mmol, 2.5 equiv.), methyl 4-bromobenzoate (21.5 mg, 0.1 mmol, 1 equiv.), $Ir[dF(CF_3)ppy)]_2(bpy)PF_6$ (3.0 mg, 0.003 mmol, 3 mol%), $NiCl_2·Py_4$ (4.5 mg, 0.01 mmol, 10 mol%), **L2** (9.4 mg, 0.015 mmol, 15 mol%) and TEMPO (31.2 mg, 0.2 mmol, 2 equiv.) or BHT (44.1 mg, 0.2 mmol, 2 equiv.) and then introduced into a nitrogen-filled glovebox. Dry THF (2 mL) was added, and the reaction vessel was then capped and removed from the glovebox. **VCP1** (33.6 mg, 0.2 mmol, 2 equiv.) was then added. The reaction was stirred (800 rpm) under irradiation with a 45 W 427 nm Kessil LED at 25 °C for 24 h. The reaction mixture was concentrated under reduced pressure and the residue was analyzed

directly by HR-MS.

Addition of TEMPO or BHT completely inhibited the desired transformation, and in the case of TEMPO, the corresponding alkyl-TEMPO adduct **71** was observed by HR-MS (Figure S2).

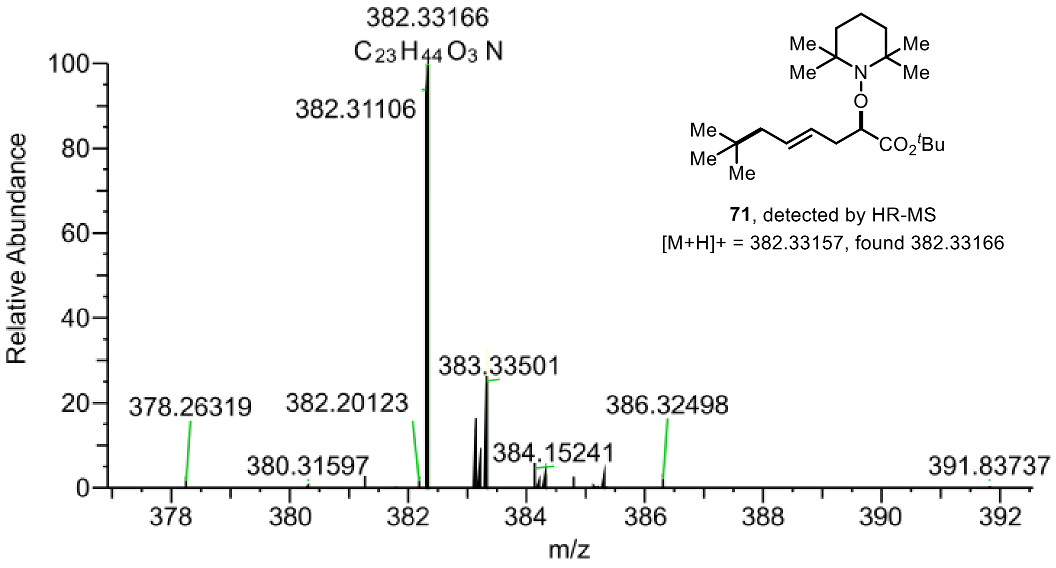

Figure S2. HR-MS for alkyl-TEMPO adduct **71**

5.3 Non-linear effect experiments

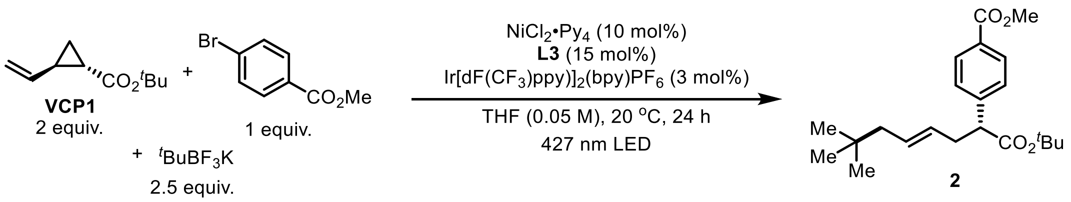

ee of ligand (%)	0	20	40	60	80	100
ee of **2** (%)	2	18	36	53	65	92

The experiments to test for non-linear effects were performed with the general procedure for remote dicarbofunctionalization. **L3** with different levels of enantiomeric purity was prepared by mixing the two enantiomers in the appropriate ratio. A linear relationship was observed between the enantiomeric excess of the ligand and that of the product, indicating a 1:1 binding pattern between **L3** and Ni in the active catalytic species.

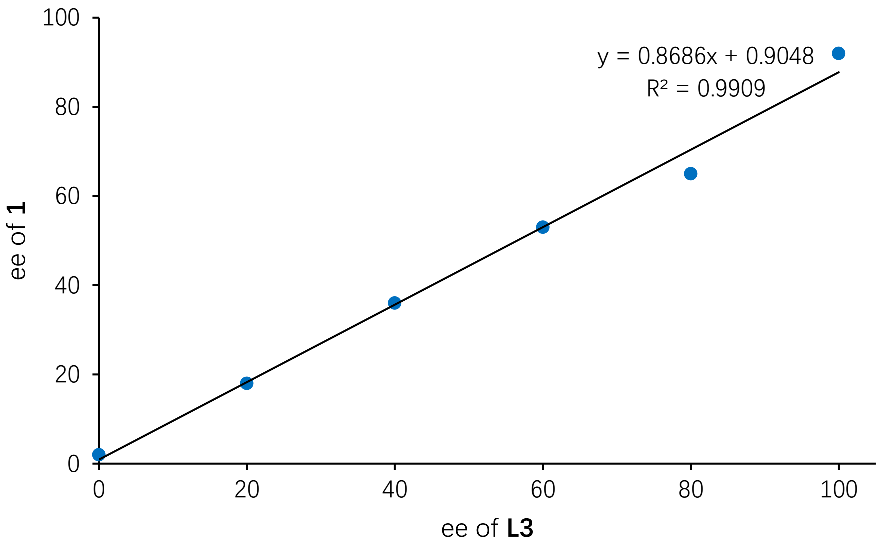

Figure S3. Remote dicarbofunctionalization using **L3** of varying ee. The line corresponds to the least-squares linear regression of the data with slope = 0.87 and R^2 = 0.99.

5.4 Stoichiometric experiments with dtbbpyNi(I)Br complex

Synthesis of dtbbpyNi(I)Br complex[14]

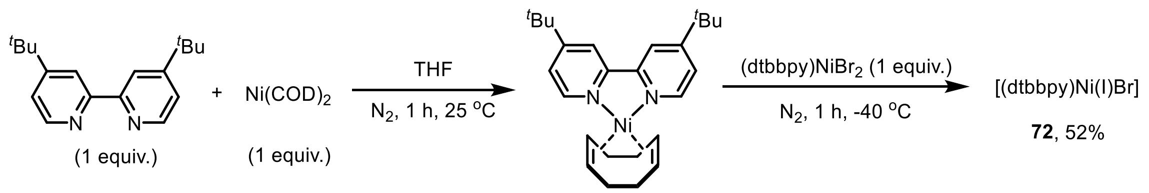

An oven-dried Schlenk flask (A) was charged with $Ni(COD)_2$ (137.5 mg, 0.5 mmol, 1 equiv) inside a N_2-containing glovebox and capped with a septum. The flask was connected to N_2 followed by addition of dtbbpy (134 mg, 0.5 mmol, 1 equiv) and dry THF (5 mL). The purple mixture was stirred for 1 h at 25 ºC. In parallel, an oven dried Schlenk flask (B) was charged with dtbbpy (134 mg, 0.5 mmol, 1 equiv), $NiBr_2 \cdot DME$ (154.5 mg, 0.5 mmol, 1 equiv) and dry THF (5 mL). The green suspension was stirred for 1 h at 25 ºC and was transferred to the previous Schlenk flask (A), which was cooled down at -40 ºC in an acetonitrile-dry ice bath, via cannula. The mixture was stirred for 1 h followed by filtration through a pad of celite. The solution was concentrated and dried under high vacuum to afford a dark brown solid (52% yield, 211 mg). The resulting complex was stored at -33 ºC in the glovebox.

Stoichiometric and catalytic reaction with dtbbpyNi(I)Br complex

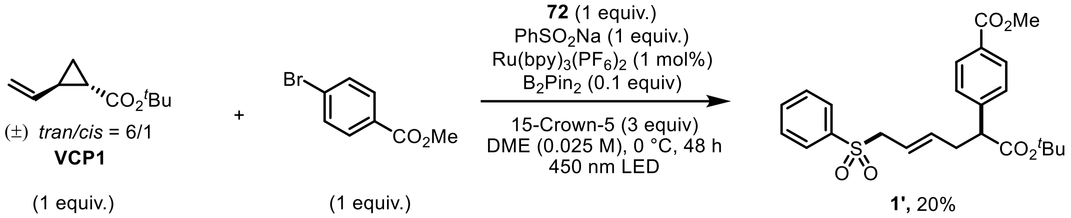

An oven-dried 7.5 mL screw-cap vial equipped with a magnetic stirring bar was charged with $PhSO_2Na$ (4.92 mg, 0.03 mmol, 1 equiv.), methyl 4-bromobenzoate (6.45 mg, 0.03 mmol, 1 equiv.), $Ru(bpy)_3(PF_6)_2$ (0.26 mg, 0.0003 mmol, 1 mol%) and B_2Pin_2 (0.76 mg, 0.003 mmol, 0.1 equiv.) and then introduced into a nitrogen-filled glovebox. There, **72** (17.7 mg, 0.03 mmol, 1 equiv.) was added, followed by dry DME (1.2 mL) and 15-crown-5 (19.8 mg, 0.09 mmol, 3 equiv.). The reaction vessel was then capped and removed from the glovebox. **VCP1** (5.05 mg, 0.03 mmol, 1 equiv.) was then added. The reaction was stirred (800 rpm) under irradiation with a 450 nm LED lamp at 0 °C for 48 h. The reaction mixture was then concentrated under reduced pressure and the residue analyzed by ^1H NMR with 1,3,5-trimethoxybenzene (4.33 mg, 0.036 mmol) as internal standard. The crude ^1H NMR showed that product **1'** was generated in 20% yield.

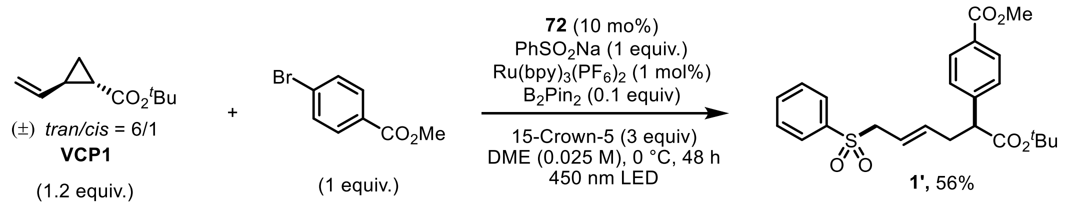

An oven-dried 7.5 mL screw-cap vial equipped with a magnetic stirring bar was charged with $PhSO_2Na$ (8.2 mg, 0.05 mmol, 1 equiv.), methyl 4-bromobenzoate (10.8 mg, 0.05 mmol, 1 equiv.), $Ru(bpy)_3(PF_6)_2$ (0.43 mg, 0.0005 mmol, 1 mol%), B_2Pin_2 (1.3 mg, 0.005 mmol, 0.1 equiv.) and **72** (2.03 mg, 0.005 mmol, 10 mol%) and then introduced into a nitrogen-filled glovebox. There, dry DME (2 mL) and 15-crown-5 (33 mg, 0.15 mmol, 3 equiv.) were sequentially added. The reaction vessel was then capped and removed from the glovebox. **VCP1** (10.1 mg, 0.06 mmol, 1.2 equiv.) was then added. The reaction was stirred (800 rpm) under irradiation with a 34 W 450 nm LED at 0 °C for 48 h. The reaction mixture was then concentrated under reduced pressure and the residue analyzed by ^1H NMR with 1,3,5-trimethoxybenzene (4.33 mg, 0.036 mmol) as internal standard. The crude ^1H NMR of the

catalytic experiment showed that product **1'** was formed in 56% yield.

Taken together, these results implicate Ni(I) species as possible active catalysts and support a Ni(0)-Ni(I)-Ni(III) catalytic cycle.

5.5 Stoichiometric experiments with ArNi(II)Br complex

Synthesis of Ar-Ni(II)-Br complex[15]

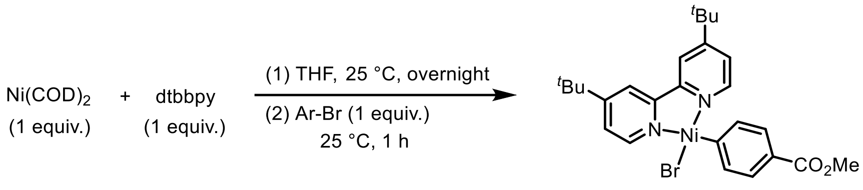

73

In a nitrogen-filled glovebox, a suspension of Ni(COD)$_2$ (82.5 mg, 0.3 mmol, 1 equiv.) in 3 mL dry THF was stirred for 1 min in an oven-dried 20 mL screw-cap vial, at which point a solution of dtbbpy (80.5 mg, 0.3 mmol, 1 equiv.) in dry THF (2 mL) was added dropwise. The resulting mixture was stirred overnight at 25 °C. Methyl 4-bromobenzoate (645 mg, 3 mmol, 10 equiv.) was dissolved in dry THF (2 mL) and then slowly added to the solution via syringe. The resulting mixture was stirred for a further 1 h. The solvent was removed under reduced pressure, and the residue was collected by filtration over a fritted funnel. The solid was washed with ether (3 × 3 mL) and pentane (3 × 3 mL) and dried under reduced pressure. Complex **73** was obtained as orange-red solid in 63% yield (112 mg), which was stored in a nitrogen-filled glovebox at -20 °C. ^{1}H NMR (400 MHz, CD$_2$Cl$_2$) δ 9.10 (d, *J* = 6.3 Hz, 1H), 7.73 (d, *J* = 11.3 Hz, 2H), 7.63 (d, *J* = 7.9 Hz, 2H), 7.48 – 7.33 (m, 3H), 6.98 (dd, *J* = 28.8, 6.1 Hz, 2H), 3.73 (s, 3H), 1.30 (s, 9H), 1.23 (s, 9H).

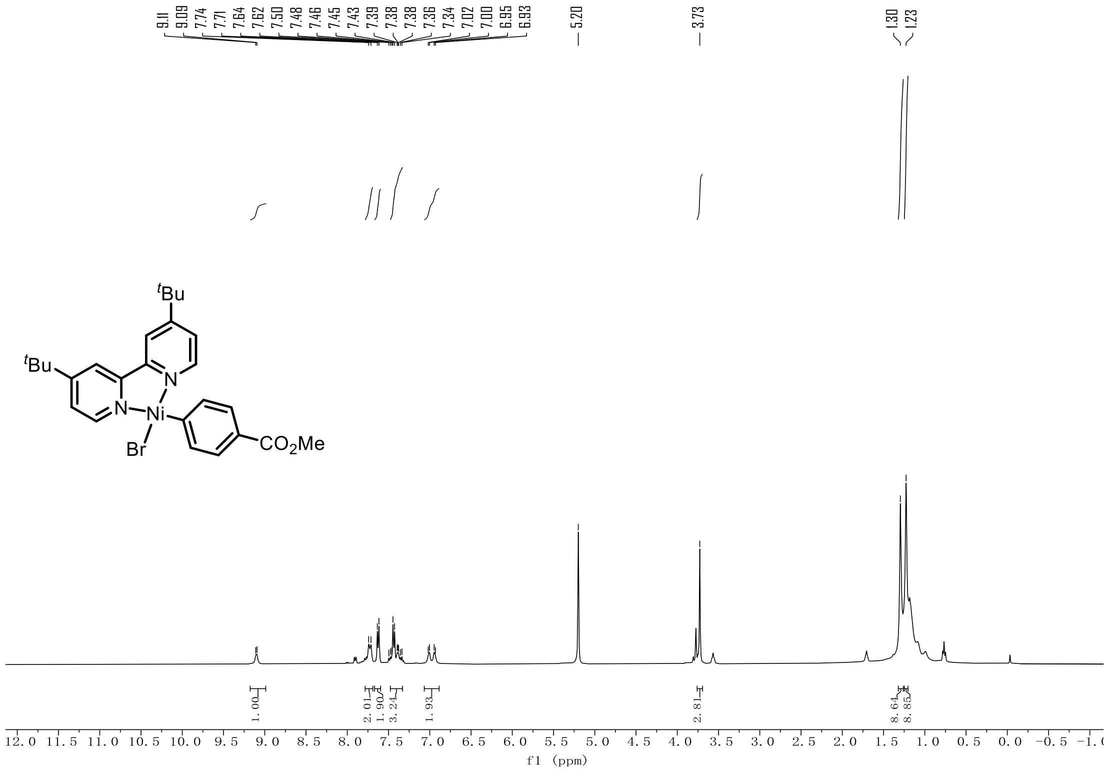

Figure S4. ^{1}H NMR (400 MHz, $CDCl_3$, 298 K) spectrum of **73**

Stoichiometric reaction with Ar-Ni(II)-Br complex

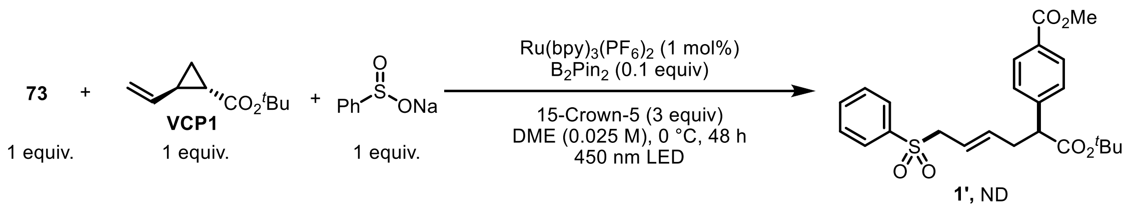

An oven-dried 7.5 mL screw-cap vial equipped with a magnetic stirring bar was charged with $PhSO_2Na$ (4.92 mg, 0.03 mmol, 1 equiv.), $Ru(bpy)_3(PF_6)_2$ (0.26 mg, 0.0003 mmol, 1 mol%) and B_2Pin_2 (0.76 mg, 0.003 mmol, 0.1 equiv.) and then introduced into a nitrogen-filled glovebox. There, **73** (17.7 mg, 0.03 mmol, 1 equiv.) was added, followed by dry DME (1.2 mL) and 15-crown-5 (19.8 mg, 0.09 mmol, 3 equiv.). The reaction vessel was then capped and removed from the glovebox. **VCP1** (5.01 mg, 0.03 mmol, 1 equiv.) was then added. The reaction was stirred (800 rpm) under irradiation with a 450 nm LED lamp at 0 °C for 48 h. The reaction mixture was then concentrated under reduced pressure and the residue analyzed by ^{1}H NMR with 1,3,5-trimethoxybenzene (4.33 mg, 0.036 mmol) as internal standard. The crude ^{1}H NMR showed no product **1'**.

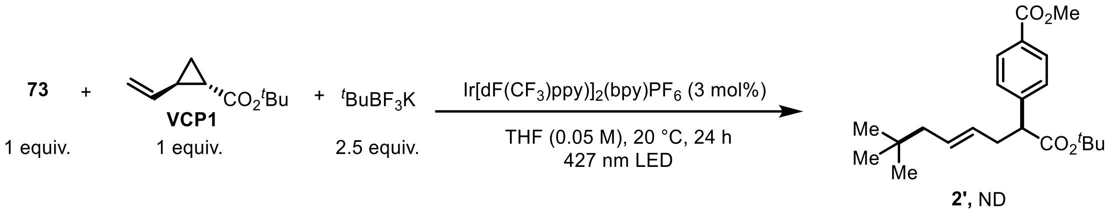

An oven-dried 7.5 mL screw-cap vial equipped with a magnetic stirring bar was charged with ^{t}BuBF$_3$K (5.72 mg, 0.03 mmol, 1 equiv.) and Ir[dF(CF$_3$)ppy)]$_2$(bpy)PF$_6$ (0.91, 0.0003 mol, 3 mol%), and then introduced into a nitrogen-filled glovebox. There, **73** (17.7 mg, 0.03 mmol, 1 equiv.) was added, followed by dry THF (1.2 mL). The reaction vessel was then capped and removed from the glovebox. **VCP1** (5.01mg, 0.03 mmol, 1 equiv.) was then added. The reaction was stirred (800 rpm) under irradiation with a 427 nm Kessil lamp at 20 °C for 24 h. The reaction mixture was then concentrated under reduced pressure and the residue was analyzed by ^{1}H NMR with 1,3,5-trimethoxybenzene (4.33 mg, 0.036 mmol) as internal standard. The crude ^{1}H NMR showed no product **2'**.

There results revealed that a Ni(II)–aryl species is likely not a competent intermediate in the catalytic cycle, making a Ni(0)/Ni(II)/Ni(III) catalytic cycle unlikely.

5.6 Catalytic and cross-over experiments with ArNi(II)Br complex

catalytic experiment:

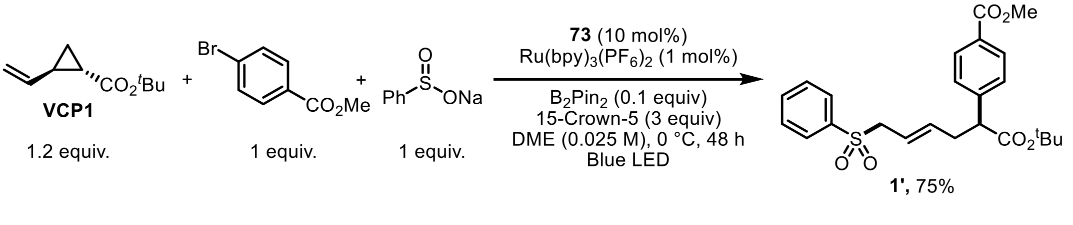

cross-over experiment:

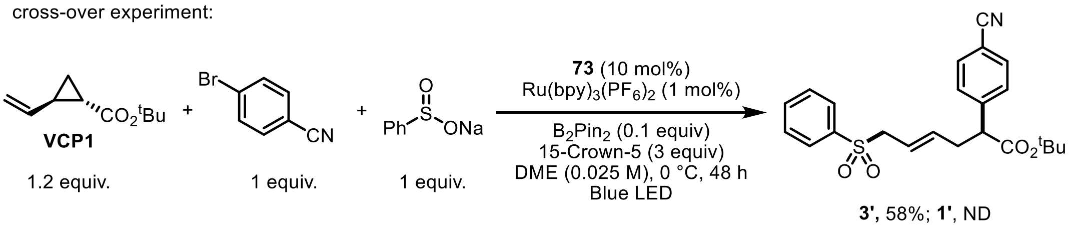

An oven-dried 7.5 mL screw-cap vial equipped with a magnetic stirring bar was charged with $PhSO_2Na$ (16.4 mg, 0.1 mmol, 1 equiv.), aryl bromide (0.1 mmol, 1 equiv.), Ru(bpy)$_3$(PF$_6$)$_2$ (0.9 mg, 0.001 mmol, 1 mol%), B$_2$Pin$_2$ (2.6 mg, 0.01 mmol, 0.1 equiv.) and **73** (5.9 mg, 0.01 mmol, 10 mol%) and then introduced into a nitrogen-filled glovebox. There, dry

DME (4 mL) and 15-crown-5 (0.3 mmol, 3 equiv.) were sequentially added. The reaction vessel was then capped and removed from the glovebox. **VCP1** (20.2 mg, 0.12 mmol, 1.2 equiv.) was then added. The reaction was stirred (800 rpm) under irradiation with a 34 W 450 nm LED at 0 °C for 48 h. The reaction mixture was then concentrated under reduced pressure and the residue was analyzed by ^{1}H NMR with 1,3,5-trimethoxybenzene (4.33 mg, 0.036 mmol) as internal standard. The crude ^{1}H NMR of the catalytic experiment showed product **1’** in 75% yield. The crude ^{1}H NMR of the cross-over experiment showed product **3’** in 58% yield whereas no product **1’** was observed.

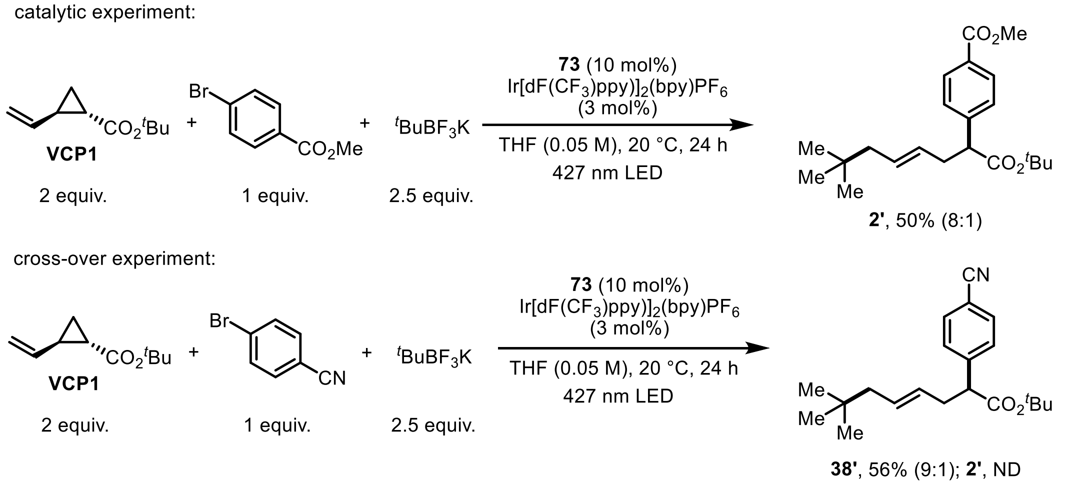

An oven-dried 7.5 mL screw-cap vial equipped with a magnetic stirring bar was charged ^{t}BuBF$_3$K (20.5 mg, 0.125 mmol, 2.5 equiv.), aryl bromide (0.05 mmol, 1 equiv.), Ir[dF(CF$_3$)ppy)]$_2$(bpy)PF$_6$ (1.5 mg, 0.0015 mmol, 3 mol%) and **73** (2.9 mg, 0.005 mmol, 10 mol%) and then introduced into a nitrogen-filled glovebox. Dry THF (1 mL) was added, then the reaction vessel was then capped and removed from the glovebox. **VCP1** (16.8 mg, 0.1 mmol, 2 equiv.) was then added. The reaction was stirred (800 rpm) under irradiation with a 45 W 427 nm Kessil LED at 25 °C for 24 h. The reaction mixture was then concentrated under reduced pressure and the residue was analyzed by ^{1}H NMR with 1,3,5-trimethoxybenzene (4.33mg, 0.036 mmol) as internal standard. The crude ^{1}H NMR of the catalytic experiment showed product **2’** in 50% yield. The crude ^{1}H NMR of the cross-over experiment showed product **38’** in 56% yield whereas no product **2’** was observed.

Taken together, these experiments suggest that, if formed, Ar-Ni(II) complex **73** serves to generate a low-valent nickel(I) species that is competent in the catalytic cycle.

6. NMR spectra

(3*s*,5*s*,7*s*)-Adamantan-1-yl 2-vinylcyclopropane-1-carboxylate (VCP2)

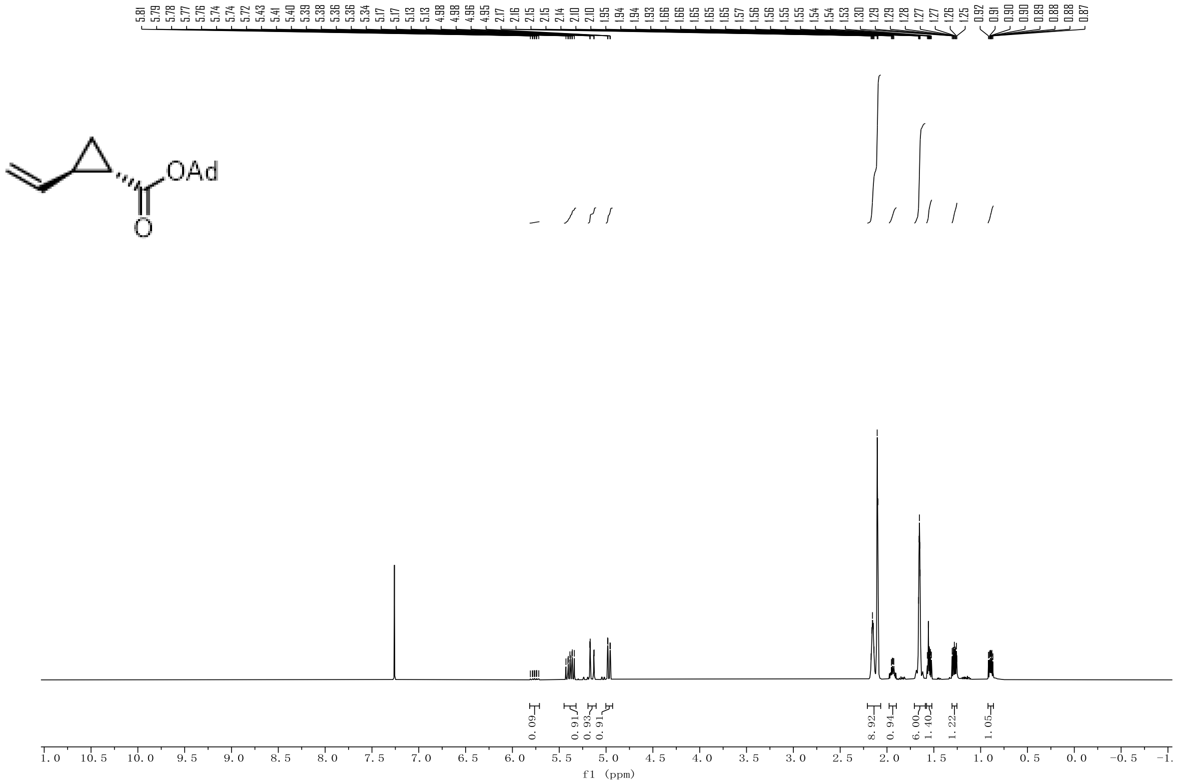

^{1}H NMR (400 MHz, $CDCl_3$, 298 K) spectrum of **VCP2**

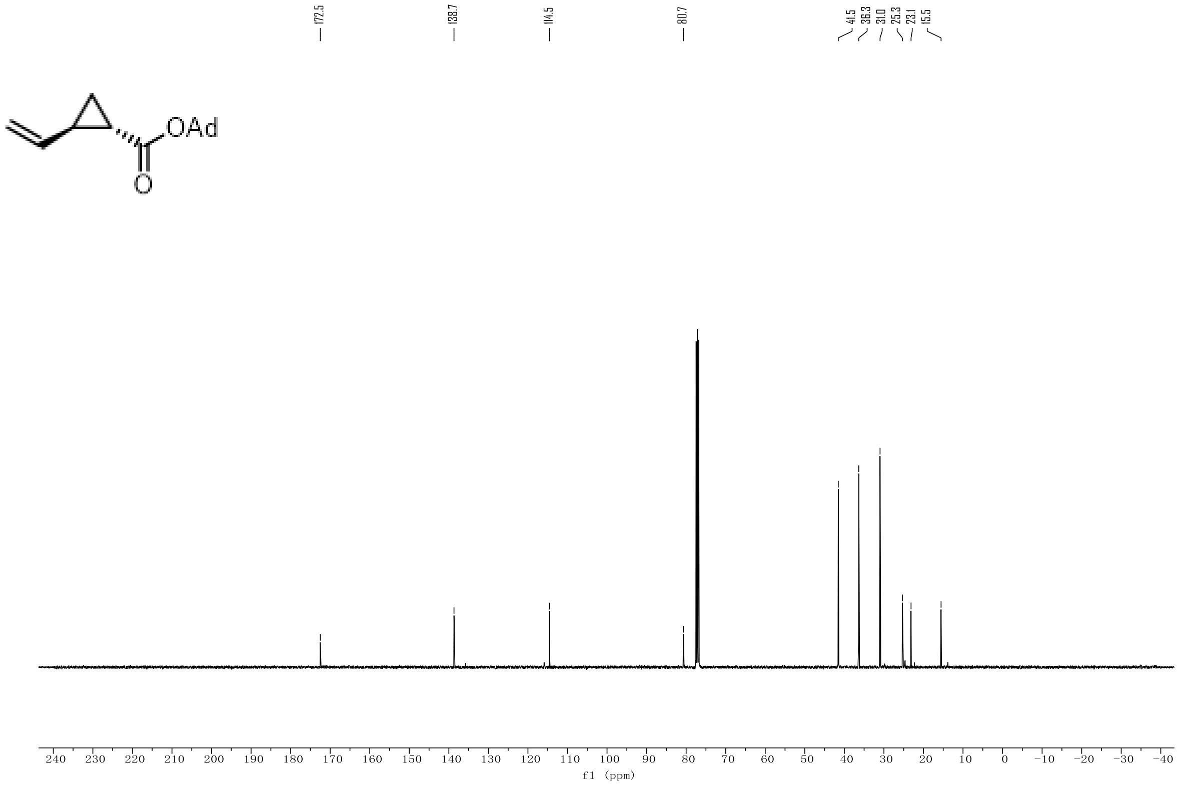

^{13}C NMR (101 MHz, $CDCl_3$, 298 K) spectrum of **VCP2**

1-Methylcyclohexyl 2-vinylcyclopropane-1-carboxylate (VCP3)

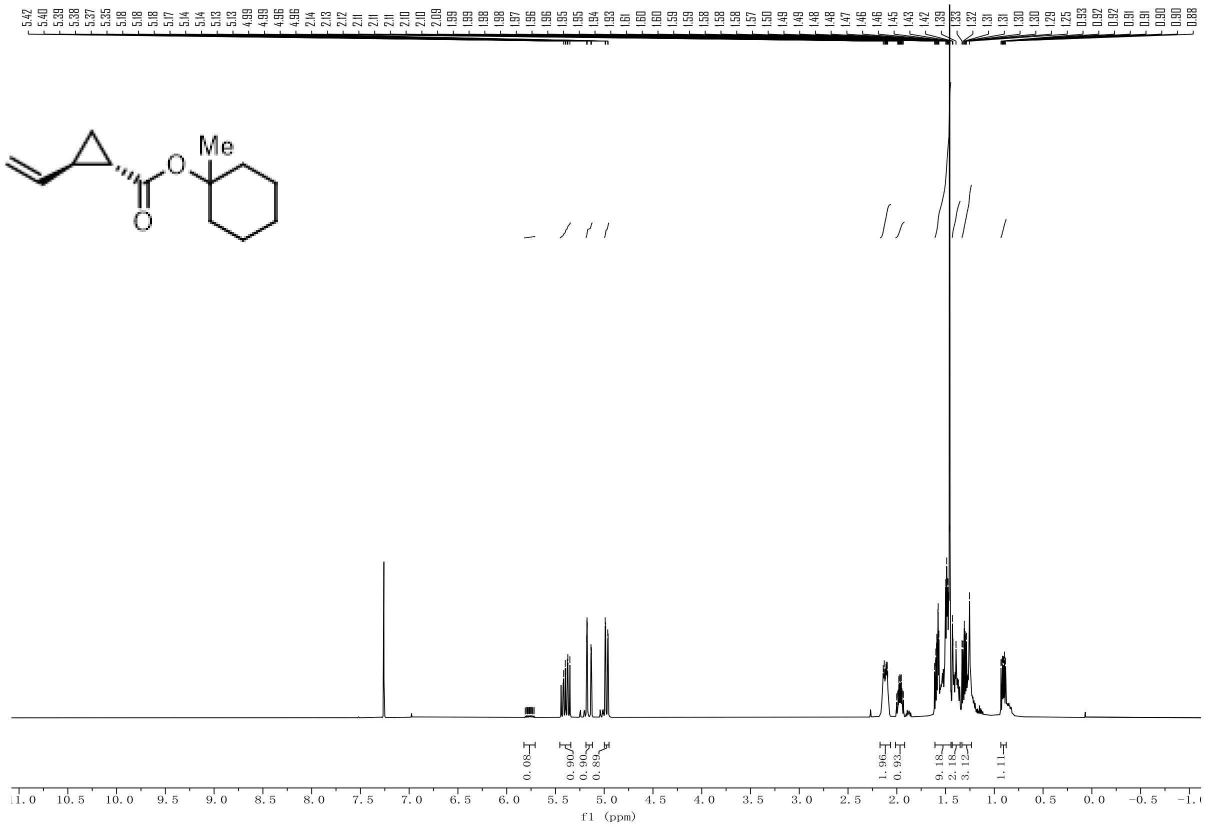

^{1}H NMR (400 MHz, $CDCl_3$, 298 K) spectrum of **VCP3**

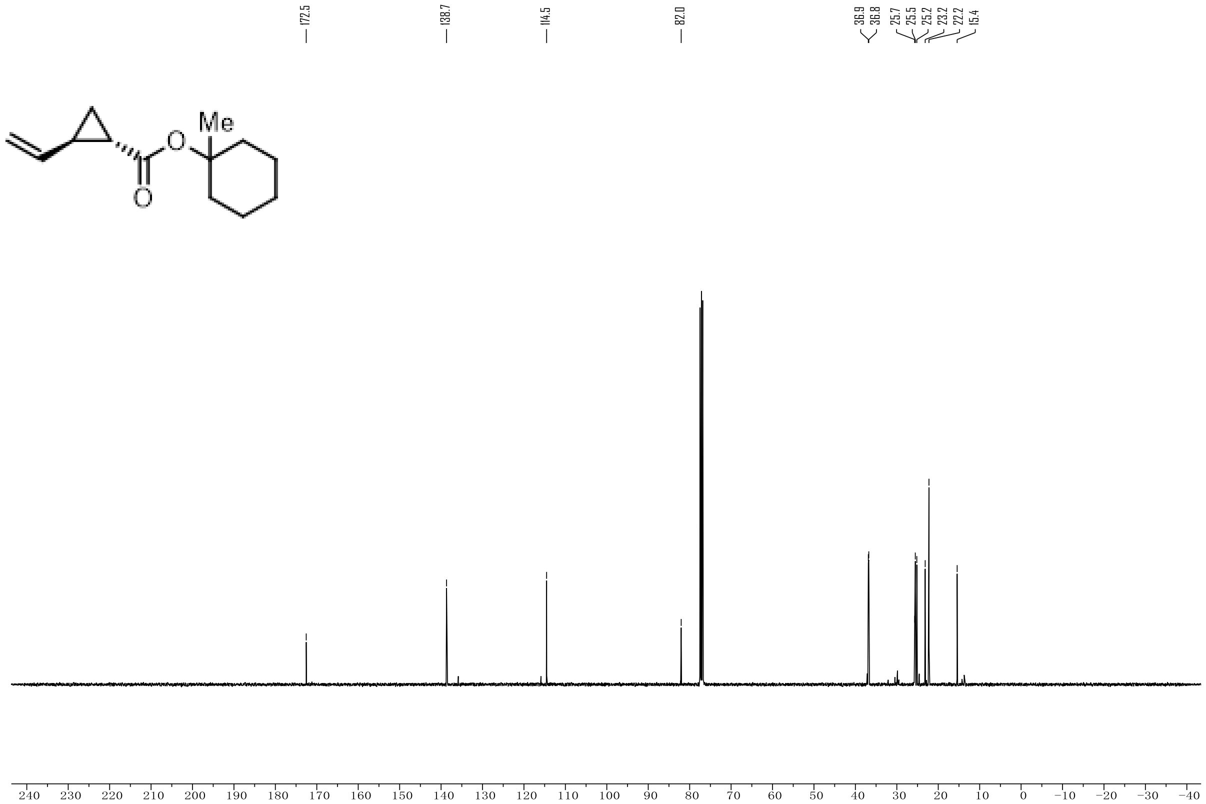

^{13}C NMR (101 MHz, $CDCl_3$, 298 K) spectrum of **VCP3**

2-Methyl-4-phenylbutan-2-yl 2-vinylcyclopropane-1-carboxylate (VCP4)

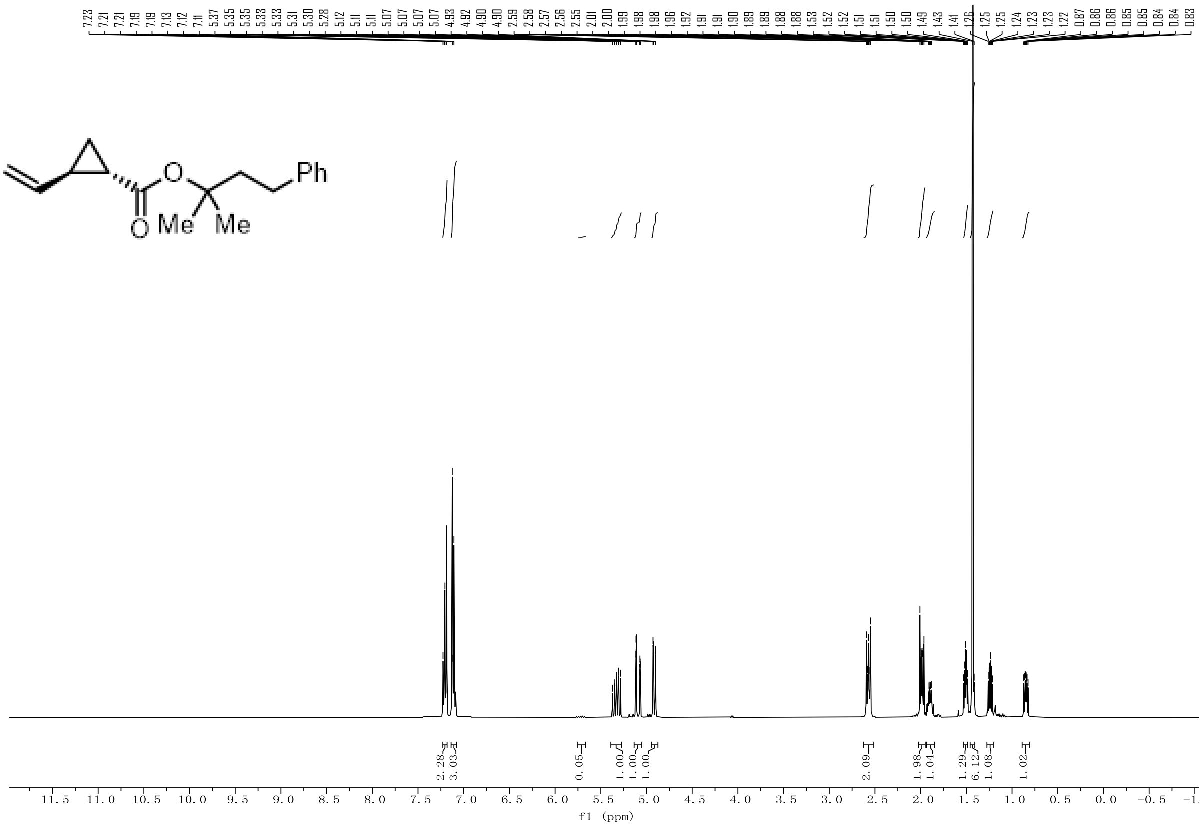

^{1}H NMR (400 MHz, $CDCl_3$, 298 K) spectrum of **VCP4**

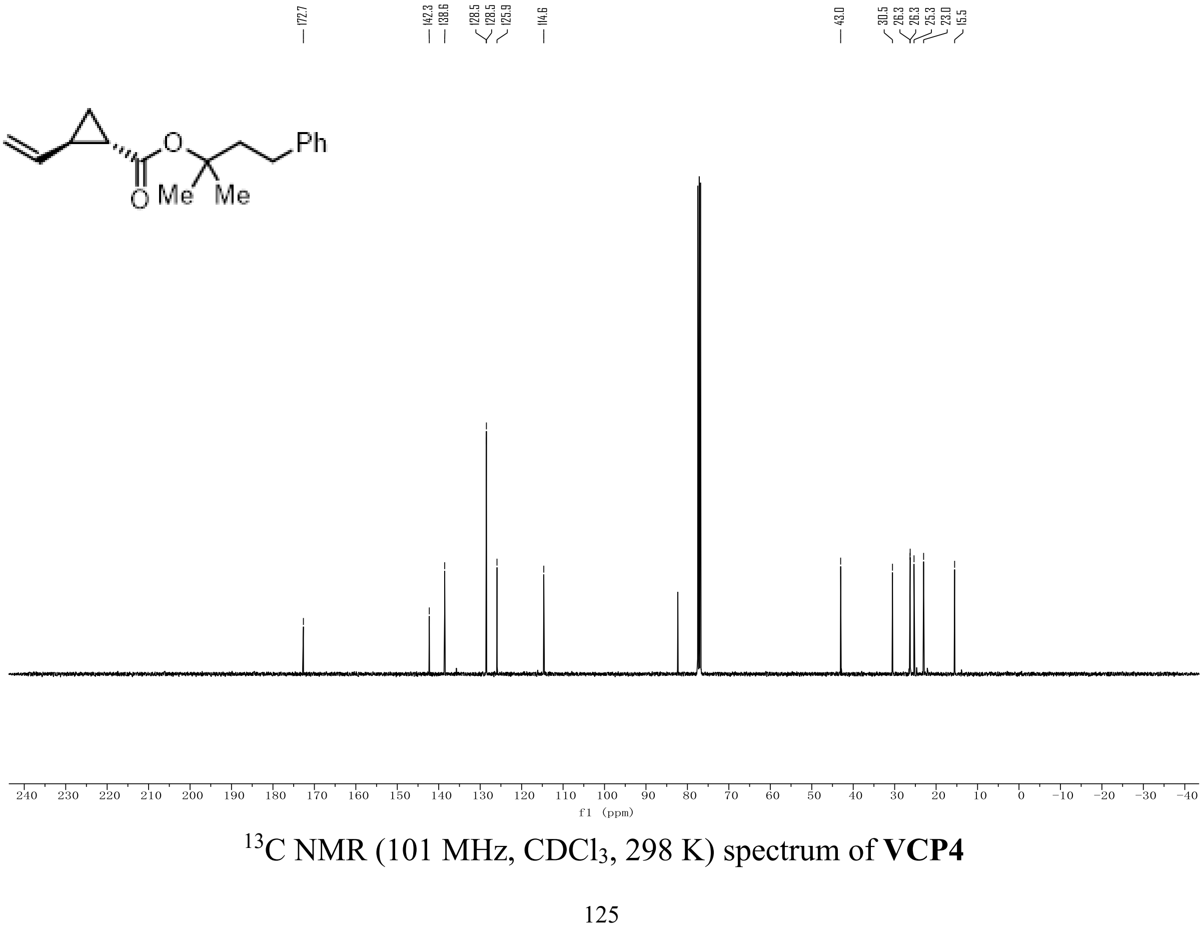

^{13}C NMR (101 MHz, $CDCl_3$, 298 K) spectrum of **VCP4**

2,4-Dimethylpentan-3-yl 2-vinylcyclopropane-1-carboxylate (VCP5)

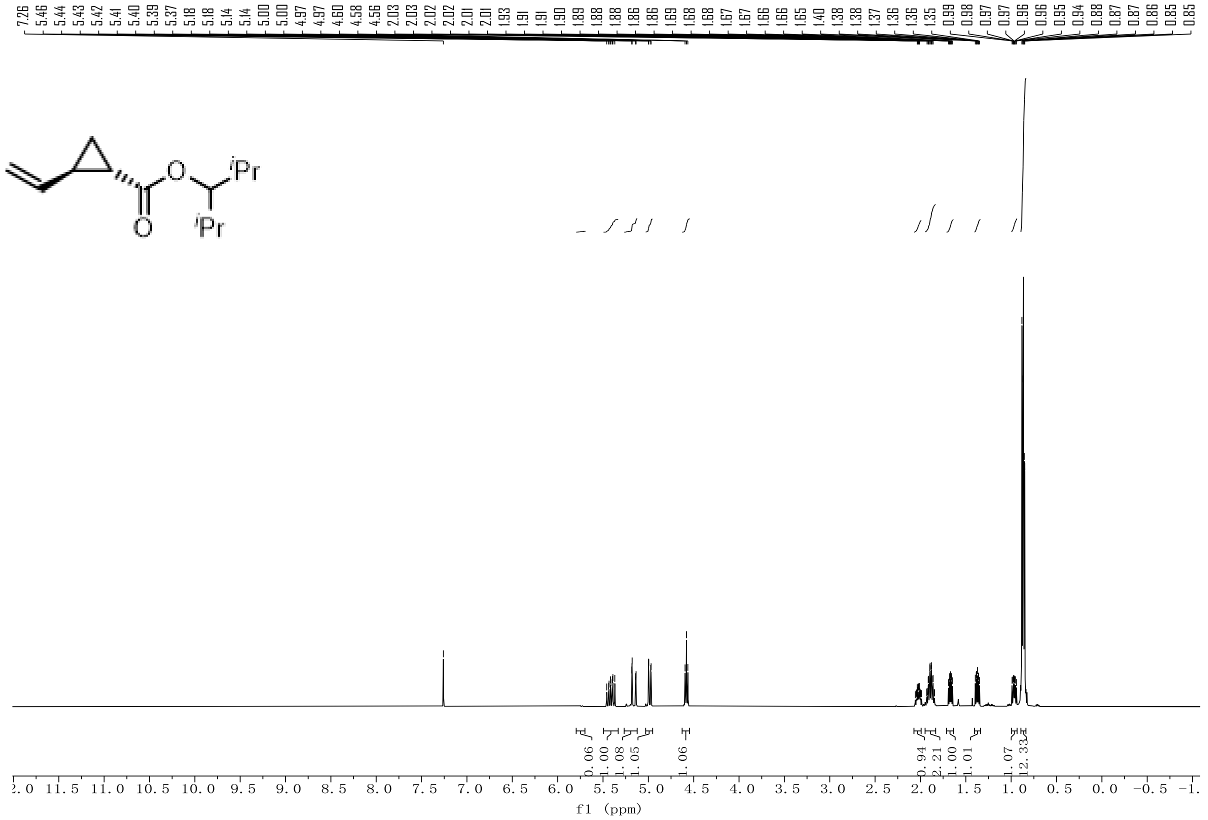

^{1}H NMR (400 MHz, CDCl$_3$, 298 K) spectrum of **VCP5**

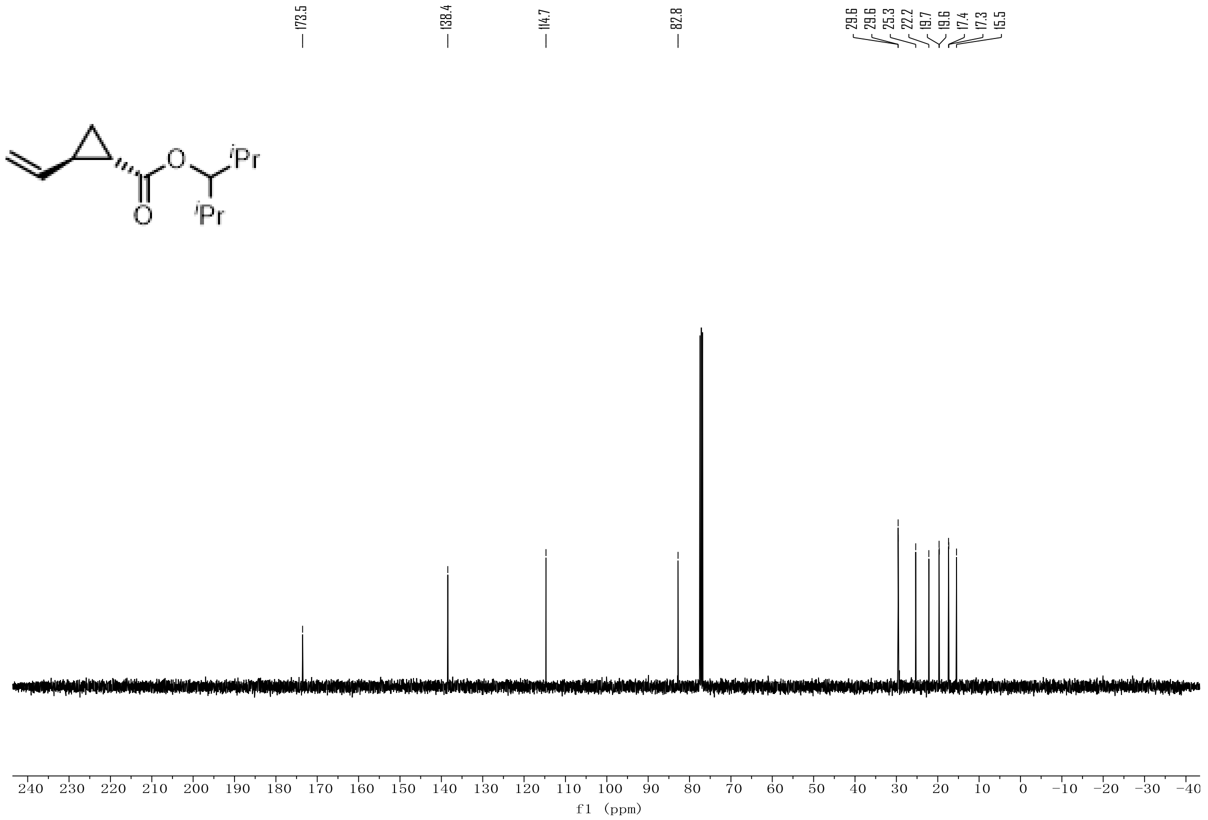

^{13}C NMR (101 MHz, CDCl$_3$, 298 K) spectrum of **VCP5**

N-(*tert*-Butyl)-2-vinylcyclopropane-1-carboxamide (VCP6)

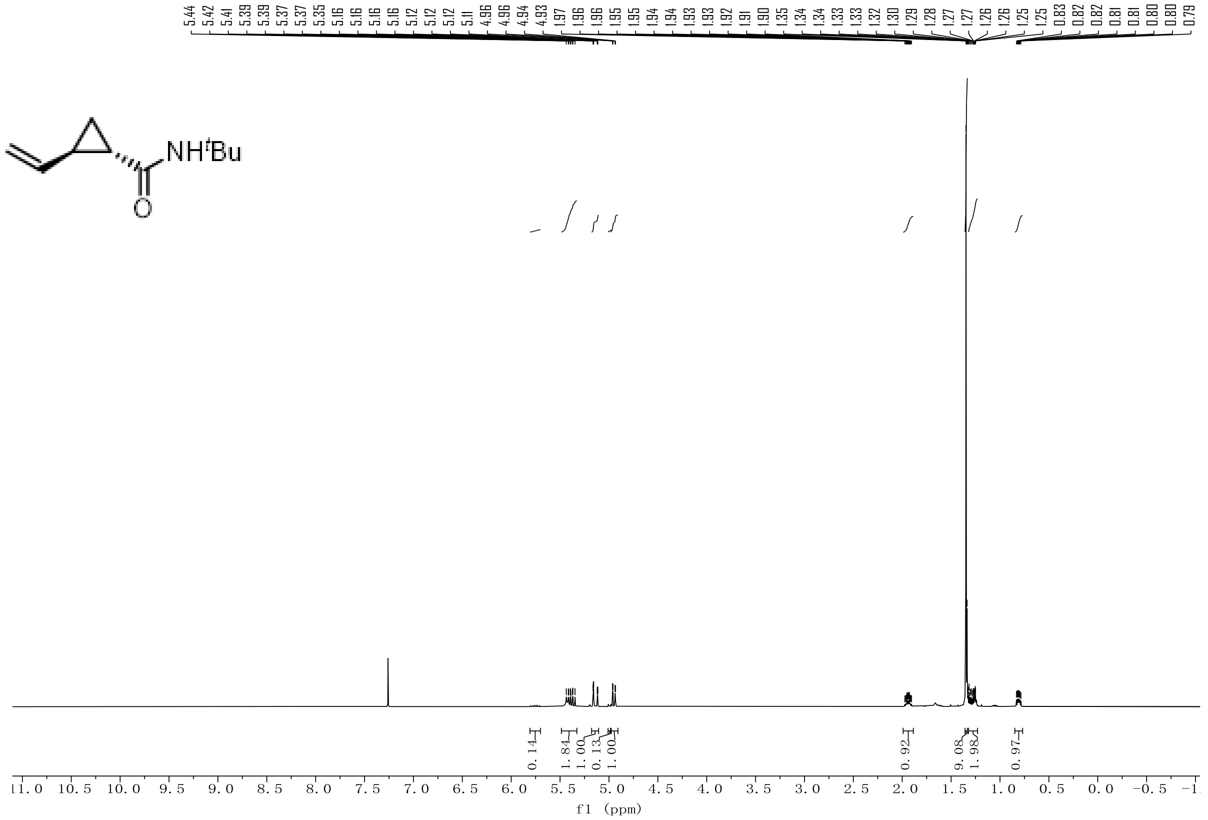

^{1}H NMR (400 MHz, $CDCl_3$, 298 K) spectrum of **VCP6**

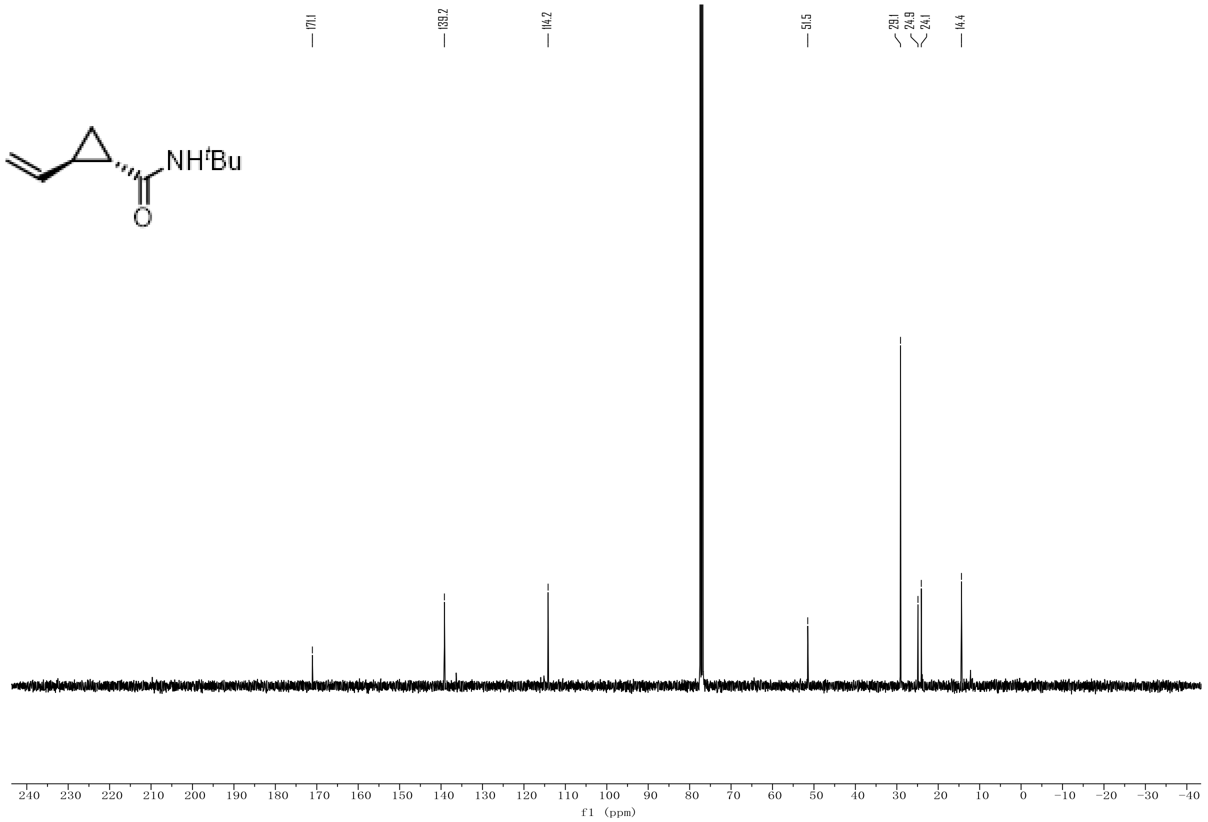

^{13}C NMR (101 MHz, $CDCl_3$, 298 K) spectrum of **VCP6**

tert-Butyl 2-methyl-2-vinylcyclopropane-1-carboxylate (VCP7)

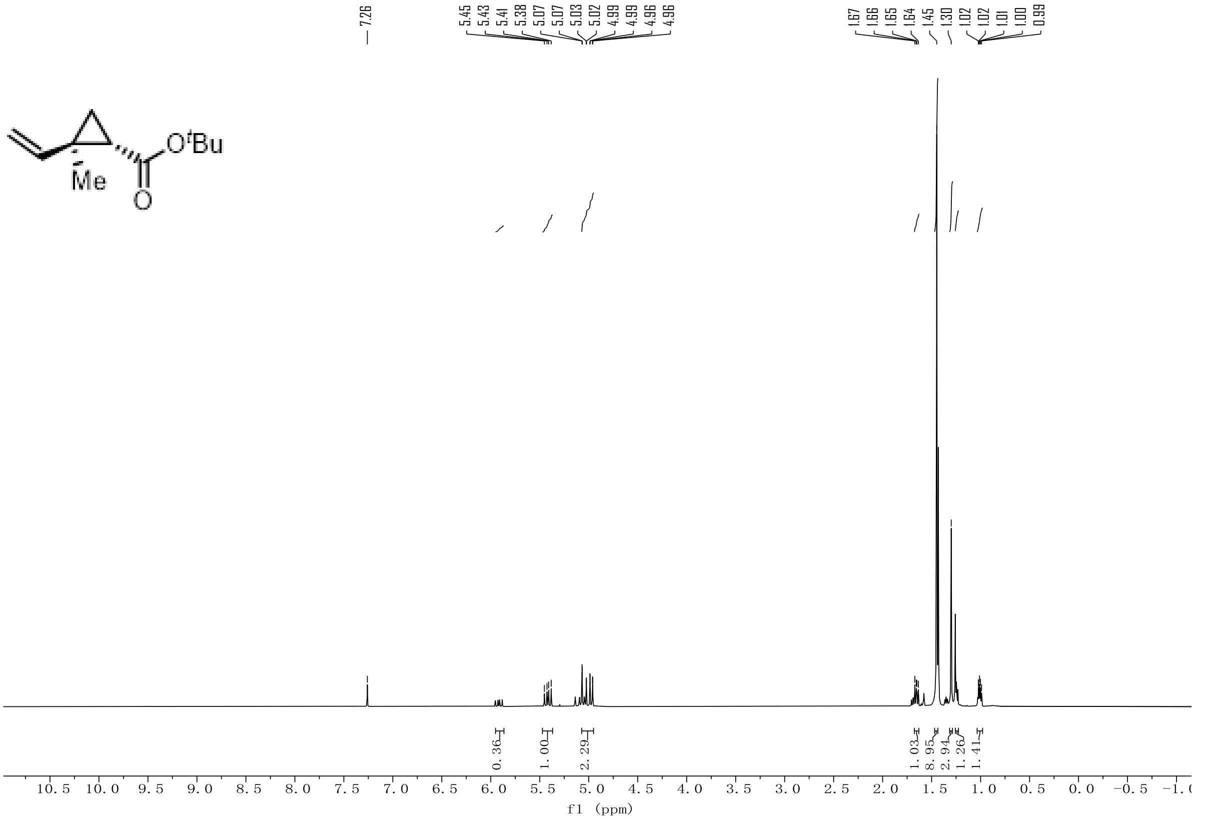

^{1}H NMR (400 MHz, $CDCl_3$, 298 K) spectrum of **VCP7**

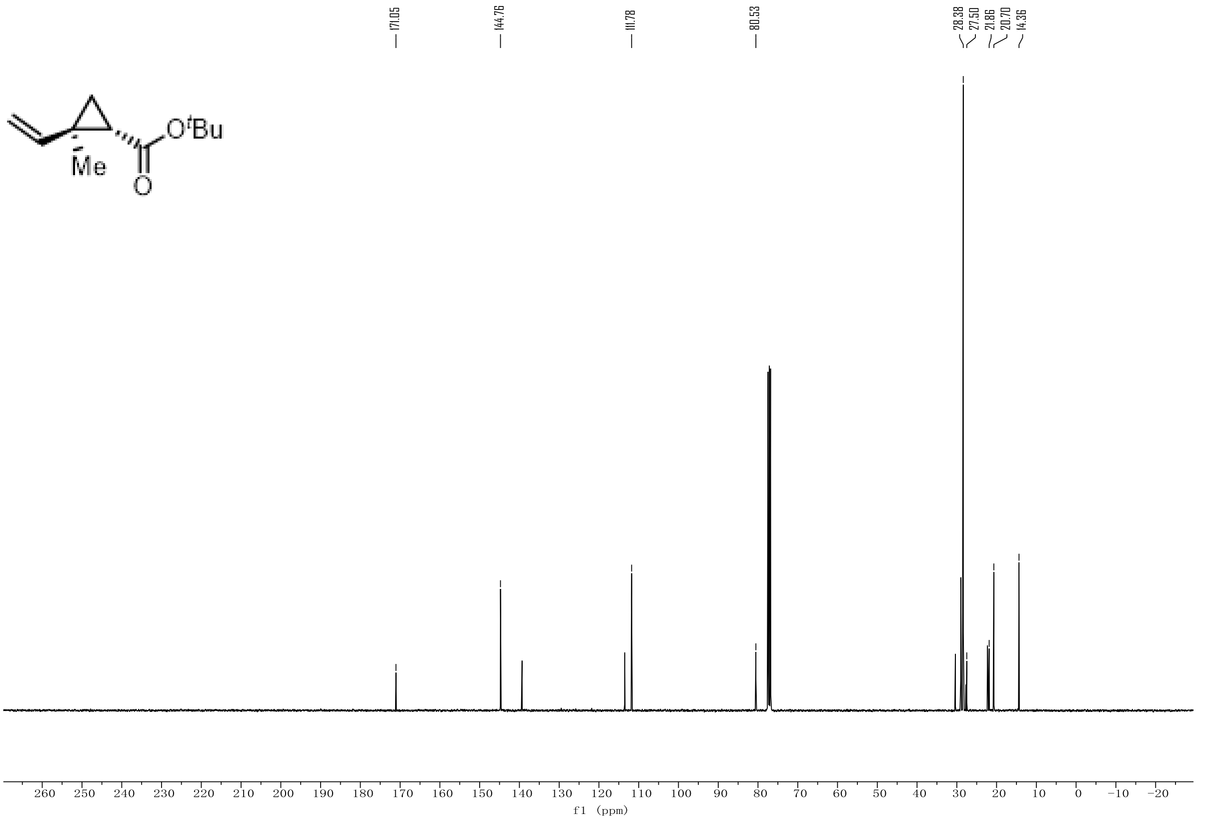

^{13}C NMR (101 MHz, $CDCl_3$, 298 K) spectrum of **VCP7**

tert-Butyl 2-(prop-1-en-2-yl)cyclopropane-1-carboxylate (VCP8)

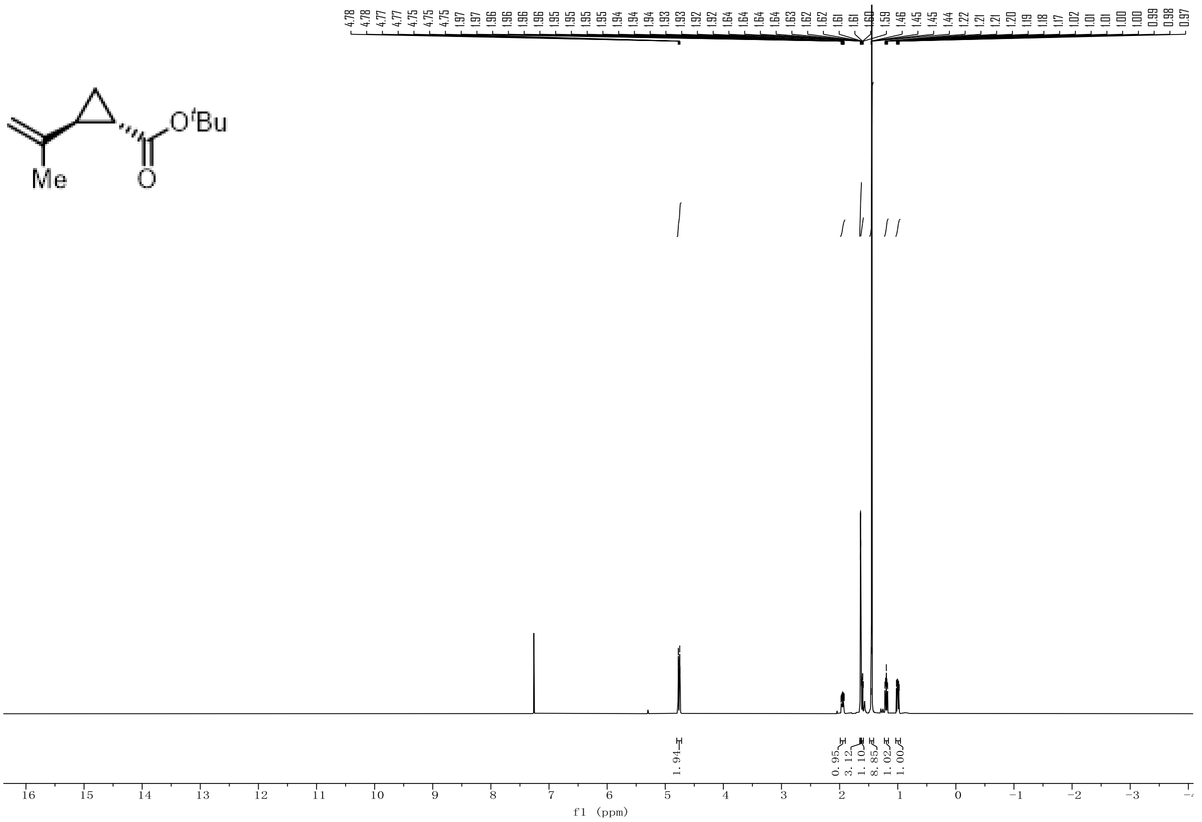

^{1}H NMR (400 MHz, $CDCl_3$, 298 K) spectrum of **VCP8**

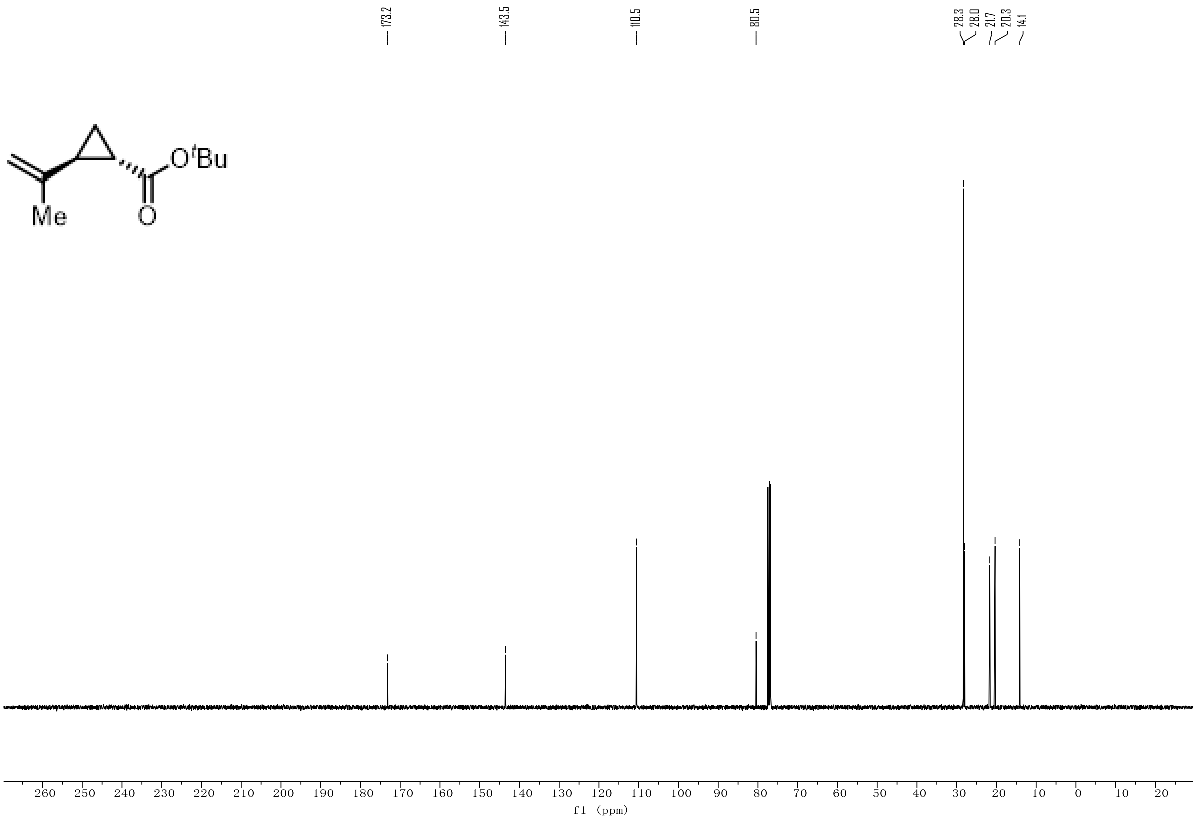

^{13}C NMR (101 MHz, $CDCl_3$, 298 K) spectrum of **VCP8**

***tert*-Butyl (1*R*, 2*R*)-2-vinylcyclopropane-1-carboxylate (VCP10)**

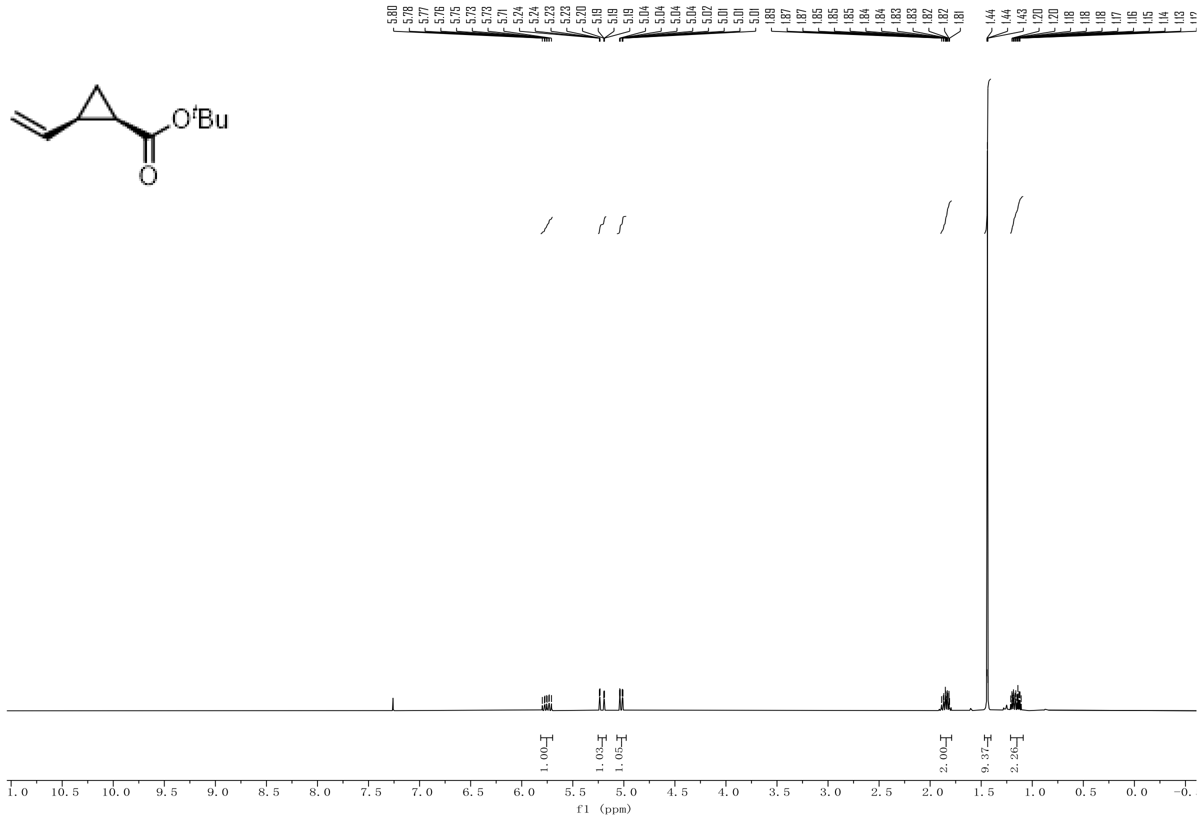

^{1}H NMR (400 MHz, $CDCl_3$, 298 K) spectrum of **VCP10**

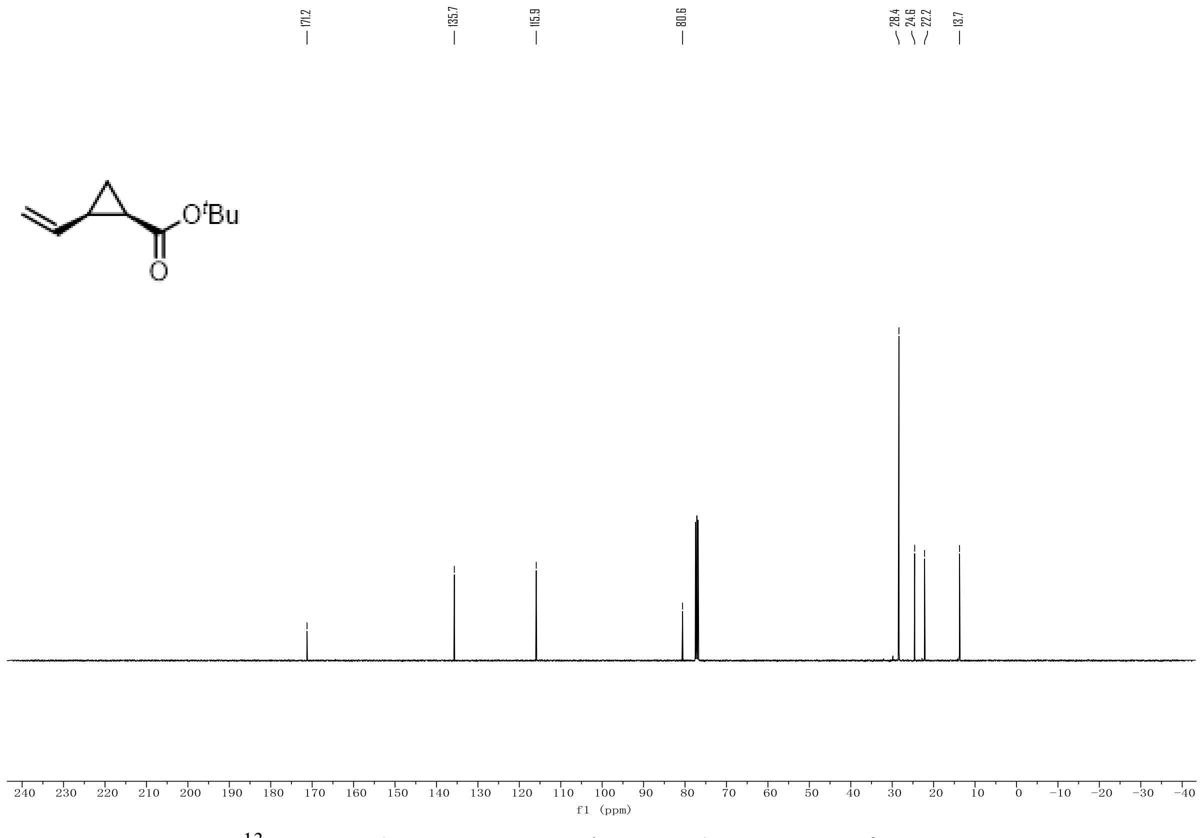

^{13}C NMR (101 MHz, $CDCl_3$, 298 K) spectrum of **VCP10**

tert-Butyl 2-phenyl-2-vinylcyclopropane-1-carboxylate (VCP11)

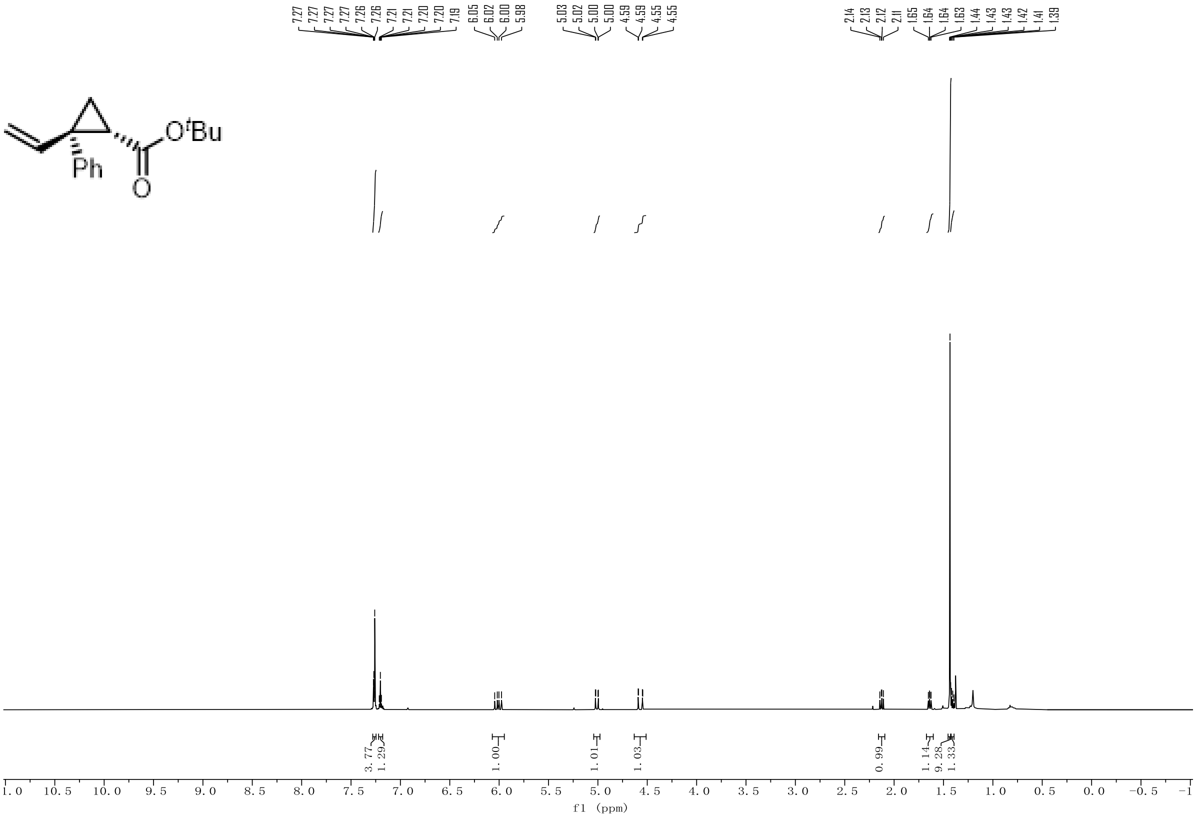

^{1}H NMR (400 MHz, $CDCl_3$, 298 K) spectrum of **VCP11**

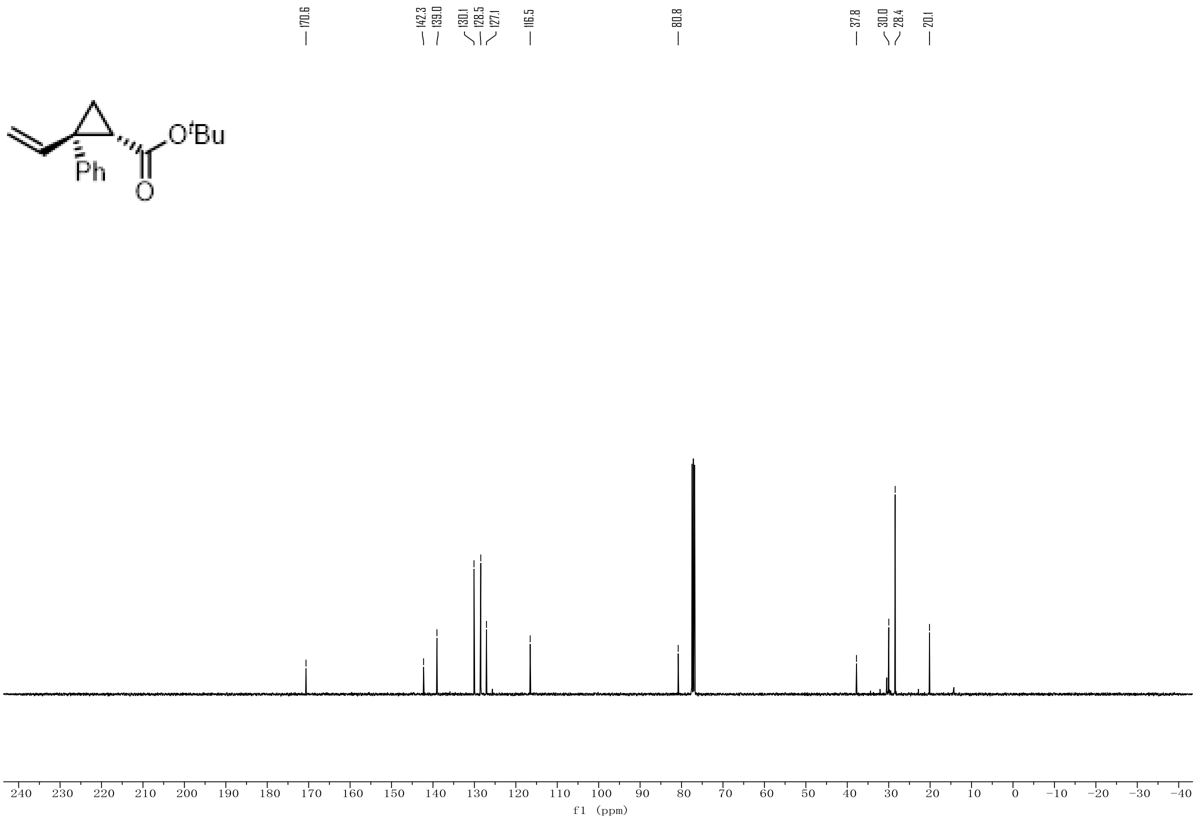

^{13}C NMR (101 MHz, $CDCl_3$, 298 K) spectrum of **VCP11**

1-chloro-4-(2-(prop-1-en-2-yl)cyclopropyl)benzene (VCP15)

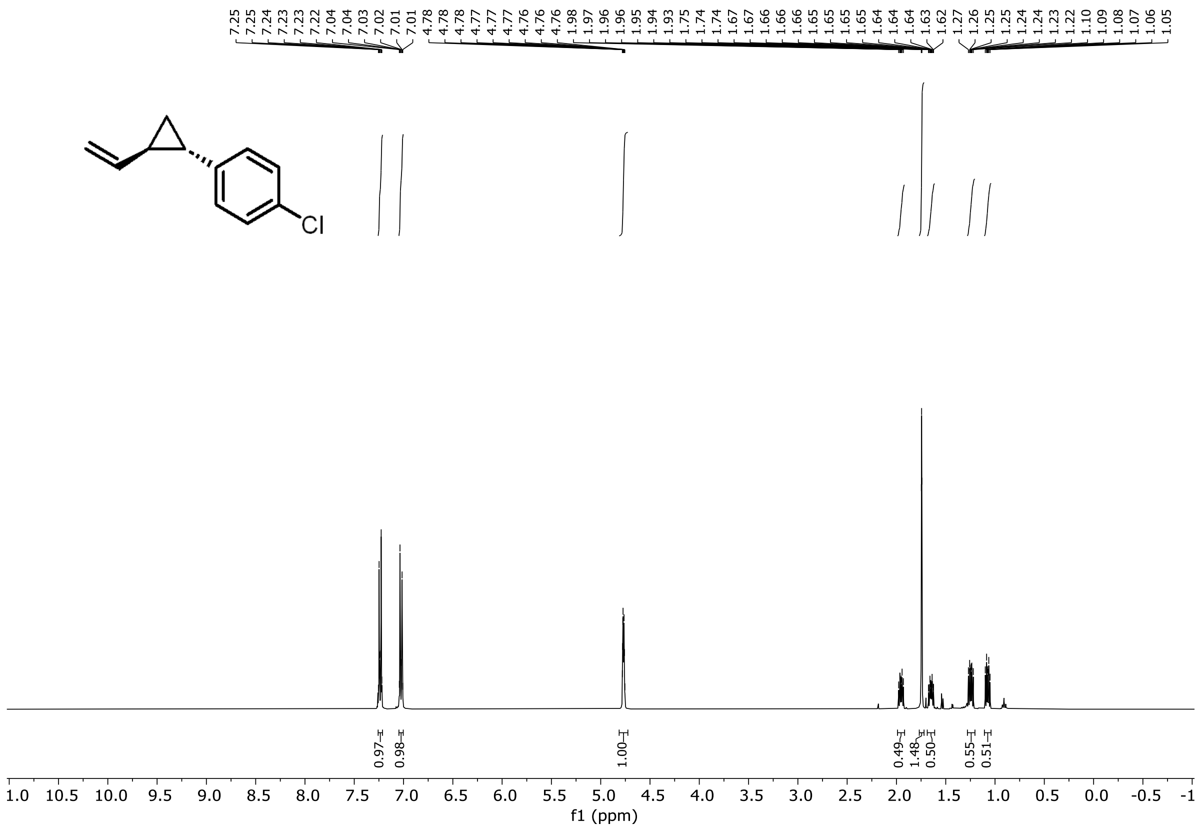

^{1}H NMR (500 MHz, $CDCl_3$, 298 K) spectrum of **VCP15**

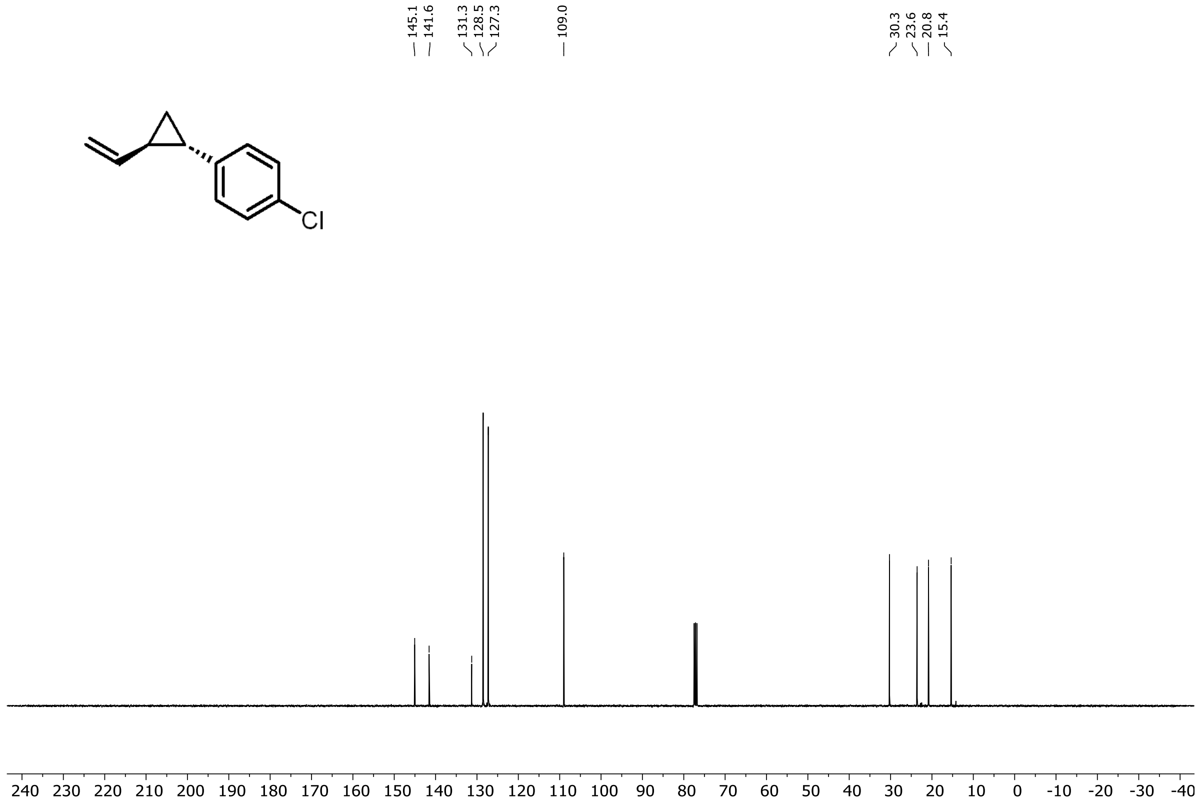

^{13}C NMR (126 MHz, $CDCl_3$, 298 K) spectrum of **VCP15**

Methyl (*R,E*)-4-(1-(*tert*-butoxy)-1-oxo-6-(phenylsulfonyl)hex-4-en-2-yl)benzoate (1)

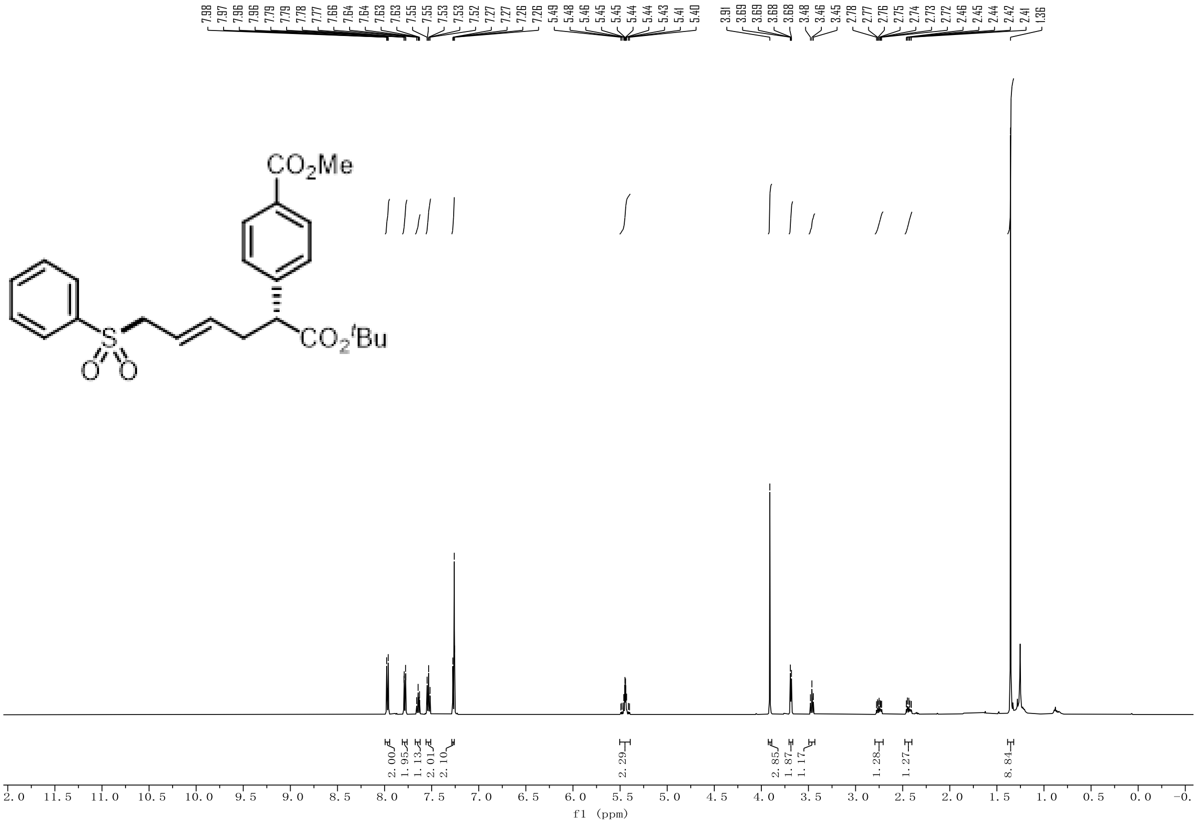

^{1}H NMR (500 MHz, $CDCl_3$, 298 K) spectrum of **1**

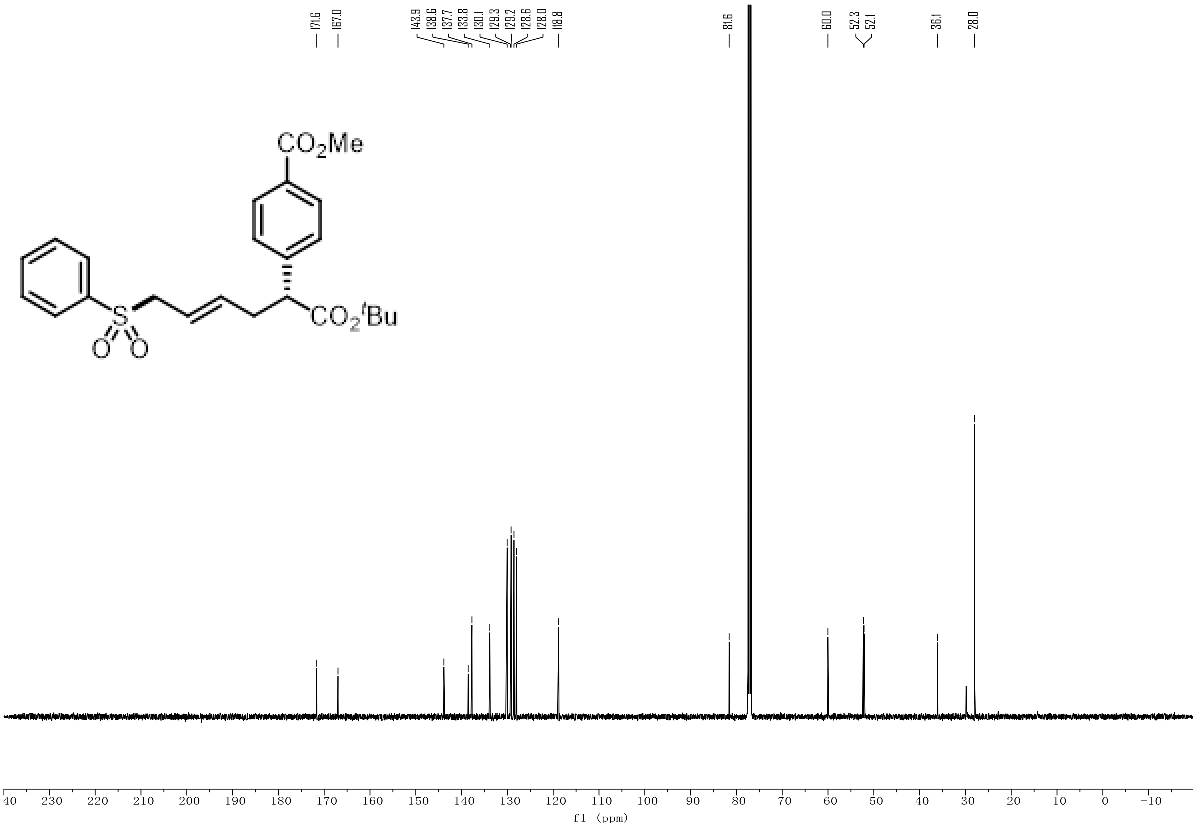

^{13}C NMR (126 MHz, $CDCl_3$, 298 K) spectrum of **1**

Methyl (*R,E*)-4-(1-(*tert*-butoxy)-7,7-dimethyl-1-oxooct-4-en-2-yl)benzoate (2)

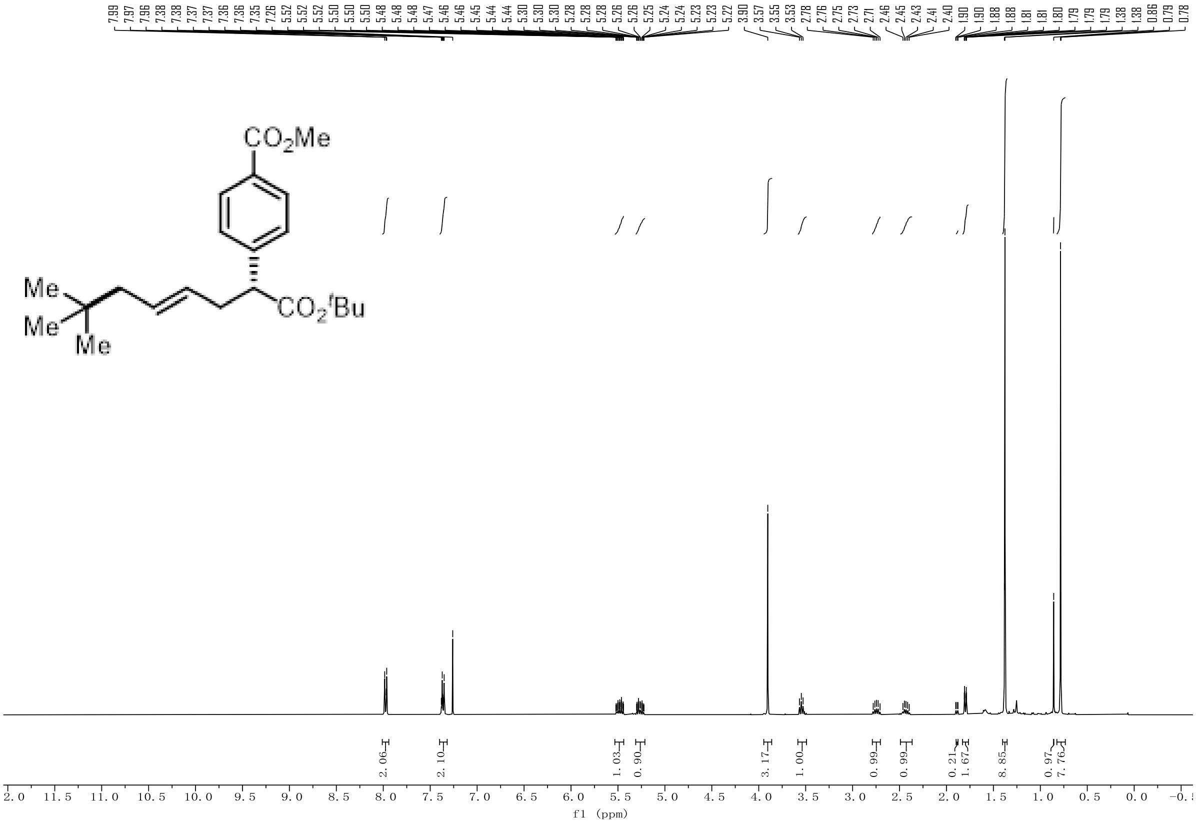

^{1}H NMR (400 MHz, $CDCl_3$, 298 K) spectrum of **2**

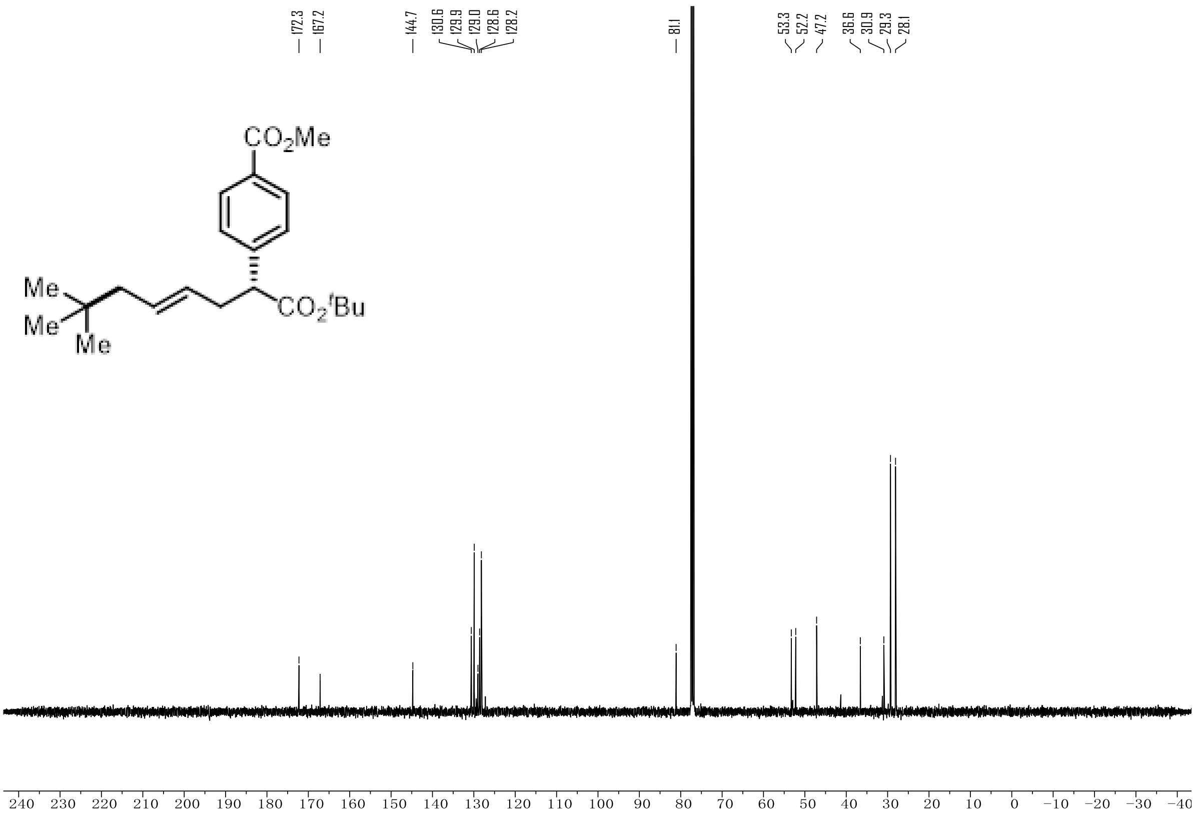

^{13}C NMR (101 MHz, $CDCl_3$, 298 K) spectrum of **2**

***tert*-Butyl (*R,E*)-2-(4-cyanophenyl)-6-(phenylsulfonyl)hex-4-enoate (3)**

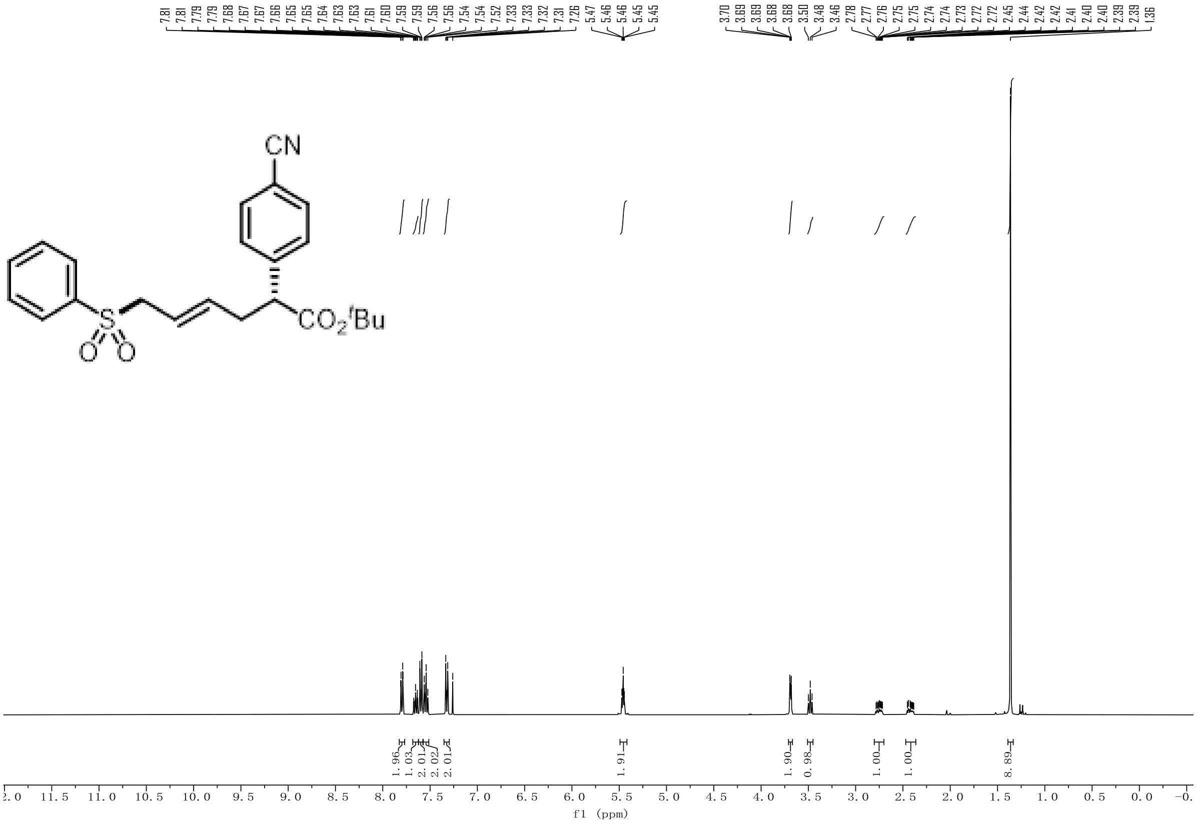

^{1}H NMR (400 MHz, $CDCl_3$, 298 K) spectrum of **3**

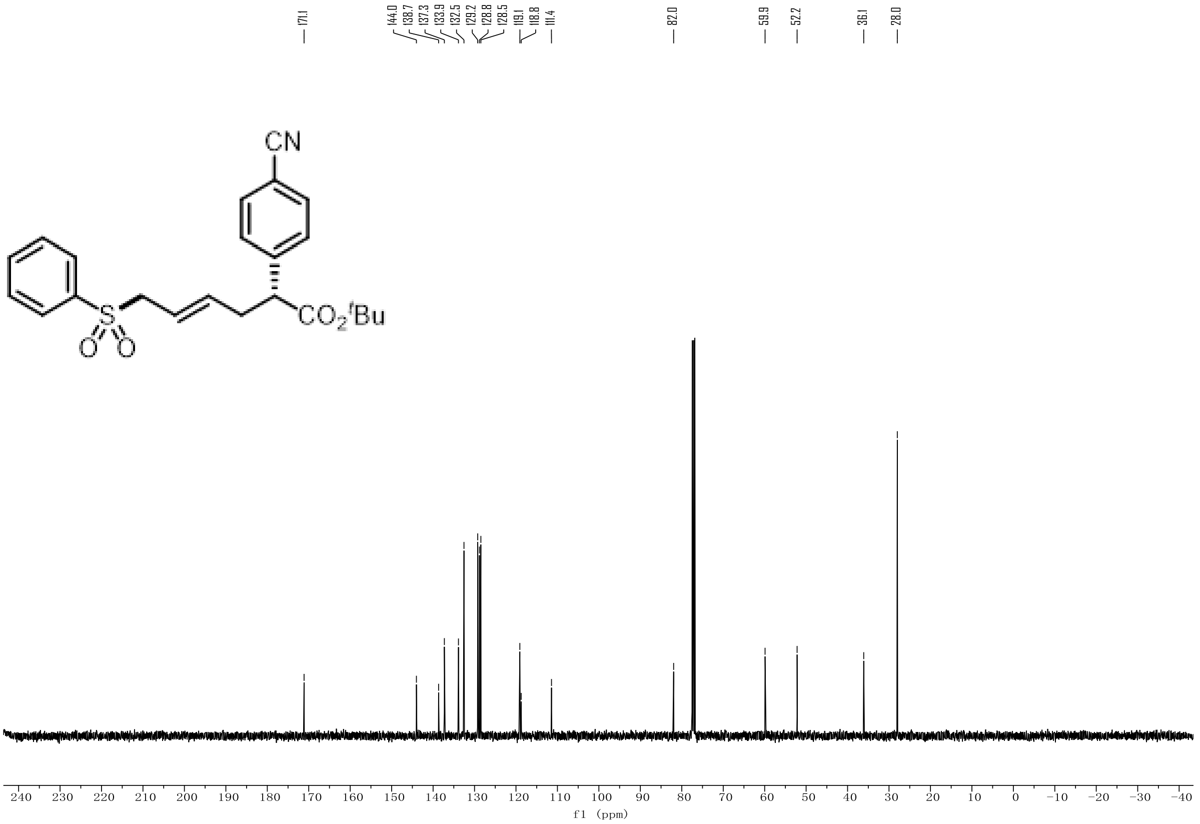

^{13}C NMR (101 MHz, $CDCl_3$, 298 K) spectrum of **3**

***tert*-Butyl (*R*,*E*)-2-(4-formylphenyl)-6-(phenylsulfonyl)hex-4-enoate (4)**

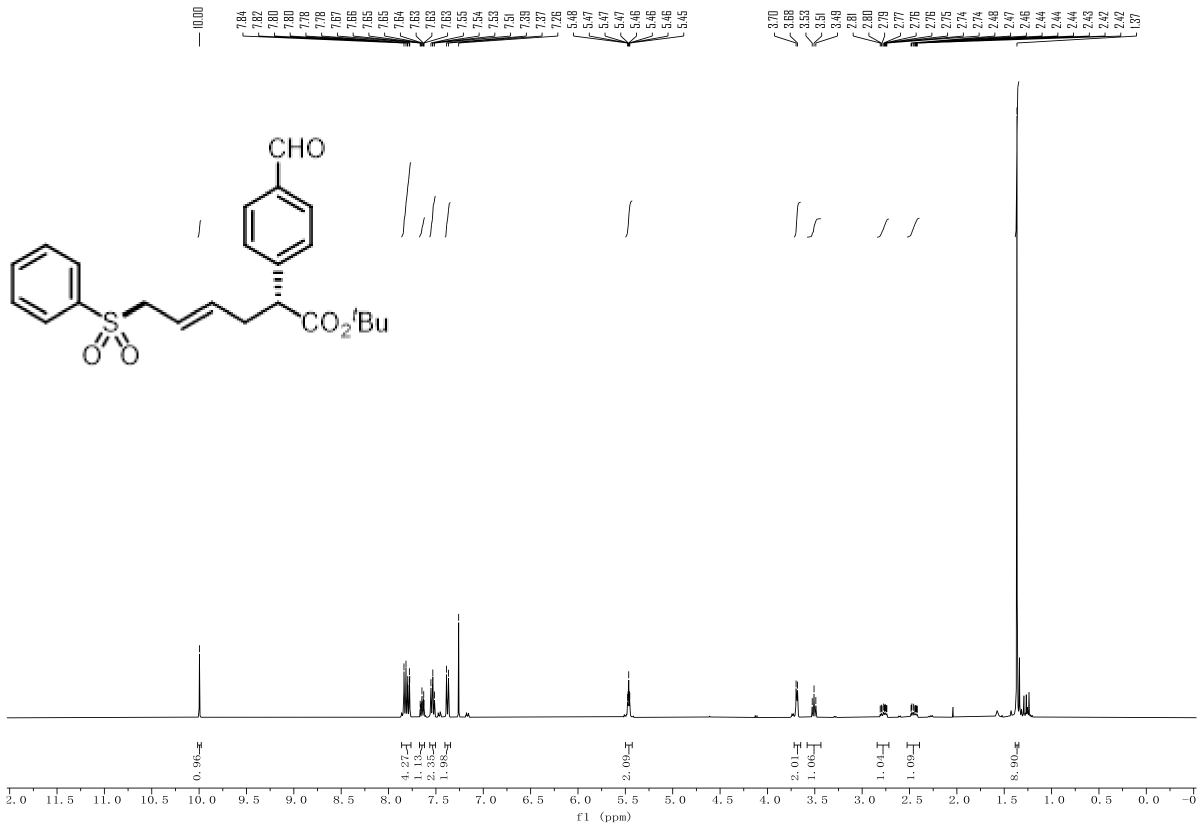

^1H NMR (400 MHz, $CDCl_3$, 298 K) spectrum of **4**

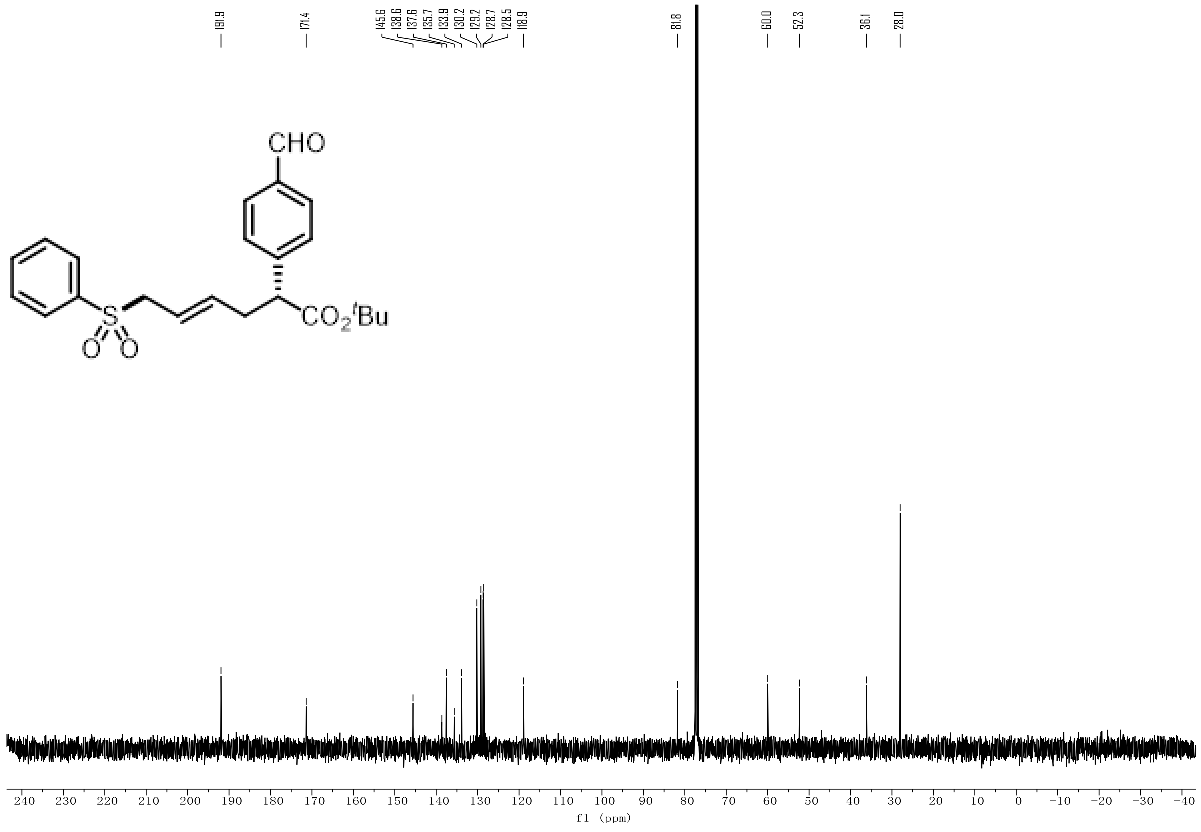

^{13}C NMR (101 MHz, $CDCl_3$, 298 K) spectrum of **4**

tert-Butyl (*R,E*)-2-(4-acetylphenyl)-6-(phenylsulfonyl)hex-4-enoate (5)

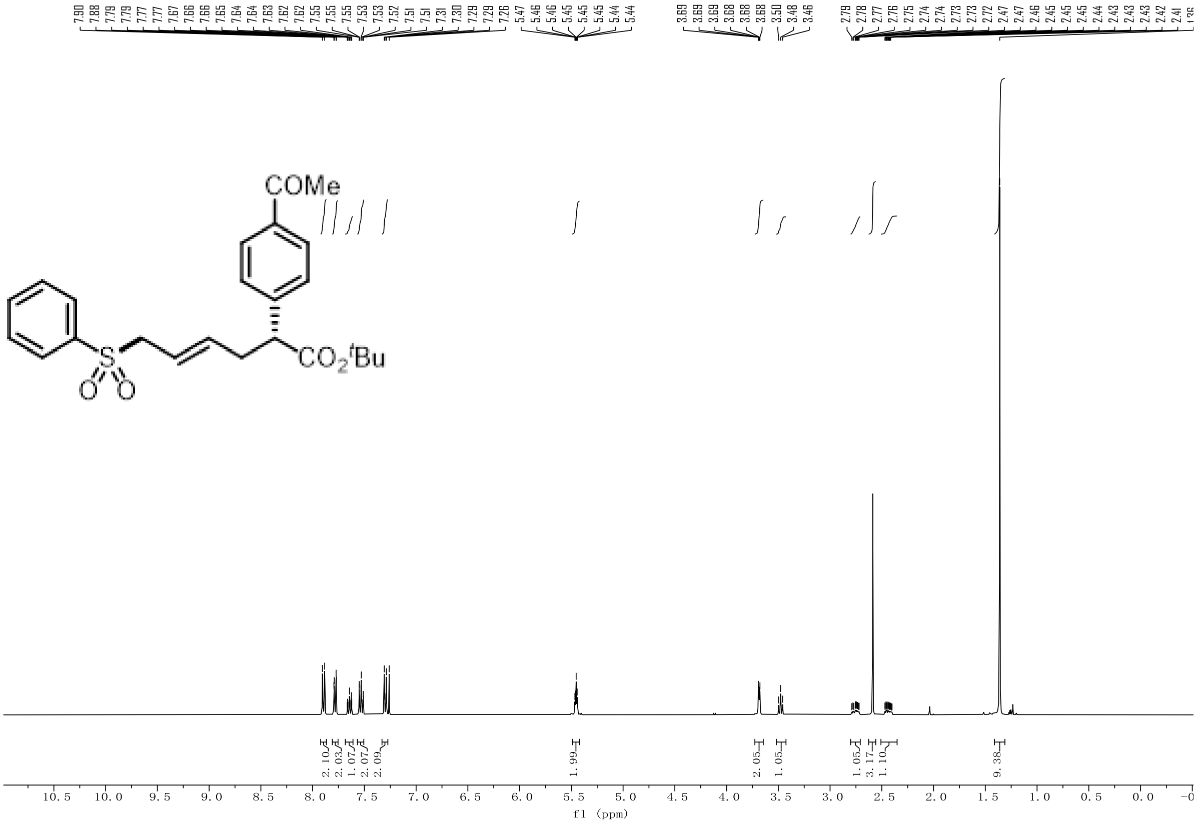

^{1}H NMR (400 MHz, $CDCl_3$, 298 K) spectrum of **5**

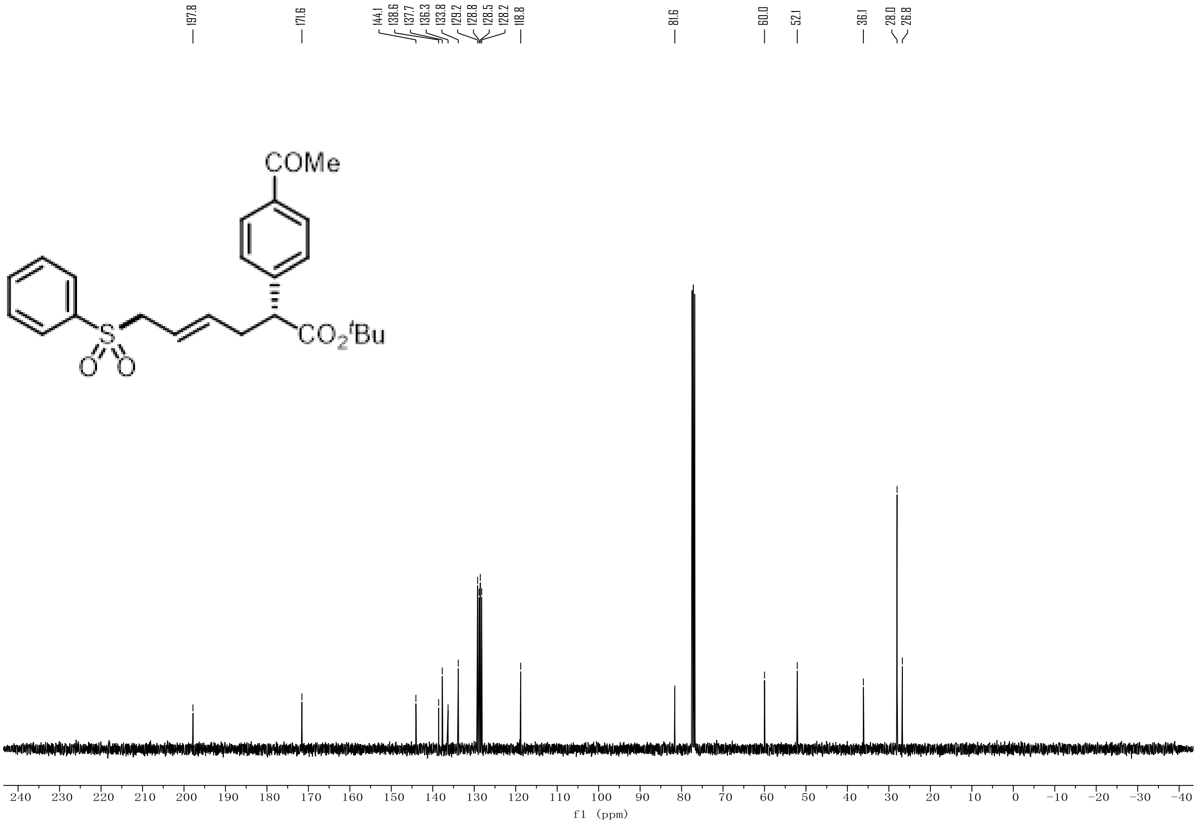

^{13}C NMR (101 MHz, $CDCl_3$, 298 K) spectrum of **5**

tert-Butyl (_R,E_)-2-(4-benzoylphenyl)-6-(phenylsulfonyl)hex-4-enoate (6)

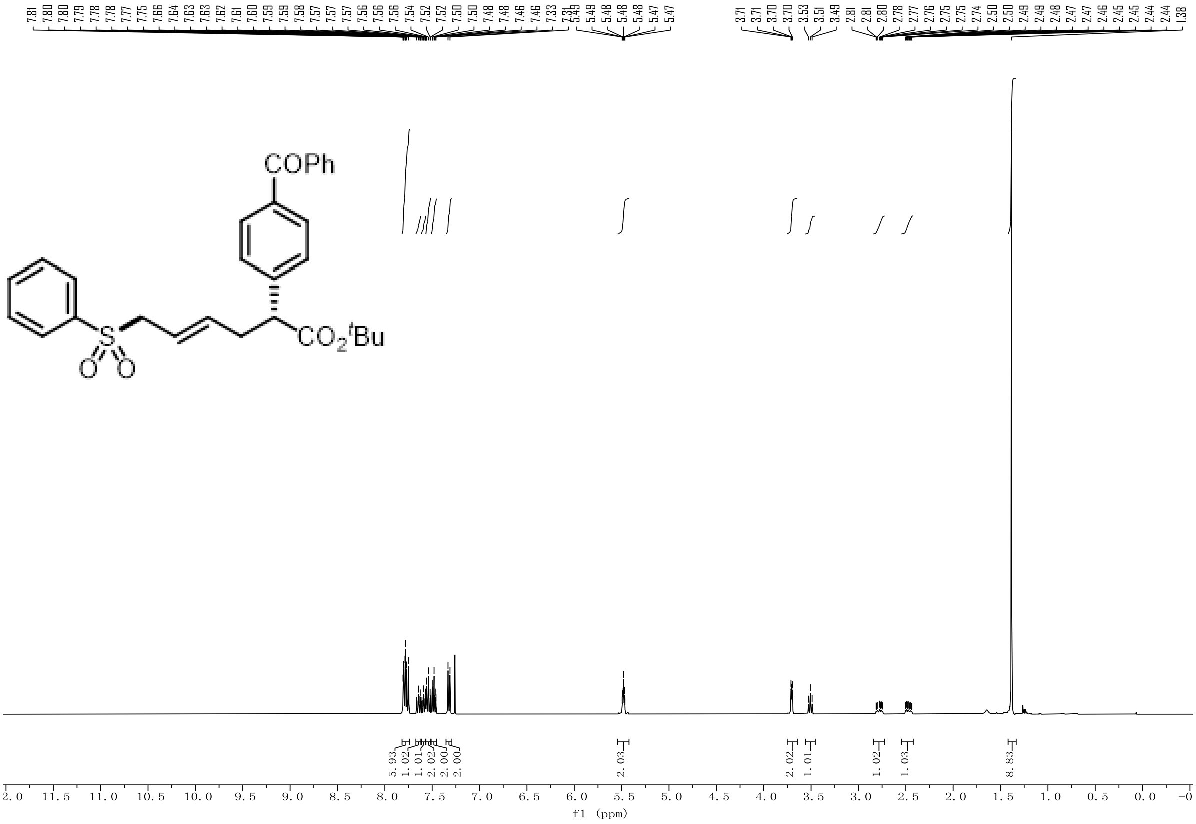

^{1}H NMR (400 MHz, $CDCl_3$, 298 K) spectrum of **6**

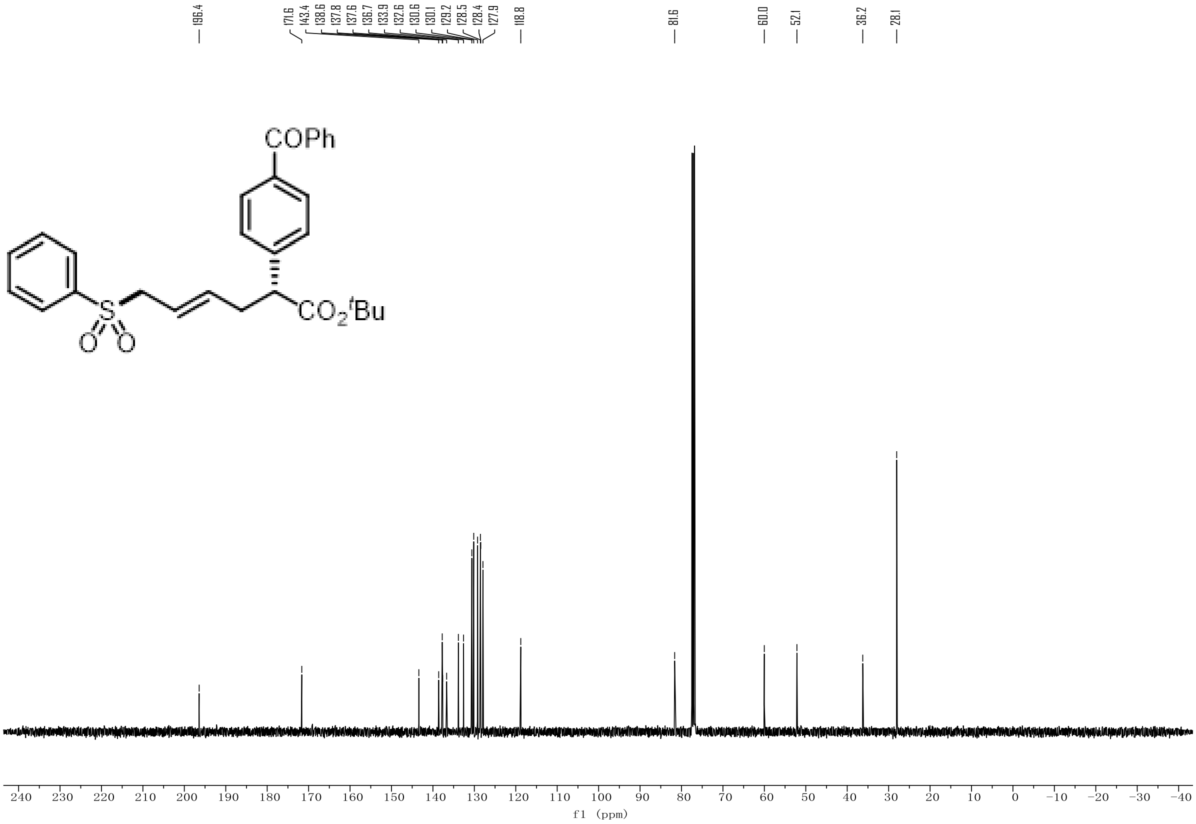

^{13}C NMR (101 MHz, $CDCl_3$, 298 K) spectrum of **6**

tert-Butyl (_R,E_)-2-(3-cyanophenyl)-6-(phenylsulfonyl)hex-4-enoate (7)

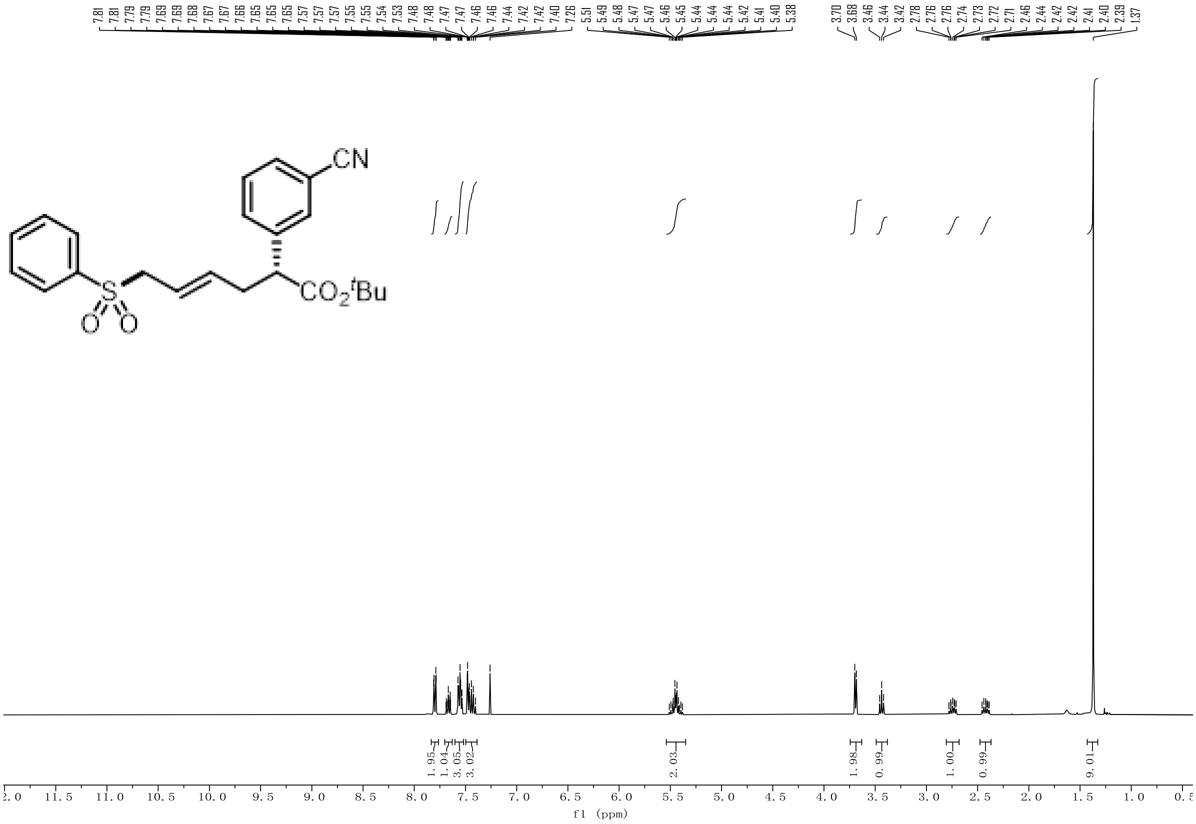

^{1}H NMR (400 MHz, CDCl$_3$, 298 K) spectrum of **7**

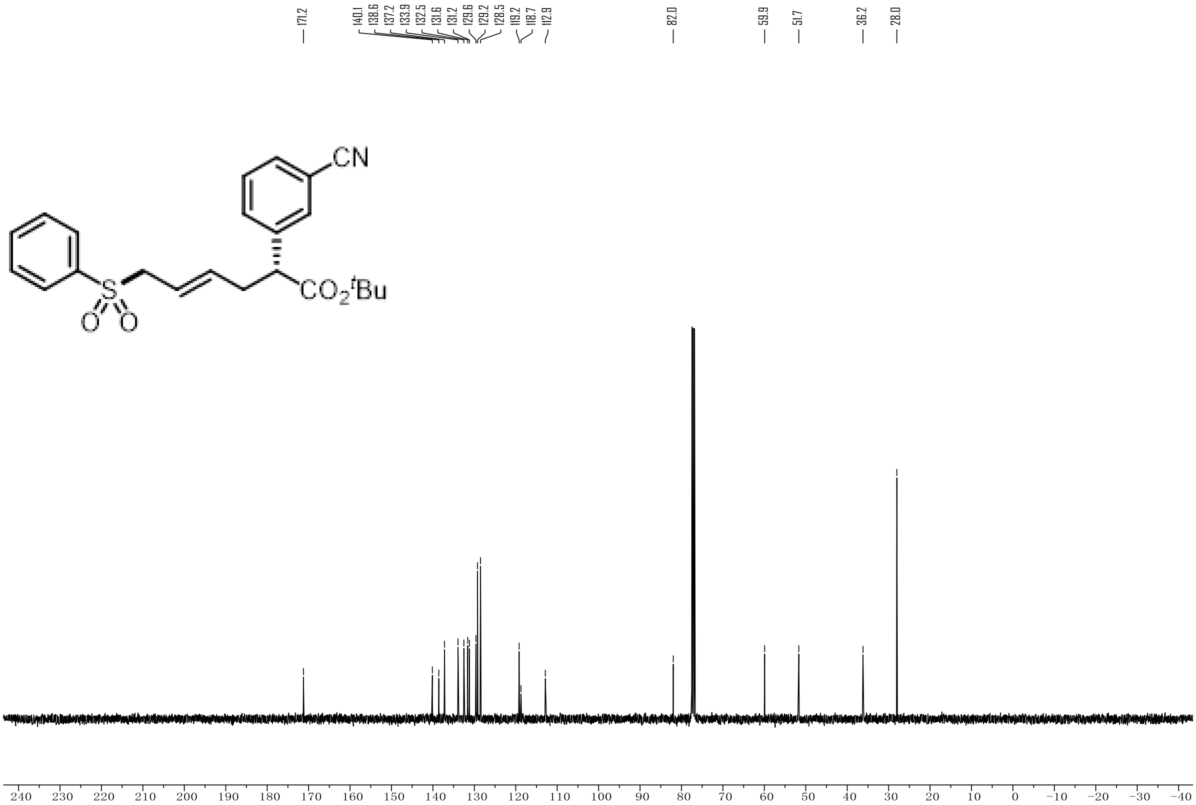

^{13}C NMR (101 MHz, CDCl$_3$, 298 K) spectrum of **7**

Methyl (*R,E*)-3-(1-(*tert*-butoxy)-1-oxo-6-(phenylsulfonyl)hex-4-en-2-yl)benzoate (8)

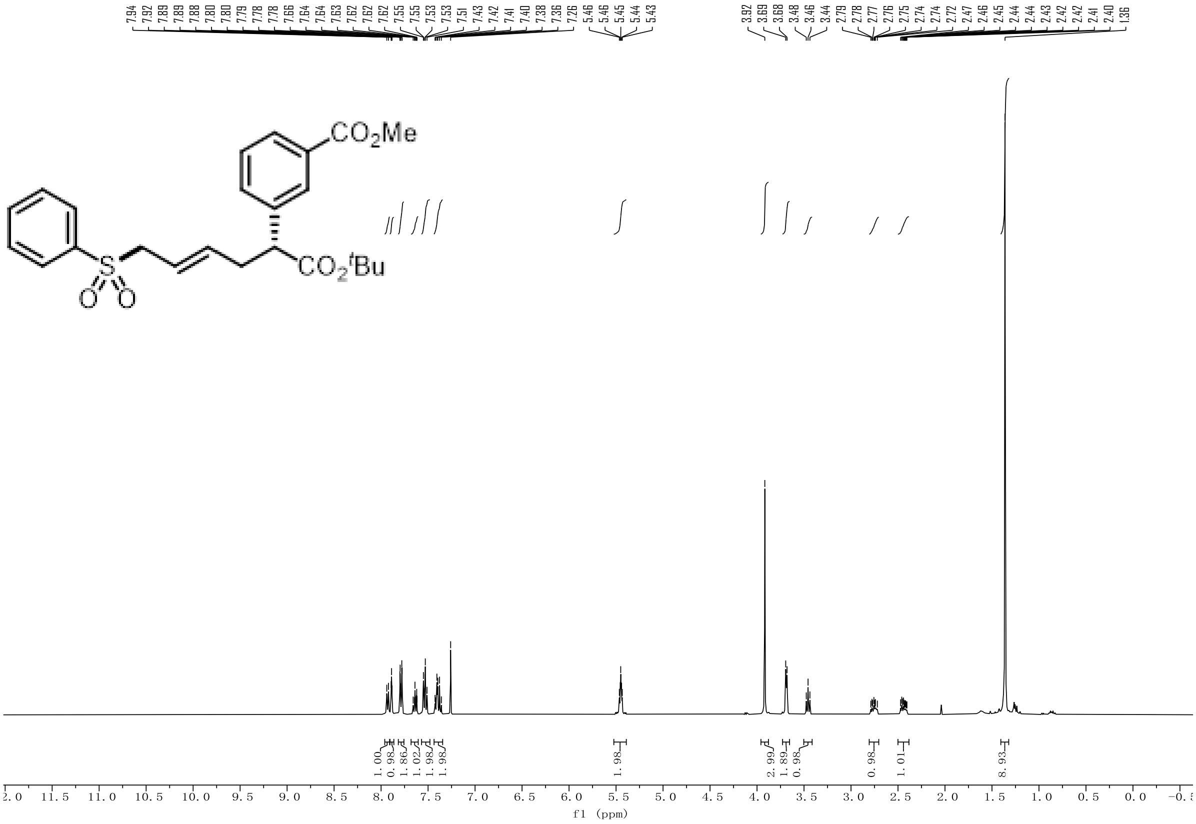

^{1}H NMR (400 MHz, $CDCl_3$, 298 K) spectrum of **8**

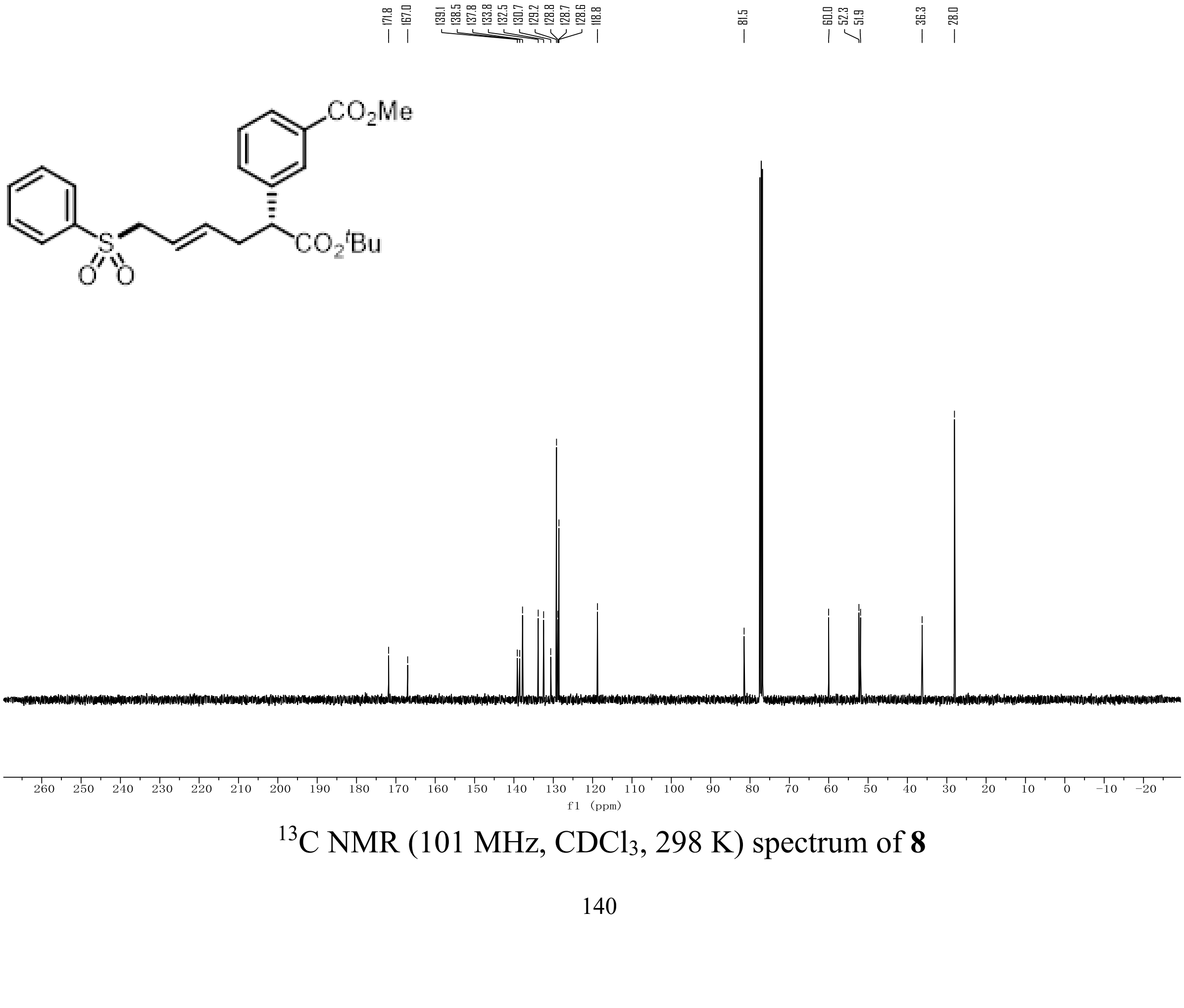

^{13}C NMR (101 MHz, $CDCl_3$, 298 K) spectrum of **8**

Ethyl (*S*)-4-(1-benzamido-2-(phenylsulfonyl)ethyl)benzoate (9)

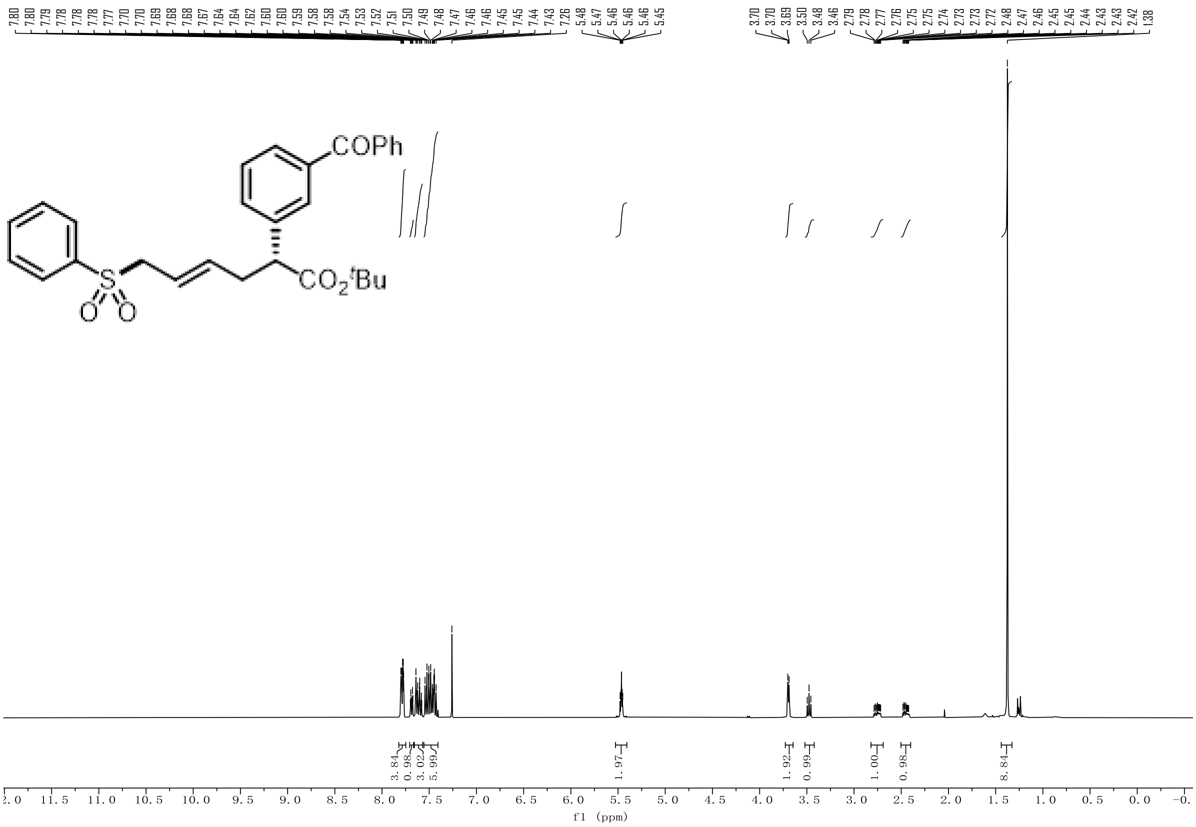

^{1}H NMR (400 MHz, $CDCl_3$, 298 K) spectrum of **9**

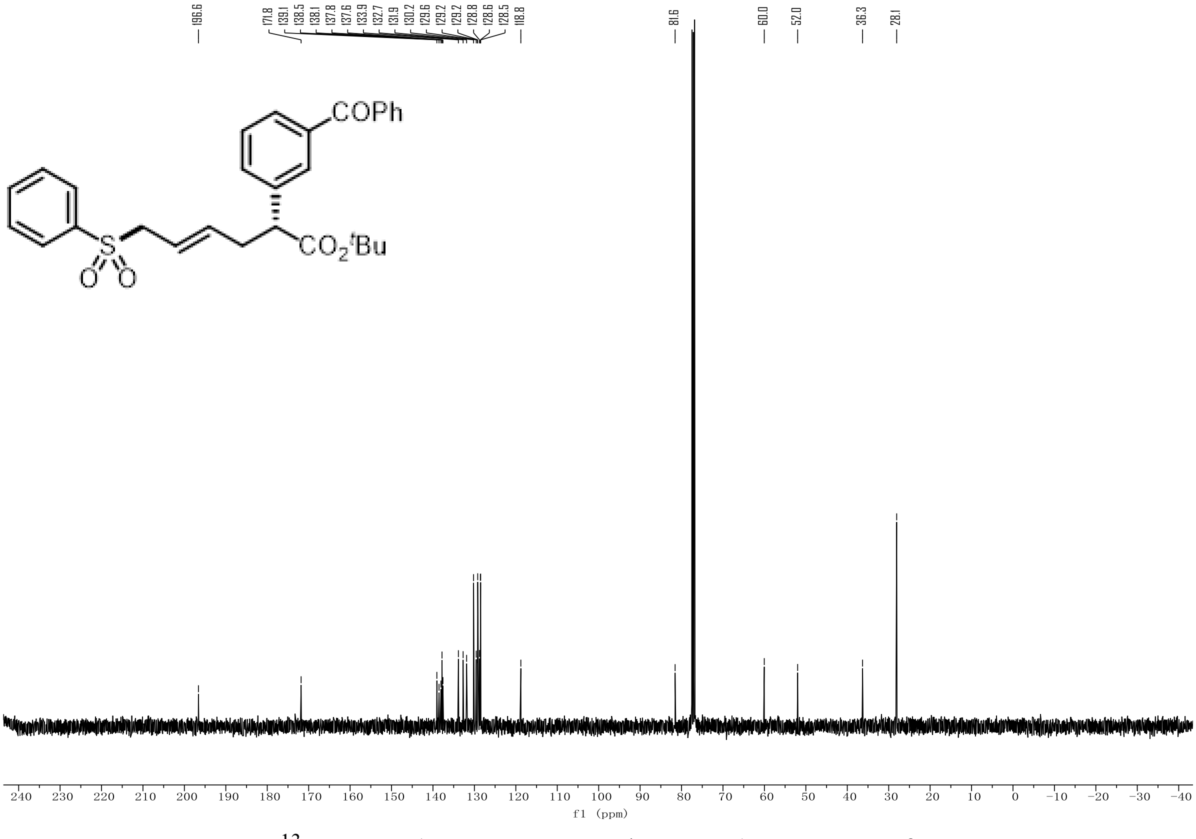

^{13}C NMR (101 MHz, $CDCl_3$, 298 K) spectrum of **9**

***tert*-Butyl (*R,E*)-2-(3-chlorophenyl)-6-(phenylsulfonyl)hex-4-enoate (10)**

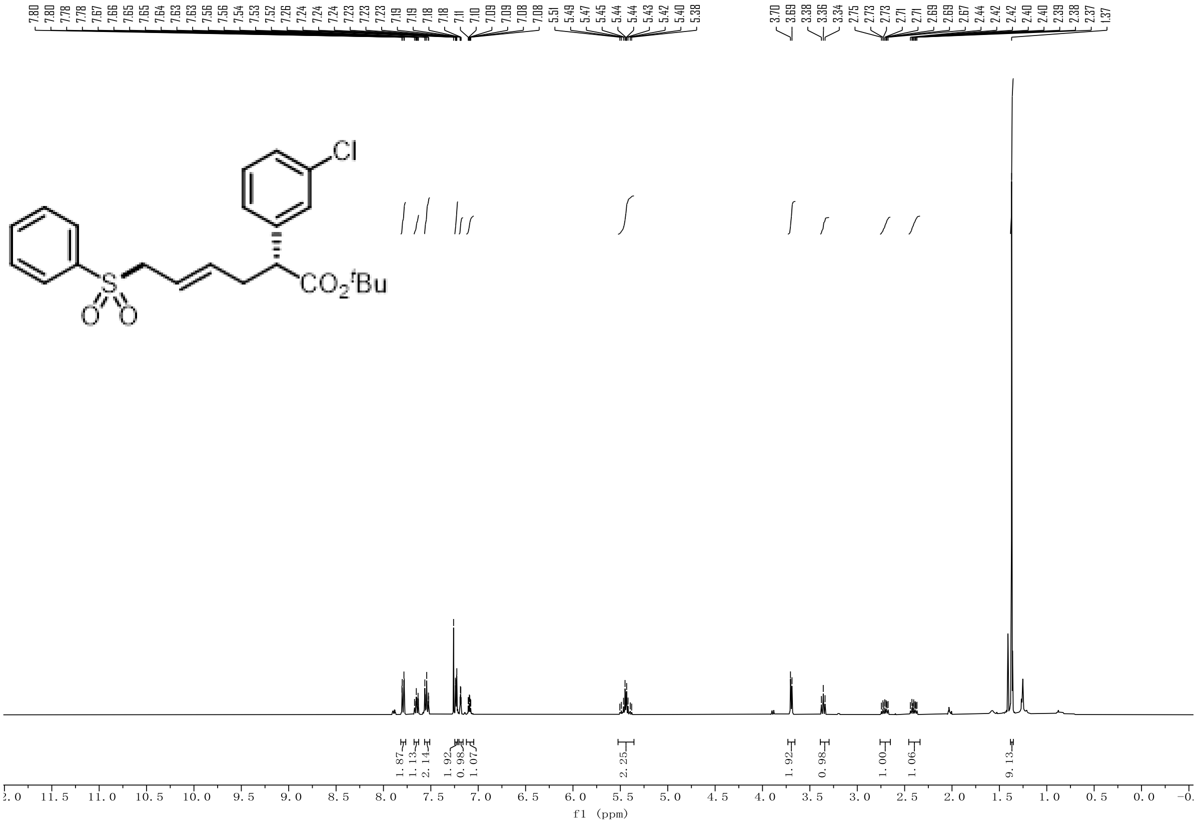

^{1}H NMR (400 MHz, $CDCl_3$, 298 K) spectrum of **10**

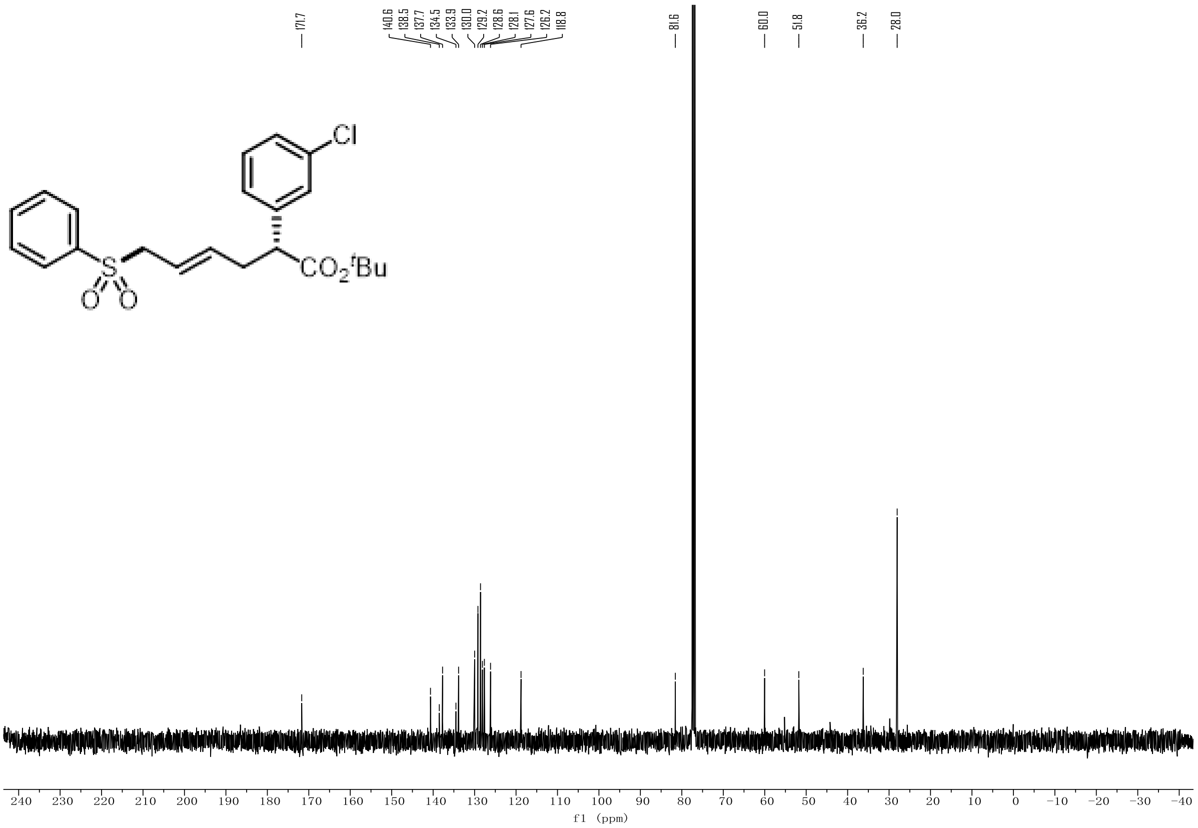

^{13}C NMR (101 MHz, $CDCl_3$, 298 K) spectrum of **10**

Methyl (*R,E*)-3-(1-(*tert*-butoxy)-1-oxo-6-(phenylsulfonyl)hex-4-en-2-yl)-5-chlorobenzoate (11)

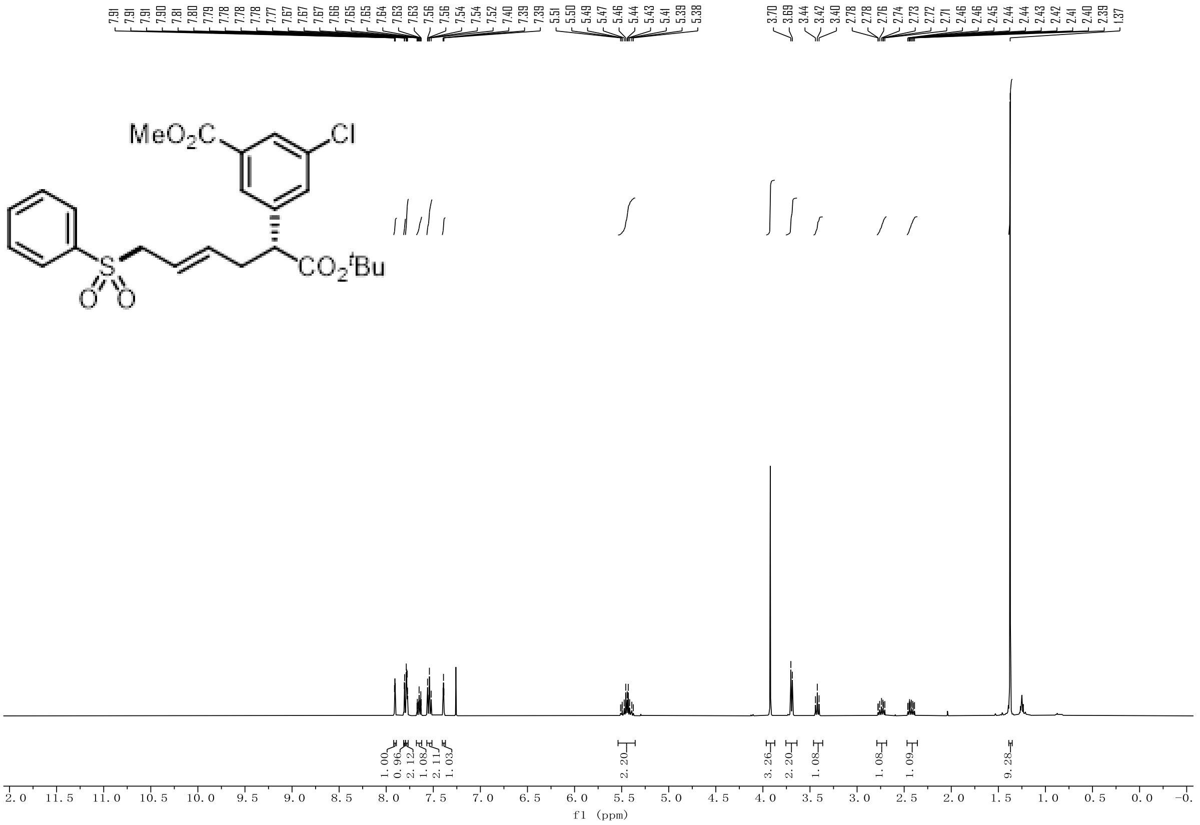

^{1}H NMR (400 MHz, $CDCl_3$, 298 K) spectrum of **11**

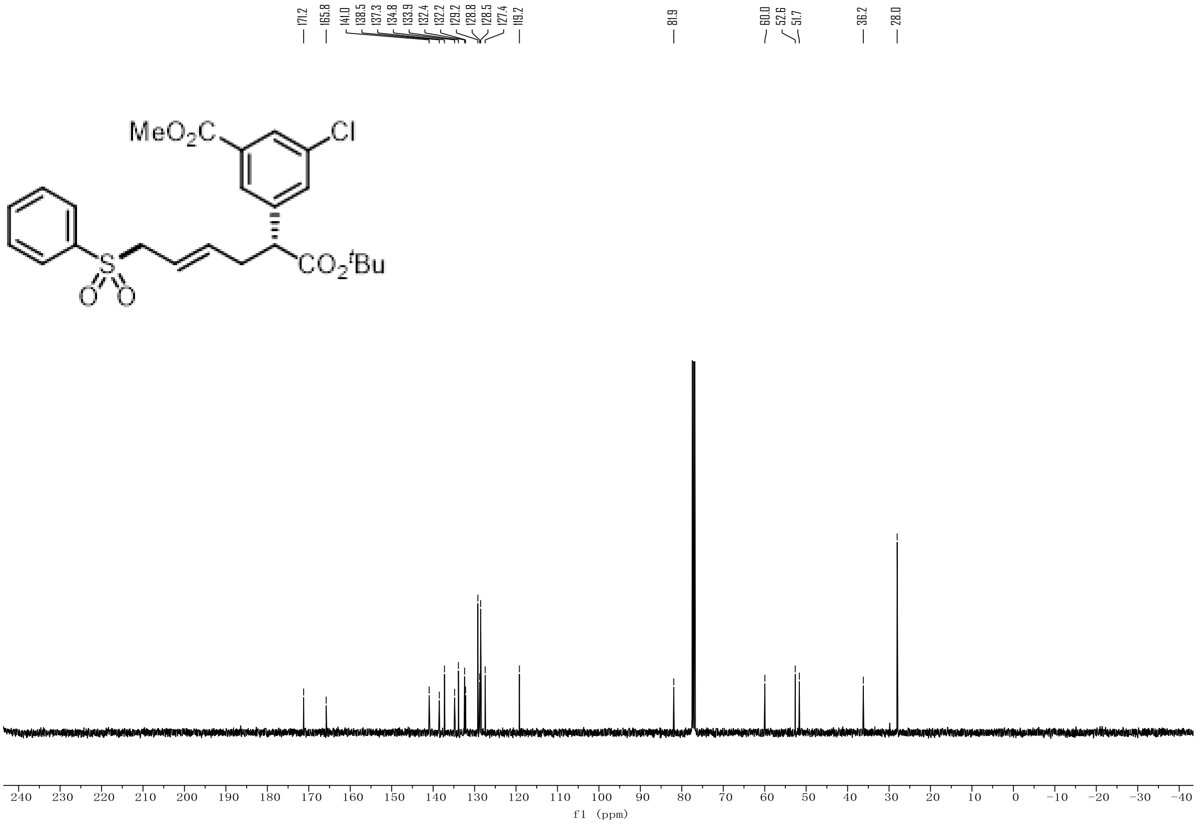

^{13}C NMR (101 MHz, $CDCl_3$, 298 K) spectrum of **11**

tert-Butyl (_R,E_)-2-(naphthalen-2-yl)-6-(phenylsulfonyl)hex-4-enoate (12)

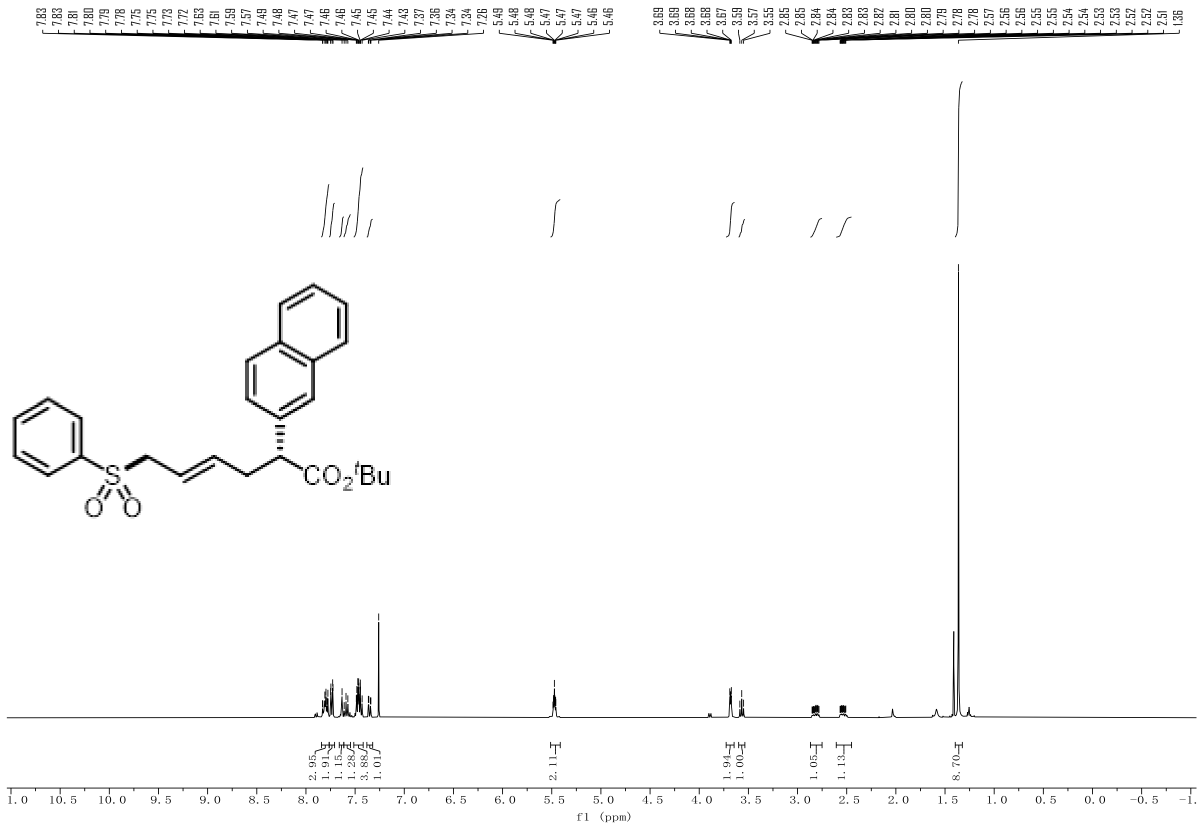

^{1}H NMR (400 MHz, $CDCl_3$, 298 K) spectrum of **12**

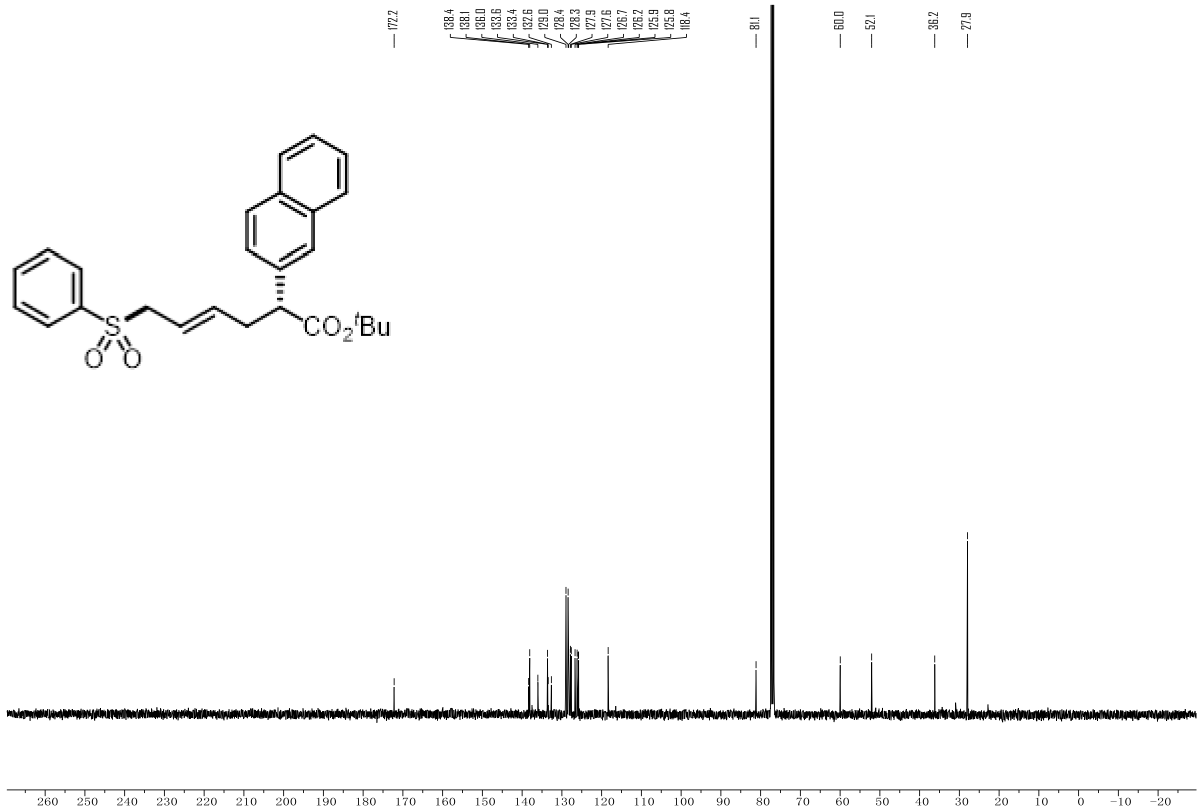

^{13}C NMR (101 MHz, $CDCl_3$, 298 K) spectrum of **12**

***tert*-Butyl (*R*,*E*)-2-(1-oxo-1,3-dihydroisobenzofuran-5-yl)-6-(phenylsulfonyl)hex-4-enoate (13)**

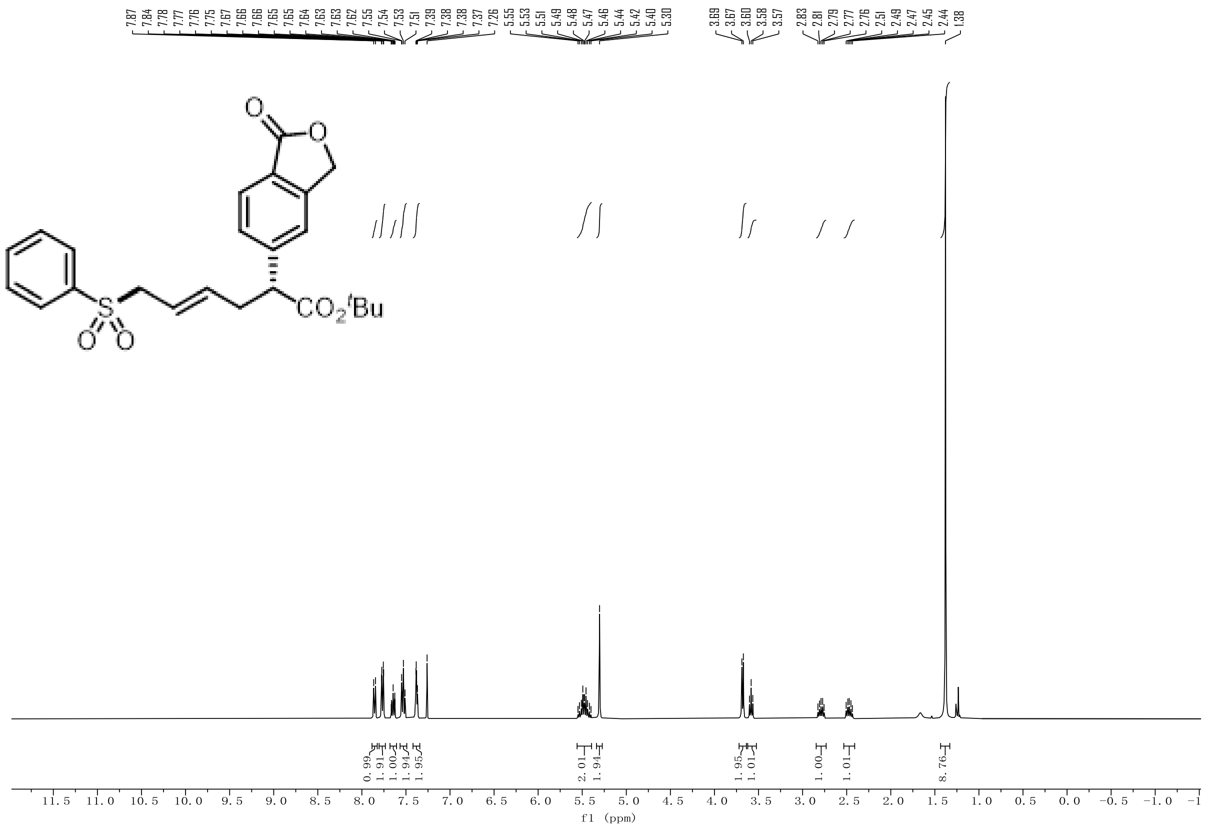

^{1}H NMR (400 MHz, $CDCl_3$, 298 K) spectrum of **13**

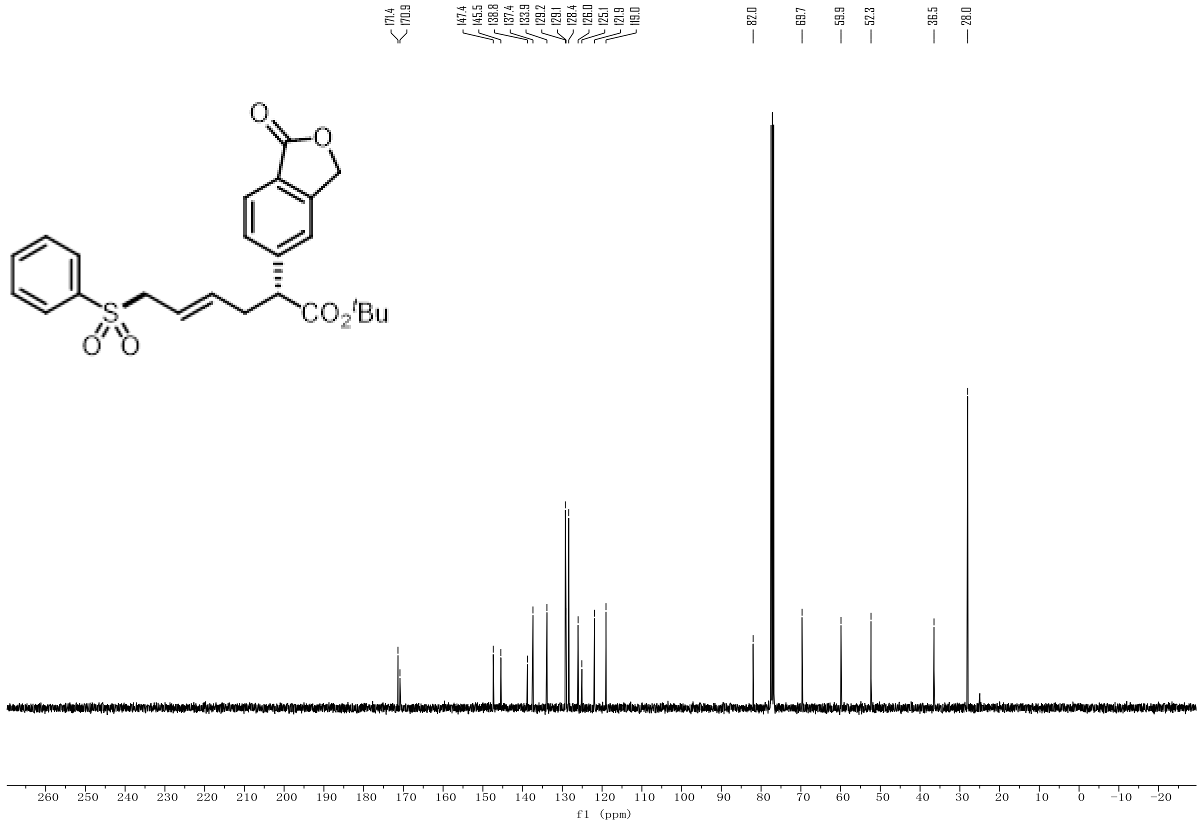

^{13}C NMR (101 MHz, $CDCl_3$, 298 K) spectrum of **13**

tert-Butyl (_R,E_)-6-(phenylsulfonyl)-2-(4-(pyridin-2-yl)phenyl)hex-4-enoate (14)

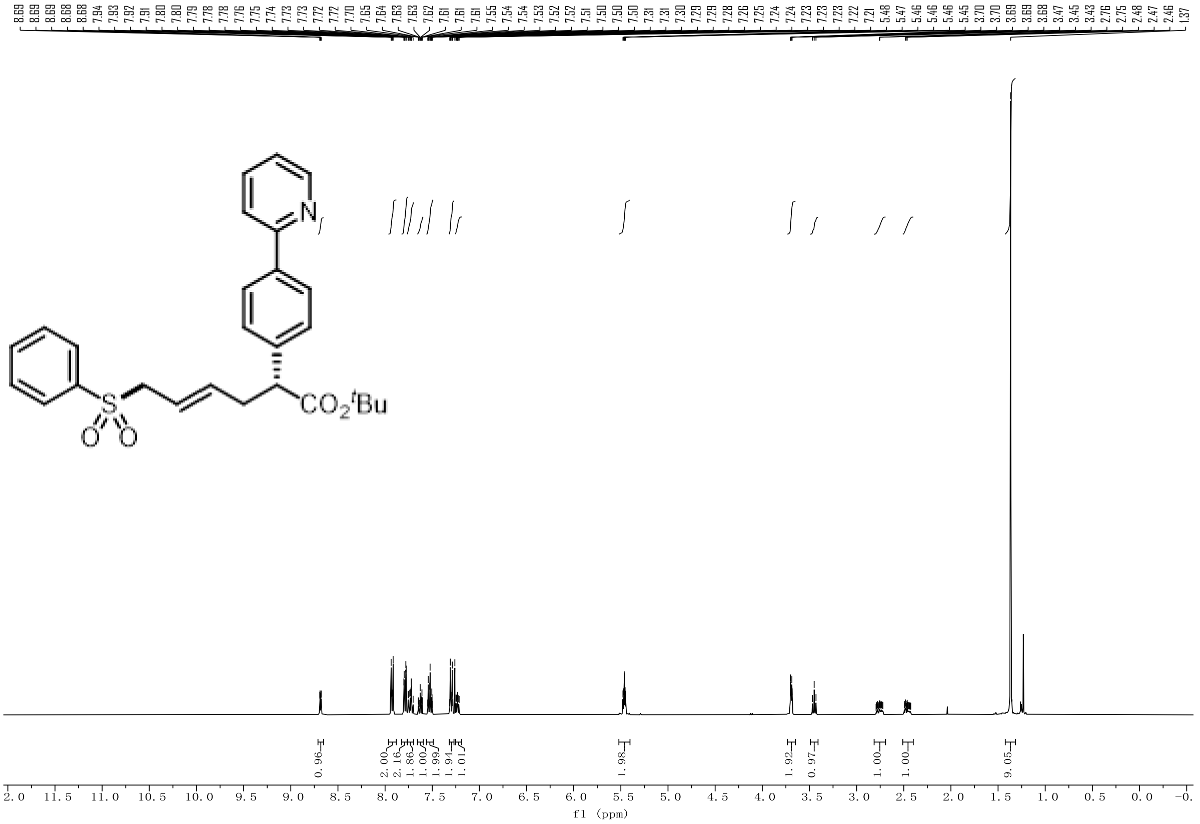

^{1}H NMR (400 MHz, $CDCl_3$, 298 K) spectrum of **14**

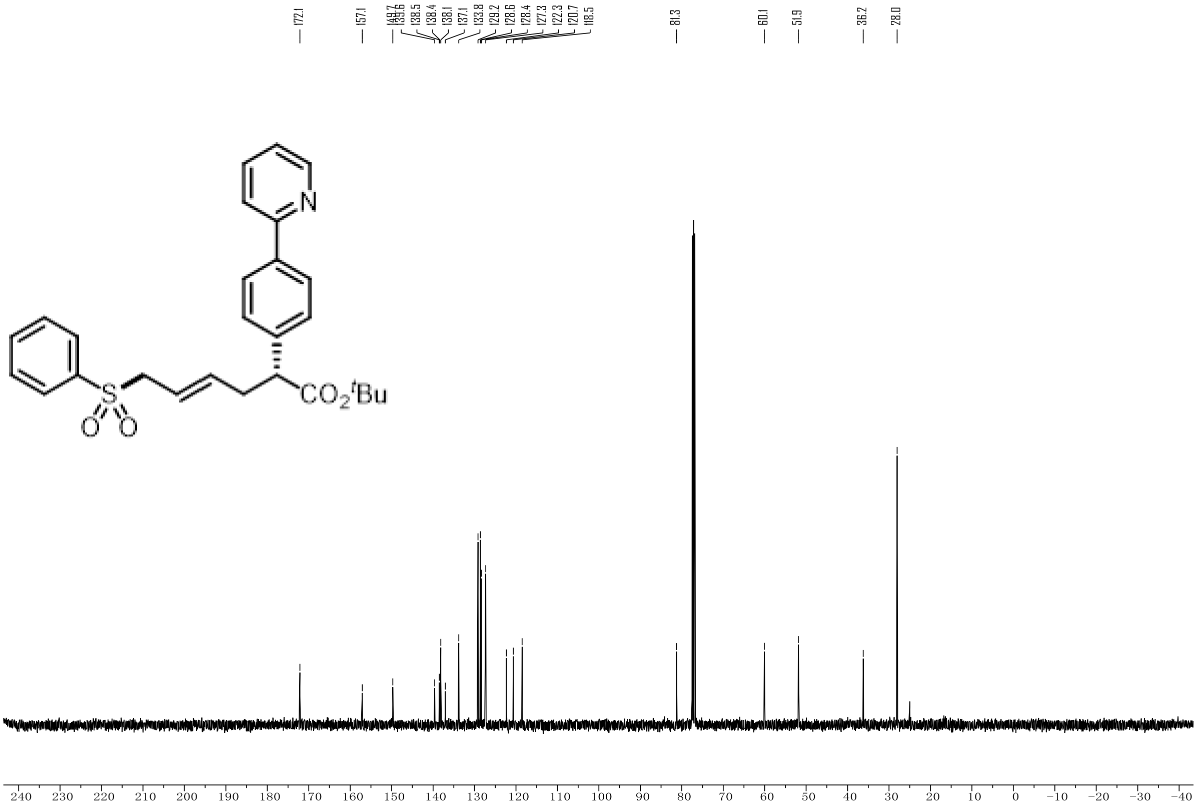

^{13}C NMR (101 MHz, $CDCl_3$, 298 K) spectrum of **14**

***tert*-Butyl (*R,E*)-6-(phenylsulfonyl)-2-(quinolin-7-yl)hex-4-enoate (15)**

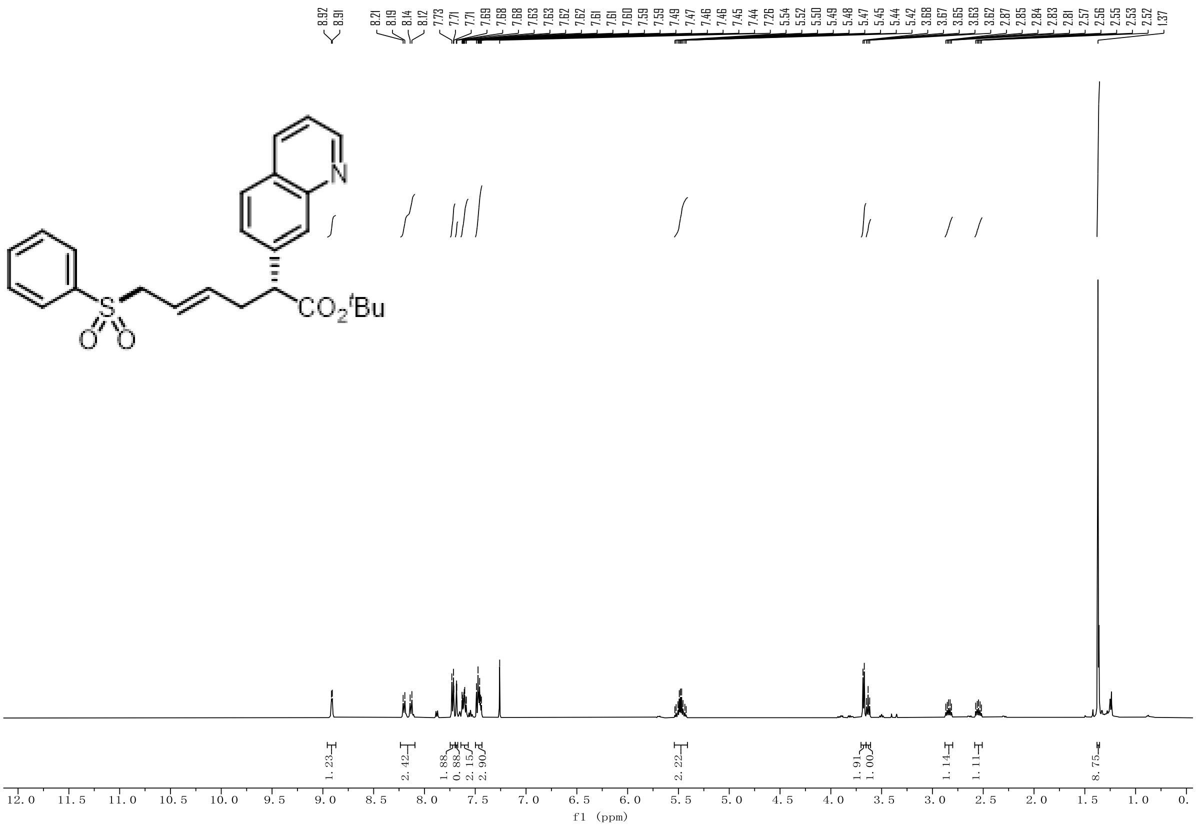

^{1}H NMR (500 MHz, $CDCl_3$, 298 K) spectrum of **15**

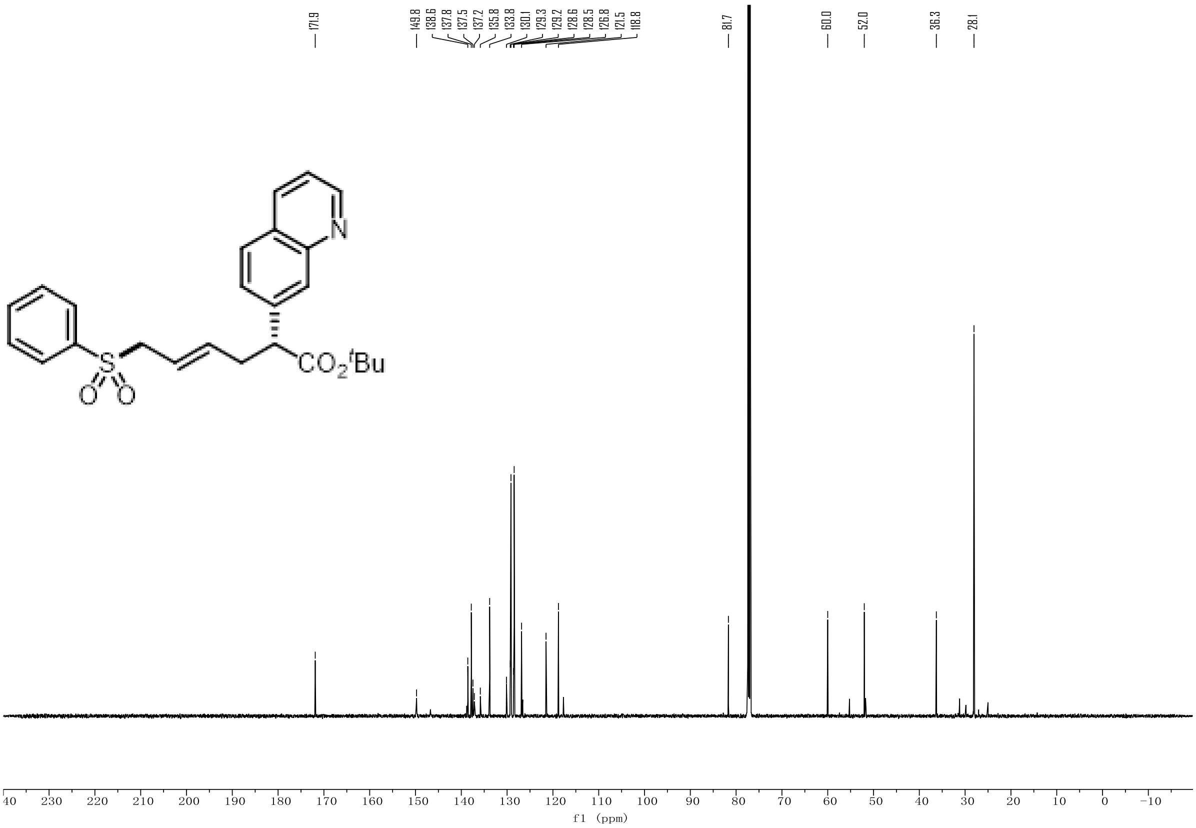

^{13}C NMR (126 MHz, $CDCl_3$, 298 K) spectrum of **15**

tert-Butyl (_R,E_)-2-(2-methyl-1,3-dioxoisoindolin-5-yl)-6-(phenylsulfonyl)hex-4-enoate (16)

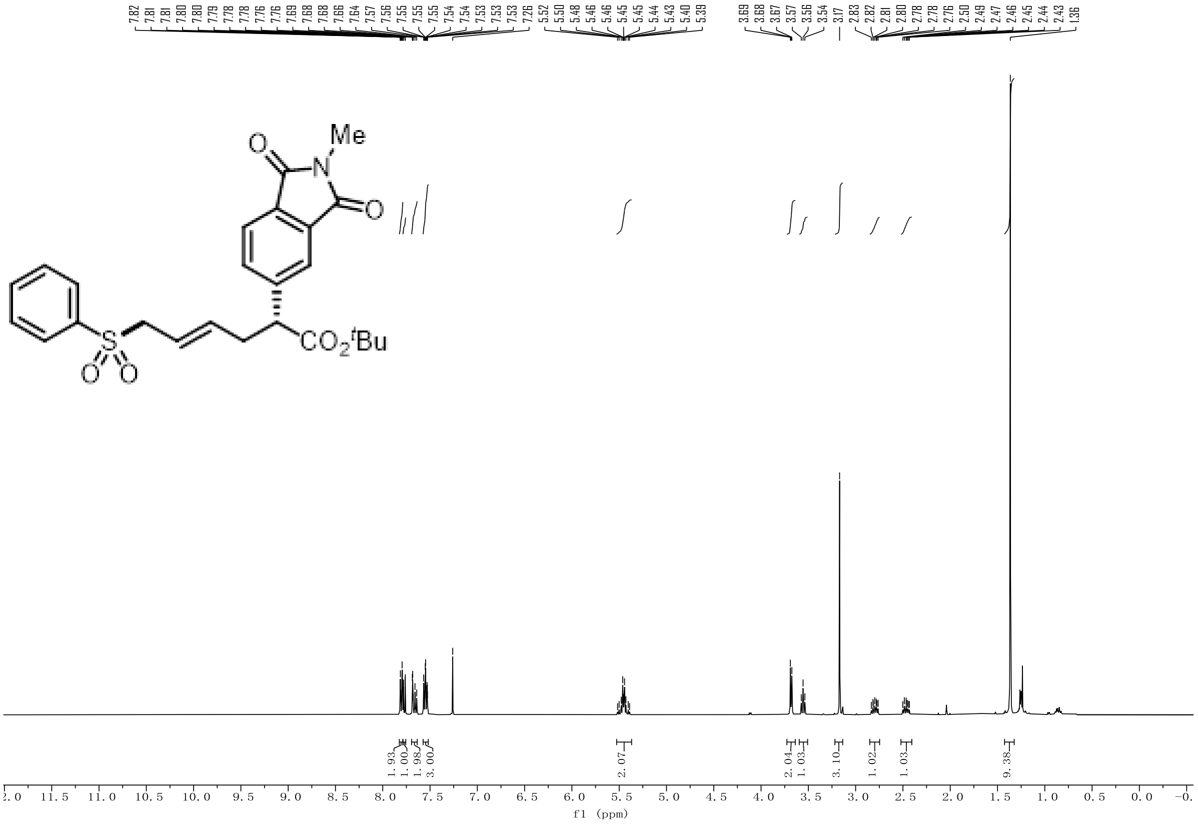

^{1}H NMR (400 MHz, $CDCl_3$, 298 K) spectrum of **16**

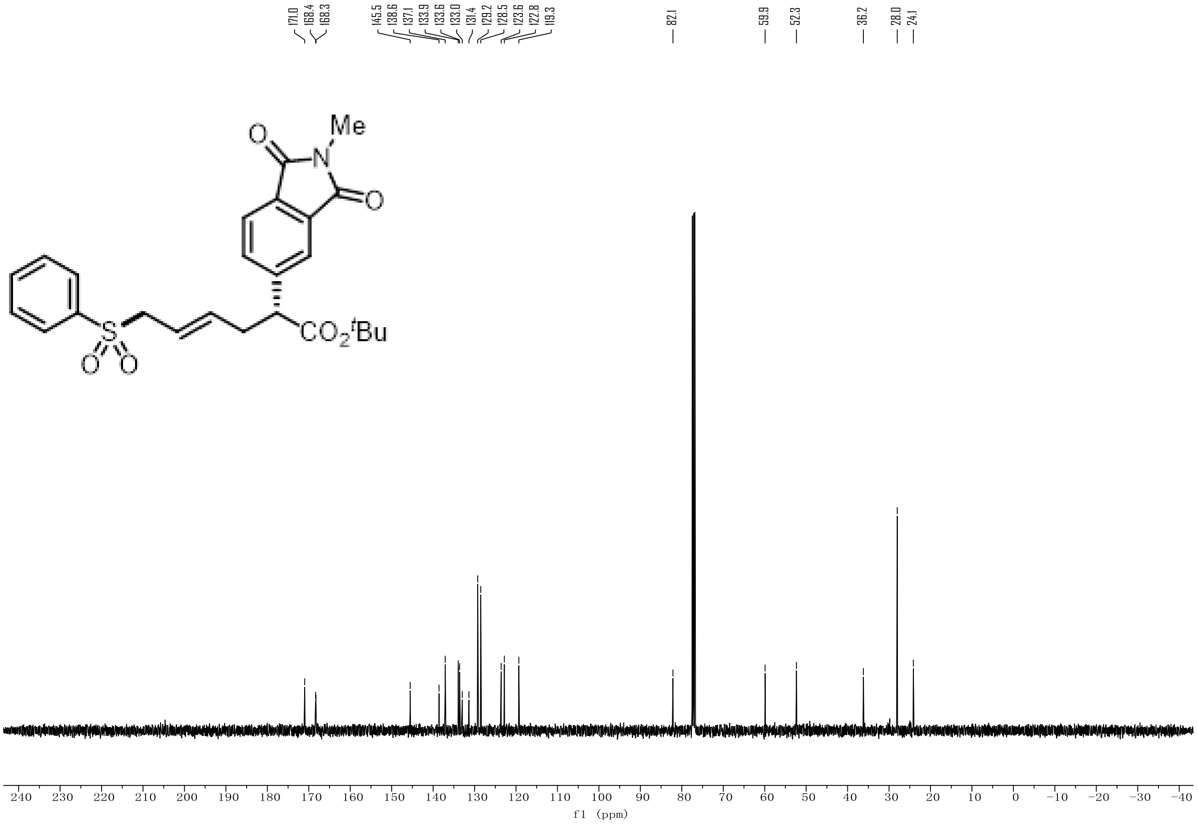

^{13}C NMR (101 MHz, $CDCl_3$, 298 K) spectrum of **16**

***tert*-Butyl (*R,E*)-2-(1H-inden-2-yl)-6-(phenylsulfonyl)hex-4-enoate (17)**

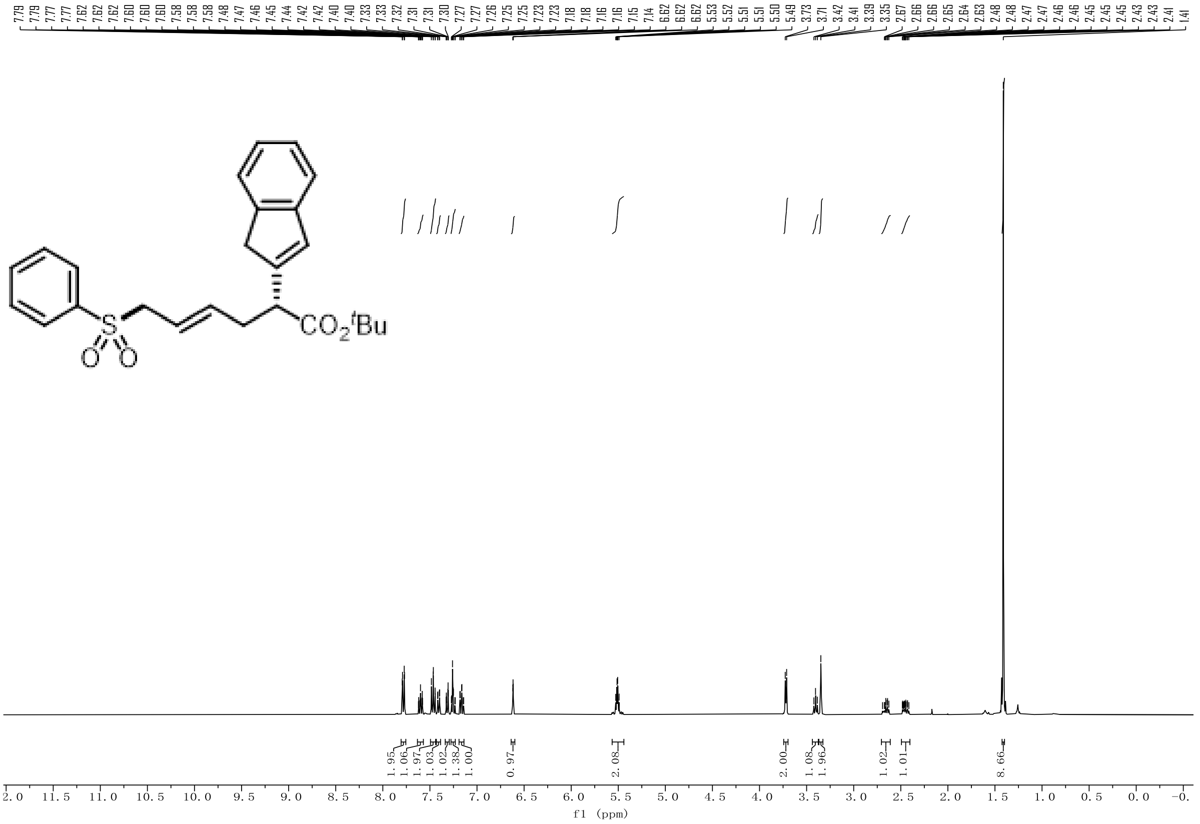

^{1}H NMR (400 MHz, $CDCl_3$, 298 K) spectrum of **17**

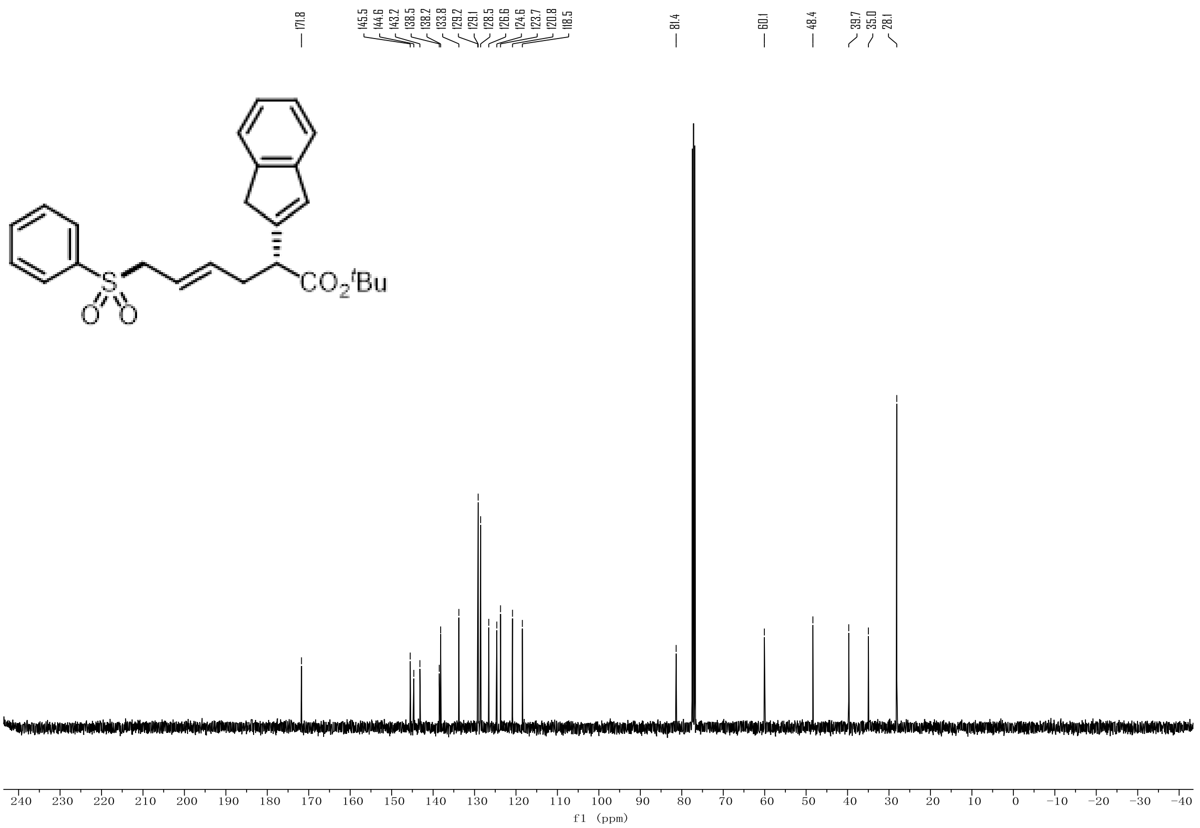

^{13}C NMR (101 MHz, $CDCl_3$, 298 K) spectrum of **17**

tert-Butyl (_R,E_)-6-(phenylsulfonyl)-2-((_E_)-styryl)hex-4-enoate (18)

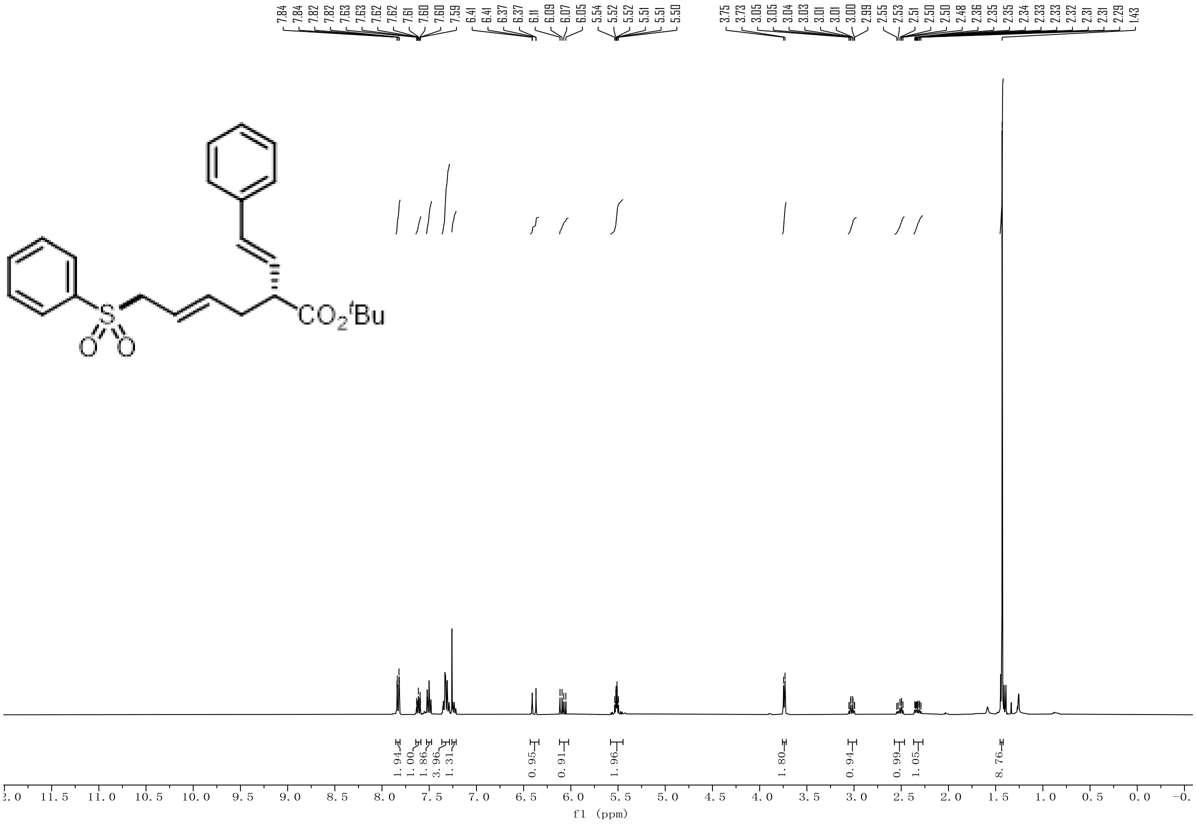

^{1}H NMR (400 MHz, $CDCl_3$, 298 K) spectrum of **18**

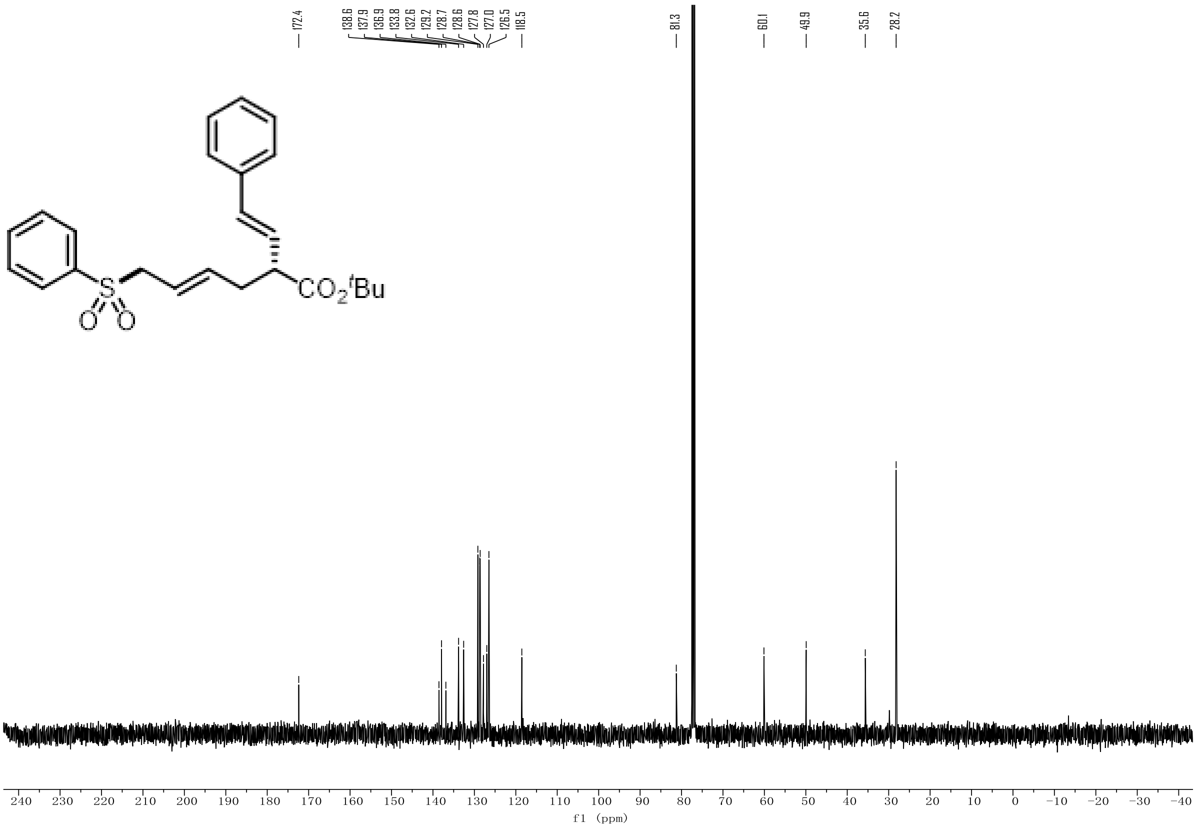

^{13}C NMR (101 MHz, $CDCl_3$, 298 K) spectrum of **18**

Methyl (*R,E*)-4-(1-((2,4-dimethylpentan-3-yl)oxy)-1-oxo-6-(phenylsulfonyl)hex-4-en-2-yl)benzoate (19)

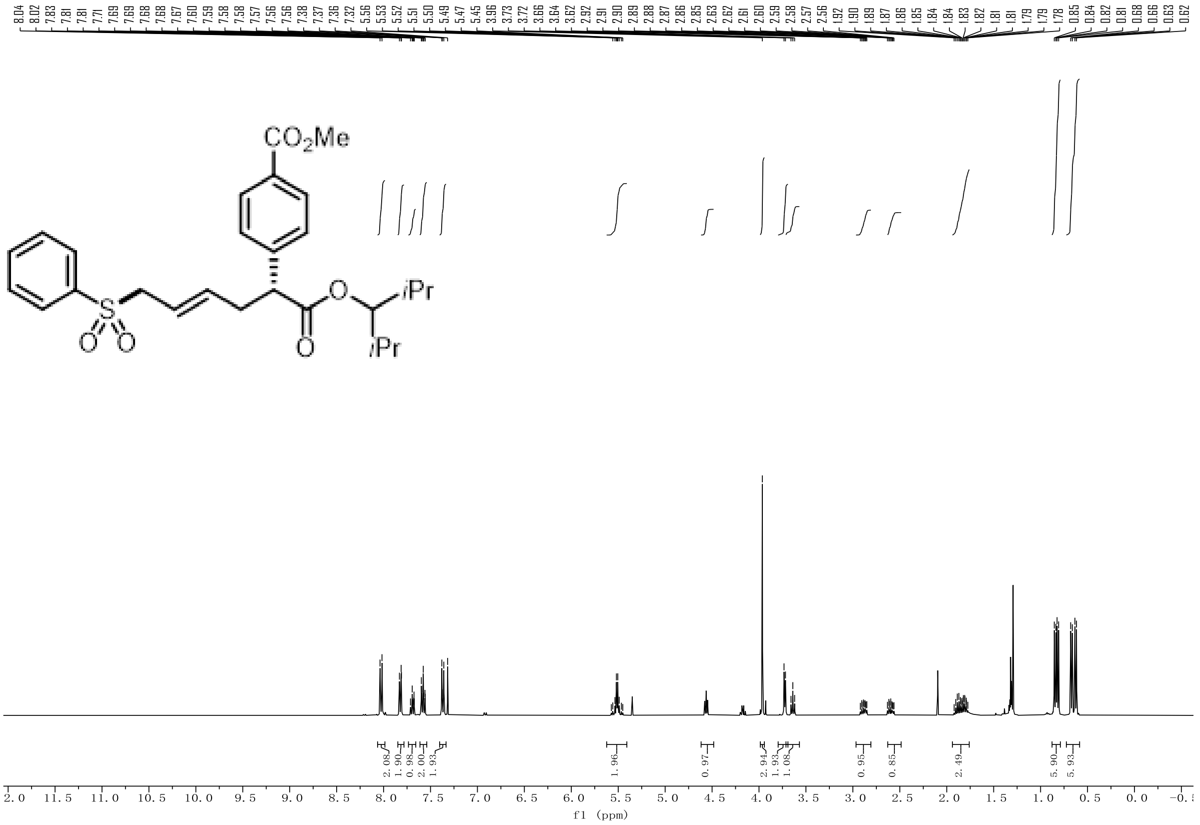

^{1}H NMR (400 MHz, $CDCl_3$, 298 K) spectrum of **19**

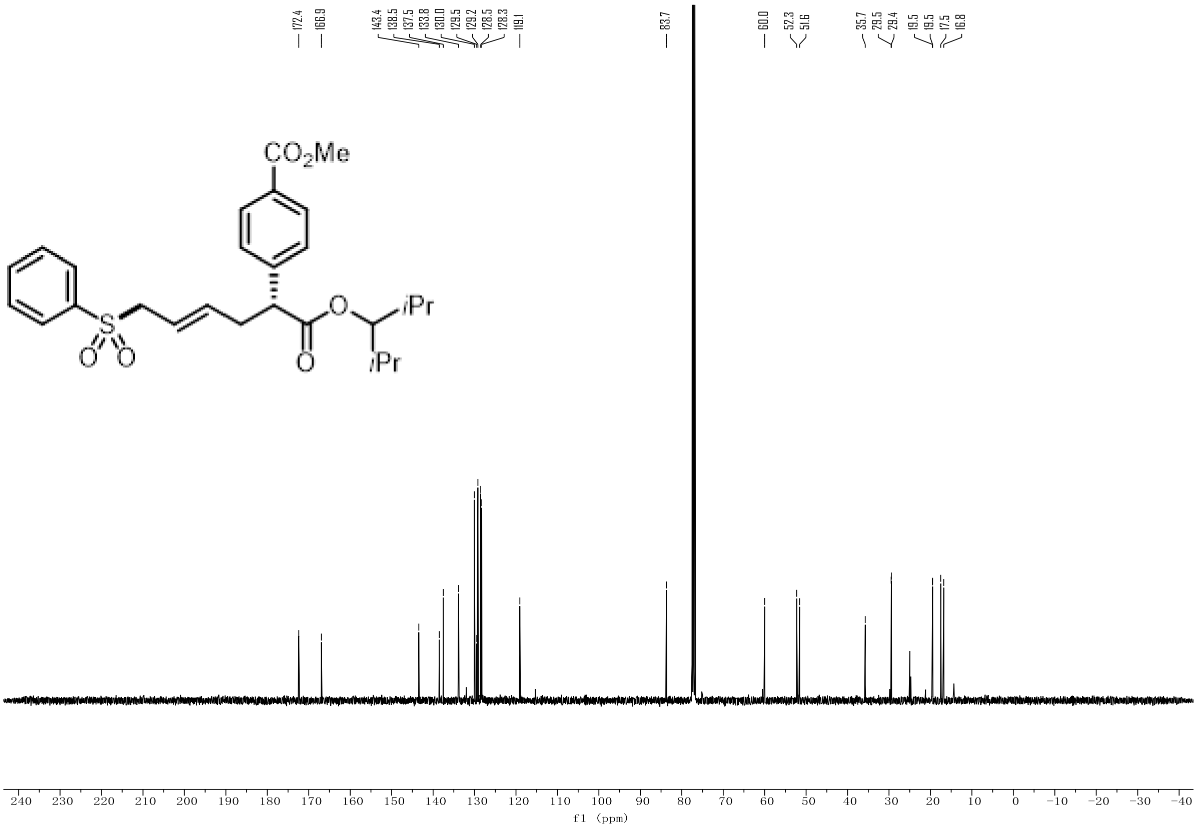

^{13}C NMR (101 MHz, $CDCl_3$, 298 K) spectrum of **19**

Methyl 4-((*R,E*)-1-(((3S,5S,7S)-adamantan-1-yl)oxy)-1-oxo-6-(phenylsulfonyl)hex-4-en-2-yl)benzoate (20)

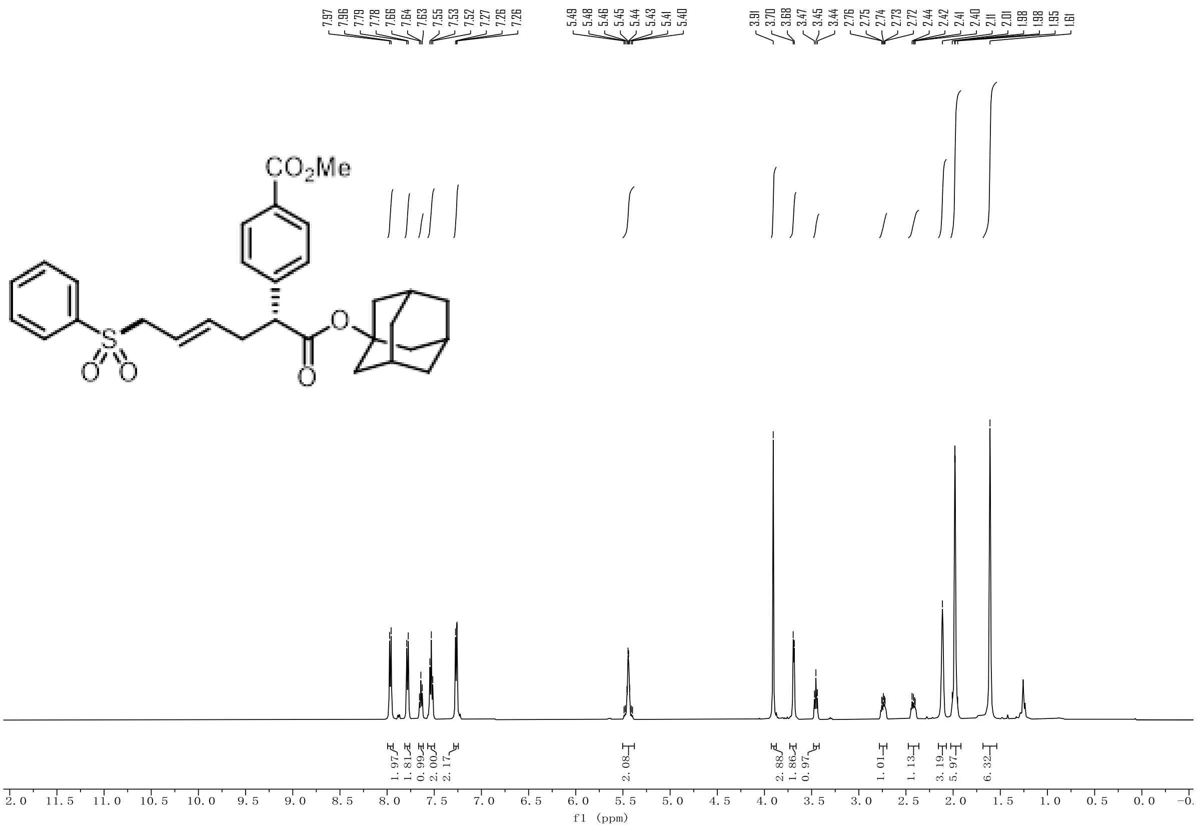

^{1}H NMR (500 MHz, $CDCl_3$, 298 K) spectrum of **20**

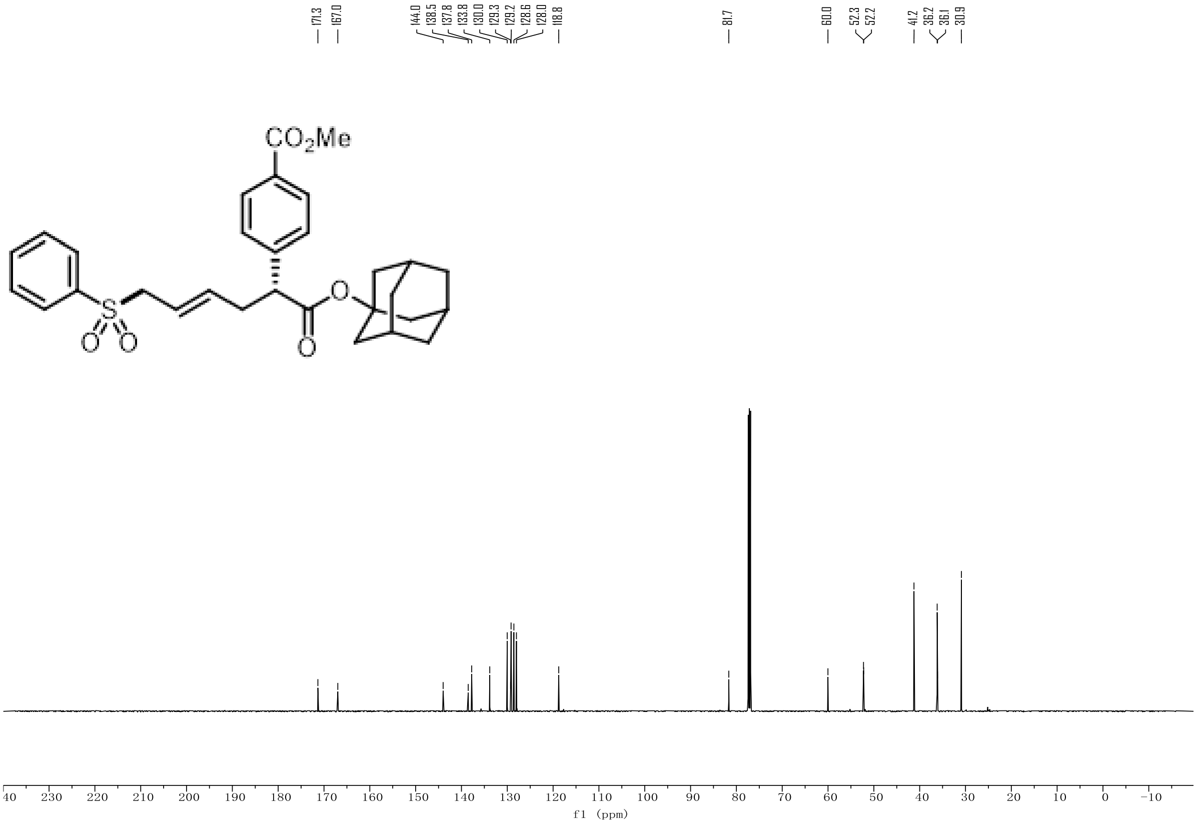

^{13}C NMR (126 MHz, $CDCl_3$, 298 K) spectrum of **20**

Methyl (*R*,*E*)-4-(1-((1-methylcyclohexyl)oxy)-1-oxo-6-(phenylsulfonyl)hex-4-en-2-yl)benzoate (21)

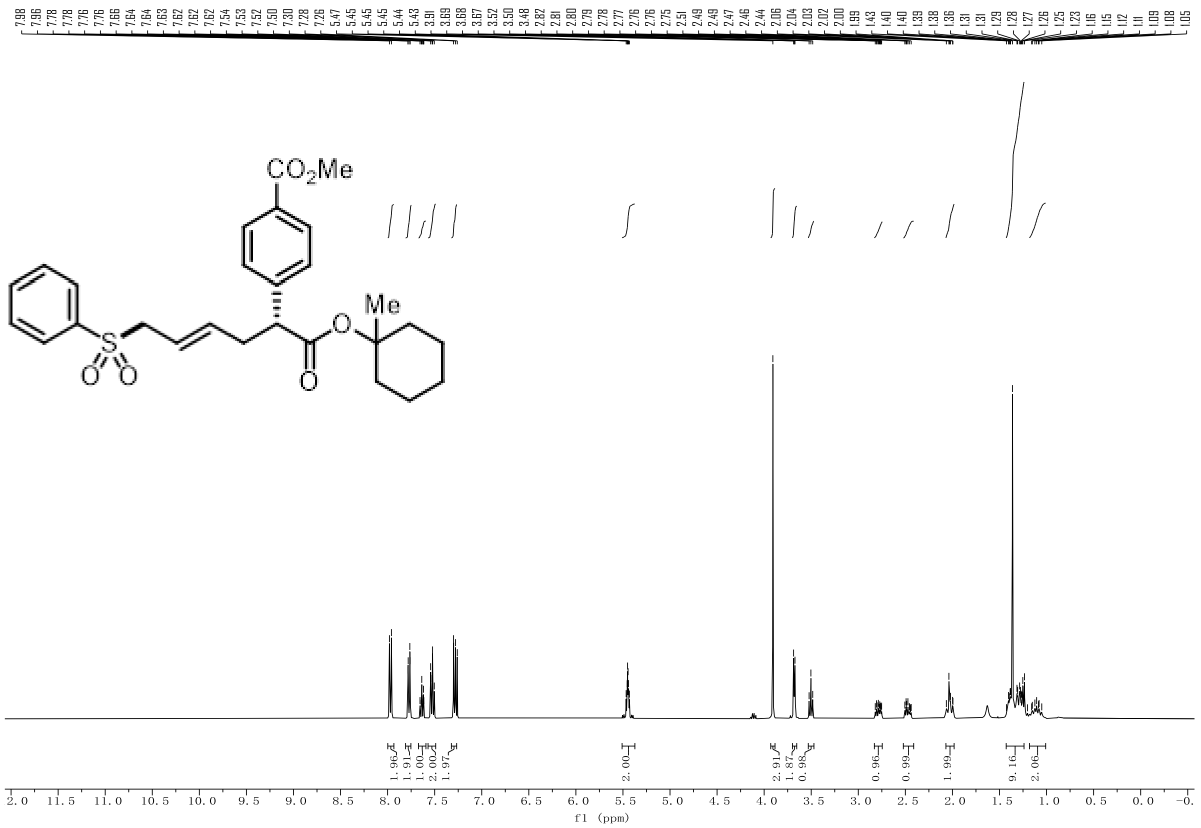

^{1}H NMR (400 MHz, $CDCl_3$, 298 K) spectrum of **21**

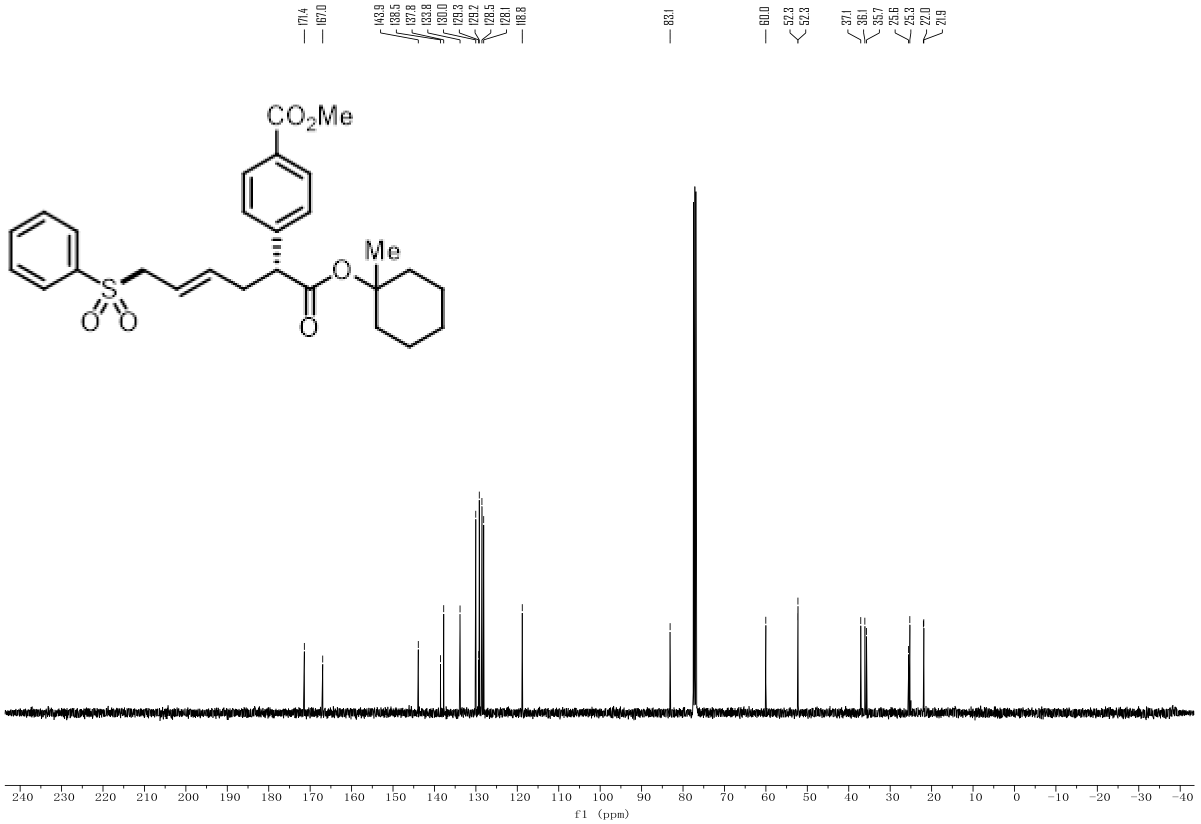

^{13}C NMR (101 MHz, $CDCl_3$, 298 K) spectrum of **21**

Methyl (*R,E*)-4-(1-((2-methyl-4-phenylbutan-2-yl)oxy)-1-oxo-6-(phenylsulfonyl)hex-4-en-2-yl)benzoate (22)

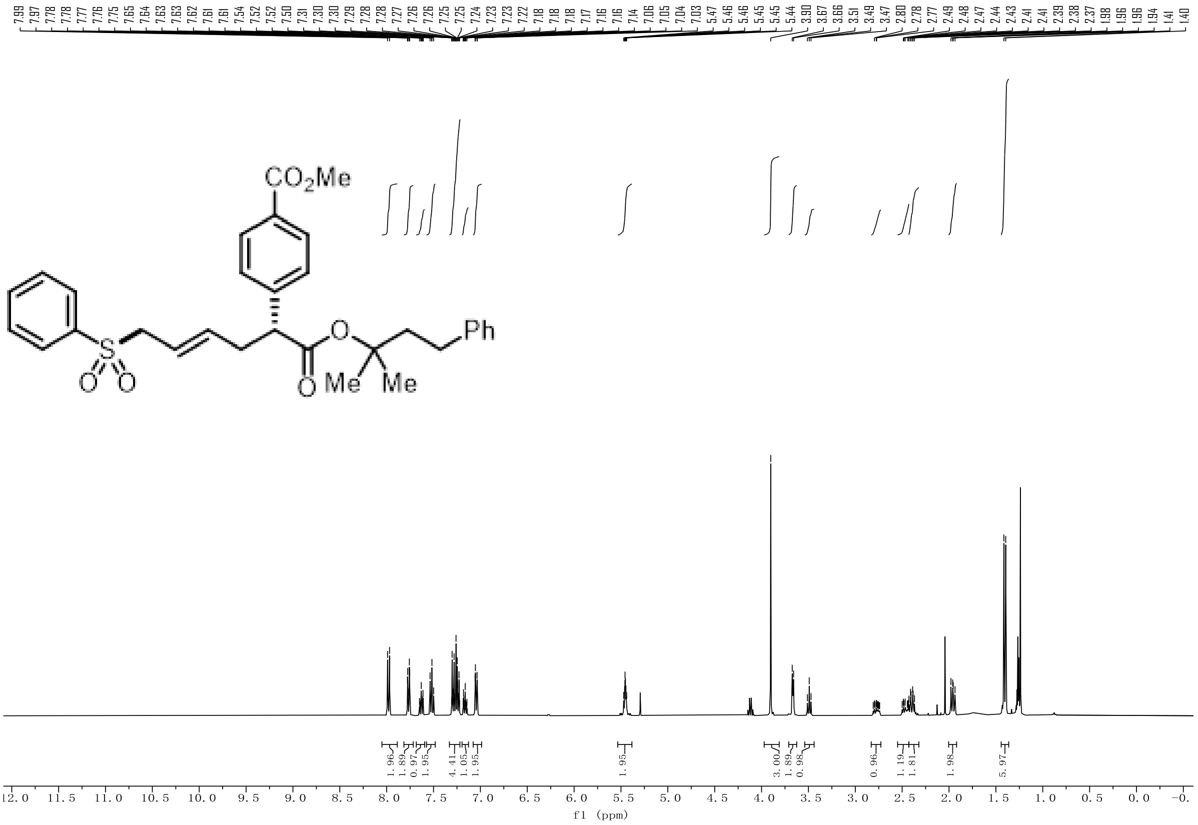

^{1}H NMR (400 MHz, CDCl$_3$, 298 K) spectrum of **22**

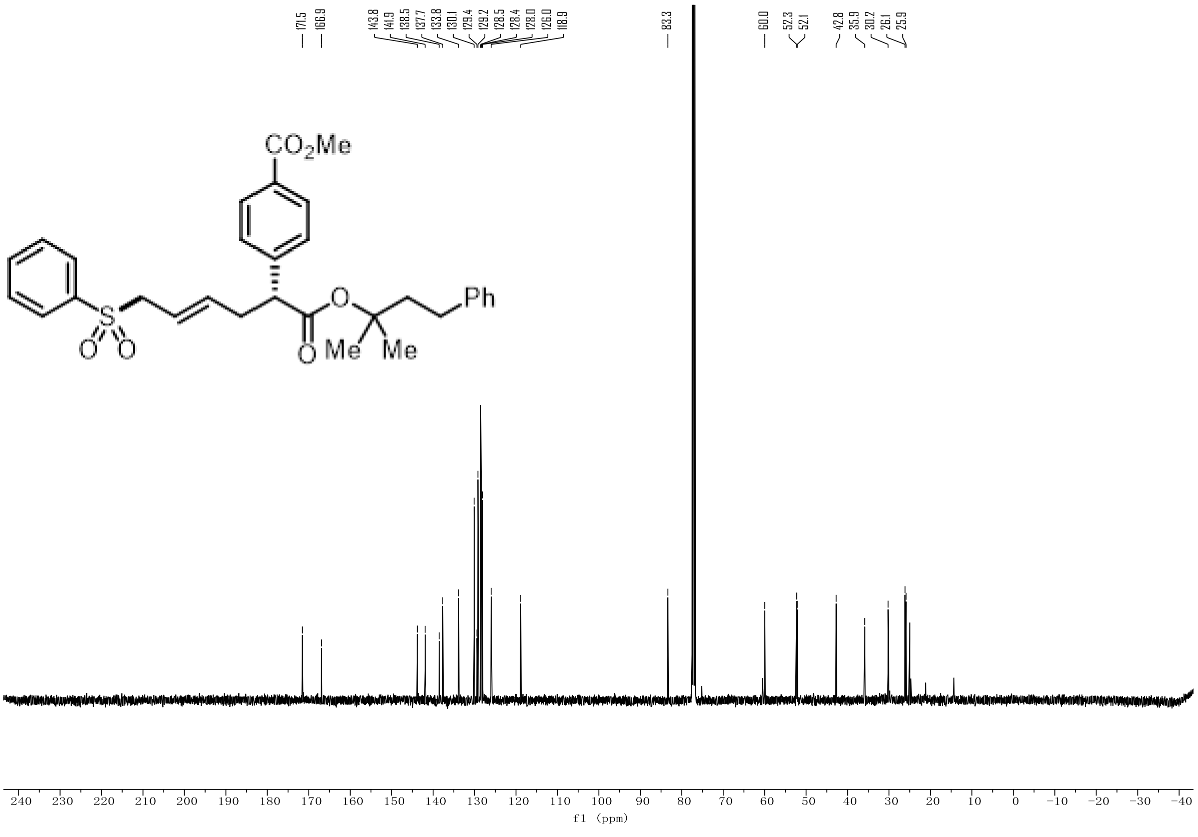

^{13}C NMR (101 MHz, CDCl$_3$, 298 K) spectrum of **22**

Methyl (*R,E*)-4-(1-(*tert*-butoxy)-5-methyl-1-oxo-6-(phenylsulfonyl)hex-4-en-2-yl)benzoate (23)

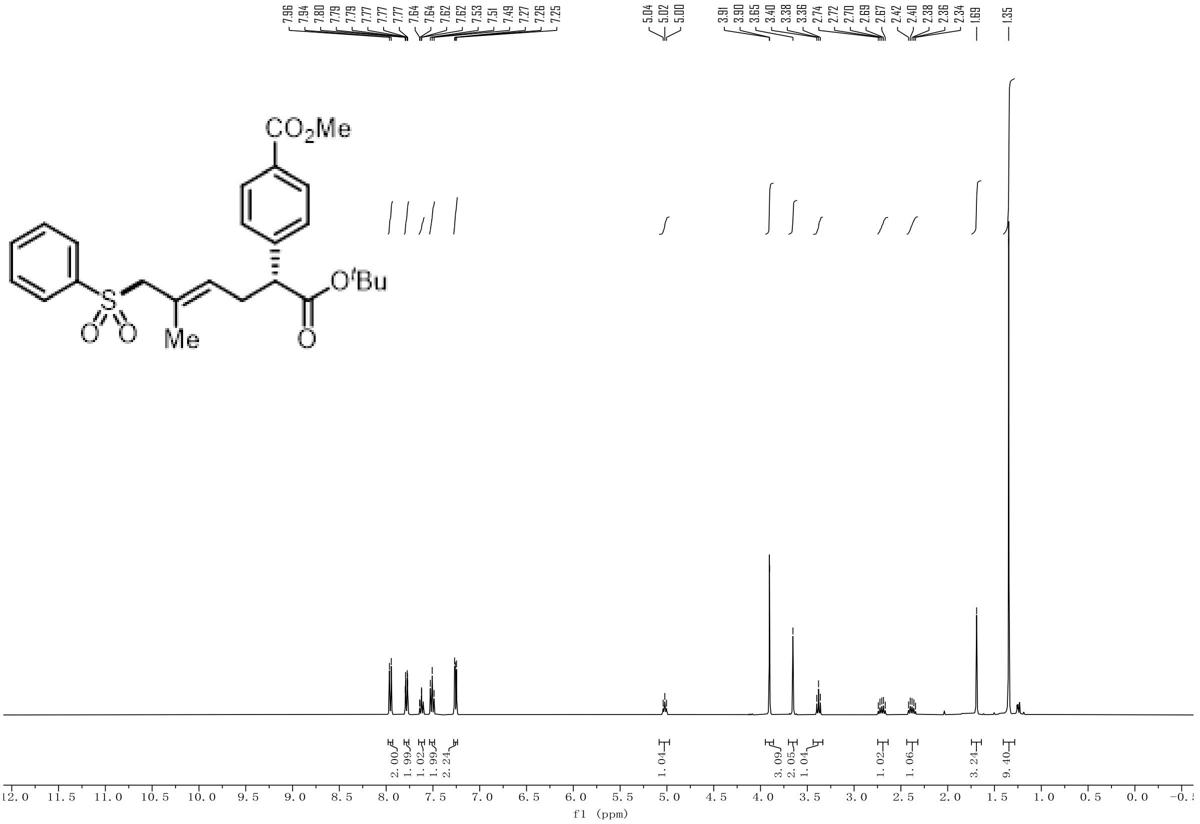

^{1}H NMR (400 MHz, $CDCl_3$, 298 K) spectrum of **23**

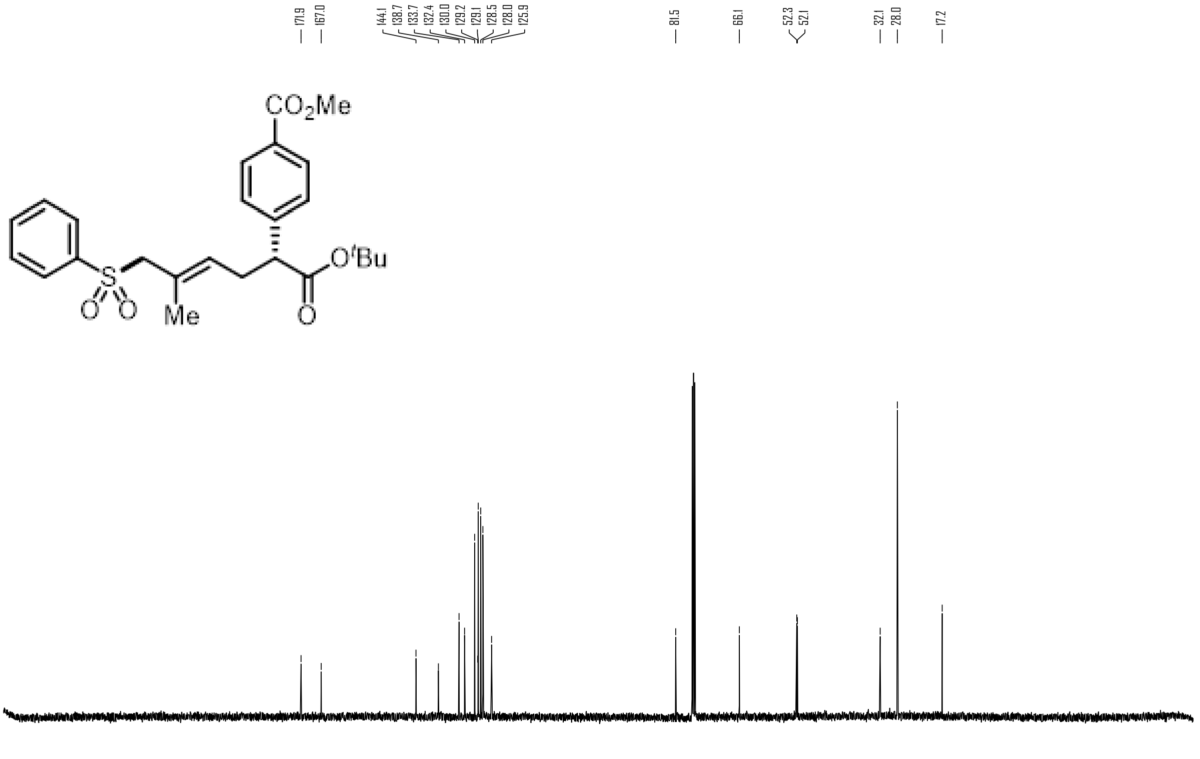

^{13}C NMR (101 MHz, $CDCl_3$, 298 K) spectrum of **23**

Methyl (*R,E*)-4-(1-(*tert*-butoxy)-1-oxo-5-phenyl-6-(phenylsulfonyl)hex-4-en-2-yl)benzoate (24)

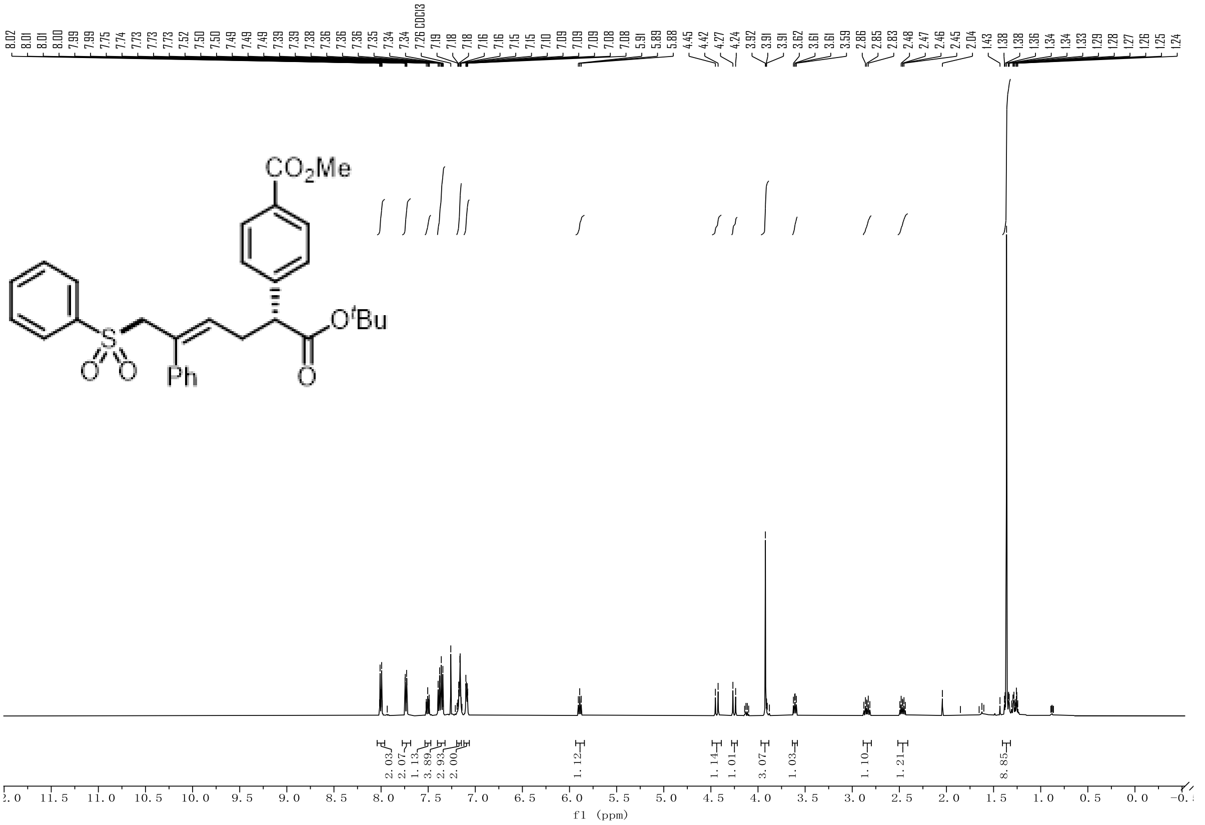

^{1}H NMR (500 MHz, $CDCl_3$, 298 K) spectrum of **24**

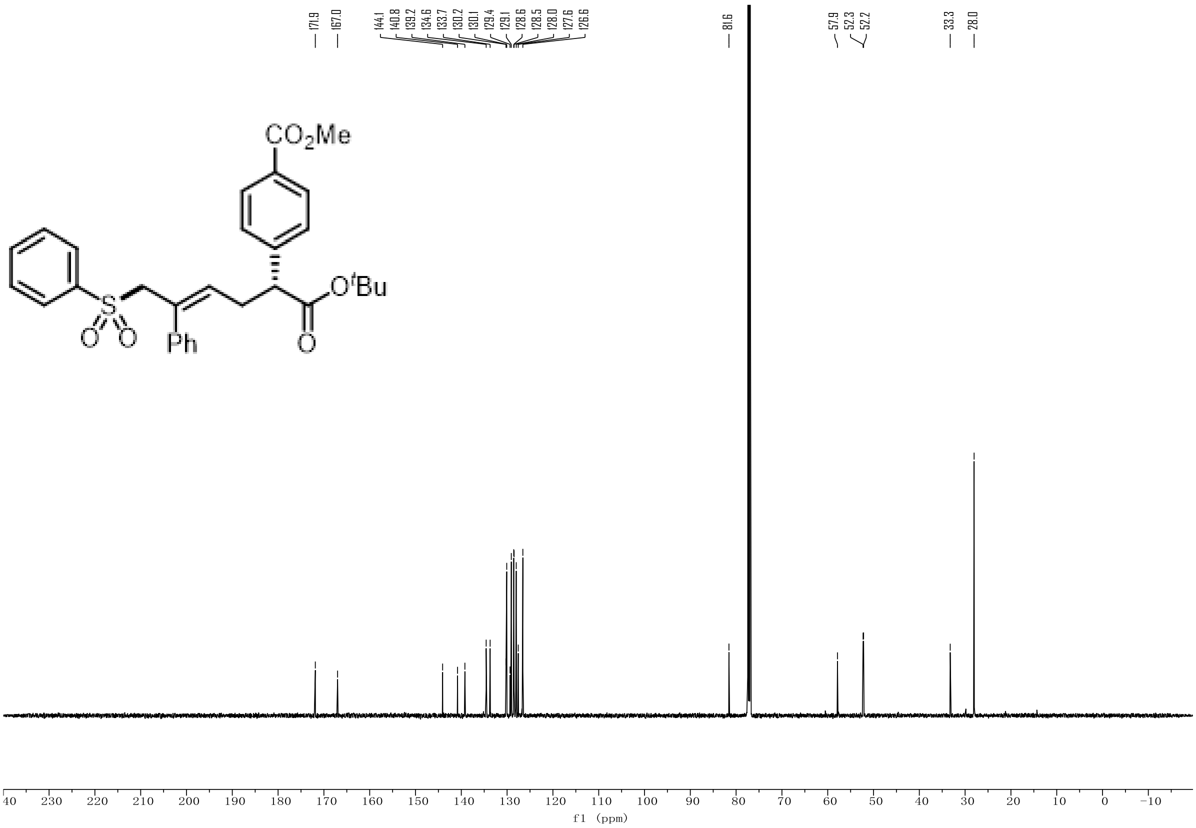

^{13}C NMR (126 MHz, $CDCl_3$, 298 K) spectrum of **24**

Methyl (*R*,*E*)-4-(1-(*tert*-butoxy)-4-methyl-1-oxo-6-(phenylsulfonyl)hex-4-en-2-yl)benzoate (25)

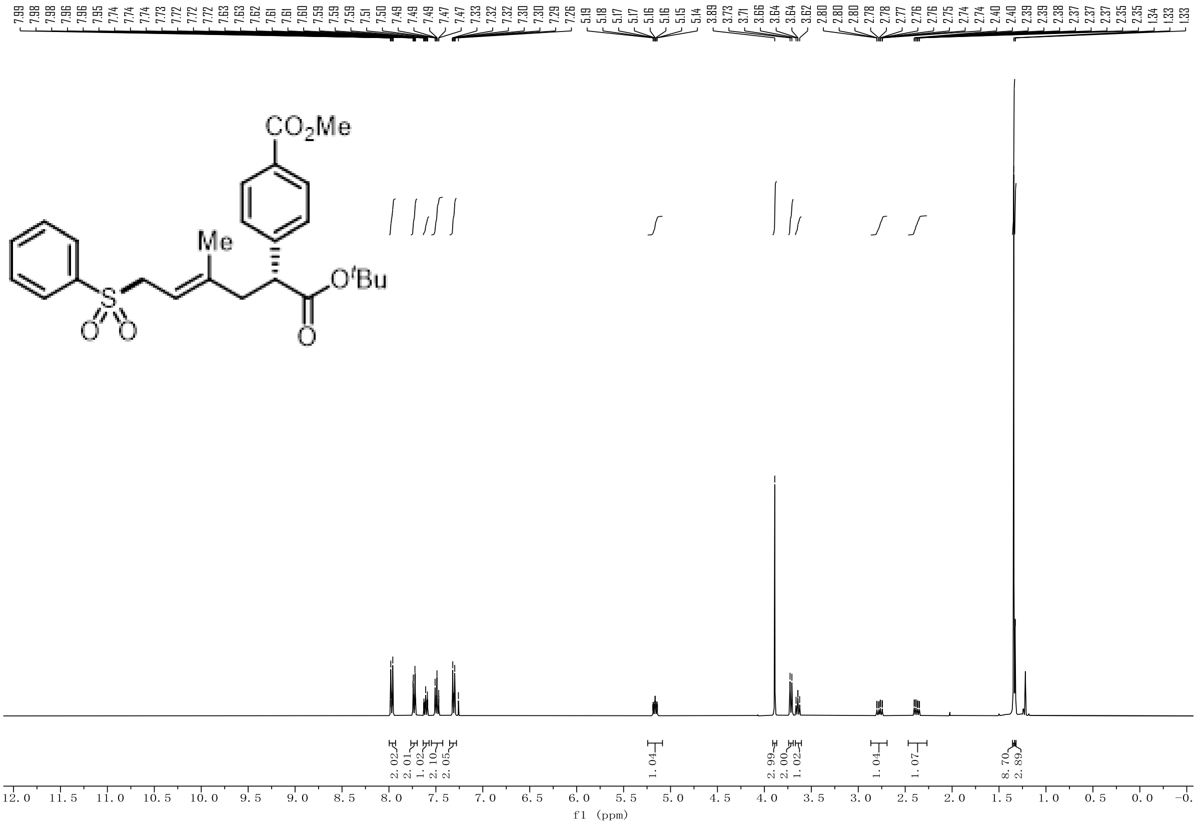

^{1}H NMR (400 MHz, $CDCl_3$, 298 K) spectrum of **25**

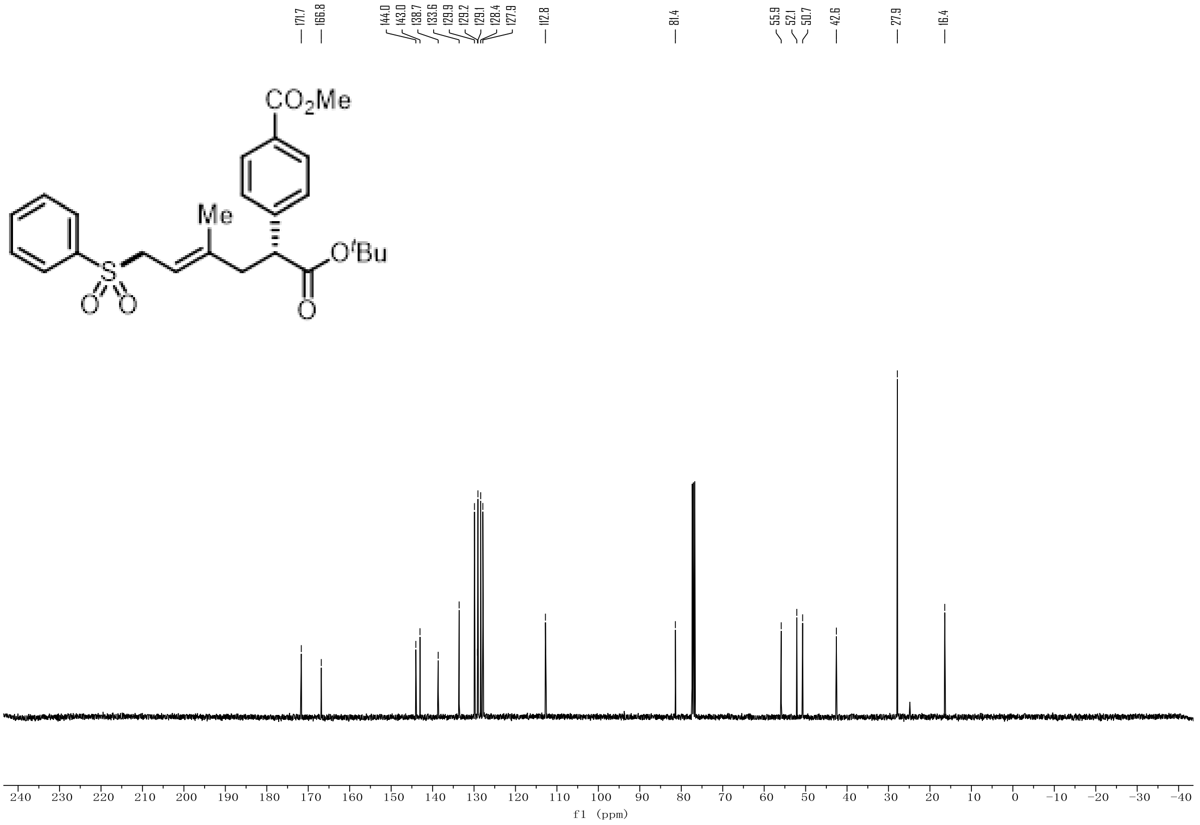

^{13}C NMR (101 MHz, $CDCl_3$, 298 K) spectrum of **25**

Methyl (*R,E*)-4-(1-(*tert*-butoxy)-4-methyl-1-oxo-6-(phenylsulfonyl)hex-4-en-2-yl)benzoate (26)

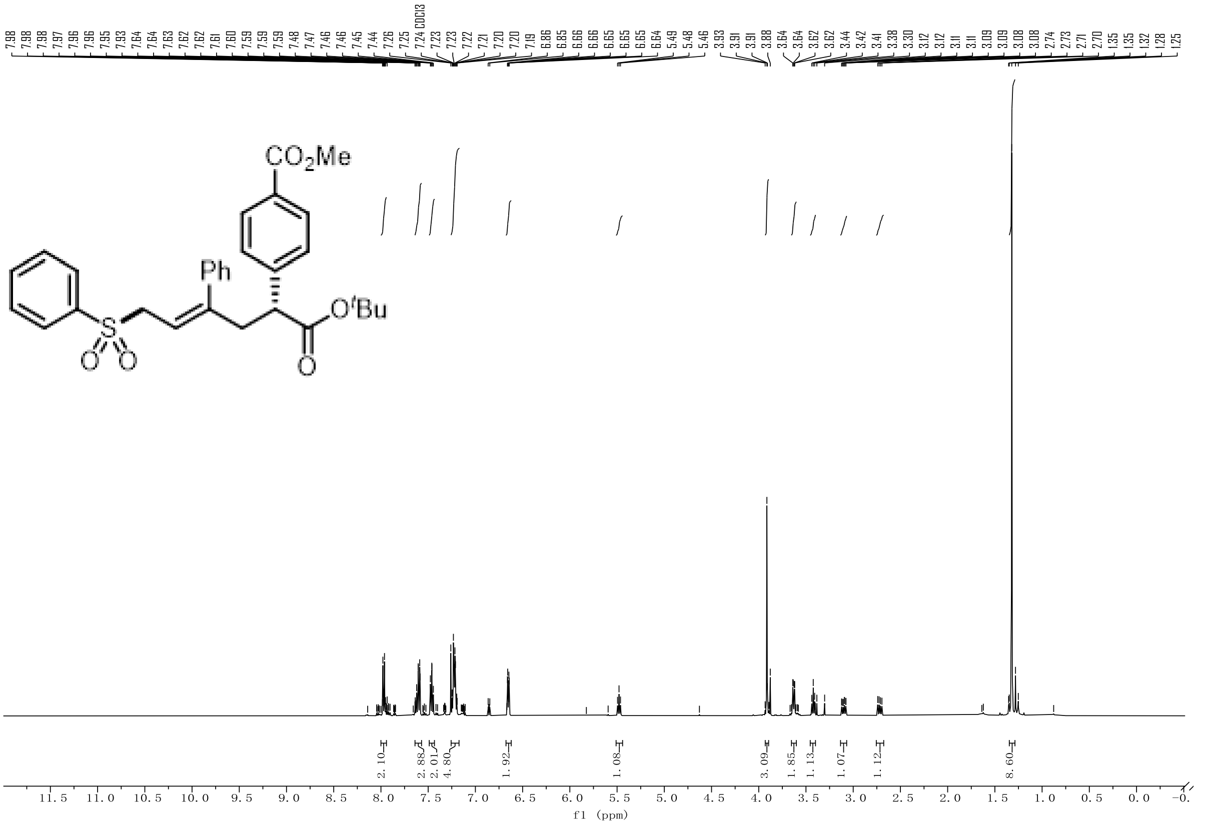

^{1}H NMR (500 MHz, CDCl$_3$, 298 K) spectrum of **26**

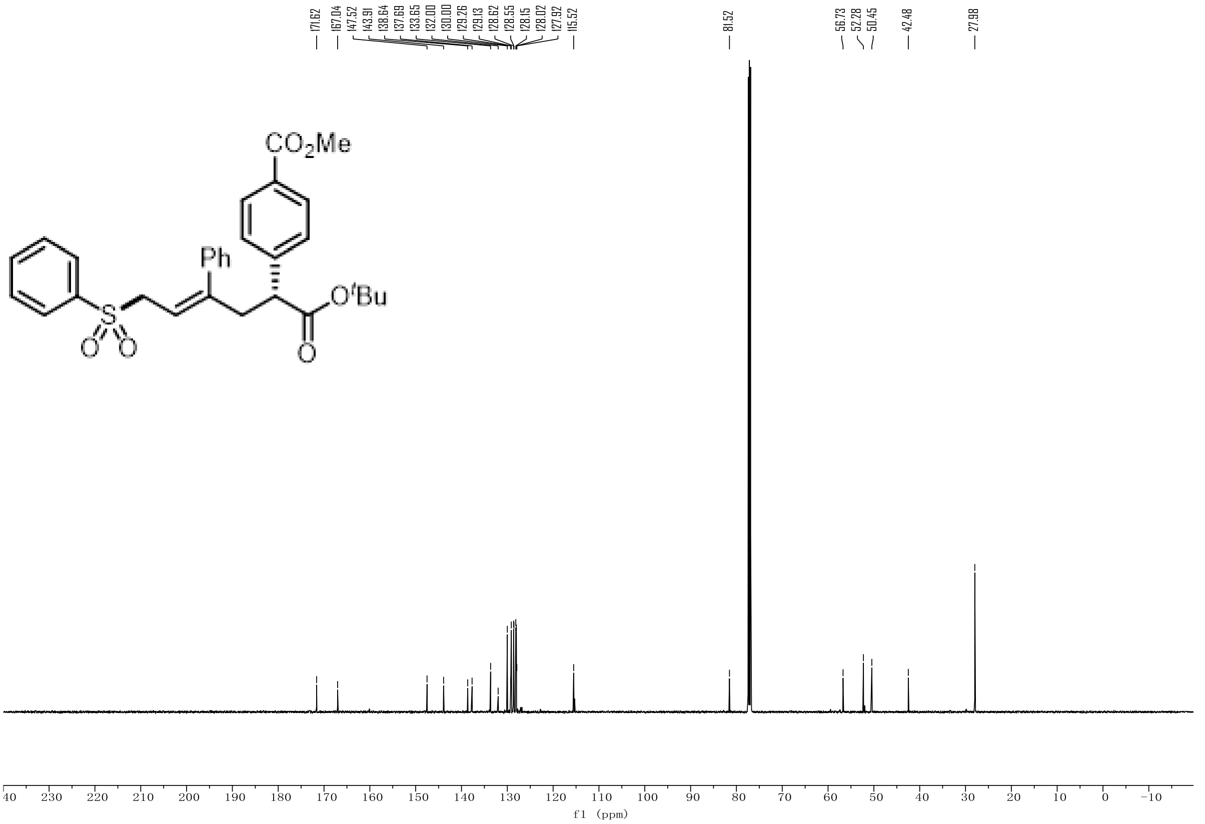

^{13}C NMR (126 MHz, CDCl$_3$, 298 K) spectrum of **26**

Methyl (*R,E*)-4-(1-(*tert*-butylamino)-1-oxo-6-(phenylsulfonyl)hex-4-en-2-yl)benzoate (27)

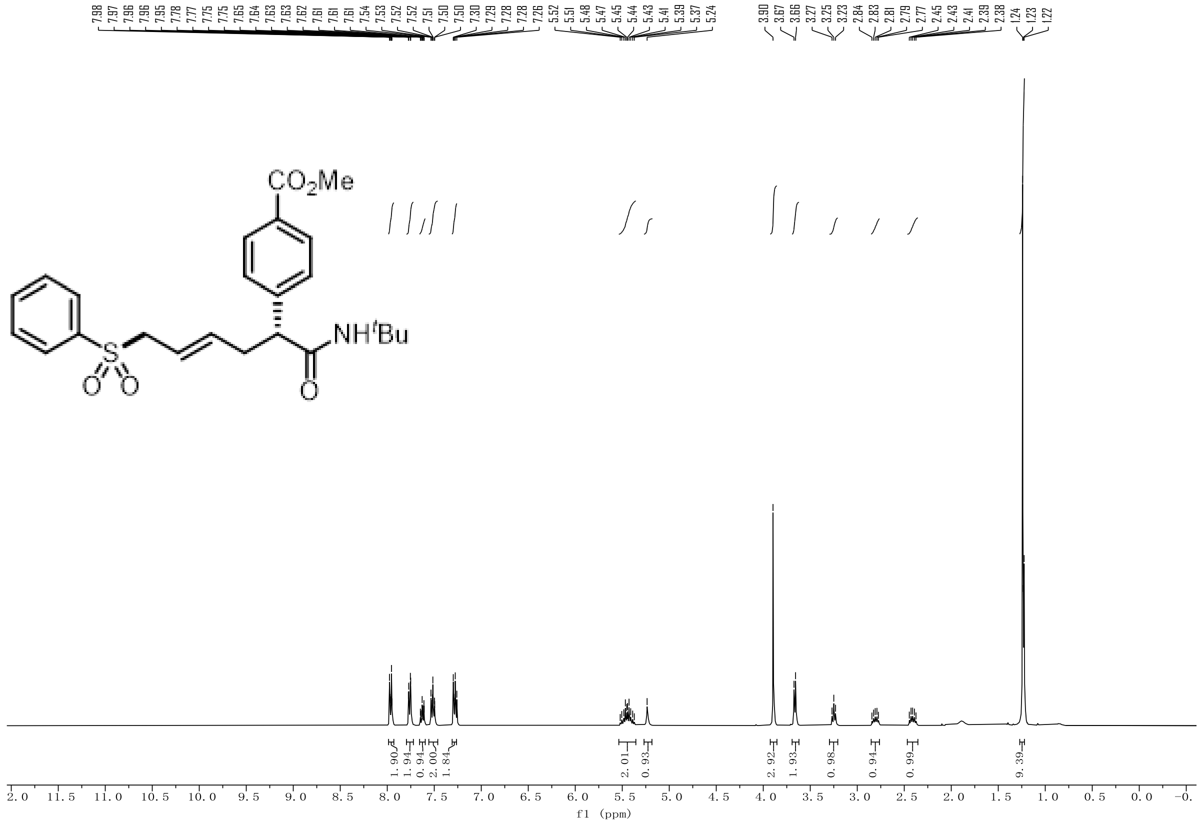

^{1}H NMR (400 MHz, $CDCl_3$, 298 K) spectrum of **27**

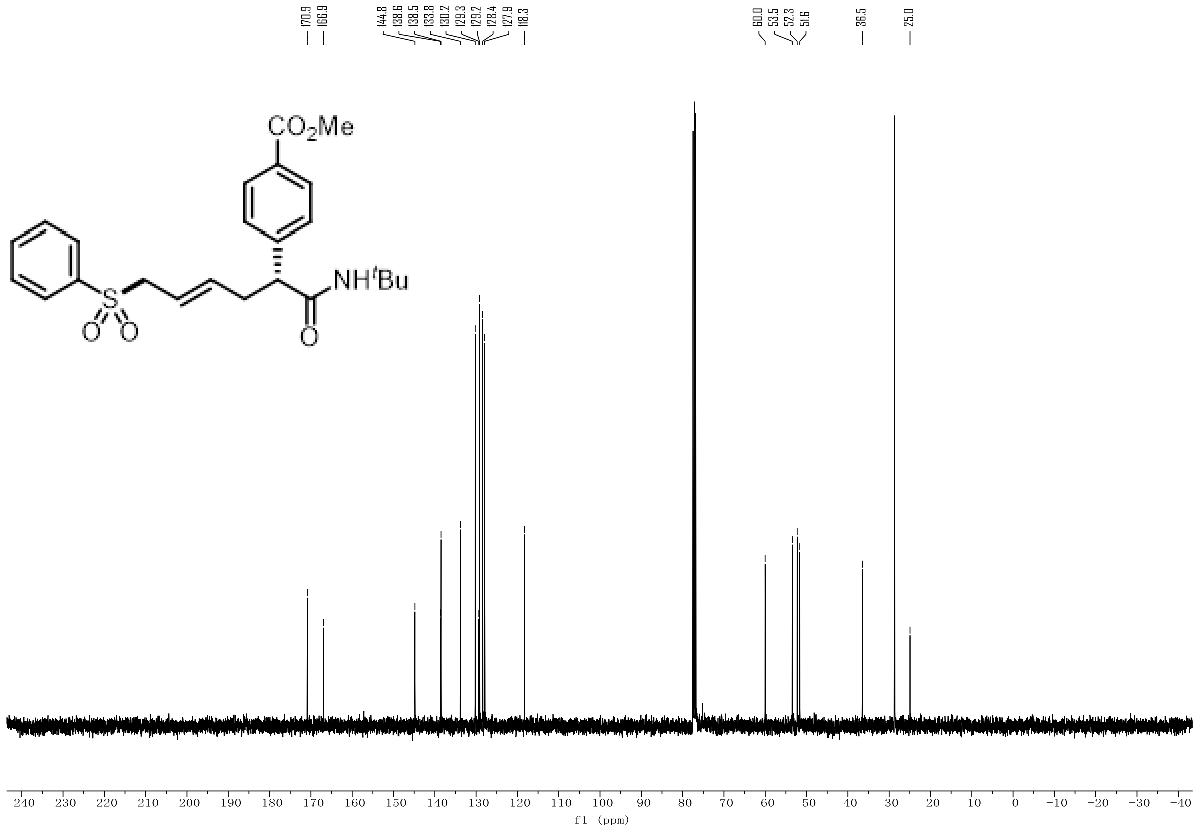

^{13}C NMR (101 MHz, $CDCl_3$, 298 K) spectrum of **27**

Methyl (*R,E*)-4-(1-oxo-1-phenyl-6-(phenylsulfonyl)hex-4-en-2-yl)benzoate (28)

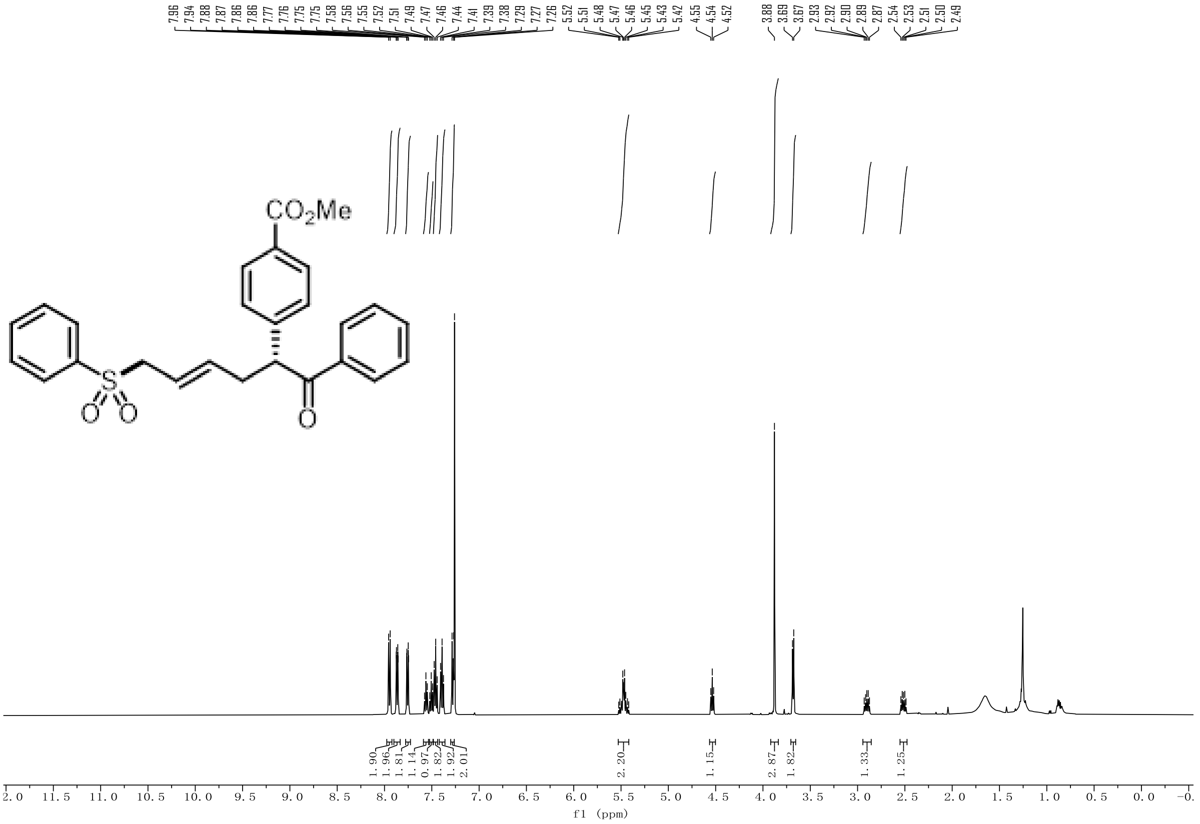

^{1}H NMR (500 MHz, $CDCl_3$, 298 K) spectrum of **28**

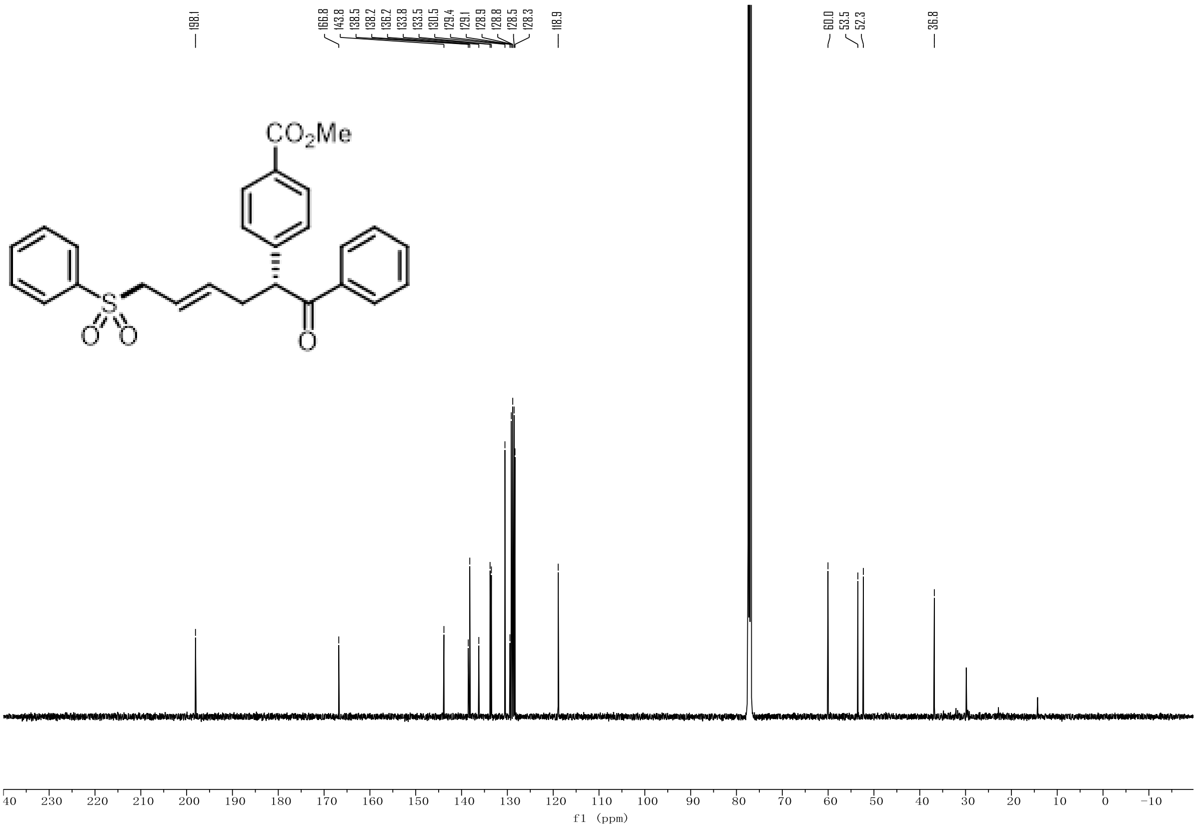

^{13}C NMR (126 MHz, $CDCl_3$, 298 K) spectrum of **28**

Methyl (*S,E*)-4-(1-phenyl-5-(phenylsulfonyl)pent-3-en-1-yl)benzoate (29)

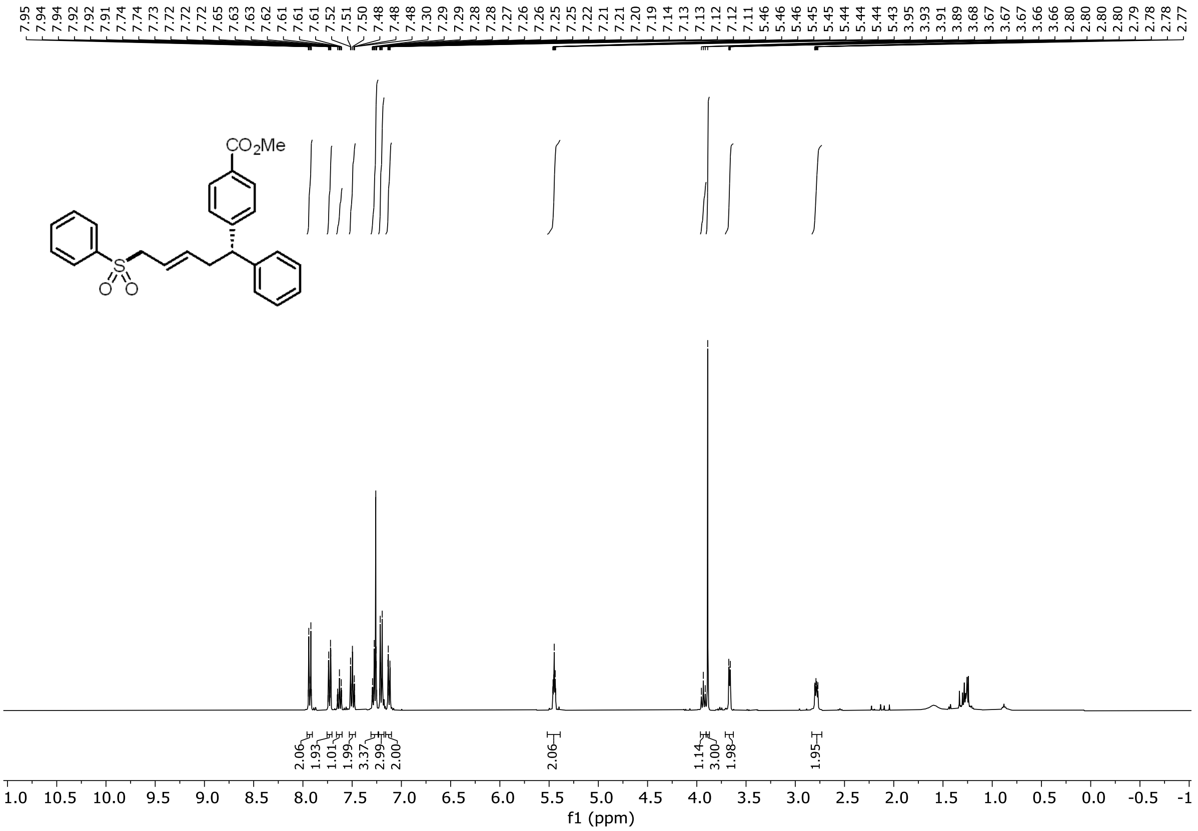

^{1}H NMR (500 MHz, $CDCl_3$, 298 K) spectrum of **29**

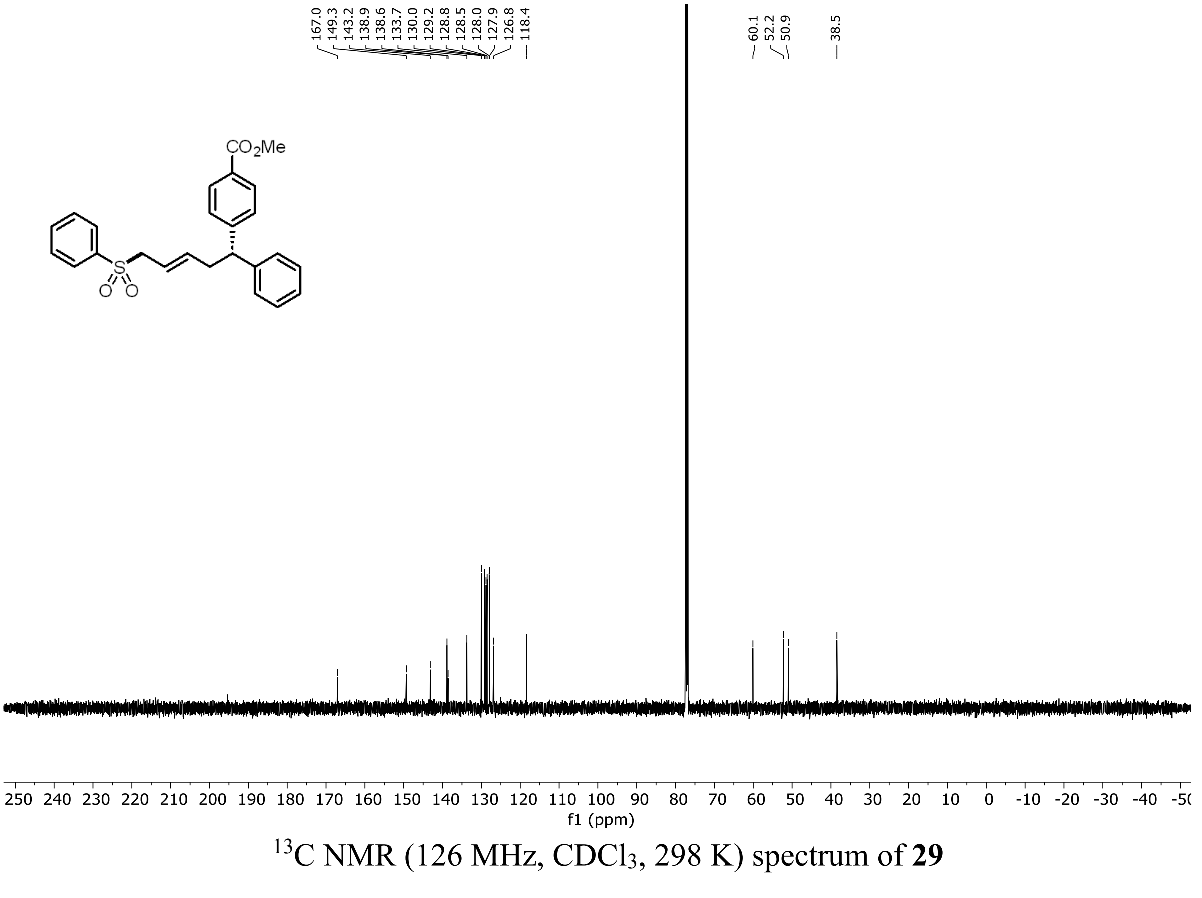

^{13}C NMR (126 MHz, $CDCl_3$, 298 K) spectrum of **29**

(*S,E*)-Phenyl(4-(1-phenyl-5-(phenylsulfonyl)pent-3-en-1-yl)phenyl)methanone (30)

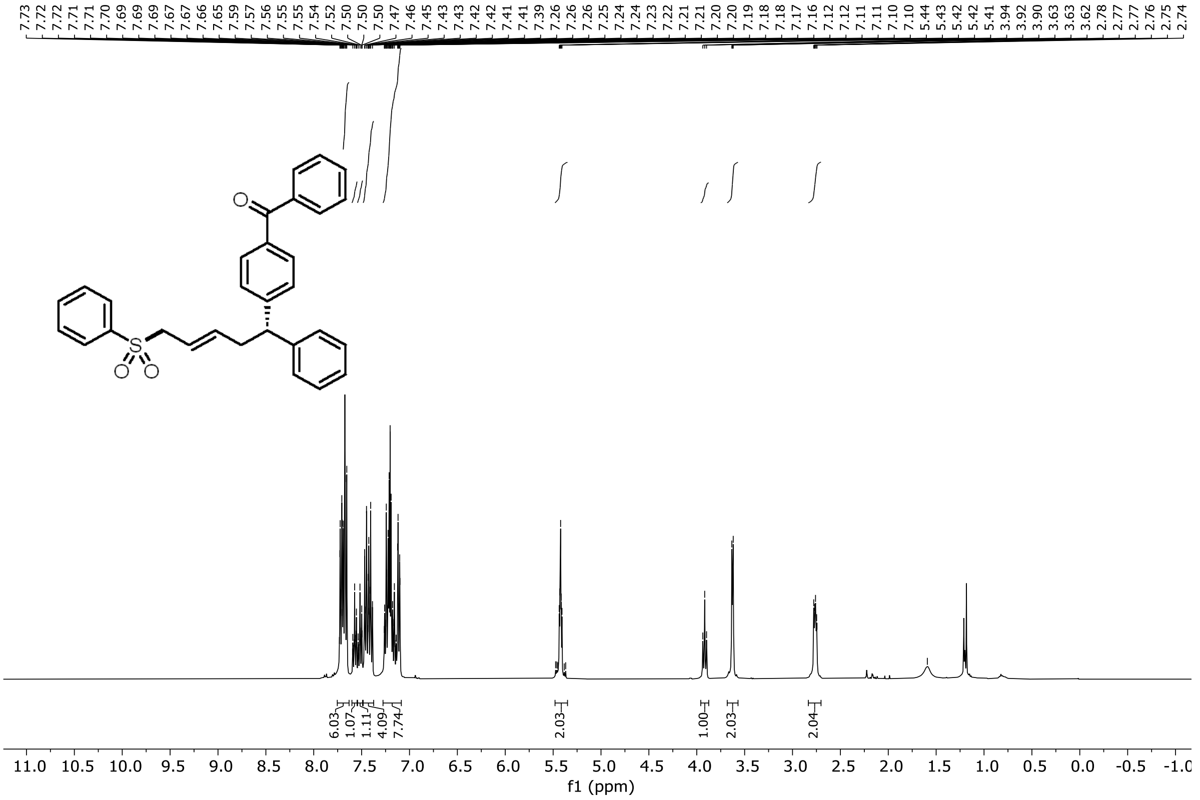

^{1}H NMR (400 MHz, CDCl$_3$, 298 K) spectrum of **30**

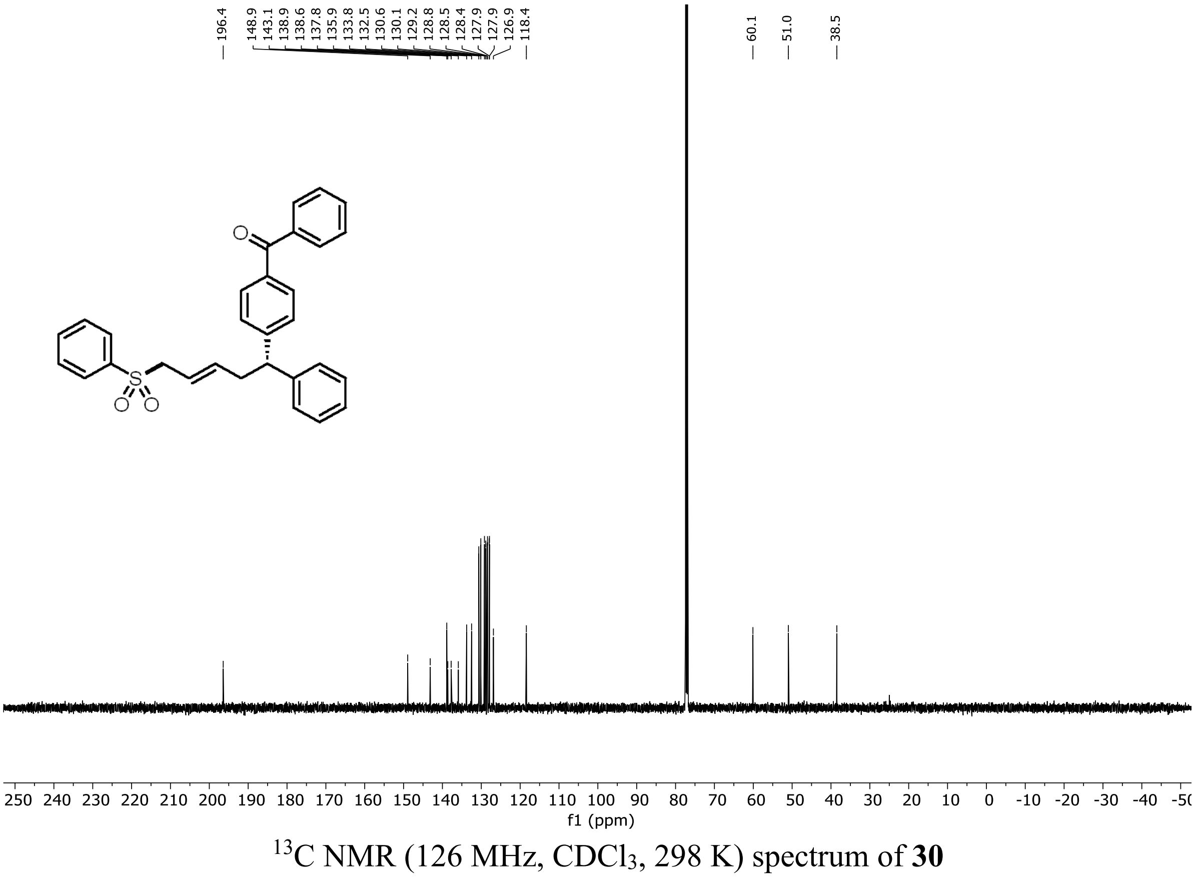

^{13}C NMR (126 MHz, CDCl$_3$, 298 K) spectrum of **30**

(*S,E*)-5-(1-Phenyl-5-(phenylsulfonyl)pent-3-en-1-yl)isobenzofuran-1(3H)-one (31)

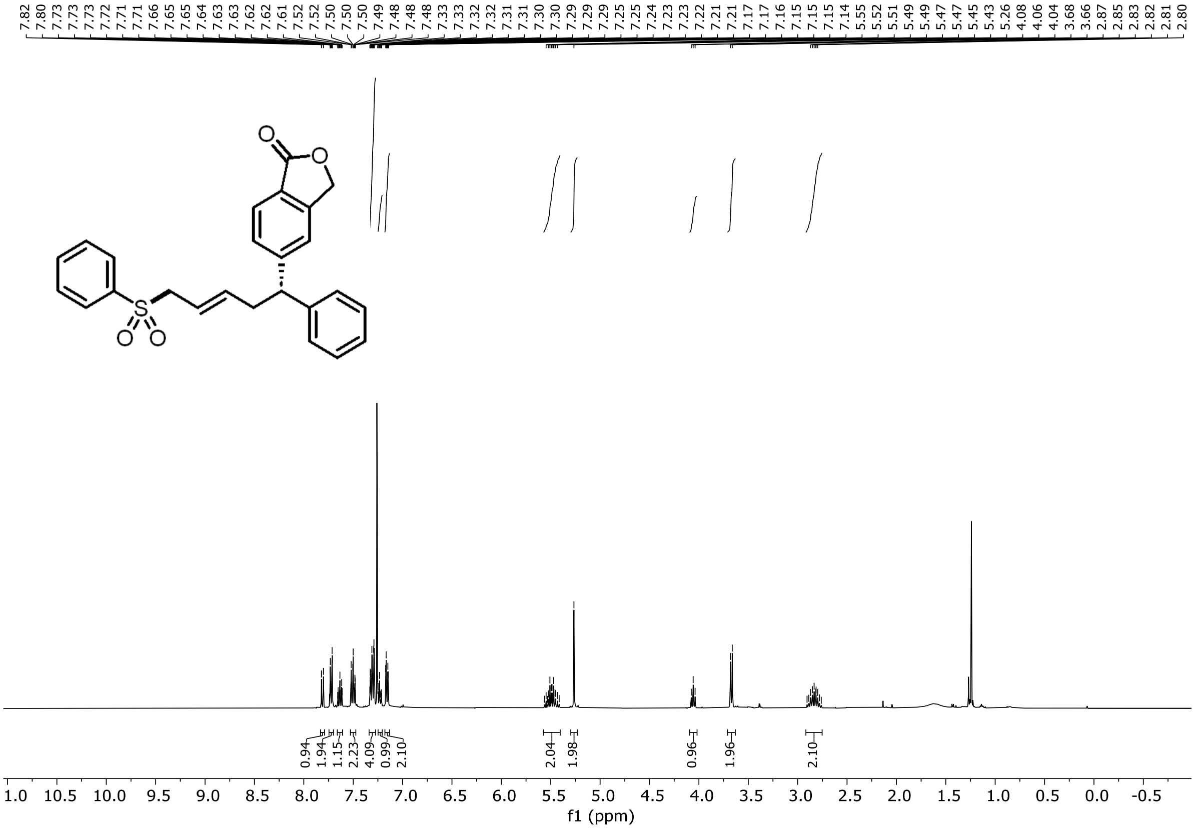

^{1}H NMR (500 MHz, $CDCl_3$, 298 K) spectrum of **31**

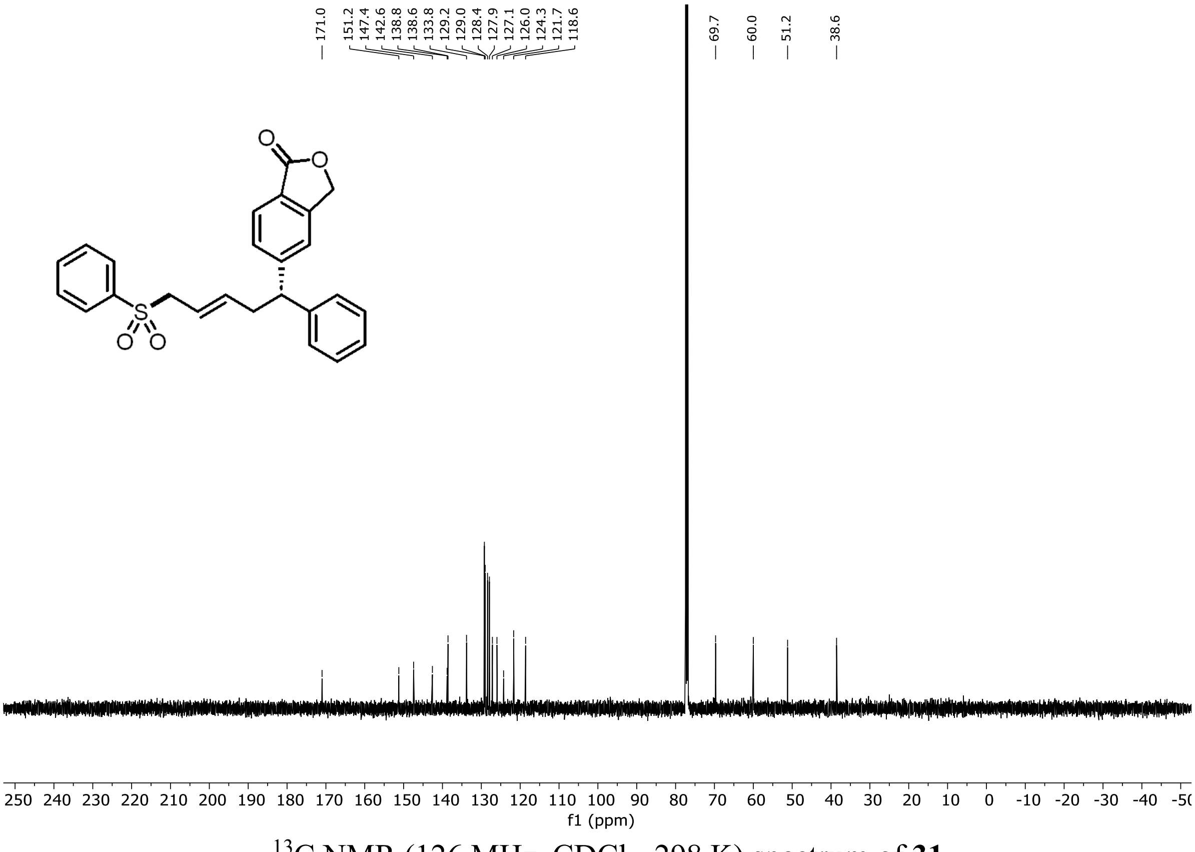

^{13}C NMR (126 MHz, $CDCl_3$, 298 K) spectrum of **31**

Methyl (*S,E*)-4-(4-methyl-1-phenyl-5-(phenylsulfonyl)pent-3-en-1-yl)benzoate (32)

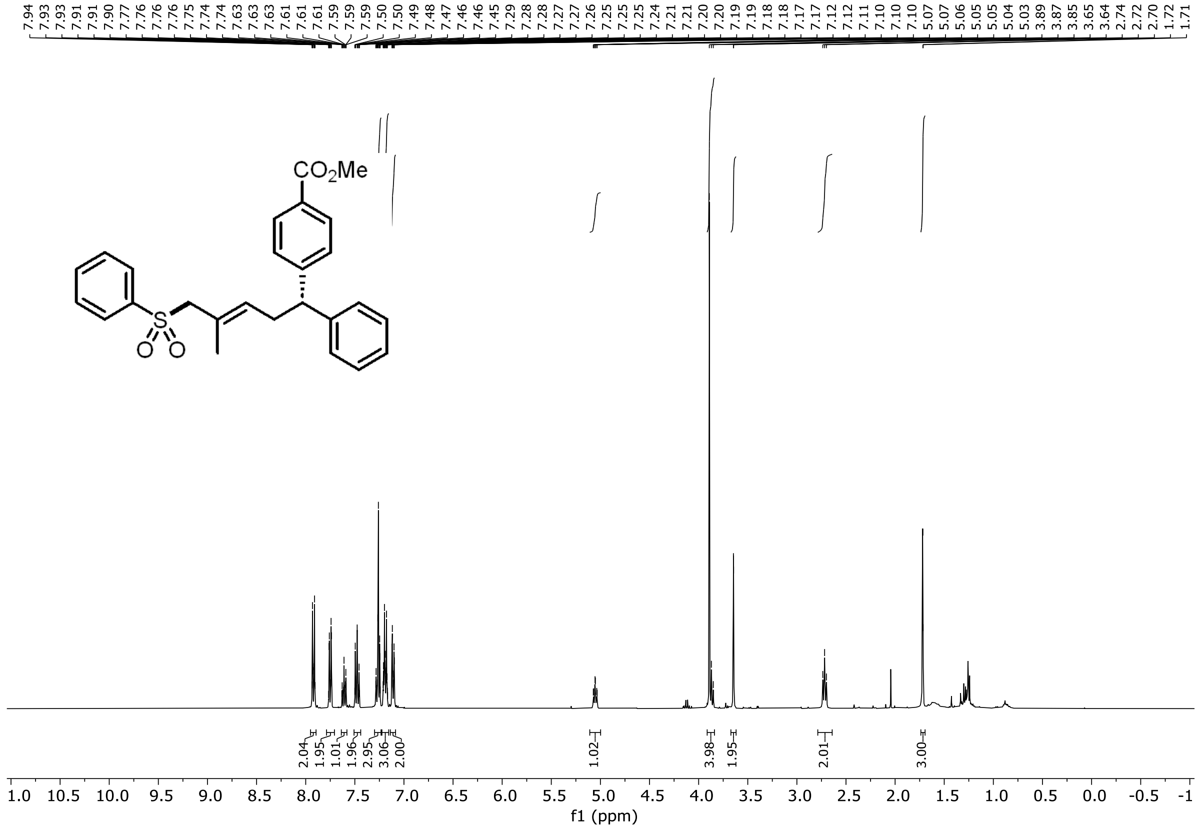

^{1}H NMR (500 MHz, CDCl$_3$, 298 K) spectrum of **32**

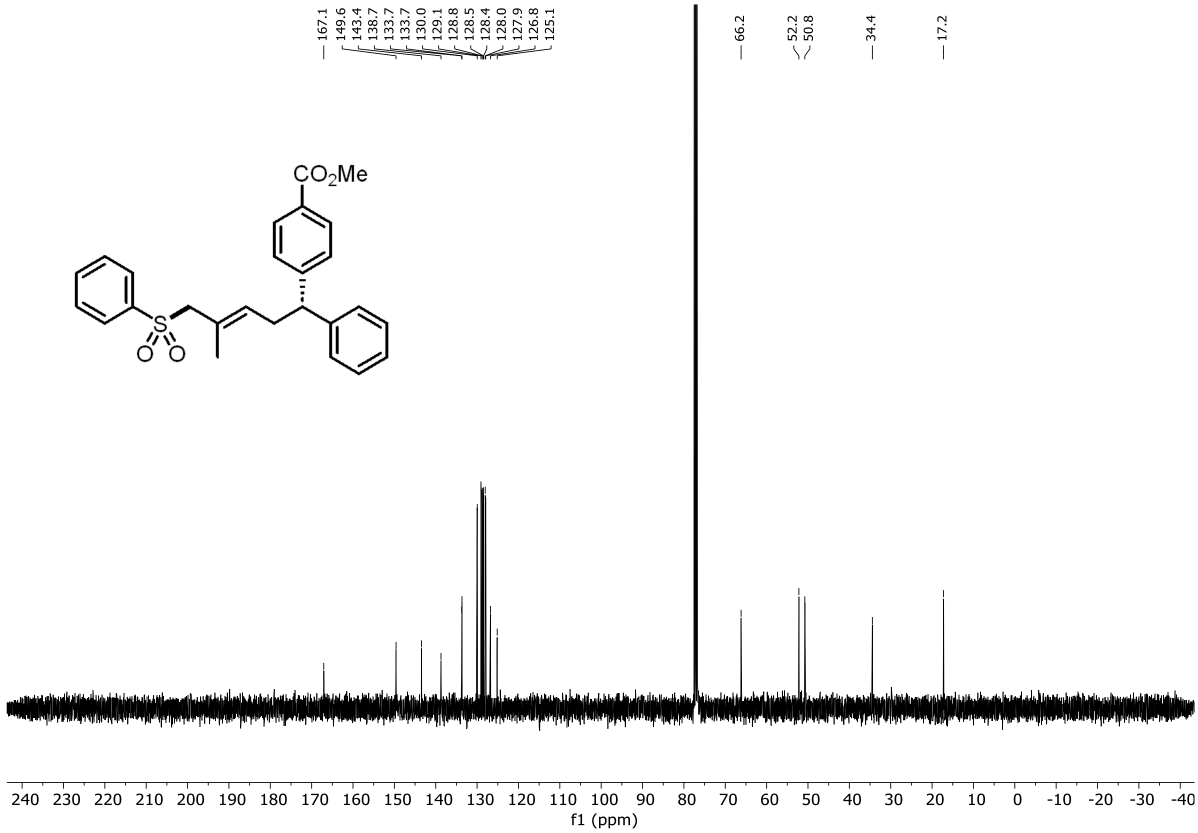

^{13}C NMR (126 MHz, CDCl$_3$, 298 K) spectrum of **32**

Methyl (*R*,*E*)-4-(1-(4-chlorophenyl)-4-methyl-5-(phenylsulfonyl)pent-3-en-1-yl)benzoate (33)

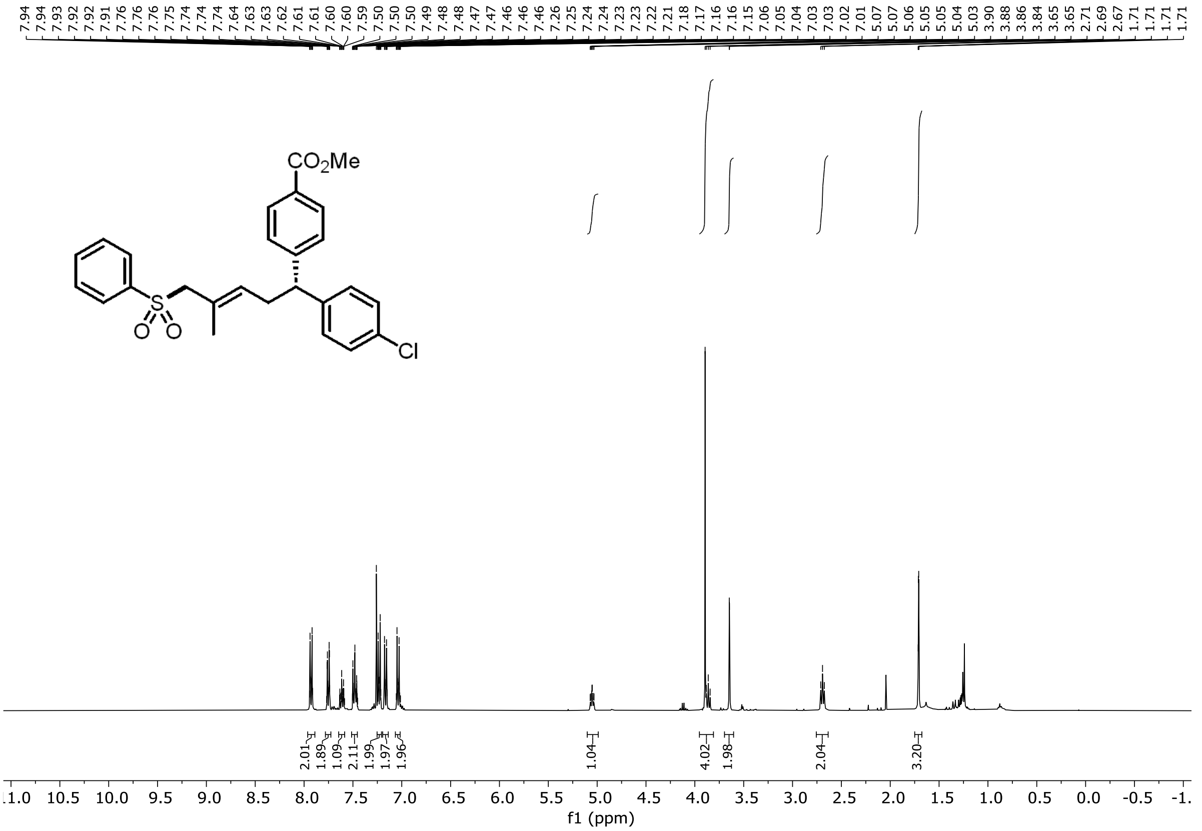

^{1}H NMR (500 MHz, $CDCl_3$, 298 K) spectrum of **33**

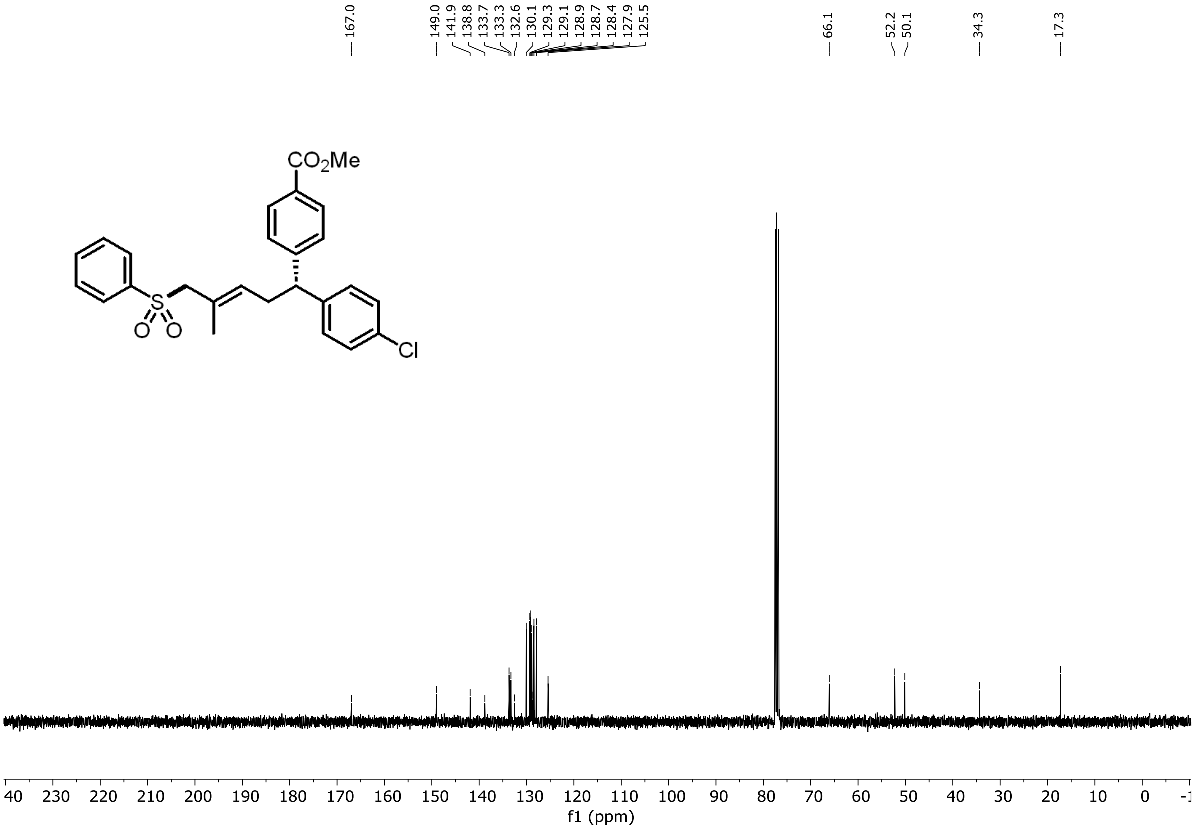

^{13}C NMR (126 MHz, $CDCl_3$, 298 K) spectrum of **33**

Methyl (*R,E*)-4-(1-(*tert*-butoxy)-1-oxo-6-tosylhex-4-en-2-yl)benzoate (34)

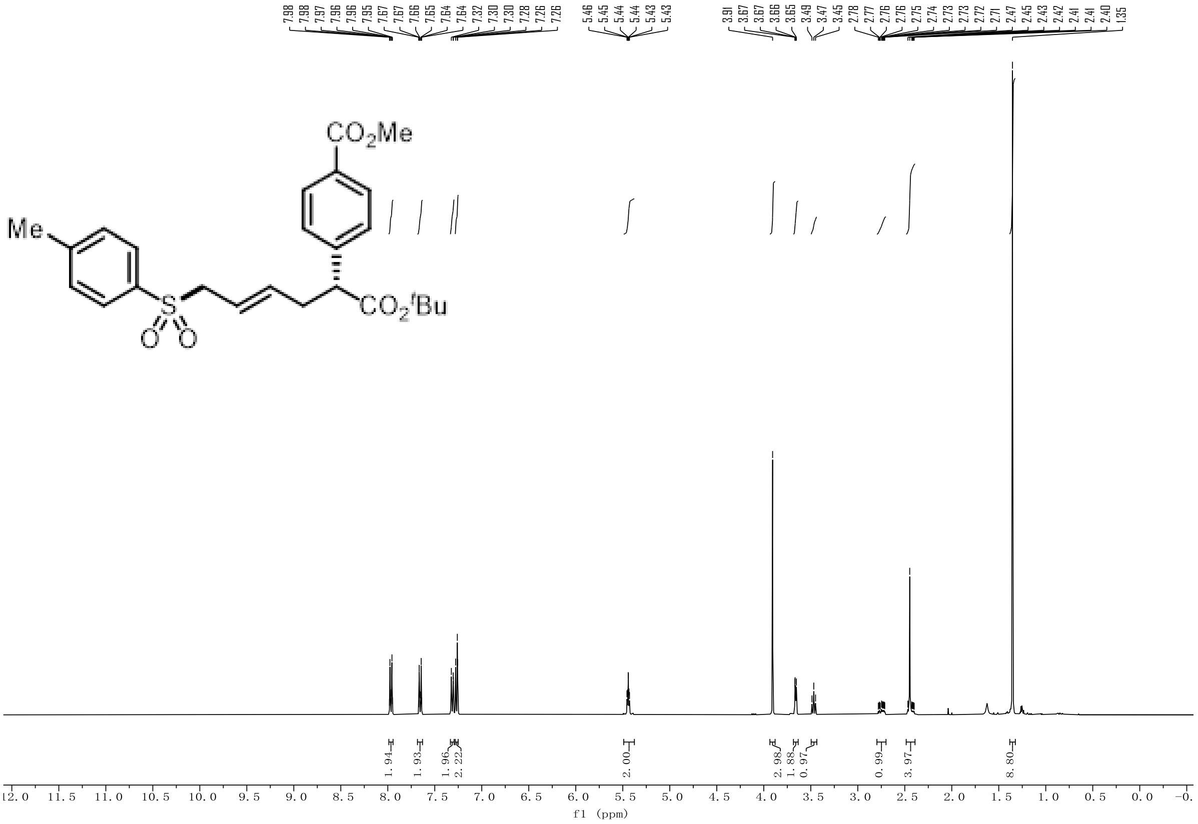

^{1}H NMR (400 MHz, $CDCl_3$, 298 K) spectrum of **34**

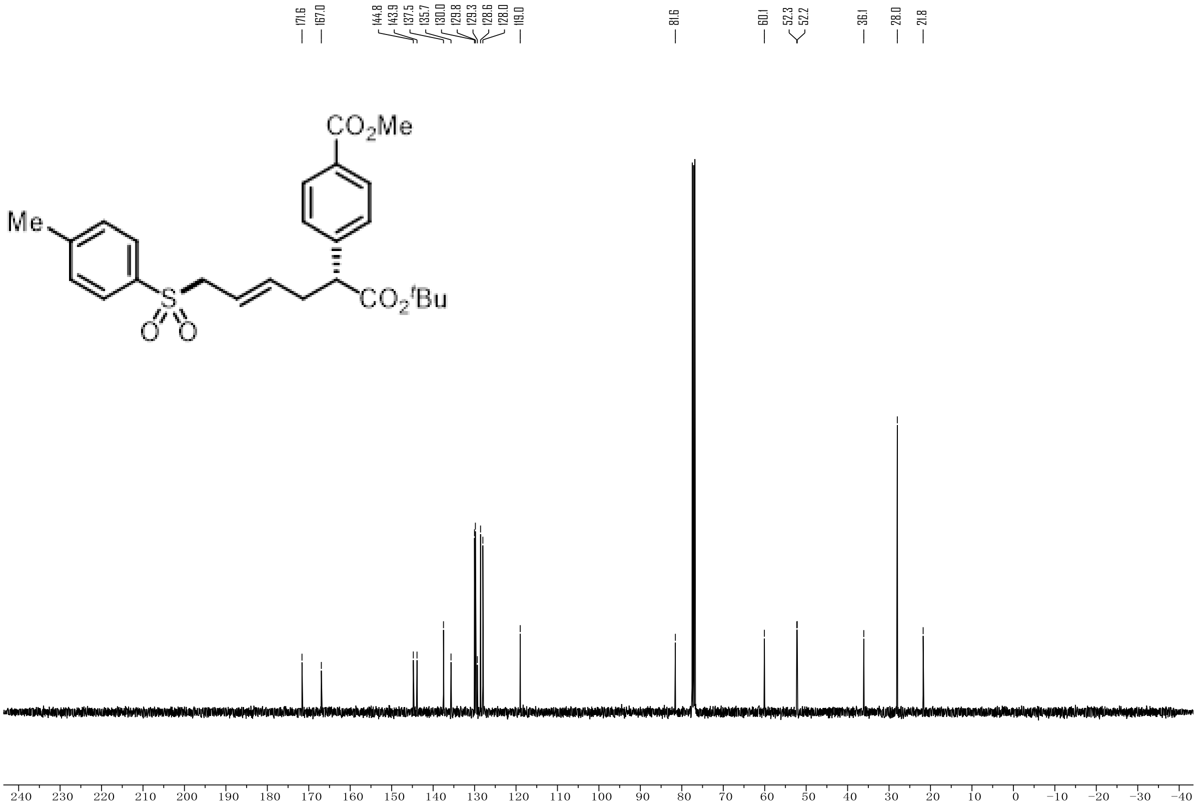

^{13}C NMR (101 MHz, $CDCl_3$, 298 K) spectrum of **34**

Methyl (*R,E*)-4-(1-(*tert*-butoxy)-6-((4-methoxyphenyl)sulfonyl)-1-oxohex-4-en-2-yl)benzoate (35)

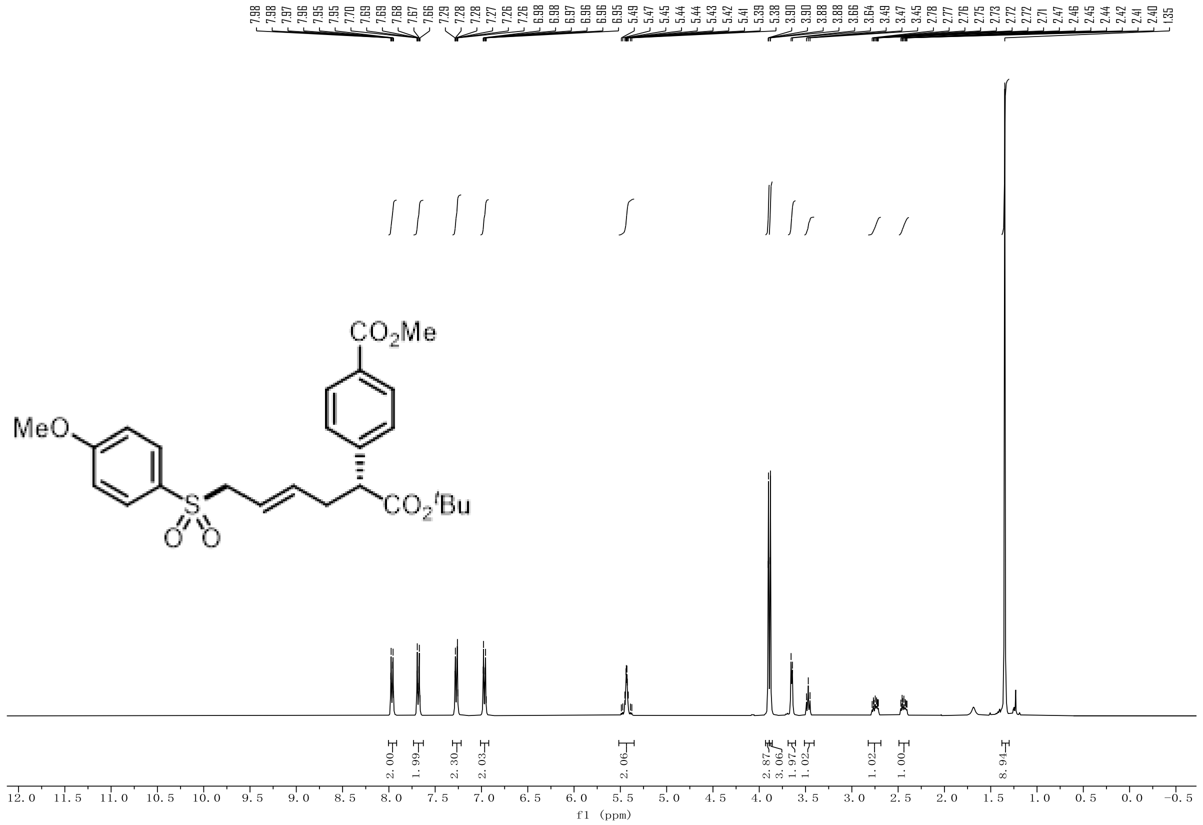

^{1}H NMR (400 MHz, CDCl$_3$, 298 K) spectrum of **35**

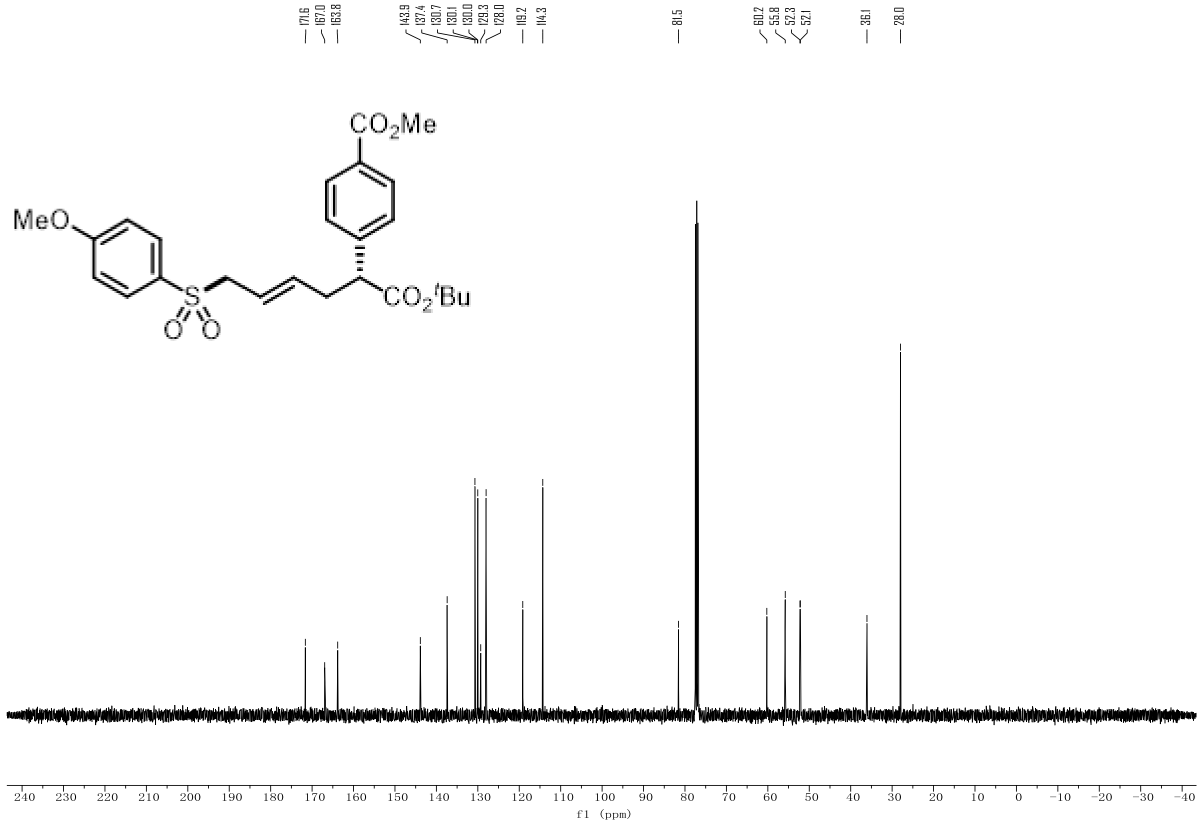

^{13}C NMR (101 MHz, CDCl$_3$, 298 K) spectrum of **35**

Methyl (*R,E*)-4-(1-(*tert*-butoxy)-6-(cyclopropylsulfonyl)-1-oxohex-4-en-2-yl)benzoate (36)

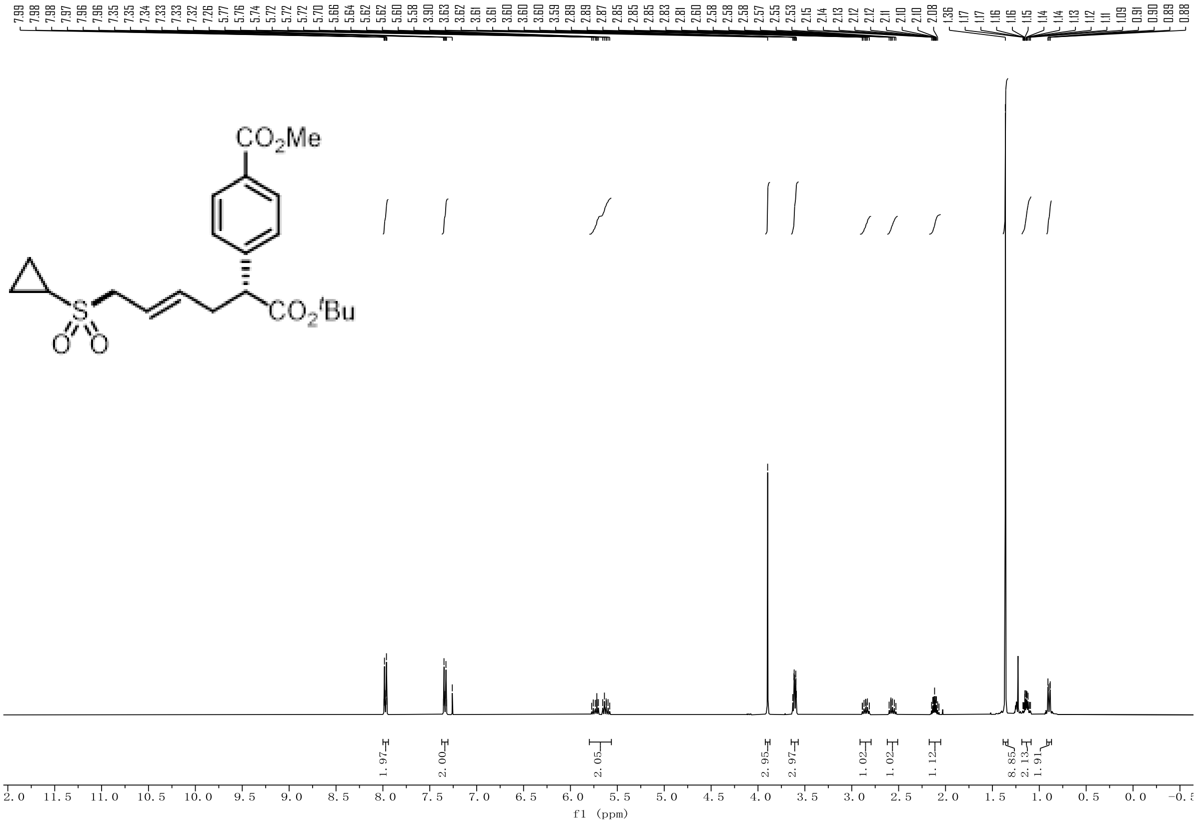

^{1}H NMR (400 MHz, $CDCl_3$, 298 K) spectrum of **36**

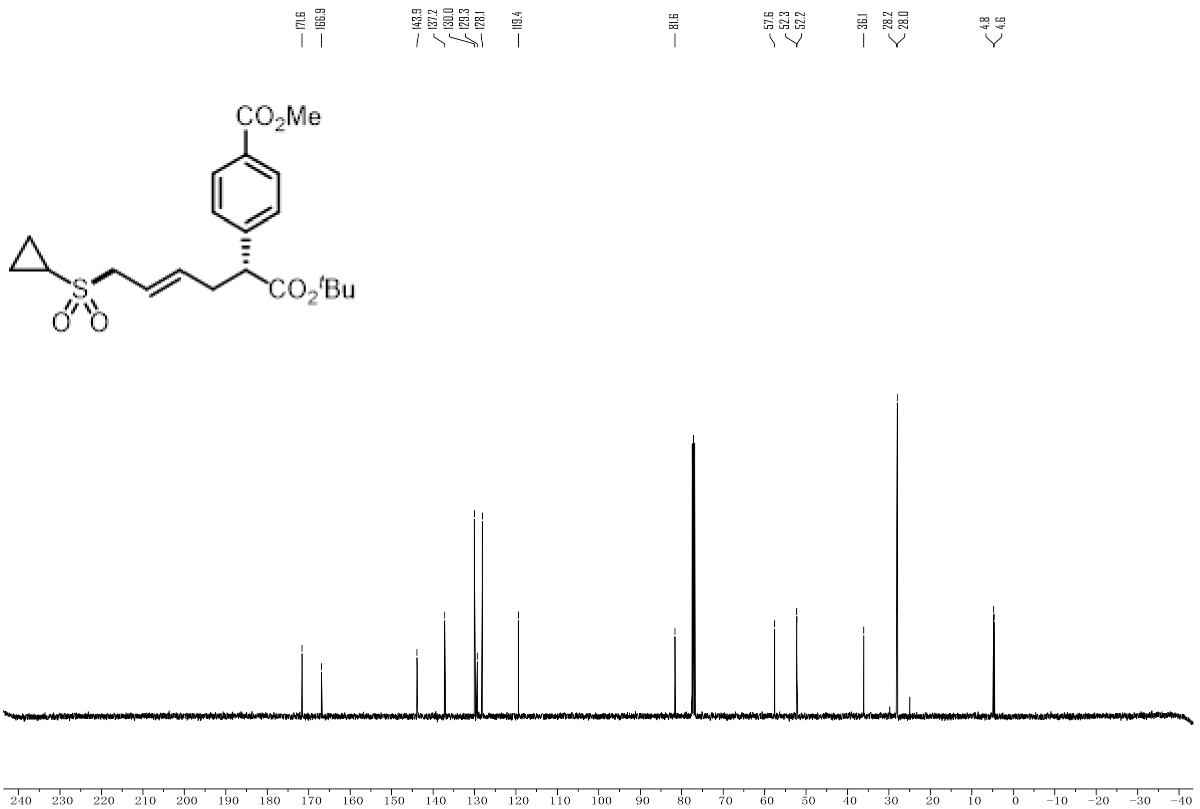

^{13}C NMR (101 MHz, $CDCl_3$, 298 K) spectrum of **36**

tert-Butyl (_R,E_)-7,7-dimethyl-2-(4-(trifluoromethyl)phenyl)oct-4-enoate (37)

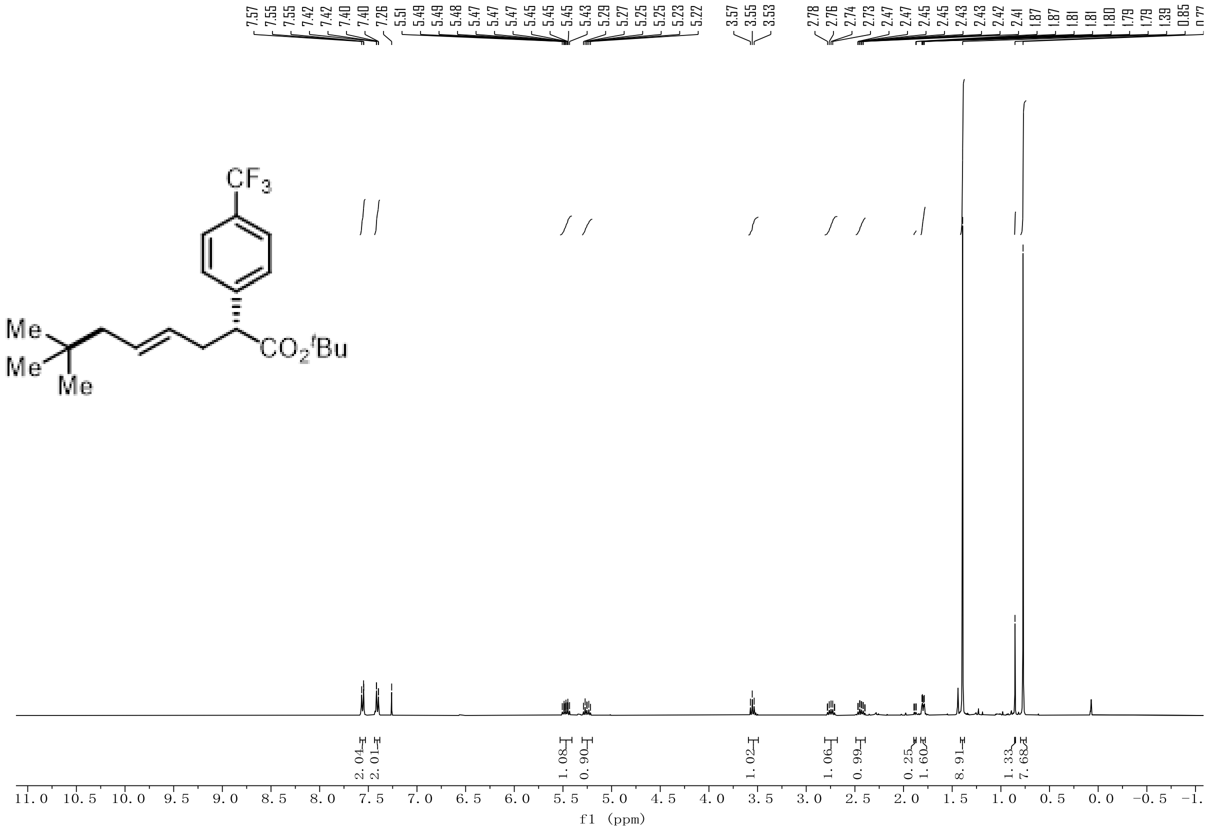

^{1}H NMR (400 MHz, $CDCl_3$, 298 K) spectrum of **37**

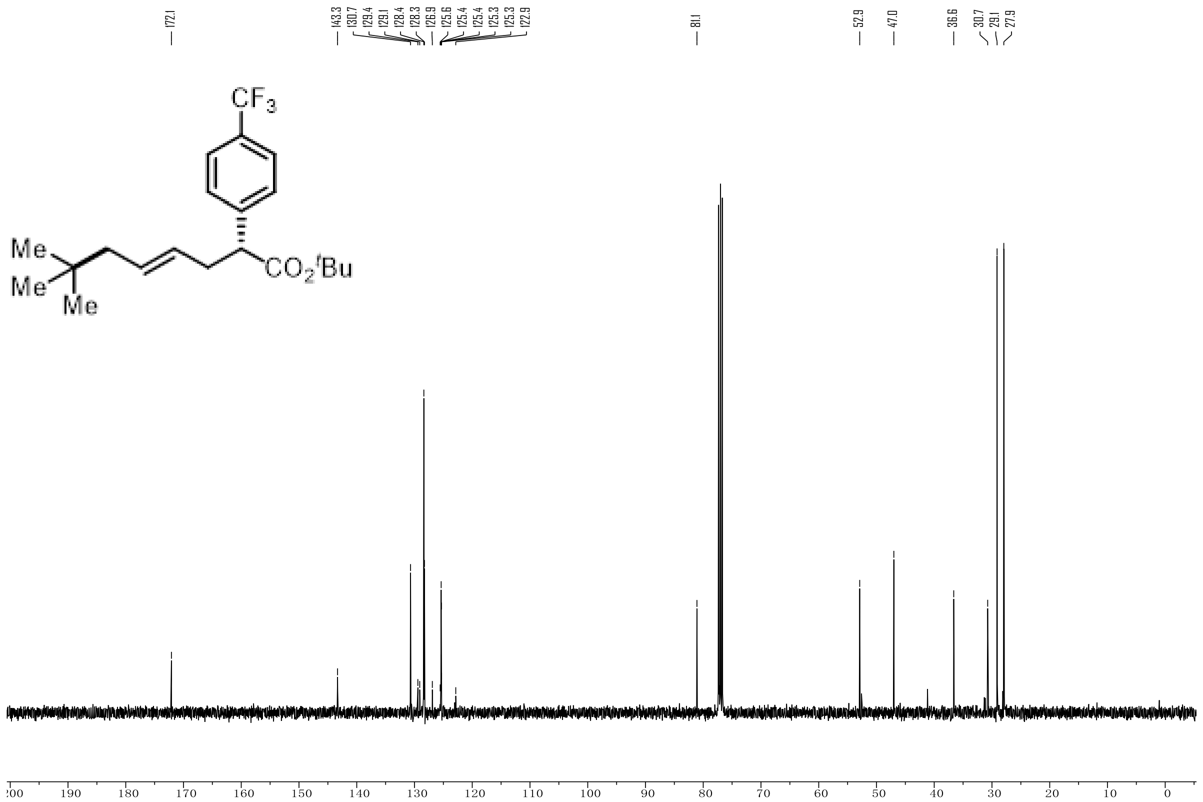

^{13}C NMR (101 MHz, $CDCl_3$, 298 K) spectrum of **37**

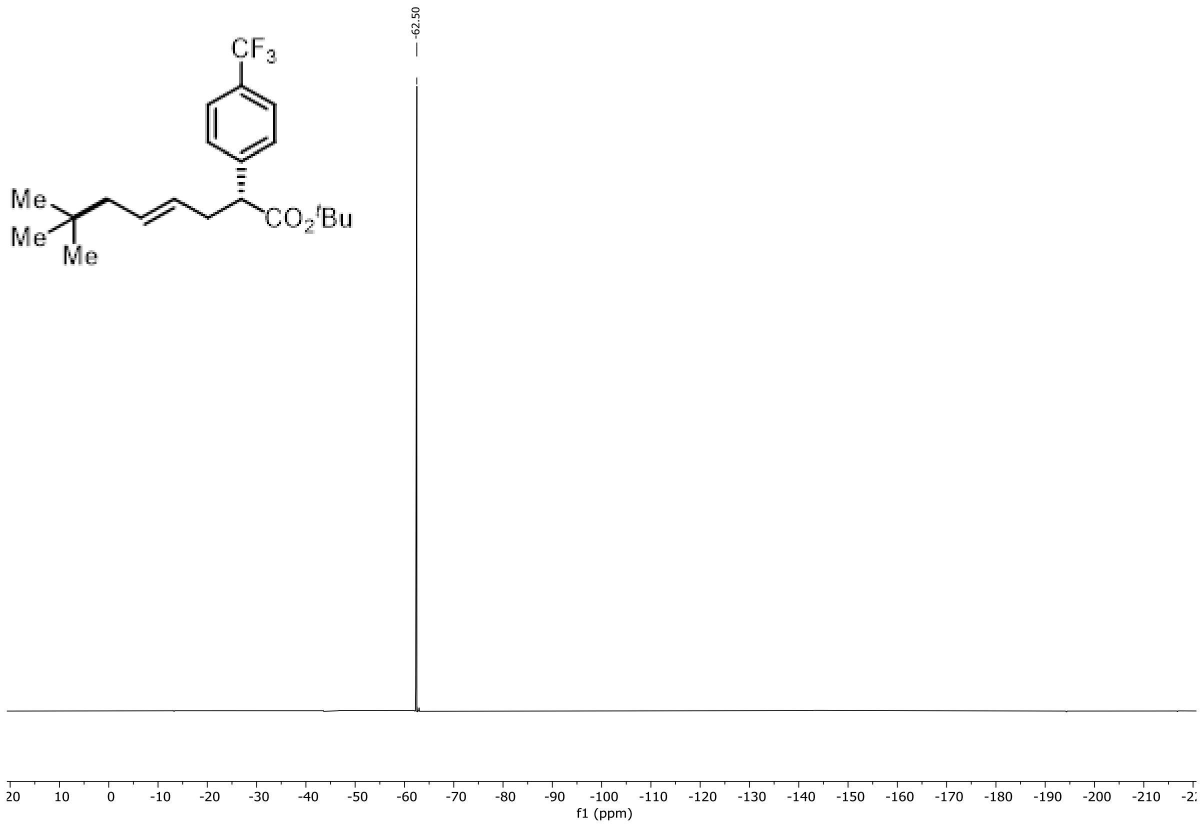

^{19}F NMR (377 MHz, $CDCl_3$, 298 K) spectrum of **37**

***tert*-Butyl (*R,E*)-2-(4-cyanophenyl)-7,7-dimethyloct-4-enoate (38)**

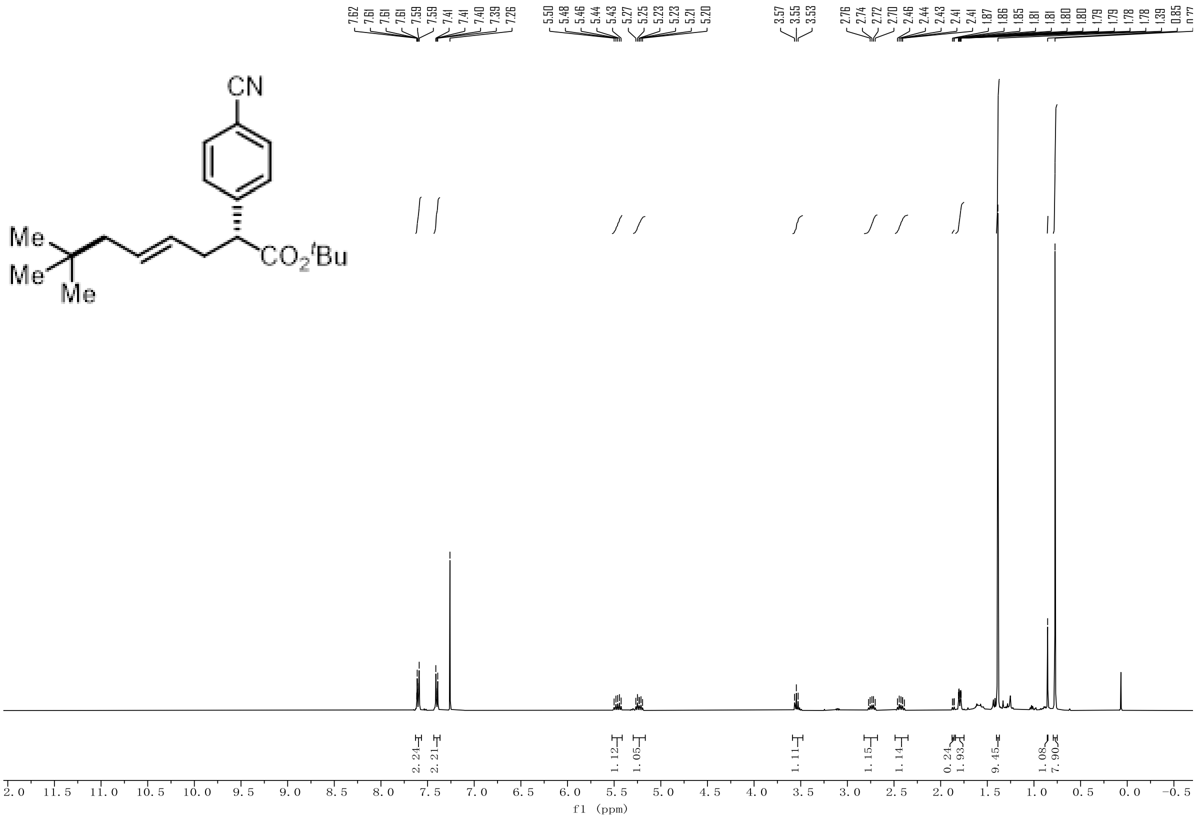

^{1}H NMR (400 MHz, $CDCl_3$, 298 K) spectrum of **38**

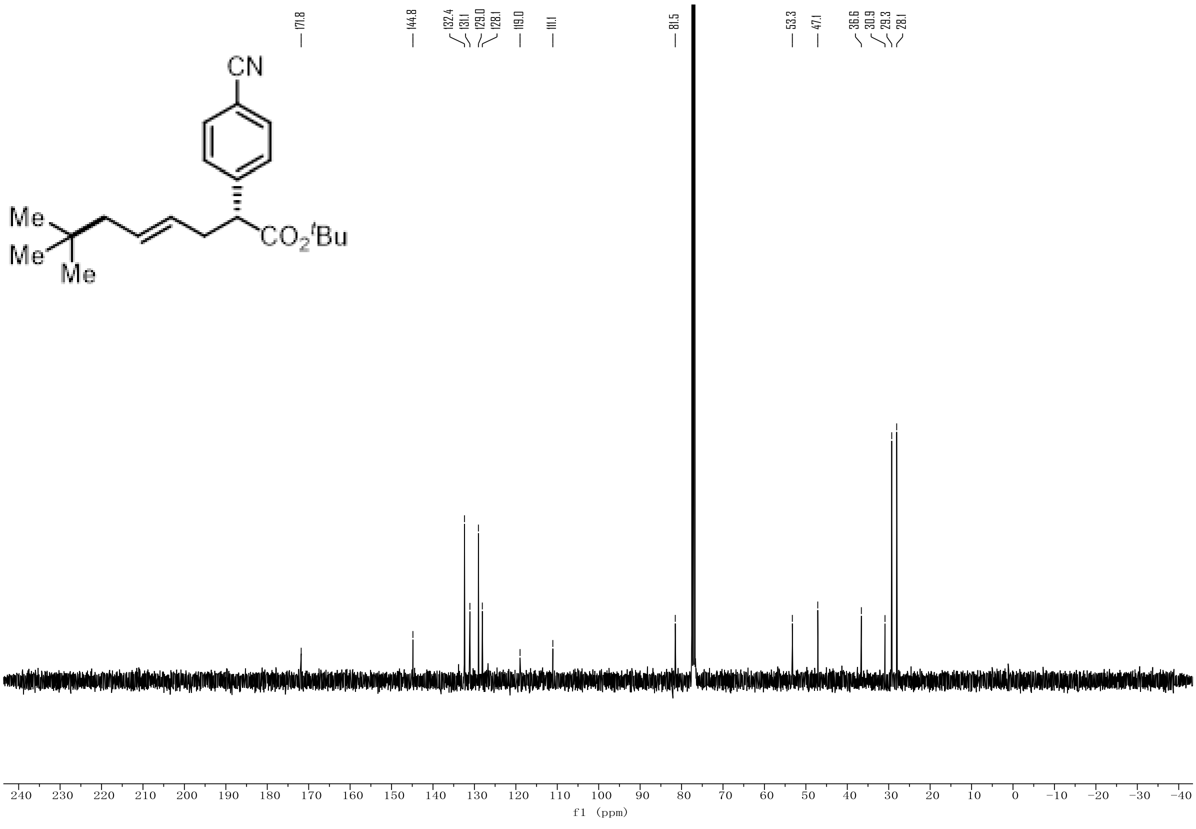

^{13}C NMR (101 MHz, $CDCl_3$, 298 K) spectrum of **38**

***tert*-Butyl (*R,E*)-7,7-dimethyl-2-(4-(methylsulfonyl)phenyl)oct-4-enoate (39)**

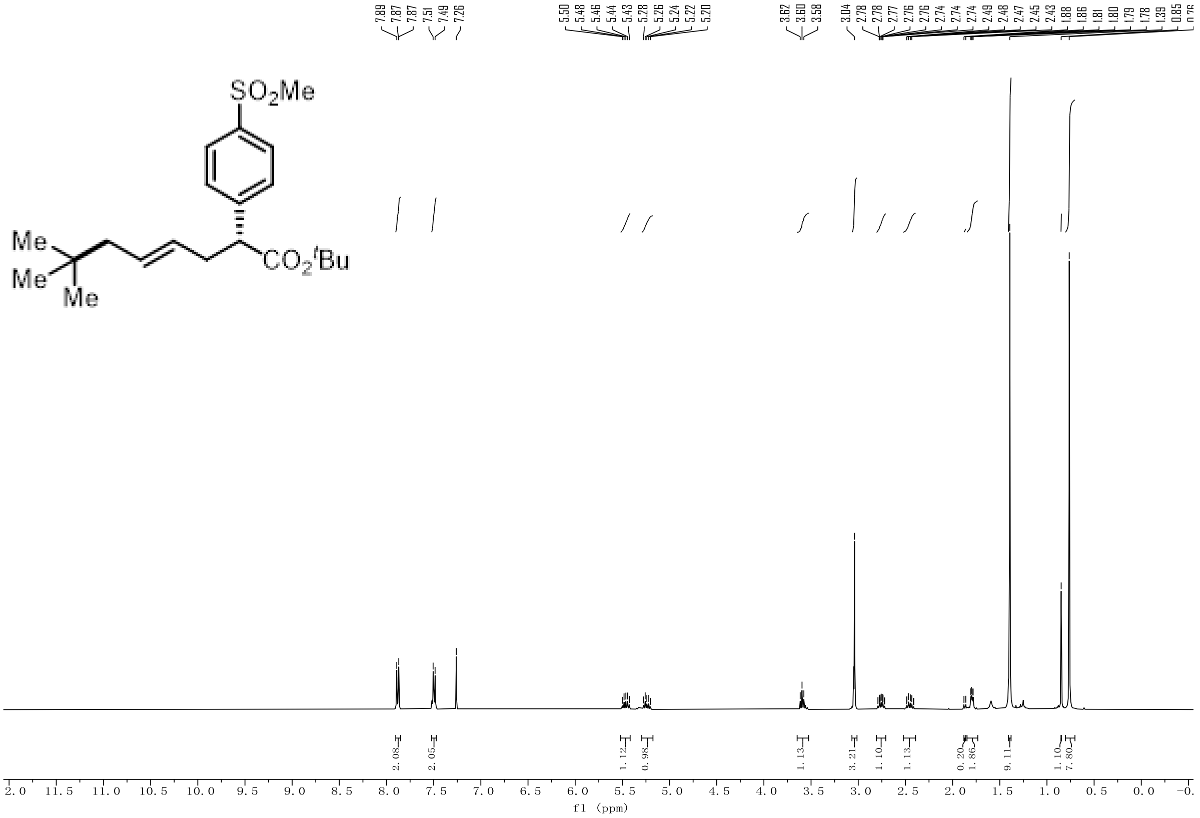

^{1}H NMR (400 MHz, $CDCl_3$, 298 K) spectrum of **39**

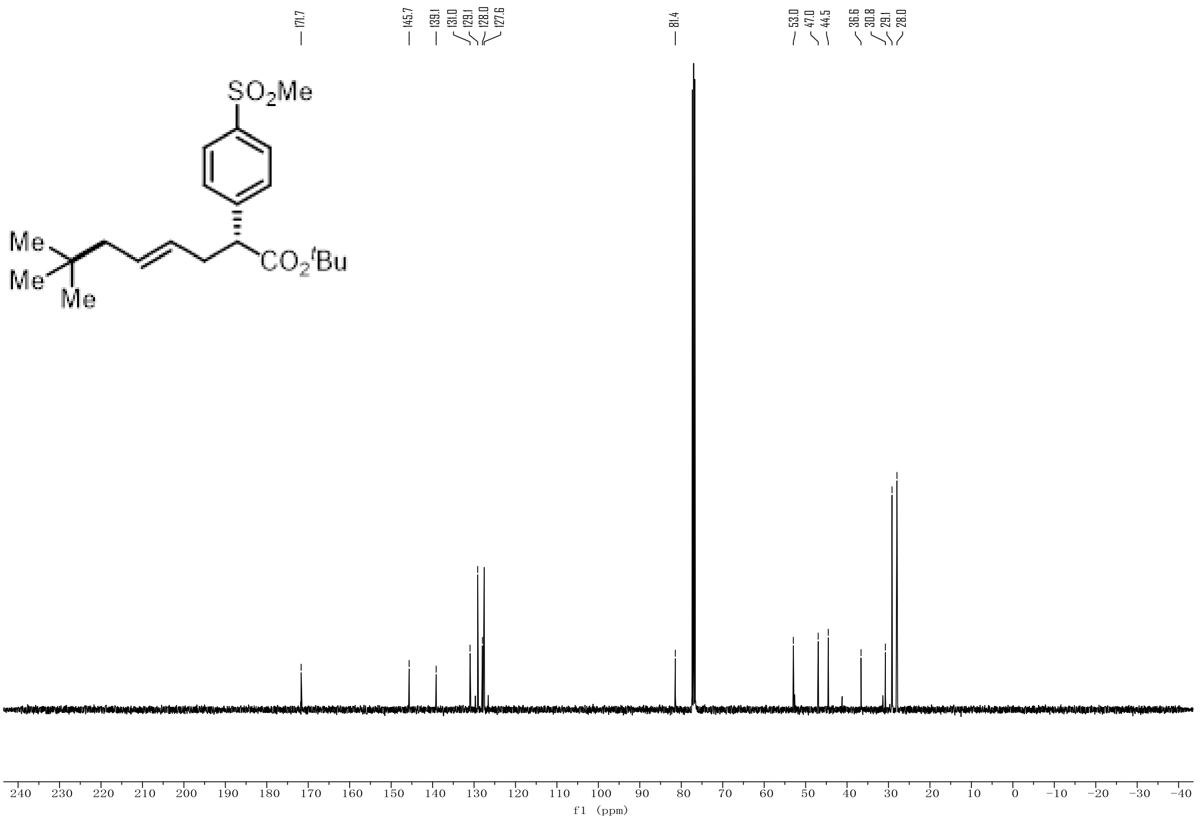

^{13}C NMR (101 MHz, $CDCl_3$, 298 K) spectrum of **39**

***tert*-Butyl (*R,E*)-2-(4-formylphenyl)-7,7-dimethyloct-4-enoate (40)**

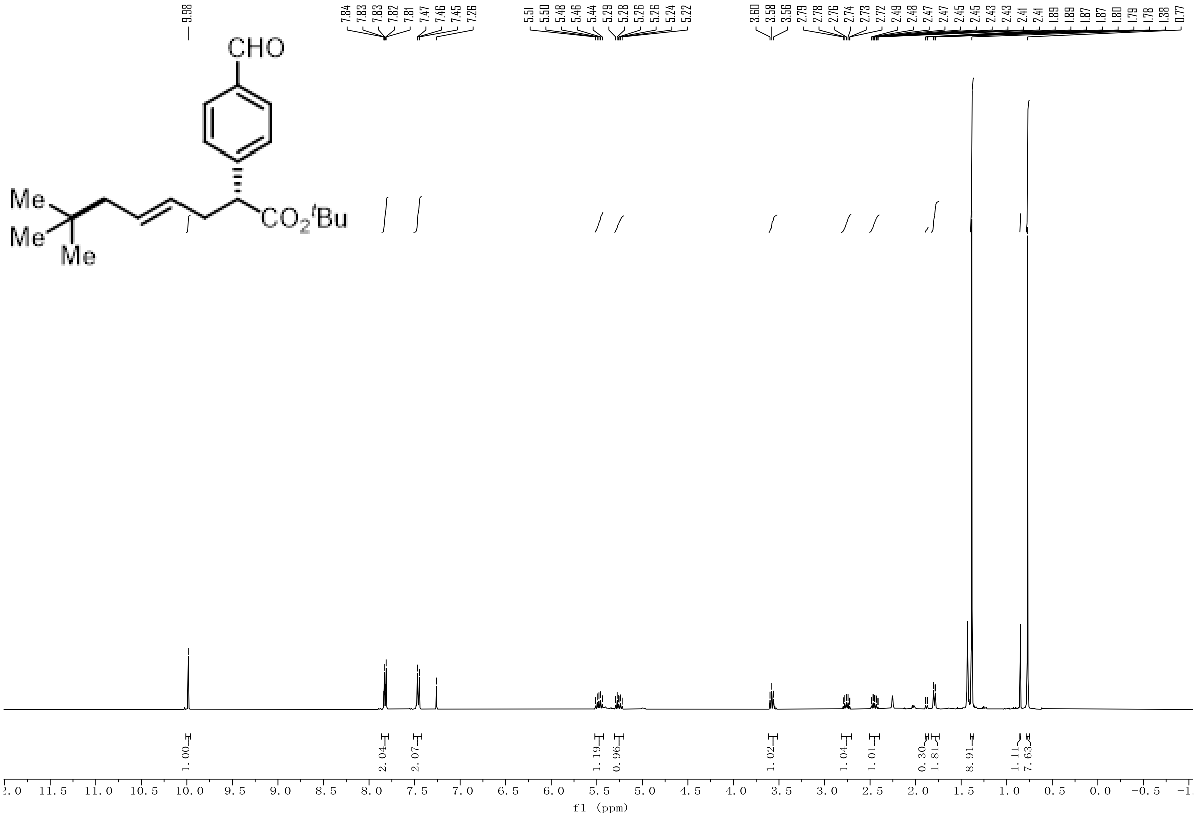

^{1}H NMR (400 MHz, $CDCl_3$, 298 K) spectrum of **40**

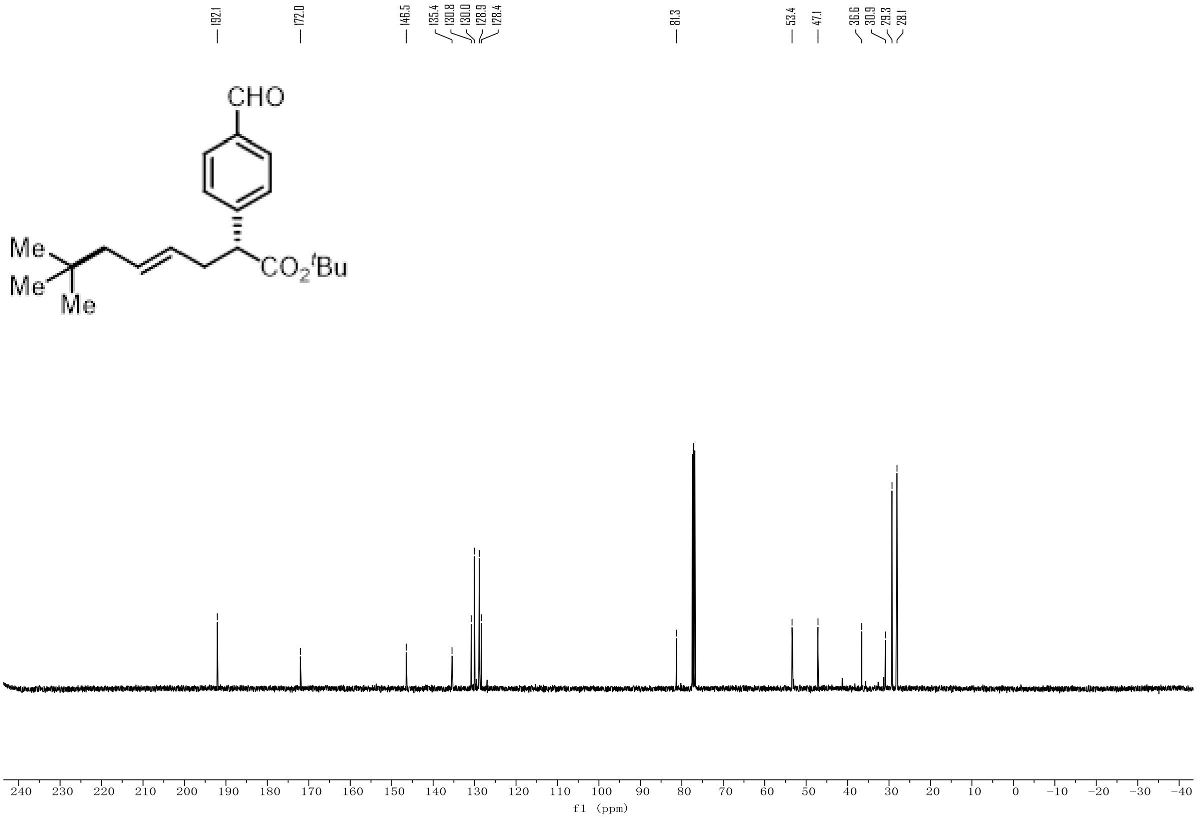

^{13}C NMR (101 MHz, $CDCl_3$, 298 K) spectrum of **40**

tert-Butyl (_R,E_)-2-(4-acetylphenyl)-7,7-dimethyloct-4-enoate (41)

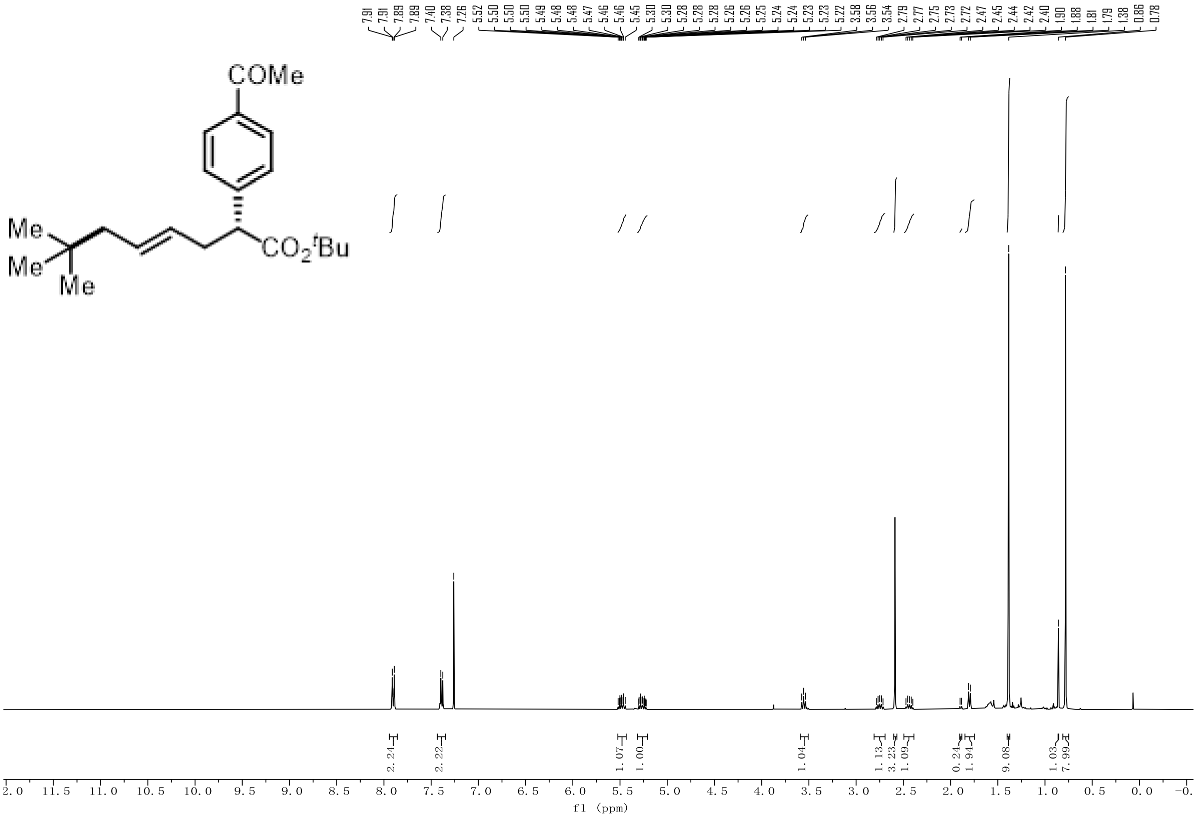

^{1}H NMR (400 MHz, $CDCl_3$, 298 K) spectrum of **41**

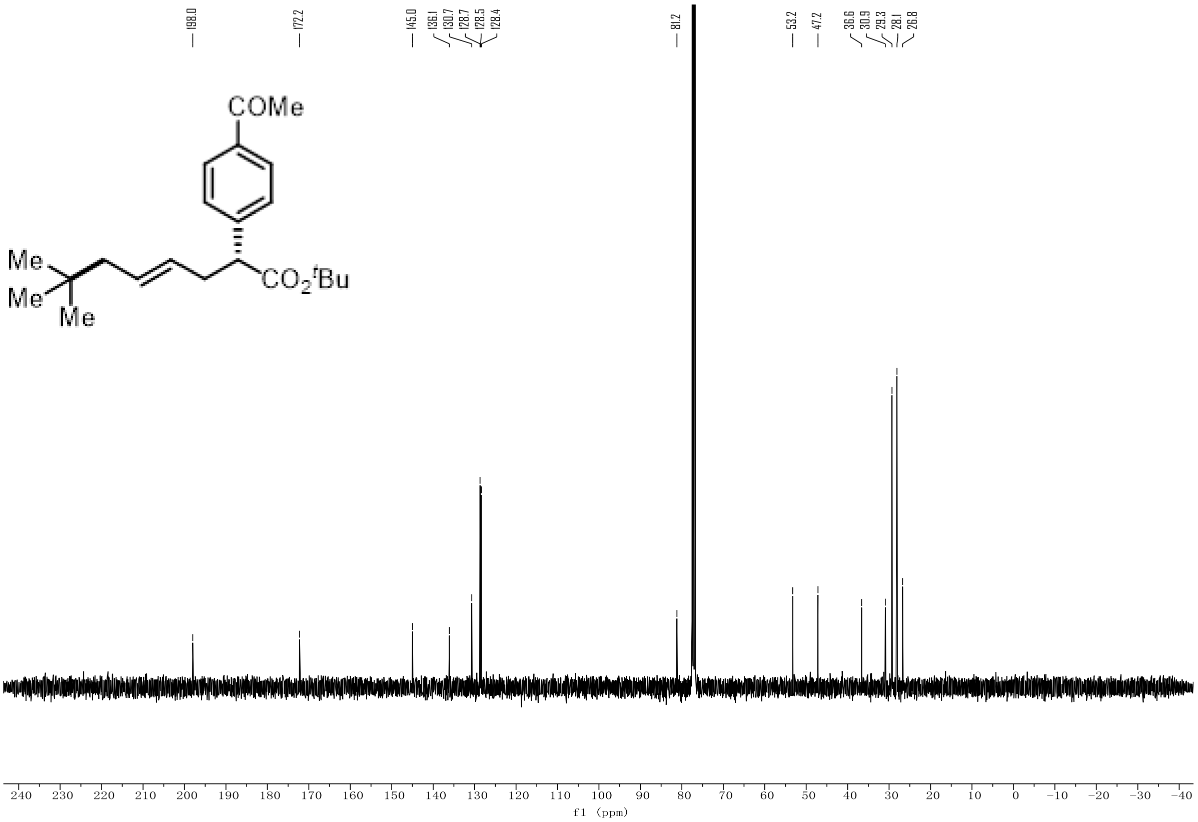

^{13}C NMR (101 MHz, $CDCl_3$, 298 K) spectrum of **41**

***tert*-Butyl (*R,E*)-2-(4-benzoylphenyl)-7,7-dimethyloct-4-enoate (42)**

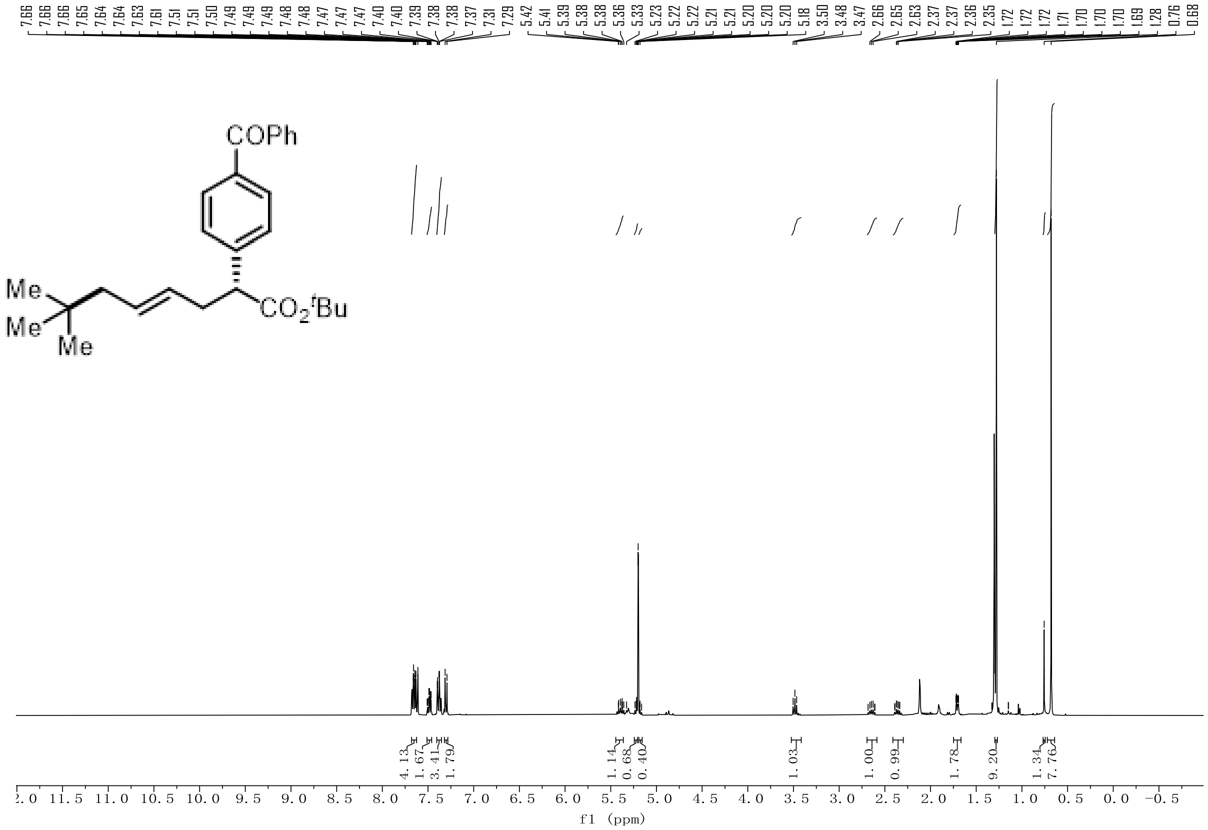

^{1}H NMR (400 MHz, CDCl$_3$, 298 K) spectrum of **42**

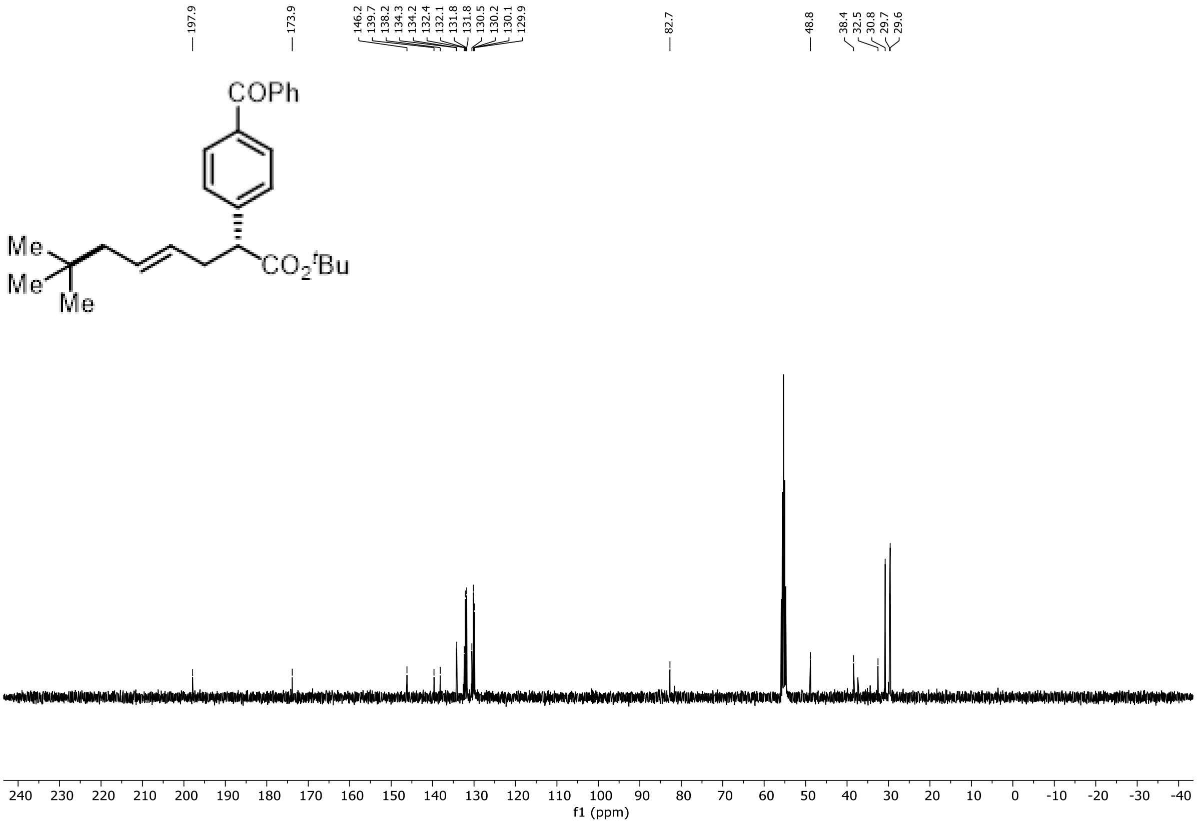

^{13}C NMR (101 MHz, CDCl$_3$, 298 K) spectrum of **42**

tert-Butyl (R,E)-2-(3-chlorophenyl)-7,7-dimethyloct-4-enoate (43)

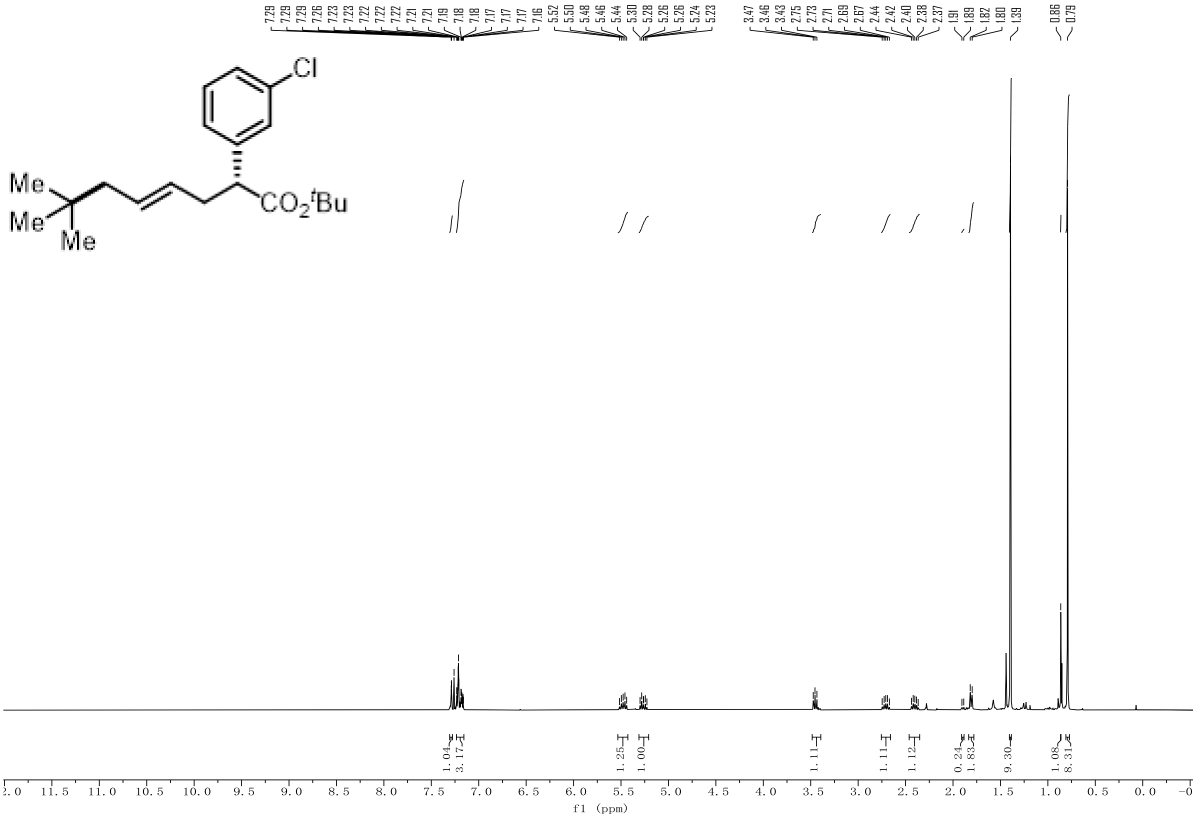

^{1}H NMR (400 MHz, $CDCl_3$, 298 K) spectrum of **43**

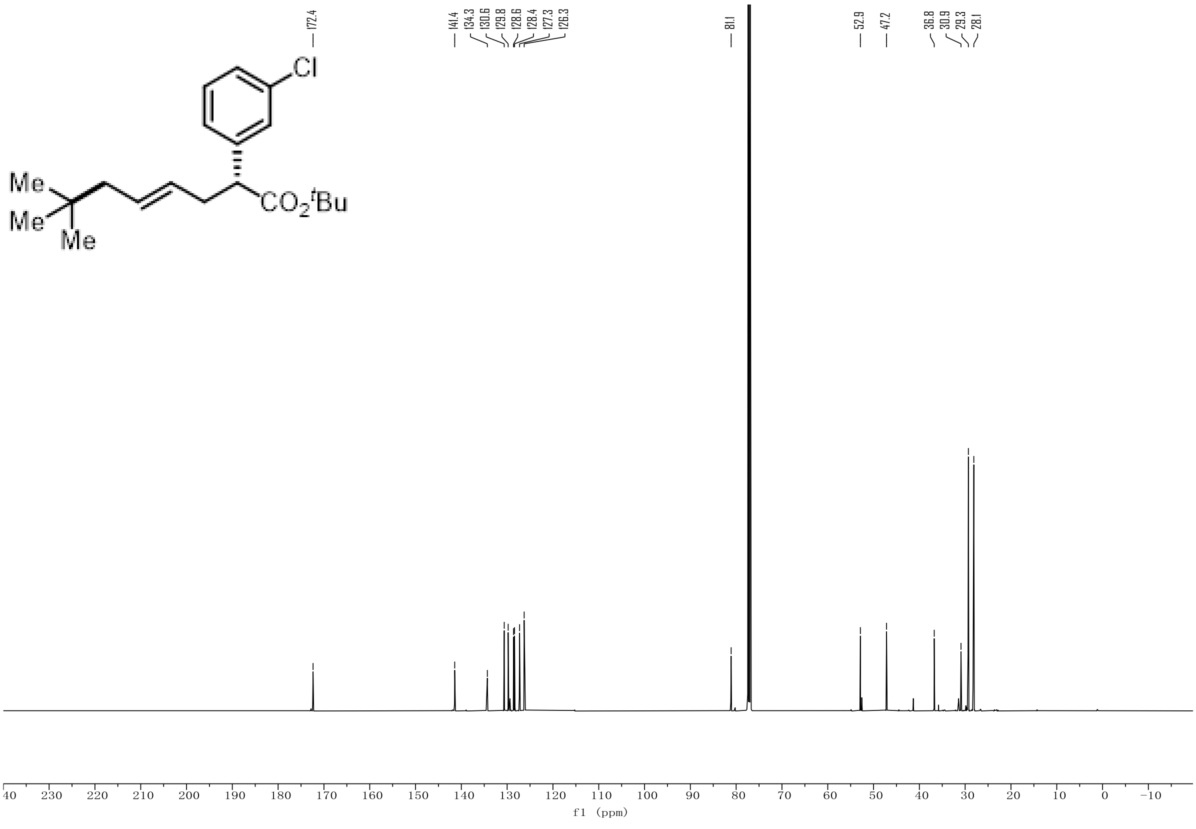

^{13}C NMR (126 MHz, $CDCl_3$, 298 K) spectrum of **43**

tert-Butyl (_R,E_)-7,7-dimethyl-2-(1-oxo-1,3-dihydroisobenzofuran-5-yl)oct-4-enoate (44)

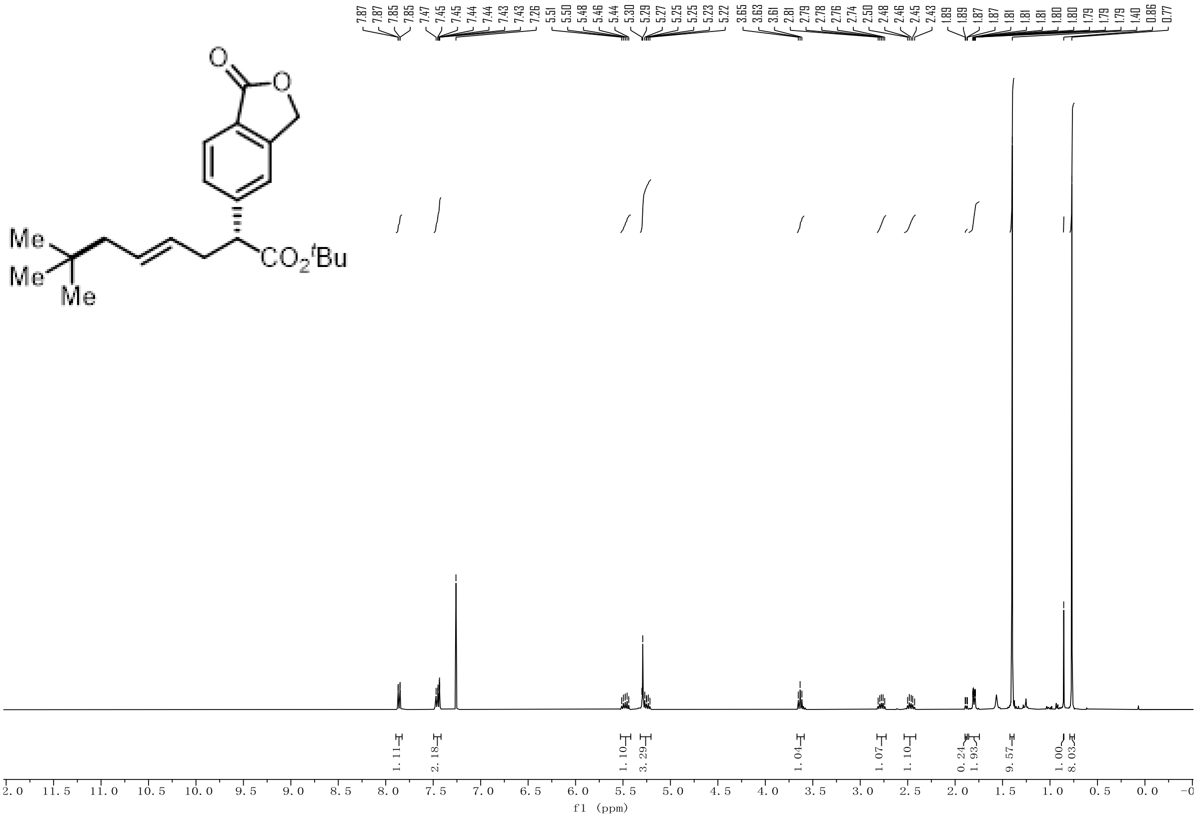

^{1}H NMR (400 MHz, $CDCl_3$, 298 K) spectrum of **44**

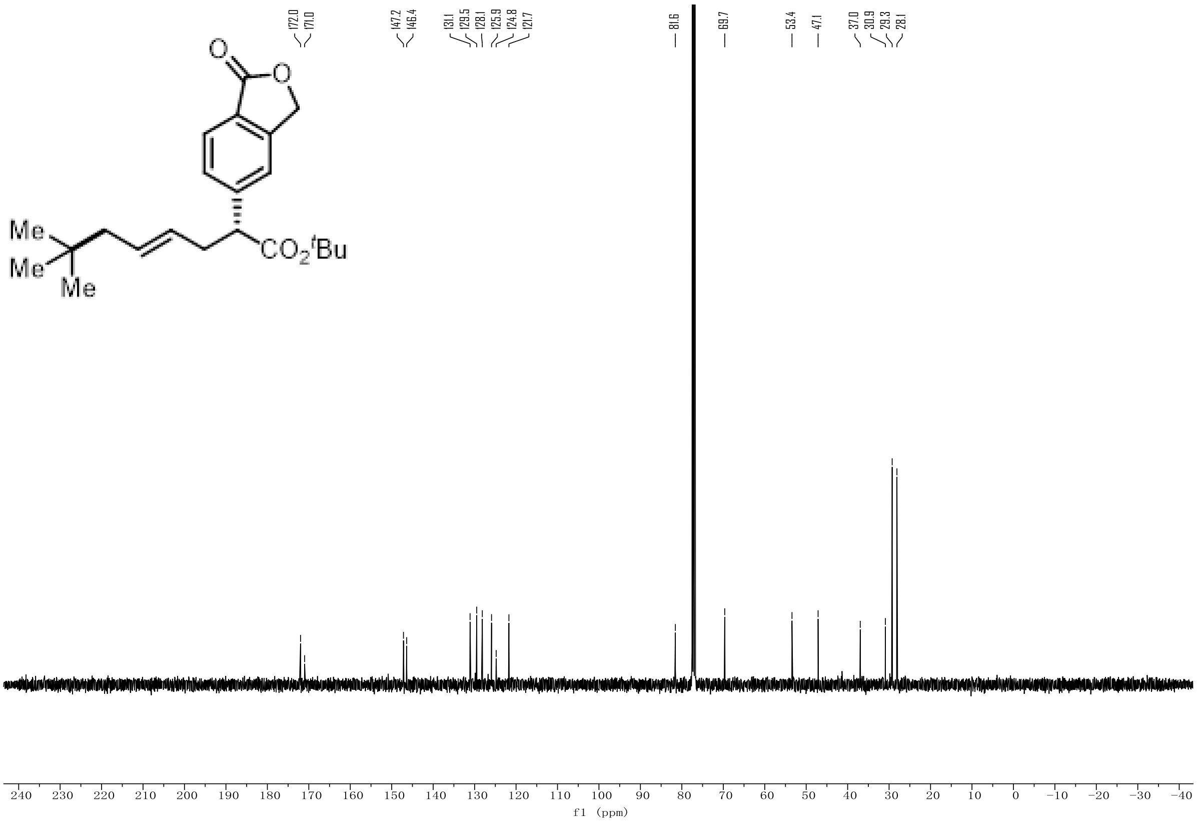

^{13}C NMR (101 MHz, $CDCl_3$, 298 K) spectrum of **44**

***tert*-Butyl (*R,E*)-2-(2-chloropyridin-4-yl)-7,7-dimethyloct-4-enoate (45)**

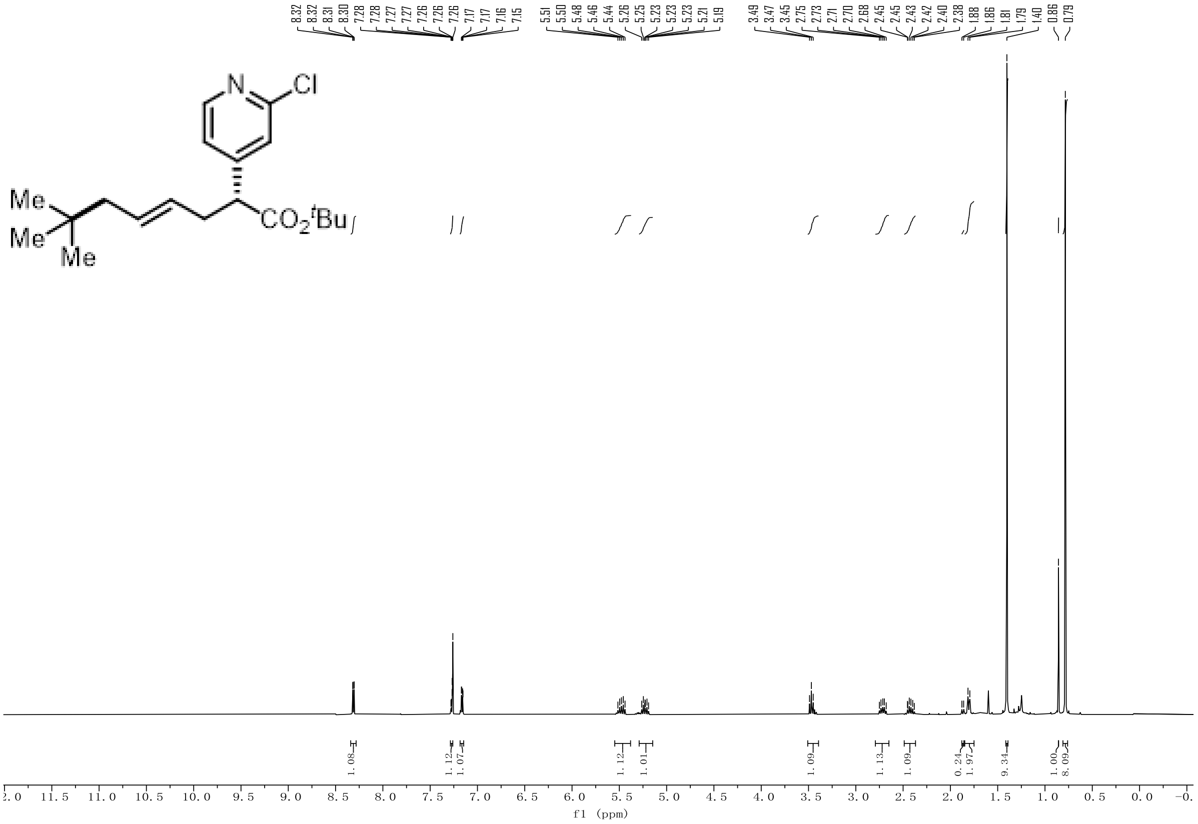

^{1}H NMR (400 MHz, $CDCl_3$, 298 K) spectrum of **45**

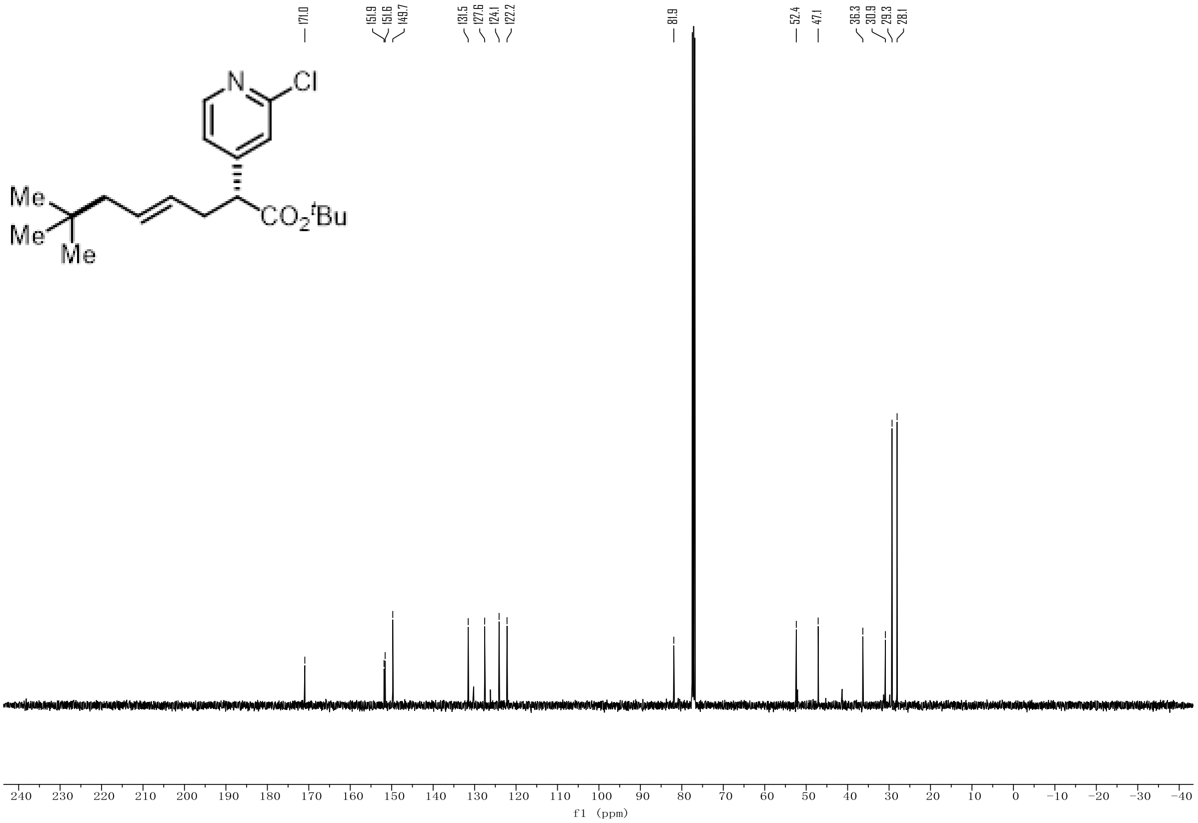

^{13}C NMR (101 MHz, $CDCl_3$, 298 K) spectrum of **45**

Methyl (*R*,*E*)-4-(1-((2,4-dimethylpentan-3-yl)oxy)-7,7-dimethyl-1-oxooct-4-en-2-yl)benzoate (46)

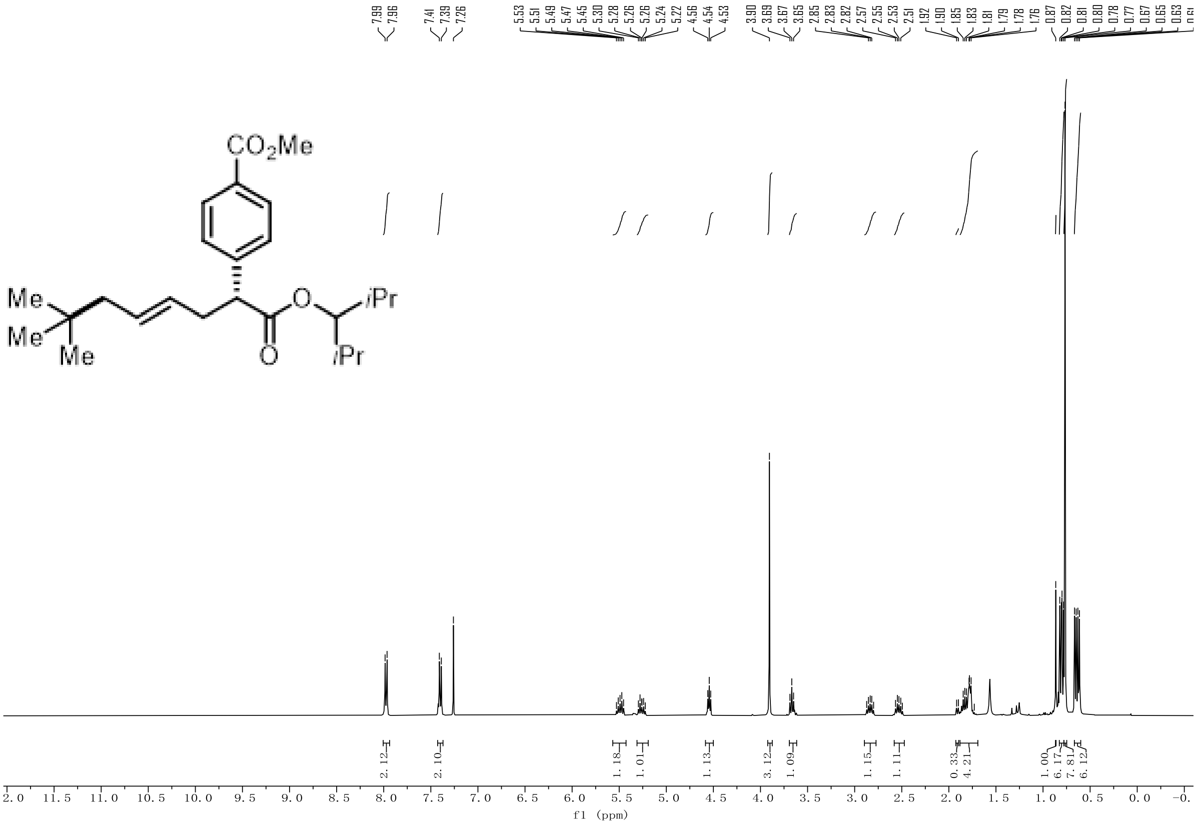

^{1}H NMR (400 MHz, $CDCl_3$, 298 K) spectrum of **46**

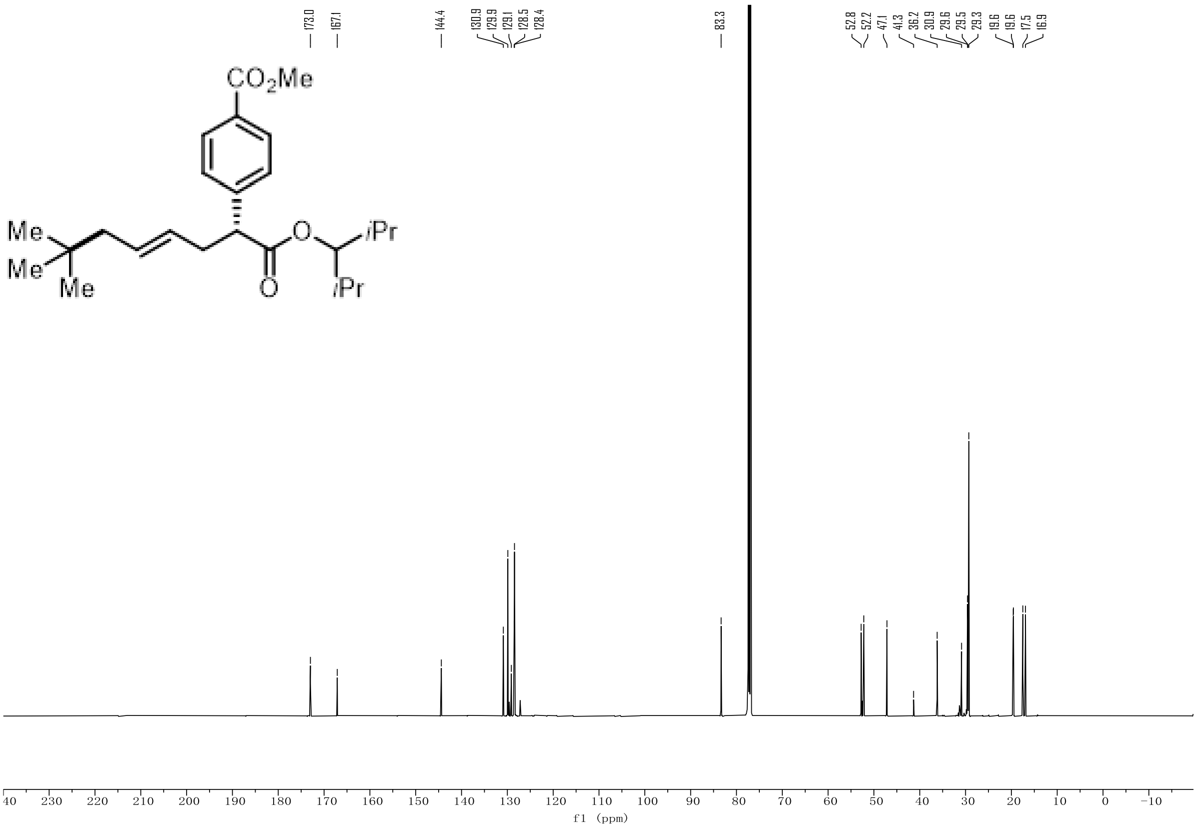

^{13}C NMR (126 MHz, $CDCl_3$, 298 K) spectrum of **46**

Methyl (*R*,*E*)-4-(7,7-dimethyl-1-((2-methyl-4-phenylbutan-2-yl)oxy)-1-oxooct-4-en-2-yl)benzoate (47)

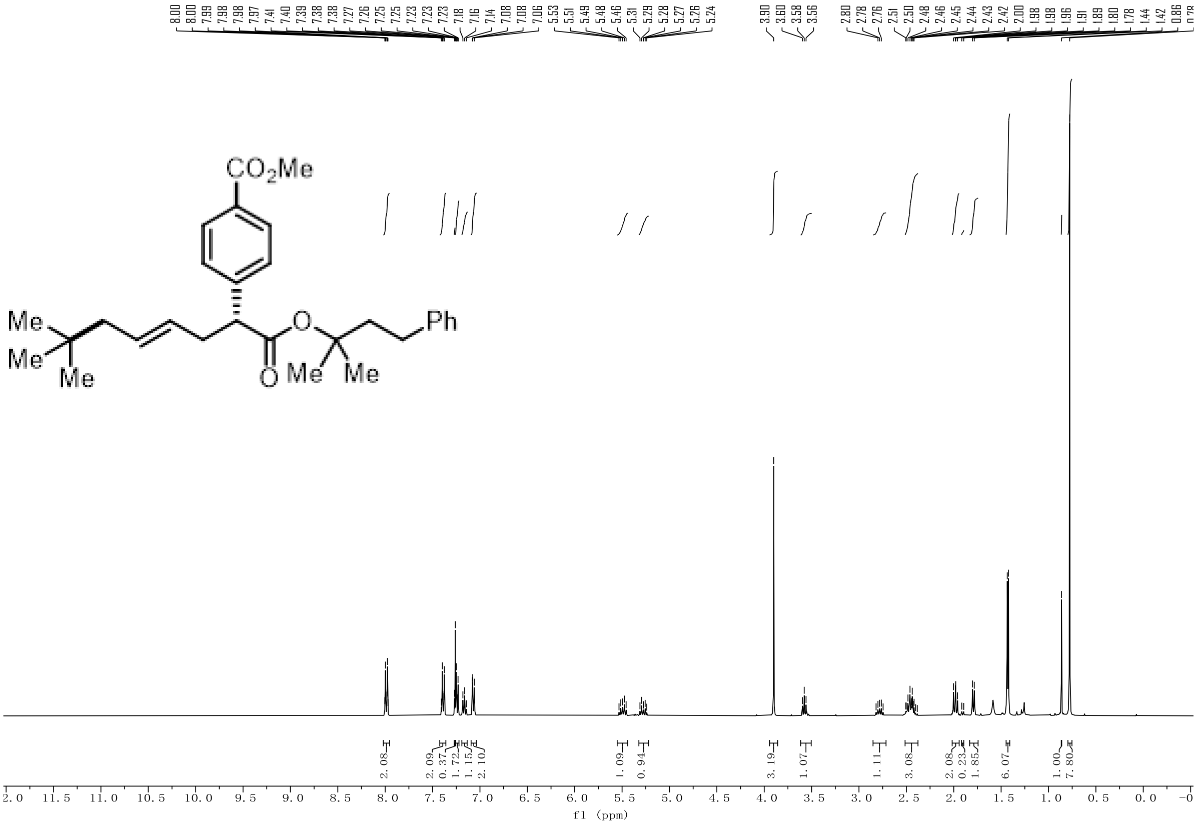

^{1}H NMR (400 MHz, $CDCl_3$, 298 K) spectrum of **47**

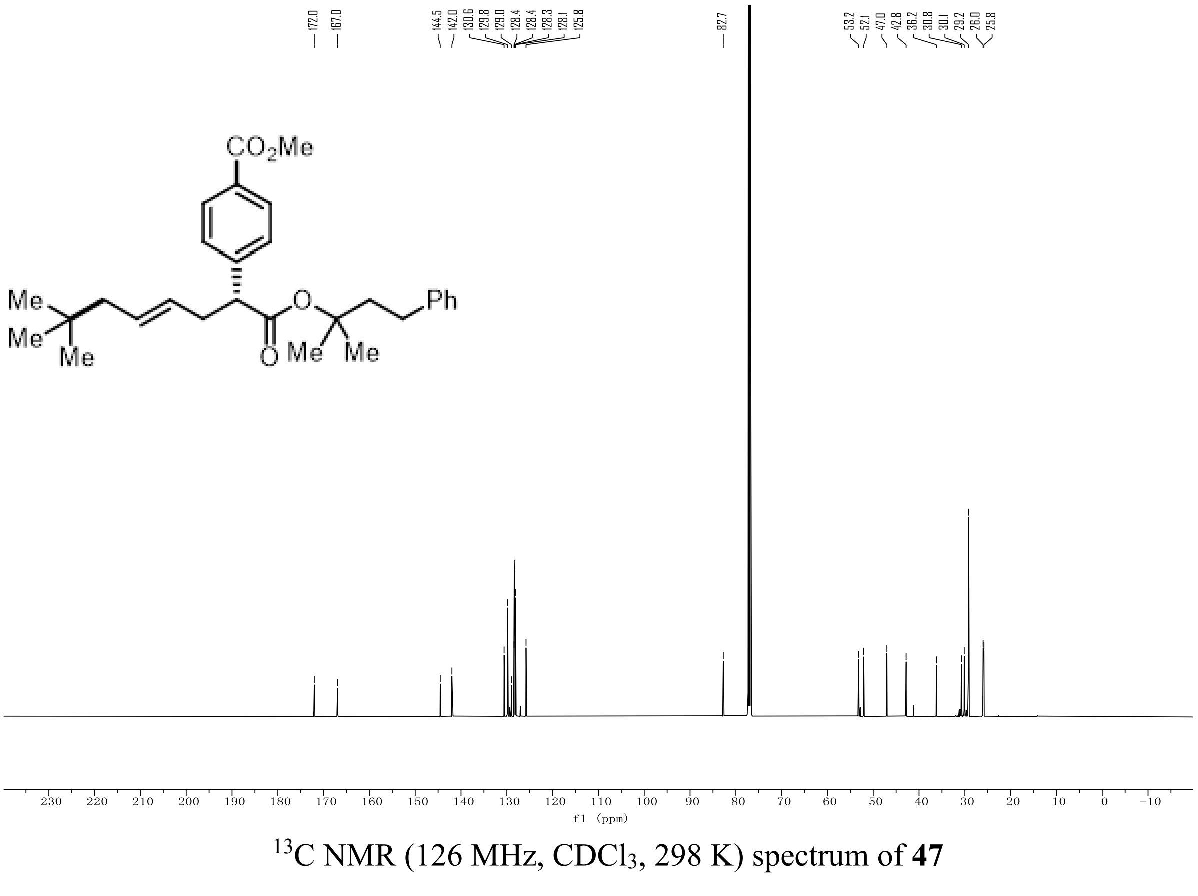

^{13}C NMR (126 MHz, $CDCl_3$, 298 K) spectrum of **47**

Methyl 4-((*R*,*E*)-1-(((3S,5S,7S)-adamantan-1-yl)oxy)-7,7-dimethyl-1-oxooct-4-en-2-yl)benzoate (48)

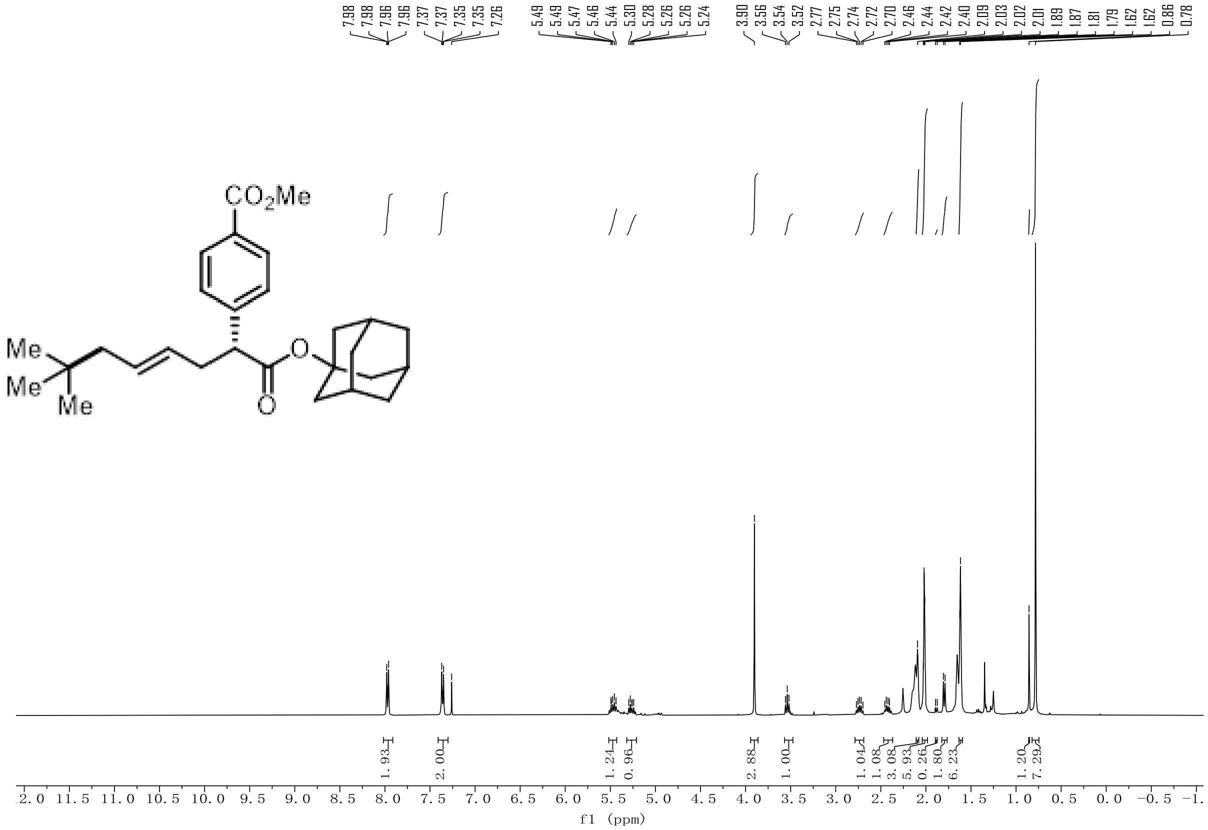

^{1}H NMR (400 MHz, $CDCl_3$, 298 K) spectrum of **48**

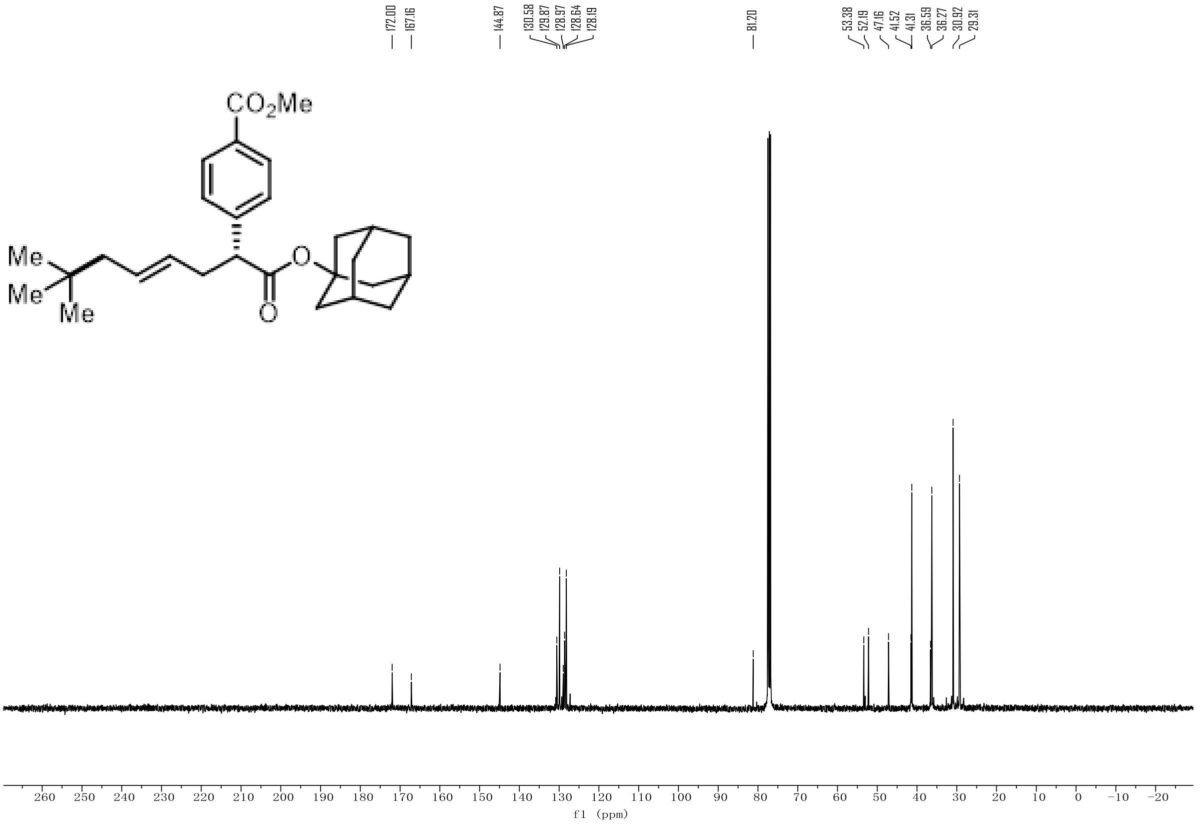

^{13}C NMR (101 MHz, $CDCl_3$, 298 K) spectrum of **48**

Methyl (*R,E*)-4-(7,7-dimethyl-1-((1-methylcyclohexyl)oxy)-1-oxooct-4-en-2-yl)benzoate (49)

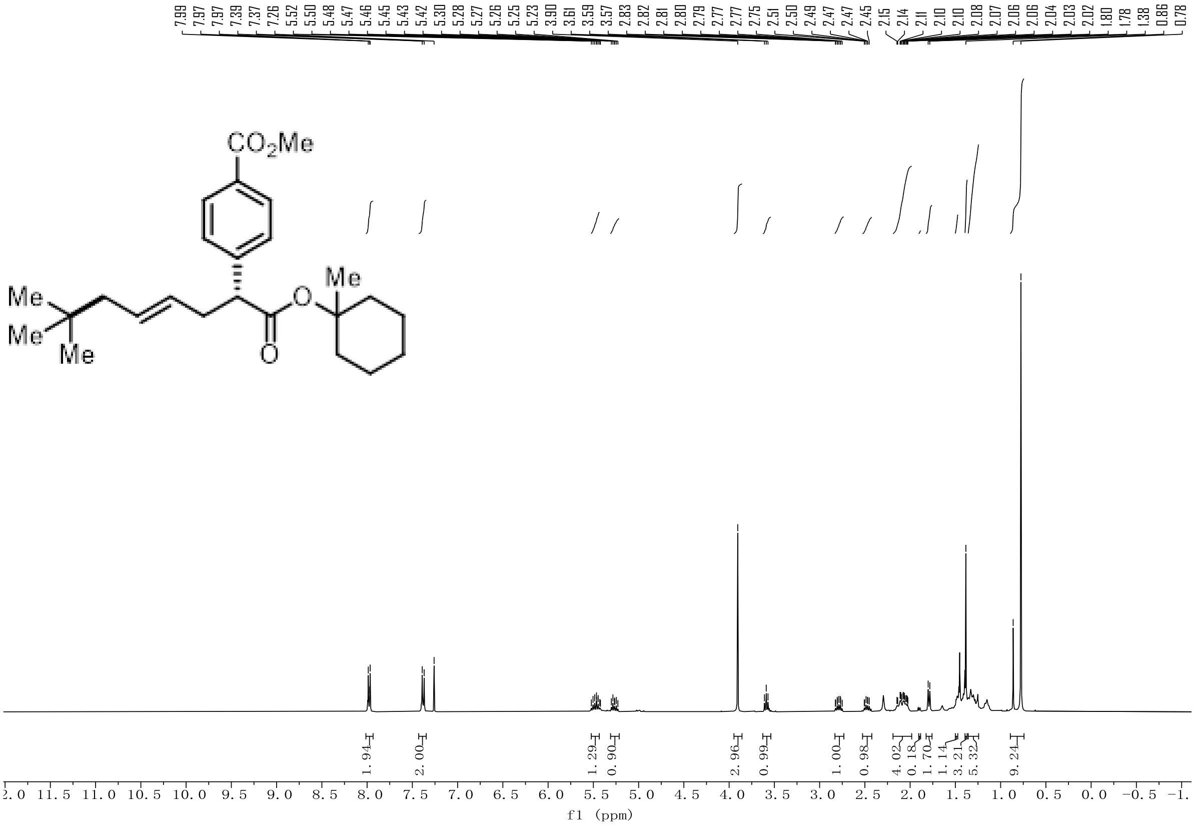

^{1}H NMR (400 MHz, $CDCl_3$, 298 K) spectrum of **49**

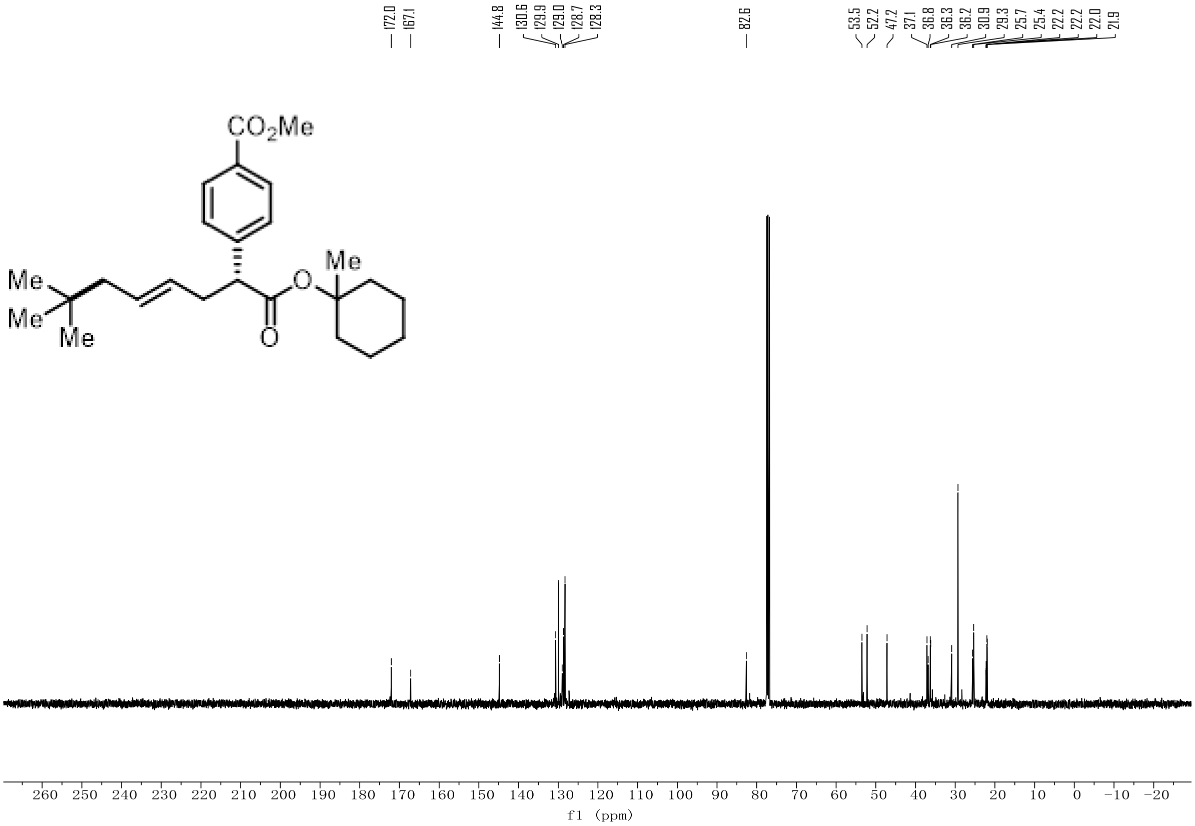

^{13}C NMR (101 MHz, $CDCl_3$, 298 K) spectrum of **49**

tert-Butyl (R,E)-2-(4-cyanophenyl)-7,7-dimethyloct-4-enoate (50)

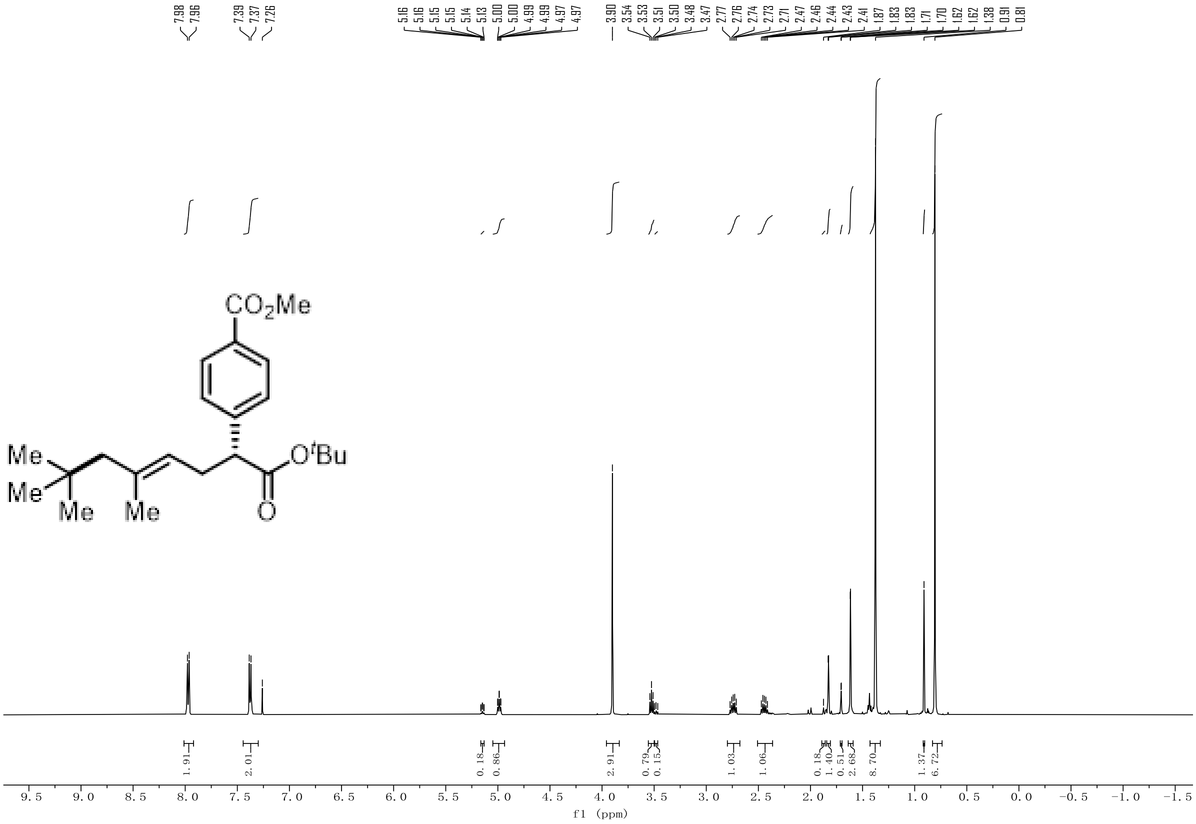

^{1}H NMR (500 MHz, CDCl$_3$, 298 K) spectrum of **50**

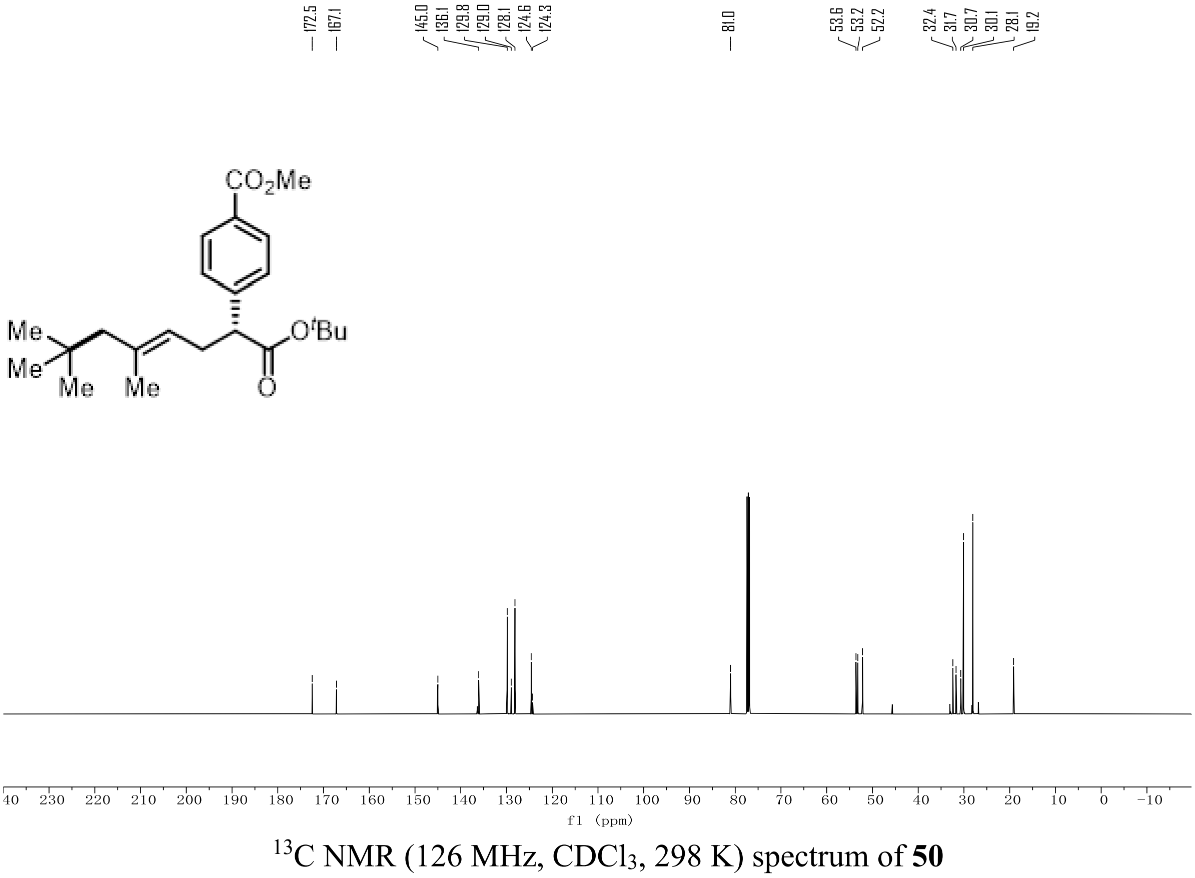

^{13}C NMR (126 MHz, CDCl$_3$, 298 K) spectrum of **50**

Methyl (*R,E*)-4-(1-(*tert*-butoxy)-4,7,7-trimethyl-1-oxooct-4-en-2-yl)benzoate (51)

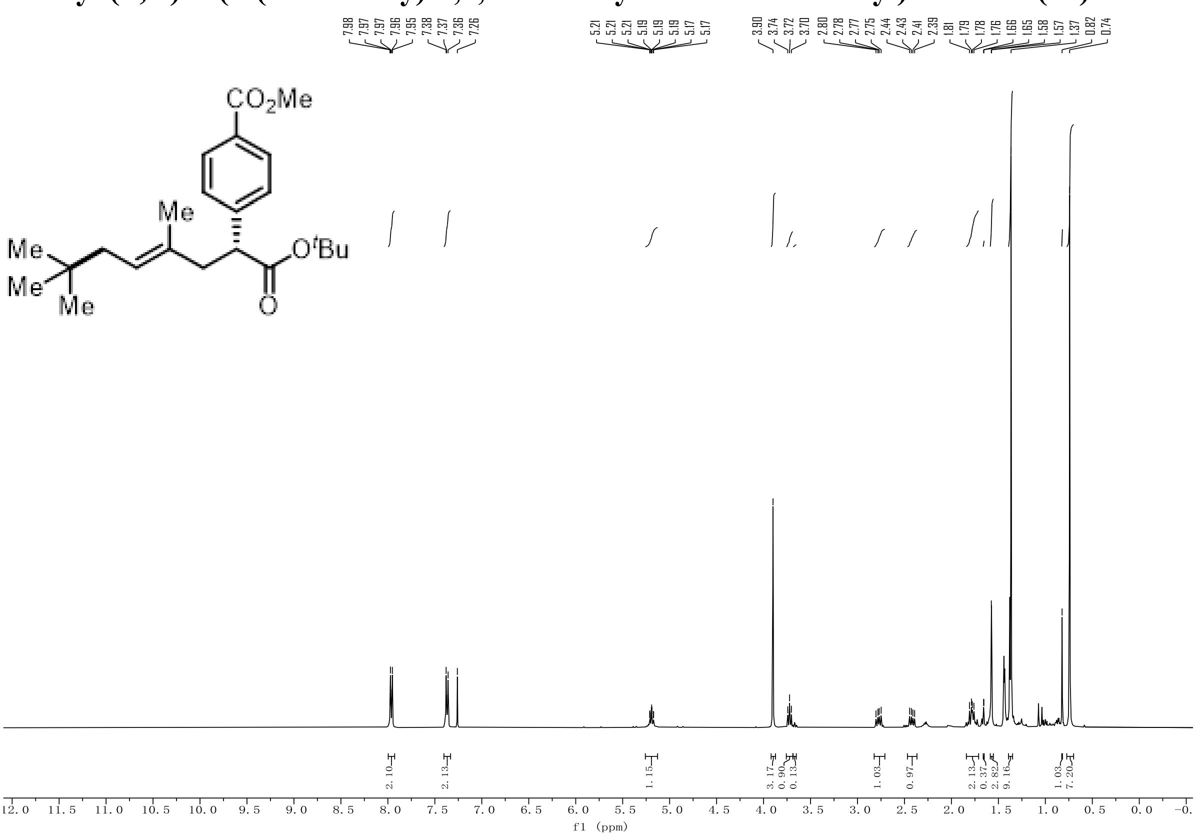

^{1}H NMR (400 MHz, $CDCl_3$, 298 K) spectrum of **51**

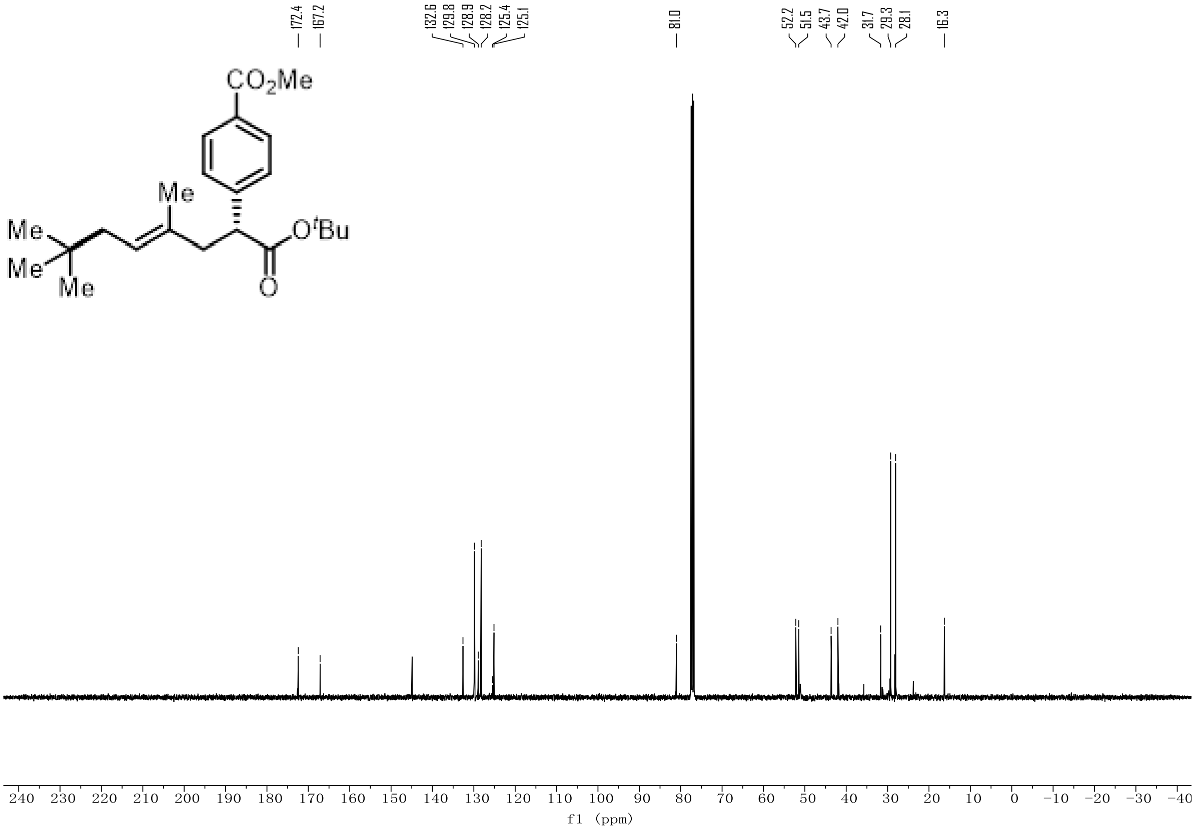

^{13}C NMR (101 MHz, $CDCl_3$, 298 K) spectrum of **51**

Methyl *(R,E)*-4-(1-(*tert*-butoxy)-7,7-dimethyl-1-oxo-9-phenylnon-4-en-2-yl)benzoate (52)

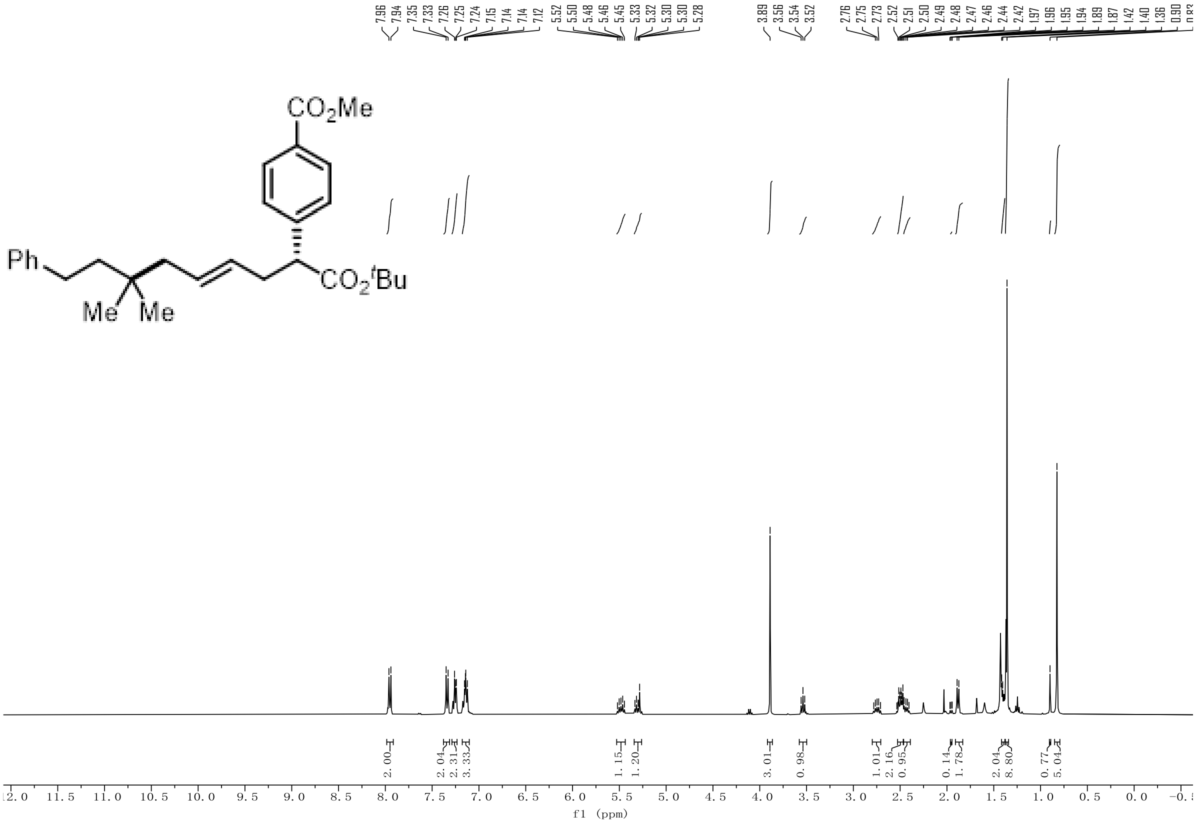

^{1}H NMR (400 MHz, CDCl$_3$, 298 K) spectrum of **52**

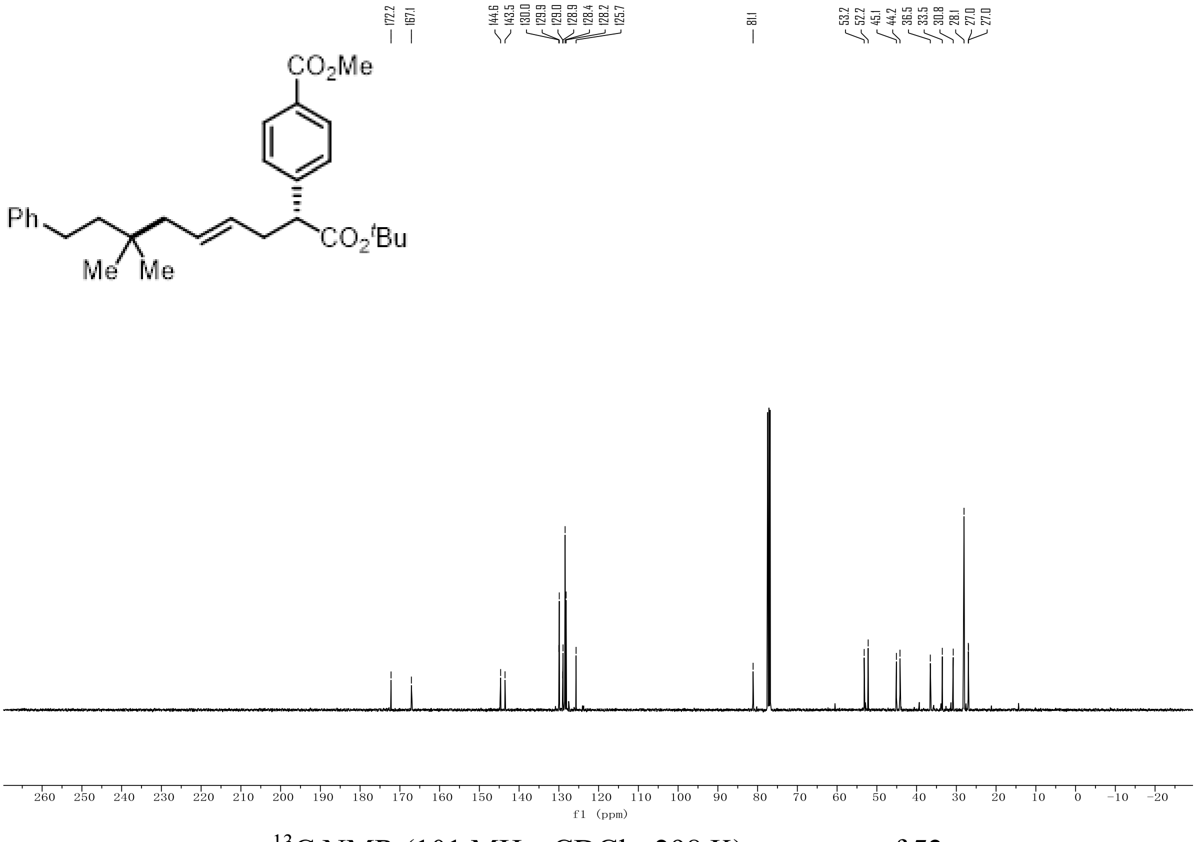

^{13}C NMR (101 MHz, CDCl$_3$, 298 K) spectrum of **52**

Methyl (*R,E*)-4-(1-(*tert*-butoxy)-7,7-dimethyl-1-oxo-8-phenyloct-4-en-2-yl)benzoate (53)

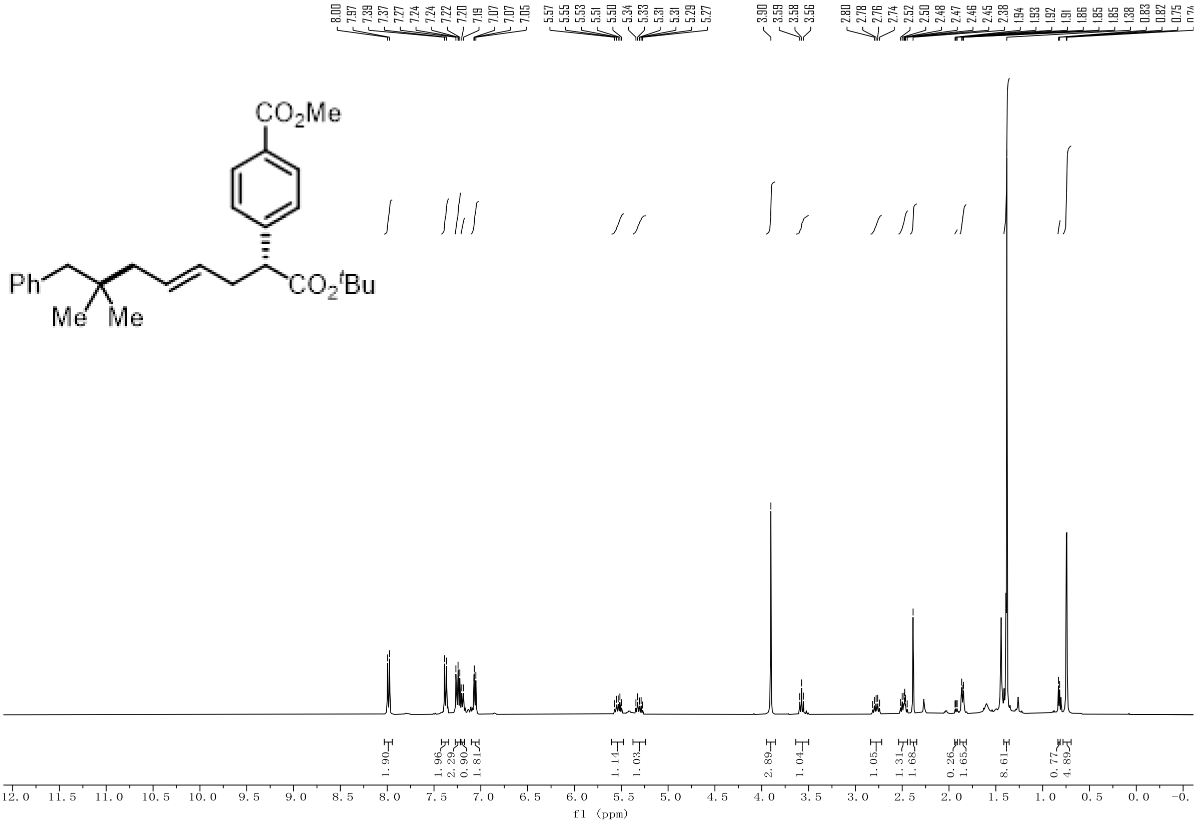

^{1}H NMR (400 MHz, $CDCl_3$, 298 K) spectrum of **53**

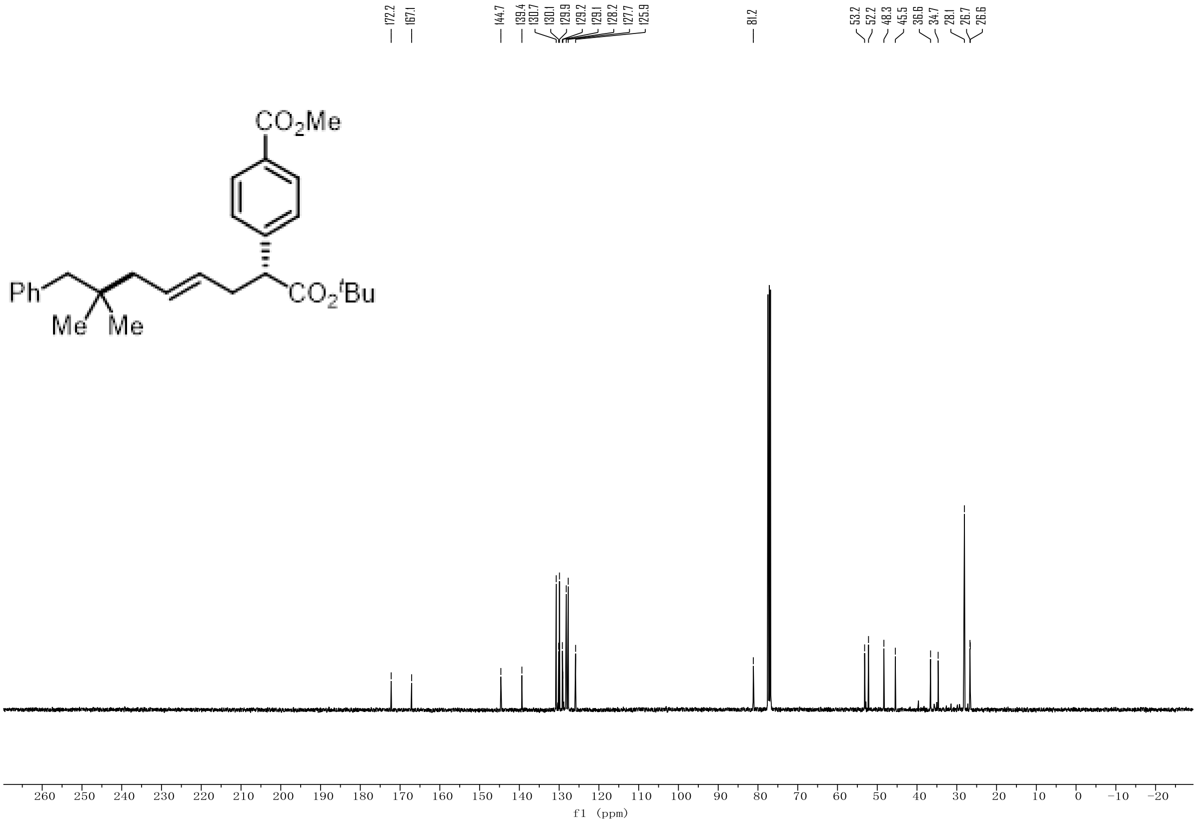

^{13}C NMR (101 MHz, $CDCl_3$, 298 K) spectrum of **53**

Methyl 4-((*R*,*E*)-6-((3R,5R,7R)-adamantan-1-yl)-1-(tert-butoxy)-1-oxohex-4-en-2-yl)benzoate (54)

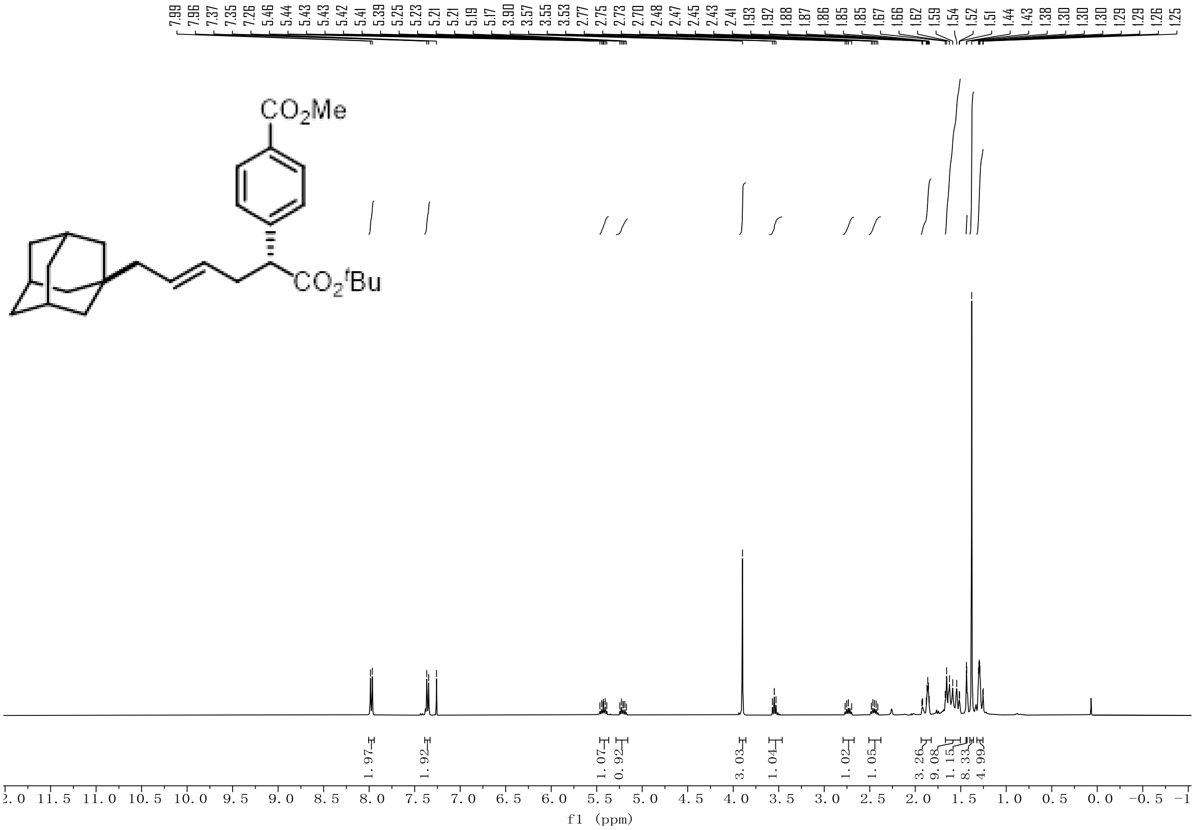

^{1}H NMR (400 MHz, $CDCl_3$, 298 K) spectrum of **54**

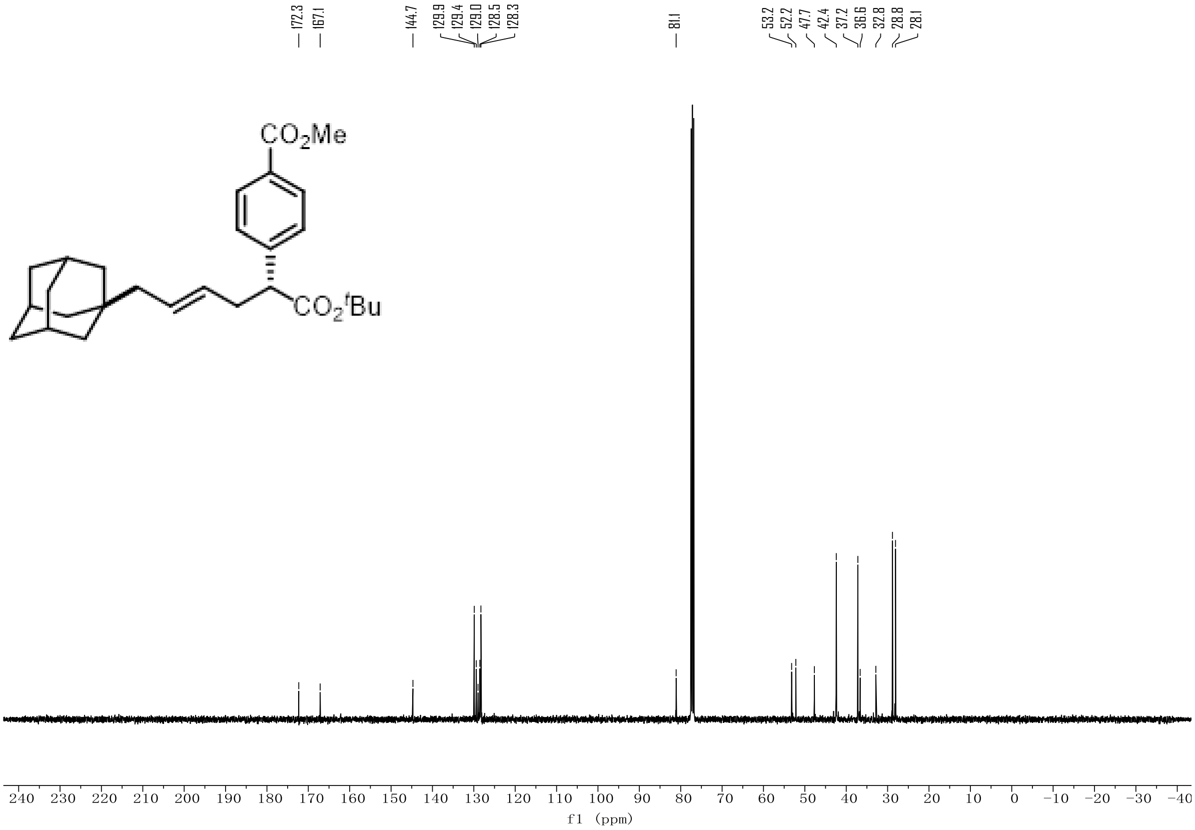

^{13}C NMR (101 MHz, $CDCl_3$, 298 K) spectrum of **54**

Methyl (*R,E*)-4-(1-(*tert*-butoxy)-6-(1-methylcyclohexyl)-1-oxohex-4-en-2-yl)benzoate (55)

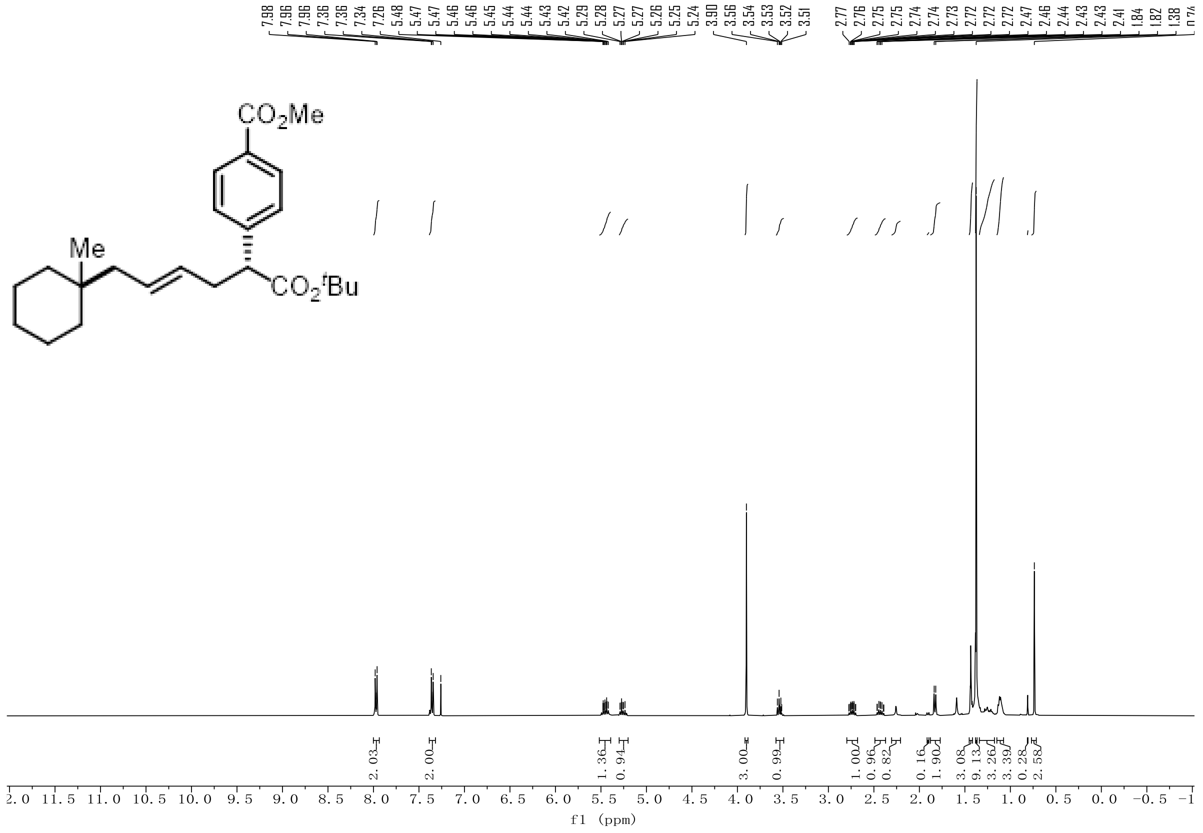

^{1}H NMR (400 MHz, $CDCl_3$, 298 K) spectrum of **55**

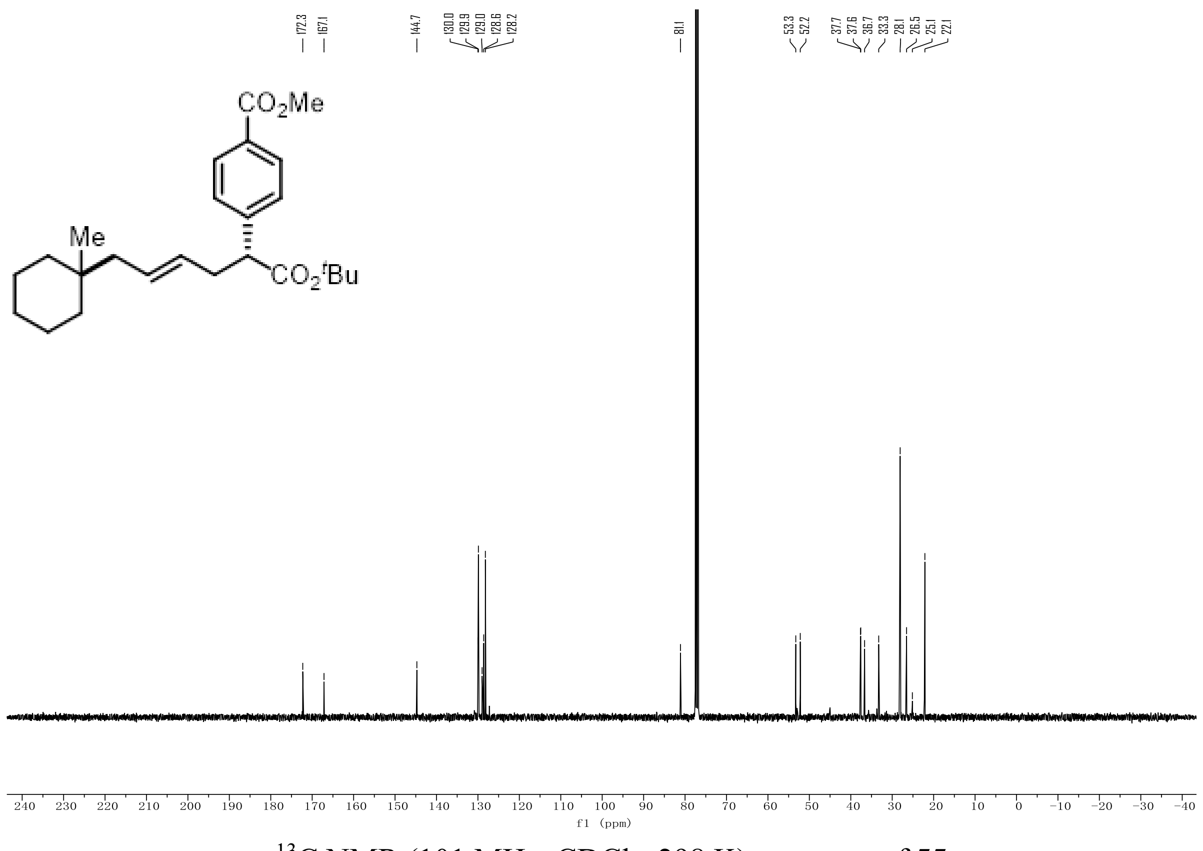

^{13}C NMR (101 MHz, $CDCl_3$, 298 K) spectrum of **55**

(8*R*,9*S*,13*R*,14*S*)-13-Methyl-17-oxo-7,8,9,11,12,13,14,15,16,17-decahydro-6H-cyclopenta[a]phenanthren-2-yl 4-((*R*,*E*)-1-(*tert*-butoxy)-1-oxo-6-(phenylsulfonyl)hex-4-en-2-yl)benzoate (56)

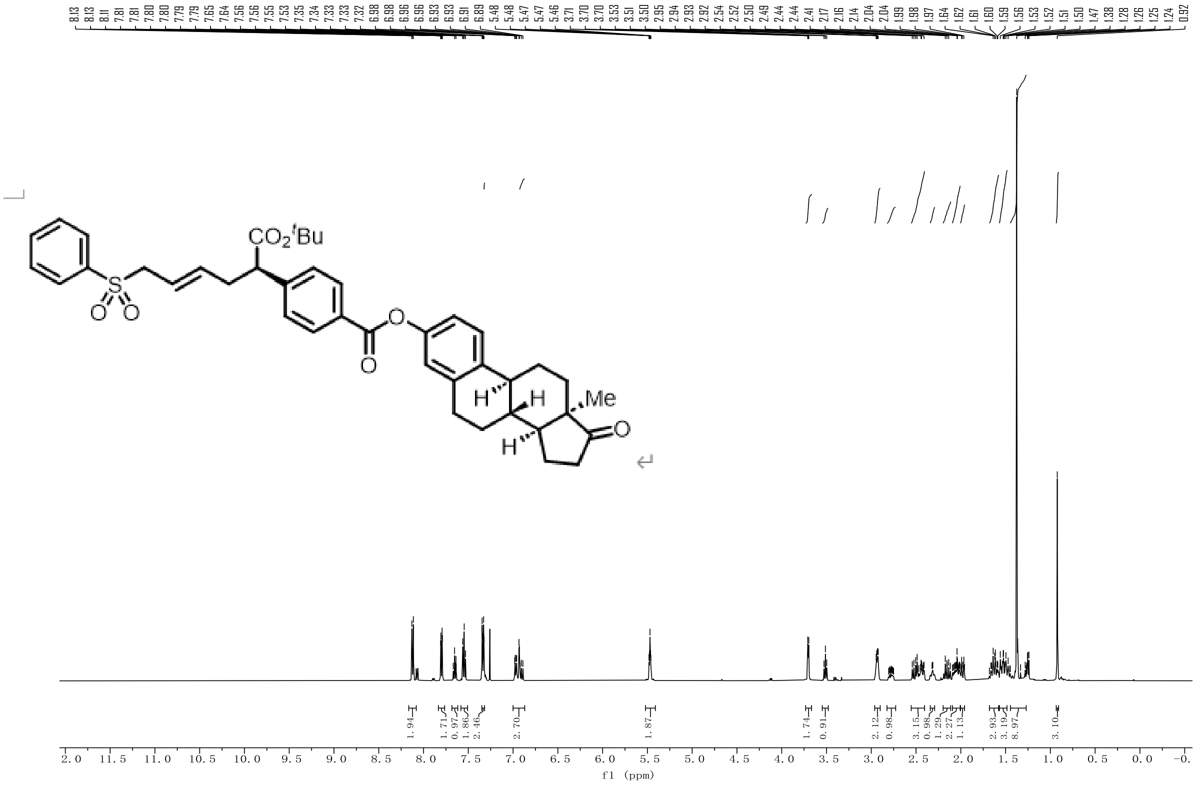

^{1}H NMR (500 MHz, $CDCl_3$, 298 K) spectrum of **56**

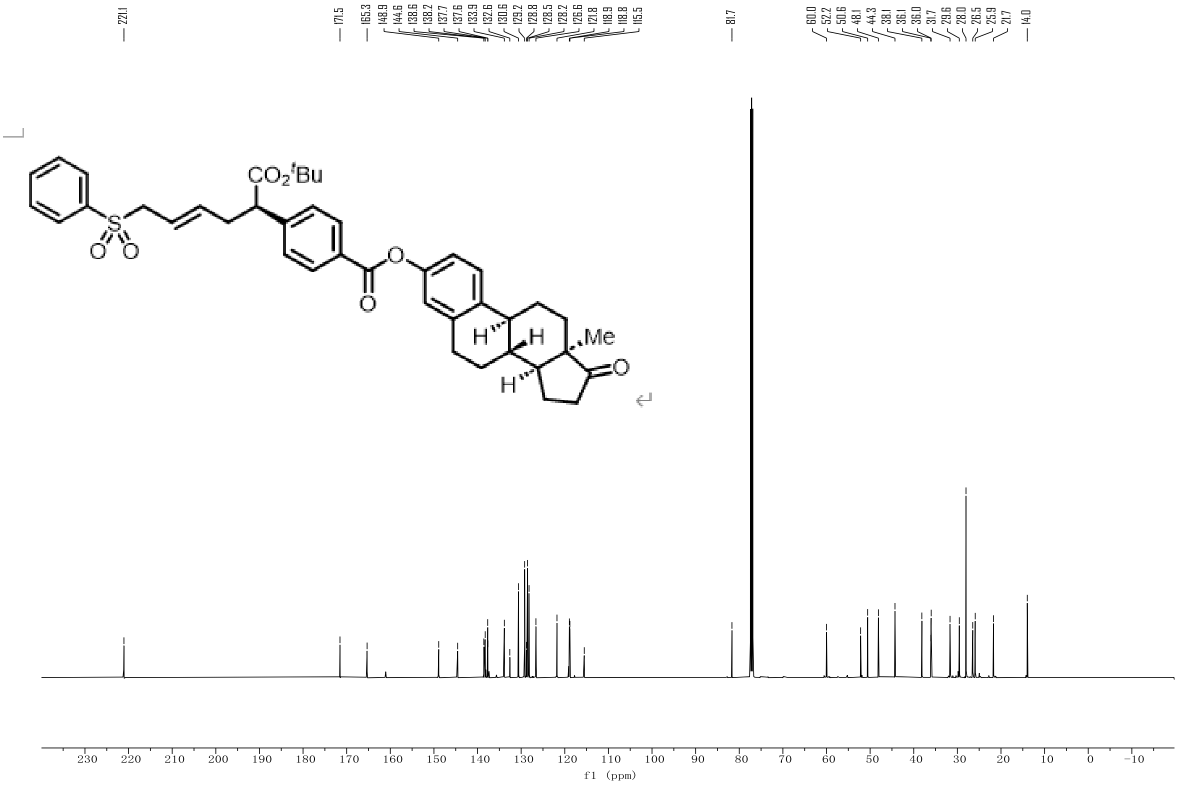

^{13}C NMR (126 MHz, $CDCl_3$, 298 K) spectrum of **56**

(1*S*,2*R*,4*R*)-2-Isopropyl-4-methylcyclohexyl 4-((*R*,*E*)-1-(*tert*-butoxy)-1-oxo-6-(phenylsulfonyl)hex-4-en-2-yl)benzoate (57)

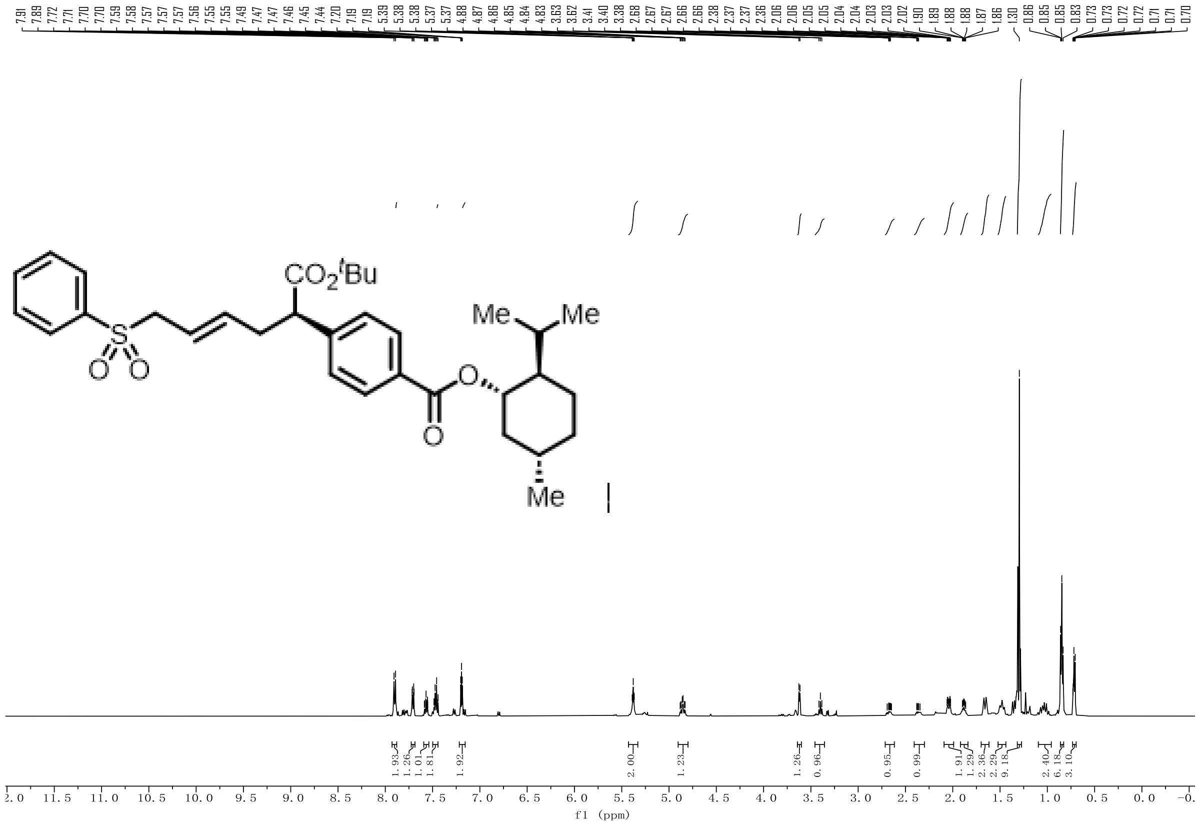

^{1}H NMR (500 MHz, $CDCl_3$, 298 K) spectrum of **57**

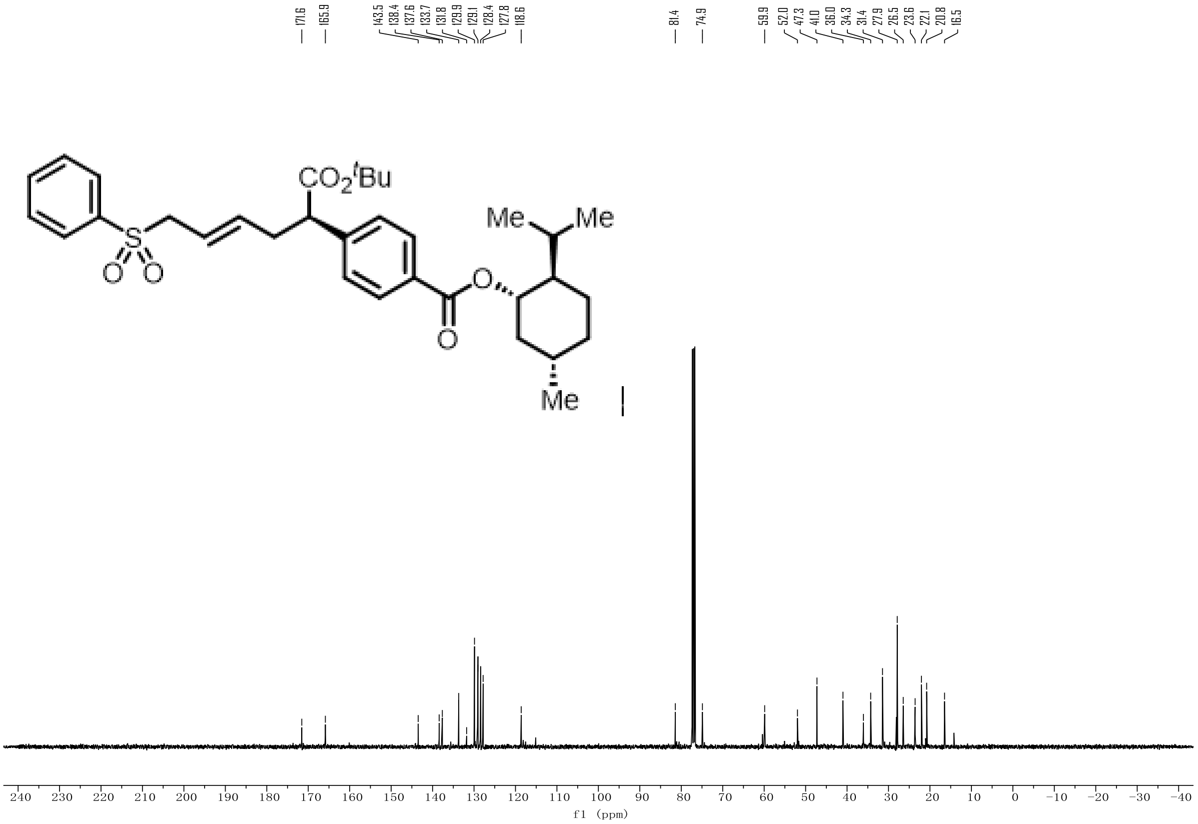

^{13}C NMR (101 MHz, $CDCl_3$, 298 K) spectrum of **57**

(1*S*,2*R*,4*R*)-2-Isopropyl-4-methylcyclohexyl 4-((*R*,*E*)-1-(tert-butoxy)-7,7-dimethyl-1-oxooct-4-en-2-yl)benzoate (58)

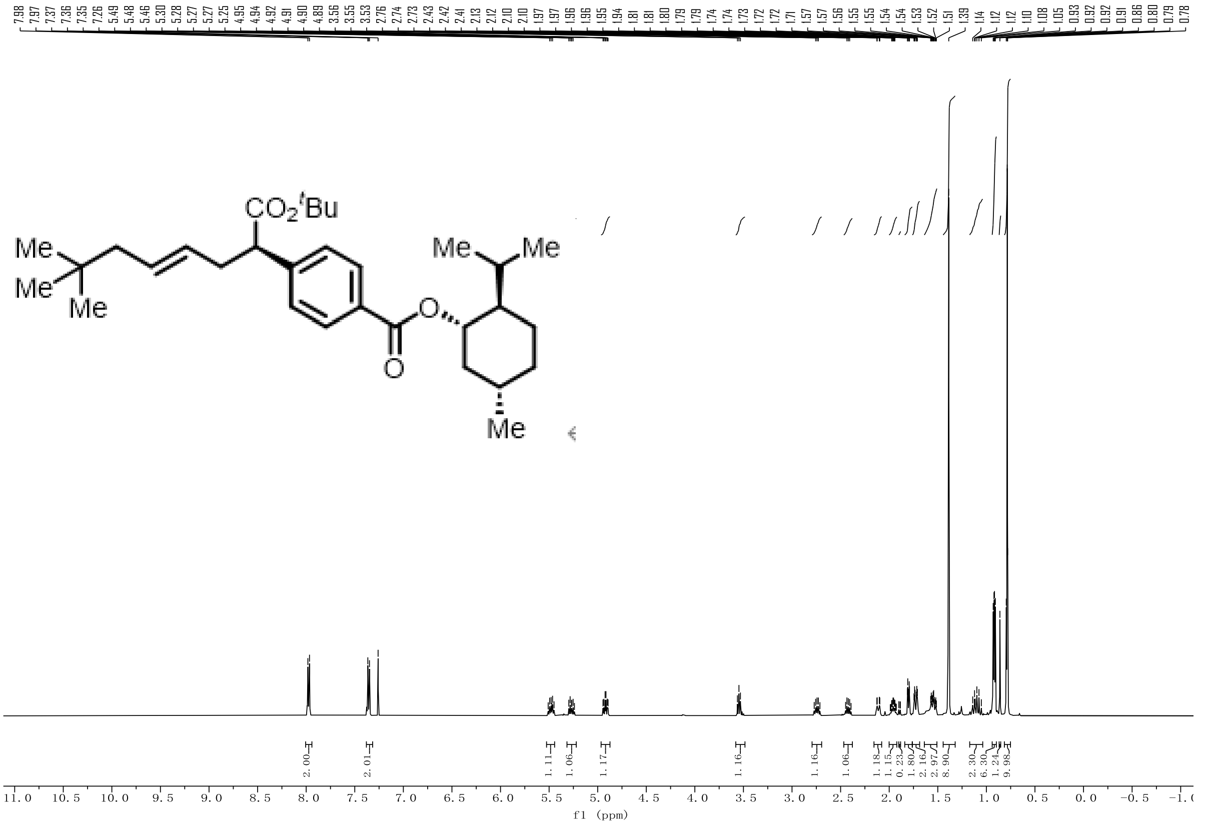

^{1}H NMR (500 MHz, $CDCl_3$, 298 K) spectrum of **58**

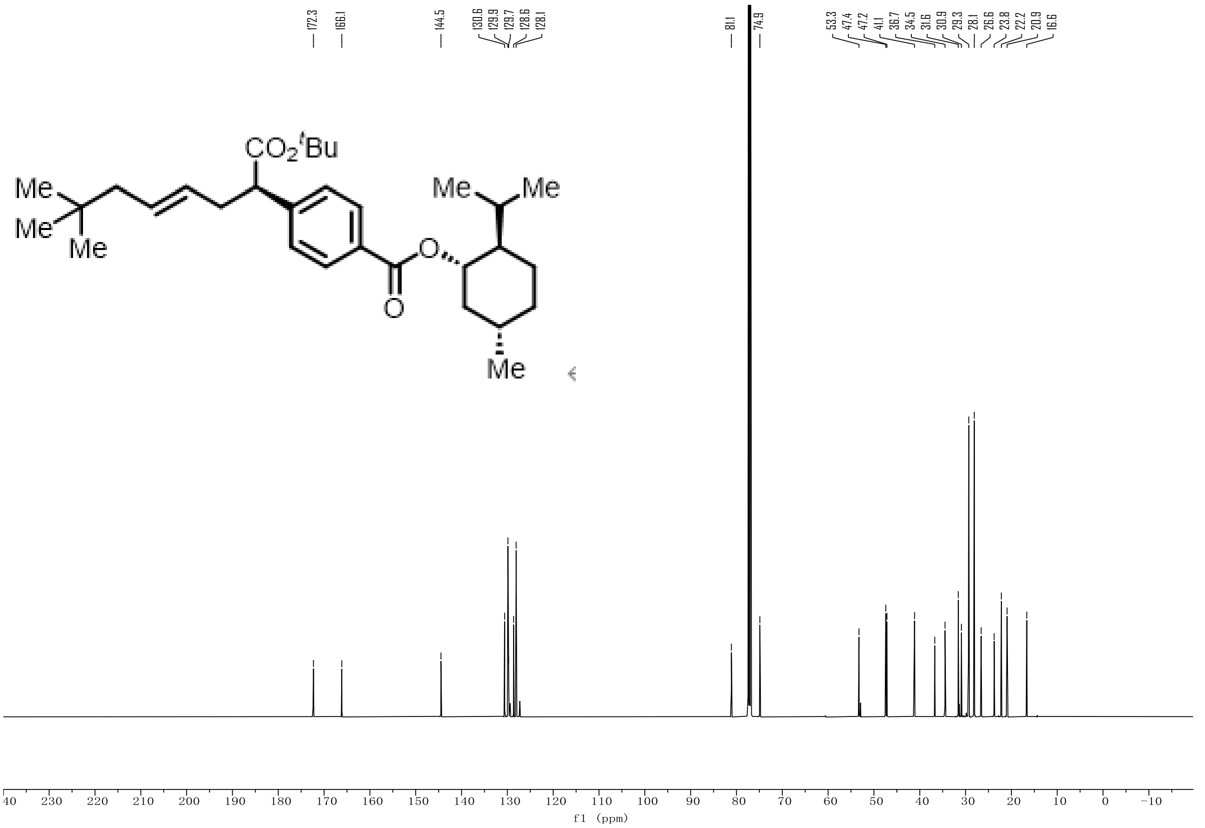

^{13}C NMR (126 MHz, $CDCl_3$, 298 K) spectrum of **58**

(*13R*)-13-Methyl-17-oxo-7,8,9,11,12,13,14,15,16,17-decahydro-6H-cyclopenta[a]phenanthren-2-yl 4-((*R,E*)-1-(*tert*-butoxy)-1-oxo-6-(phenylsulfonyl)hex-4-en-2-yl)benzoate (59)

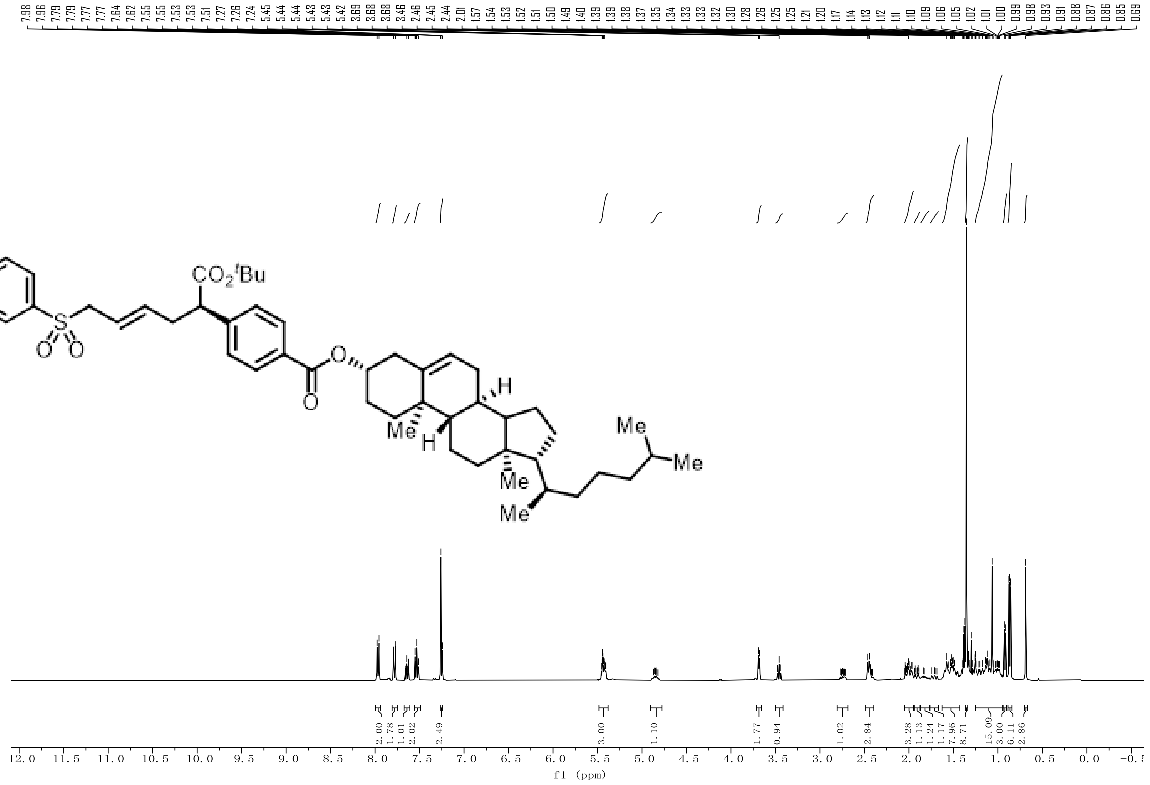

^{1}H NMR (400 MHz, $CDCl_3$, 298 K) spectrum of **59**

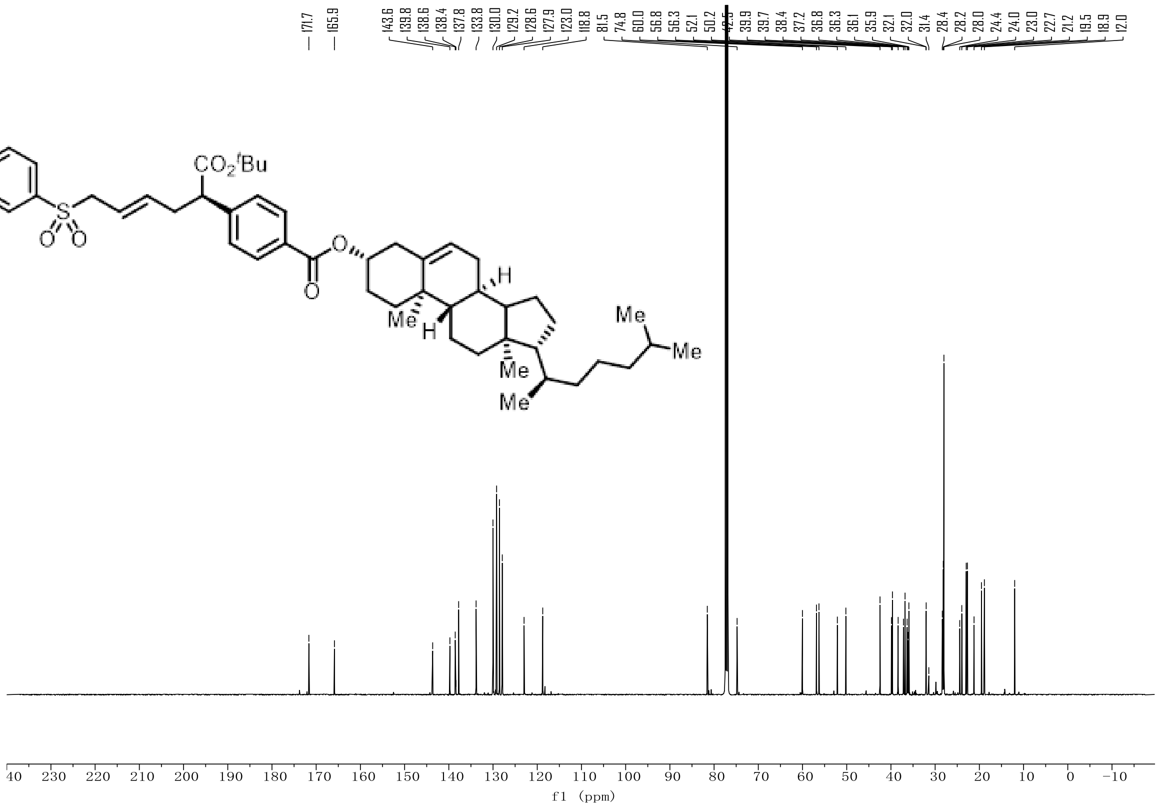

^{13}C NMR (126 MHz, $CDCl_3$, 298 K) spectrum of **59**

(3*S*,8*S*,9*S*,10*R*,13*R*,17*R*)-10,13-Dimethyl-17-((*R*)-6-methylheptan-2-yl)-2,3,4,7,8,9,10,11,12,13,14,15,16,17-tetradecahydro-1H-cyclopenta[a]phenanthren-3-yl 4-((*R*,*E*)-1-(*tert*-butoxy)-7,7-dimethyl-1-oxooct-4-en-2-yl)benzoate (60)

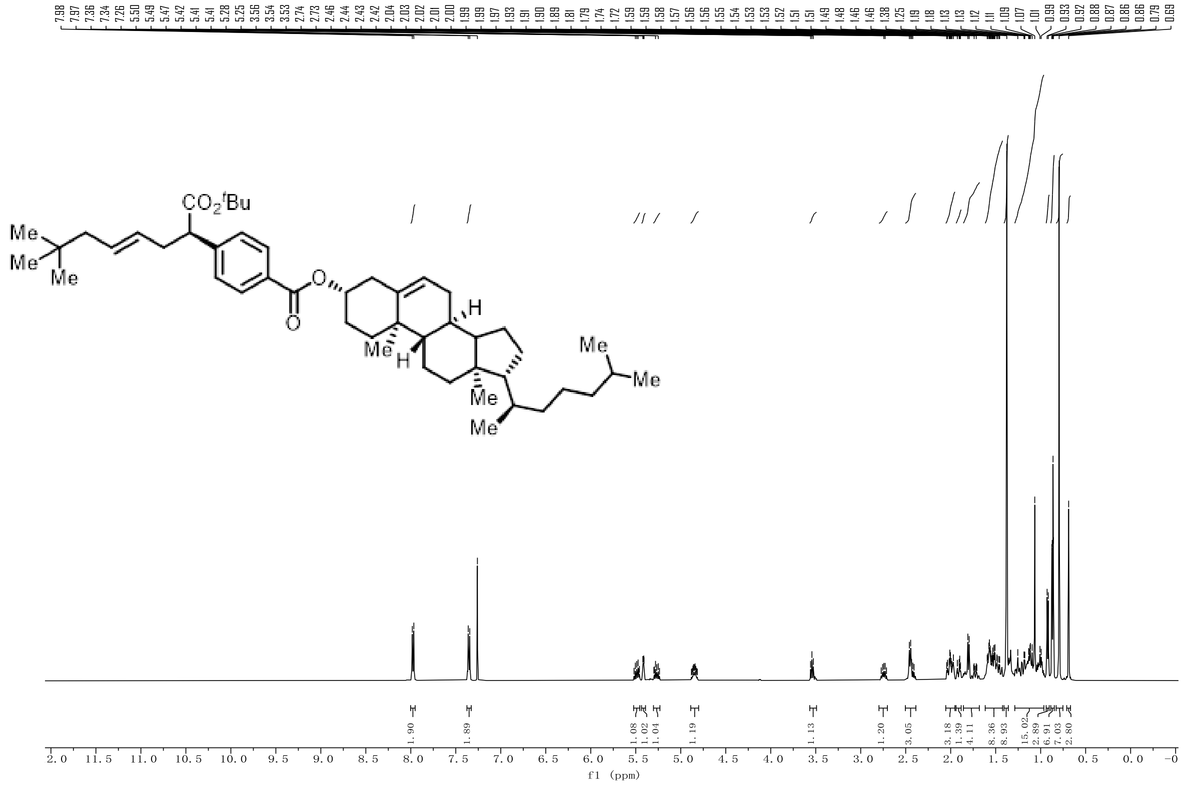

^{1}H NMR (500 MHz, CDCl$_3$, 298 K) spectrum of **60**

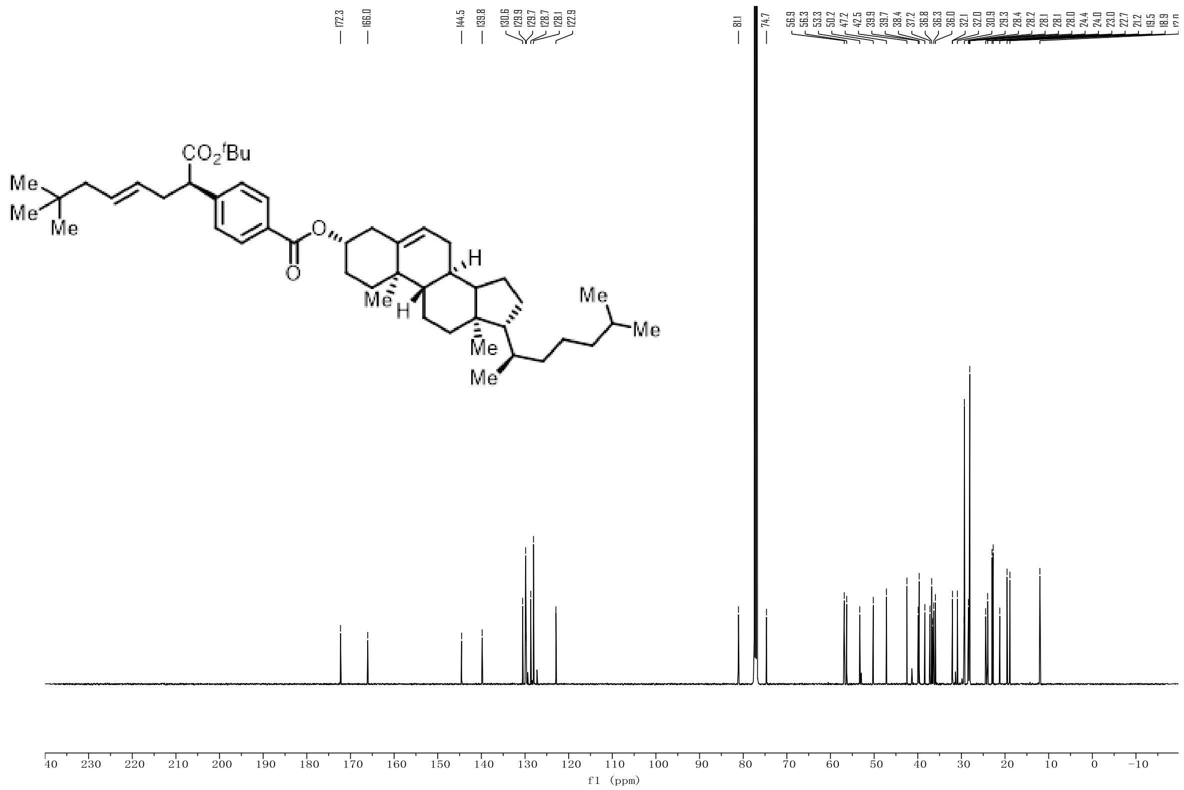

^{13}C NMR (126 MHz, CDCl$_3$, 298 K) spectrum of **60**

(3a*R*,5*R*,6*S*,6a*R*)-5-((*S*)-2,2-dimethyl-1,3-dioxolan-4-yl)-2,2-Dimethyltetrahydrofuro[2,3-d][1,3]dioxol-6-yl 4-((*R,E*)-1-(*tert*-butoxy)-1-oxo-6-(phenylsulfonyl)hex-4-en-2-yl)benzoate (61)

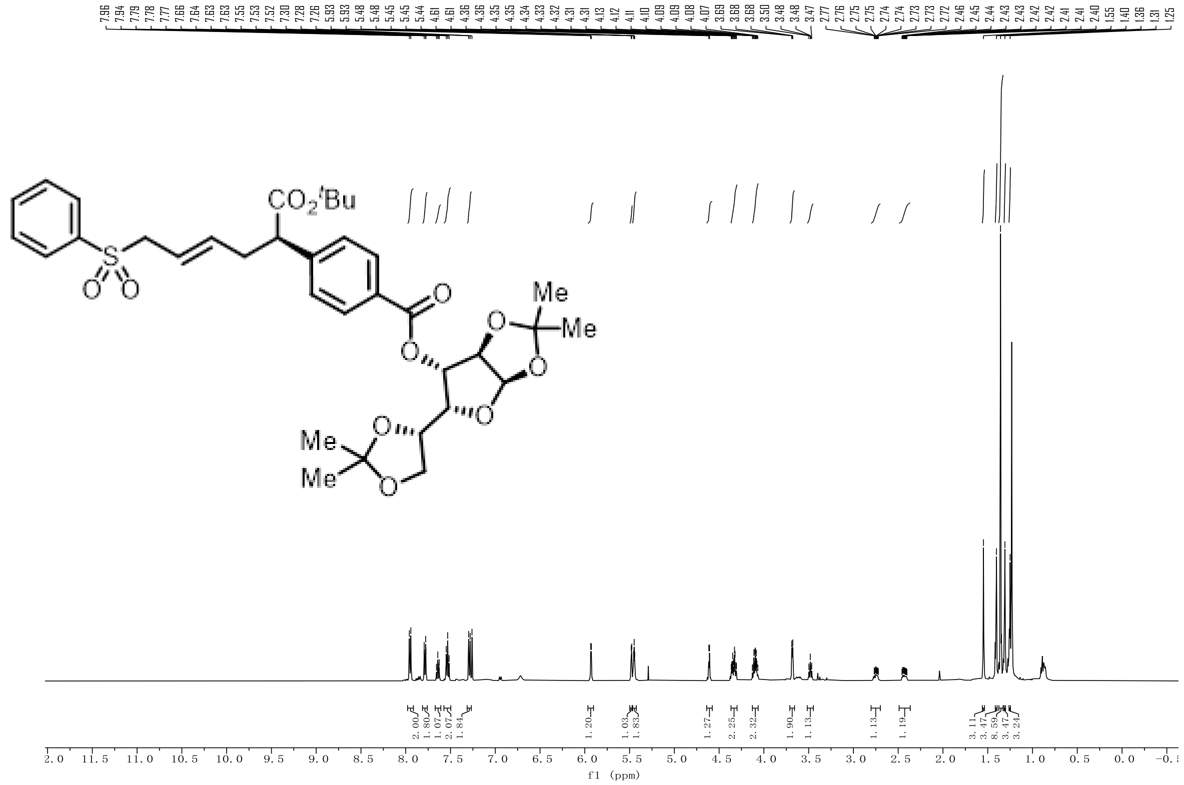

^{1}H NMR (500 MHz, $CDCl_3$, 298 K) spectrum of **61**

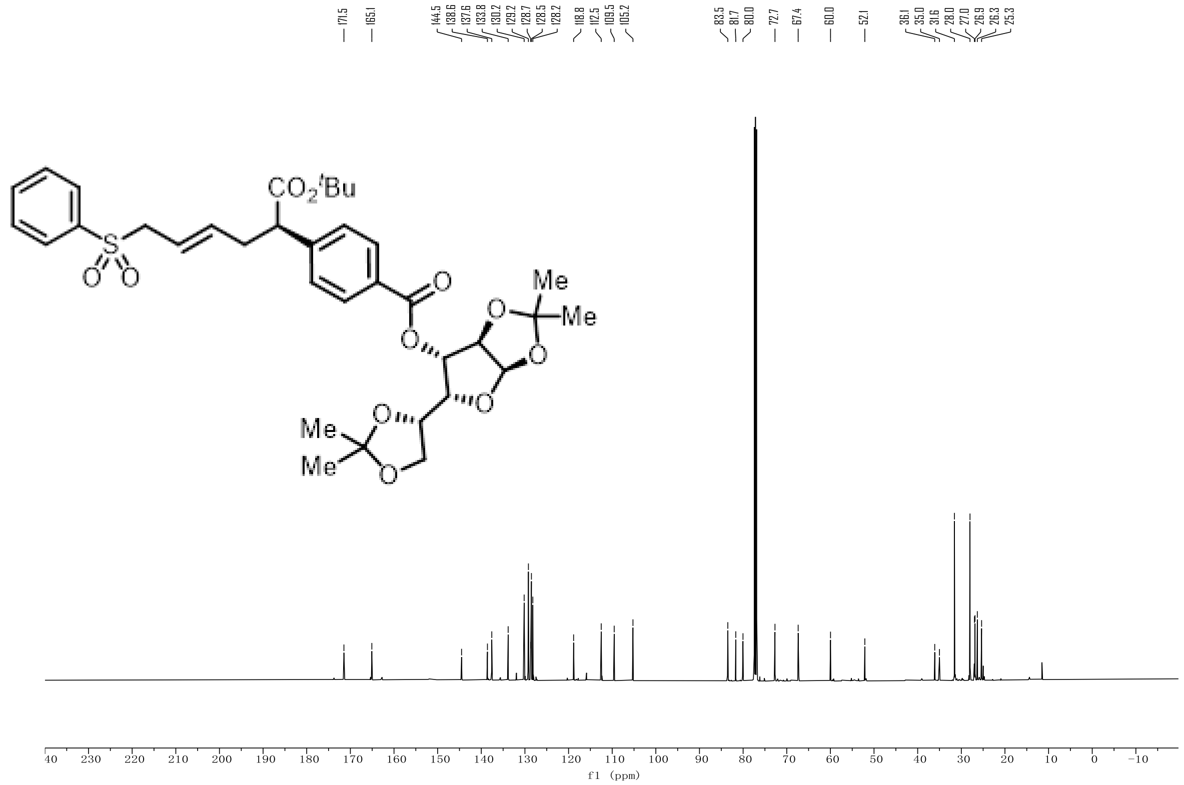

^{13}C NMR (126 MHz, $CDCl_3$, 298 K) spectrum of **61**

(3a*R*,5*R*,6*S*,6a*R*)-5-((*S*)-2,2-Dimethyl-1,3-dioxolan-4-yl)-2,2-dimethyltetrahydrofuro[2,3-d][1,3]dioxol-6-yl 4-((*R*,*E*)-1-(*tert*-butoxy)-7,7-dimethyl-1-oxooct-4-en-2-yl)benzoate (62)

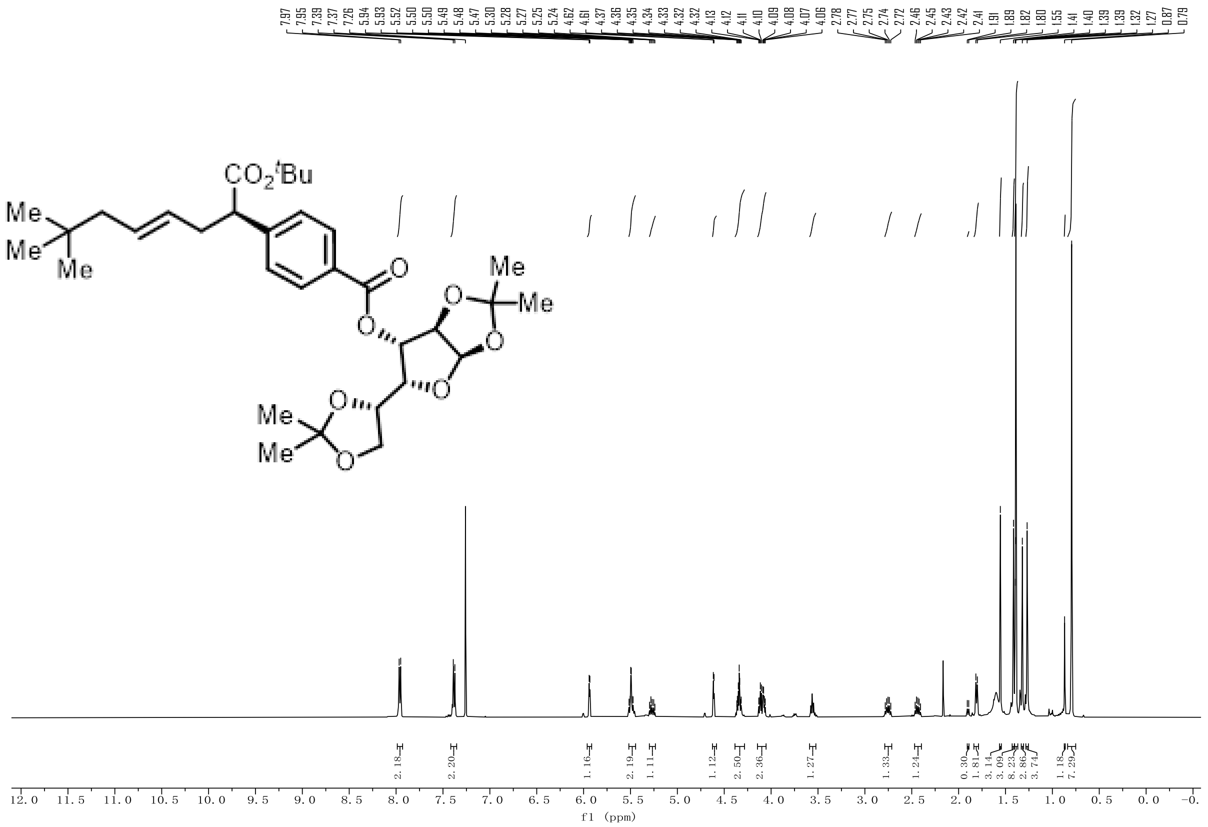

^{1}H NMR (500 MHz, $CDCl_3$, 298 K) spectrum of **62**

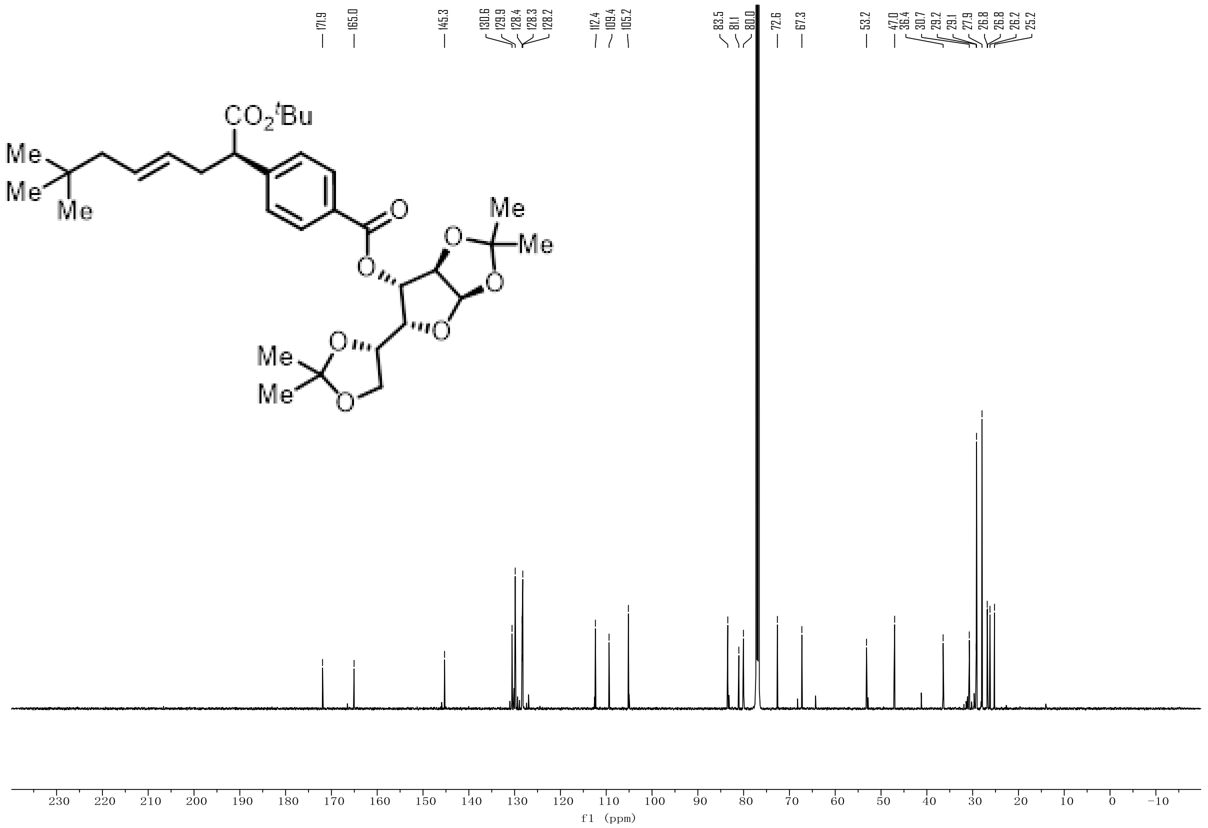

^{13}C NMR (126 MHz, $CDCl_3$, 298 K) spectrum of **62**

(*R*)-2,5,7,8-Tetramethyl-2-((4*R*,8*R*)-4,8,12-trimethyltridecyl)chroman-6-yl 4-((*R,E*)-1-(*tert*-butoxy)-1-oxo-6-(phenylsulfonyl)hex-4-en-2-yl)benzoate (63)

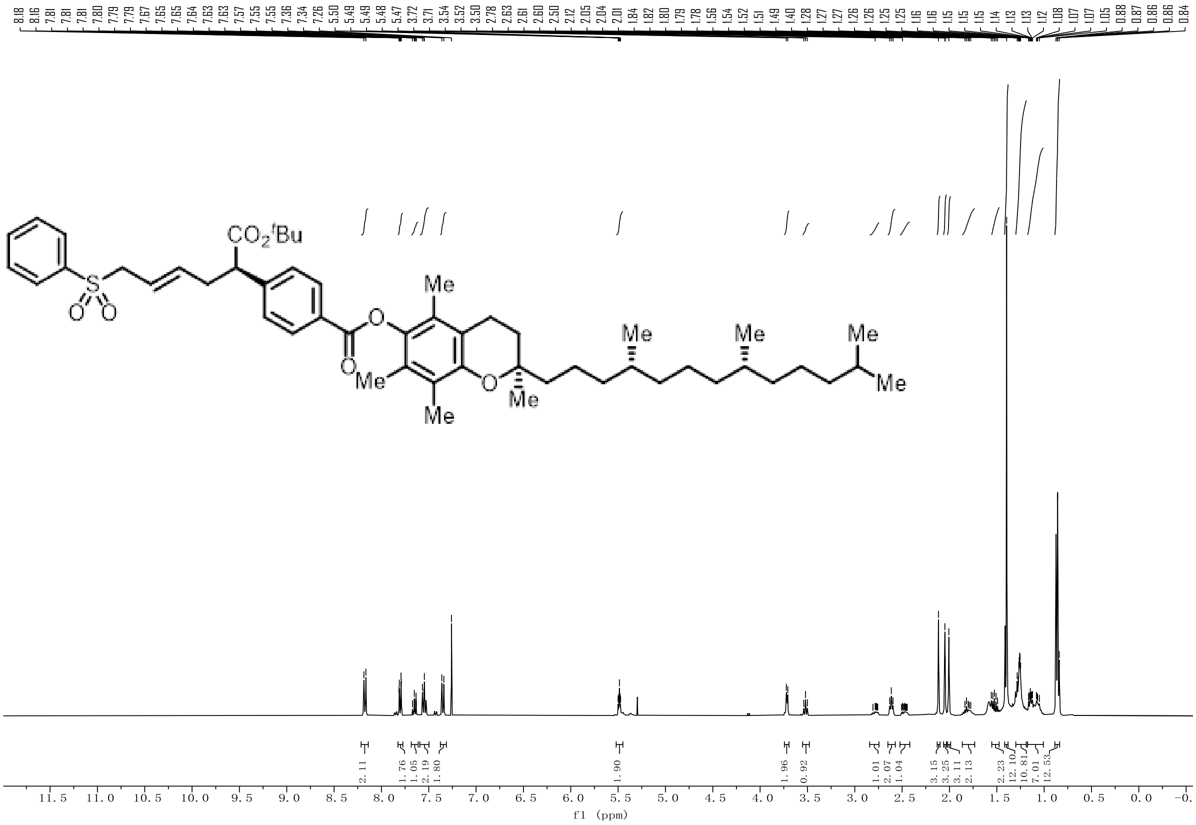

^{1}H NMR (400 MHz, $CDCl_3$, 298 K) spectrum of **63**

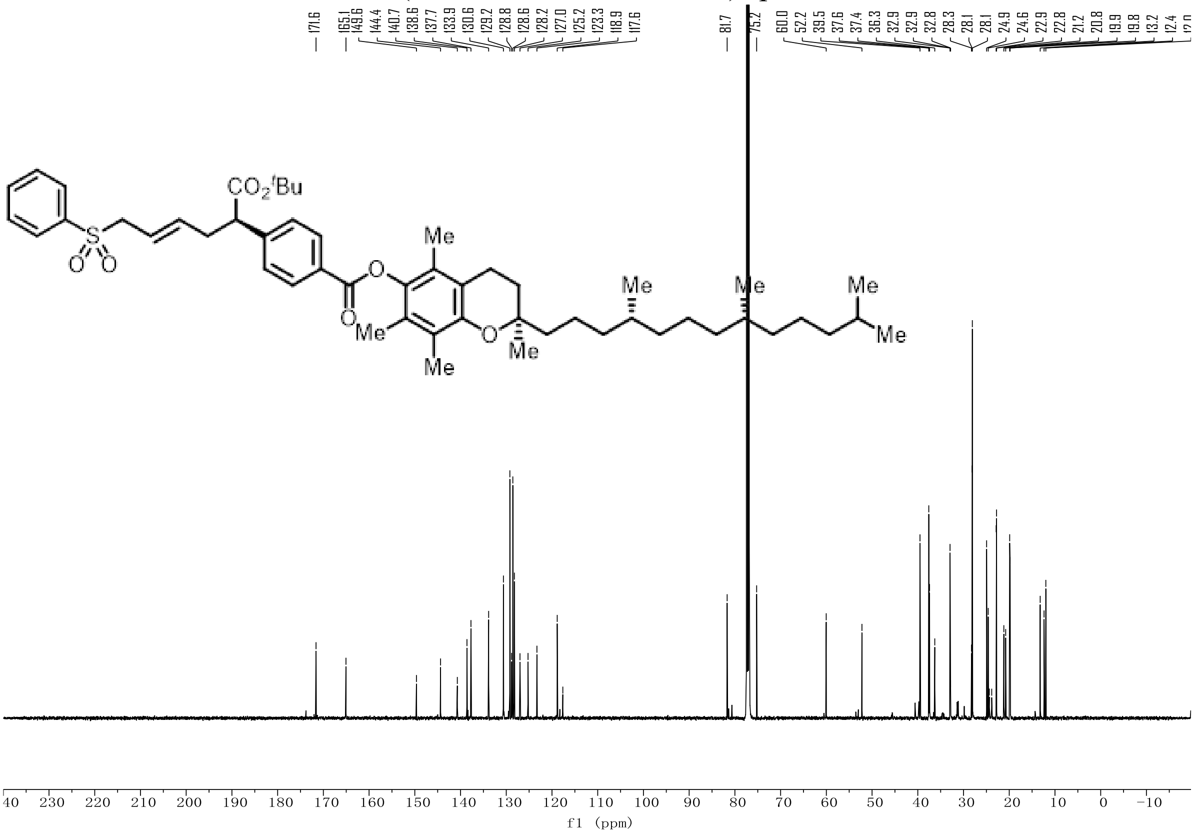

^{13}C NMR (126 MHz, $CDCl_3$, 298 K) spectrum of **63**

(*R*)-2,5,7,8-Tetramethyl-2-((4*R*,8*R*)-4,8,12-trimethyltridecyl)chroman-6-yl 4-((*R,E*)-1-(*tert*-butoxy)-7,7-dimethyl-1-oxooct-4-en-2-yl)benzoate (64)

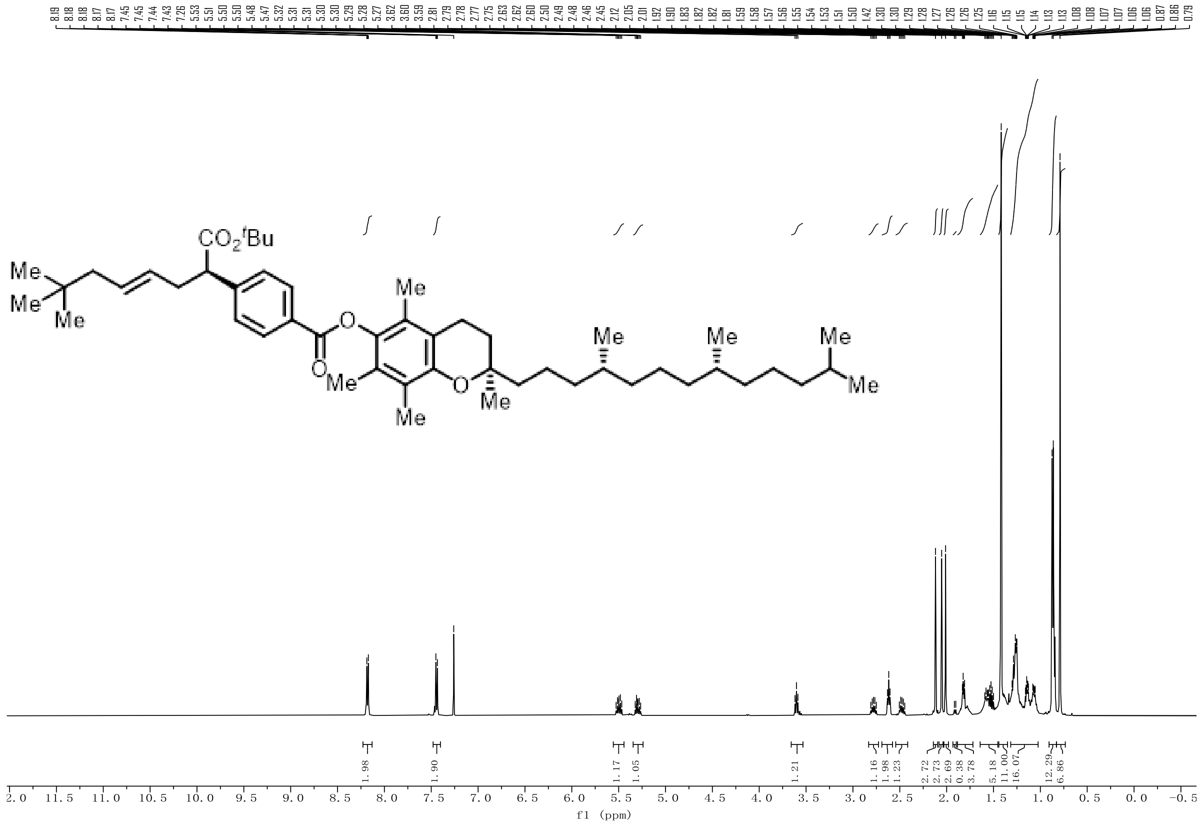

^{1}H NMR (500 MHz, $CDCl_3$, 298 K) spectrum of **64**

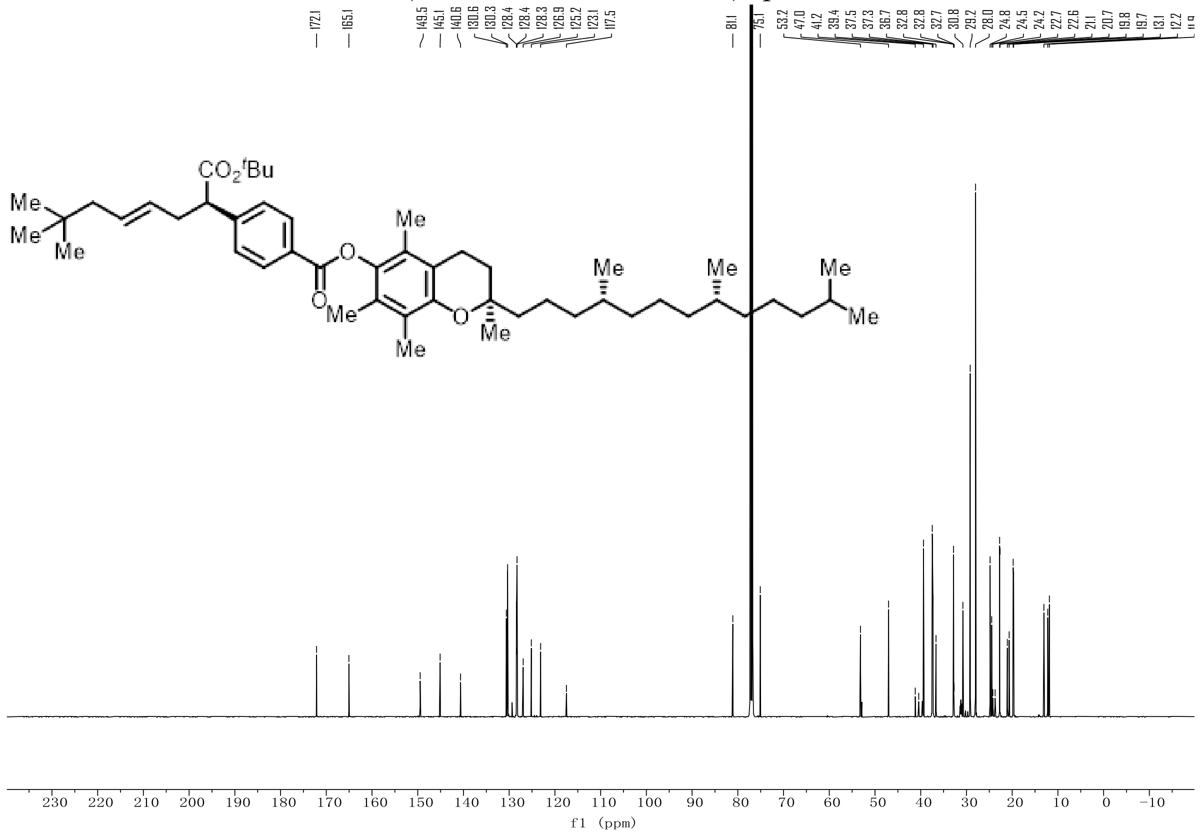

^{13}C NMR (126 MHz, $CDCl_3$, 298 K) spectrum of **64**

Methyl (*R*)-4-(1-(*tert*-butoxy)-1-oxo-6-(phenylsulfonyl)hexan-2-yl)benzoate (65)

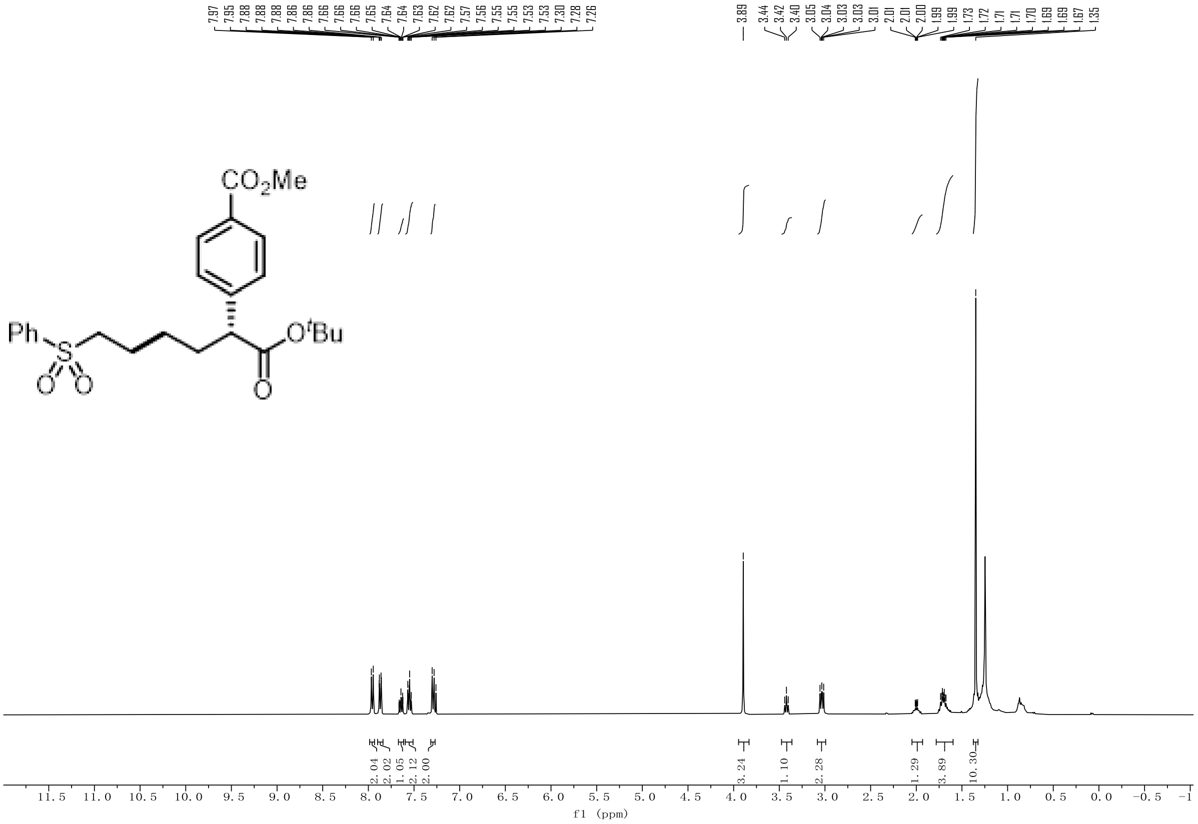

^{1}H NMR (400 MHz, CDCl$_3$, 298 K) spectrum of **65**

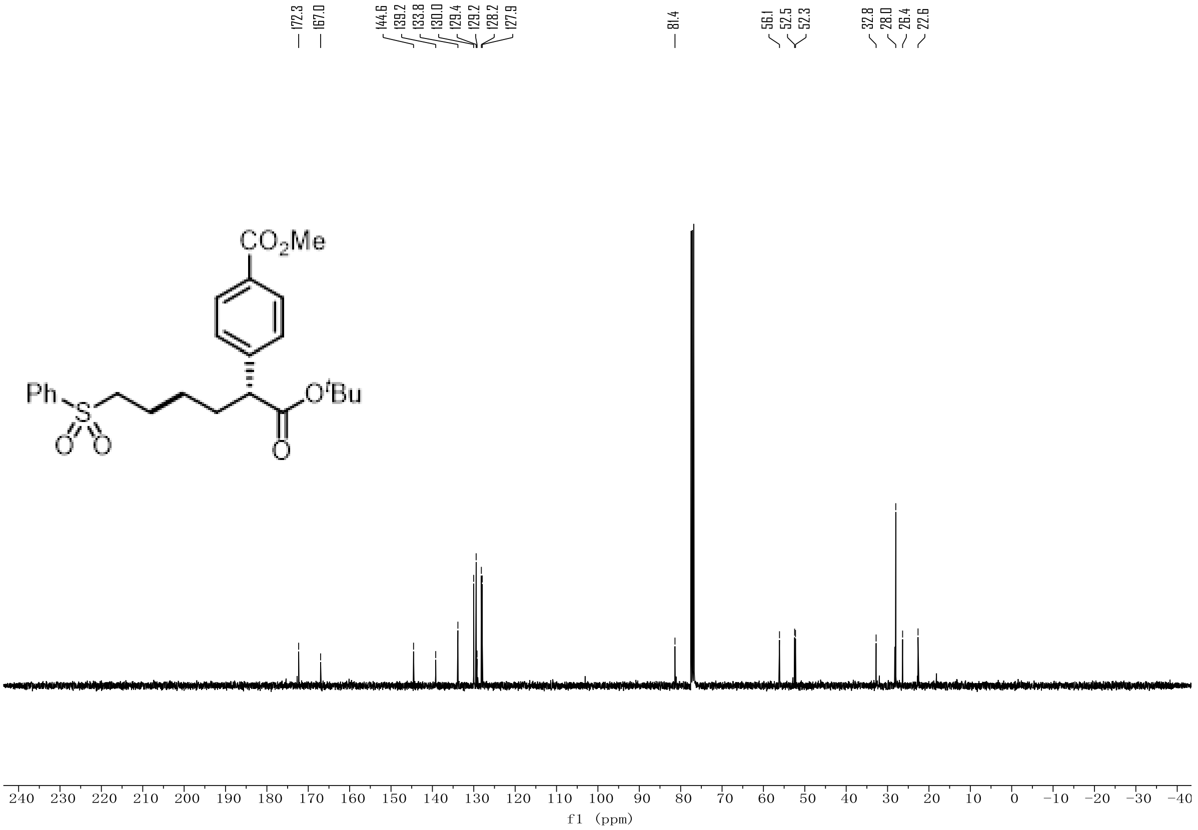

^{13}C NMR (101 MHz, CDCl$_3$, 298 K) spectrum of **65**

Methyl (*R*)-4-(1-(*tert*-butoxy)-7,7-dimethyl-1-oxooctan-2-yl)benzoate (66)

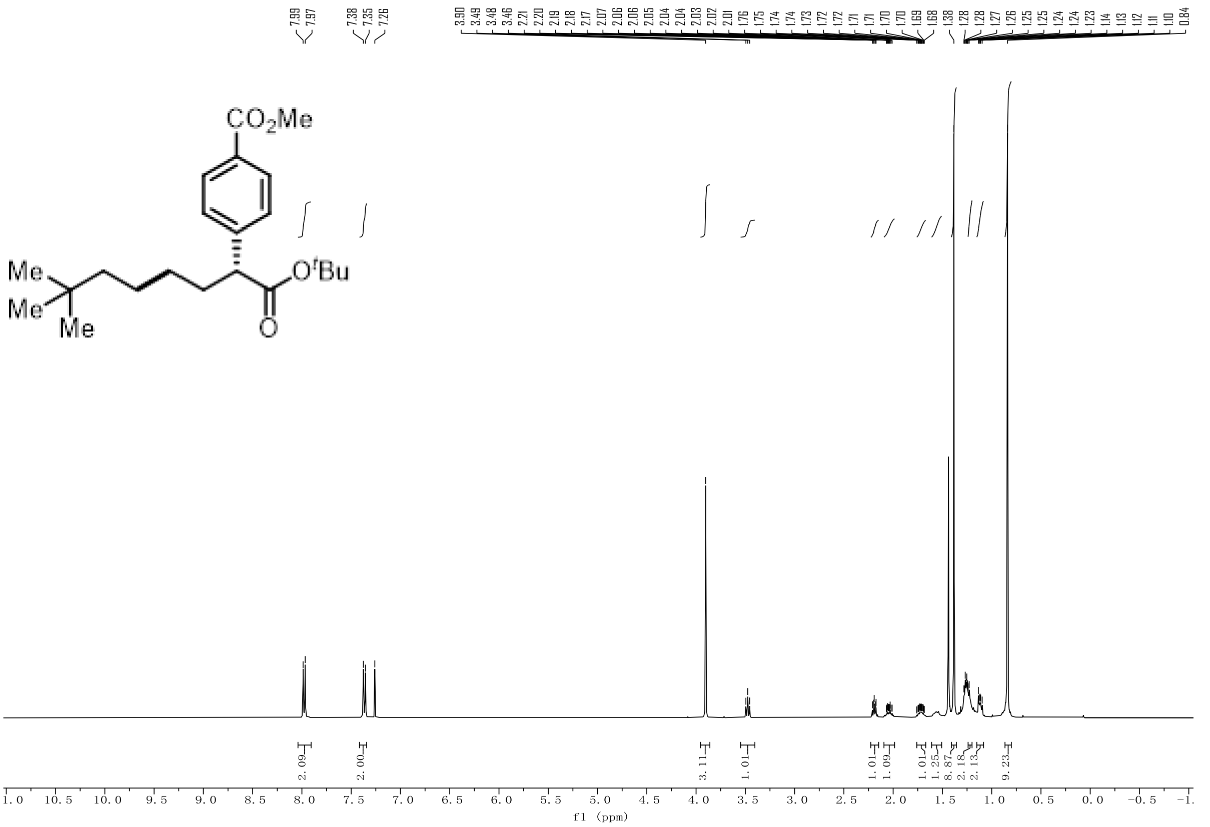

^{1}H NMR (400 MHz, $CDCl_3$, 298 K) spectrum of **66**

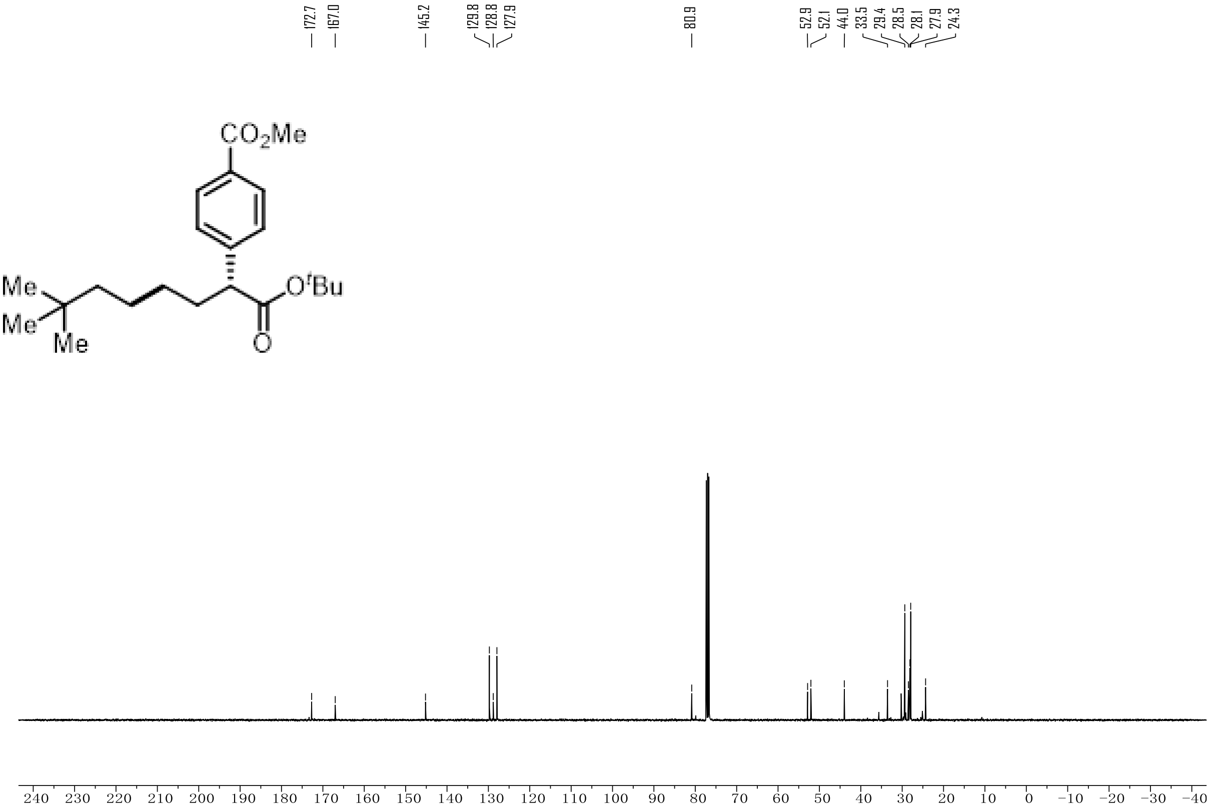

^{13}C NMR (101 MHz, $CDCl_3$, 298 K) spectrum of **66**

Methyl (*R,E*)-4-(1-methoxy-1-oxo-6-(phenylsulfonyl)hex-4-en-2-yl)benzoate (67)

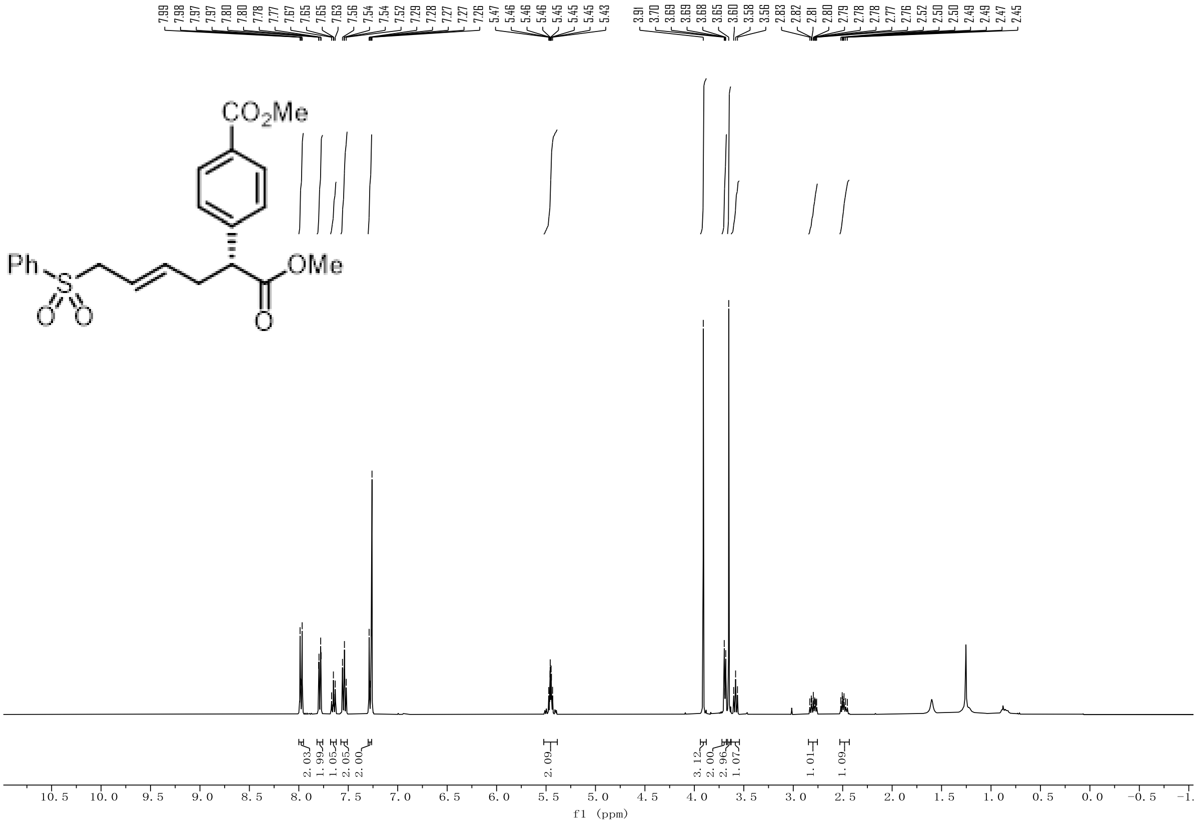

^{1}H NMR (400 MHz, $CDCl_3$, 298 K) spectrum of **67**

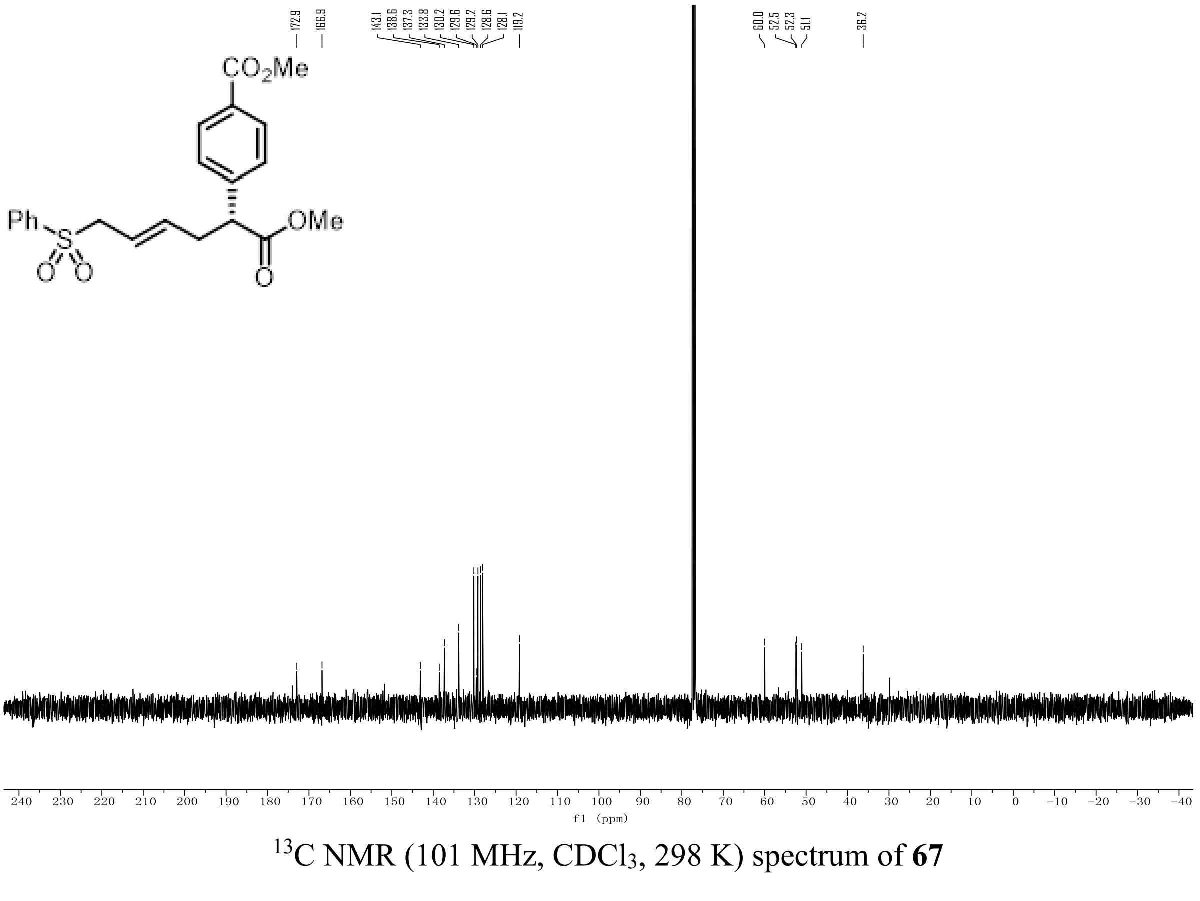

^{13}C NMR (101 MHz, $CDCl_3$, 298 K) spectrum of **67**

Methyl (*R,E*)-4-(1-methoxy-7,7-dimethyl-1-oxooct-4-en-2-yl)benzoate (68)

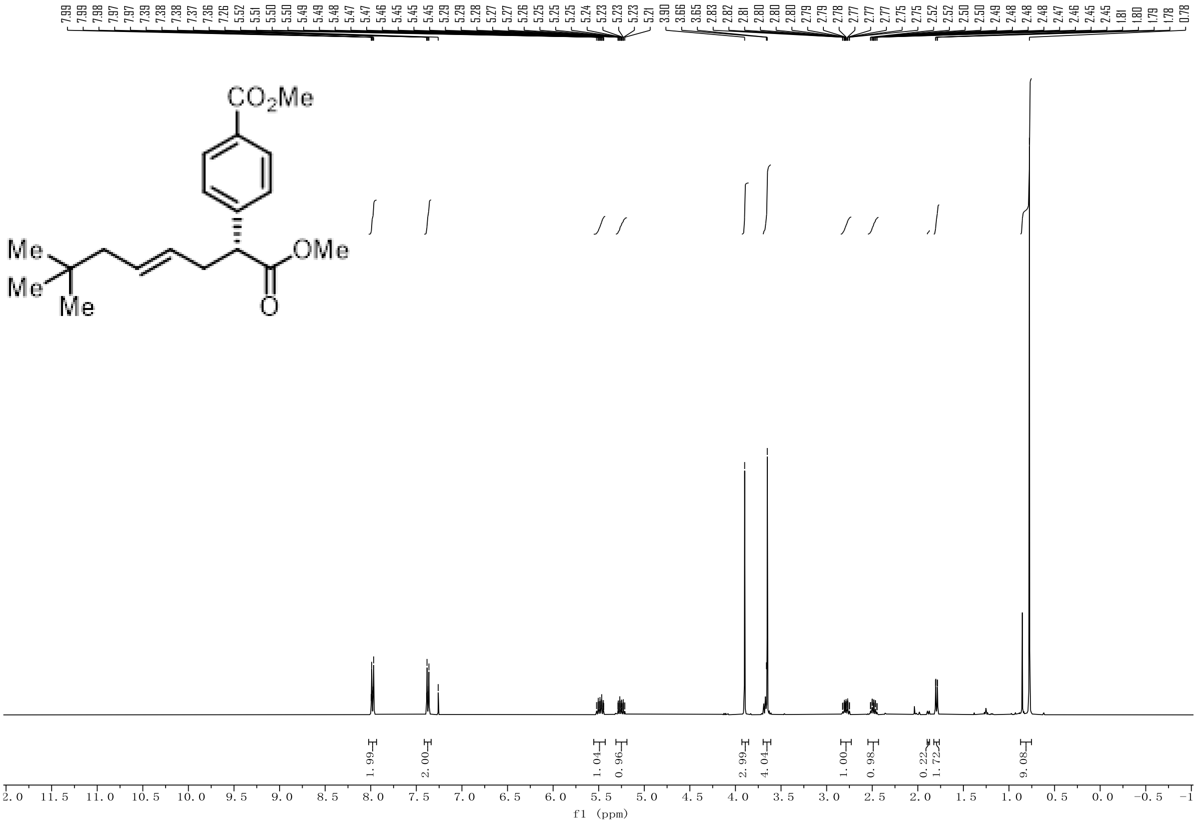

^{1}H NMR (400 MHz, $CDCl_3$, 298 K) spectrum of **68**

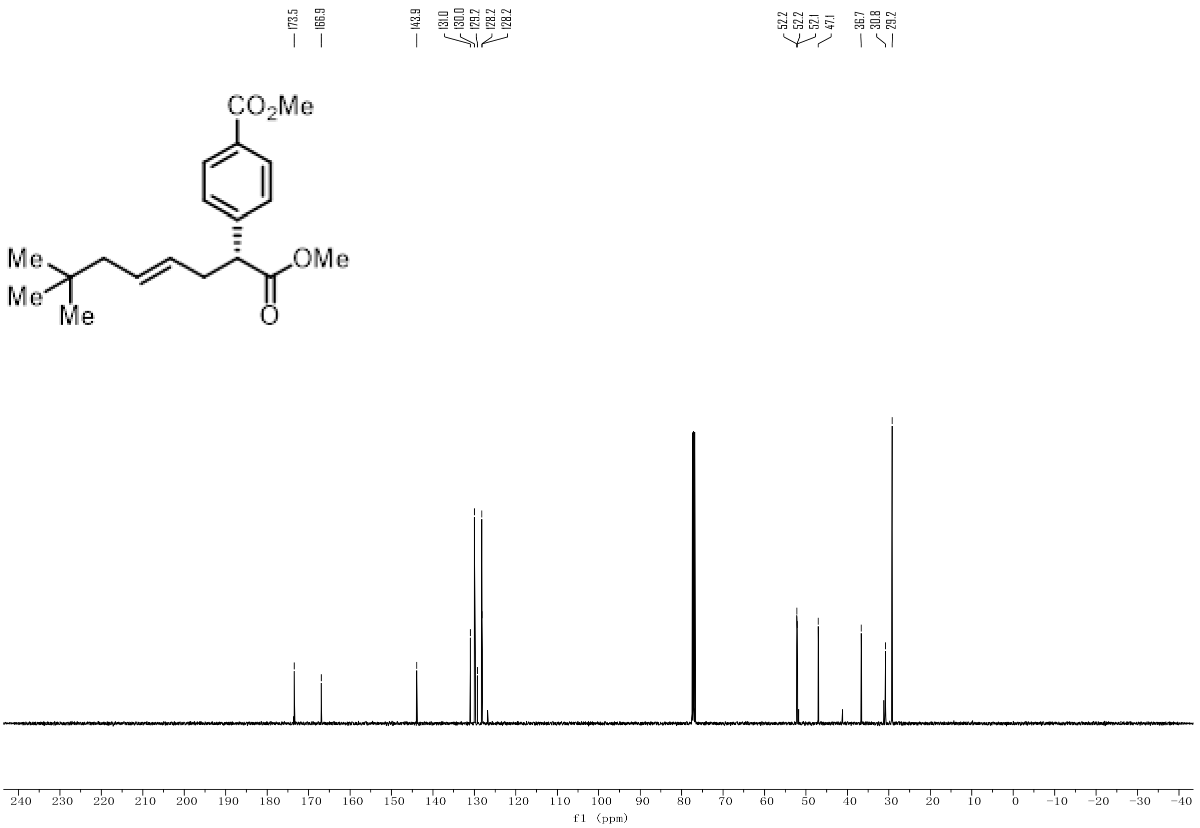

^{13}C NMR (101 MHz, $CDCl_3$, 298 K) spectrum of **68**

Methyl (*R,E*)-4-(1-methoxy-1-oxo-6-(phenylsulfonyl)hex-4-en-2-yl)benzoate (69)

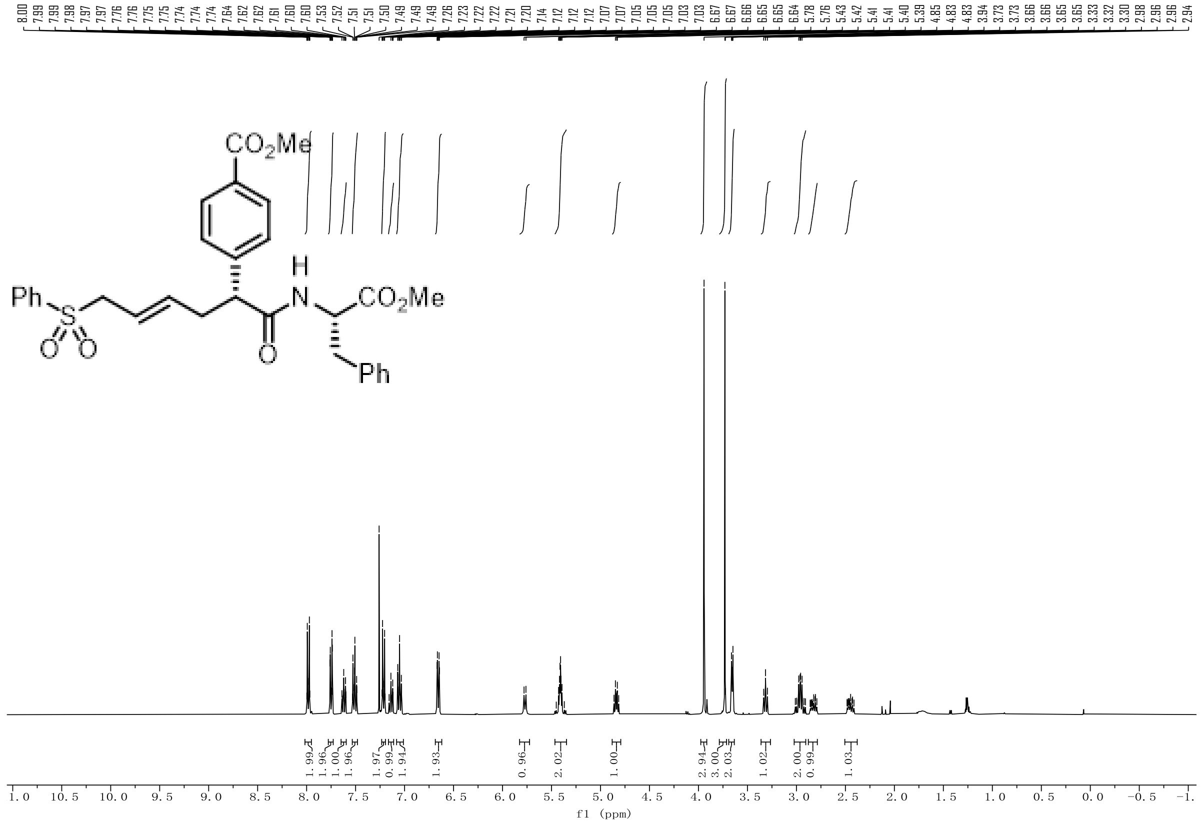

^{1}H NMR (400 MHz, $CDCl_3$, 298 K) spectrum of **69**

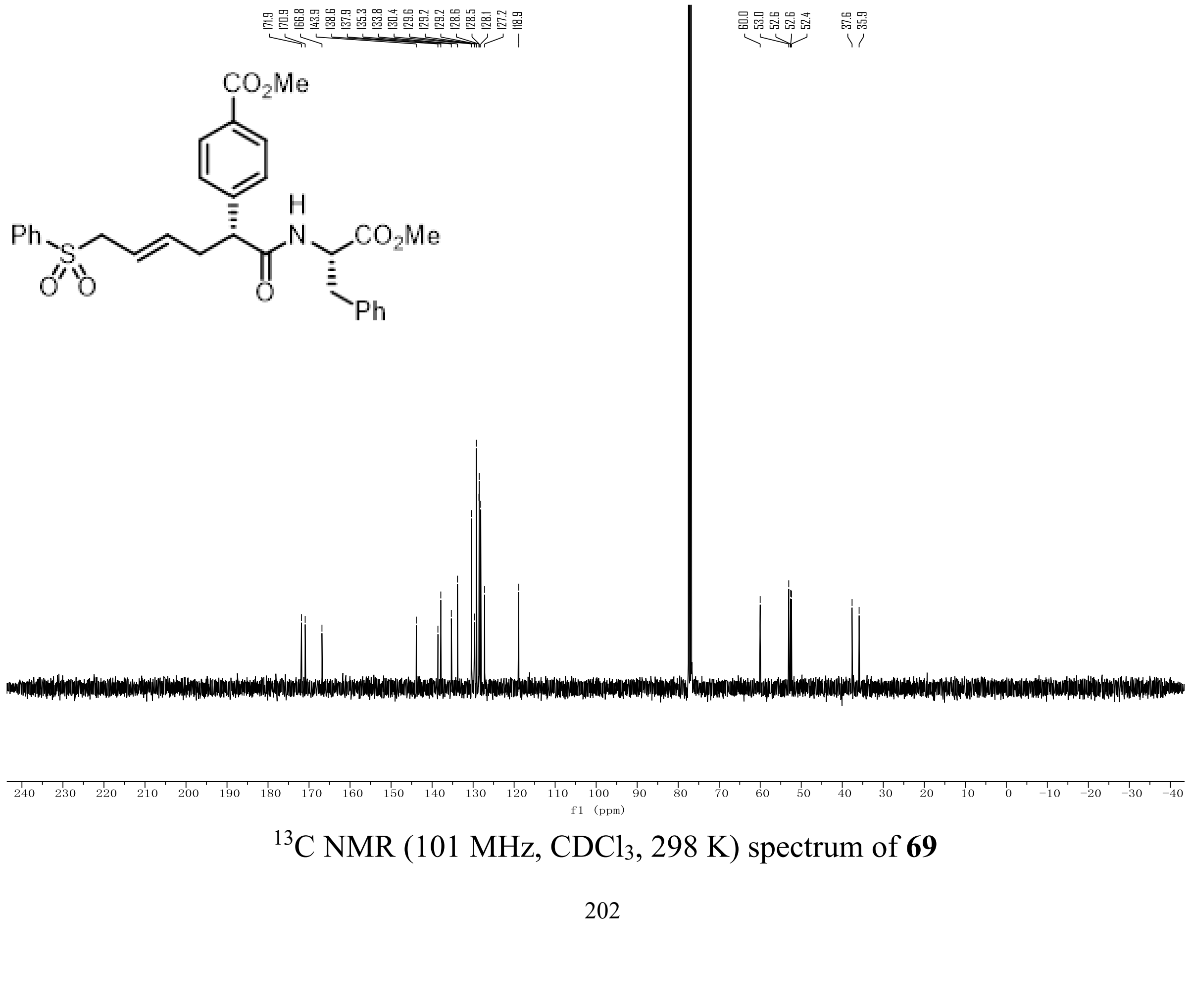

^{13}C NMR (101 MHz, $CDCl_3$, 298 K) spectrum of **69**

Methyl 4-((*R,E*)-1-(((*S*)-1-methoxy-1-oxo-3-phenylpropan-2-yl)amino)-7,7-dimethyl-1-oxooct-4-en-2-yl)benzoate (70)

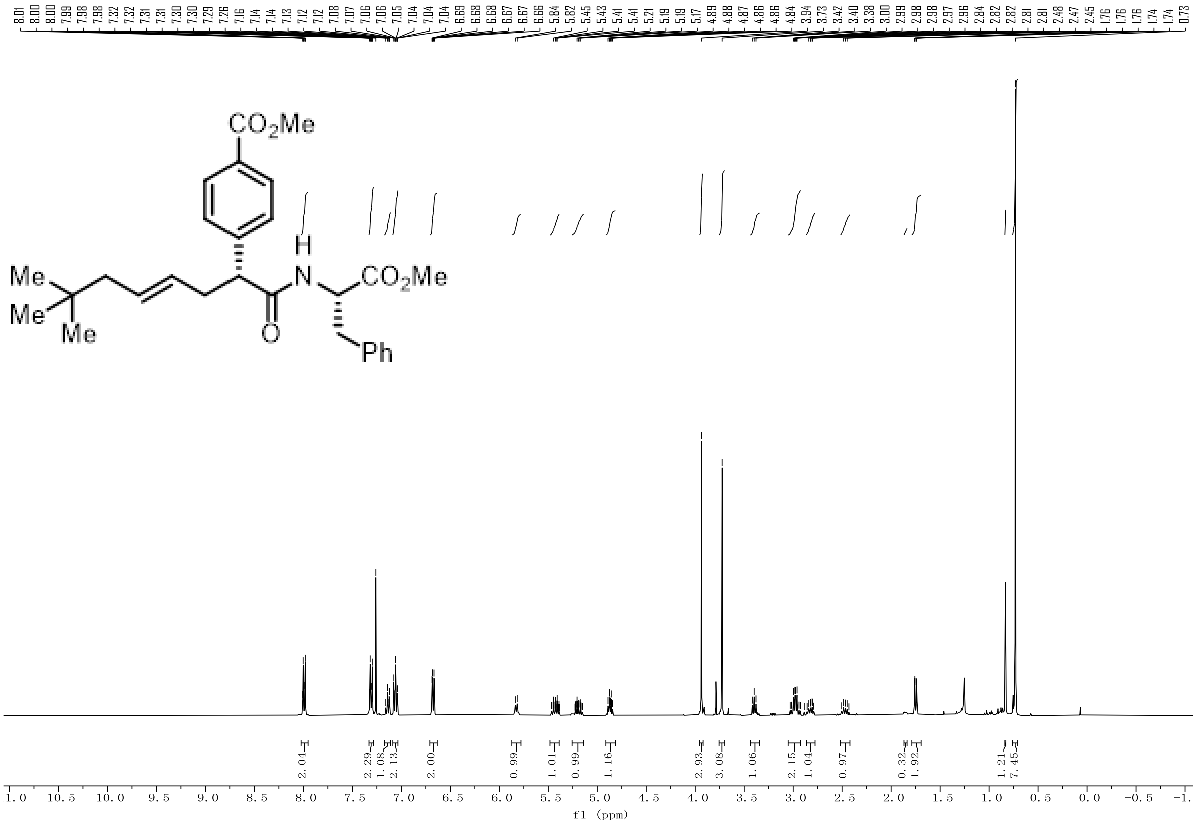

^{1}H NMR (400 MHz, $CDCl_3$, 298 K) spectrum of **70**

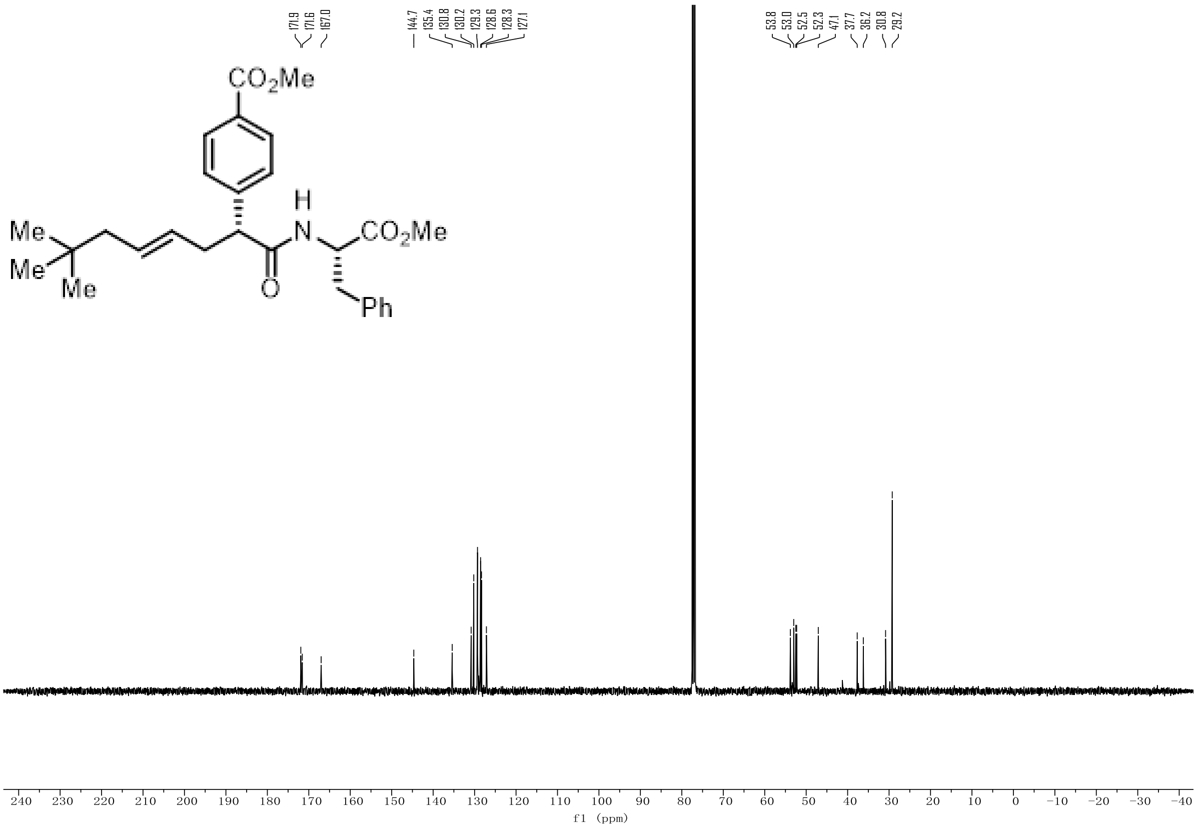

^{13}C NMR (101 MHz, $CDCl_3$, 298 K) spectrum of **70**

7. X-ray Crystallographic Data

Compound 60 (CCDC 2421141)

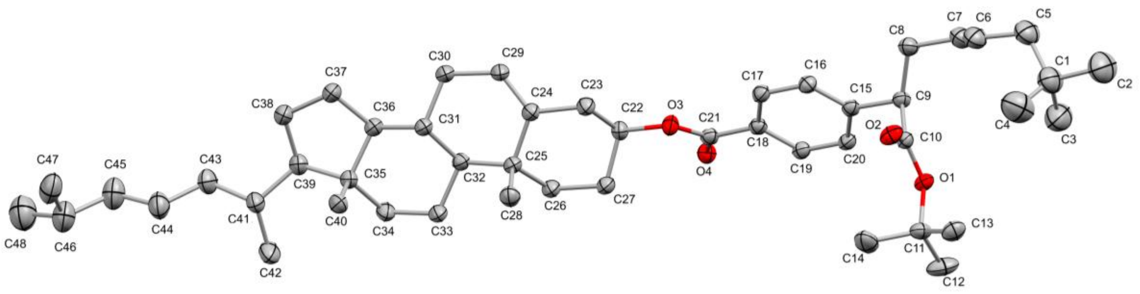

Figure S5. The molecular structure of **60** (one of the two independent molecules). Displacement ellipsoids are drawn at the 30% probability level.

Table S9. Crystal data and structure refinement for 60.

Identification code	60
Empirical formula	$C_{48}H_{74}O_4$
Formula weight	715.07
Temperature/K	160.0(1)
Crystal system	monoclinic
Space group	C2
a/Å	32.9922(4)
b/Å	6.15300(10)
c/Å	45.1978(7)
α/°	90
β/°	104.629(2)
γ/°	90
Volume/Å^3	8877.8(2)
Z	8

ρ_{calc}g/cm^3	1.070
μ/mm^{-1}	0.503
F(000)	3152.0
Crystal size/mm^3	0.22 × 0.04 × 0.02
Radiation	Cu Kα (λ = 1.54184)
2Θ range for data collection/°	5.536 to 149.002
Index ranges	$-40 \leq h \leq 40$, $-7 \leq k \leq 7$, $-56 \leq l \leq 53$
Reflections collected	94302
Independent reflections	18123 [R_{int} = 0.0705, R_{sigma} = 0.0484]
Data/restraints/parameters	18123/813/1066
Goodness-of-fit on F^2	1.038
Final R indexes [I>=2σ (I)]	R_1 = 0.0653, wR_2 = 0.1782
Final R indexes [all data]	R_1 = 0.0817, wR_2 = 0.1931
Largest diff. peak/hole / e Å^{-3}	0.41/-0.33
Flack parameter	-0.08(12)

8. References

1. Luan, Y.-Y.; Li, J.-Y.; Shi, W.-Y.; Zhang, Z.; Jiao, R.-Q.; Chen, X.; Liu, X.-Y.; Liang, Y.-M., Ruthenium-Catalyzed Difunctionalization of Vinyl Cyclopropanes for Double m-C(sp2)-H/C-5(sp3)-H Functionalization. *Org. Lett.* **26**, 3213-3217 (2024).
2. Bray, C. D.; de Faveri, G., Stereocontrolled Synthesis of trans-Cyclopropyl Sulfones from Terminal Epoxides. *J. Org. Chem.* **75**, 4652-4655 (2010).
3. Chen, C.; Shen, X.; Chen, J.; Hong, X.; Lu, Z., Iron-Catalyzed Hydroboration of Vinylcyclopropanes. *Org. Lett.* **19**, 5422-5425 (2017).
4. Verbicky, C. A.; Zercher, C. K., Olefin cross-metathesis in the preparation of polycyclopropanes: formal synthesis of FR-900848. *Tetrahedron Lett.* **41**, 8723-8727 (2000).
5. Popov, A. G.; Viviani, V. R.; Skumial, P.; Jefferson, T. L.; Salman, S. G.; Baxter, H. H.; Hull, K. L., Copper-Catalyzed Three-Component 1,5-Carboamination of Vinylcyclopropanes. *Org. Lett.* **26**, 4621-4625 (2024).
6. Payne, G. B., Cyclopropanes from reactions of ethyl dimethylsulfuranylideneacetate with .alpha.,.beta.-unsaturated compounds. *J. Org. Chem.* **32**, 3351-3355 (1967).
7. Blankley, C. J.; Sauter, F. J.; House, H. O., Crotyl diazoacetate. *Org. Syn.* **49**, 22 (1969).
8. Martín-Vilà, M.; Hanafi, N.; Jiménez, J. M.; Alvarez-Larena, A.; Piniella, J. F.; Branchadell, V.; Oliva, A.; Ortuño, R. M., Controlling π-Facial Diastereoselectivity in the 1,3-Dipolar Cycloadditions of Diazomethane to Chiral Pentenoates and Furanones: Enantioselective Stereodivergent Syntheses of Cyclopropane Hydroxy Acids and Didehydro Amino Acids. *J. Org. Chem.* **63**, 3581-3589 (1998).
9. Salikov, R. F.; Trainov, K. P.; Levina, A. A.; Belousova, I. K.; Medvedev, M. G.; Tomilov, Y. V., Synthesis of Branched Tryptamines via the Domino Cloke–Stevens/Grandberg Rearrangement. *J. Org. Chem.* **82**, 790-795 (2017).
10. Alvarez, E. M.; Bai, Z.; Pandit, S.; Frank, N.; Torkowski, L.; Ritter, T., O-, N- and C-bicyclopentylation using thianthrenium reagents. *Nat. Synth.* **2**, 548-556 (2023).
11. Huang, H.-M.; Bellotti, P.; Erchinger, J. E.; Paulisch, T. O.; Glorius, F., Radical Carbonyl Umpolung Arylation via Dual Nickel Catalysis. *J. Am. Chem. Soc.* **144**, 1899-1909 (2022).

12. Liu, S.; Robert, F.; Landais, Y., Dual photoredox nickel-catalyzed silylation of aryl/heteroaryl bromides using hydrosilanes. *Chem. Commun. (Cambridge, U. K.)* **59**, 11369-11372 (2023).

13. Cheung, C. W.; Zhurkin, F. E.; Hu, X., Z-Selective Olefin Synthesis via Iron-Catalyzed Reductive Coupling of Alkyl Halides with Terminal Arylalkynes. *J. Am. Chem. Soc.* **137**, 4932-4935 (2015).

14. Cuesta-Galisteo, S.; Schörgenhumer, J.; Hervieu, C.; Nevado, C., Dual Nickel/Photoredox-Catalyzed Asymmetric Carbamoylation of Benzylic C(sp3)−H Bonds. *Angew. Chem. Int. Ed.* **63**, e202313717 (2024).

15. Yang, T.; Xiong, W.; Sun, G.; Yang, W.; Lu, M.; Koh, M. J., Multicomponent Construction of Tertiary Alkylamines by Photoredox/Nickel-Catalyzed Aminoalkylation of Organohalides. *J. Am. Chem. Soc.* **146**, 29177-29188 (2024).
